# Bioinspired peptide cyclisation enables exploration of cytochrome P450 catalysed sequential oxidation in rufomycin biosynthesis

**DOI:** 10.1039/d6sc01721e

**Published:** 2026-08-05

**Authors:** Jessica Peate, Yaoyu Ding, Joshua Tomkins, Harry R. Winter, Jingwen Zhang, Cameron M. L. Coleman, Priya Solanki, Jake H. Nicholson, Toby Hopkinson, Gustavo Perez-Ortiz, Alex P. S. Brogan, Timothy D. McHugh, Robert E. Jefferson, Sarah M. Barry

**Affiliations:** a Department of Chemistry, King's College London Britannia House, 7 Trinity St. London SE1 1DB UK sarah.barry@kcl.ac.uk; b University College London, Centre for Clinical Microbiology Royal Free Campus, Rowland Hill St. London NW3 2QG UK

## Abstract

Natural product biosynthetic cytochrome P450s (CYPs) are of increasing interest for their incredible ability to selectively activate inert C–H bonds on highly complex substrates. However, these enzymes are understudied due to difficulties accessing substrates. Here, we use our recently developed peptide cyclisation chemistry to produce a library of natural product peptide analogues to study the rufomycin tailoring CYP, RufM. Rufomycins are antimicrobial non-ribosomal cyclic peptides that undergo sequential alkyl oxidation catalysed by RufM. This transformation is essential for rufomycin antimycobacterial activity but results in several naturally produced derivatives, of which one intermediate has the greatest activity. Due to current interest in engineering the rufomycin pathway to generate derivatives for improved properties, we use our peptide library to explore the complex interplay of enzyme catalysed and spontaneous transformations involved in RufM oxidation, suprising RufM substrate promiscuity and the role of a mechanistically important residue in sequential oxidation. Our work demonstrates the power of our chemistry both as a tool to study biosynthetic tailoring CYPs as potential late-stage functionalizing biocatalysts and to enable antibiotic development.

## Abstract 2

Chemoenzymatic route to rufomycin analogues reveals fundamental insight into RufM catalysed sequential oxidation and surprising substrate tolerance.

## Graphical abstract

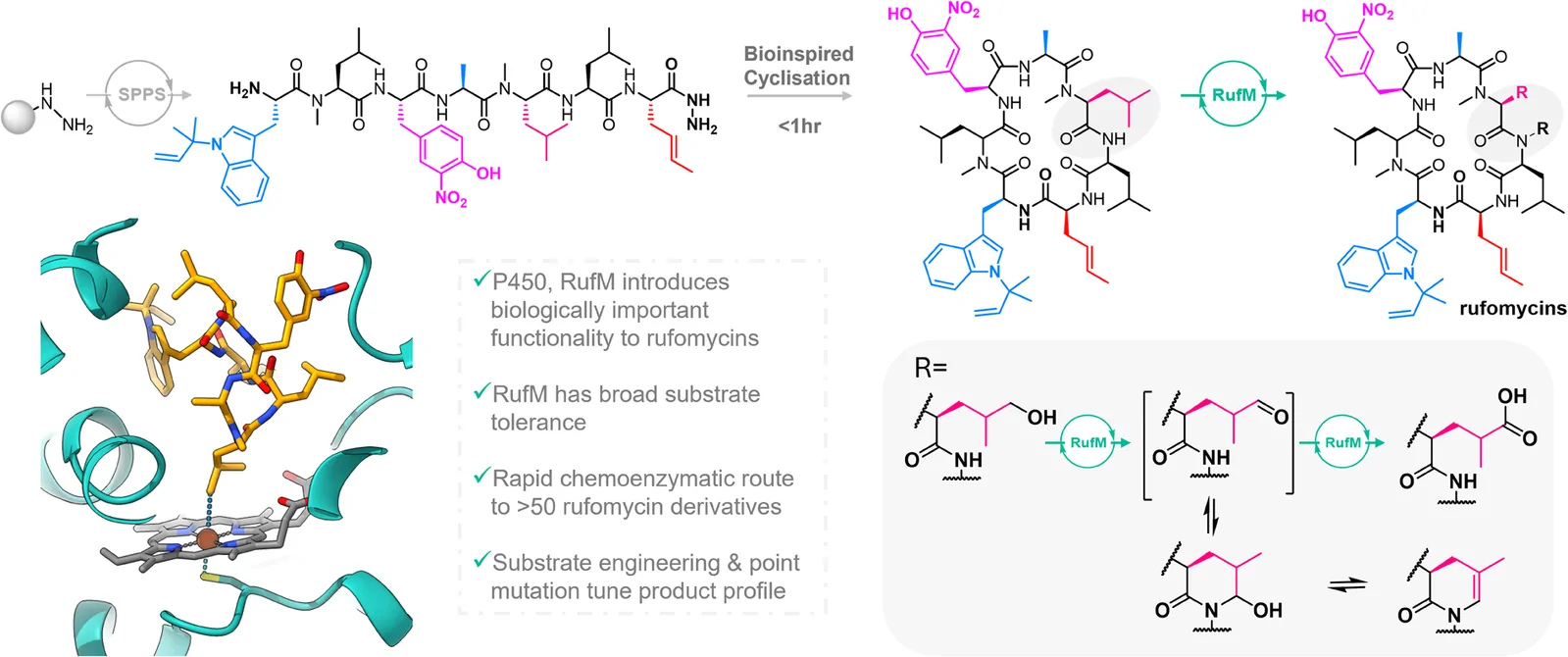

## Introduction

Cytochromes P450 (CYPs) are ubiquitous heme dependent enzymes that catalyse activation of inert C–H bonds, providing access to many synthetically challenging oxidative transformations.^1^ CYP catalysed stereo- and regiospecific oxidation is a frequent feature of bioactive natural product biosynthesis, including clinically important antibiotics, *e.g.* vancomycin,^2^ rifamycin.^3^ These so called tailoring CYPs frequently introduce functionality on elaborate substrates including peptides, polyketides and terpenes, resulting in improved biological activity.^4–6^ However, due to challenges accessing structurally complex substrates, synthetically or from natural sources, biosynthetic tailoring enzymes such as CYPs, are understudied with respect to substrate tolerance and structure, hampering efforts to engineer biosynthetic pathways or harness such enzymes as late-stage functionalising biocatalysts.^7,8^

Rufomycins are antimycobacterial cyclic heptapeptides (also known as ilamycins) produced by several strains of *Streptomyces atratus*.^9–13^ Rufomycins have been of recent interest, as rising multidrug resistance against frontline tuberculosis (TB) antibiotics such as rifampicin and isoniazid has revealed the urgent need for new antimycobacterials.^14^ Rufomycins are biosynthesised by a non-ribosomal peptide synthetase (NRPS, RufT),^9,15^ a modular, multi-domain enzyme that incorporates non-proteinogenic amino acids, catalyses *N*-methylation of the peptide backbone and cyclisation to produce rufomycin B (Fig. 1A and S4, S5).^16^ RufM, sequentially oxidises a leucine residue and in concert with epoxidising CYP, RufS, produces multiple oxidised derivatives in culture, the most potent of which are the bicyclic rufomycins (*e. g.* rufomycin A.), resulting from the intriguing spontaneous cyclisation of the aldehyde intermediate (Fig. 1A).^9^ However, the major product in bacterial extract is the less active carboxylic acid *e.g.* rufomycin C.^9,17–19^

**Fig. 1:**
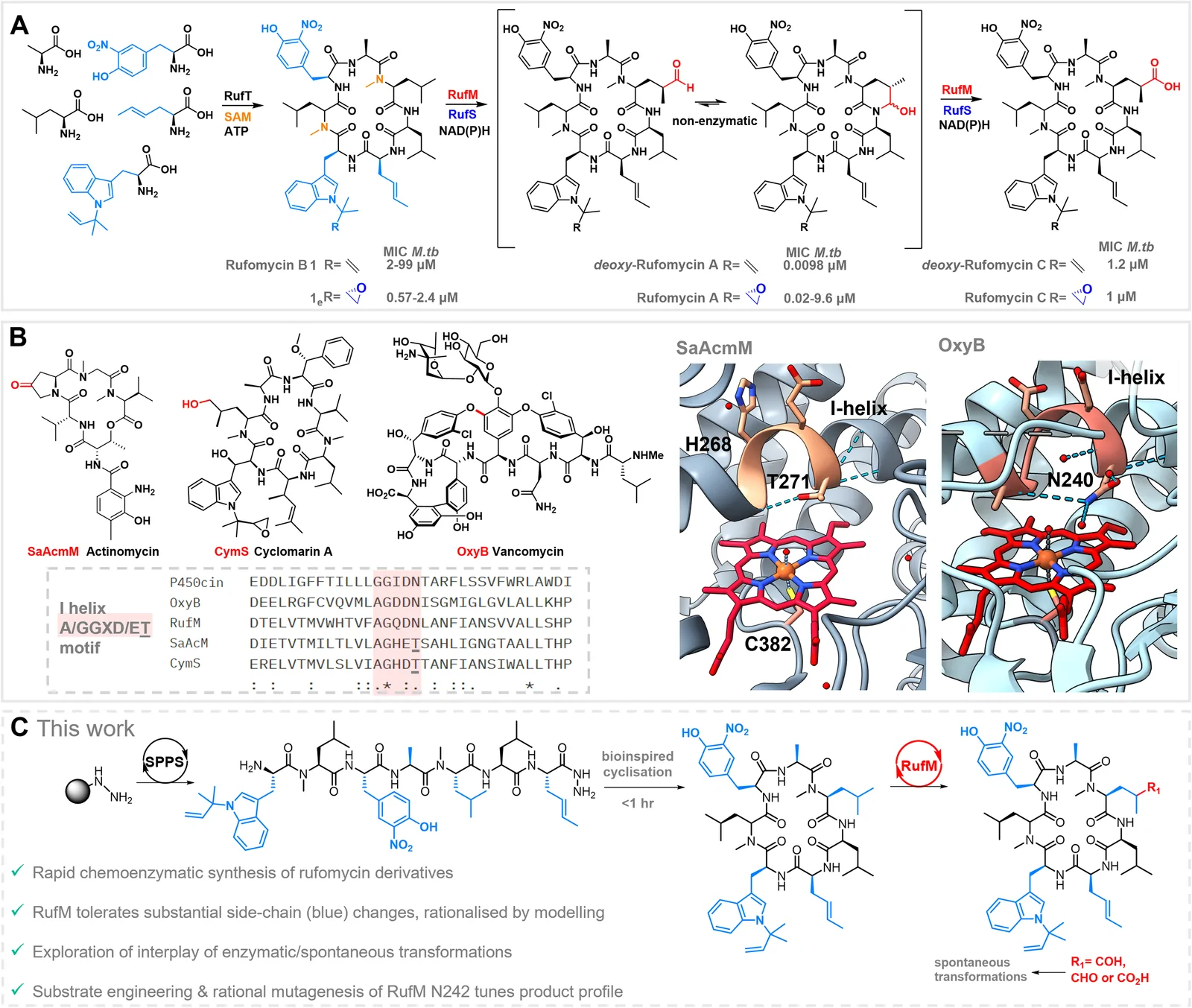
Cytochrome P450s as peptide tailoring enzymes. (A) Summary of rufomycin biosynthesis including RufT catalysed cyclic peptide synthesis including incorporation of non-proteinogenic amino acids (blue) SAM dependent *N*-methylation of peptide backbone (orange). RufM oxidation of leucine and epoxidation by RufS and MIC values against *M. tuberculosis*.^9,13,20,21^ (B) Biosynthetic CYPs, CymS (44% identity) cyclomarin A, SaAcmM (43.5% identity) actinomycin, OxyB (30.6%) vancomycin. (Inset) Alignment of CYP I-helix sequences, highlighted region shows the A/GXD/ET I-helix motif. Conserved threonine residue underlined. SaAcmM (5NWS) I-helix consensus (orange) indicating Thr to Ala backbone H-bond (2.67 Å).^22^ OxyB (1LFK) I-helix consensus (coral) indicating Asn to Ala backbone H-bond (3.47 Å).^23^ (C) This work utilises bioinspired cyclisation chemistry to produce rufomycin derivatives (residues changed in the derivative library are in blue) and reacted with RufM to understand sequential oxidation and substrate tolerance.

There have been several reported semi- and total syntheses of rufomycins to confirm the 3-dimensional structure and generate derivates for structure–activity relationship (SAR) and mechanism of action studies.^19,24,25^ However, the oxidative modifications crucial for biological activity are introduced prior to peptide synthesis through synthesis of oxidised amino acids. Derivatives can also be produced by engineering the rufomycin biosynthetic pathway, however this is challenging without understanding of RufM and RufS substrate tolerance. Thus, a chemoenzymatic, late-stage functionalisation modification of the cyclic peptide rufomycin B, would represent a concise, divergent approach that would shed light on the catalysis and substrate tolerance of RufM to inform engineering strategies.

In line with this strategy, we recently reported a method for the rapid synthesis of cyclic peptides where the linear peptide is chosen based on biosynthetic sequence.^26^ The rapid (<1 h), selective (no dimer) and high yielding reaction overcomes the key challenge of slow and inefficient peptide cyclisation.^15,26^ This facile method could be transformational by providing access to cyclic peptide substrates enabling mechanistic and substrate scope studies of cyclic peptide tailoring enzymes (Fig. 1C).

In this work we demonstrate the utility of this chemistry to produce rufomycin B 1 and a library of rufomycin analogues to understand several aspects of RufM biochemistry: (1) the interplay of enzymatic and spontaneous transformations crucial to biological activity (Fig. 1A), (2) RufM substrate tolerance, as RufM can accept epoxidized and alkene derivatives as substrates, we hypothesised it may accept other structural changes and (3) the substrate features important for catalysis and whether they align with those important for biological activity.

RufM is also one of the few characterised natural product biosynthetic tailoring CYPs that catalyse sequential oxidation of peptide substrates.^1,27^ RufM has high similarity to peptide tailoring CYPs, SaAcmM that also carries out sequential oxidation in actinomycin biosynthesis as well as CymS (60%), which catalyses just a single leucine hydroxylation in cyclomarin biosynthesis, a peptide structurally and biosynthetically analogous to rufomycin.^22,28^ Despite their different chemistry, SaAcmM and CymS maintain the so called I-helix consensus (Fig. 1B and S6). CYPs contain several catalytically important motifs including the cysteine containing loop that binds the heme cofactor and the I-helix motif A/GGXD/ET that sits over the heme where threonine forms a H-bond between the side chain and I helix backbone enabling oxygen activation *via* proton transfer (Fig. 1B).^29–31^

RufM, contains an asparagine in place of threonine in the I-helix, a change shared with P450cin, where it is proposed to control stereo and regioselectivity,^32–34^ and several OxyB homologues, involved in oxidative crosslinking in the biosynthesis of glycopeptides vancomycin, keratinimicin and teicoplanin (Fig. 1B).^35^ OxyB homologues are of interest as late-stage functionalising enzymes for chemoenzymatic glycopeptide antibiotic synthesis.^23,35,36^ Previous studies of sequentially oxidising CYPs have demonstrated that numerous factors impact the extent of oxidation including substrate, intermediate binding and partner electron transport proteins. Thus, here we investigate if the unusual I-motif residue, N242 is linked to sequential oxidation.

This work reveals the power of our peptide cyclisation chemistry to create substrate analogues to interrogate biosynthetic tailoring enzymes (Fig. 1C). It provides significant new insight into rufomycin biosynthesis and CYP biochemistry to inform pathway engineering revealing surprisingly broad substrate tolerance of RufM, and how substrate engineering and point mutation can tune product profile. We hope this will promote further study of CYPs as late-stage functionalising biocatalysts.^10,37^

## Results and discussion

Rufomycins consist of several biosynthetically expensive non-proteinogenic amino acids and peptide backbone modifications. l-Leucine is methylated while bound to RufT by SAM dependent methylation domains; 3-nitro-l-tyrosine is produced *via* CYP and nitric oxide synthase mediated nitration of a ribosomal peptide;^9,12,38^*N*-prenyl-l-tryptophan is proposed to be prenylated by a prenyltransferase and the *trans*-hexene moiety is polyketide derived (Fig. 1A and S4, S5).^9,11,15^ Our recent study of the RufT thioesterase domain indicated that these complex sidechains are not required for biosynthetic peptide cyclisation, but we do not know if they are required by RufM.^15^

Our initial biosynthetic study indicated that RufM had modest tolerance to substrate changes, as it accepted rufomycin B 1, as well as epoxidized derivatives 1e and rufomycin A, produced in partnership with RufS (Fig. 1A).^11^ However, this study was hampered by a typical problem in this field, reliance on small quantities of substrate, rufomycin B 1, extracted from bacterial culture.^11^ Fortunately, our rapid cyclic peptide chemistry provides easy access to both rufomycin B 1 and analogues to explore substrate tolerance.

Thus, non-proteinogenic amino acids *N*-prenyl-l-tryptophan and amino-*trans* hexenoic acid (not commercially available) were synthesised as previously described^26^ and linear rufomycin B 1 and a library of 17 analogues were synthesized using solid phase peptide synthesis (SPPS). Crude or purified fully deprotected linear peptides were subjected to our bioinspired cyclisation protocol giving cyclic peptides in almost quantitative conversion (Fig. 3A).^26^ Cyclic peptides were purified by semi-preparative HPLC and fully characterised by NMR and HRMS (Fig. S38).

With synthetic rufomycin B 1 in hand, our first goal was to more carefully interrogate RufM catalysis to identify transient intermediates (Fig. 2A).^26^

**Fig. 2:**
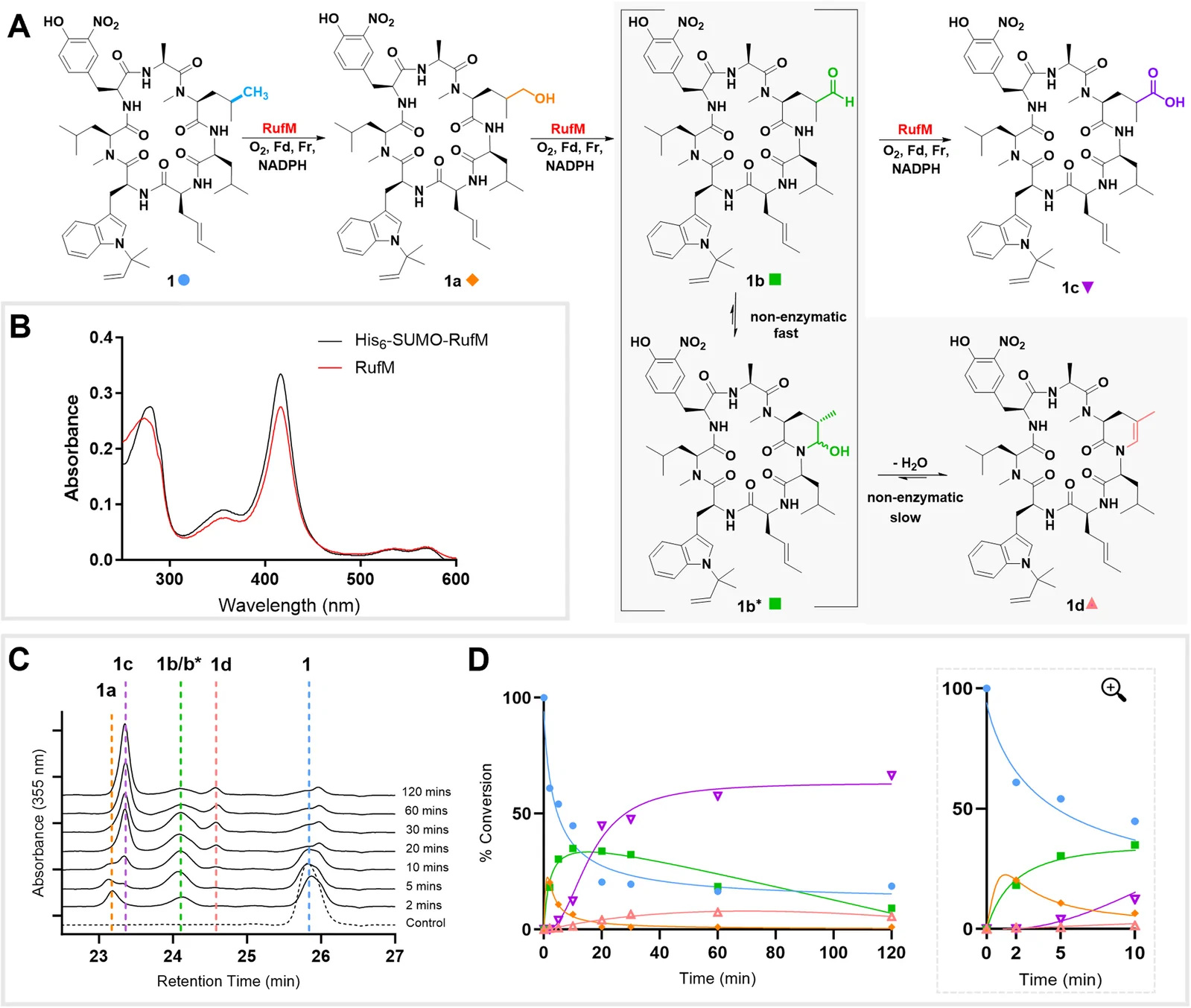
*In vitro* oxidation of the natural substrate 1 by RufM. (A) RufM reaction and all products, rufomycin B 1, alcohol 1a, aldehyde 1b in equilibrium with hemiaminal 1b* and enamine 1d. Aldehyde 1b is oxidised to carboxylic acid 1c. (B) UV-visible spectra of His_6_-SUMO-RufM (black) and following His_6_-SUMO tag cleavage (red) showing retention of heme Soret band *λ*_max_ 416 nm. (C) His_6_-SUMO-RufM catalysed conversion of 1 monitored by LC-MS (355 nm) at 30 °C for 120 minutes. Reaction contains RufM (20 µM), rufomycin B 1 (50 µM), Tris (25 mM, pH 8), ferredoxin (Fd, 45.5 µM), ferredoxin reductase (Fr, 0.04 U), NADPH (1 mM). Fd and Fr are NADPH binding and electron transport proteins. Control contains heat inactivated RufM. (D) Data from LCMS monitoring (area under the curve for each LC peak) was plotted showing conversion and consumption of 1 (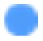), 1a (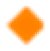), 1b/b* (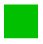), 1c (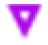), 1d (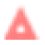), plotted using non-linear regression (Graphpad Prism, eqn (S5)).

### RufM catalysed sequential oxidation of synthetic rufomycin B: a new intermediate and a complex profile

Recombinant RufM was expressed as a *N*-terminal His_6_-tagged SUMOylated fusion protein as previously reported (Fig. 2B).^11^ His_6_-SUMO-RufM, purified by nickel affinity and size exclusion chromatography, was incubated with rufomycin B 1, spinach ferredoxin-NADP^+^ reductase, spinach ferredoxin and NADPH in Tris (pH 8) for 2 hours at 30 °C. The reaction (and all enzyme reactions in this work) were monitored initially by low resolution HPLC mass spectrometry (LCMS) and products confirmed *via* high resolution mass spectrometry (LC-HRMS) (Tables S11 and S12). Initial reactions of RufM with synthetic rufomycin B 1 show that it produces the same product distribution as previously recorded for isolated rufomycin B 1.^11^ Removal of the His_6_-SUMO tag had no impact on activity, thus all further experiments utilised only His_6_-SUMO-RufM (described below as RufM) (Fig. 2B and S10, S11). We note that the ratio of enzyme to substrate in assays is high perhaps due to product inhibition. A reaction where extracted assay products were added to fresh enzyme showed little additional turnover, leading us to suspect product inhibition by carboxylic acid 1c (Fig. S37).

To observe the kinetics of both enzymatic and non-enzymatic reactions of rufomycin B 1, a scaled-up reaction (300 µL) was monitored by LCMS analysis. Formation of alcohol 1a, as the first major product is observed within 2 minutes (Fig. 2C and D). This demonstrates for the first time that alcohol 1a is released by RufM and can rebind to allow further oxidation. Hydroxylated rufomycins have been isolated from one *S. atratus* strain.^21^ This is interesting as alcohol 1a in our experiment is rapidly consumed to form the intermediate aldehyde 1b, in equilibrium with cyclic hemiaminal 1b* (epimeric mixture), peaking at 10 min before being slowly consumed to form carboxylic acid 1c.^24^ Consumption of alcohol 1a and aldehyde 1b in the presence of the unoxidized substrate 1 indicates a preference for the oxidised intermediates over rufomycin B 1.

Additional HRMS analysis identified another product alongside 1b, peaking at 10 min. The product has an *m*/*z* consistent with loss of water from 1b, indicating formation of enamine 1d (Fig. 2A and C). Rufomycins containing this moiety have been produced semi-synthetically *via* treatment with HCL.^25^

To further investigate formation of 1d, oxidation assays were carried out using the model substrate 12. This was chosen, as reaction with RufM results in the highest conversion of the corresponding enamine 12d (25%) (Fig. 3D, H and S36). On completion of the enzymatic assay (carried out as above), sodium borohydride was added. Subsequent LCMS analysis shows reduction of aldehyde–hemiaminal 12b/b* to alcohol 12a. The enamine product 12d is reduced slightly, indicating slow interconversion between enamine and hemiaminal (Fig. S36). This slow rate of equilibrium is likely impacted by pH and may be a factor in the accumulation of the aldehyde intermediate in bacterial culture.^21^

**Fig. 3:**
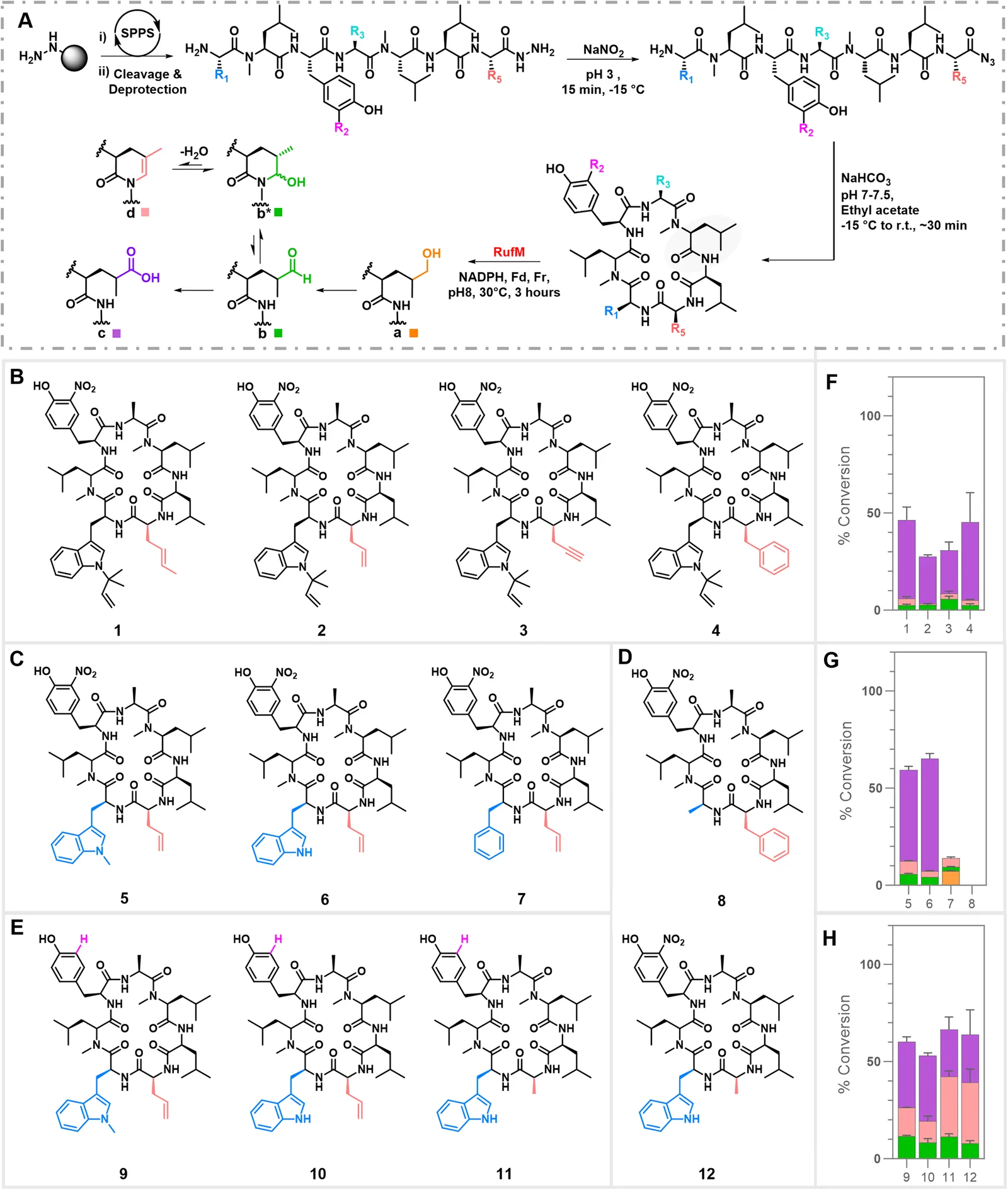
RufM substrate tolerance using a peptide library synthesized using bioinspired cyclisation. (A) Synthesis of linear peptide acyl hydrazide and bioinspired cyclisation followed by RufM catalysed oxidation.^26^R_1_–R_5_ indicate points of derivatization. Synthetic cyclic peptides 1–12 are characterised by NMR & HRMS. Enzyme assays were carried out in Tris buffer (25 mM, pH 8), His_6_-SUMO-RufM (20 µM); purified by NTA chromatography only, peptide (50 µM), ferredoxin (Fd, 45.45 µM), ferredoxin reductase (Fr, 0.04 U) and NADPH (1 mM) for 3 hours, 30 °C. Panels (B–E) peptide library screened for activity with RufM with variation at R_1_, R_2_ and R_5_. Panels (F–H) product conversions of 1–12 from RufM reactions. Bar colours correspond to the proportion of each product resulting from RufM reaction with each peptide: alcohol a (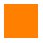), aldehyde/hemiaminal b/b* (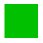), carboxylic acid, c (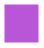), enamine, d (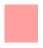). Products are predicted from LC-HRMS. Conversions (%) are average of 3 biological repeats determined from LC-MS peak area. 355 nm-for peptides 1–8 & 12, 280 nm for peptides 9–11.

Taken together, this data indicates an unusually complex CYP reaction profile including multiple oxidation steps, alongside several spontaneous transformations, with numerous species in equilibrium (hemiaminal, enamine, aldehyde, *gem*-diol). Access to synthetic substrate enabled scaled up reactions to detect transient intermediates and develop robust analytical methods to separate and identify the compounds *via* HRMS.

### Substrate screening and the impact of substrate structure on RufM product distribution

With the ability to separate and detect all possible RufM products from catalysed and spontaneous reactions, we could explore RufM substrate tolerance, including product profile changes resulting from altered substrate structure.

The synthesised peptide library 2–18 was designed to interrogate the importance of different structural features to binding and catalysis with four questions in mind (Fig. 3 and 4): the-non-proteinogenic Trp and alkene amino acids require multistep syntheses, are they essential for RufM activity and biological activity? The nitro group is essential for antimycobacterial activity, is it required by RufM for binding and catalysis? Rufomycins are highly hydrophobic, will more polar amino acids be tolerated by RufM? Will RufM tolerate changes to the site of oxidation?

**Fig. 4:**
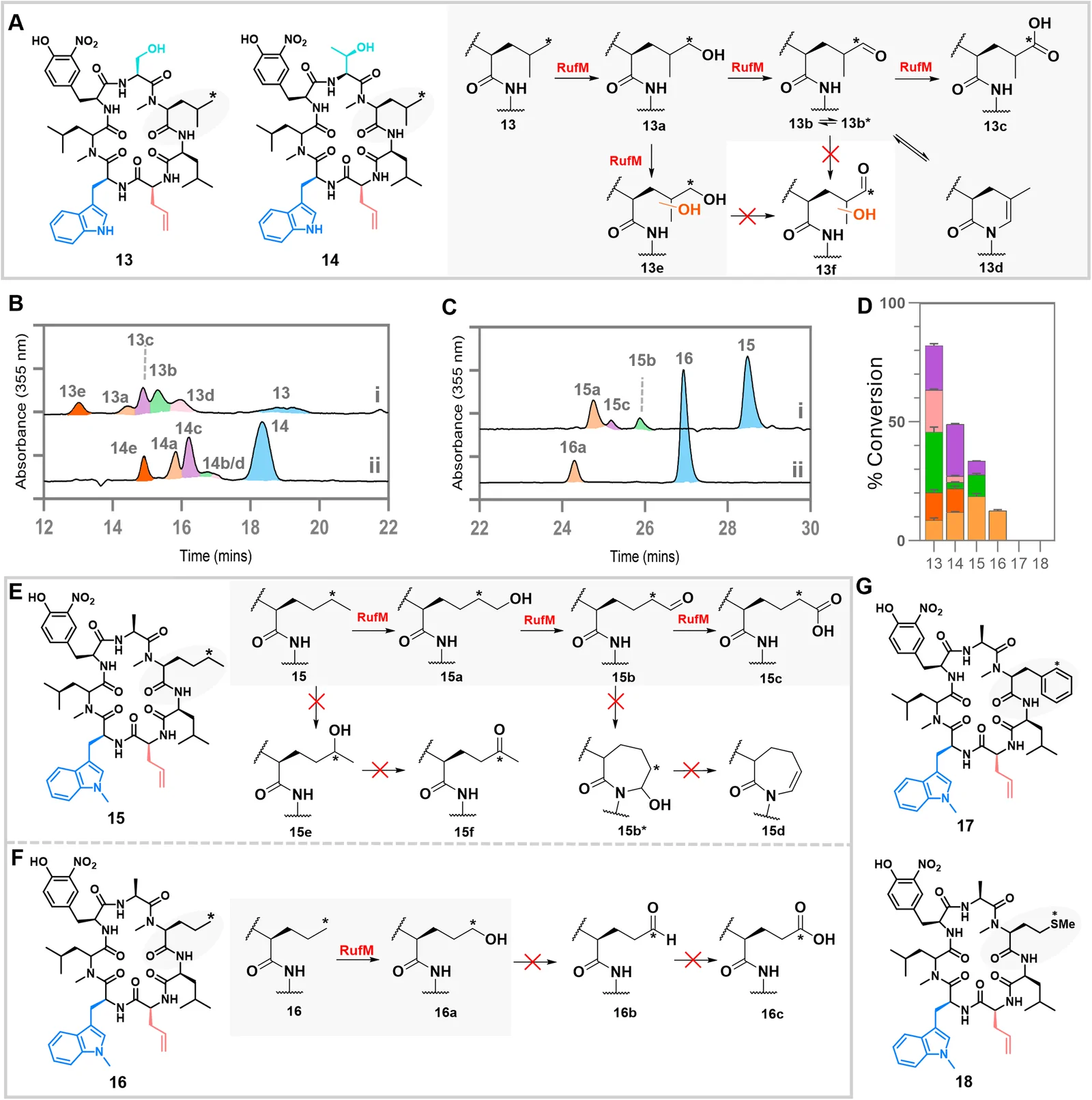
RufM catalysed oxidations of peptides 13–18. Target residue for oxidation is highlighted in grey in each structure, *indicates the expected site of oxidation based on the natural substrate. All products are assigned based on LC-HRMS data. (A) Structures of peptides 13 & 14 additionally modified at R_3_ with polar amino acids (cyan). Proposed reaction scheme for peptide 13 (and 14). Diol formation occurs on the same leucine residue based on MS^e^ fragmentation (see Fig. S22) preventing further oxidation. (B) LCMS chromatograms from analysis of RufM (purified *via* NTA only) reactions (carried out as described above) with peptide (i) 13 and (ii) 14. (C) LCMS chromatograms from analysis of RufM reactions (carried as described above) with peptide (i) 15 and (ii) 16. (D) % conversions of peptides 13–18 following RufM reaction. Bar colours correspond to the proportion of oxidised products resulting from RufM reaction with each peptide: unoxidised peptide (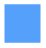), alcohol a (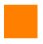), aldehyde b/aminal b* (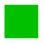), carboxylic acid, c (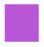), enamine, d (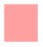), diol e (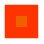). % conversions are averages of 3 biological repeats and were determined using LC peak area (355 nm) for peptides 13–16. Peptides 17 & 18 resulted in no detectable products. (E) Structure of synthetic peptide 15. Proposed reaction scheme for RufM oxidation of peptide 15. Detected products suggest oxidation at carbon 5 following a single route forming 15a, 15b and 15c. Enamine intermediate 15b* was not detected. Oxidation at carbon 4 to 15e or ketone 15f was not observed. (F) Structure of synthetic peptide 16. Proposed reaction scheme for RufM oxidation of peptide 16. Only alcohol 16a was detected. (G) Structures of synthetic peptides 17 and 18.

### Altering the specialised amino acids, *N*-prenyl-l-tryptophan and l-2-amino-*trans*-4-hexenoic acid

The natural substrate rufomycin B 1 contains the unusual non-proteinogenic, l-2-amino-*trans*-4-hexenoic acid (Fig. S4). We first tested derivatives 2–4 with variation at position R_5_, assuming that this bespoke residue would be essential for RufM substrate recognition (Fig. 3A and B).^26^

As expected, rufomycin B 1 saw the highest conversion of substrate to products amongst this group, predominately forming the carboxylic acid (40.6%) with minor aldehyde/hemiaminal (2.3%) and enamine products (3.6%). Conversion of 1 is reduced (relative to Fig. 1) as RufM was purified using only nickel affinity chromatography and any residual imidazole could bind to CYP heme iron and inhibit RufM.^39^ Additional size exclusion chromatography improves activity of the isolated enzyme, but results in aggregation and loss of protein. Thus, to enable further studies, proteins were purified using NTA only.

Assays with derivatives 2–4, reveal that RufM appears tolerant of substantial structural changes in steric bulk and functionality at R_5_. All three peptides give good overall conversion, 2 (27.4%), 3 (30.8%) 4 (45.3%) and produce similar product distributions to the natural product 1.

Following this promising result, peptides 5–8 were assessed to determine the importance of another bespoke amino acid, *N*-prenyl-l-tryptophan (Fig. 3C and D). These peptides were synthesized as analogues of 2 as l-allylglycine is commercially available, and well tolerated by RufM (Fig. 3A, C and F). Peptide 5 differs from 2 by replacing the bulky prenyl with a methyl group. Intriguingly, this resulted in an increase in total conversion (59.4%) in comparison to 2, alongside a marginal increase in intermediates 5b/b* (5.6%) and 5d (6.7%). Reducing steric bulk further by introducing unmodified l-Trp to give 6, results in greater conversion than 5 (65.2%). Interestingly, we observed a similar trend for the thioesterase (TE) domain of NRPS, RufT, that catalyses biosynthetic peptide cyclisation to form rufomycin B 1 (Fig. S5).^15^ In a substrate tolerance study of RufT-TE, we surprisingly found that an *N*-methyl-Trp containing linear peptide is a significantly better substrate for RufT thioesterase domain than the native prenylated substrate.^15^

Peptide 7, where l-tryptophan is replaced by l-phenylalanine, resulted in a major reduction in conversion (13.9%) with alcohol 7a (7.1%) as the major product and intermediates, aldehyde/hemiaminal 7b/b* (2.2%) and 7d (4.6%). Finally, peptide 8 containing alanine at R_1_ and thus a methyl group instead of the tryptophan moiety, resulted in no detectable products. Taken together the data implies that an aromatic ring (ideally an indole ring) is required at R_1_ for substrate recognition (Fig. 3C and G).

Peptide 12 has modifications to both R_1_ and R_5_ groups with reduced steric bulk at both positions (tryptophan at R_1_ and alanine at R_5_) (Fig. 3D). Peptide 12 gave greater overall conversion to products (63.8%) compared to natural peptide 1 (Fig. 3H). Excitingly, as mentioned above, the major product of 12's oxidation is hemiaminal/enamine intermediates 12b* and 12d. This implies, that RufM has reduced affinity for oxidised intermediates 12b and 12d relative to the natural substrate 1, reducing further oxidation and allowing their accumulation.

These data indicate that bespoke amino acids are not essential for RufM processing of rufomycin derivatives. To determine if the prenylated Trp and the *trans* hexene moieties are essential for biological activity, we selected 3 peptides (4, 5 and 8) for biological testing against *Mycobacterium tuberculosis* H37Rv, revealing that while all three have modest MICs (32–128 µg mL^−1^), they are comparable to rufomycin B 1 (Table S13). Thus, we conclude that the synthetically demanding residues *N*-prenyl-l-Trp and amino-hexenoic acid are likely not essential for biological activity and could be extensively modified synthetically, enzymatically or *via* pathway engineering to enable SAR studies.

### 3-Nitro-l-tyrosine is essential for rufomycin activity, but is it required for RufM catalysis?

The nitro group is important for rufomycin biological activity, but non-nitrated analogues have been isolated from culture.^21^ Peptides 9–11 were synthesized using l-tyrosine instead of 3-nitro-l-tyrosine thus generating non-nitrated analogues of 5, 6 & 12 respectively (Fig. 3C, D and E). Intriguingly, RufM appears to accept the non-nitrated derivatives in the same manner, with both sets displaying very similar overall conversion, with a small reduction in carboxylic acid formation for 9 and 10 relative to 5 and 6 (Fig. 3H and G). Peptide 11 gives identical conversion and product distribution to nitrated peptide 12. Pleasingly, this result partly explains the origin of non-nitrated oxidised rufomycin analogues isolated from *S. atratus* MJM3502. The adenylation domain in module 3 of RufT appears to be able to incorporate l-Tyr instead of 3-nitro-l-Tyr and the resulting cyclic peptide is fully processed by RufM (Fig. S5).^21^

### Introducing polar amino acids changes the regioselectivity of oxidation

Rufomycins have poor solubility in water, thus introducing more polar residues (*e.g.* serine) may be attractive for SAR studies. As this is a substantial structural change, we speculated that conversion would be low. Thus, to maximise the chances of product detection we derivatised peptide 6 as it gave maximum conversion in the initial screen (Fig. 3C). Thus, peptides 13 and 14 were synthesized with serine or threonine modifications instead of alanine (R_3_) as more hydrophilic derivatives of 6 (Fig. 3A and 4A). Fascinatingly, when assayed with RufM, peptide 13 showed the highest overall product conversion of any substrate (82%) with an intriguing product profile including formation of a new product with a mass indicating dihydroxylation (Fig. 4A, B, D and S22). The selectivity of the oxidation changed, suggesting the peptide 13 binds to RufM in multiple orientations allowing oxidation of a secondary site. Peptide 14 behaves similarly but with lower conversion (49.0%), probably due to increased steric bulk of threonine.

To identify the position of the additional oxidation, we carried out MS^e^ fragmentation of the product and analysed the fragments using mMass (Fig. S22 and Table S5).^40^ This strongly indicates that the second hydroxylation occurs on the same leucine residue at carbon 3 or 4 (Fig. 4A). Interestingly, further oxidation products of the diol 13e or 14e were not detected. Two rufomycin analogues have been previously isolated containing dihydroxylated leucine, indicating that some RufM homologues oxidise at multiple positions instead of sequential oxidation at one carbon.^20,21^

### Modifying the site of oxidation

The variety of modifications tolerated by RufM at different positions led us to explore whether we could introduce different residues in place of *N*-methyl-l-leucine at the site of oxidation (R_4_) (Fig. 3A and 4E, F). Thus, we synthesised peptides 15 and 16 to investigate the impact of reducing or increasing the length of the aliphatic chain as well as 17 and 18, to change the size and electronic properties of the substrate (Fig. 4G). Peptide 18 mimics a rufomycin derivative isolated from *S. atratus* SCSIO ZH16 Δ*ilaR* (IlaR is a homologue of RufS) where *N*-methyl-methionine is incorporated in place of *N*-methyl leucine, but the methionine side chain is oxidised to a sulfoxide.^20^ Several CYPs are known to catalyse sulfoxidation and aromatic oxidation, although for RufM to be successful a change of mechanism from aliphatic oxidation would be required.^41,42^

We first assessed peptide 15, as we hypothesised that producing a ketone on carbon 4, 15f, would be a neat way of using substrate engineering to halt further oxidation to a carboxylic acid thus accumulating the corresponding bicyclic product 15b* (Fig. 4E). However, reaction of peptide 15 with RufM, resulted in lower overall conversion than the corresponding *N*-methyl-l-leucine containing peptide 5 (Fig. 3C and 4D, E). The primary oxidation product is alcohol 15a (18.7%), with clear evidence of higher oxidation products aldehyde 15b (8.9%) and carboxylic acid 15c (5.9%) (Fig. 4D and E) proving substrate directed changes in regioselectivity to the terminal carbon 5, instead of the expected carbon 4. The intermediate appears to be aldehyde 15b rather than hemiaminal 15b*. This was inferred due to the sharp LC peak, indicating it is not an equilibrium mixture of epimers, and the corresponding enamine 15d was not detected. The absence of 15b* is due to 7 membered ring formation being unfavourable (Fig. 4E).

†13 and 14 product profiles resulted in poor HPLC separation (Fig. S19 and S20).To determine the impact of a less sterically bulky, shorter sidechain at R_4_, peptide 16 containing *N*-methyl-l-norvaline was assayed with RufM. It results in much lower conversion than 15 or 5 (leucine derivative) producing only alcohol 16a (Fig. 4C, D and F). This data indicates a key hydrophobic interaction between the branched leucine CH_3_ and the protein is important for binding and catalysis.

Peptides 17 and 18, containing *N*-methyl-l-phenylalanine or *N*-methyl-l-methionine respectively, were not accepted as substrates by RufM. It was unclear if this resulted from poor binding or because RufM is unable to effect the change in mechanism required to carry out sulfoxidation or aromatic oxidation (Fig. 4D and G).^41,43^ However, the result is in line with the lack of further oxidation of diol 13e and 14e indicating that increased polarity at this position are not tolerated (Fig. 4A). It also suggests that the sulfoxidation of methionine containing rufomycin derivatives from *S. atratus* SCSIO ZH16 Δ*ilaR* is spontaneous or the result of non-specific oxidases rather than RufM.^20^

### Understanding the substrate tolerance of RufM

The surprising results from this series prompted us to understand more about RufM substrate binding. Our early efforts to observe binding of rufomycins to RufM were hampered by the nitro moiety obscuring heme absorbance in binding assays monitored *via* UV-visible spectroscopy. The method relies on a substrate displacing the distal water molecule bound to heme-Fe(iii) resulting in a change in Fe-heme geometry, shifting the Soret band of the heme cofactor from ∼420 nm to ∼390 nm (Fig. 5A).^44^ However, with non-nitrated rufomycin derivatives 9–11 in hand we could revisit substrate binding.

**Fig. 5:**
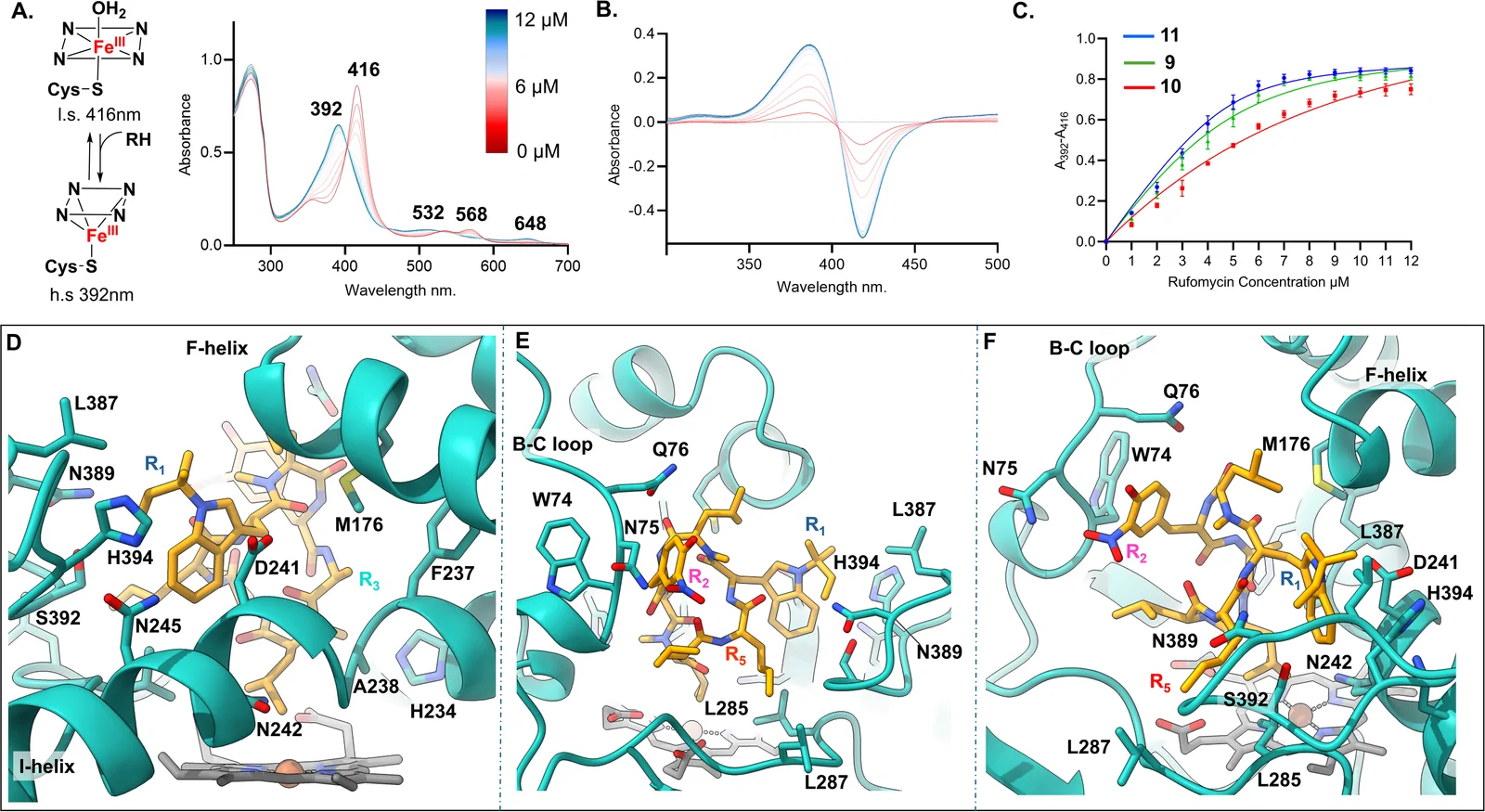
Binding of rufomycins to RufM. *K*_D_ measurement and effect of peptides 9, 10 and 11 on the visible absorption spectrum of RufM. (A) Change in heme complex (porphyrin ring represented by four nitrogens) on substrate binding, low spin (l. s.) to high spin (h. s.). Chromatogram shows titration of 5 µM RufM with peptide 11 (0–12 µM) in Tris buffer (25 mM, pH 8). Monitored by UV-visible spectroscopy. (B) Difference spectra Δ*A*_392–416_ for RufM binding to 11. (C) RufM binding curves (0–12 µM). For 9 (*K*_D_ 1.18 µM ± 0.26); 10*K*_D_ (3.34 µM ± 0.72) and 11 (*K*_D_ 0.67 µM ± 0.13) (Fig. S18). (D–F) Views of rufomycin B 1 docked RufM model generated using AlphaFill and RosettaLigand. The oxidation site is 2.92 Å from heme-iron in line with solved crystal structures (Fig. 6F).^45,46^ Naming of structural features as per alignment (Fig. S40), naming of rufomycin structural features as per Fig. 3A.

The UV-visible spectrum of RufM is typical of the CYP family.^29,47^ In the presence of an increasing concentration of rufomycin 11, the heme Soret band at 416 nm (low spin heme iron) shifts to 392 nm (high spin heme iron) producing a type I spectrum.^45–47^ The same spectral change was observed for peptides, 10 and 9 (Fig. 5A, B and S13–S15, S18). *K*_D_ values were estimated in the low µM range (0.6–3.5 µM) and follow the same trend as the conversions (Fig. 3H, 5C and S18). These values, not unusual for CYPs (*e.g.* P450_bit_,^30^ TamI^31^), provide binding constants for the unoxidized substrates but may not correlate to oxidised intermediates.^30^ For example, lower conversion of 11 (and 12) to carboxylic acid products relative to 10 indicates that the hexenoic acid moiety sidechain is more important for strong binding of intermediate oxidation products than the initial substrate (Fig. 3C and H). The low µM *K*_D_'s also tempted us to determine if the absence of activity of RufM with peptides 17 and 18, derivatised at the point of oxidation (Fig. 4A), was due to their inability to bind to RufM. The titration is distorted by the absorbance of the peptides (300–355 nm), but the qualitative data is sufficient to show that there is no binding of 17 or 18 to RufM (Fig. S25), confirming that RufM cannot accommodate the methionine or phenylalanine sidechains, preventing substrate binding and catalysis.

To further rationalise RufM's substrate tolerance, we created a model of RufM complexed to heme and docked the natural substrate. The model was created using AlphaFill, a deep learning method for transplanting small molecule ligands into protein structures predicted by AlphaFold.^48^ After docking refinement of heme, the loop from residues 70–79 (Fig. S39 and S40) had low structural confidence and was removed for subsequent docking of rufomycin B 1. The top-scoring docked structures were filtered for interatomic distance between the gamma carbon of leucine (site of oxidation, R_4_) and the heme iron and the model with the best interface score was selected as the final docked substrate pose. The removed loop residues were remodelled around this pose.

The model (built without knowledge of the experimental data) shows crucial substrate binding detail that aligns with our experimental results (Fig. 5D–F and S39). The terminal carbon of leucine points directly towards the heme at 2.92 Å, in line with solved crystal structures of *e.g.* EryF (Fig. 6F).^45^ In the absence of the branched leucine side chain, there is space for the longer chains to bend enabling access to the terminal carbon thus explaining regioselective changes in oxidation of peptide 15 (Fig. 4C and E).

**Fig. 6:**
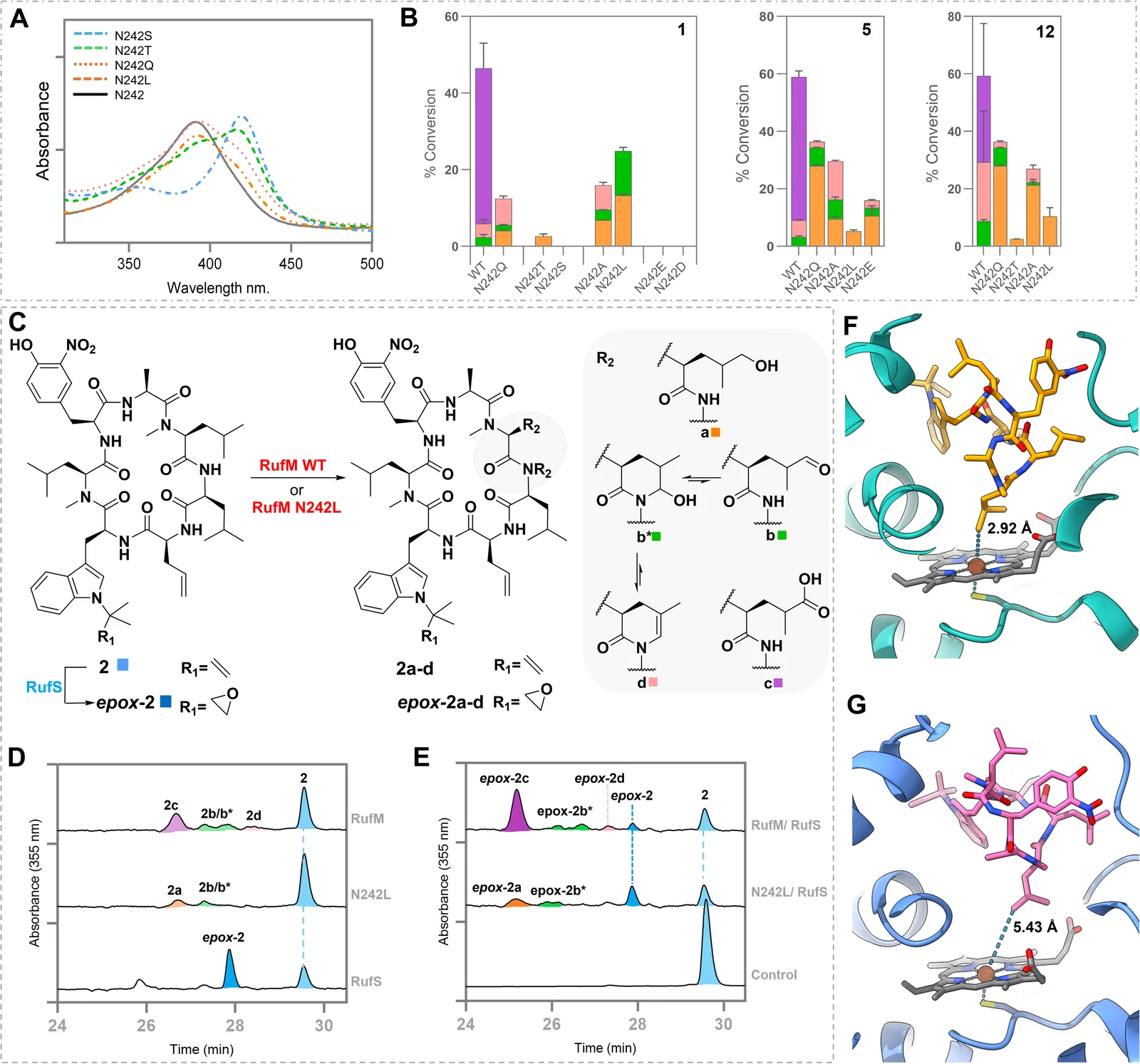
Rational mutagenesis of RufM. (A) UV-visible spectra (310–500 nm) of RufM mutants (5 µM, normalized for heme content) upon the addition of peptide 11 (12 µM). N242 (grey solid), N242L (orange), N242Q (pink), N242T (green), N242S (blue). (B) Screening results for RufM WT and N242 mutants with peptides 1, 5 and 12 (Fig. 3). % conversions, determined using LC peak area at 355 nm, are averages of 3 biological repeats. Bar colours correspond to the proportion of product resulting from RufM reaction: alcohol a (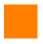), aldehyde b/hemiaminal b* (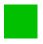), carboxylic acid, c (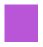), enamine, d (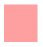). (C) Reaction of RufM WT or RufM–N242L and RufS with peptide 2 (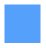), inset shows structures of oxidation products colour coded as for panel (B). (D) LC-MS chromatograms for RufM, N242L and RufS with the peptide 2 (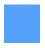) (355 nm). Reactions performed as described in Fig. 3A. Colours are as for panel (B and C). (E) LC-MS chromatograms for RufM/RufS and N242L/RufS with peptide 2 (355 nm). Cascade reactions: Tris buffer (25 mM, pH 8) with RufM WT or N242L (20 µM), RufS (20 µM), peptide 2 (50 µM), ferredoxin (Fd, 90.90 µM), ferredoxin reductase (Fr, 0.08 U), NADPH (1 mM), 3 hours. Control contains heat inactivated RufM/RufS. (F) Model of rufomycin B 1 bound to RufM. (G) Model of rufomycin B 1 bound to RufM N242L.

The nitro group (R_2_) appears to point into solvent with a single polar interaction with the sidechain of N75 explaining the lack of significant impact on binding or catalysis of exchanging the 3-nitrotyrosine for tyrosine (Fig. 5E and F). The tryptophan moiety (R_1_) packs against D241, N242 (J helix, Fig. S40), and L285, L387, S392, and H394 in more unstructured regions (Fig. S40). However, the prenyl group appears to point towards the solvent explaining why its absence is broadly tolerated (Fig. 3 and 5D). Similarly, the *trans*-hexene moiety (R_5_) packs against L285, but otherwise has a large volume around it, explaining the tolerance for change to the aryl sidechain of 4 (R_5_, Fig. 3A, B and 5E). Finally, alanine points towards a surface formed between residues M176 and F237/A238 of the I-helix (R_3_, Fig. 5D and S39). Interestingly, it is located near H234 (I helix) perhaps enabling an additional polar/H-bonding interaction with serine or threonine in peptides 13, 14 that may explain their status as excellent substrates (Fig. 4, 5D and S39).

### Mutating N242 residue abolishes conversion to carboxylic acid

With an understanding of RufM binding and demonstration of its extraordinary substrate tolerance, we turned to investigating the unusual I-helix residue, N242, and its impact, if any, on sequential oxidation. RufM is one of a few characterised biosynthetic tailoring CYPs that catalyse sequential oxidation on peptides (*e.g.* TxtC, MycG, BiolI, PikC).^1,27,49–52^ CYPs have highly conserved tertiary structures including the catalytically important I-helix motif A/GGXD/ET that sits over the heme cofactor.^29^ The threonine residue has been shown to be important as a proton shuttle to enable O–O bond cleavage to generate the reactive oxygen species, compound I.^30^ RufM deviates from the I-helix consensus sequence with asparagine replacing threonine as in P450cin, OxyB among others (Fig. 1E).^23,35,36^ The crystal structure of P450cin shows that N242 forms a H-bond to oxygen in the substrate and mutation results in loss of stereo- and regioselectivity.^32–34^

To investigate the influence of RufM N242 on catalysis and product distribution, seven mutant proteins with different properties were generated: amide (N242Q), aliphatic (N242L, N242A), carboxylate (N242D, N242E) and hydroxyl mutants (N242S, N242T). We observed variation in heme incorporation and the Soret *λ*_max_ for each mutant indicating significant changes around the heme active site. N242S was purified with very low heme incorporation compared to the wild type (WT : N242S = 1 : 0.15) (Fig. S8, S12 and Table S3).

The mutants were first assayed with natural substrate 1 (Fig. 6B). Interestingly, as for P450cin, replacing asparagine with threonine to restore the I-helix consensus resulted in almost no activity. The serine mutant is also inactive.^32,33^ This contrasts with EryF, where changing the non-canonical alanine residue in this position to threonine resulted in increased substrate tolerance (Fig. 6B).^45,53^ The carboxylate mutants, N242E and N242D, were also inactive. However, N242Q, N242A and N242L were excitingly found to only form intermediate oxidation products with N242L forming highest levels of the target intermediate 1b/b* (Fig. 6B). This is surprising as, while N242 and N242Q could act like threonine *i.e.* H-bonding and forming a proton shuttle to bound dioxygen,^54^ and N242A would leave space for a water molecule to act *in lieu* of the glutamine sidechain, it is unlikely that leucine (N242L) could act in this manner. This suggests that the N242 residue may have an alternative mechanistic role, as reported for EryF and P450cin.^32,53^

To gain more insight into mutant substrate tolerance, peptides 3, 4, 5, 8, 11, 12, 17 and 18 were additionally screened with selected mutants (Fig. 6B and S30–S35). Peptides 8, 17 and 18 were chosen because they were not converted by the WT, 5 because it gave highest conversion and 12 because it produced the highest proportion of the target intermediate aldehyde/hemiaminal. N242T was largely inactive however all others produced primarily alcohol products compared to N242, indicating that this residue is important for successful binding of the intermediate oxidation states to enable sequential oxidation. Peptides 8, 17 and 18 (Fig. S25 and S33) were not turned over by any mutants, while peptides 3 and 4 were only converted by WT and N242A resulting in roughly equal proportions of intermediate products (Fig. S30 and S31). Interestingly, each mutant behaved differently with the substrates suggesting the exciting possibility that substrate engineering and minimal mutation enables RufM tuning towards a target substrate or preferred product profile (Fig. 6B and C).

To investigate further, substrate binding of RufM mutants was assessed using model non-nitrated substrate 11 (Fig. 3 and 6A) as assays with peptide 11 gave a similar product profile to the corresponding nitrated peptide 12 (Fig. 6A and S13–S17, S35). WT N242 fully converts to high spin iron(iii) (392 nm) in the presence of 11 (12 µM). N242S, remains low-spin indicating that its inactivity is due to loss of substrate binding. However, while N242T produces ∼50% high spin, both N242L and N242Q almost fully convert to high spin (Fig. 6A). This indicates that N242L should have equivalent activity to N242Q, but while N242Q displays good conversion of substrates 11 and 12, N242L (and N242T) give low conversion to the alcohol product (Fig. 6A, B, S15, S16, S34 and S35). It is worth noting that the distal water does not have to be completely displaced for activity to occur.^55^

The low activity of N242T is surprising, as the I-helix H-bond should be maintained (Fig. 1B). However, in OxyB, where Asn also replaces the conserved I-helix Thr, the I-helix H-bond is longer than structures containing the canonical sequence. Thus, the N242T mutation could still form a I-helix H-bond, but the shorter length may still disrupt the active site negatively impacting substrate binding and catalysis.^23,35^

In contrast, the N242L mutation should lose the I-helix H-bond completely so it is surprising that it maintains apparently good substrate binding and the best activity with the natural substrate 1. To interrogate the structural impact of the N242L mutation, it was modelled with rufomycin B 1. The substrate sits further away from heme iron (5.43 Å) and forms weaker contacts to the enzyme (by ∼3.2 Rosetta energy units) than the WT, providing some explanation for reduced catalytic activity (Fig. 6F and G). Interestingly, there are significant changes in the binding pose of rufomycin B 1 in the active site of N242L relative to the WT (Fig. 6G and S39) indicating wider structural change in the binding pocket that could lead to weaker binding of substrate and oxidised intermediates. The N242L sidechain, unable to hydrogen bond to the I-helix backbone, rotates into the substrate binding site, displacing the substrate tryptophan away from the I helix. This movement appears to cause rotation of the cyclic peptide, shifting the terminal carbon of leucine, the site of oxidation, away from heme iron. The substrate movement causes a rotameric change of 3-nitrotyrosine but is still accommodated by the flexible remodelled loop. Thus, it is possible that absence of the I-helix H-bond allows substrate binding, but not always in an orientation to enable productive catalysis thus explaining the misalignment of binding and catalysis data compared to N242Q.

### One-pot conversion of rufomycin analogue with RufS/RufM–N242L

With mutants in hand that restrict production of carboxylic acid derivatives, we decided to determine if the best performing mutant with natural substrate 1, N242L, could process rufomycins in concert with the epoxidizing enzyme RufS (Fig. 1 and 6). Previously, we reported a one-pot reaction containing RufM and RufS could convert rufomycin B to rufomycin C with few intermediates.^11^ As RufS activity has not been demonstrated with non-native derivatives, we chose peptide 2 that behaves similarly to the native substrate 1 (Fig. 6D, E and S28, S29).

His_6_-RufS was produced as previously reported (Fig. S8) and assays of RufS with peptide 2 resulted in epoxidized product with the same conversion as the natural substrate.^11^ We previously observed that the success of RufM/RufS reactions were strongly dependent on electron transport protein (ferredoxin (Fd)/ferredoxin reductase (Fr)) concentration and we observed similar results in this work with peptide 5 (Fig. S19). Thus, Fd and Fr concentration were optimised using wild type RufM and then the RufM/RufS cascade resulting in a majority of the rufomycin C derivative epox-2c (Fig. 6D and S28, S29). When wild type RufM was replaced with the N242L mutant, epoxidized alcohol epox-2a and aldehyde/hemiaminal epox-2b/b* products were detected but no carboxylic acid epox-2c (Fig. 6E). While conversions are low, we have demonstrated the ability to prevent further oxidation of rufomycins beyond the aldehyde by single point mutation both in single enzyme and cascade reactions.

## Conclusions

RufM has proven to be a fascinating enzyme with a complex product profile encompassing catalysed and spontaneous reactions. RufM shows surprising promiscuity, even accepting derivatives with modifications at the site of catalysis. Our substrate screen revealed several examples of substrate driven changes in regioselectivity including more polar peptides, containing Ser 13 or Thr 14 in place of Ala, that resulted in novel oxidised derivatives. In addition, and perhaps most surprisingly, non-proteinogenic bespoke amino acids that result from pathway specific biosynthetic processes, are not crucial for tailoring enzyme substrate recognition or activity. For example, peptides with tyrosine in place of 3-nitrotyrosine, known to be important for antimycobacterial activity, were readily accepted by RufM, with minimal changes in activity.

Finally, mutagenesis of a key residue, N242, in the catalytically important I-helix suggests that N242 contributes substantially to maintaining the integrity of the RufM active site to ensure productive substrate binding, particularly of intermediate oxidation products. Intriguingly, our data shows RufM to be tunable *via* minimal mutation towards new products and substrates.^8^ In addition, mutant RufM–N242L facilitated a one-pot transformation with RufS, allowing conversion of a rufomycin analogue to the corresponding epoxidized alcohol and aldehyde intermediates, thus accumulating the most biologically active intermediate.

Our compelling data on RufM substrate promiscuity and insight into the activity of N242 mutants could be rationalised by modelling these enzymes in complex with native substrate rufomycin B 1. However, the results also illustrate that there is still much more to understand about how changes in this conserved region of CYPs impact active site structure and dynamics that requires more detailed structural and computational data to fully explain.

CYP biosynthetic tailoring enzymes catalyse synthetically challenging, stereo and regiospecific C–H bond activating reactions, but are often under studied due to difficulty accessing their complex substrates. Here we demonstrate the power of rapid bioinspired peptide cyclisation chemistry as a tool to provide facile access to cyclic peptide substrate analogues, enabling detailed investigations of an unusual CYP tailoring enzyme.

Our chemoenzymatic approach has produced >50 rufomycin derivatives and will inform engineering of the rufomycin pathway and NRPS pathways more generally. This work illustrates the potential of biosynthetic non-ribosomal cyclic peptide (NRcP) tailoring enzymes as late-stage functionalizing biocatalysts. We hope that our success promotes the use of late-stage enzymatic functionalisation to create derivative peptide libraries to tackle antimicrobial resistance.

## Experimental

All the experimental details including biochemistry, molecular biology, small molecule and peptides synthesis are provided in the SI. The authors have cited additional references within the SI.^56–66^

## Author contributions

J. P., Y. D., J. T., H. R. W., T. H., J. Z., C. M. L. C., P. S. and G. P.-O. carried out the experiments and J. P., Y. D., J. T., H. R. W., T. H., G. P.-O., S. M. B., C. M. L. C., P. S., T. D. M. and J. H. N. analysed the data. R. E. J. carried out the computational and docking studies. J. P. and S. M. B. wrote the paper with editorial contributions from all authors.

## Conflicts of interest

Y. D., S. M. B. and J. T. are named inventors on a UK priority patent application filed by King's College London describing bioinspired peptide cyclisation (GB2402551.2).

## Supplementary Material

SC-017-D6SC01721E-s001

## Data Availability

Supplementary information (SI): all small molecule and peptide characterization, and other experimental data. See DOI: https://doi.org/10.1039/d6sc01721e.
