# Supplementary material for: Bioinspired peptide cyclisation enables exploration of cytochrome P450 catalysed sequential oxidation in rufomycin biosynthesis
Source: Chem Sci. 2026 Aug 5;17(37):17841–53. doi: 10.1039/d6sc01721e (PMC13439498; doi:10.1039/d6sc01721e)
Supplement: Supplementary file 1 [file SC-017-D6SC01721E-s001.pdf]

Supplementary Information (SI) for Chemical Science.
This journal is © The Royal Society of Chemistry 2026

Supplementary information for:

Bioinspired Peptide Cyclisation Enables Exploration of Cytochrome P450 Catalysed Sequential Oxidation in Rufomycin Biosynthesis

Jessica Peate[a], Yaoyu Ding[a], Joshua Tomkins[a], Harry R. Winter[a], Jingwen Zhang[a], Cameron M. L. Coleman[b], Priya Solanki[b], Jake H. Nicholson[a], Toby Hopkinson[a], Gustavo Perez Ortiz[a], Alex P. S. Brogan[a], Timothy D. McHugh[b], Robert E. Jefferson[a], Sarah M. Barry*[a]

[a]*Department of Chemistry, Faculty of Natural, Mathematical and Engineering Sciences, King's College London, Britannia House, 7 Trinity Street, London, SE1 1DB, UK*
[b]*University College London, Centre for Clinical Microbiology, Royal Free Campus, Rowland Hill St., London NW3 2QG*

* To whom correspondence should be addressed. Email: sarah.barry@kcl.ac.uk

Supplementary and methods

Contents

General methods and procedures 8

1. Peptide Synthesis 8

1.1. General procedure of hydrazine functionalizing 2-chlorotrityl resin and manual peptide coupling method 8

1.2. General procedure for solid phase synthesis of linear rufomycin peptides and derivatives. 9

1.3. General procedure for peptide cleavage from resin 9

1.4. Chemical peptide cyclisation 10

Chromatography Methods for Peptide Purification and Analysis 22

1.5. LC-MS chromatography 23

1.6. Analytical HPLC 23

1.7. Semi-preparative HPLC 23

1.8. UPLC-HRMS 23

2. **Production and Characterisation of RufM and Mutants** 24

2.1. *rufM* site directed mutagenesis 27

2.2. Production of RufM, RufS and Mutants 29

2.3. Cleavage of His_6-SUMO-RufM 31

2.4. UV-Visible Analysis of RufM and mutants 32

2.5. Heme determination of RufM and Mutants 34

2.6. RufM-Peptide Binding Studies 34

2.6.1 Method 1: K_d calculation 34

2.6.2 Method 2: K_d calculation 39

2.7. Catalytic Activity of RufM and Mutants 41

2.8. Determining the identity of the intermediate (12d) 58

2.9. Assessing RufM product inhibition 60

2.10. RufM assays: Conversion tables and HRMS of substrates and products. 61

3. Computational Modelling 71

3.1. Docking Heme into RufM model 71

3.2. Docking rufomycin B, 1a, into heme bound RufM model 71

Testing of Selected Rufomycin Derivatives against *Mtb* 75

3.3. Microbiological activity of peptides 75

Supplementary References 76

Supplementary Characterization Data..78

Figure S1: Functionalisation of 2-chlorotrityl resin to give 2-chlorotrityl hydrazine resin and loading of first amino acid. Exemplified by the synthesis of peptide **3**. See hazard note above. 9
Figure S2: General procedure for solid phase peptide synthesis. Exemplified by synthesis of peptide **3**. 9
Figure S3: General procedure for peptide cleavage. Exemplified by synthesis of peptide **3**. 9
Figure S4 Biosynthesis of rufomycin B on the nonribosomal peptide synthetase, RufT. Inset: The C terminal TE domain cyclises the peptide. 24
Figure S5: Biosynthesis of rufomycin B on the nonribosomal peptide synthetase, RufT. Inset: The C terminal TE domain cyclises the peptide. 25
Figure S6: Clustal Omega alignment of RufM with key P450s. P450Eryf (PDB: 6DEB), P450SaAcm (PDB: 5NWS), P450CymS (SARE4557; CP000850.1),[2] P450cam (PDB: 5CP4), P450cin (PDB: 4FB2). Highlighted in yellow is the catalytically important I helix consensus sequence ((A/G) Gx(E/D)T) that differs among the P450s. 26
Figure S7: Plasmid map of His_6-SUMO-RufM . Map shows the general features of the plasmid and the location of the gene rufM. Features include the Small Ubiquitin-like Modifier (SUMO) solubility partner tag for ease of purification, an ampicillin resistance gene (AmpR) for selection, a 6x polyhistidine tag for protein purification with HisTrap columns, a TEV cleavage site and T7 promoter and lac repressor protein (lacI) for IPTG-induced gene expression. 27
Figure S8: SDS-gel of purified CYP proteins A. His_6-SUMO-RufM (56.1 kDa), L. Protein Ladder, 1. WT N242, 2. N242T, 3. N242A, 4. N242S 5. N242D, 6. N242E, 7. N242Q, 8. N242L, **B.** RufM purification steps, 9. Purified RufM by HisTrap, 10. Purified RufM by HisTrap then SEC, **C.** His_6-RufS (47.9 kDa). Note: activity of RufM lane 1 A and lane 9 B is comparable. 30
Figure S9: Analysis of ULP-1 production A) SDS-gel of ULP-1 protein expression and purification: 1. Protein ladder, 2. Pellet after final wash, 3. Lysate after final wash, 4. HisTrap binding buffer wash, 5. HisTrap binding buffer with imidazole wash, 6. peak 6 in urea buffer wash, 7. peak 7 in urea buffer wash, 8. peak 8 in elution buffer, 9. peak 8 after concentration, 10. final protein 31
Figure S10: Analysis of His_6-SUMO-RufM cleavage. A) SDS-PAGE analysis of His_6-SUMO-rufM cleavage: 1. Protein Ladder, 2. ULP-1 SUMO-cleavage reaction after 18 hours at 4°C, 3. Buffer A wash, 4. Buffer B flowthrough, 5. RufM after nickel affinity chromatography post cleavage, 6. His_6-SUMO-RufM after nickel affinity chromatography prior to cleavage, 7. ULP-1. B) UV-Visible spectra of His_6-SUMO-RufM shown in the red hashed line and RufM is shown in the blue solid line. 32
Figure S11: LC-MS analysis showing conversion of Peptide 1 by His_6-SUMO-RufM and RufM. Traced recorded at 355 nm absorbance. Dotted line shows the peptide 1 standard, lower line is His_6-SUMO-RufM, and the higher line is RufM. The assay was carried out for 3 hours at 30 °C thrice according to section 2.7. 32
Figure S12: UV-Visible spectra of the P450 RufM and its mutants. Spectra recorded from 200 – 600 nm for N242, N242Q, N242T, N242S, N242A, N242L, N242E and N242D...........33
Figure S13: UV-Visble spectra studies of N242 RufM with increasing concentrations of 10 (0- 12 µM). A) UV-visible spectra 250-600 nm. Low spin iron (II) peak identified at 416 nm for the non-substrate bound protein and high spin iron (II) identified at 392 nm with 12 µM substrate bound. B) Zoom in (×3) of the UV-visible spectra 470-700 nm showing the absorbance maxima for the substrate free protein at 535 nm and 568 nm. The substrate bound

protein shows a maxima at 648 nm. C) Absorbance difference between 391 and 416 nm spectra with increasing concentrations with an isosbestic point at 404 nm. D) Absorbance difference plotted versus **10** concentration used to determine a K_d value of 5.32 μM ± 1.29 μM. 35
Figure S14: UV-Vis spectra studies of N242 RufM with increasing concentrations of 9 (0 - 12 μM). A) UV-visible spectra 250-600 nm. Low spin iron (II) peak identified at 416 nm for the non-substrate bound protein and high spin iron (II) identified at 392 nm with 12 μM substrate bound. B) Zoom in (×3) of the UV-visible spectra 470-700 nm showing the absorbance maxima for the substrate free protein at 535 nm and 568 nm. The substrate bound protein shows a maxima at 648 nm. C) Absorbance difference between 391 and 416 nm spectra with increasing concentrations with an isosbestic point at 404 nm. D) Absorbance difference plotted versus **9** concentration used to determine a K_d value of 1.06 μM ± 0.338 μM. 36
Figure S15: UV-Vis spectra studies of N242 RufM with increasing concentrations of 11 (0 - 12 μM). A) UV-visible spectra 250-600 nm. Low spin iron (II) peak identified at 416 nm for the non-substrate bound protein and high spin iron (II) identified at 392 nm with 12 μM substrate bound. B) Zoom in (×3) of the UV-visible spectra 470-700 nm showing the absorbance maxima for the substrate free protein at 535 nm and 568 nm. The substrate bound protein shows a maxima at 648 nm. C) Absorbance difference between 391 and 416 nm spectra with increasing concentrations with an isosbestic point at 404 nm. D) Absorbance difference plotted versus **11** concentration used to determine a K_d value of 0.49 μM ± 0.119 μM. 37
Figure S16: UV-Vis spectra studies of N242T RufM with increasing concentrations of 11 (0 - 12 μM). A) UV-visible spectra 250-600 nm. Low spin iron (II) peak identified at 419 nm for the non-substrate bound protein and high spin iron (II) identified at 392 nm with 12 μM substrate bound. B) Zoom in (×3) of the UV-visible spectra 470-700 nm showing the absorbance maxima for the substrate free protein at 537 nm and 568 nm. The substrate bound protein shows a maxima at 643 nm. C) Absorbance difference between 391 and 416 nm spectra with increasing concentrations with an isosbestic point at 404 nm. D) Absorbance difference plotted versus **11** concentration used to determine a K_d value of 9.53 μM ± 0.556 μM. 38
Figure S17: UV-Vis spectra studies of N242L RufM with increasing concentrations of 11 (0 - 12 μM). A) UV-visible spectra 250-600 nm. Low spin iron (II) peak identified at 416 nm for the non-substrate bound protein and high spin iron (II) identified at 392 nm with 12 μM substrate bound. B) Zoom in (×3) of the UV-visible spectra 470-700 nm showing the absorbance maxima for the substrate free protein at 540 nm and 570 nm. The substrate bound protein shows a maxima at 646 nm. C) Absorbance difference between 392 and 422 nm spectra with increasing concentrations with an isosbestic point at 404 nm. D) Absorbance difference plotted versus **11** concentration used to determine a K_d value of 1.26 μM ± 0.189 μM. 39
Figure S18: Alternative fittings for binding curves of RufM and mutants with peptides **9**, **10** and **11** (0-12 μM) calculated using equations S2 and S3. Absorbance difference plotted versus peptide concentration used to determine a K_d values of **A**) peptide **10** with RufM WT - 3.34 μM ± 0.72 μM. **B**.) peptide **9** with RufM WT - 1.18 μM ± 0.26 μM. **C**) peptide **11** with RufM WT - 0.67 μM ± 0.13 μM. **D**) peptide **11** with RufM N242L – 1.59 μM ± 0.34 μM. **E**) peptide **12** with RufM N242T – 3.15 μM ± 0.71 μM. **Inset**: table summarising K_d data. 41
Figure S19: Optimising concentrations of Ferredoxin and Ferredoxin reductase in RufM assays. Left) LC-MS analysis (355 nm) showing products formed by RufM (N242) with peptide **5** at different concentrations of ferredoxin and ferredoxin reductase. Products are assigned by colour where blue = starting substrate, green = aldehyde product, pink =

dehydrated product, purple = carboxylic acid. Right) table indicating concentrations used. All other conditions as described in Table S4. 42
Figure S20: Optimising concentration of RufM (N242). Left) LC-MS trace at 355 nm showing product formation by different concentrations of RufM (N242) with peptide **5** (50μM) Right) Negative control (bottom) and RufM N242 (20 μM) reacted with 80 μM peptide **5** (right). Products are assigned by colour where blue = starting substrate, green = aldehyde product, pink = dehydrated product, purple = carboxylic acid product. All other conditions were as given in table S4. 43
Figure S21: **RufM Wild Type (N242) LC-MS assay with peptides 1-8, 12-16, measured at absorbance 355 nm.** Products are assigned by colour where blue = starting substrate (**1-8, 12-16**), orange = alcohol product (**1a-8a, 12a-16a**), green = aldehyde product (**1b/b*-8b/b*, 12-16b/b***), pink = dehydrated product (**1d-8d, 12d-16d**), purple = carboxylic acid product (**1c-8c, 12-c16c**). The symbol * indicates where products are not easily separated via this gradient. 44
Figure S22: RufM Wild Type (N242) LC-MS assay with Peptides 13 and 14 A) LCMS chromatogram of RufM reaction with peptide **13** with an optimised LC method for better separation (1 min with 40% ACN mixed with 60% water, linear gradient from 40%- 90% ACN over 38 mins, then wash 2 min with 95% ACN, then back to 5% ACN over 2 min). at 355 nm **B)** LCMS chromatogram of RufM reaction with peptide **14** with 40-90% gradient for separation at 355 nm . Products are assigned by colour where blue = starting substrate (**13** or **14**), orange = alcohol/diol product, green = aldehyde product, pink = dehydrated produc, purple = carboxylic acid product. The symbol * represents the product with multiple alcohol groups. C) Reaction scheme with peptide **13** and products including possible structures of dihydroxylated product * D) Fragmentation observed rules out possible structures **C**, **D**, **F**. **E)** Fragment observed rules out structures **B** and **D**. Thus, **A** is the most likely product. 45
Figure S23: MS^e spectrum of dihydroxylated product from reaction of RufM with peptide 11. y-ions are labelled. 46
Figure S24: RufM Wild Type (N242) LC-MS assay with peptides 10(top), 9(middle) and 11(bottom) at absorbance 280 nm. Products are assigned by colour where blue = starting substrate (**9-11**), green = aldehyde product (**9b-11b**), pink = dehydrated product (**9d-11d**), purple = carboxylic acid product (**9c-11c**). 48
Figure S25: Reaction and binding of RufM with peptides **17** and **18**. Effect of peptides **17** and **18** on the visible absorption spectra of wild type RufM and their activity assay. **A)** UV-visible binding assay of N242 RufM (5 μM, 25mM Tris pH8) with increasing concentrations of **17** (0 - 80 μM). **B)** UV-visible spectra studies of N242 RufM (5 μM, 25mM Tris pH8) with increasing concentrations of **18** (0 - 80 μM). Low spin iron (II) peak identified at 416 nm for the non-substrate bound protein. **C**) LC-MS trace (355 nm) to analyse reactions of RufM N242 with peptides **17** and **18**. Controls contain heat inactivated RufM. Products are labelled by colour where blue = substrate. **D**) LC-MS trace at 355 nm showing product formation by RufM (N242, N242A, N242Q, N242L) with peptide **17**. **E)** LC-MS trace at 355 nm showing product formation by RufM (N242, N242A, N242Q, N242L) with peptide **18**. 49
Figure S26: LC-MS assay trace of peptide 1. Traces show N242 and mutants N242Q, N242T, N242S, N242A, N242L, N242E and N242D recorded at absorbance 355 nm. Products are assigned by colour where blue = starting substrate **1**, orange = alcohol product **1a**, green = aldehyde product **1b/b***, pink = dehydrated product **1d**, purple = carboxylic acid product **1c**. 50
Figure S27: LC-MS assay trace of peptide 2. Traces show N242 and mutants N242T, N242S, N242A and N242L recorded at absorbance 355 nm. Products are assigned by colour where blue = starting substrate **2**, orange = alcohol product **2a**, green = aldehyde product **2b/b***, pink = dehydrated product **2d**, purple = carboxylic acid product **2c**. 51

Figure S28: LC-MS assay trace of peptide 2 with varying amounts of Fd/ Fr. Traces show assays containing RufM N242/ RufS and mutant N242L/ RufS recorded at absorbance 355 nm. Products are assigned by colour where light blue = substrate **2**, dark blue = substrate **2** with epoxide **epoxy-2**, orange = alcohol product **2a**, light green = aldehyde product **2b/b*,** dark green = aldehyde product with epoxide **epoxy2b/b*,** light pink = dehydrated product **2d**, dark pink = dehydrated product with epoxide **epoxy-2d**, light purple = carboxylic acid product **2c**, dark purple = carboxylic acid product with epoxide **epoxy2c**. 51
Figure S29: Transformation of 2 with RufM/ N242L and RufS. 52
Figure S30: **LC-MS assay trace of peptide 3.** Traces show N242 and mutants N242Q, N242T, N242S, N242A and N242L recorded at absorbance 355 nm. Products are assigned by colour where blue = starting substrate **3**, orange = alcohol product **3a**, green = aldehyde product **3b**/**b***, pink = dehydrated product **3d**, purple = carboxylic acid product **3c** 53
Figure S31: LC-MS assay trace of peptide 3 & 4. Top) Traces show N242 and mutants N242T, N242A, N242D and N242L recorded at absorbance 355 nm. Bottom) Chart showing proportion of products formed from reaction of RufM WT or N242A with peptides **3** & **4**. Conversions are averages of 3 biological repeats and were determined using LC-MS peak area at 355 nm. Products are assigned by colour where blue = starting substrate **2**, orange = alcohol product **4a**, green = aldehyde product **4b**/**b***, pink = dehydrated product **4d**, purple = carboxylic acid product **4c.** 54
Figure S32: LC-MS assay trace of peptide 5. Traces show N242 and mutants N242Q, N242T, N242A, N242L, N242E and N242D recorded at absorbance 355 nm. Products are assigned by colour where blue = starting substrate **5**, orange = alcohol product **5a**, green = aldehyde product **5b**/**b***, pink = dehydrated product **5d**, purple = carboxylic acid product **5c** 55
Figure S33: LC-MS assay trace of peptide 8. Traces show N242 and mutants N242T and N242A recorded at absorbance 355 nm. Products are assigned by colour where blue = starting substrate **8**.. 56
Figure S34: LC-MS assay trace of peptide 12. Traces show N242 and mutants N242Q, N242T, N242S, N242A, N242L, N242E and N242D recorded at absorbance 355 nm. Products are assigned by colour where blue = starting substrate **12**, orange = alcohol product **12a**, green = aldehyde product **12b**/**b***, pink = dehydrated product **12d**, purple = carboxylic acid product **12c**. 57
Figure S35 LC-MS assay trace of peptide 11. Traces show N242 and mutants N242Q, N242T, N242S and N242L recorded at absorbance 280 nm. Products are assigned by colour where blue = starting substrate **11**, orange = alcohol product **11a**, green = aldehyde product **11b**/**b***, pink = dehydrated product **11d**, purple = carboxylic acid product **11c**........................ 58
Figure S36: Determining the identity of the intermediates b and d. Peptide **12** (50 µM) assay with N242 RufM (20 µM) and the assay components (See Table S6) at 30°C for 30 minutes (A), following the reaction the peptide was reduced using sodium borohydride (1 mM) for 30 minutes (B1) and for 60 minutes (B2). 59
Figure S37: Assessing product inhibition of RufM. LC-MS trace at 355 nm showing products formation by RufM with peptide **5**. Light blue line is the products formation of round 1. Dark blue line is the product formation of round 2. Conversions (average of 3 repeats) were determined using LC-MS peak area at 355 nm. Products are assigned by colour where blue = substrate, green = aldehyde, pink = enamine, purple = carboxylic acid. 60
Figure S38: Purity of synthesised cyclic peptides. HPLC traces of peptides **1-18** at absorbance 214 nm using gradient 1. Peaks at 30 &~39 min are HPLC artefacts due to changes in solvent…………………………………………………………………………..70

Figure S39: Modelling of RufM N242 and RufM N242L. A) Rufomycin B bound to RufM N242; B) Rufomycin B bound to RufM N242L C) Rufomycin B bound to RufM N242 highlighting heme, I helix and N242 residue; D) Rufomycin B bound to RufM N242L……………………73

Figure S40: Clustal Omega alignment of RufM with key P450s. P450SaAcm (PDB: 5NWS), P450CymS indicating conserved structural motifs and tertiary structure. Cylinders indicate alpha helices and arrows beta sheets. Line indicates unstructured region……………………74

Table S1: Primers used for mutagenesis of RufM in PCR reactions. The triple codon is in bold and the nucleotide undergoing the point mutation is underlined. 28
Table S2: PCR cycle parameters for the mutagenesis of RufM...29
Table S3: P450 RufM Heme incorporation estimation and Soret shifts. Heme incorporation reported as ratio between the absorbance max for the Soret band and the protein peak at 280 nm (A_{420}/A_{280}). Soret band wavelength at its absorbance max (λ_{max}) determined using UV-Visible spectroscopy. 34
Table S4: Reaction mix used to measure the catalytic activity of RufM and its mutants. 42
Table S5: Full Table of fragments generated by MMass[8] analysis from LC-HRMS of Peptide 11 dihydroxylated product. Fragments used to determine structure of the product in Figure S17 are highlighted in green. 47
Table S6: Large scale reaction mix for peptide 12 and N242 RufM. 59
Table S7: Wild Type (N242) LC-MS assay % conversions taken from HPLC traces at 355nm. Values are average of 3 experiments +/- standard deviation. 61
Table S8: Mutant LC-MS assay % conversions with peptide 1 from HPLC traces at 355nm. Values are average of 3 experiments +/- standard deviation. 61
Table S9: Mutant LC-MS assay % conversions with peptide 5 from HPLC traces at 355nm. Values are average of 3 experiments +/- standard deviation. 61
Table S10: Mutant LC-MS assay % conversions with peptide 12 from HPLC traces at 355nm. Values are average of 3 experiments +/- standard deviation. 62
Table S11: HRMS data of synthesised hydrazide peptides and cyclic peptides. All peptide mass listed are reported as $[M+H]^+$ and only monoisotopic peaks are listed in the table. All peptide samples are analysed on UPLC-HRMS using Gradient 6. The synthesis of peptides **1,2,4,5,8,12,13**, **14 17 and 18** have been reported previously.[1] 64
Table S12 HRMS data of products from assay with RufM. All peptide mass listed are reported as $[M+H]^+$ and only monoisotopic peaks are listed in the table. All peptide samples are analysed on UPLC-HRMS using Gradient 6. Dash indicates that species wasn't detected 68
Table S13: Reults of MIC testing for peptides 4, 5 and 8...75

General methods and procedures

Reagents and solvents were purchased from suppliers and used without additional purification. Reactions run under N_2 atmosphere were conducted in glassware that was oven dried. DMF (sequencing grade) for Solid Phase Peptide Synthesis was purchased from Cambridge Reagent Ltd. Nuclear Magnetic Resonance (NMR) spectra were collected using a Brucker UltraShieldTM 600 MHz or 700 MHz at 18°C. Chemical shifts (δ) are reported in parts per million (ppm) and peak assignments are made using the following abbreviations: s, singlet; d, doublet; dd, doublet of doublets; t, triplet; q, quartet. Resonances that cannot be easily interpreted as individual signals were assigned as multiplets (m). The chemical shifts are assigned in reference to the residual solvent signal. High resolution mass spectra (HRMS) were recorded using a Waters Acquity UPLC-Class I equipped with an ACQUITY UPLC column and the detector was a Waters Xevo-G2-XS QTof with electrospray ionization source. The instrument was in positive mode full scan with a 50 to 2000 Da detection window. For peptide fragmentation, a collision energy ramp from 15 V to 40 V was employed.

For bacterial studies, the procedures were carried out under sterile conditions in a laminar flow hood (Envair). When stated, protein purification was performed on an AKTA Fast Protein Liquid Chromatography (FPLC) Instrument (GE Healthcare). Stock solutions for expression include: carbenicillin (50 mg mL^{-1}), isopropyl-β-D-thiogalactopyranoside IPTG (0.5 M), dithiothreitol DTT (1 M), and 5-aminolevulinic acid hydrochloride (0.25 M) and were made in distilled water then filter sterilized using Minisart NML Syringe Filters (cellulose acetate membrane, 0.2 µM, 26 mm), then stored in aliquots at -20 °C. Iron (II) sulfate heptahydrate (0.1 M) was prepared fresh in distilled water and filter sterilized. Fresh solutions of 20 mM nicotinamide adenine dinucleotide phosphate were prepared in dH_2O. Ferredoxin (5 mg) and ferredoxin NADP reductase (5 units) from *Spinacia oleracea* from Sigma Aldrich were resuspended in 0.5 mL buffer containing Tris pH 8, 100 mM NaCl and 30% glycerol and stored in aliquots at -20 °C. The *EZ-Run* Prestained Rec Protein Ladder was purchased from Fisher BioReagents. PCRs were performed using an Eppendorf Mastercycler Nexus gradient eco thermocycler. Sequencing of plasmids was obtained from GENEWIZ. Primers were purchased from Integrated DNA technologies.

1. Peptide Synthesis

Peptides **3, 6, 7, 9, 10, 11, 15, 16, 17, 18** were synthesised for this studied. Batches of peptides **1, 2, 4, 5, 8, 12, 13, 14** were made as previously reported here.[1]

(NOTE - Hazard: Hydrazine hydrate is toxic (hazard codes H302, H330, H311, H314, H317, H318, H350, H400, H410) and recommended safety precautions should be taken. 2-Chlorotrityl hydrazine resin is commercially available).

1.1. General procedure of hydrazine functionalizing 2-chlorotrityl resin and manual peptide coupling method

2-chlorotrityl chloride resin (substitution: 1.06 mmol/g) (150 mg, 0.15 mmol, 1 equiv.) was stirred in dimethylformamide (DMF) (1.5 mL) at 0 °C for 15 mins. A mixture of triethylamine (63 µL, 0.45 mmol, 3 equiv.) and hydrazine hydrate (14.5 µL, 0.3 mmol, 2 equiv.) in DMF (0.5 mL) was added dropwise at 0 °C, and then the resulting mixture was stirred for 1 hour at room temperature. Methanol (1 mL) was added and stirred for 15 min. The resin was filtered by vacuum, washed with DMF (3 x 8 mL), water (2 x 5 mL), DMF (2 x 5 mL), methanol (2 x 5 mL) and DMF (3 x 5 mL). Then, the C-terminal Fmoc amino acid was loaded manually, a mixture

of Fmoc amino acid (0.3 mmol, 2 equiv.), K-Oxyma (54.6 mg, 0.3 mmol, 2 equiv.) and *N, N'*-Diisopropylcarbodiimide (DIC) (47.5 µL, 0.3 mmol, 2 equiv.) in DMF (1.5 mL) was added to the resin. The mixture was swelled with nitrogen flow for 45 min. The resin was filtered and double coupled using the same procedure. After that, the resin was filtered and washed with DMF (5 x 2 mL).

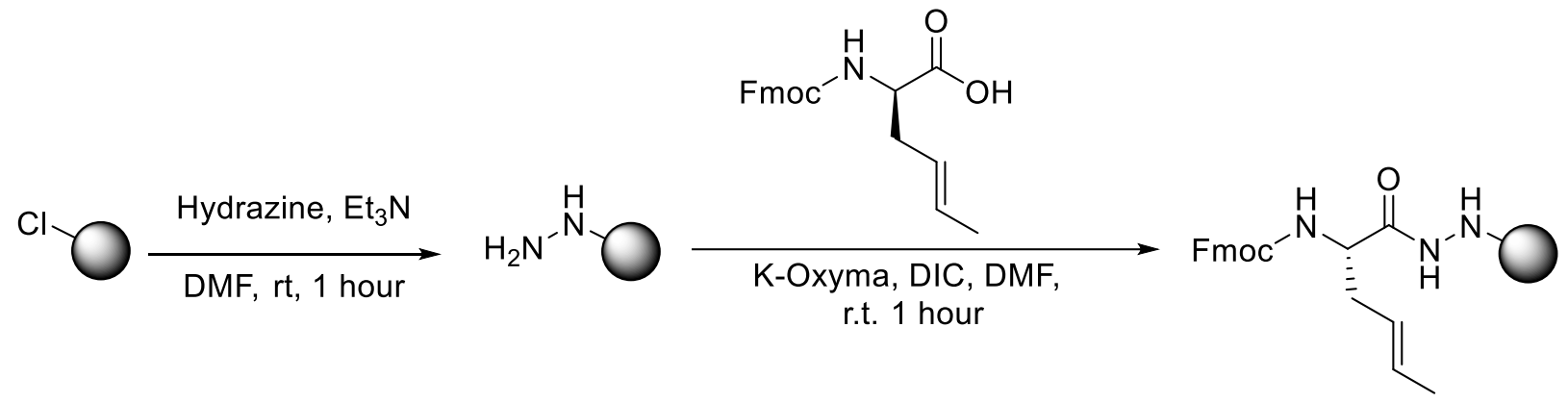

Figure S1: Functionalisation of 2-chlorotrityl resin to give 2-chlorotrityl hydrazine resin and loading of first amino acid. Exemplified by the synthesis of peptide **3**. See hazard note above.

1.2. General procedure for solid phase synthesis of linear rufomycin peptides and derivatives.

Solid phase peptide synthesis (SPPS) was performed on a Biotage initiator+ Alstra peptide synthesiser at room temperature using a standard Fluorenylmethoxycarbonyl (Fmoc) Strategy. Fmoc- amino acids (4 equiv.) was coupled at room temperature for 1 hour with DIC (4 equiv.) and Oxyma (4 equiv.). The Fmoc deprotection was carried out by swelling the resin using 20% piperidine in DMF twice at room temperature. The first deprotection was carried out for 1 minute followed by an additional deprotection for 15 minutes.

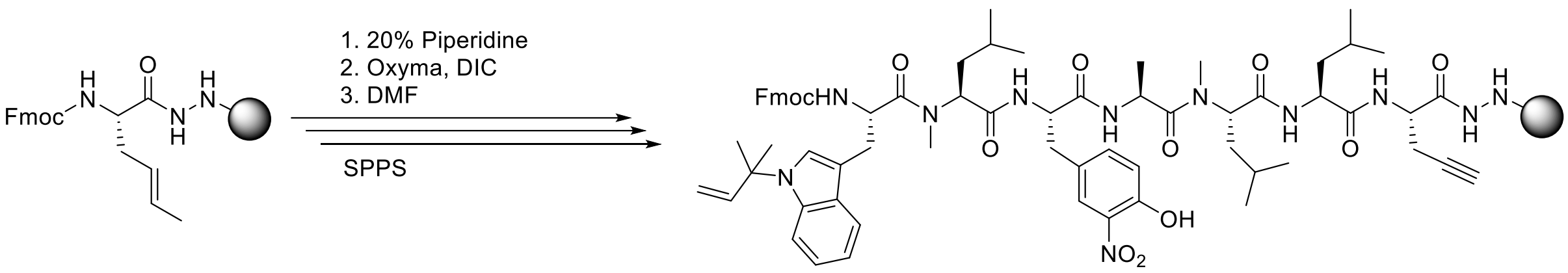

Figure S2: General procedure for solid phase peptide synthesis. Exemplified by synthesis of peptide **3**.

1.3. General procedure for peptide cleavage from resin

Peptidyl resins (150 mg) were treated with 3 mL of cleavage cocktail. Cocktail A (1% TFA. 2% TIS and 97% DCM) was used for peptides containing N-prenylated tryptophan and Cocktail B (95% TFA, 2.5% TIS and 2.5% water) was used for all other peptides. The resulting solution was rocked at room temperature for 2 hours. The resin was filtered and washed with DCM (2 x 5 mL) and the filtrates were evaporated under nitrogen. Cold diethyl ether (3 x 10 mL) was added to precipitate hydrazide peptide. Peptides were dissolved in 50% acetonitrile and analysed by LC-MS or HRMS.

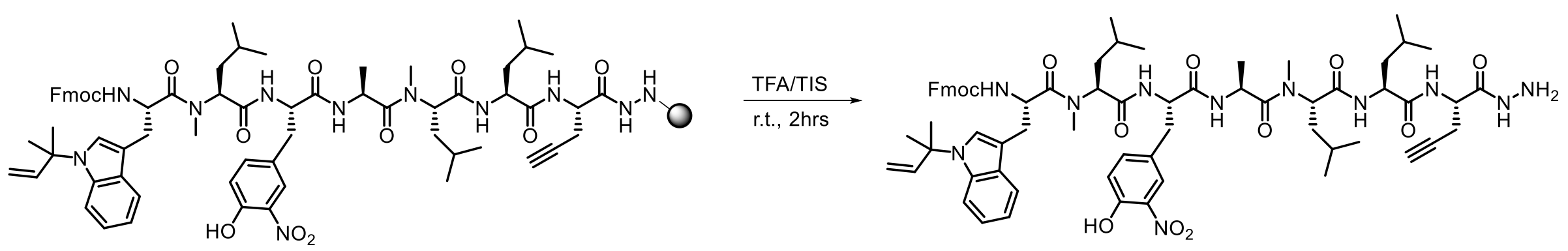

Figure S3: General procedure for peptide cleavage. Exemplified by synthesis of peptide **3**.

1.4. Chemical peptide cyclisation

All buffers and solutions used in the peptide cyclisation were prepared freshly and then degassed prior to use by stirring and bubbling under nitrogen overnight. All peptides were initially dissolved in degassed cyclisation buffer (6 M GdmCl, 50 mM NaH_2PO_4, 1.5 mM EDTA pH 5). A 5 M sodium nitrite solution was prepared using degassed distilled water under nitrogen. Rufomycins are known to exist in multiple conformers in solution[1] in each case only the major conformer is assigned.

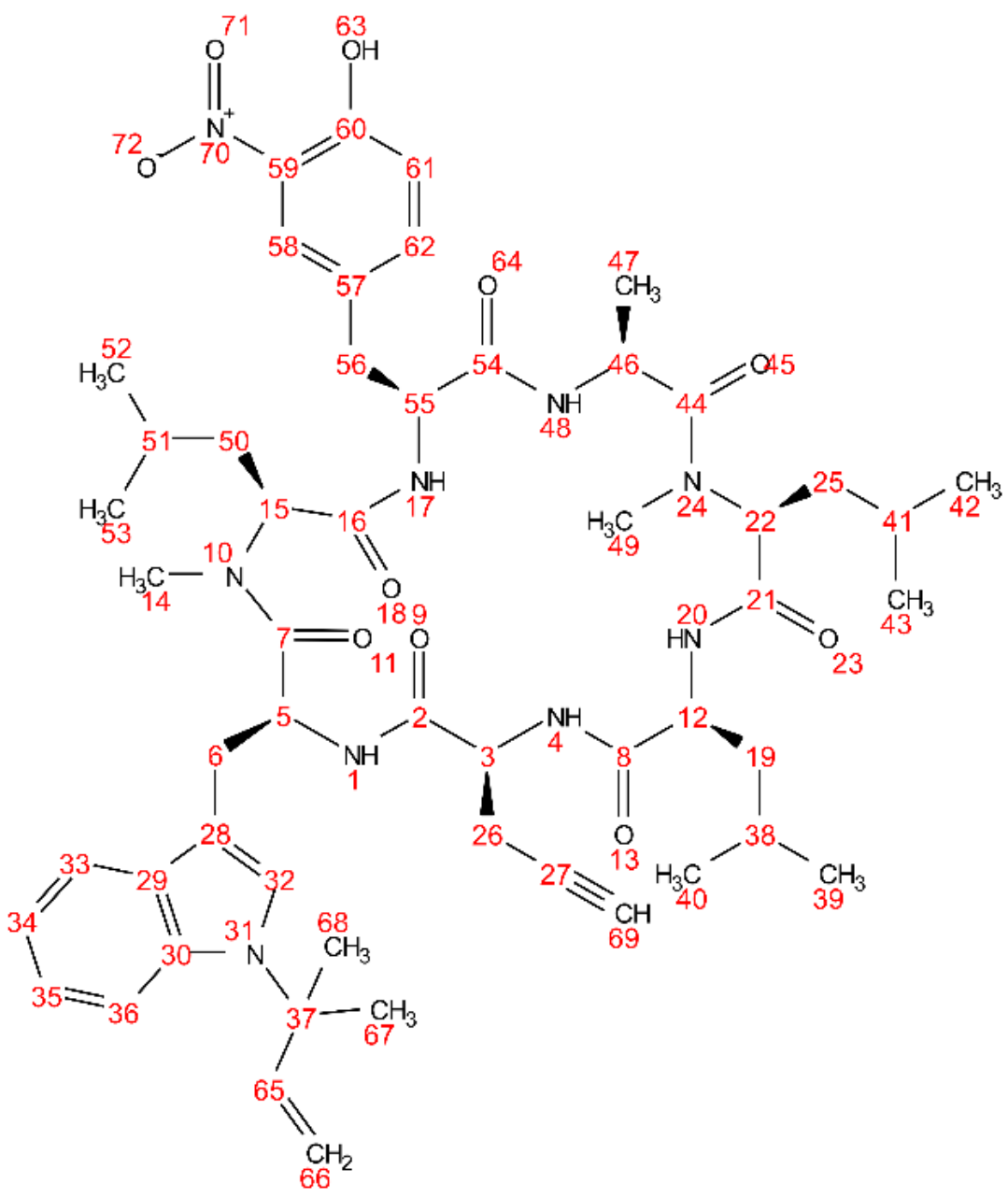

3

Peptide 3: Purified and dried linear hydrazide peptide **3-linear** (15.07 µmol, 15 mg, 1 equiv.) was dissolved in 10 mL cyclisation buffer and sodium nitrite stock solution (300 uL) was added at -15 °C under nitrogen. The solution was stirred for 20 minutes at -15 °C. The mixture was gently neutralised using saturated sodium bicarbonate (~1000 µL) to pH 7.5. 15 mL of ethyl acetate was added, and the mixture stirred vigorously for 45 minutes at room temperature. The mixture was further extracted with ethyl acetate (4 x 10 mL), and the combined organic layers were concentrated *in vacuo*, before being dissolved in 80% acetonitrile in water and purified by semi-preparative HPLC using Gradient 3. Purified peptide was lyophilised and isolated as a yellow powder. (Isolated yield (56%)).

^{1}H NMR (CD_3OD, 600 MHz) δ 8.84 (d, *J* = 8.4 Hz, 1H, NH-N17), 7.77 (d, *J* = 2.2 Hz, 1H, CH-C58), 7.56 – 7.50 (m, 2H, CH-C33 & CH-C36), 7.44 (dd, *J* = 8.6, 2.2 Hz, 1H, CH-C62), 7.13 (s, 1H, CH-C32), 7.09 – 7.00 (m, 3H, CH-C61, CH-C34 & CH-C35), 6.16 – 6.10 (m, 1H, CH-C65), 5.23 – 5.15 (m, 2H, CH_2-C66), 4.98 – 4.89 (m, 2H, CH-C5 & CH-C22), 4.89 – 4.86 (m, 1H, CH-C46), 4.72 (dd, *J* = 11.8, 3.3 Hz, 1H, CH-C15), 4.65 – 4.56 (m, 3H, CH-C3 CH-C12 & CH-C55), 3.45 (dd, *J* = 13.3, 11.2 Hz, 1H, CH-C6), 3.18 – 3.13 (m, 1H, CH-C6), 3.13 – 3.06 (m, 2H, CH-C56 & CH-C26), 2.90 – 2.82 (m, 1H, CH-C26), 2.74 (dd, *J* = 13.3, 4.7 Hz, 1H, CH-C56), 2.70 (s, 3H, CH_3-C49), 2.64 (s, 3H, CH_3-C14), 2.35 (t, *J* = 2.5, 1H, CH-C69), 1.95 (ddd, *J* = 13.7, 7.6, 5.7 Hz, 1H, CH-C25), 1.87 – 1.78 (m, 2H, CH-C19), 1.77 – 1.67 (m, 7H, CH-

C38, CH_3-C68 & CH_3-C67), 1.65 – 1.59 (m, 1H, CH-C50), 1.58 – 1.51 (m, 1H, CH-C25), 1.51 – 1.45 (m, 1H, CH-C41), 1.22 (d, *J* = 6.9 Hz, 3H, CH_3-C47), 1.14 – 1.05 (m, 1H, CH-C51), 1.02 – 0.88 (m, 12H, CH_3-C39, CH_3-C40, CH_3-C42 & CH_3-C43), 0.37 (d, *J* = 6.7 Hz, 3H, CH_3-C53 or CH_3-C52), 0.16 (d, *J* = 6.7 Hz, 3H, CH_3-C52 or CH_3-C53), -0.64 – -0.7 (m, 1H, CH-C50).

^{13}C NMR (CD_3OD, 151 MHz) δ 175.02 (C11), 174.47 (C34), 173.38 (C25), 172.61 (C30), 172.18 (C16), 171.39 (C3), 169.64 (C20), 154.62 (C44), 145.43 (C65), 139.28 (C46), 136.94 (C57), 135.51 (C43), 130.45 (C56), 129.89 (C41), 126.77 (C42), 125.55 (C59), 122.15 (C62), 121.10 (C45), 120.35 (C61), 119.48 (C60), 115.31 (C63), 114.10 (C68), 108.94 (C55), 79.59 (C51), 73.02 (C69), 60.23 (C66), 60.20 (C2), 59.82 (C22), 55.78 (C17), 55.05 (C36), 52.26 (C31), 51.83 (C28), 46.64 (C12), 44.63 (C37), 38.76 (C23), 38.43 (C1), 38.40 (C40), 29.79 (C10 & C26), 28.53 (C67 or C68), 28.43 (C67 or C68), 28.09 (C39), 25.96 (C52), 25.76 (C5), 25.48 (C48), 23.53 (C7 or C8), 23.49 (C50 or C49), 23.00 (C38), 22.99 (C53 or C54), 22.91 (C7 or C8), 22.40 (C53 or C54), 20.92 (C49 or C50), 17.06 (C15).
This cyclic peptide has rotamers, only major peaks are assigned.
HRMS (ESI) calc. for $C_{53}H_{73}N_9O_{10}$ calc. for $[M+H]^+$: 996.5558 m/z, found: 996.5568 m/z.
Purification: Purified through semi-preparative HPLC using Gradient 3.

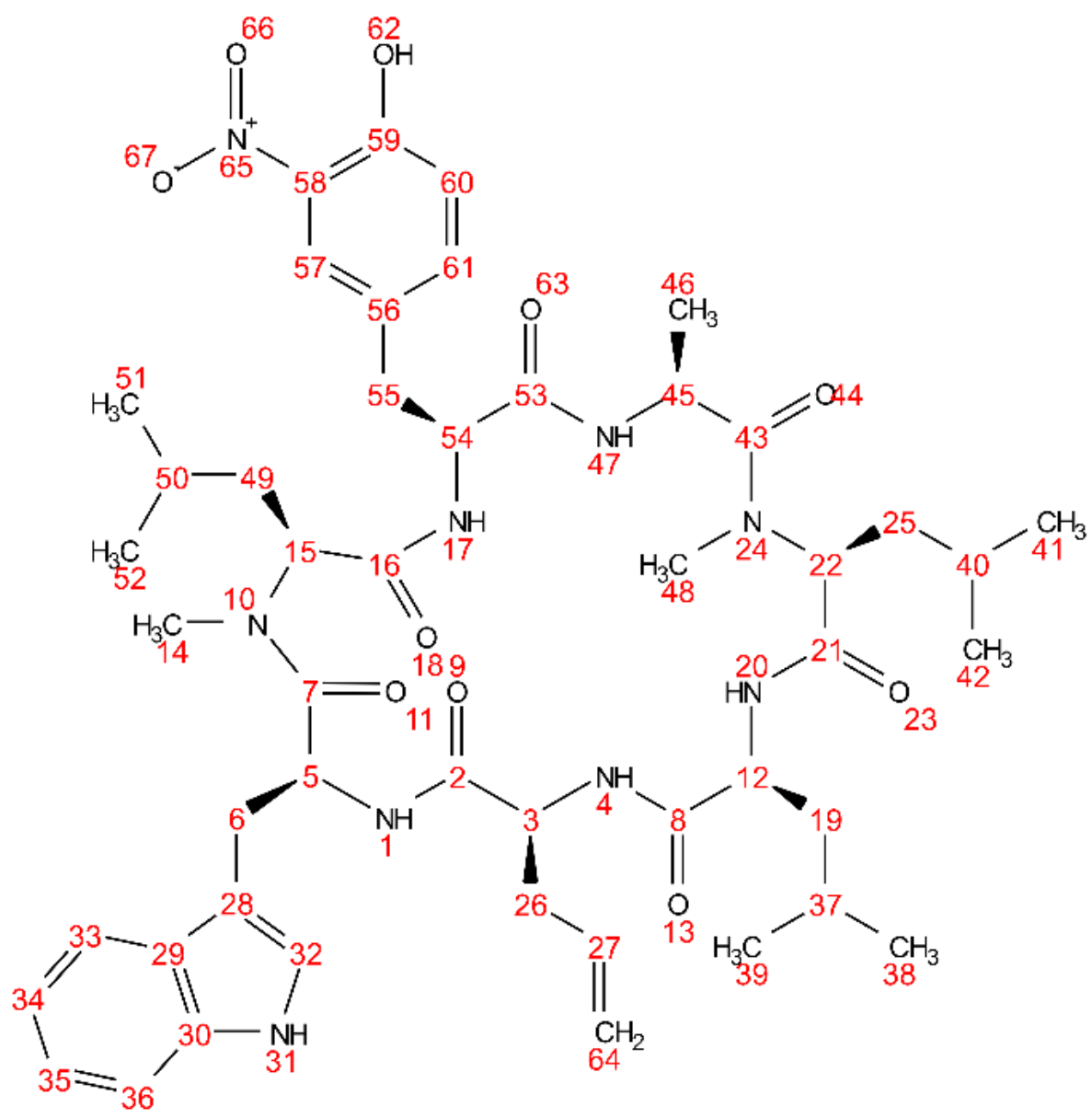

6

Peptide 6: Purified and dried linear precursor hydrazide peptide **6-linear** (16.15 µmol, 15 mg, 1 equiv.) was dissolved in 10 mL cyclisation buffer and sodium nitrite stock solution (300 uL) was added at -15 °C under nitrogen. The solution was stirred for 20 minutes at -15 °C. The mixture was gently neutralised using saturated sodium bicarbonate (~1000 µL) to pH 7.5. 15 mL of ethyl acetate was added, and the mixture stirred vigorously for 45 minutes at room

temperature. The mixture was further extracted with ethyl acetate (4 x 10 mL) and the combined organic layers were concentrated *in vacuo*, before being dissolved in 80% acetonitrile in water and purified by semi-preparative HPLC using Gradient 5. Purified peptide was lyophilised. (Isolated yield (34%)).

^{1}H NMR (CD_3OD, 700 MHz) δ 7.81 (d, 1H, CH-C57), 7.52 (d, 1H, CH-C33), 7.43 (dd, 1H, CH-C61), 7.36 (d, 1H, CH-C36), 7.12 (t, 1H CH-C34), 7.08 (d, 1H, CH-C60), 7.06 – 7.00 (m, 2H, CH-C32 & CH-C35), 5.59 – 5.66 (m, 1H, CH-C27), 5.14 – 5.07 (m, 2H, CH-C64), 4.96 – 5.00 (m, 1H, CH-C5), 4.89 – 4.80 (m, 1H, CH-C45, overlap with water), 4.70 – 4.66 (m, 1H, CH-C3), 4.63 – 4.57 (m, 4H, CH-C12, CH-C15, CH-C22, CH-C54), 3.48 (dd, 1H, CH-C6), 3.17 (dd, 1H, CH-C6), 3.08 (dd, 1H, CH-C55), 2.86 – 2.82 (m, 1H, CH-C26), 2.75 (d, 1H, CH-C55), 2.74 (s, 3H, CH-C48), 2.60 – 2.57 (m, 1H, CH-C26), 2.55 (s, 3H, CH-C14), 1.96 – 2.01 (m, 1H, CH-C25), 1.72 – 1.80 (m, 3H, CH-C19 & CH-C37), 1.59 – 1.54 (m, 2H, CH-C40 & CH-C49), 1.48 – 1.52 (m, 1H, CH-C25), 1.25 (d, 3H, CH-C46), 1.02 – 1.04 (m, 1H, CH-C50), 0.97 – 1.01 (m, 12H, CH-C38, CH-C39, CH-C41 & CH-C42), 0.44 (d, 3H, CH-C51 or CH-C52), 0.25 (d, 3H, CH-C51 or CH-C52), -0.53 (t, 1H, CH-C49).

^{13}C NMR (CD_3OD, 151 MHz) δ 175.15 (CH-C43), 173.86 (CH-C7), 173.28 (CH-C2), 173.20 (CH-C8), 172.53 (CH-C53), 171.61 (CH-C21), 169.85 (CH-C16), 154.59 (CH-C59), 139.21 (CH-C61), 137.99 (CH-C30), 135.50 (CH-C58), 133.12 (CH-C27), 129.82 (CH-C56), 128.51 (CH-C29), 126.74 (CH-C57), 124.61 (CH-C32), 122.77 (CH-C35), 121.07 (CH-C34), 120.43 (CH-C60), 120.18 (CH-C64), 119.21 (CH-C33), 112.69 (CH-C36), 110.33 (CH-C28), 60.09 (CH-C22), 59.98 (CH-C15), 55.65 (CH-C12), 54.98 (CH-C54), 53.30 (CH-C3), 52.18 (CH-C5), 46.86 (CH-C45), 44.55 (CH-C37), 38.60 (CH-C25), 38.07 (CH-C49), 37.44 (CH-C55), 34.95 (CH-C26), 28.53 (CH-C48), 28.48 (CH-C14), 26.00 (CH-C6), 25.81 (CH-C19), 25.50 (CH-C50), 23.01 (CH-C51), 22.87 (CH-C40), 22.26 (CH-C38), 21.93 (CH-C39), 21.34 (CH-C41), 21.28 (CH-C42), 21.11 (CH-C52), 17.08 (CH-C46).

HRMS (ESI) calc. for $C_{48}H_{65}N_9O_{12}$ calc. for $[M+H]^+$: 962.5464 m/z, found: 962.5437 m/z.
Purification: Purified through semi-preparative HPLC using Gradient 5.

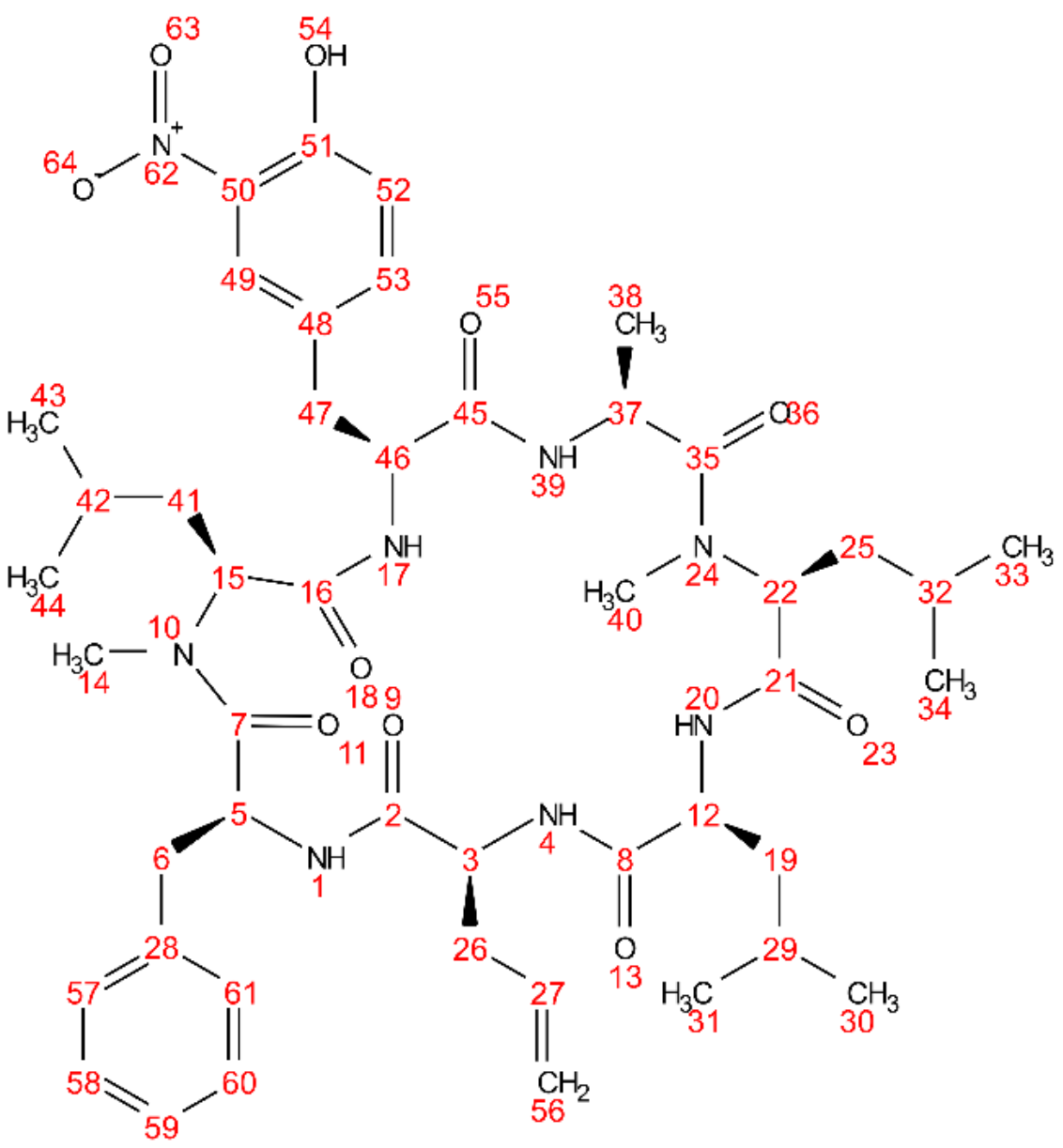

7

Peptide **7**: Purified and dried linear peptide hydrazide precursor peptide **7-linear** (16.26 µmol, 15 mg, 1 equiv.) was dissolved in 10 mL cyclisation buffer and sodium nitrite stock solution (300 uL) was added at -15 °C under nitrogen. The solution was stirred for 20 minutes at -15 °C. The mixture was gently neutralised using saturated sodium bicarbonate (~1000 µL) to pH 7.5. 15 mL of ethyl acetate was added, and the mixture stirred vigorously for 45 minutes at room temperature. The mixture was further extracted with ethyl acetate (4 x 10 mL) and the combined organic layers were concentrated *in vacuo*, before being dissolved in 80% acetonitrile in water and purified by semi-preparative HPLC using Gradient 5. Purified peptide was lyophilised. (Isolated yield (67%)).

^{1}H NMR (CD_3OD, 700 MHz) δ 7.82 (d, 1H, CH-C49), 7.54 (d, 1H, CH-C53), 7.34 – 7.30 (m, 2H, CH-C58 & CH-C60), 7.28 – 7.26 (m, 2H, CH-C57 & CH-C61), 7.24 – 7.22 (m, 1H, C59), 7.09 (d, 1H, CH-C52), 5.66 – 5.60 (m, 1H, CH-C27), 5.11 – 5.06 (m, 2H, CH-C56), 4.98 – 4.93 (m, 1H, CH-C5), 4.85 (m, 1H, CH-C37 overlap with methanol), 4.69 – 4.66 (m, 1H, CH-C3), 4.64 – 4.61 (m, 1H, CH-C46), 4.59 – 4.54 (m, 1H, CH-C12), 3.28 – 3.24 (m, 2H, CH-C6), 3.11 – 3.07 (m, 1H, CH-C26), 2.84 – 2.80 (m, 1H, CH-C26), 2.78 – 2.76 (m, 1H, CH-C47), 2.75 (s, 3H, CH-C40), 2.58 (s, 3H, CH-C14), 2.54 – 2.52 (m, 1H, CH-C47), 1.80 – 1.66 (m, 6H, CH-C19, CH-C25, CH-C29, CH-C32), 1.56 – 1.50 (m, 2H, CH-41), 1.27 (d, 3H, CH-C38), 1.05 – 1.01 (m, 1H, CH-C42), 0.99 – 0.91 (m, 12H, CH-C30, CH-C31, CH-C33 & CH-C34), 0.77 – 0.70 (m, 6H, CH-C44 & CH-C43).

^{13}C NMR (CD_3OD, 151 MHz) δ 174.20 (C35), 173.73 (C8), 172.63 (C7), 171.84 (C2), 171.59 (C45), 171.00 (C21), 170.65 (C16), 153.21 (C51), 137.83 (C28), 136.31 (C53), 134.12 (C50), 131.68 (C27), 129.22 (C48), 128.54 (C59), 128.34 (C58 & C60), 127.01 (C57 & C61), 125.36 (C49), 119.69 (C52), 119.00 (C56), 58.63 (C22), 58.56 (C15), 54.09 (C12), 53.45 (C3), 51.80 (C5), 51.19 (C46), 45.39 (C37), 42.93 (C19), 37.25 (C6), 37.16 (C25), 36.85 (C41), 36.45

(C47), 35.76 (C26), 28.48 (C14), 28.25 (C40), 24.60 (C32), 24.40 (C29), 22.33 (C31), 22.14 (C30), 21.60 (C43 or C44), 22.07; 21.98; 21.79; 21.57 (C30, C31, C33, C34), 21.47 (C42), 20.23 (C43 or C44), 15.63 (C38).

HRMS (ESI) calc. for $C_{46}H_{64}N_8O_{12}$ calc. for $[M+H]^+$: 891.4980 m/z, found: 891.5040 m/z.
Purification: Purified through semi-preparative HPLC using Gradient 5.

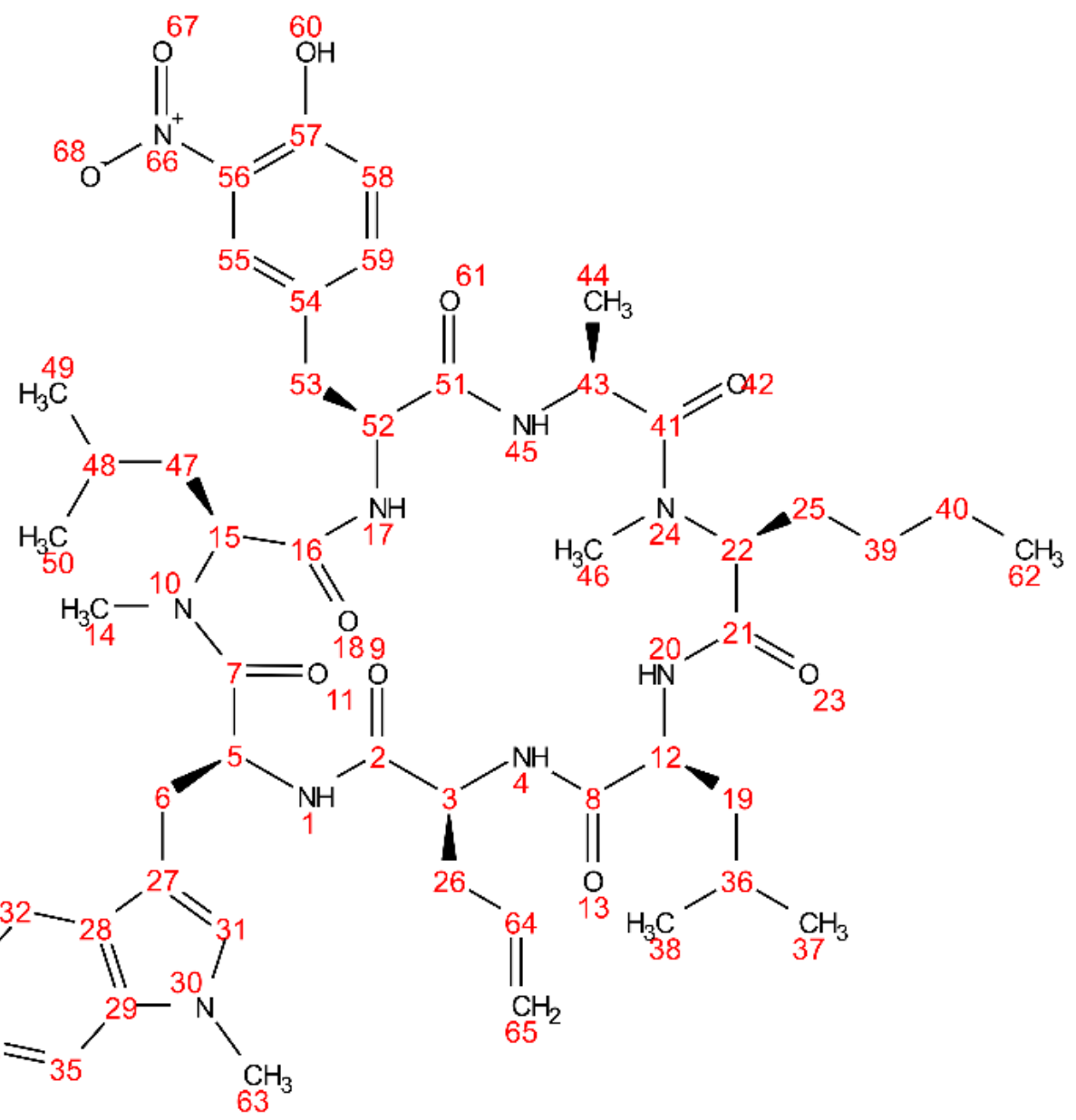

15

Peptide 15: Purified and dried linear hydrazide precursor peptide **15-linear** (31 µmol, 29 mg, 1 equiv.) was dissolved in 10 mL cyclisation buffer and sodium nitrite stock solution (120 µL) was added -15 °C under nitrogen. The solution was stirred for 20 minutes at -15 °C. The mixture was gently neutralised using saturated sodium bicarbonate to pH 7. 15 mL of ethyl acetate was added, and the mixture stirred vigorously for 20 minutes at room temperature. The mixture was further extracted with ethyl acetate (3 x 15 mL) and the combined organic layers were concentrated *in vacuo*, before being dissolved in 80% acetonitrile in water and purified by preparative HPLC using Gradient 5. Purified peptide was lyophilised. (Isolated yield (59%)).

^{1}H NMR (CD_3OD, 600 MHz) δ 7.85 – 7.81 (m, 1H, CH-C55), 7.54 – 7.52 (d, 1H, CH-C35), 7.46 – 7.42 (dd,1H CH-C58), 7.38 – 7.36 (d, 1H, CH-C59), 7.22 – 7.17 (m, 1H, CH-C33), 7.11 – 7.07 (m, 1H, CH-C32), 7.07 – 7.04 (m, 1H, CH-C34), 6.98 – 6.96 (s, 1H, CH-C31), 5.65 – 5.59 (m, 1H, CH-C64), 5.14 – 5.08 (m, 2H, CH-C65), 5.05 – 5.02 (m, 1H, CH-C52), 4.95 – 4.92 (dd, 1H, CH-C5), 4.90 – 4.86 (1H (overlap with water), CH-C22), 4.84 – 4.79 (m, 1H, CH-C43), 4.69 – 4.66 (m, 1H, CH-C3), 4.64 – 4.59 (m, 2H, CH-C12 & CH-C15), 3.80 – 3.72 (s, 3H, CH-C63), 3.48 – 3.44 (t, 1H, CH-C6), 3.16 – 3.13 (dd, 1H, CH-C6), 3.10 – 3.06 (dd, 1H, CH-C53), 2.89 – 2.84 (m, 1H, CH-C26), 2.83 – 2.79 (dd, 1H, CH-C39), 2.77 – 2.71 (m, 3H, CH-C46), 2.61 – 2.55 (m, 3H, CH-C14), 1.98 – 1.94 (m, 1H, CH-C25), 1.80 – 1.77 (m, 1H, CH-C36), 1.76 – 1.70 (m, 2H, CH-C19), 1.60 – 1.55 (m, 1H, CH-C25), 1.46 – 1.35 (m, 2H,

CH-C47 & CH-C36), 1.33 – 1.30 (m, 1H, CH-C40), 1.26 – 1.18 (m, 3H, CH-C44), 1.07 – 1.02 (m, 1H, CH-C48), 1.02 – 1.79 (m, 9H, CH-C38 & CH-C37 & CH-C62), 0.69 – 0.58 (dd, 2H, CH-C39), 0.49 – 0.48 (m, 3H, CH-C50), 0.26 – 0.29 (m, 3H, CH-C49), -0.55 – -0.60 (t, 1H, CH-C47).

^{13}C NMR (CD3OD, 151 MHz) δ 173.96 (C8 or C16), 172.91 (C2), 172.43 (C3 or C12 or C15), 171.78 (C21 or C7 or C51), 171.08 (C21 or C7 or C51), 170.63 (C41), 170.28 (C8 or C16), 168.37 (C21 or C7 or C51), 153.19 (C29), 138.57 (C) 137.78 (C58), 137.09 (C28), 134.09 (C56 or C57), 131.69 (C65), 133.17 (C27), 127.67 (C31), 125.30 (C55), 121.57 (C32), 119.67 (C33 or C34 or C35), 119.19 (C64) 119.05 (C33 or C34 or C35), 118.93 (C33 or C34 or C35), 118.21 (C59), 109.29 (C58), 60.35 (C43), 58.47 (C3), 54.29 (C12 or C15), 50.75 (C22), 45.45 (C12 or C15), 43.31 (C19), 36.88 (C36), 36.51 (C48), 36.06 (C26), 31.40 (C63), 28.36 (C14 or C46) 28.05 (C14 or C46), 27.41 (C25), 24.59 (C47 or C36), 23.96 (C47 or C36), 22.28 (C37 or C38 or C62), 22.01 (C40), 21.98 (C50), 21.81 (C37 or C38 or C62), 20.51 (C37 or C38 or C62), 19.78 (C39), 15.71 (C44), 12.87 (C49).

HRMS (ESI) calc. for $C_{46}H_{64}N_8O_{12}$ calc. for $[M+H]^+$: 944.5240 m/z, found: 944.5269 m/z.
Purification: Purified through semi-preparative HPLC using Gradient 5.

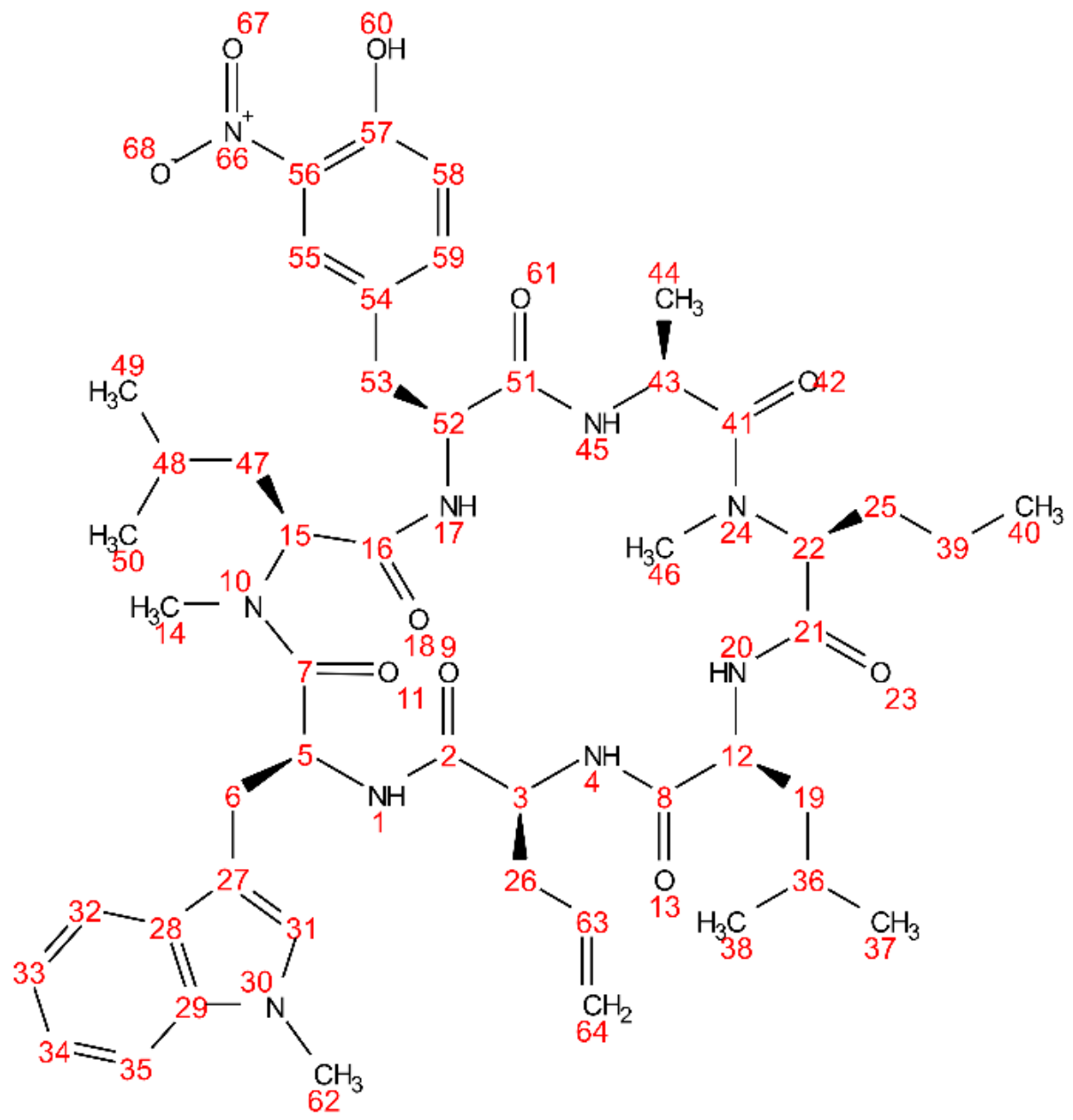

16

Peptide 16: Purified and dried linear hydrazide precursor peptide **16-linear** (16.15 µmol, 15 mg, 1 equiv.) was dissolved in 10 mL cyclisation buffer and sodium nitrite stock solution (300 uL) was added at -15 °C under nitrogen. The solution was stirred for 15 minutes at -15 °C. The mixture was gently neutralised using saturated sodium bicarbonate (~1000 µL) to pH 7.5. 15 mL of ethyl acetate was added, and the mixture stirred vigorously for 40 minutes at room temperature. The mixture was further extracted with ethyl acetate (3 x 10 mL), and the combined organic layers were concentrated *in vacuo*, before being dissolved in 80%

acetonitrile in water and purified by semi-preparative HPLC using Gradient 4. Purified peptide was lyophilised. (Isolated yield (72%)).

^{1}H NMR (CD_3OD, 700 MHz) δ 7.77 (d, 1H, CH- C55), 7.55 – 7.53 (d, 1H, CH-C32), 7.41 – 7.43 (dd, 1H, CH-C59), 7.35 – 7.36 (d, 1H, CH-C35), 7.14 – 7.19 (m, 1H, CH-C33), 7.03 – 7.08 (m, 1H, CH-C58), 6.99 – 7.03 (m , 1H, C34), 6.95 (s, 1H, C31), 5.57 – 5.64 (m, 1H, C63), 5.05 – 5.15 (m, 3H, CH-C52 & CH-C64), 4.98 – 5.03 (m, 1H, CH-C5), 4.89 – 4.93 (dd, 1H, CH-C22), 4.64 – 4.69 (m, 2H, CH-C3 & CH-C12), 4.58 – 4.60 (m, 1H, CH-C15), 4.49 – 4.52 (m, 1H, CH-C43), 3.75 (s, 3H, CH-C62), 3.40 – 3.45 (dd, 1H, CH-C6), 3.11 – 3.14 (dd, 1H, CH-C6), 3.03 – 3.07 (dd, 1H, CH-C53), 2.84 – 2.86 (m, 1H, CH-C26), 2.72 – 2.75 (dd, 1H, CH-C53), 2.67 (d, 3H, CH-C46), 2.59 – 2.61 (m, 1H, CH-C26), 2.57 (s, 3H, CH-C14), 1.81 – 1.87 (m, 2H, CH-C25), 1.70 – 1.76 (m, 3H, CH-C19 & CH-C36), 1.61 – 1.69 (m, 2H, CH-C39), 1.55 – 1.59 (m, 1H, CH-C47), 1.11 – 1.13 (d, 3H, CH-C44), 1.00 – 1.04 (m, 1H, CH-C48), 0.94 – 0.98 (m, 6H, CH-C37 & CH-C38), 0.93 – 0.94 (m, 3H, CH-C40), 0.41 (d, 3H, CH-C49 or CH-C50), 0.13 (d, 3H, CH-C49 or CH-C50), -0.62 (ddd, 1H, CH-C47).

^{13}C NMR (CD_3OD, 151 MHz) δ 175.75 (C41), 175.29 (C8), 174.34 (C7), 173.79 (C2), 173.09 (C51), 172.35 (C21), 169.76 (C16), 154.60 (C57), 139.17 (C29), 138.47 (C59), 135.49 (C56), 133.07 (C64), 130.76 (C63), 129.76 (C54), 129.30 (C31), 129.03 (C28), 126.71 (C55), 122.99 (C34), 121.09 (C33), 120.37 (C58), 119.65 (C52), 119.39 (C32), 110.72 (C27), 109.91 (C35), 59.84 (C15), 53.30 (C3), 52.01 (C5), 49.68 (C22), 46.66 (C12), 44.76 (C43), 43.23 (C19), 37.97 (C53), 37.47 (C47), 36.52 (C26), 31.27 (C62), 29.74 (C39), 29.48 (C25), 28.48 (C46), 28.48 (C14), 26.12 (C6), 25.32 (C36), 23.74 (C48), 21.94 (C50), 21.19 (C37), 20.79 (C38), 20.61 (C40), 18.11 (C49), 16.72 (C44).

HRMS (ESI) calc. for $C_{46}H_{64}N_8O_{12}$ calc. for $[M+H]^+$: 930.5189 m/z, found: 930.5157 m/z.
Purification: Purified through semi-preparative HPLC using Gradient 4.

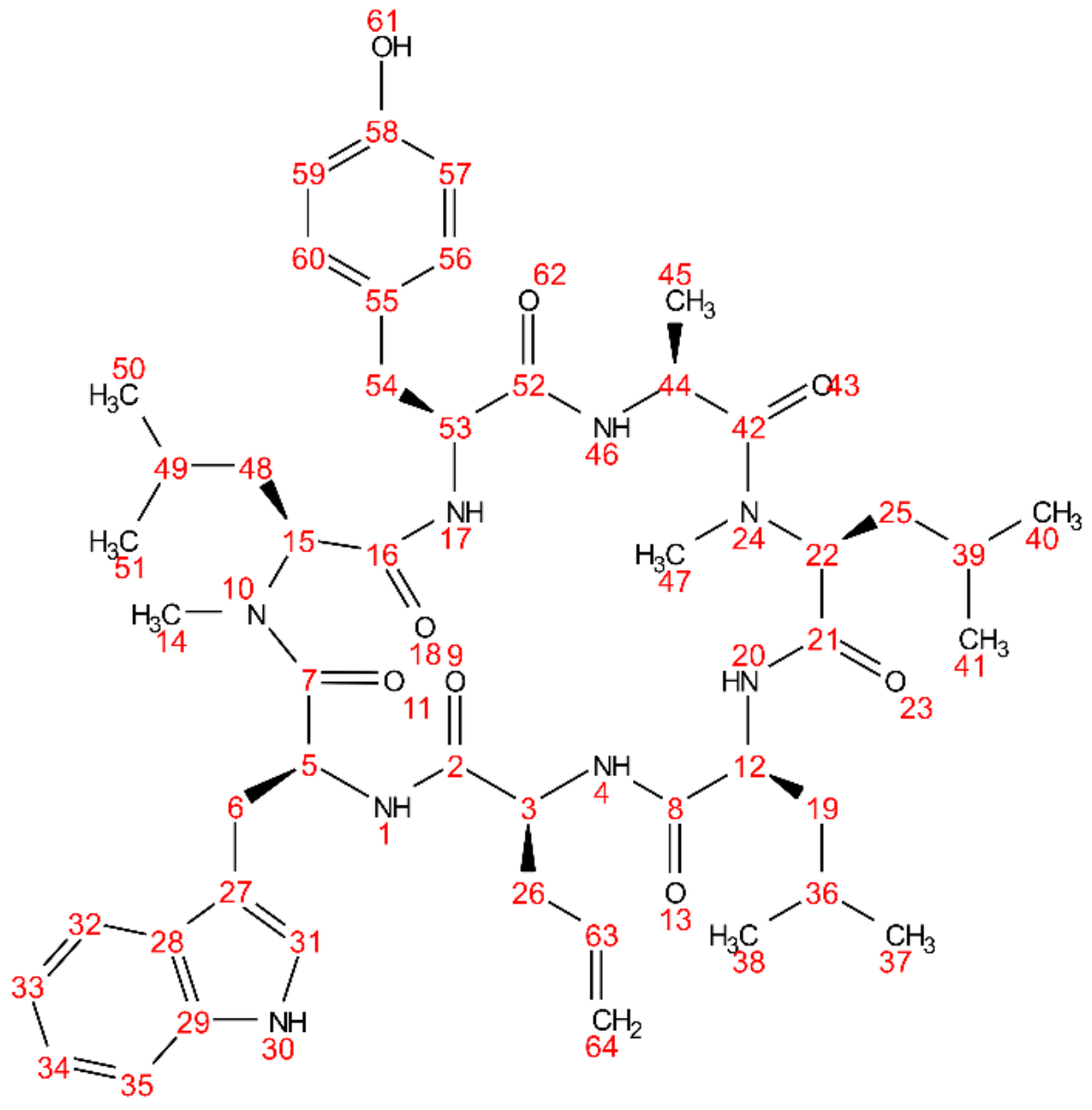

10

Peptide 10: Purified and dry linear hydrazide precursor peptide **10-linear** (3.4 mg, 3.83 µmol, 1 equiv.) was dissolved in 1.5 mL cyclisation buffer and sodium nitrite stock solution (23 uL) was added at -15 °C under nitrogen. The solution was stirred for 15 minutes at -15 °C. The mixture was gently neutralised using saturated sodium bicarbonate to pH 7. 1.5 mL of ethyl acetate was added, and the mixture stirred vigorously for 15 minutes at room temperature. The mixture was further extracted with ethyl acetate (3 x 15 mL), and the combined organic layers were concentrated *in vacuo*, before being dissolved in 80% acetonitrile in water and purified by semi-preparative HPLC using Gradient 4. Purified peptide was lyophilised.

^{1}H NMR (CD_3OD, 600 MHz) δ 7.50 (d, 1H, CH-C32), 7.34 (d, 1H, CH-C35), 7.12 – 7.09 (m, 1H, CH-C33), 7.02 – 6.99 (m, 2H, CH-C31 & CH-C34), 6.93 – 6.94 (m, 2H, CH-C56 & CH-C60), 6.65 – 6.66 (m, 2H, CH-C57 & CH-C59), 5.58 – 5.64 (m, 1H, CH-C63), 5.07 – 5.13 (m, 2H, CH-C64), 4.97 (dd, 1H, CH-C22), 4.93 (dd, 1H, CH-C5), 4.66 – 4.63 (m, 2H, CH-C15 & CH-C53), 4.60 – 4.56 (m, 2H, CH-C12 & CH-C3), 3.46 (dd, 1H, CH-C6) 3.16 (dd, 1H, CH-C6), 3.00 (dd, 1H, CH-C26), 2.87 – 2.91 (m, 1H, CH-C54), 2.73 (s, 3H, CH-C47), 2.59 – 2.61 (m, 1H, CH-C26), 1.94 – 1.97 (m, 1H, CH-C25), 1.71 – 1.75 (m, 1H, CH-C36), 1.61 – 1.63 (m, 1H, CH-C19), 1.49 – 1.53 (m, 3H, CH-C25 & CH-C39 & CH-C48), 1.28 (d, 3H, CH-C45), 1.01 (dd, 1H, CH-C49), 0.98 – 1.00 (m, 12H, CH-C37 & CH-C38 & CH-C40 & CH-C41), 0.42 (d, 3H, CH-C50), 0.25 (d, 3H, CH-C51), -0.57 (ddd, 1H, CH-C48).

^{13}C NMR (CD_3OD, 151 MHz) δ 175.62 (C42), 175.16 (C2), 174.16 (C7), 173.86 (C8), 172.52 (C52), 171.62 (C21), 169.84 (C16), 154.59 (C58), 137.99 (C29), 133.11 (C63), 130.79 (C56 & C60), 128.71 (C55), 128.50 (C28), 124.61 (C31), 122.76 (C34), 121.07 (C33), 120.18 (C64), 119.21 (C32), 117.34 (C57 & C59), 112.69 (C35), 110.32 (C27), 60.10 (C22), 59.98 (C15), 55.65 (C53), 54.98 (C12), 53.30 (C3), 52.18 (C5), 46.86 (C44), 44.55 (C19), 38.59 (C54), 38.07 (C25), 37.83 (C26), 37.44 (C48), 30.21 (C47), 29.77 (C14), 26.00 (C6), 25.81 (C36),

25.50 (C39), 23.73 (C49), 23.48 (C40), 23.41 (C38), 23.01 (C41), 22.87 (C50), 21.93 (C37), 21.13 (C51), 17.08 (C45).

HRMS (ESI) calc. for $C_{48}H_{68}N_8O_8$ calc. for $[M+H]^+$: 885.5239 m/z, found: 885.5259 m/z.
Purification: Purified through semi-preparative HPLC using Gradient 4.

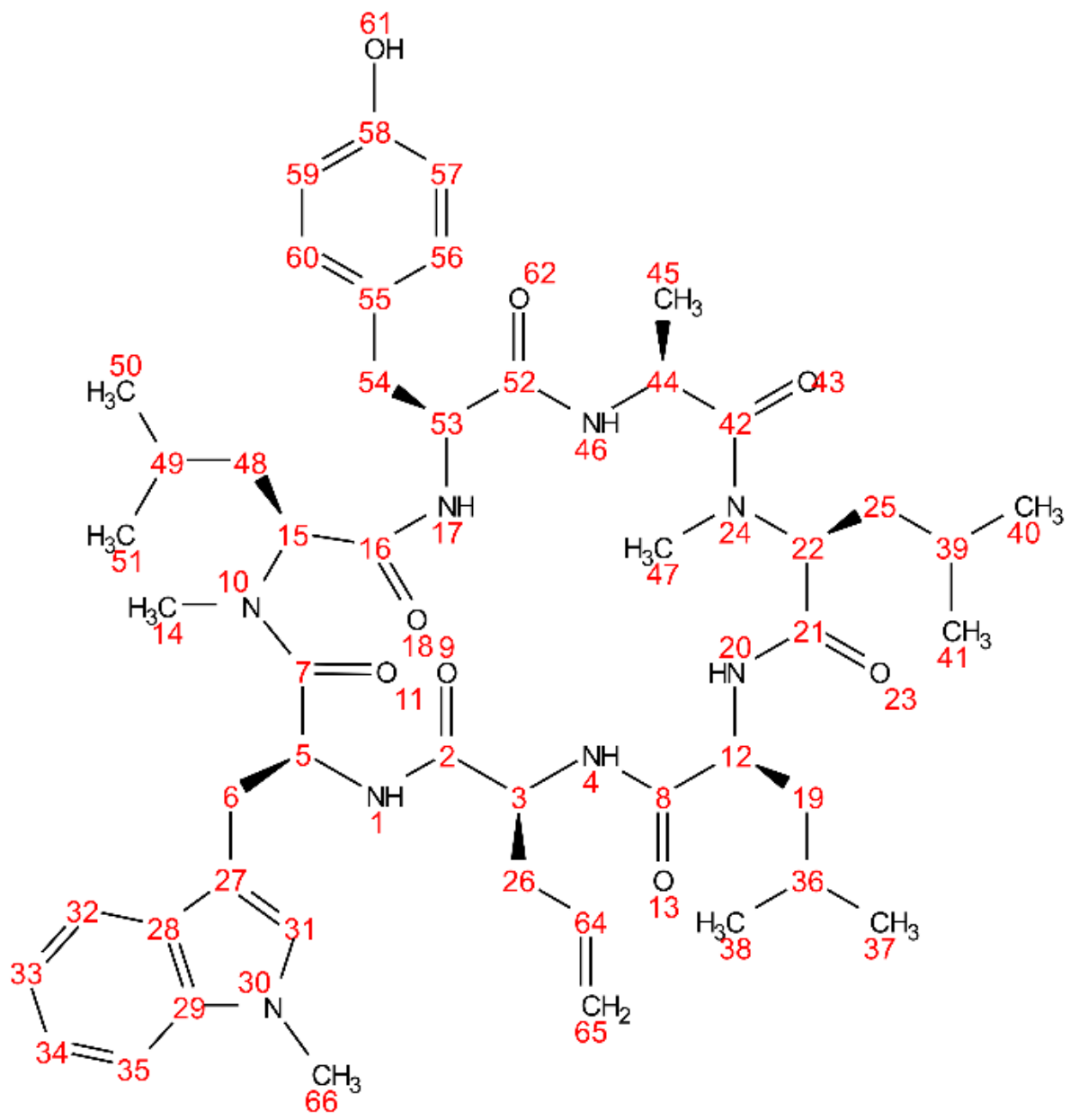

9

Peptide 9: Purified and dried linear hydrazide precursor peptide **9-linear** (15 mg, 16.69 μmol, 1 equiv.) was dissolved in 10 mL cyclisation buffer and sodium nitrite stock (300 μL) was added at -15 °C under nitrogen. The solution was stirred for 20 minutes at -15 °C. The mixture was gently neutralised using saturated sodium bicarbonate (~1000 μL) to pH 8. 20 mL of ethyl acetate was added, and the mixture was stirred vigorously for 60 minutes at room temperature. The mixture was further extracted with ethyl acetate (4 x 10 mL), and the combined organic layers were concentrated *in vacuo*, before being dissolved in 80% acetonitrile in water and purified by semi-preparative HPLC using Gradient 5. Purified peptide was lyophilised. (Isolated yield (77%)).

^{1}H NMR (CD_3OD, 600 MHz) δ 7.52 (d, 1H, CH-C32), 7.36 (m, 1H, CH-C35), 7.22 – 7.17 (m, 1H, CH-C33), 7.10 – 7.04 (m, 1H, CH-C34), 6.97 – 6.95 (m, 3H, CH-C31, CH-C56, CH-C60), 6.64 – 6.71(m, 2H, CH-C59 & CH-C58), 5.66 – 5.60 (m, 1H, CH-C64), 5.14 – 5.09 (m, 2H, CH-C65), 4.98 (s, 1H, CH-C22), 4.93 (dd, 1H, CH-C5), 4.83 – 4.78 (m, 1H, CH-C44, overlap with water), 4.68 – 4.62 (m, 2H, CH-C3 & CH-C53), 4.63 – 4.55 (m, 2H, CH-C15 & CH-CH12), 3.76 (s, 3H, CH-CH66), 3.47 (dd, 1 H, CH-C6), 3.15 (dd, 1H, CH-C6), 3.04 – 2.99 (m, 1H, CH-C26), 2.97 – 2.94 (dd, 1 H, CH-C54), 2.92 – 2.89 (m, 1H, CH-C54), 2.74 (s, 3H, CH-C47), 2.58 – 2.62 (m, H, CH-C26), 2.46 (s, 3H, CH-C14), 1.77 – 1.71 (m, 1H, CH-C36), 1.64 (m, 2H, CH-C19), 1.60 – 1.50 (m, 4H, CH-C48, CH-C25, CH-39), 1.22 (s, 3H, CH-C45),1.06 – 1.04 (m, 1H, CH-C49), 1.04 – 0.98 (m, 12H, CH3-40, CH3-C41, CH3-C38 & CH3-C37), 0.45 (d, 3H, CH-C50 or CH-C51), 0.24 (d, 3H,CH-C50 or CH-C51), -0.54 (ddd, 1 H, CH-C48).

^{13}C NMR (CD_3OD, 151 MHz) δ 175.50 (C42), 173.81 (C8), 173.12 (C7), 172.35 (C2), 171.55 (C52), 169.98 (C21), 169.59 (C16), 157.26 (C58), 138.49 (C29), 133.04 (C64), 131.30 (31), 129.07 (C60 & C56), 129.02 (28), 128.50 (C55), 122.93 (C33), 120.49 (C34), 120.25 (C65), 118.94 (C32), 116.31 (C57 & C59), 110.61 (C35), 109.82 (C27), 60.17 (C22), 59.97 (C15), 54.90 (C53), 53.18 (C12), 52.29 (C3), 50.94 (C5), 46.78 (C44), 43.39 (C36), 38.50 (C48), 38.17(C25), 37.34 (C26), 37.15 (C54), 31.48 (C66), 29.98 (C14), 29.89 (C47), 25.95 (C6), 25.74 (C49), 25.42 (C39), 23.72 (C50), 23.45 (C19), 23.41; 23.33; 22.94; 21.61 (C37, C38, C40 & C41),21.16 (C51), 17.26 (C45).

HRMS (ESI) calc. for $C_{49}H_{70}N_8O_8$ calc. for $[M+H]^+$: 899.5395 m/z, found: 899.5389 m/z.

Purification: Purified through semi-preparative HPLC using Gradient 5.

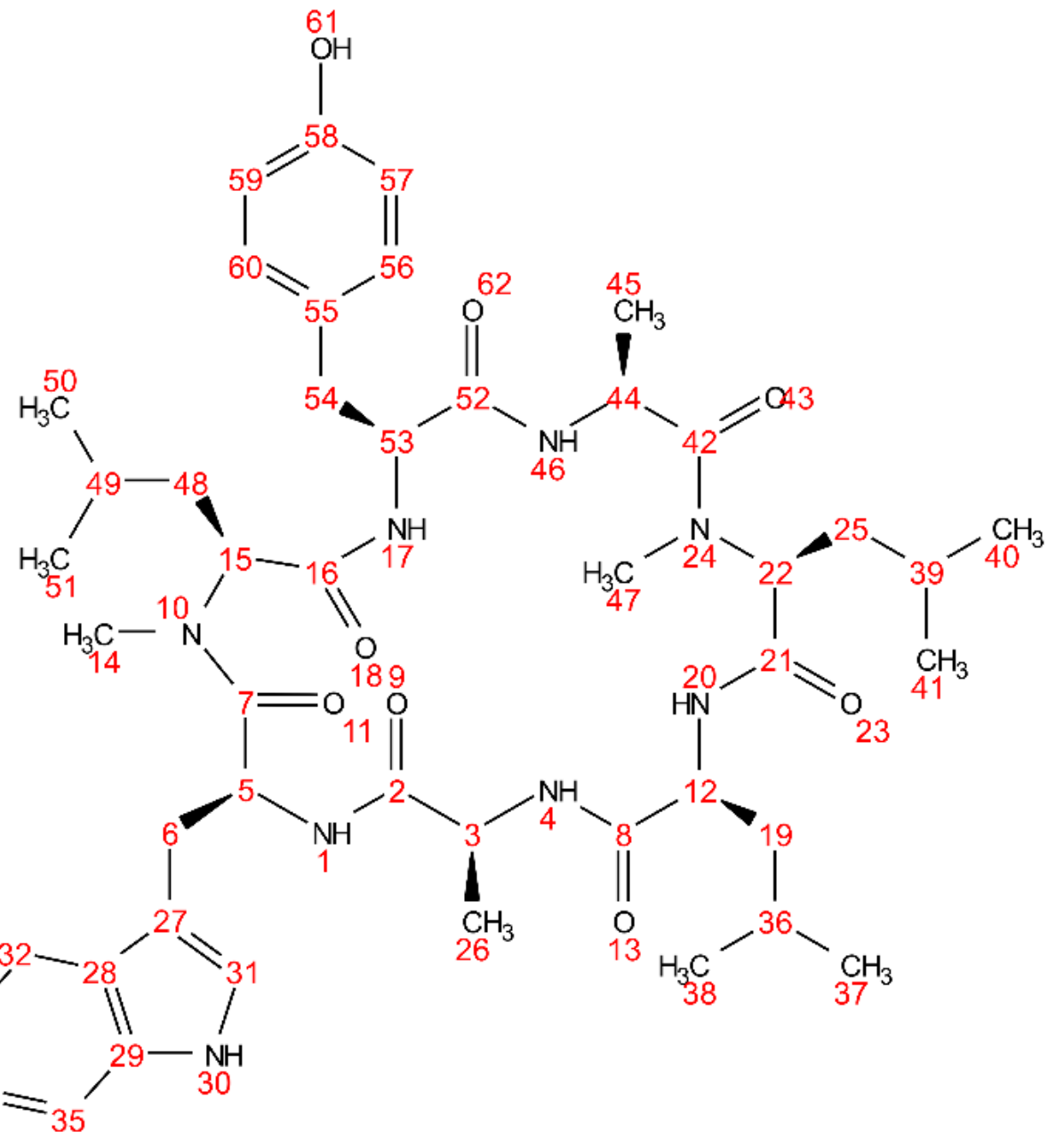

11

Peptide 11: Purified dry hydrazide linear peptide **11-linear** (4.5 mg, 5.065 µmol, 1 equiv.) was dissolved in 2 mL cyclization buffer and sodium nitrite stock (40 µL) was added at -15 °C under nitrogen. The solution was stirred for 15 minutes at -15 °C. The mixture was gently neutralised using saturated sodium bicarbonate to pH 7. 1.5 mL of ethyl acetate was added, and the mixture was stirred vigorously for 15 minutes at room temperature. The mixture was further extracted with ethyl acetate (3 x 15 mL), and the combined organic layers were concentrated *in vacuo*, before being dissolved in 80% acetonitrile in water and purified through semi-preparative HPLC using Gradient 4. Purified peptide was lyophilised. (Isolated yield (62%)).

^{1}H NMR (CD_3OD, 700 MHz) δ 7.52 (d, 1H, CH-C32), 7.38 – 7.34 (m, 1H, CH-C35), 7.14 – 7.09 (m, 1H, CH-C34), 7.04 – 7.01 (m, 2H, CH-C31 & CH-C33), 6.94 – 6.92 (m, 2H, CH-C56 & CH-C60), 6.69 – 6.65 (m, 2H, CH-C57 & CH-C59), 4.98 (dd, 1H, CH-C22), 4.94 (dd, 1H, CH-C5), 4.73 (dd, 1H, CH-C44), 4.70 – 4.66 (m, 1H, CH-C53), 4.60 – 4.53 (m, 3H, CH-C12 & CH-C15 & CH-C3), 3.44 (dd, 1H, CH-C6), 3.19 – 3.15 (m, 1H, CH-C6), 2.97 (dd, 1H, CH-

C54), 2.75 (s, 3H, CH-C47), 2.63 (dd, 1H, CH-C54), 2.38 (s, 3H, CH-C14), 1.99 – 1.95 (m, 1H, CH-C25), 1.70 – 1.57 (m, 5H, CH-C25 & CH-C39 & CH-C19 & CH-C36), 1.49 (d, 1H, CH-C48), 1.43 (d, 3H, CH-C26), 1.32 (d, 3H, CH-C45), 1.04 (dd, 1H, CH-C49), 1.01 – 0.99 (m, 12H, CH-C37, CH-C38, CH-C41 & CH-C40), 0.46 (dd, 3H, CH-C50 or CH-C51), 0.29 (dd, 3H, CH-C50 or CH-C51), -0.45 (ddd, 1H CH-C48).

^{13}C NMR (CD_3OD, 151 MHz) δ 175.64 (C42), 174.64 (C2), 174.33 (C7), 174.04 (C8), 172.50 (C52), 171.67 (C21), 170.67 (C16), 157.29 (C58), 137.97 (C29), 131.48 (C56 & C60), 128.53 (C55), 128.39 (C28), 124.59 (C31), 122.73 (C34), 120.17 (C33), 119.29 (C32), 116.30 (C57 & C59), 112.57 (C35), 110.32 (C27), 60.02 (C22), 59.84 (C15), 55.01 (C53), 54.61 (C12), 52.28 (C5), 49.70 (C3), 46.79 (C44), 43.34 (C19), 38.46 (C25), 38.19 (C54), 37.33 (C48), 29.75 (C47), 29.32 (C14), 28.73 (C6), 26.10 (C36), 25.69 (C39), 25.52 (C49), 23.54 (C38), 23.45 (C40), 23.23 (C51), 22.97 (C41), 21.78 (C37), 21.32 (C50), 19.38 (C26), 17.26 (C45).

HRMS (ESI) calc. for $C_{46}H_{66}N_8O_8$ calc. for $[M+H]^+$: 859. 5070 m/z, found: 859.5083 m/z.

Purification: Purified through semi-preparative HPLC using Gradient 4.

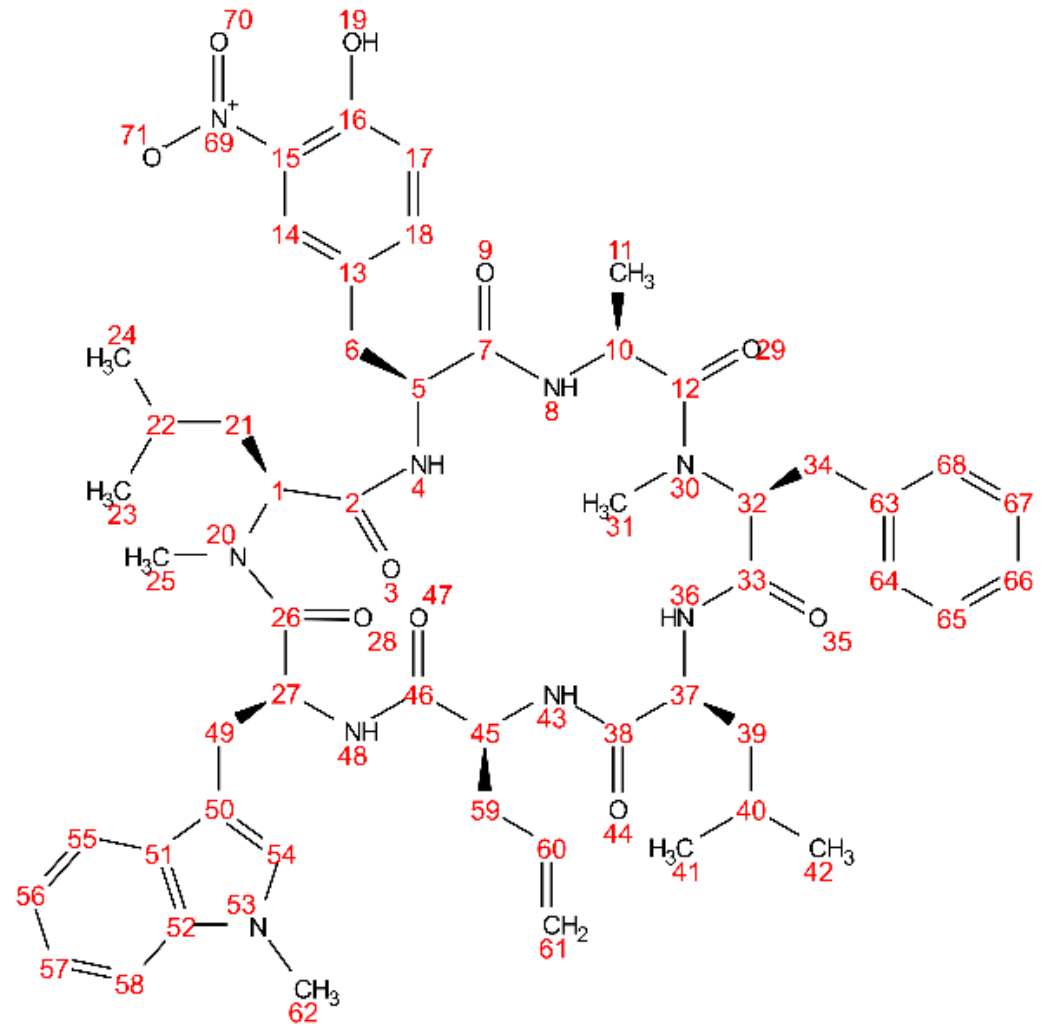

17

Peptide 17: Crude linear peptide hydrazide **17-linear** (38.5 mg, 38.14 μ mol, 1 equiv.) was dissolved in 10 mL cyclisation buffer and sodium nitrite stock solution (900 uL) was added at 15 °C under nitrogen. The solution was stirred for 15 minutes at -15 °C. The mixture was gently neutralised using saturated sodium bicarbonate (1200 μL) to pH 7.5. 15 mL of ethyl acetate was added, and the mixture stirred vigorously for 60 minutes at room temperature. The mixture was further extracted with ethyl acetate (3 x 15 mL), and the combined organic layers were concentrated *in vacuo*, before being dissolved in 50% acetonitrile in water and purified by semi-preparative HPLC using Gradient 5. Purified peptide was lyophilised and isolated as a yellow powder. (Isolated yield 38%).

^{1}H NMR (($(CD_3)_2SO$, 700 MHz) δ 9.32 (d, 1H, NH-N8), 8.86 (d, 1H, NH-N48), 8.54 – 8.50 (m, 2H, NH-N4 or N36 or N43), 7.57 (d, 1H, CH-C14), 7.50 – 7.48 (m, 2H , CH-C17 & CH-C55), 7.28 (d, 1H, CH-C64 or C68) 7.24 (m, 3H, CH-C65 & C66 & C67), 7.21 – 7.09 (m, 3H, CH-C18 & C57 & C64 or C68), 7.07 (s, 1H, CH-C54), 7.00 (ddd, 1H, CH-C58), 6.96 (d, 1H, H58), 5.47 (m, 1H, CH-C60), 4.99 – 4.93 (m, 2H, CH-C61), 4.81 (dt, 1H, CH-C27), 4.48 – 4.45 (m, 2H, CH-C45 & C5), 4.38 – 4.33 (m, 2H, CH- C32 & C10), 4.26 – 4.22 (m, 2H, CH-C1 & C37), 3.71 (s, 3H, CH3-C62), 3.26 (dd, 1H, CH-C49), 3.06 (dd, 1H, CH-49), 2.92 – 2.83 (m, 2H, CH2-C6), 2.71 (s, 3H, CH3-C25 or C31), 2.64 – 2.59 (m, 2H, CH2-C59), 2.58 – 2.52 (m, 2H, CH2-C34), 2.38 (s, 3H, CH3 – C25 or C31), 1.57 – 1.51 (m, 3H, CH3-C11), 1.48 (ddd, 1H, CH-C21), 0.99 (ddd, 1H, CH-C21), 0.84 (dd, 6H, CH3-C41 & C42), 0.44 (d, 3H, CH2-C39 & CH-C40), 0.26 (d, 3H, CH3-C23 or C24), 0.14 (d, 3H, CH3-C23 or C24), -0.24 (ddd, 1H, CH-C22).

^{13}C NMR (($(CD_3)_2SO$, 151 MHz) δ 172.98 (C2 or C38) 172.85 (C2 or C38), 171.74 (C26), 171.45 (C7), 171.28 (C15), 170.94 (C46), 169.27 (C12), 151.39 (C16), 137.03 (C13) 136.92 (C52), 136.75 (C57), 136.48 (C64 or C68), 130.13 (C65 or C66 or C67), 128.85 (C63), 128.60(C54), 128.20 (C65 or C66 or C67), 127.79 (C50), 126.99 (C65 or C66 or C67), 125.92 (C55), 121.72 (C56), 119.32 (C61), 119.06 (C58), 118.91 (C17), 58.06 (C32), 53.66 (C5), 53.39 (C10 or C32), 51.65 (C45), 50.42 (C27), 44.90 (C1), 43.18 (C11), 37.34 (C22), 37.12 (C6), 36.20 (C59), 34.17 (C40), 32.73 (C62), 29.47 (C25 or C31), 28.85 (C25 or C31), 27.29 (C49) , 24.64 (C11), 24.11(C21), 23.33 (C39), 23.07 (C41 or C42), 21.94 (C41 or C42), 21.36 (C23 or C24), 15.60 (C23 or C24).

HRMS (ESI) calc. for $C_{52}H_{67}N_9O_{10}$ calc. for $[M+H]^+$: 978.5081m/z, found: 978.5071 m/z.

Purification: Purified through semi-preparative HPLC using Gradient 4.

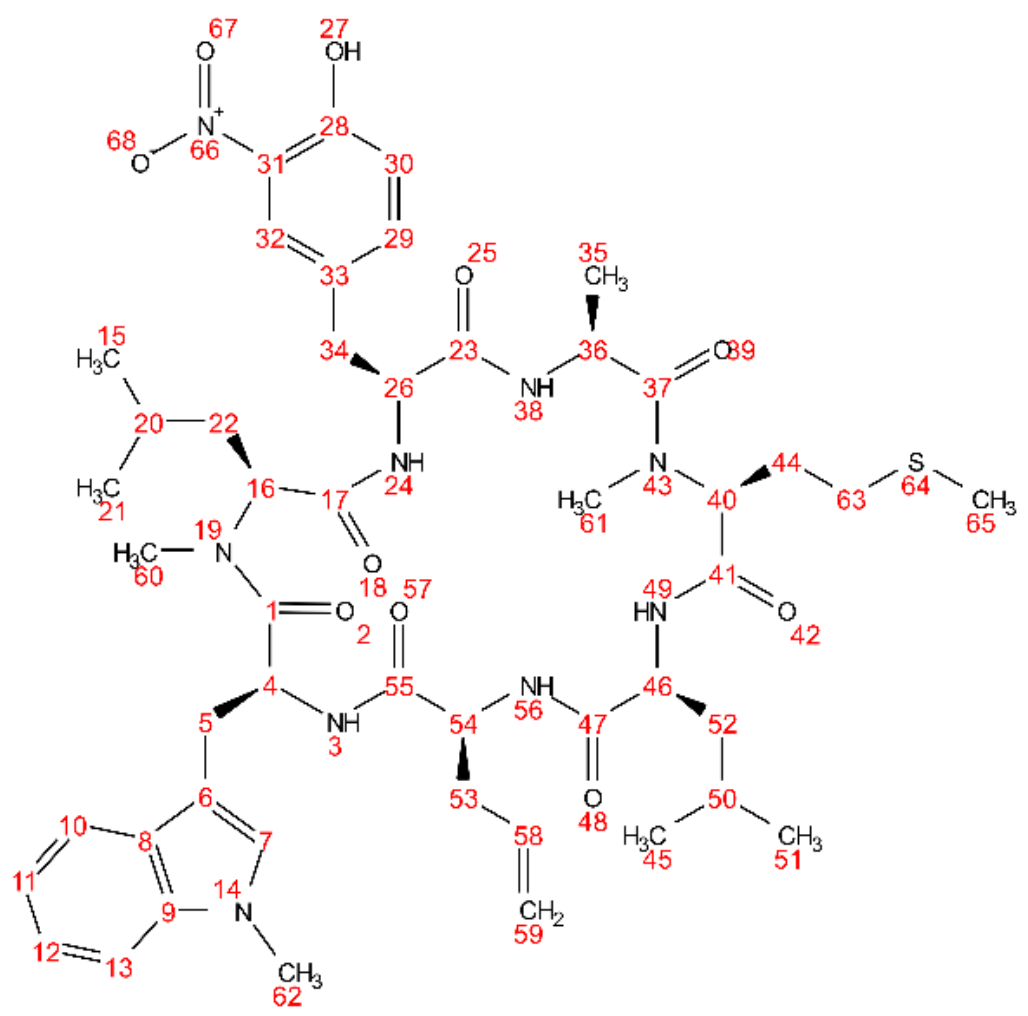

18

Crude peptide **18-linear** (99.2 mg, 99 µmol, 1 equiv.) was dissolved in 10 mL cyclisation buffer and sodium nitrite stock solution (900 uL) was added at -15 °C under nitrogen. The solution was stirred for 15 minutes at -15 °C. The mixture was gently neutralised using saturated sodium bicarbonate (1200 µL) to pH 7.5. 15 mL of ethyl acetate was added, and the mixture stirred vigorously for 60 minutes at room temperature. The mixture was further extracted with ethyl acetate (3 x 15 mL), and the combined organic layers were concentrated *in vacuo*, before being dissolved in 50% acetonitrile in water and purified by semi-preparative HPLC using Gradient 5. Purified peptide was lyophilised and isolated as a yellow powder. (Isolated yield 47.6 %).

^{1}H NMR ($(CD_3)_2SO$, 700 MHz) δ 9.29 (d, 1H, NH-N24), 9.09 (d, 1H, NH-N38), 8.42 (d, 1H, NH-N49), 8.40 (d, 1H, NH-N56), 7.54 (d, 1H, CH-C32), 7.49 (d, 1H, CH-C12 or C13), 7.40 – 7.35 (m, 1H, CH-C12 or C13), 7.19 (dd, 1H, CH-C10), 7.05 (s, 1H, CH-C7), 7.01 – 6.95 (m, 3H, CH-C11 & C29 & C30), 5.47 (ddd, 1H, CH-C58), 4.98 – 4.94 (m, 2H, CH2-C59), 4.87 – 4.85 (m, 1H, CH-C4), 4.83 – 4.80 (m, 1H, CH-C26), 4.69 (dd, 1H, CH-C36), 4.57 – 4.44 (m, 2H, CH-C16 & C40), 4.45 (ddd, 1H, CH- C54), 4.27 – 4.24 (m, 1H, CH-C46), 3.71 (s, 3H, CH3-C62), 3.26 (dd, 1H, CH-C34), 3.08 – 3.03 (m, 2H, CH2-C5), 2.92 (dd, 1H, CH-C34), 2.61 (s, 3H, CH3- C60 or C61), 2.59 – 2.54 (m, 2H, CH2-C53), 2.36 – 2.32 (m, 2H, CH2-C44), 2.42 (s, 3H, CH3-C60 or C61), 2.04 (s, 3H, CH3-C65), 1.89 – 1.83 (m, 2H, CH2 – C63), 1.57 – 1.53 (m, 2H, CH2- C52), 1.52 – 1.47 (m, 2H, CH2-C22), 1.14 (d, 3H, CH3- C35), 1.04 – 0.97 (m, 1H, CH-C20), 0.85 – 0.82 (m, 6H, CH3 – C45 & C51), 0.44 (d, 3H, CH3 – C15 or C21) 0.27 (d, 3H, CH3- C15 or C21), -0.22 (ddd, 1H, CH-C50).

^{13}C NMR ($(CD_3)_2SO$, 151 MHz) δ 171.77 (C23), 171.58 (C55), 172.22 (C47), 171.08 (C41), 169.26 (C1), 167.76 (C17), 151.43 (C8), 136.91 (C9), 136.63 (C10), 132.89 (C58), 128. 59 (C33), 128.17 (C7), 127.79 (C28), 126.04 (C6), 119.45 (C29 or C30 or C11), 119.21 (C29 or C30 or C11), 119.04 (C32), 118.93 (C29 or C30 or C11), 110.08 (C12 or C13), 109.04 (C31), 58.74 (C4), 58.03 (C16), 53.73 (C40), 53.40 (C46), 51.66 (C54), 50.36 (C26), 45.48 (C36), 42.53 (C52), 37.39 (C50), 36.99 (C34), 36.20 (C53), 32.72 (C62), 30.03 (C63), 28.94 (C60 or C61), 28.89 (C60 or C61), 28.36 (C44), 27.32 (C5), 23.33 (C20), 23.16 (C51 or C45), 21.84 (C51 or C45), 21.35 (C15 or C21), 17.39 (C15 or C21), 15.01 (C65).

HRMS (ESI) $C_{48}H_{68}N_9O_{10}S$ calc. for $[M+H]^+$: 962. 4804m/z, found: 962.4795 m/z.

Purification: Purified through semi-preparative HPLC using Gradient 4.

Chromatography Methods for Peptide Purification and Analysis

Typical gradients for the following chromatography methods involve using mixture of two solvents are described below.

- Solvent A: H_2O containing 0.1% TFA
- Solvent B: MeCN containing 0.1% TFA.
- Solvent C: 70% MeCN in H_2O containing 0.1% TFA
- Solvent D: 30% MeCN in H2O containing 0.1% TFA

- Solvent E: H_2O containing 0.1% FA
- Solvent F: MeCN containing 0.1% FA

1.5. LC-MS chromatography

The LC-MS used was an Advion expression compact mass spectrometer with Aligent 1260 Infinity HPLC and Advion Expression CMS mass spectrometer with a reverse phase Agilent Zorbax C8 column (150 x 4.6 mm, 5 µm particle size) at a constant flow rate of 0.5 mL /min at 40 °C. The UV-vis absorbance of the eluent was monitored at 220, 280 and 355 nm.

Gradient 1: 1 min with 5% E, linear gradient from 5–95% F over 38 mins, then wash 2 min with 95% F, then back to 5% E over 2 min.

1.6. Analytical HPLC

Analytical high-performance liquid chromatography (HPLC) was performed on an Agilent 1260 Infinity II instrument equipped with a DAD detector with an Agilent Eclipse XDB (C8 4.6 x 150 mm) column at a constant flow rate of 1 mL /min at 40 °C. The UV-vis absorbance of the eluent was monitored at 220 nm, 280 nm and 355 nm.

Gradient 2: 1 min with 5% B, linear gradient from 5–95% B over 26 mins, then wash 2 min with 95% B, then back to 5% B over 2 min.

1.7. Semi-preparative HPLC

In semi-preparative mode, an Agilent Eclipse XDB-C8 5 µm 9.4 x 250 mm column was used at a constant flow rate of 3.5 mL/min at 60 °C. The UV-vis absorbance of the eluent was monitored at 220, 280 and 355 nm.

Gradient 3: linear gradient 30% C in D to 100% over 43 min, then wash 1 min with 100% C, then back to 30% D in C over 2 min. (C18 9.4 x 250 mm)

Gradient 4: 1 min with 5% B, linear gradient from 5–95% B over 26 mins, then wash 2 min with 95% B, then back to 5% B over 2 min.

Gradient 5: 1 min with 50% B, linear gradient from 50–80% B over 17 mins, then 80–95% for 1 min, then 95% for 3 min, then 95–50% for 1 min, then 3 min of 50% B.

1.8. UPLC-HRMS

The peptides were analysed on a Waters ACQUITY UPLC BEH C18, 1.7 µm, 2.1 x 50 mm column with constant flow of 0.4 mL/min 40 °C. High-resolution mass spectra were recorded on a Waters Acquity UPLC-Class I equipped with an ACQUITY UPLC column. The detector was a Waters Xevo-G2-XS QTof with electrospray ionization source. The instrument was operated in positive mode full scan with detection window set from 50 to 2000 Da. For peptide fragmentation, a collision energy ramp from 15 V to 40 V was employed.

Gradient 6: 0.5 min with 5% solvent F in solvent E, linear gradient from 5–95% solvent F over 4 min, wash 1 min with 95% solvent F, then back to 5% over 0.5 min and then 0.5 min with 5% solvent F.

2. Production and Characterisation of RufM and Mutants

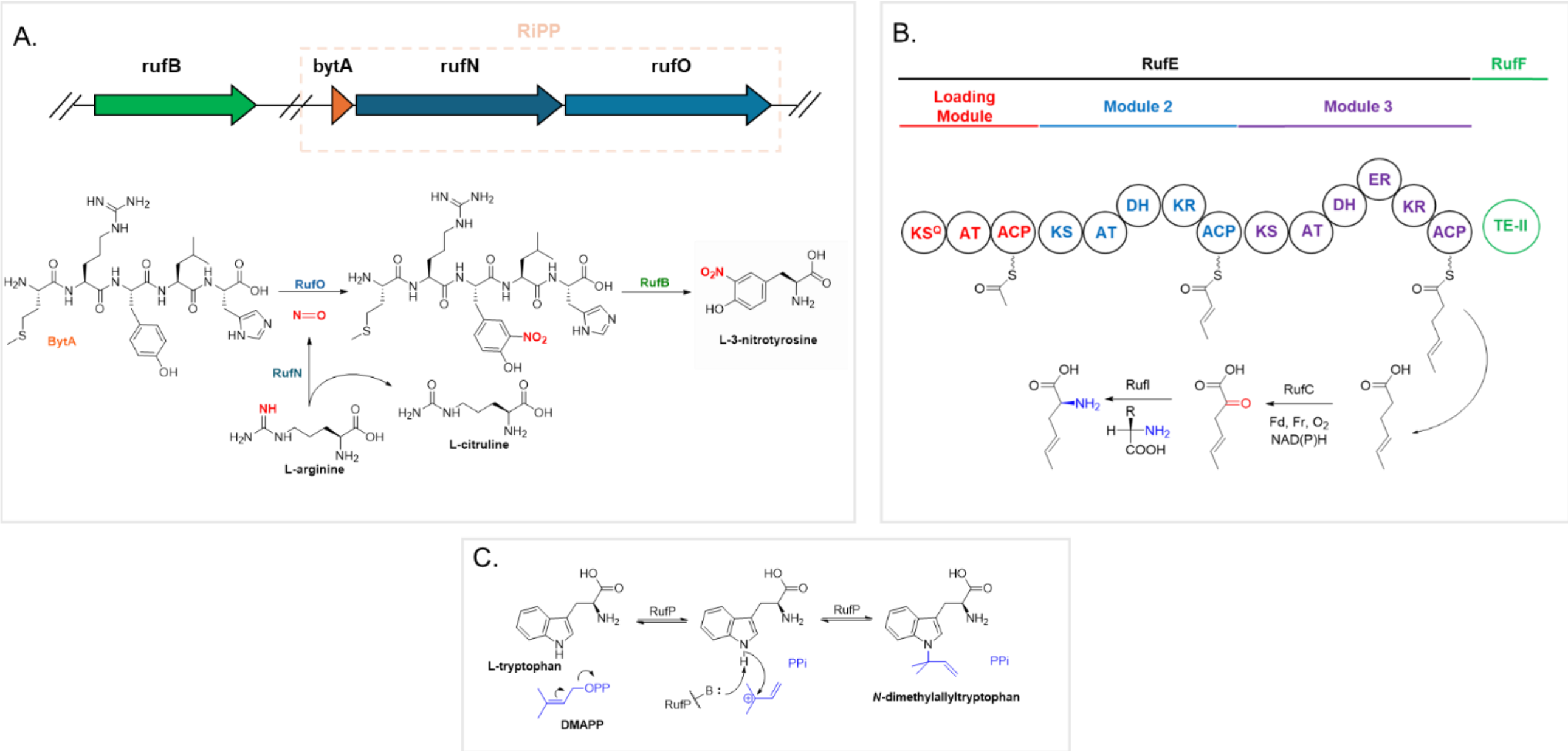

Figure S4 Biosynthesis of rufomycin B on the nonribosomal peptide synthetase, RufT. Inset: The C terminal TE domain cyclises the peptide.

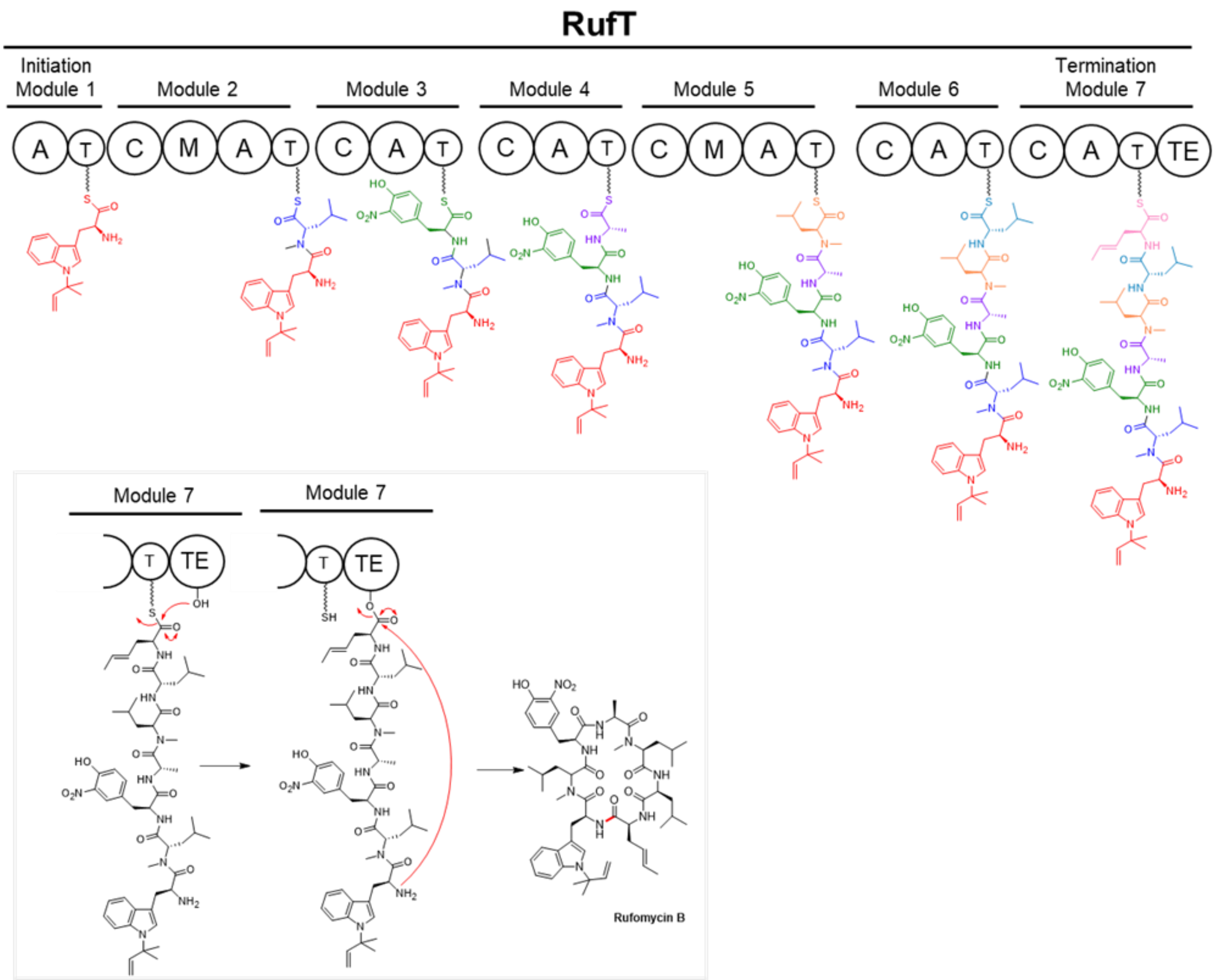

Figure S5: Biosynthesis of rufomycin B on the nonribosomal peptide synthetase, RufT. Inset: The C terminal TE domain cyclises the peptide.

```
P450EryF       --------------------------TTVPDLESDSFHVDWYRTYAELRETAPVTPVRF     33
P450RufM       ----------------VLNSENARIDIPEIAMIDPEVLHNPISAYGQARERSPIVRLMA     43
P450SaAcM      ---GHMSHASHDTGSTTVAGAACPAA---GPHMMDPDLLADPFTGYGRLRERGPVVRGRF    54
P450CymS       ----------------MYGDTEESAGVHSANNVTPLISTDPHAAYEHLREQAPACPIRS     43
P450cam        TTETIQSNANLAPLPPHVPEHLVFDFDMYNPSNLSAGVQEAW--AVLQESNVPDLVWTRC    58
P450cin        -----------------MTSLFTTADHYHTPLGPDGTPHAFFEALRDEAETTPIGWSEA     42
                                                                 :

P450EryF       LG-QDAWLVTGYDEAKAALSDLRLSSDPKK----KYPGVEVEFPAYLGFPEDVRNYFATN    88
P450RufM       PGYGSIWAVTRHGDAKAMLGDSRLAMTADSWN-------------QRIPDDIKPYML-T     88
P450SaAcM      VDGTPVWFVTRYDDVRAVLRDPRFVNTPSPVPGEGGADPREGMMDLLNVPEPLRVYLLGS    114
P450CymS       IEGSLMWIVARHDGVRSVLGDARFANNPASVPGGKAPQLQANAMRRLGLDEELIPYLADT    103
P450cam        --NGGHWIATRGQLIREAYEDYRHFSSECPF----------------IPREAGEAYDFI     99
P450cin        --YGGHWVVAGYKEIQAVIQNTKAFSNKGVT----------------FPRYETGEFELM     83
                     * .:      :    : :                         .
                        C helix
P450EryF       MGTSDPPTHTRLRKLVSQEFTVRRVEAMRPRVEQITAELLDEVG----DSGVVDIVDRFA    144
P450RufM       MSHMDGADHGRLRKLAVRAFTARRVAARRDMILKSTERLLDALPGHA-EQGTVDLLEHFA    147
P450SaAcM      ILDSDPPDHPRLRRLVTRAFAARRILGLRPGIERIADRLLAELPRREEEDGTVDLLEHFA    174
P450CymS       LLDSDGKDHNRLRRLVTPAFSARKVKALGPRIERLTDDLIDALPQHA-GGGVTDLLEHFA    162
P450cam        PTSMDPPEQRQFRALANQVVGMPVVDKLENRIQELACSLIESLR----PQGQCNFTEDYA    155
P450cin        MAGQDDPVHKKYRQLVAKPFSPEATDLFTEQLRQSTNDLIDARI----ELGEGDAATWLA    139
                   *   : : * *.   .           : . :  *:          *  :     *

P450EryF       HPLPIKVICELLGVDEKYRGEFGRWSSEILVMDPERAEQRGQAAREVVNFILDLVERRRT    204
P450RufM       RPLPTFVVGDVMGIPEADRPLWRAYVARMGS--GQT---YDDALRDAVAGAKQVVALRRE    202
P450SaAcM      YPLSITVICELVGIPATDLERWREWGGDLVSMRPER---LRHSFPVMIDYCHRLIEQRRA    231
P450CymS       YPLPINVIGELVGVPAADLPAWLRWTHDLS-FNQQA---LGQTLRPLVDYTYDLVRHRYD    218
P450cam        EPFPIRIFMLLAGLPEEDIPHLKYLTDQMTR--PDGSMTFAEAKEALYDYLIPIIEQRRQ    213
P450cin        NEIPARLTAILLGLPPEDGDTYRRWVWAITH--VENPEEGAEIFAELVAHARTLIAERRT    197
                 :   :   : *:              :     :      .           ::   *
                                   I helix
P450EryF       EPGDDLLSALIRVQDDDDGRLSADELTSIALVLLLAGFEASVSLIGIGTYLLLTHPDQLA    264
P450RufM       EPGDDLVSDLLQAQAEAADRITDTELVTMVWHTVFAGQDNLANFIANSVVALLSHPEQLA    262
P450SaAcM      ALTDDLLSELIRAQDDDGGRLSDIETVTMILTLVLAGHETSAHLIGNGTAALLTHPGQWA    291
P450CymS       EPGEDLISELIQARDADGDRLSERELVTMVLSLVIAGHDTTANFIANSIWALLTHPEQLA    278
P450cam        KPGTDAISIVANGQV-NGRPITSDEAKRMCGLLLVGGLDTVVNFLSFSMEFLAKSPEHRQ    272
P450cin        NPGNDIMSRVIMSKI-DGESLSEDDLIGFFTILLLGGIDNTARFLSSVFWRLAWDIELRR    256
                   * :* :   :      ::  :   :    :..* :  . ::.     *
                      K helix
P450EryF       LVRRDPSALPNAVEEILRYIAPPE-TTTRFAAEEVEIGGVAIPQYSTVLVANGAANRDPK    323
P450RufM       ALRADPGLMPRAVEELMRWRPPLLLTAIRYALEDFELCGAPVRRGDSVVAVIASANRDPR    322
P450SaAcM      LLRKDPALLPRAVHELMRWCGSVHVARLRYATEDLELAGTPVARGDAVQLVLVSANFDPR    351
P450CymS       LLRADPGLMPQAVQELLRWCGPVLVSRMRYATEDVDLGWGTFQTGDAVTVALSGGNHDPR    338
P450cam        ELIERPERIPAACEELLRRFSLVADG--RILTSDYEFHGVQLKKGDQILLPQMLSGLDER    330
P450cin        RLIAHPELIPNAVDELLRFYGPAMVG--RLVTQEVTVGDITMKPGQTAMLWFPIASRDRS    314
                :   *  :* * .*::*          *   .:  .    .   .        .. *
                             Heme biniding pocket
P450EryF       QFPDPHRFDVTRDTRG----HLSFGQGIHFCMGRPLAKLEGEVALRALFGRFPALSLGID    379
P450RufM       VFADPDTLDITRPAGT--AVQFGFGHGPHYCIGASLAAVEAEVALGALLNRFPDLALAVP    380
P450SaAcM      HYSDPDRLDITRHQEGQAENHVGFGHGIHYCLGATLARQEGEVALARLLETYPDLALADG    411
P450CymS       RFTDPGRLDITRQHDTRGEVHLSFGAGPHYCLGAALARHEAEIALSRMLARHPDLALAVP    398
P450cam        ENACPMHVDFSRQKVS----HTTFGHGSHLCLGQHLARREIIVTLKEWLTRIPDFSIAPG    386
P450cin        AFDSPDNIVIERTPNR----HLSLGHGIHRCLGAHLIRVEARVAITEFLKRIPEFSLDPN    370
                   *  . . *        :  :* * * *:*  *   *   :::   :   *  :::

P450EryF       ADDVVWRRSLLLRGIDHLPVRLDG------    403
P450RufM       PQEVPRLPNPGSWHLTALPVTL*-------    402
P450SaAcM      DPEVRRARLPGSWRLDALRLRLRP------    435
P450CymS       AEQLQWHAVSGMRKLATLPVRL--------    420
P450cam        A-QIQHKSG-IVSGVQALPLVWDPATTKAV    414
P450cin        K-ECEWLMG-QVAGMLHVPIIFPKGKRLSE    398
                 :           :  : :
```

Figure S6: Clustal Omega alignment of RufM with key P450s. P450Eryf (PDB: 6DEB), P450SaAcm (PDB: 5NWS), P450CymS (SARE4557; CP000850.1),[2] P450cam (PDB:

5CP4), P450cin (PDB: 4FB2). Highlighted in yellow is the catalytically important I helix consensus sequence ((A/G) Gx(E/D)T) that differs among the P450s.

2.1. *rufM* site directed mutagenesis

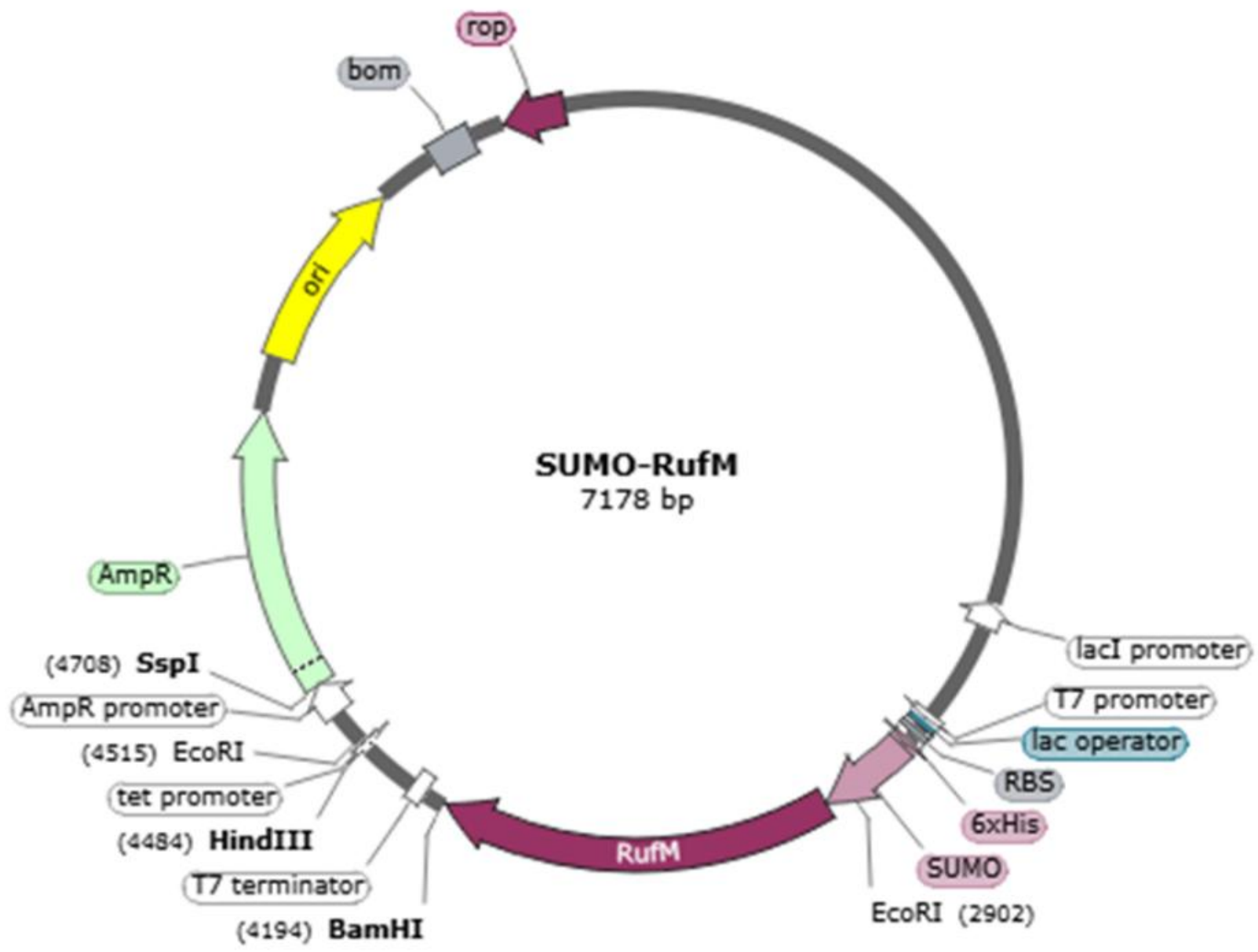

Figure S7: Plasmid map of His$_6$-SUMO-RufM . Map shows the general features of the plasmid and the location of the gene *rufM.* Features include the Small Ubiquitin-like Modifier (SUMO) solubility partner tag for ease of purification, an ampicillin resistance gene (AmpR) for selection, a 6x polyhistidine tag for protein purification with HisTrap columns, a TEV cleavage site and T7 promoter and lac repressor protein (lacI) for IPTG-induced gene expression.

All mutants were generated using the QuikChange IIXL Site-Directed Mutagenesis Kit (Agilent Technologies). Only one base pair was changed at a time, so the mutant N242T was made first for the required codon change AAC to ACC. This mutant was then used to convert ACC to GCC.

Point mutations performed on His$_6$-SUMO-RufM are shown in the table below. For the mutants N242A, N242E, N242Q and N242L multiple base pair modifications were needed from the original plasmid. In these cases, other mutant DNA was used as the template. For example, for N242A was created using the N242T mutant as template. The same applied for N242E, N242Q and N242L, where each mutant was generated using the previous mutants DNA and N242D was used for N242E.

**His$_6$-sumo-*rufM* Mutagenesis**				
Point Mutation	Nucleotide Change	Forward Primer (5'-3')	Reverse Primer (5'-3')	Amino acid

N242T	AAC→A**C**C	TCTTCGCAGGTCAGGAC**ACC**CTTGCGAACTTCATCGCC	GGCGATGAAGTTCGCAAGG**TGG**TCCTGACCTGCGAAGA	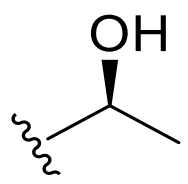
N242A	ACC→**G**CC	ACCGTCTTCGCAGGTCAGGAC**GCC**CTTGCGAACTTCATCGC	GCGATGAAGTTCGCAAGG**CGG**TCCTGACCTGCGAAGACGGT	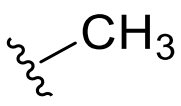
N242S	ACC→A**G**C	ACCGTCTTCGCAGGTCAGGAC**AGC**CTTGCGAACTTCATCGCC	GCGATGAAGTTCGCAAGG**CTG**TCCTGACCTGCGAAGACGGT	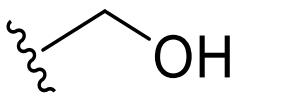
N242D	AAC→**G**AC	ACCGTCTTCGCAGGTCAGGAC**GAC**CTTGCGAACTTCATCGCC	GGCGATGAAGTTCGCAAG**GTC**GTCCTGACCTGCGAAGACGGT	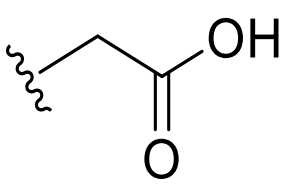
N242E	GAC→GA**G**	ACCGTCTTCGCAGGTCAGGAC**GAG**CTTGCGAACTTCATCGCC	GGCGATGAAGTTCGCAAG**CTC**GTCCTGACCTGCGAAGACGGT	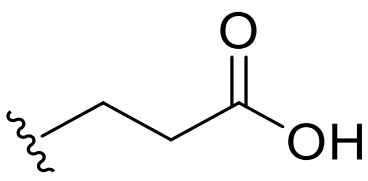
N242Q	GAG→**C**AG	ACCGTCTTCGCAGGTCAGGAC**CAG**CTTGCGAACTTCATCGCC	GGCGATGAAGTTCGCAAG**CTG**GTCCTGACCTGCGAAGACGGT	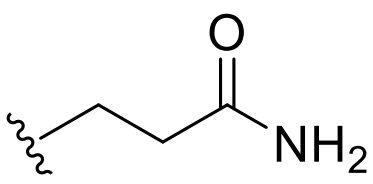
N242L	CAG→C**T**G	ACCGTCTTCGCAGGTCAGGAC**CTG**CTTGCGAACTTCATCGCC	GGCGATGAAGTTCGCAAG**CAG**GTCCTGACCTGCGAAGACGGT	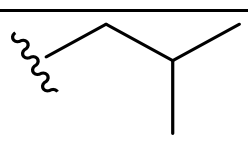

Table S1: Primers used for mutagenesis of RufM in PCR reactions. The triple codon is in bold and the nucleotide undergoing the point mutation is underlined.

Each PCR reaction was run in triplicate: one with just the forward primer, one with just the reverse primer and one with both. The reactions were ran following the instructions of the kit, each PCR reaction was prepared with the reagents provided in the kit as follows: 10× Reaction buffer (5 µL), Quick Solution (3 µL), PfuUltra HF DNA polymerase (2.5U, 1 µL) and dNTP mix (1 µL), as well as the rufM template DNA (1 µL, 10 ng/ µL), forward primer (12.5 µL, 10 ng/ µL), reverse primer (12.5 µL, 10 ng/ µL) and sterile filtered water (14 µL).

The below cycling parameters were used. For the mutants N242D, N242E, N242Q and N242L the PCR failed to work at 65°C. To improve the annealing of primers in stage 2,1 µL DMSO was added to the reaction, the PCR reactions were prepared in triplicate, and a gradient was used for the cycling parameters. Three temperatures were tested at 65°C, 70°C and 75°C.

Stage	Cycles	Temperature			Time
1	1	95 °C			1 minute
2	18	95 °C			50 seconds
		65 °C	70 °C	75 °C	50 seconds
		68 °C			1 minute

3	1	68 °C	7 minutes

Table S2: PCR cycle parameters for the mutagenesis of RufM.

On completion, the PCR reaction was cooled on ice to reach 37 °C, 1 µL of Dpn I was added to each reaction and then incubated at 37 °C for one hour. 2 µL of PCR product was used to transform a 45 µL aliquot of XL10 Gold Ultra Competent *E. coli* cells that had been pre-treated with 2 µL of β-mercaptoethanol. Transformed mixtures were then plated on LB agar (50 µg/ mL carbenicillin) and incubated for 16 hours at 37 °C.

A single colony of transformed cells were grown in 10 mL of LB liquid media containing 50 µg mL^{-1} of carbenicillin, incubated overnight at 37 °C with shaking (220 rpm). The DNA was extracted and purified using the protocol provided with QIAprep® Spin Minikit (QIAGEN). The DNA concentrations were determined using pre-set software in NanoDrop ND-8000, 8 Sample spectrophotometer (Labtech). The recombinant DNA was sequenced to confirm the correct mutations using Genewiz.

2.2. Production of RufM, RufS and Mutants

Plasmids were used to transform Tuner-DE3 (Sigma-Aldrich) competent cells as follows: Cells were left to thaw for 2 minutes on ice, then 1 µL of plasmid (10 ng/ uL) was added directly. The cells were heat shocked at 42 °C by a water bath for 30 seconds, then left for 30 minutes on ice. 500 µL of SOC media preheated to 42 °C was added to the cells. They were then incubated at 37 °C with shaking (225 rpm) for 1 hour. Transformed mixtures were then plated on LB plates (50 µg/ mL carbenicillin) and incubated for 16 hours at 37 °C.

Single colonies of the previously transformed Tuner cells were grown in 10 mL of LB liquid media (50 µg mL^{-1} of carbenicillin) and then left to incubated overnight at 37 °C with shaking (220 rpm). 500 mL of LB liquid media (50 µg mL^{-1} of carbenicillin) were inoculated with 3 mL of overnight culture and incubated at 37 °C with shaking (220 rpm). Once the OD_{600} reached between 0.8-1.0, the cultures were cooled and induced with isopropyl-β-D-thiogalactopyranoside (IPTG, final concentration: 1 mM) and supplements of iron (II) sulfate heptahydrate (final concentration: 1 mM) and 5-aminolevulinic acid (final concentration: 5 mM). The cultures were incubated overnight at 15 °C with shaking (220 rpm).

The cultures were then centrifuged (4200 rpm, 20 minutes, 4 °C), the supernatant was discarded. The pellet was resuspended in a minimal amount of Buffer A (Tris 20 mM, pH 8 Imidazole 50 mM, NaCl 100 mM, 10% Glycerol) dithiothreitol (DTT) 0.5 mM), pepstatin (50 µL per 1.5 L culture), DNAse (0.2 mg mL^{-1}) and 1 Complete™ protease inhibitor cocktail tablet (Sigma) per 2L of culture. Cell lysis was carried out on the cell disruptor IXT4A (Constant System LTD) with a pressure of 25 kPa. The lysate was then centrifuged (18000 rpm, 42 minutes, 4 °C). Protein purification using AKTA Pure Chromatography System (GE) was then carried out on the supernatant.

All buffers used on the AKTA were filtered and degassed. A 5 mL His-TrapTM Fast Flow nickel affinity column (GE) was washed with water (5 column volumes) and then Buffer A (5 column volumes) before the supernatant was loaded onto the column. The column was then washed with Buffer A until the 280 absorbance returns to baseline. The protein was then eluted with Buffer B (Tris 20 mM, pH 8, Imidazole 200 mM, NaCl 100 mM, 10% Glycerol) and the fractions containing the protein were combined and concentrated. The protein was concentrated to 500 µL and then buffer exchanged with Buffer C (Tris 20 mM, pH 8 NaCl 100 mM, 10% Glycerol) thrice by concentrating to 500 µL and diluting the protein in 20 mL Buffer C. The protein was

finally concentrated to 250 µM before being mixed with an equal volume of Buffer D (Tris 20 mM, NaCl 100 mM, 30% Glycerol). The protein solution was concentrated again to a concentration of 250 µL and then divided into 20 µL aliquots, flash frozen and stored at -80 °C.

Where stated the His_6-SUMO-RufM was additionally purified by size-exclusion chromatography following nickel affinity column chromatography. Fractions containing the protein were combined and concentrated to 5 mL and injected onto a HiLoadTM 16/600 Superdex 200pg size exclusion chromatography column (GE) equilibrated with Buffer C (20 mM Tris buffer, pHx?, 100 mM NaCl, 10% Glycerol), Proteins were eluted with buffer C and fractions containing the protein were concentrated, mixed with an equal volume of Buffer D (20 mM Tris buffer, pHx? 100 mM NaCl, 30% glycerol) and divided into 50 µL aliquots, flash frozen and stored at -80°C.

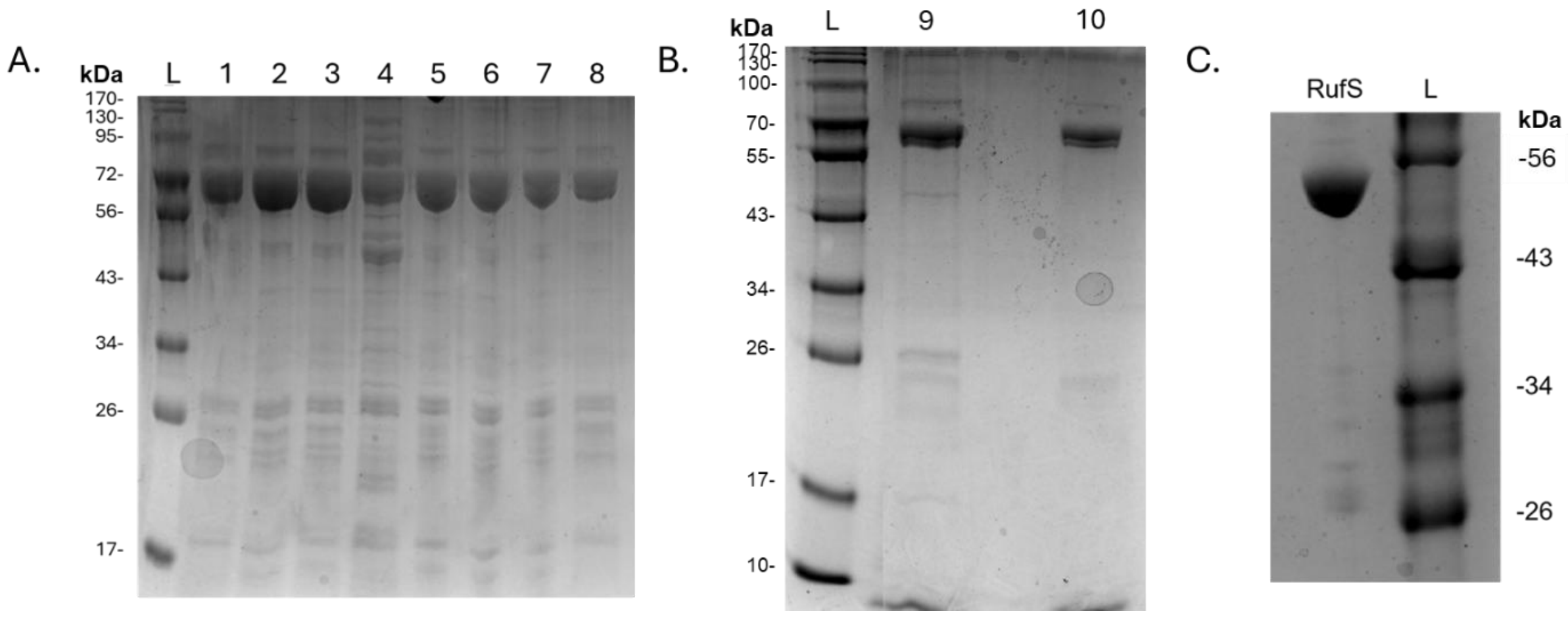

Figure S8: SDS-gel of purified CYP proteins A. His_6-SUMO-RufM (56.1 kDa), L. Protein Ladder, 1. WT N242, 2. N242T, 3. N242A, 4. N242S 5. N242D, 6. N242E, 7. N242Q, 8. N242L, **B.** RufM purification steps, 9. Purified RufM by HisTrap, 10. Purified RufM by HisTrap then SEC, **C.** His_6-RufS (47.9 kDa). Note: activity of RufM lane 1 A and lane 9 B is comparable.

Production of ULP-1

Rosetta BL21 (DE3) cells transformed with the ULP-1 C48/pET30 plasmid were plated from a glycerol stock on LB plates with 50 µg mL^{-1} kanamycin and incubated at 37 ˚C overnight. Single colonies were selected and inoculated in 10 mL non-inducing minimal medium (MDG, 0.5% glucose, 0.25 % aspartate, 2 mM $MgSO_4$, 25 mM Na_2HPO_4, 25 mM KH_2PO_4, 50 mM NH_4Cl, 5 mM Na_2SO_4 with 40 µg mL^{-1} kanamycin and incubated for 16 hours at 37 °C shaking (220 rpm). 1 mL of starter culture was diluted in 1 L of auto induction media (TYM-5052)[3] with 40 µg mL^{-1} kanamycin and incubated for 16 hours at 37 °C shaking (220 rpm). When the OD_{600} reached 2.0, the cells were centrifuged (15 minutes, 4 °C, 4200 rpm). The pellets were weighed and for each gram of pellet 5 mL lysis buffer (Tris 50 mM, NaCl 150 mM, EDTA 0.1 mM, DTT 5 mM and 5% glycerol, pH 7.9) with 1% Triton X-100 was added. The pellet was then resuspended. The cells were lysed by passing them through a cell disruptor (Constant System) at a 25 kPa pressure twice. The lysate was centrifuged (15 minutes, 4 °C, 18000 rpm). The insoluble material was washed with 5 mL lysis buffer with 1% Triton X-100 per gram of original pellet and the pellet was resuspended. The cells were centrifuged (15 minutes, 4 °C, 18000 rpm), and the pellet was resuspended in lysis buffer. The cells were centrifuged (15

minutes, 4 °C, 18000 rpm) and the pellet was dissolved in 2.5 mL binding buffer (Tris 50 mM, NaCl 150 mM, guanidine chloride 6M, imidazole 5 mM and 5% glycerol, pH 7.9) per gram of pellet. The sample was rocked at room temperature for 60 minutes, then centrifuged (15 minutes, 4 °C, 18000 rpm).

The protein was purified using the AKTA with chromatograms monitored at 280 nm. A 5 mL His-TrapTM Fast Flow nickel affinity column (GE) was washed with 5 column volumes water and 5 column volumes binding buffer. The supernatant was then loaded onto the column and left overnight. The column was washed with 6 column volumes binding buffer and 3 column volumes binding buffer with 10 mM imidazole. The column was then washed with 3 column volumes of urea buffer (Tris 50 mM, NaCl 150 mM and Urea 6 M, pH 8.2). The mobile phase was changed to the elution buffer (Tris 38 mM, NaCl 120 mM, urea 4.5 mM and imidazole 500 mM, pH 7.9) and the eluent was collected in 1.5 mL fractions. The fractions containing protein were concentrated by centrifugation to a concentration of 0.4 mg mL^{-1}. The concentrated protein was diluted in refolding buffer (L-arginine 1M and 25% glycerol, pH 7.9) as a 1 in 50 dilution. The solution was mixed and incubated on ice for one hour to allow refolding. The solution was transferred to 3.5 MWCO dialysis tubing and dialysed against 2L of dialysis buffer (Tris 50 mM, NaCl 150 mM and DTT 1 mM, pH 8.2) overnight at 4 °C. The solution was dialysed against 2 L of fresh buffer for 3 hours at 4 °C. The protein was then concentrated to 0.4 mg mL^{-1}, flash frozen, and stored at -80 °C.

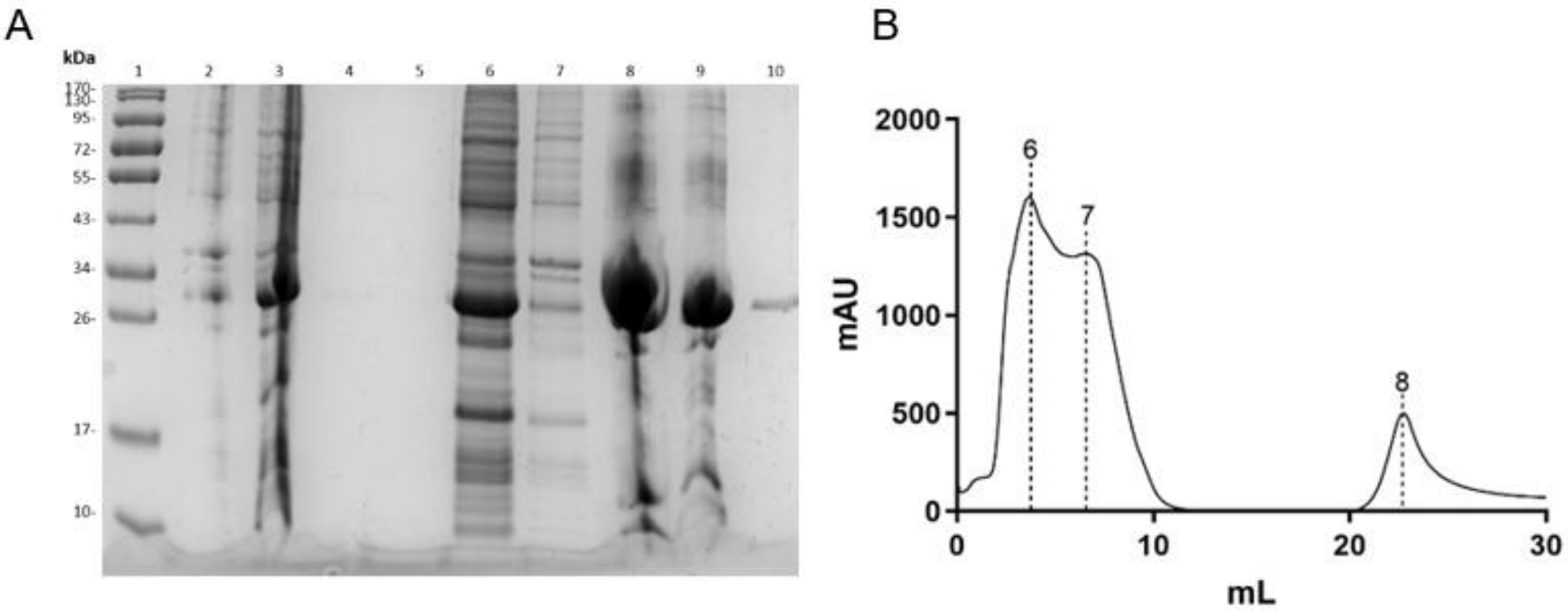

Figure S9: Analysis of ULP-1 production A) SDS-gel of ULP-1 protein expression and purification: 1. Protein ladder, 2. Pellet after final wash, 3. Lysate after final wash, 4. HisTrap binding buffer wash, 5. HisTrap binding buffer with imidazole wash, 6. peak 6 in urea buffer wash, 7. peak 7 in urea buffer wash, 8. peak 8 in elution buffer, 9. peak 8 after concentration, 10. final protein

2.3. Cleavage of His_6-SUMO-RufM

His_6-SUMO-RufM was expressed as described (Section 2.2), it was purified by His-Trap Fast Flow nickel affinity column (GE). Fractions were collected and then concentrated to 250 µL. The protein was diluted in cleavage buffer (Tris 25 mM, NaCl 150 mM, DTT 1 mM, pH 8) to a concentration of 50 µM. ULP-1 was added to a final concentration of 10 µM, to achieve a 5:1 ratio of His_6-SUMO-RufM: ULP-1 respectively at a 10 mL total volume. The solution was transferred to 3.5 MWCO dialysis tubing and dialysed against 2 L buffer C at 4 °C for 18 hours. RufM was diluted to 20 mL using buffer C before again loading on to a 5 mL His-TrapTM Fast Flow nickel affinity column (GE). The protein was eluted in the Buffer A in 1.5 mL factions. The

fractions containing the protein were concentrated to 250 μM and mixed with an equal volume of Buffer D. The protein solution was then divided into 50 μL aliquots, to be flash frozen and kept at -80 °C.

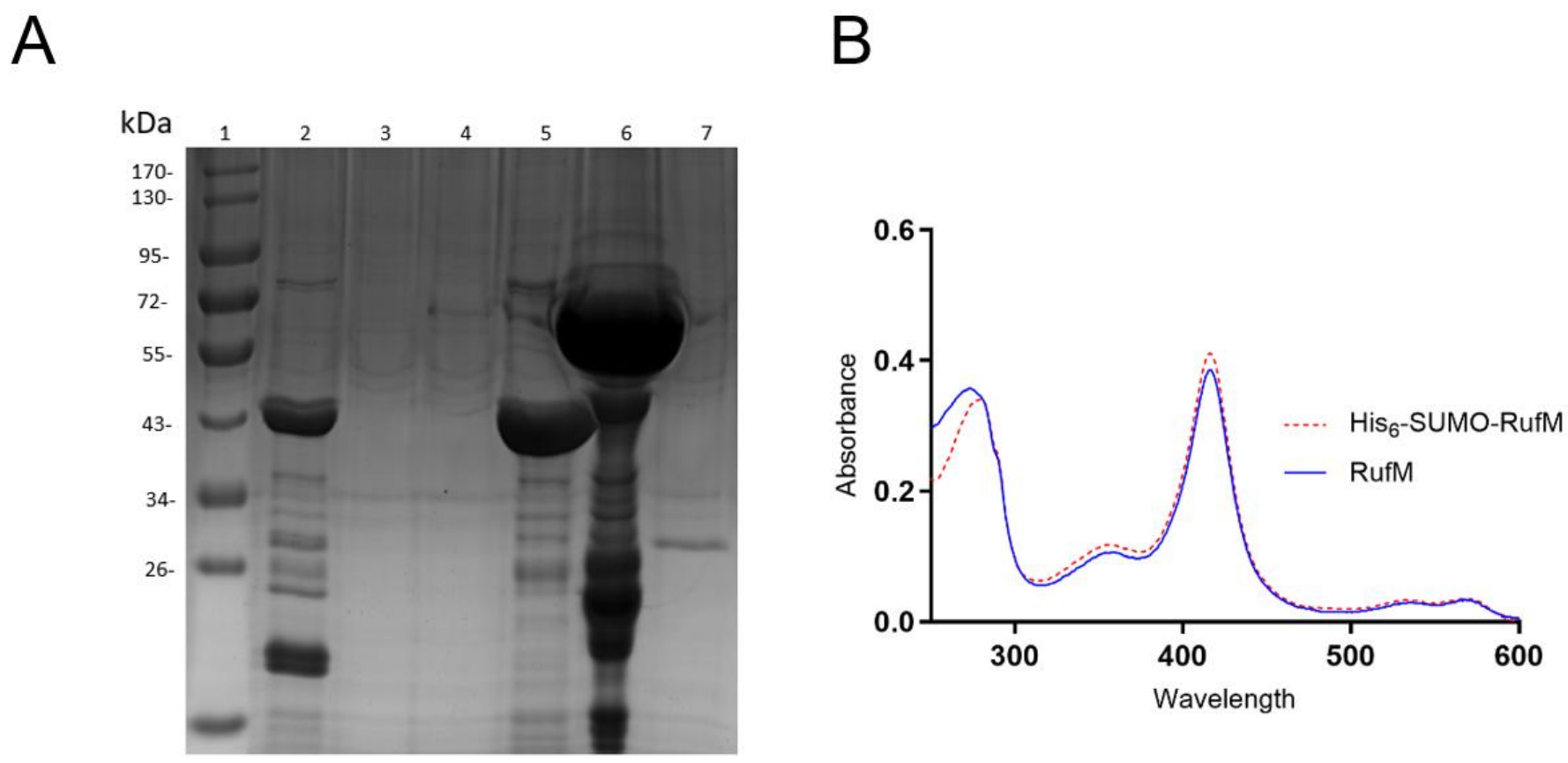

Figure S10: Analysis of His$_6$-SUMO-RufM cleavage. A) SDS-PAGE analysis of His$_6$-SUMO-rufM cleavage: 1. Protein Ladder, 2. ULP-1 SUMO-cleavage reaction after 18 hours at 4°C, 3. Buffer A wash, 4. Buffer B flowthrough, 5. RufM after nickel affinity chromatography post cleavage, 6. His$_6$-SUMO-RufM after nickel affinity chromatography prior to cleavage, 7. ULP-1. B) UV-Visible spectra of His$_6$-SUMO-RufM shown in the red hashed line and RufM is shown in the blue solid line.

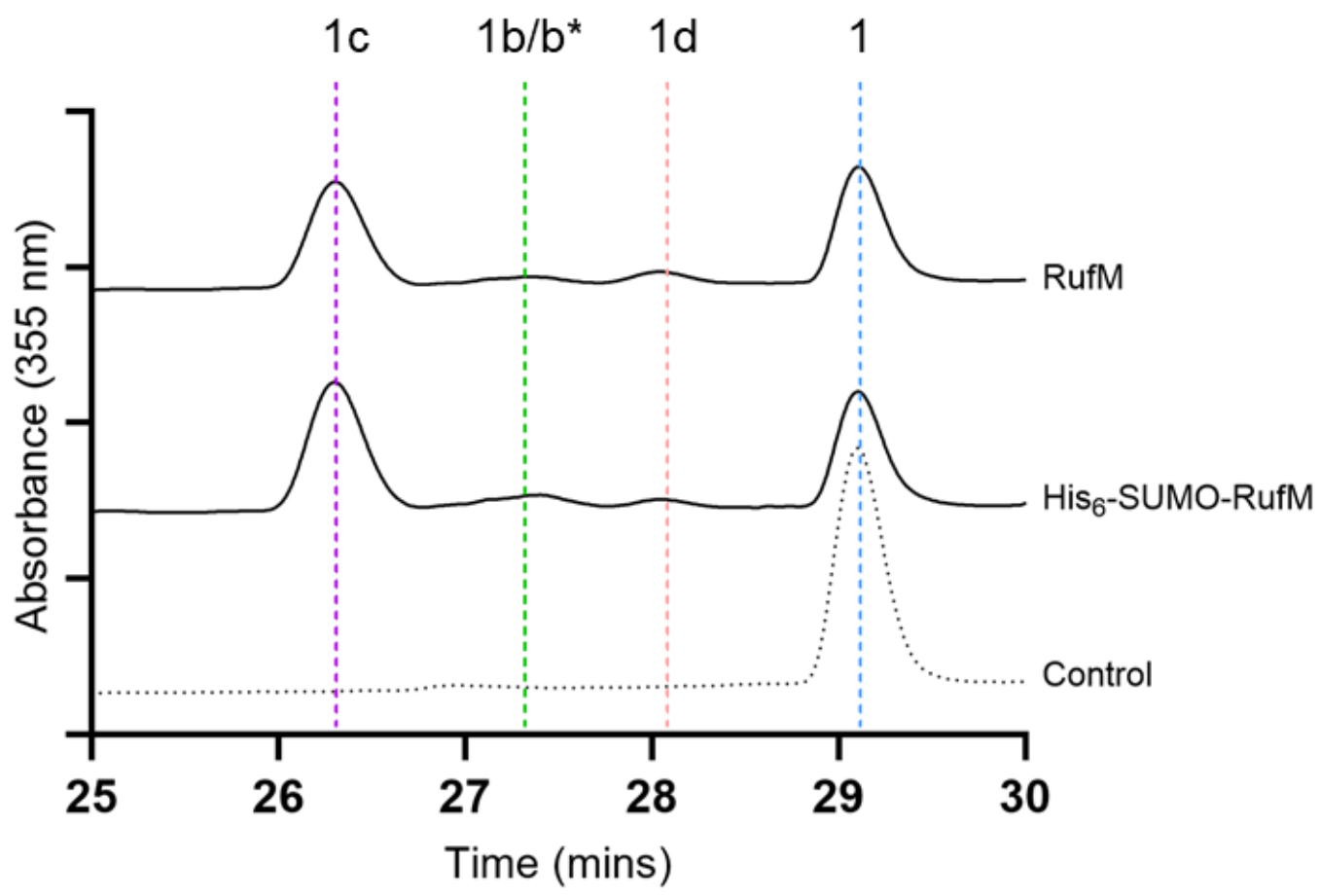

Figure S11: LC-MS analysis showing conversion of Peptide 1 by His$_6$-SUMO-RufM and RufM. Traced recorded at 355 nm absorbance. Dotted line shows the peptide 1 standard, lower line is His$_6$-SUMO-RufM, and the higher line is RufM. The assay was carried out for 3 hours at 30 °C thrice according to section 2.7.

2.4. UV-Visible Analysis of RufM and mutants

UV-Vis spectra was recorded for the RufM and the mutants N242Q, N242T, N242S, N242A, N242L, N242E and N242D using a Shimadzu UV-Vis Spectrophotometer UV-2700i. Spectra of solutions of 5 μM protein were made in 25 mM Tris buffer (pH 8) were measured in a ultra Micro Hellma® absorption cuvette.

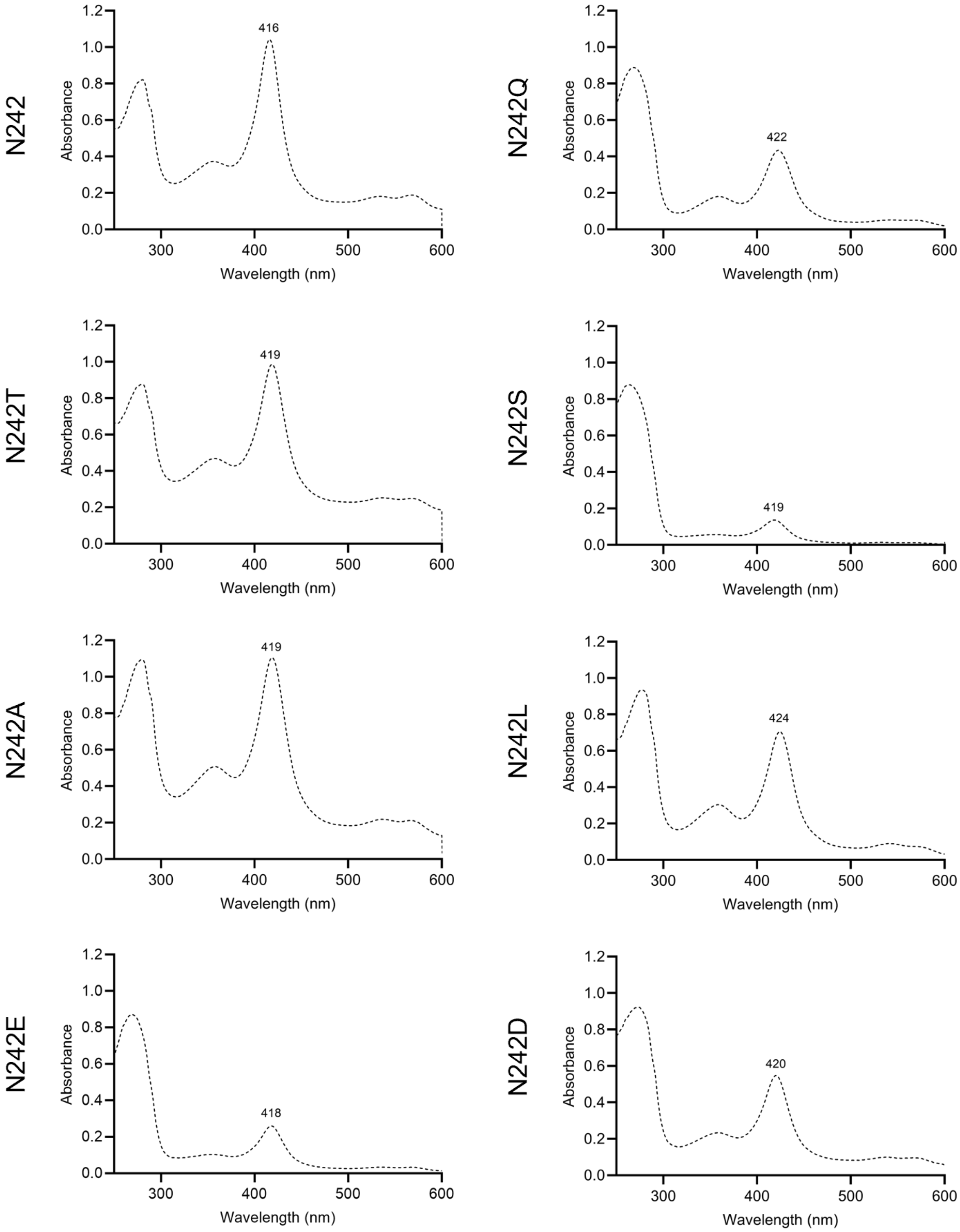

Figure S12: UV-Visible spectra of the P450 RufM and its mutants. Spectra recorded from 200 – 600 nm for N242, N242Q, N242T, N242S, N242A, N242L, N242E and N242D.

2.5. Heme determination of RufM and Mutants

The concentration of Heme for P450 enzymes was determined from the UV-Vis spectra with the absorbance of the Soret Band and was calculated using Beer-Lambert's Law with the ε = 176.5 cm^{-1} mM^{-1} as related to the low spin Fe^{III} in the heme. [4]

Heme incorporation was estimated for the P450 enzymes using the absorbance of the protein peak (280 nm) and the Soret band (~420 nm) (A_{420}/A_{280}). [5]

P450 RufM	Heme incorporation (A_{420}/A_{280})	Soret band λ_{max} (nm)
N242	1.27	416
N242Q	0.55	422
N242T	1.12	419
N242S	0.19	419
N242A	1.01	419
N242L	0.76	424
N242E	0.33	418
N242D	0.62	420

Table S3: P450 RufM Heme incorporation estimation and Soret shifts. Heme incorporation reported as ratio between the absorbance max for the Soret band and the protein peak at 280 nm (A_{420}/A_{280}). Soret band wavelength at its absorbance max (λ_{max}) determined using UV-Visible spectroscopy.

2.6. RufM-Peptide Binding Studies

2.6.1 Method 1: K_d calculation

The capability of the P450's to bind to peptides was studied using peptides **9**, **10**, and **11** The spectra were measured the same way as above using a Shimadzu UV-Vis Spectrophotometer UV-2700i. Spectra of 5 µM protein solutions were made in 25 mM Tris buffer (pH 8) and measured in a ultra Micro Hellma® absorption cuvette. Peptide stocks (5 mM) dissolved in DMSO were added at increasing concentrations (1 µM – 12 µM) and gently mixed in the cuvette before scanning. Reactions were performed in triplicate.

The apparent K_d value was then determined using the following equation:

$$\Delta A = \frac{\Delta A_{max}}{2P}\left[P + S + K_D - \sqrt{(P + S + K_D)^2 - 4PS}\right]$$

Equation S1:

Where P is the total enzyme concentration and S is the total substrate concentration. [6,7]

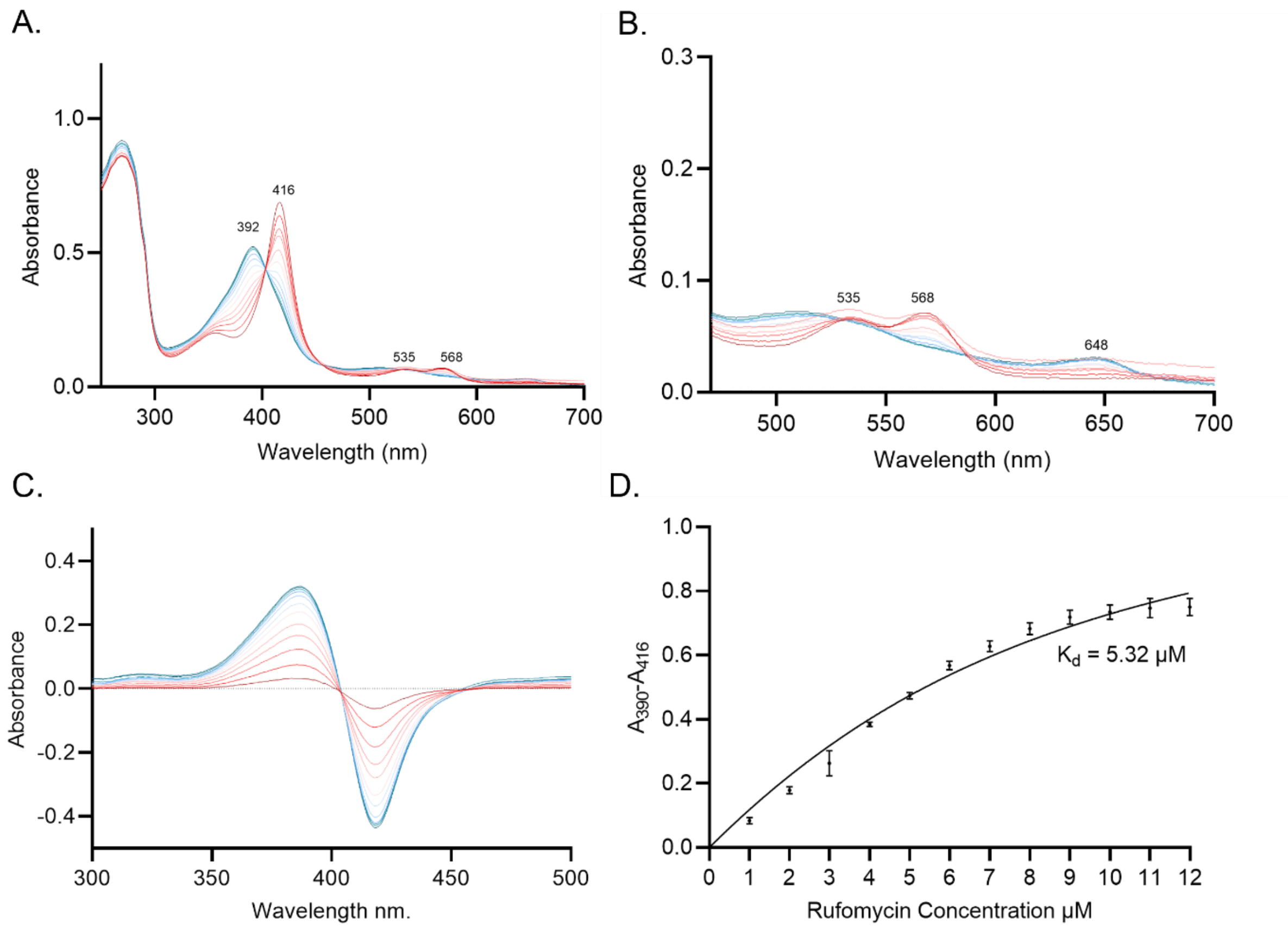

Figure S13: UV-Visble spectra studies of N242 RufM with increasing concentrations of 10 (0- 12 µM). A) UV-visible spectra 250-600 nm. Low spin iron (II) peak identified at 416 nm for the non-substrate bound protein and high spin iron (II) identified at 392 nm with 12 µM substrate bound. B) Zoom in (×3) of the UV-visible spectra 470-700 nm showing the absorbance maxima for the substrate free protein at 535 nm and 568 nm. The substrate bound protein shows a maxima at 648 nm. C) Absorbance difference between 391 and 416 nm spectra with increasing concentrations with an isosbestic point at 404 nm. D) Absorbance difference plotted versus **10** concentration used to determine a K_d value of 5.32 µM ± 1.29 µM.

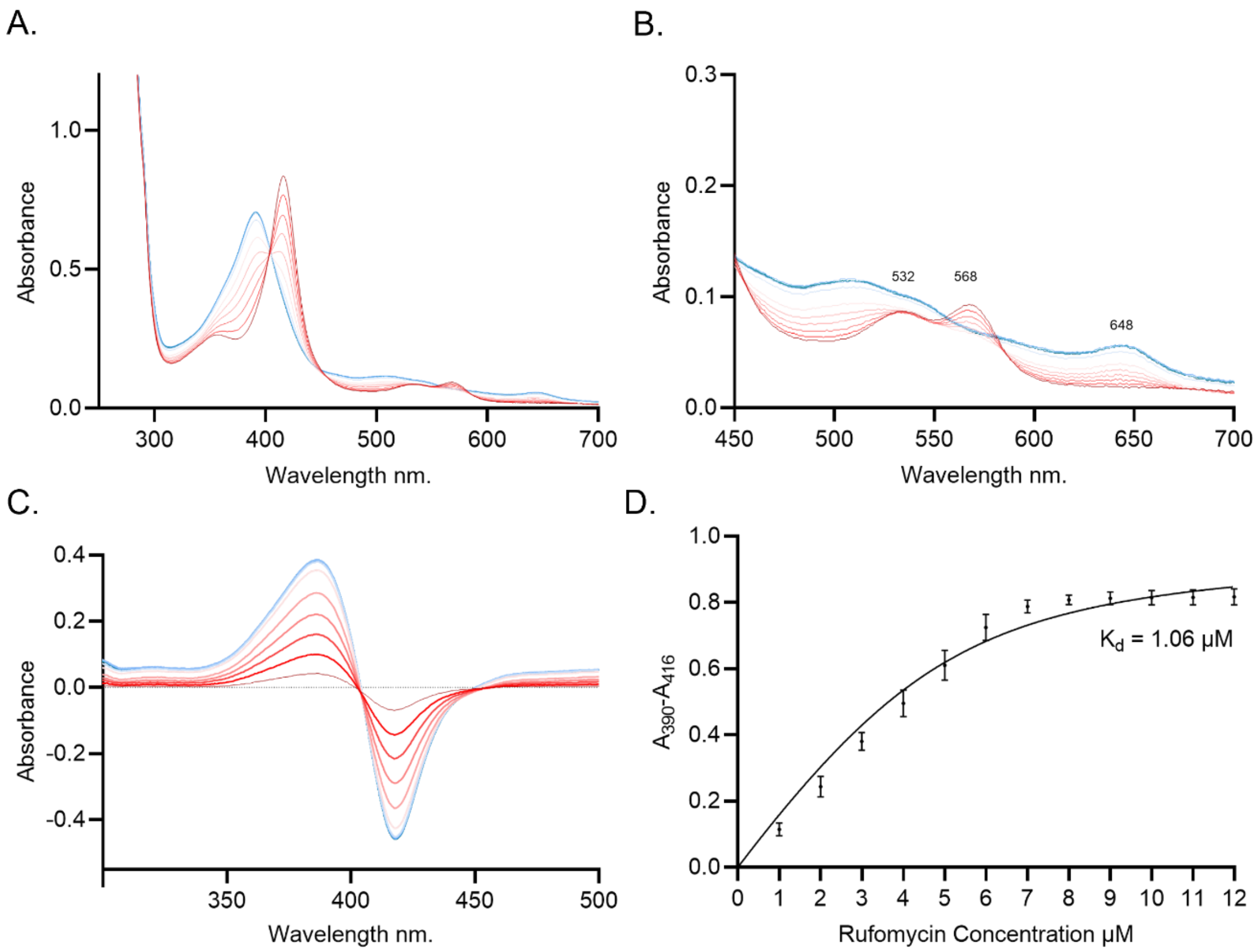

Figure S14: UV-Vis spectra studies of N242 RufM with increasing concentrations of 9 (0 - 12 μM). A) UV-visible spectra 250-600 nm. Low spin iron (II) peak identified at 416 nm for the non-substrate bound protein and high spin iron (II) identified at 392 nm with 12 μM substrate bound. B) Zoom in (×3) of the UV-visible spectra 470-700 nm showing the absorbance maxima for the substrate free protein at 535 nm and 568 nm. The substrate bound protein shows a maxima at 648 nm. C) Absorbance difference between 391 and 416 nm spectra with increasing concentrations with an isosbestic point at 404 nm. D) Absorbance difference plotted versus **9** concentration used to determine a K_d value of 1.06 μM ± 0.338 μM.

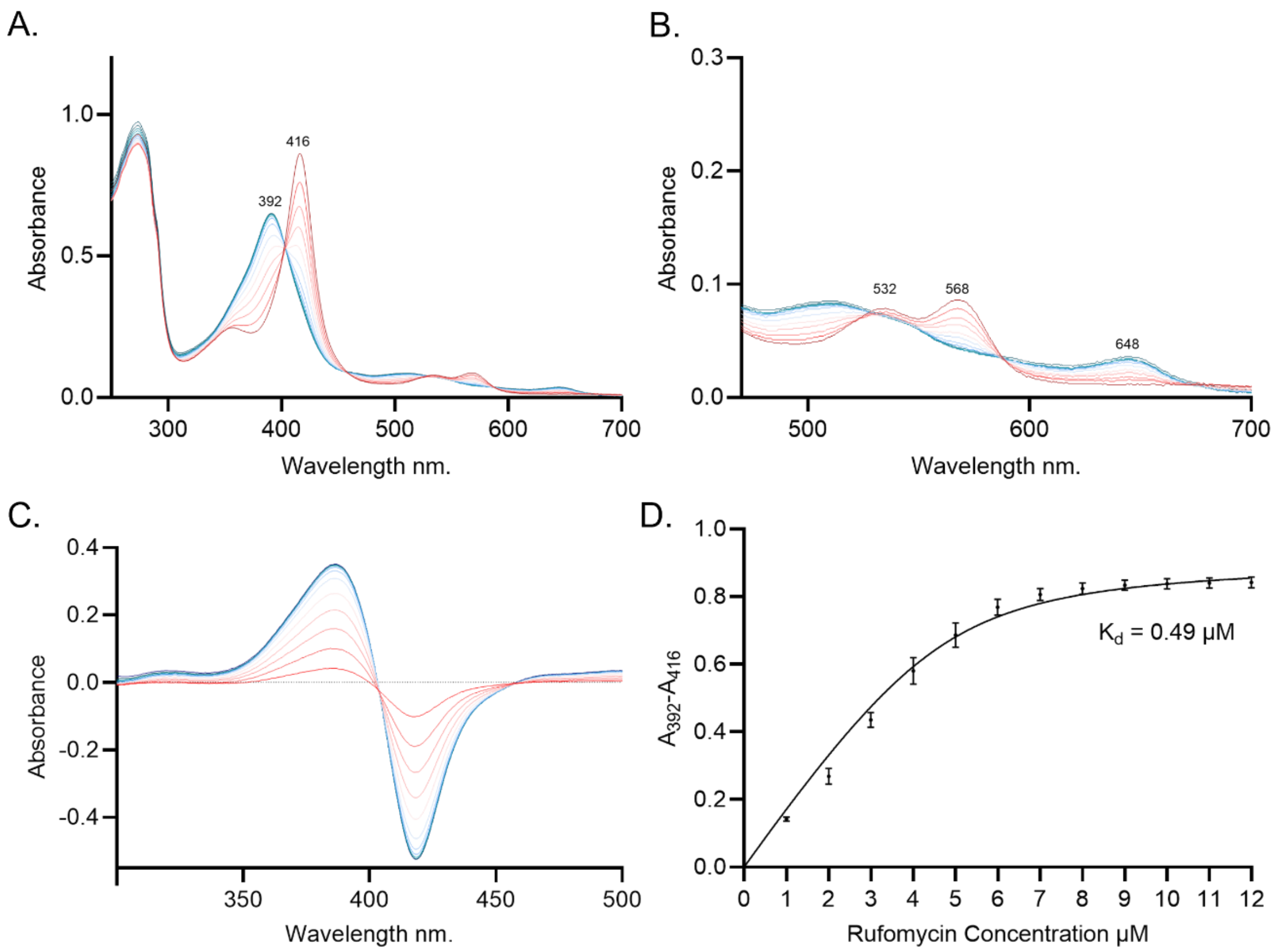

Figure S15: UV-Vis spectra studies of N242 RufM with increasing concentrations of 11 (0 - 12 μM). A) UV-visible spectra 250-600 nm. Low spin iron (II) peak identified at 416 nm for the non-substrate bound protein and high spin iron (II) identified at 392 nm with 12 μM substrate bound. B) Zoom in (×3) of the UV-visible spectra 470-700 nm showing the absorbance maxima for the substrate free protein at 535 nm and 568 nm. The substrate bound protein shows a maxima at 648 nm. C) Absorbance difference between 391 and 416 nm spectra with increasing concentrations with an isosbestic point at 404 nm. D) Absorbance difference plotted versus **11** concentration used to determine a K_d value of 0.49 μM ± 0.119 μM.

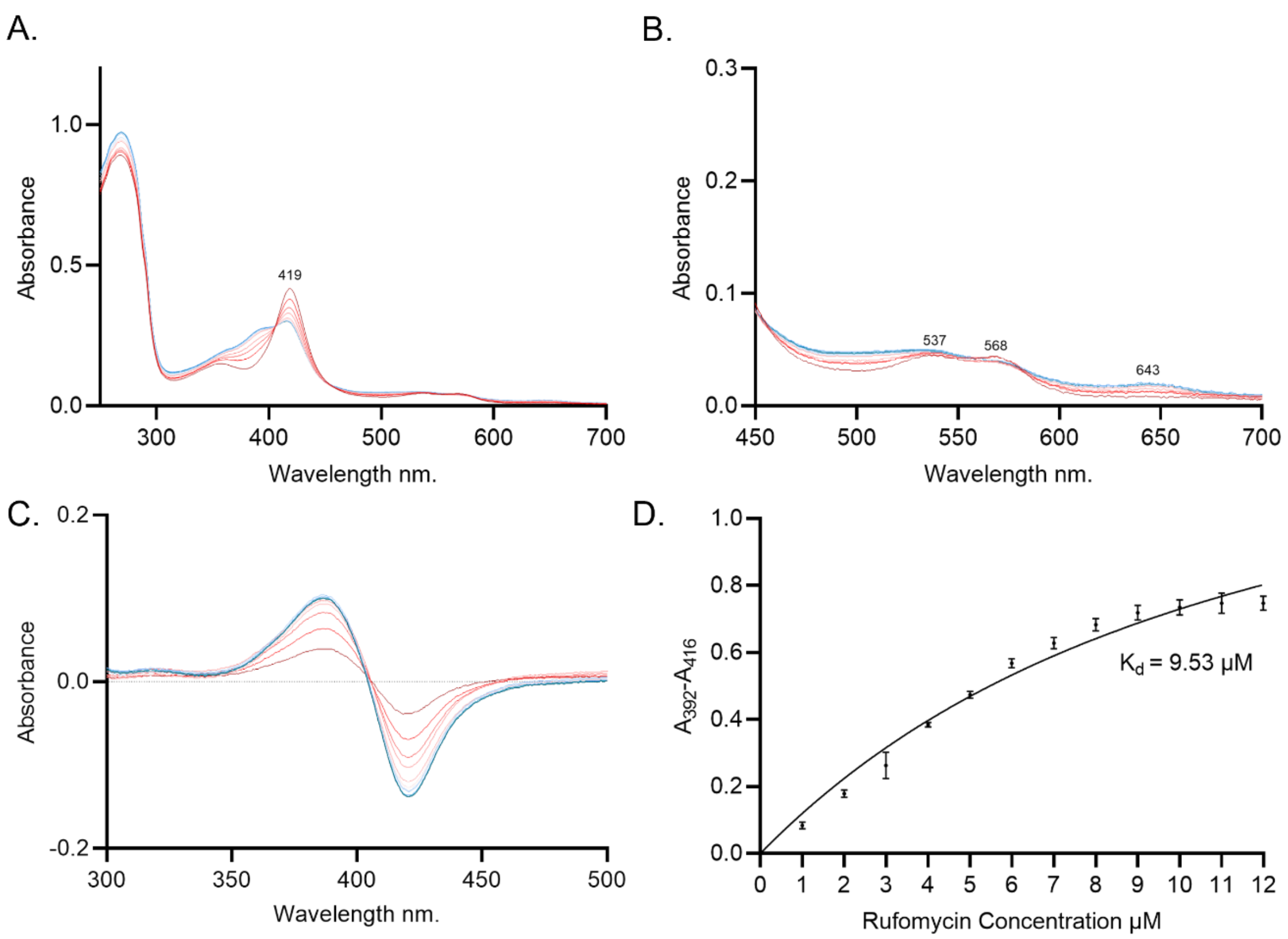

Figure S16: UV-Vis spectra studies of N242T RufM with increasing concentrations of 11 (0 - 12 µM). A) UV-visible spectra 250-600 nm. Low spin iron (II) peak identified at 419 nm for the non-substrate bound protein and high spin iron (II) identified at 392 nm with 12 µM substrate bound. B) Zoom in (×3) of the UV-visible spectra 470-700 nm showing the absorbance maxima for the substrate free protein at 537 nm and 568 nm. The substrate bound protein shows a maxima at 643 nm. C) Absorbance difference between 391 and 416 nm spectra with increasing concentrations with an isosbestic point at 404 nm. D) Absorbance difference plotted versus **11** concentration used to determine a K_d value of 9.53 µM ± 0.556 µM.

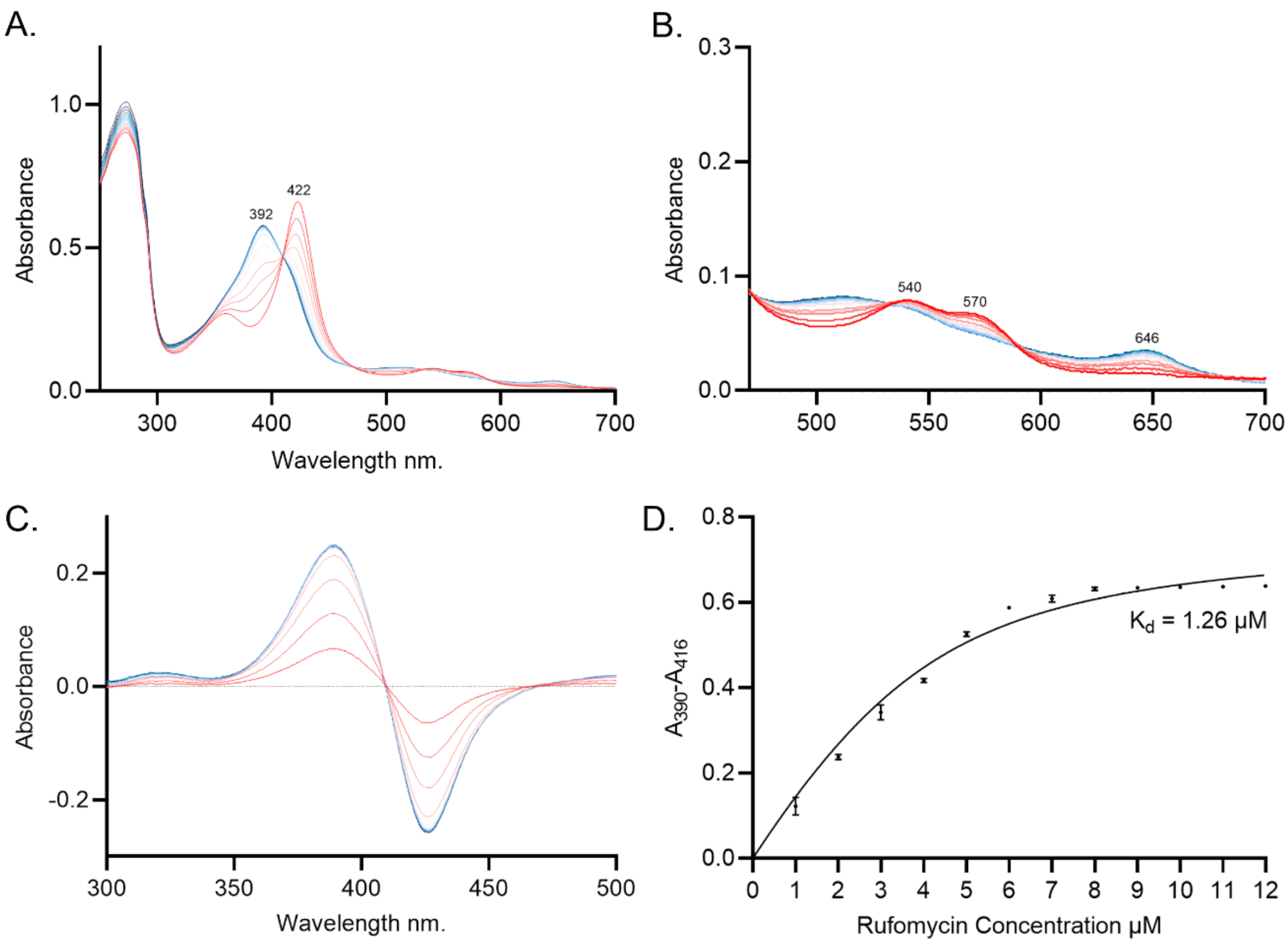

Figure S17: UV-Vis spectra studies of N242L RufM with increasing concentrations of 11 (0 - 12 µM). A) UV-visible spectra 250-600 nm. Low spin iron (II) peak identified at 416 nm for the non-substrate bound protein and high spin iron (II) identified at 392 nm with 12 µM substrate bound. B) Zoom in (×3) of the UV-visible spectra 470-700 nm showing the absorbance maxima for the substrate free protein at 540 nm and 570 nm. The substrate bound protein shows a maxima at 646 nm. C) Absorbance difference between 392 and 422 nm spectra with increasing concentrations with an isosbestic point at 404 nm. D) Absorbance difference plotted versus **11** concentration used to determine a K_d value of 1.26 µM ± 0.189 µM.

2.6.2 Method 2: K_d calculation

As the concentration of the enzyme was relatively high compared to the apparent K_d and similar to the concentration of rufomycin added, the concentration of free rufomycin in solution was significantly different to the amount added. Therefore, the apparent K_d value was then determined by fitting the following equation to the data:

Equation S2:
$$\Delta A = \frac{\Delta A_{max}}{2P}\left[P + S + K_d - \sqrt{(P + S + K_d)^2 - 4PS}\right]$$

Where P is the total enzyme concentration and S is the total substrate concentration.

Alternatively, the data was processed using the approximation that the amount of free rufomycin was equal to the amount added minus the amount bound to the protein.

Equation S3: $$S_{Free} = S - P\frac{\Delta A}{\Delta A_{max}}$$

This data was subsequently fit using the one site, specific binding nonlinear regression model in graphpad prism

Equation S4: $$\Delta A = \frac{\Delta A_{max} S_{Free}}{K_d + S_{Free}}$$

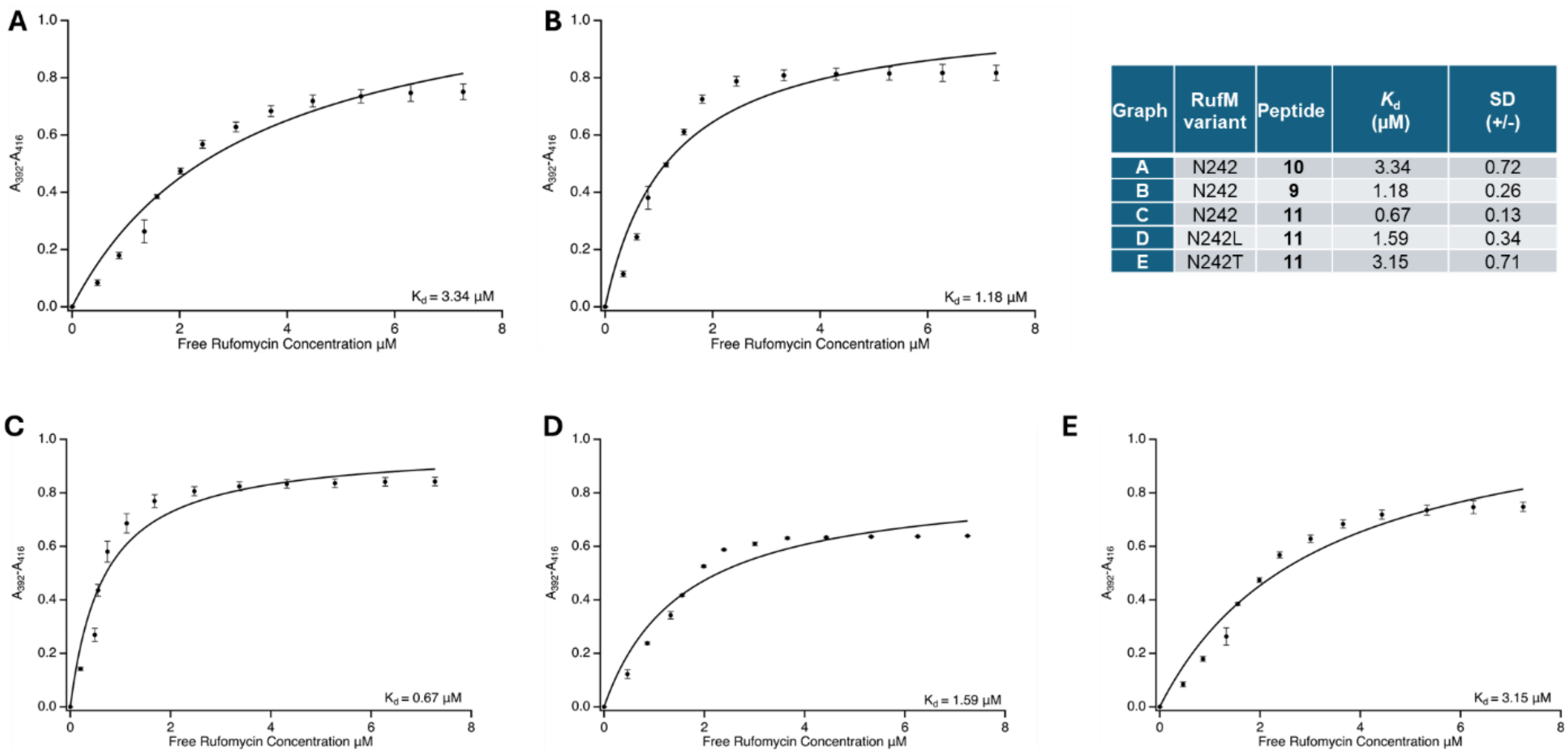

Graph	RufM variant	Peptide	K_d (µM)	SD (+/-)
A	N242	**10**	3.34	0.72
B	N242	**9**	1.18	0.26
C	N242	**11**	0.67	0.13
D	N242L	**11**	1.59	0.34
E	N242T	**11**	3.15	0.71

Figure S18: Alternative fittings for binding curves of RufM and mutants with peptides **9**, **10** and **11** (0-12 µM) calculated using equations S2 and S3. Absorbance difference plotted versus peptide concentration used to determine a K_d values of **A**) peptide **10** with RufM WT - 3.34 µM ± 0.72 µM. **B**.) peptide **9** with RufM WT - 1.18 µM ± 0.26 µM. **C**) peptide **11** with RufM WT - 0.67 µM ± 0.13 µM. **D**) peptide **11** with RufM N242L – 1.59 µM ± 0.34 µM. **E**) peptide **12** with RufM N242T – 3.15 µM ± 0.71 µM. **Inset**: table summarising K_d data.

2.7. Catalytic Activity of RufM and Mutants

The activity assays were carried with mutants at 20 µM according to their heme incorporation (Table S2), or as indicated, with commercial ferredoxin (final conc. 0.08 g L^{-1}) and ferredoxin reductase (final conc. 0.08 U) from *Spinacia oleracea* (Sigma) or as indicated. Rufomycin's in DMSO (final conc. 50 µM) were used as substrates. 25 mM Tris buffer pH 8 was added to reach a final volume of 100 µL. Nicotinamide adenine dinucleotide phosphate (NADPH final conc. 1 mM) was added last to initiate the reaction. The reactions were incubated for 3 hours at 30 °C with shaking (800 rpm) in a ThermoMixer C (Eppendorf). Negative controls were carried out as above, but the enzymes were inactivated prior to addition by heating (>100 °C, 15 minutes). After 3 hours, the reactions and the negative controls were extracted with ethyl acetate (2 × 500 µL). The solvent layers were collected and evaporated to dryness. The solid was resuspended in 50 µL 5:95 water:acetonitrile. The samples were analysed by LC-MS using Gradient 1. Percentage conversions were determined using the area under the peaks of determined products.

Material	Stock	Initial Concentration	Final Concentration	Vol (µL)
RufM	Stock in Buffer D	-	20 µM	-

Ferredoxin	0.5 mL buffer D added to vial, in 50 uL aliquots	4 g L^{-1} (45.45 μM)	0.08 g L^{-1}	2 μL
Ferredoxin Red	0.5 mL buffer D added to vial, in 50 uL aliquots	10 U in 1 mL buffer	0.08 U	2 uL
NADPH	2 mg in 50 μL Tris buffer	50 mM	1 mM	2 μL
Rufomycin	Stock in DMSO	-	50 μM	-
DMSO	Used to dissolve rufomycin	100%	2%	-
Tris Buffer	25 mM pH 8	25 mM	25mM	Up to 100 μL

Table S4: Reaction mix used to measure the catalytic activity of RufM and its mutants.

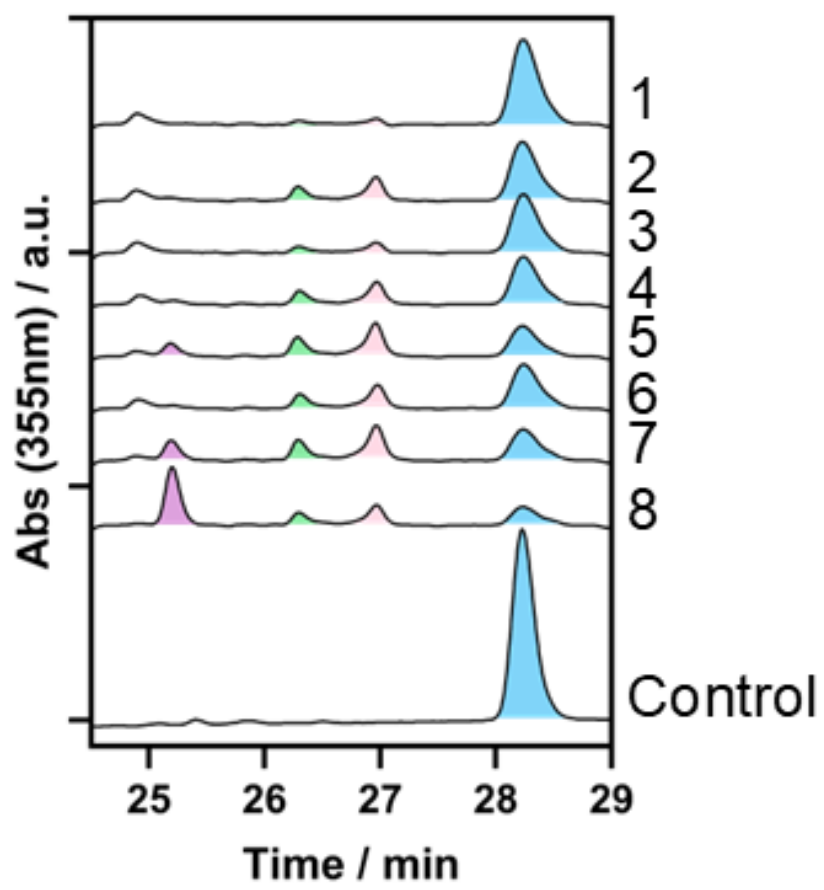

	Ferredoxin (g/L)	Ferredoxin reductase (U)
1	0.08	0.02
2	0.08	0.08
3	0.16	0.02
4	0.16	0.04
5	0.16	0.08
6	0.32	0.02
7	0.32	0.04
8	0.32	0.08

Figure S19: Optimising concentrations of Ferredoxin and Ferredoxin reductase in RufM assays. Left) LC-MS analysis (355 nm) showing products formed by RufM (N242) with peptide **5** at different concentrations of ferredoxin and ferredoxin reductase. Products are assigned by colour where blue = starting substrate, green = aldehyde product, pink = dehydrated product, purple = carboxylic acid. Right) table indicating concentrations used. All other conditions as described in Table S4.

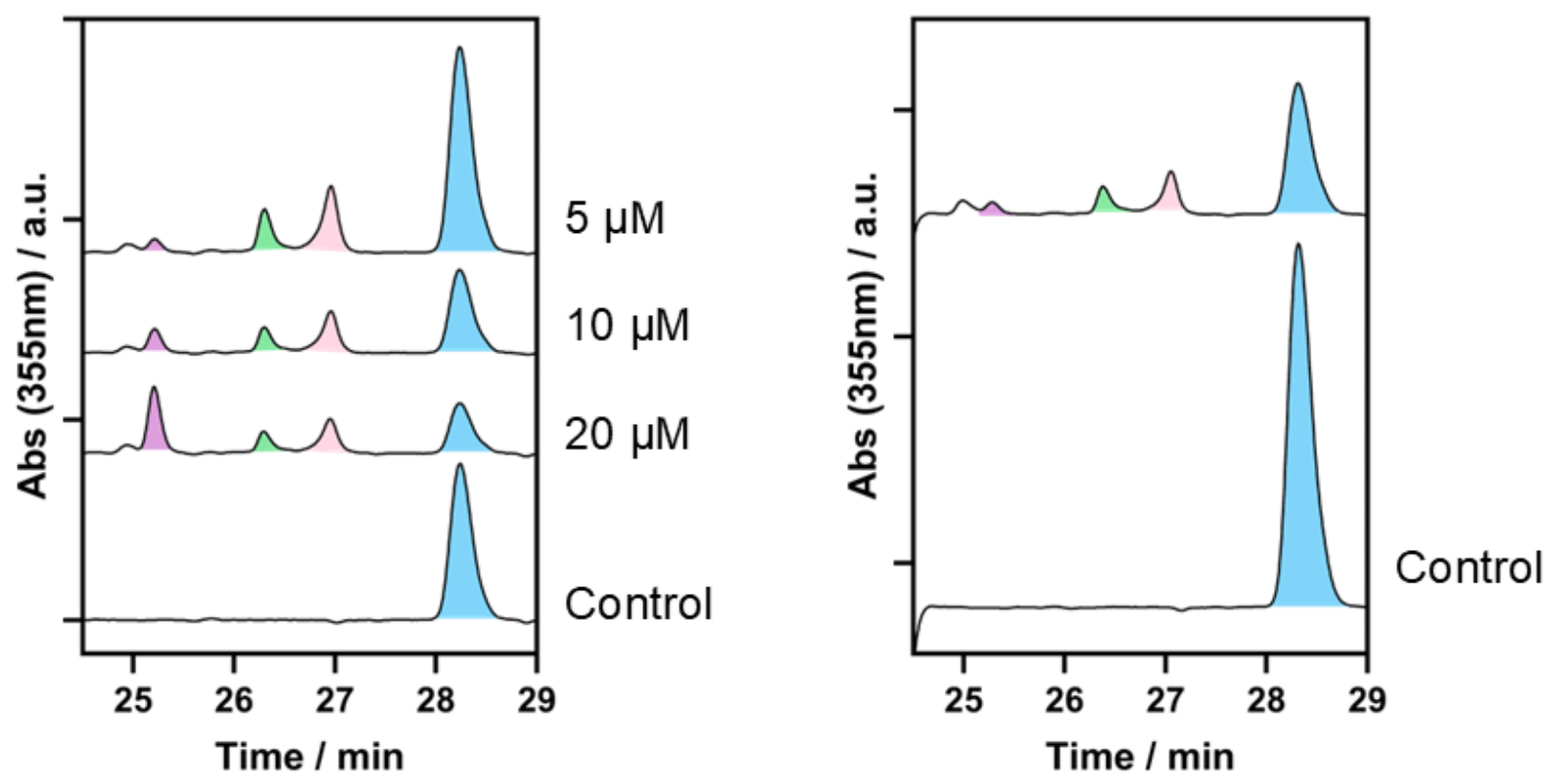

Figure S20: Optimising concentration of RufM (N242). Left) LC-MS trace at 355 nm showing product formation by different concentrations of RufM (N242) with peptide **5** (50µM) Right) Negative control (bottom) and RufM N242 (20 µM) reacted with 80 µM peptide **5** (right). Products are assigned by colour where blue = starting substrate, green = aldehyde product, pink = dehydrated product, purple = carboxylic acid product. All other conditions were as given in table S4.

Equation S5: for the time course reaction of natural substrate 1 by RufM.

$$Y(t) = Y_{min} + \frac{Y_{max} - Y_{min}}{1 + 10^{(t_{50} - t)h}}$$

Where Y(t) is the percentage conversion at time t, Y_{min} is the lower asymptote (baseline conversion), Y_{max} is the upper asymptote (maximum conversion), t_{50} is the time at which 50% conversion is achieved, and h is the Hill slope.

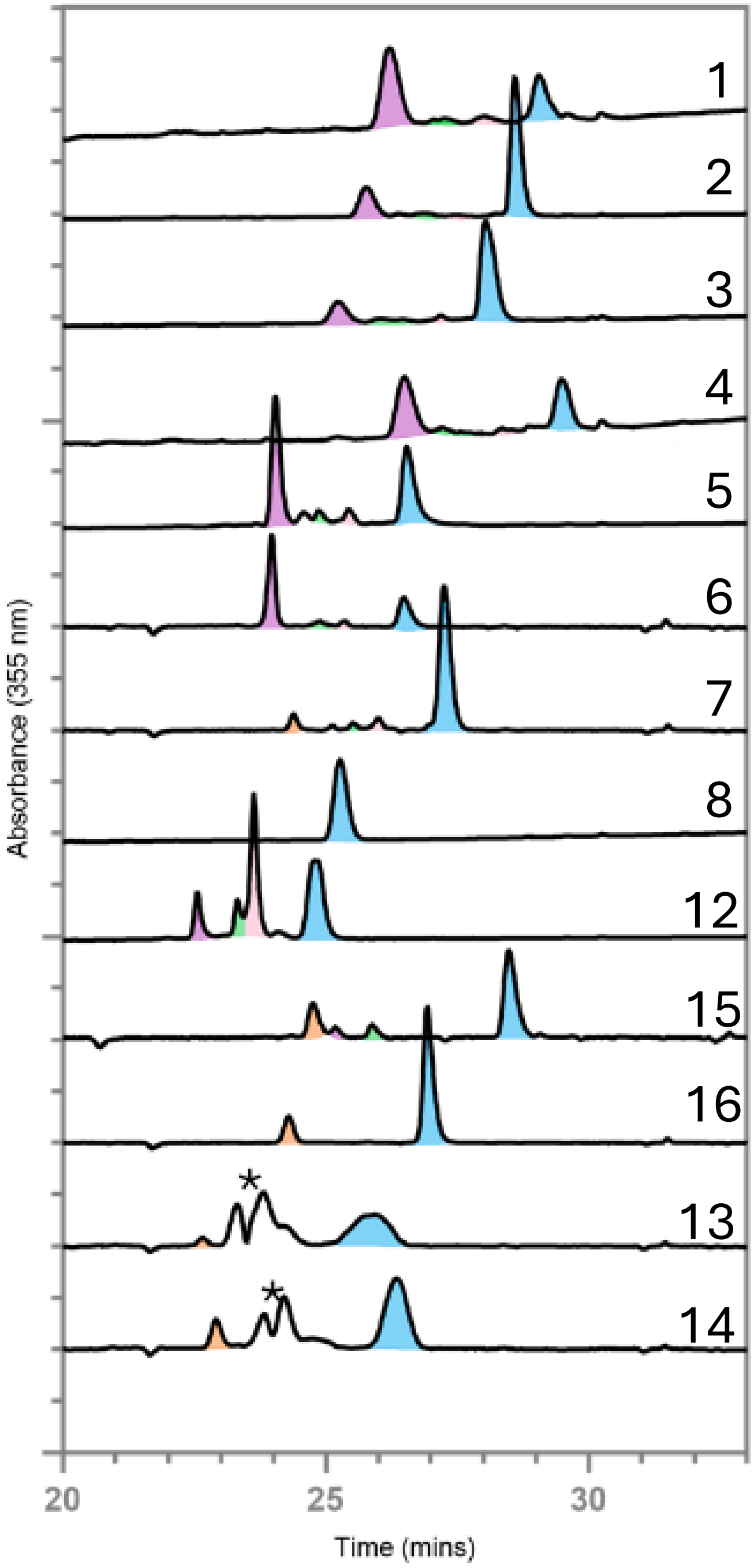

Figure S21: **RufM Wild Type (N242) LC-MS assay with peptides 1-8, 12-16, measured at absorbance 355 nm.** Products are assigned by colour where blue = starting substrate (**1-8, 12-16**), orange = alcohol product (**1a-8a, 12a-16a**), green = aldehyde product (**1b/b*-8b/b*, 12-16b/b***), pink = dehydrated product (**1d-8d, 12d-16d**), purple = carboxylic acid product (**1c-8c, 12-c16c**). The symbol * indicates where products are not easily separated via this gradient.

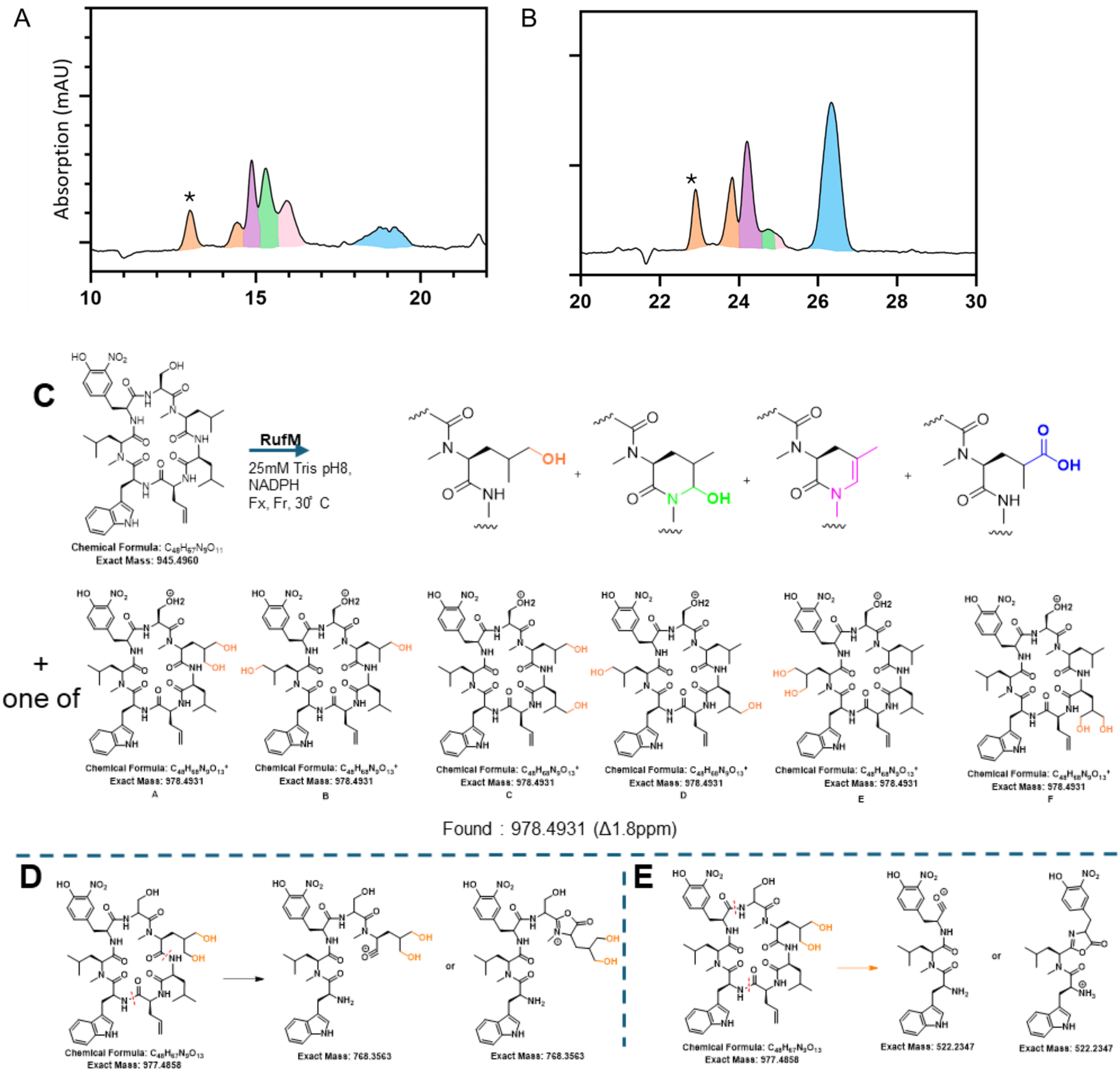

Figure S22: RufM Wild Type (N242) LC-MS assay with Peptides 13 and 14 A) LCMS chromatogram of RufM reaction with peptide **13** with an optimised LC method for better separation (1 min with 40% ACN mixed with 60% water, linear gradient from 40%- 90% ACN over 38 mins, then wash 2 min with 95% ACN, then back to 5% ACN over 2 min). at 355 nm **B)** LCMS chromatogram of RufM reaction with peptide **14** with 40-90% gradient for separation at 355 nm . Products are assigned by colour where blue = starting substrate (**13** or **14**), orange = alcohol/diol product, green = aldehyde product, pink = dehydrated produc, purple = carboxylic acid product. The symbol * represents the product with multiple alcohol groups. C) Reaction scheme with peptide **13** and products including possible structures of dihydroxylated product * D) Fragmentation observed rules out possible structures **C**, **D**, **F**. **E)** Fragment observed rules out structures **B** and **D**. Thus, **A** is the most likely product.

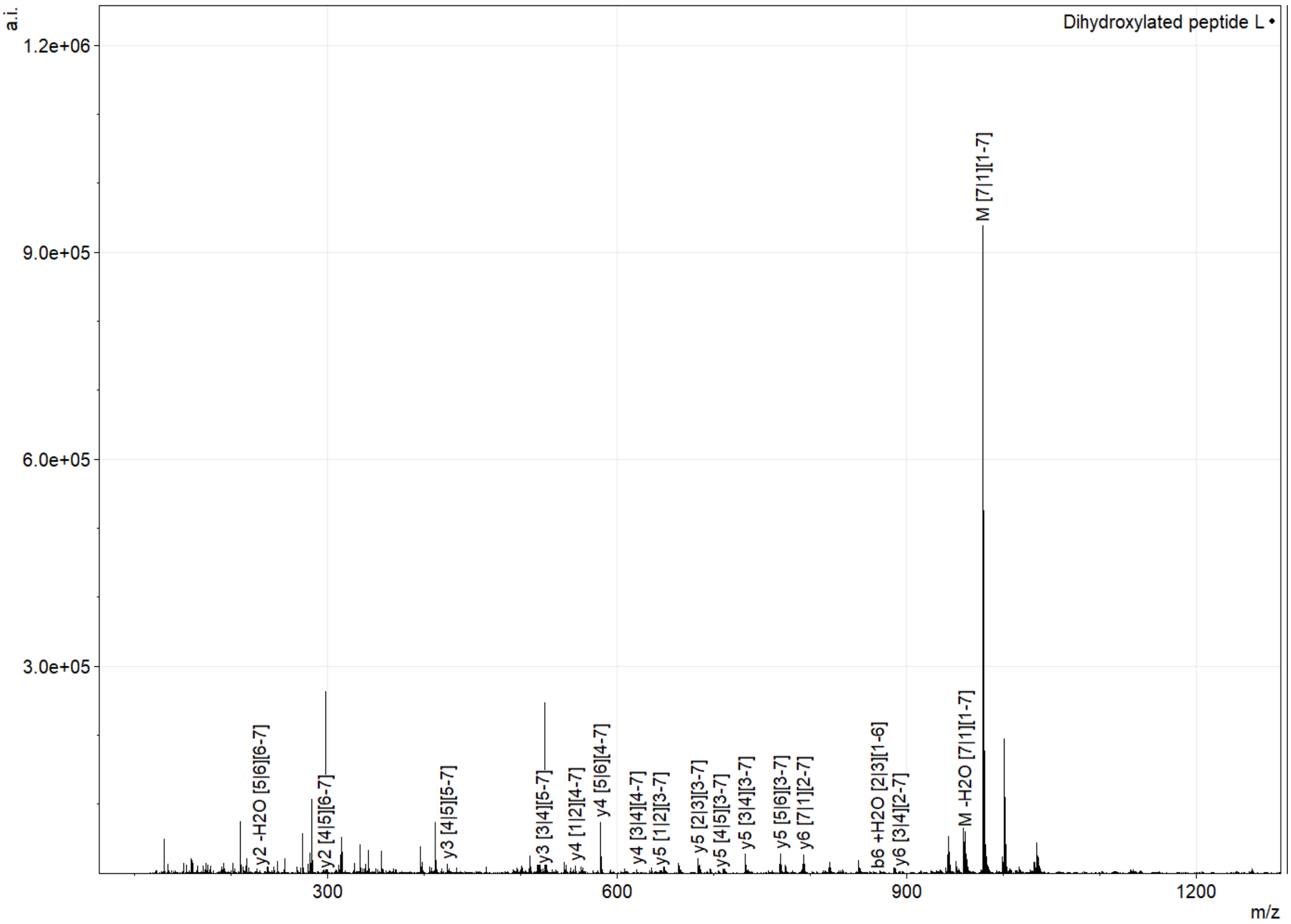

Figure S23: MS^e spectrum of dihydroxylated product from reaction of RufM with peptide 11. y-ions are labelled.

Ion	Slice	m/z	z	Sequence	Mass error / ppm
M	[7|1][1-7]	978.4931	1	. [Trp | NMe-Leu | 3NO2-Tyr | Ser | NMe-Leu-2OH | Leu | AllylG] .	1.8
M -H2O	[7|1][1-7]	960.4825	1	. [Trp | NMe-Leu | 3NO2-Tyr | Ser | NMe-Leu-2OH | Leu | AllylG] .	1.3
a2	[6|7][1-2]	256.1444	1	. [AllylG | Trp] . NMe-Leu	-1
a3	[1|2][1-3]	198.0999	2	. [NMe-Leu | 3NO2-Tyr | Ser] . NMe-Leu-2OH	-2
a4	[3|4][1-4]	429.2708	1	. [Ser | NMe-Leu-2OH | Leu | AllylG] . Trp	4.3
a4	[7|1][1-4]	581.2718	1	. [Trp | NMe-Leu | 3NO2-Tyr | Ser] . NMe-Leu-2OH	0.9
a4 - H2O	[1|2][1-4]	536.2715	1	. [NMe-Leu | 3NO2-Tyr | Ser | NMe-Leu-2OH] . Leu	-0.7
a5	[5|6][1-5]	704.3766	1	. [Leu | AllylG | Trp | NMe-Leu | 3NO2-Tyr] . Ser	-1.6
a6 - H2O	[3|4][1-6]	362.7233	2	. [Ser | NMe-Leu-2OH | Leu | AllylG | Trp | NMe-Leu] . 3NO2-Tyr	-0.1
b2	[2|3][1-2]	296.0877	1	. [3NO2-Tyr | Ser] . NMe-Leu-2OH	2.9
b2 - H2O	[3|4][1-2]	229.1183	1	. [Ser | NMe-Leu-2OH] . Leu	0.3
b3	[1|2][1-3]	423.1874	1	. [NMe-Leu | 3NO2-Tyr | Ser] . NMe-Leu-2OH	3.5
b3	[7|1][1-3]	522.2347	1	. [Trp | NMe-Leu | 3NO2-Tyr] . Ser	-3
b4	[1|2][1-4]	582.277	1	. [NMe-Leu | 3NO2-Tyr | Ser | NMe-Leu-2OH] . Leu	4
b4	[2|3][1-4]	568.2613	1	. [3NO2-Tyr | Ser | NMe-Leu-2OH | Leu] . AllylG	-1.2
b4	[4|5][1-4]	556.313	1	. [NMe-Leu-2OH | Leu | AllylG | Trp] . NMe-Leu	0.3

b4	[6|7][1-4]	619.2875	1	. [AllylG | Trp | NMe-Leu | 3NO2-Tyr] . Ser	-1.7
b5	[3|4][1-5]	643.345	1	. [Ser | NMe-Leu-2OH | Leu | AllylG | Trp] . NMe-Leu	2.4
b5	[4|5][1-5]	683.4127	1	. [NMe-Leu-2OH | Leu | AllylG | Trp | NMe-Leu] . 3NO2-Tyr	1.5
b5	[5|6][1-5]	732.3715	1	. [Leu | AllylG | Trp | NMe-Leu | 3NO2-Tyr] . Ser	-1.6
b5	[6|7][1-5]	706.3195	1	. [AllylG | Trp | NMe-Leu | 3NO2-Tyr | Ser] . NMe-Leu-2OH	4.5
b5	[7|1][1-5]	768.3563	1	. [Trp | NMe-Leu | 3NO2-Tyr | Ser | NMe-Leu-2OH] . Leu	0
b5 - H2O	[4|5][1-5]	665.4021	1	. [NMe-Leu-2OH | Leu | AllylG | Trp | NMe-Leu] . 3NO2-Tyr	2.5
b6	[1|2][1-6]	792.4138	1	. [NMe-Leu | 3NO2-Tyr | Ser | NMe-Leu-2OH | Leu | AllylG] . Trp	4.3
b6	[4|5][1-6]	891.4611	1	. [NMe-Leu-2OH | Leu | AllylG | Trp | NMe-Leu | 3NO2-Tyr] . Ser	-0.5
b6 +H2O	[2|3][1-6]	869.404	1	. [3NO2-Tyr | Ser | NMe-Leu-2OH | Leu | AllylG | Trp] . NMe-Leu	4.3
c2 - H2O	[1|2][1-2]	335.1714	1	. [NMe-Leu | 3NO2-Tyr] . Ser	1.7
c2 - H2O	[1|2][1-2]	168.0893	2	. [NMe-Leu | 3NO2-Tyr] . Ser	-3.6
c4	[2|3][1-4]	293.1476	2	. [3NO2-Tyr | Ser | NMe-Leu-2OH | Leu] . AllylG	2.8
c5	[1|2][1-5]	712.3876	1	. [NMe-Leu | 3NO2-Tyr | Ser | NMe-Leu-2OH | Leu] . AllylG	4.7
c6	[7|1][1-6]	898.4669	1	. [Trp | NMe-Leu | 3NO2-Tyr | Ser | NMe-Leu-2OH | Leu] . AllylG	-4.3
c6 - H2O	[1|2][1-6]	791.4298	1	. [NMe-Leu | 3NO2-Tyr | Ser | NMe-Leu-2OH | Leu | AllylG] . Trp	-3.3
x3	[5|6][5-7]	481.1565	1	NMe-Leu . [3NO2-Tyr | Ser | NMe-Leu-2OH] .	-4.9
y2	[4|5][6-7]	296.0877	1	NMe-Leu . [3NO2-Tyr | Ser] .	2.9
y2 - H2O	[5|6][6-7]	229.1183	1	3NO2-Tyr . [Ser | NMe-Leu-2OH] .	0.3
y3	[3|4][5-7]	522.2347	1	AllylG . [Trp | NMe-Leu | 3NO2-Tyr] .	-3
y3	[4|5][5-7]	423.1874	1	Trp . [NMe-Leu | 3NO2-Tyr | Ser] .	3.5
y4	[1|2][4-7]	556.313	1	Ser . [NMe-Leu-2OH | Leu | AllylG | Trp] .	0.3
y4	[3|4][4-7]	619.2875	1	Leu . [AllylG | Trp | NMe-Leu | 3NO2-Tyr] .	-1.7
y4	[5|6][4-7]	582.277	1	Trp . [NMe-Leu | 3NO2-Tyr | Ser | NMe-Leu-2OH] .	4
y4	[6|7][4-7]	568.2613	1	NMe-Leu . [3NO2-Tyr | Ser | NMe-Leu-2OH | Leu] .	-1.2
y5	[1|2][3-7]	643.345	1	3NO2-Tyr . [Ser | NMe-Leu-2OH | Leu | AllylG | Trp] .	2.4
y5	[2|3][3-7]	683.4127	1	Ser . [NMe-Leu-2OH | Leu | AllylG | Trp | NMe-Leu] .	1.5
y5	[3|4][3-7]	732.3715	1	NMe-Leu-2OH . [Leu | AllylG | Trp | NMe-Leu | 3NO2-Tyr] .	-1.6
y5	[4|5][3-7]	706.3195	1	Leu . [AllylG | Trp | NMe-Leu | 3NO2-Tyr | Ser] .	4.5
y5	[5|6][3-7]	768.3563	1	AllylG . [Trp | NMe-Leu | 3NO2-Tyr | Ser | NMe-Leu-2OH] .	0
y5 - H2O	[2|3][3-7]	665.4021	1	Ser . [NMe-Leu-2OH | Leu | AllylG | Trp | NMe-Leu] .	2.5
y6	[3|4][2-7]	891.4611	1	Ser . [NMe-Leu-2OH | Leu | AllylG | Trp | NMe-Leu | 3NO2-Tyr] .	-0.5
y6	[7|1][2-7]	792.4138	1	Trp . [NMe-Leu | 3NO2-Tyr | Ser | NMe-Leu-2OH | Leu | AllylG] .	4.3

***Table S5: Full Table of fragments generated by MMass[8] analysis from LC-HRMS of Peptide 11 dihydroxylated product**. Fragments used to determine structure of the product in Figure S17 are highlighted in green.*

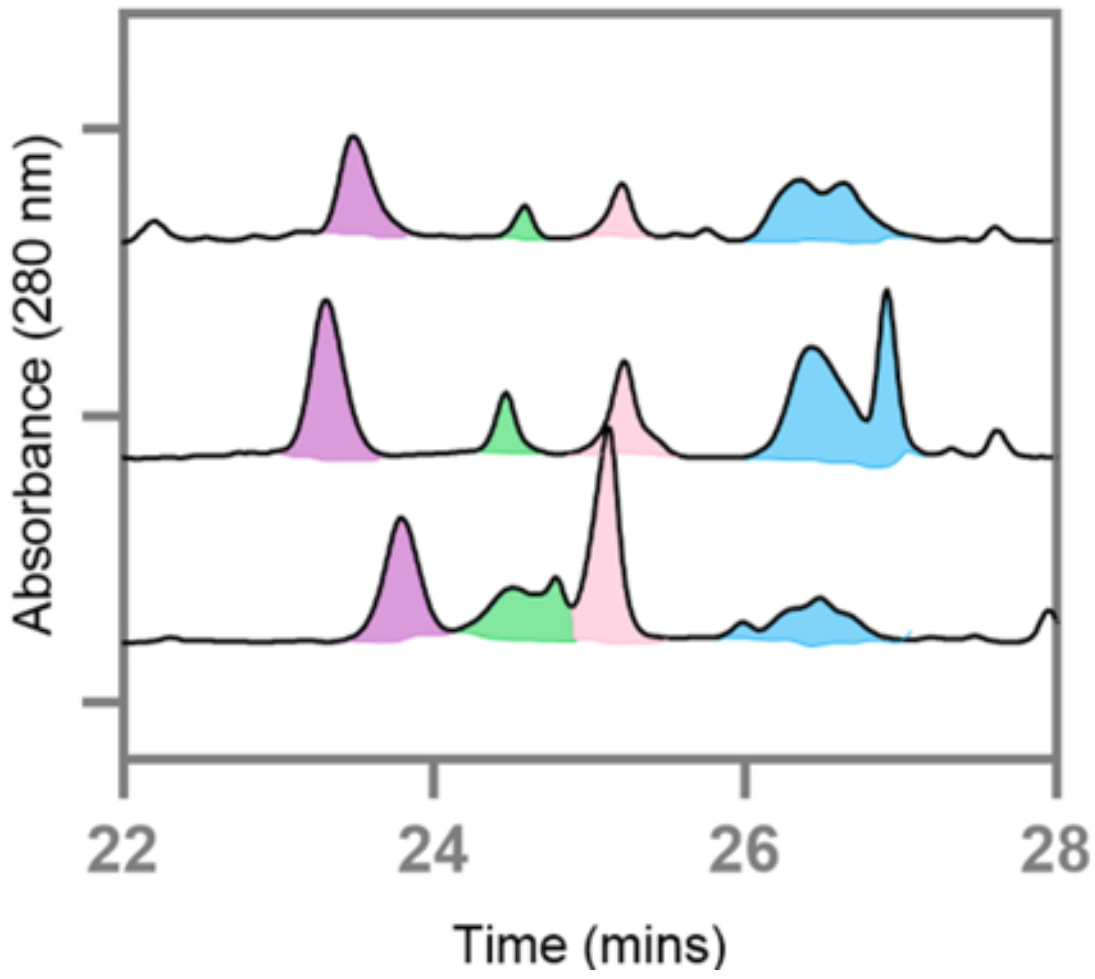

Figure S24: RufM Wild Type (N242) LC-MS assay with peptides 10(top), 9(middle) and 11(bottom) at absorbance 280 nm. Products are assigned by colour where blue = starting substrate (**9-11**), green = aldehyde product (**9b-11b**), pink = dehydrated product (**9d-11d**), purple = carboxylic acid product (**9c-11c**).

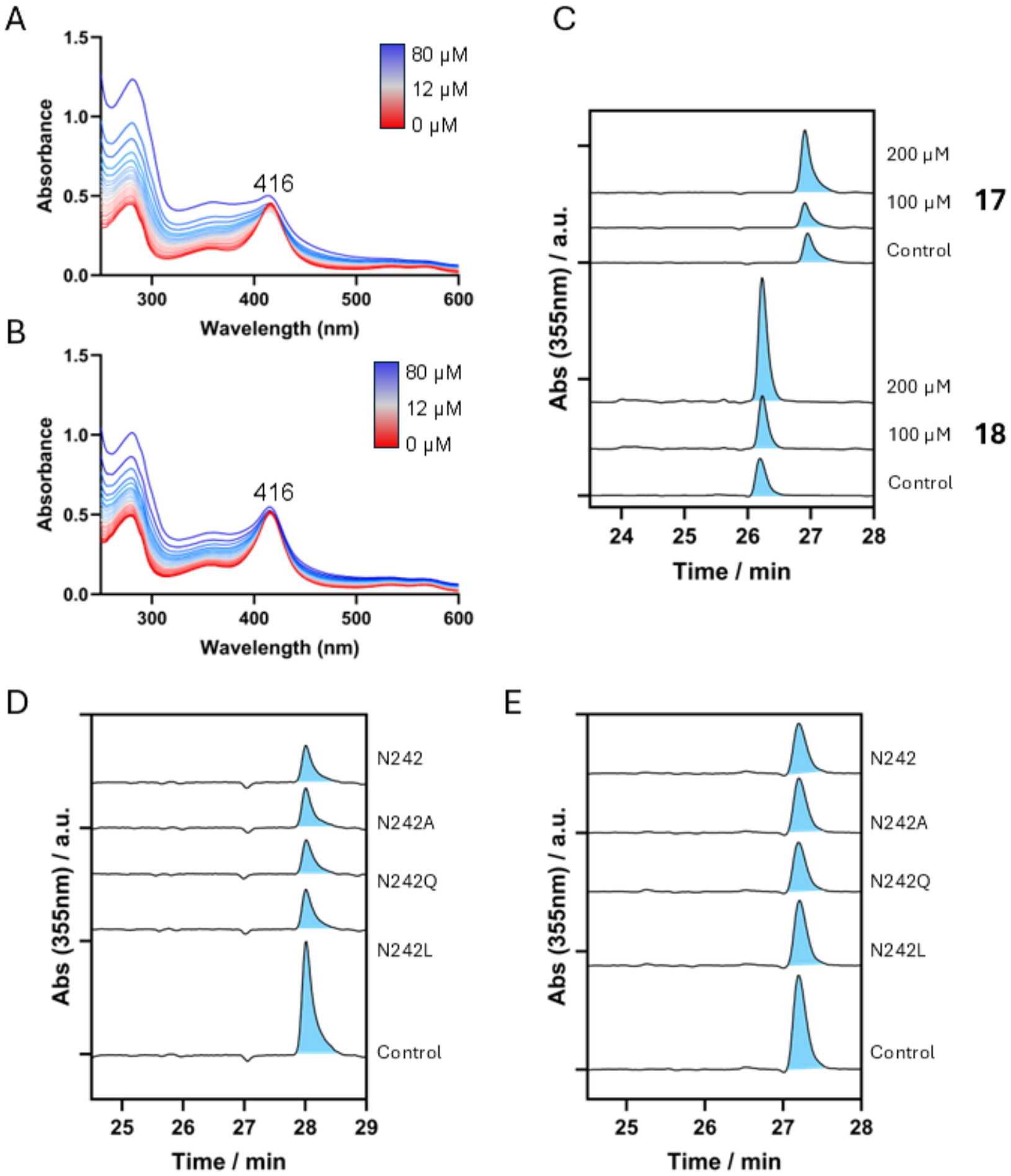

Figure S25: Reaction and binding of RufM with peptides **17** and **18**. Effect of peptides **17** and **18** on the visible absorption spectra of wild type RufM and their activity assay. **A)** UV-visible binding assay of N242 RufM (5 μM, 25mM Tris pH8) with increasing concentrations of **17** (0 - 80 μM). **B)** UV-visible spectra studies of N242 RufM (5 μM, 25mM Tris pH8) with increasing concentrations of **18** (0 - 80 μM). Low spin iron (II) peak identified at 416 nm for the non-substrate bound protein. **C**) LC-MS trace (355 nm) to analyse reactions of RufM N242 with peptides **17** and **18**. Controls contain heat inactivated RufM. Products are labelled by colour where blue = substrate. **D**) LC-MS trace at 355 nm showing product formation by RufM (N242, N242A, N242Q, N242L) with peptide **17**. **E)** LC-MS trace at 355 nm showing product formation by RufM (N242, N242A, N242Q, N242L) with peptide **18**.

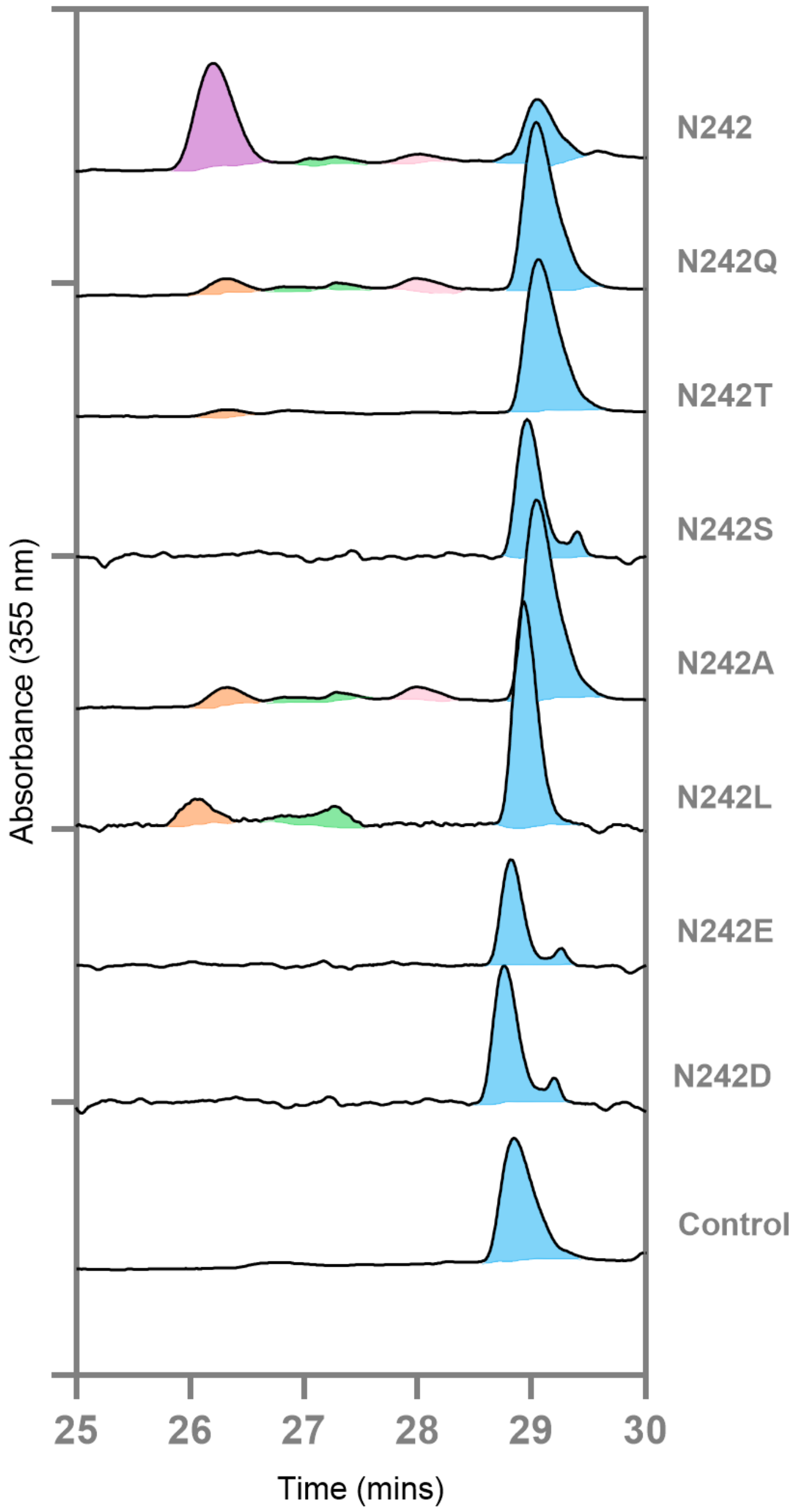

Figure S26: LC-MS assay trace of peptide 1. Traces show N242 and mutants N242Q, N242T, N242S, N242A, N242L, N242E and N242D recorded at absorbance 355 nm. Products are assigned by colour where blue = starting substrate **1**, orange = alcohol product **1a**, green = aldehyde product **1b**/**b***, pink = dehydrated product **1d**, purple = carboxylic acid product **1c**.

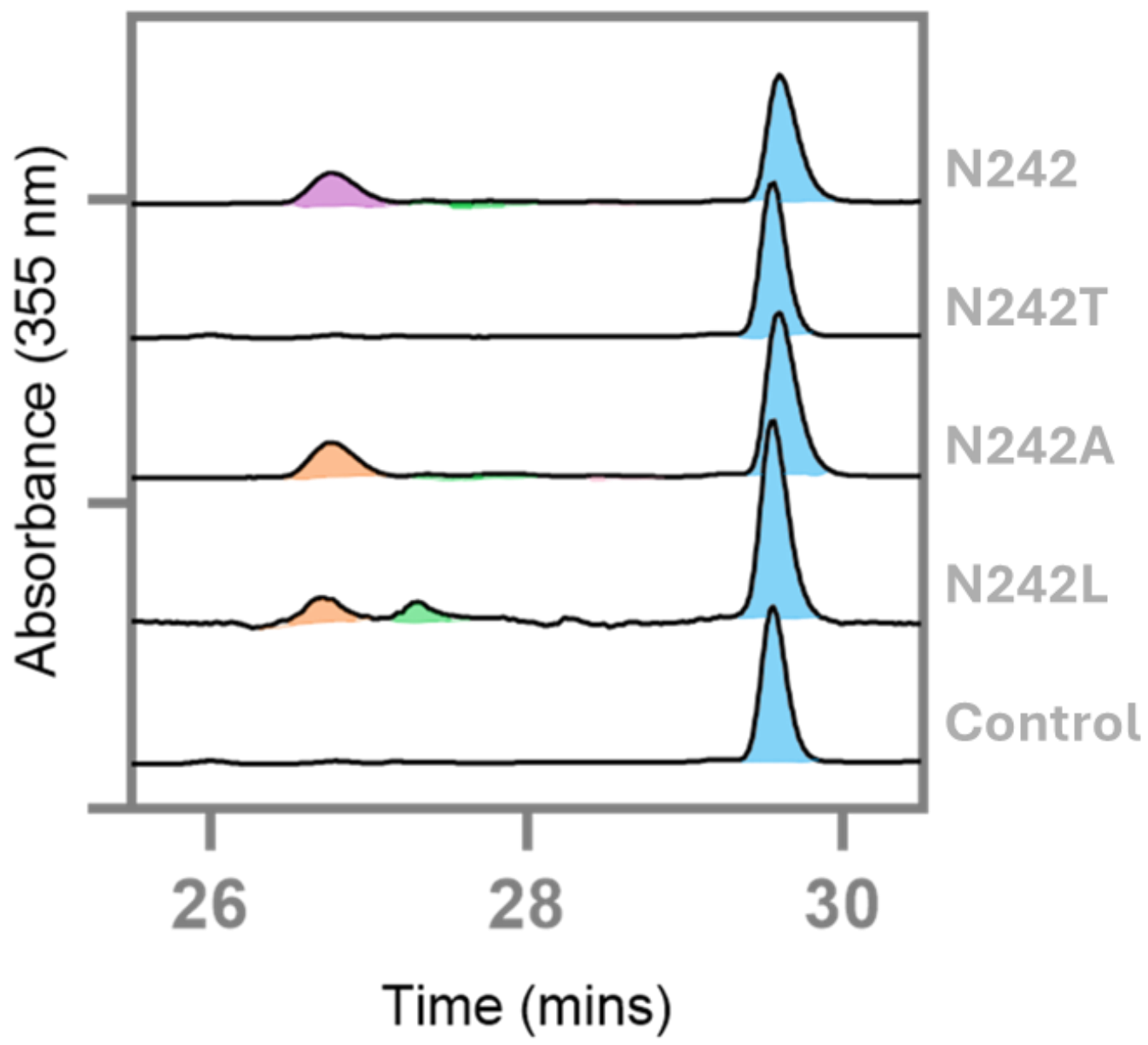

Figure S27: LC-MS assay trace of peptide 2. Traces show N242 and mutants N242T, N242S, N242A and N242L recorded at absorbance 355 nm. Products are assigned by colour where blue = starting substrate **2**, orange = alcohol product **2a**, green = aldehyde product **2b**/**b***, pink = dehydrated product **2d**, purple = carboxylic acid product **2c**

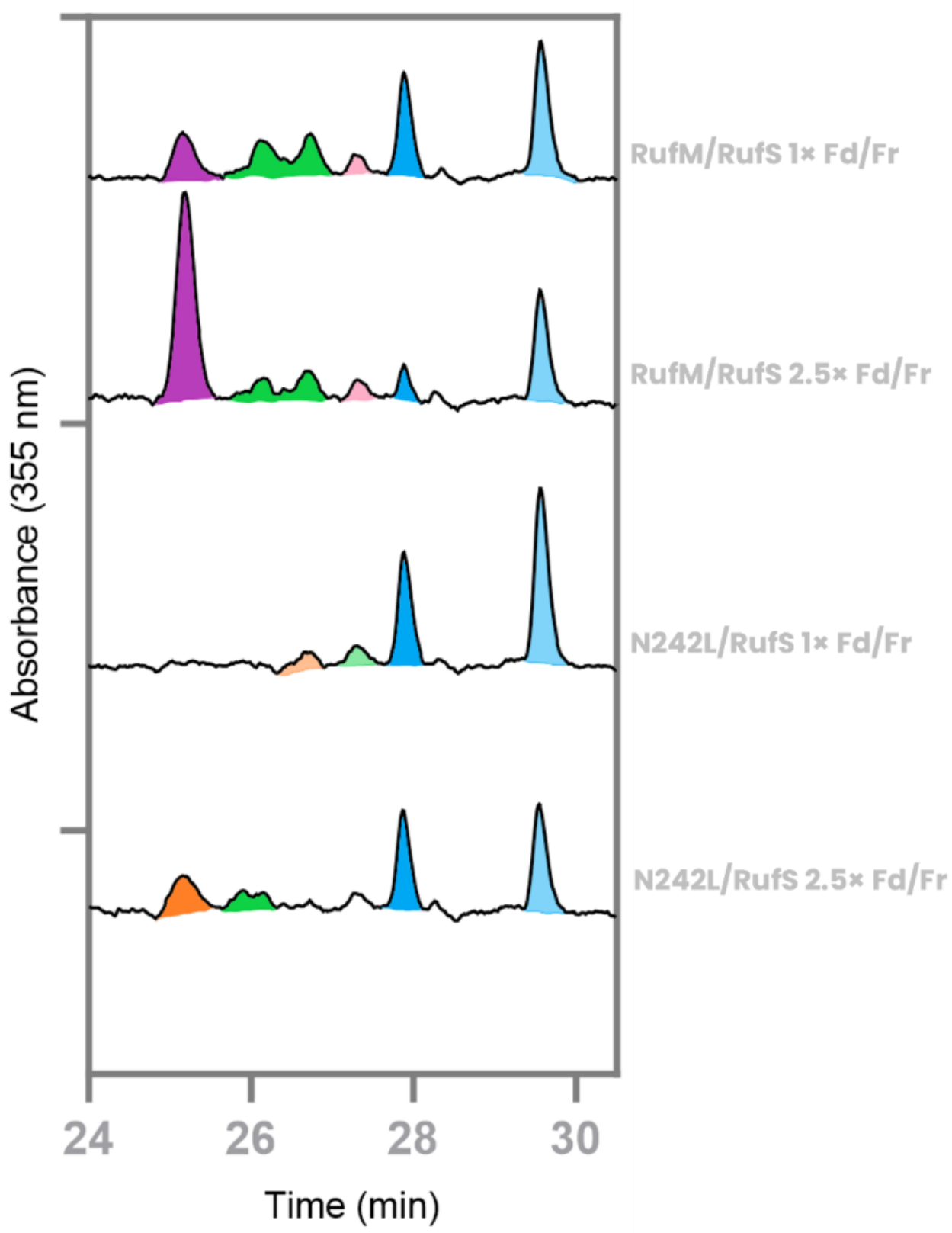

Figure S28: LC-MS assay trace of peptide 2 with varying amounts of Fd/ Fr. Traces show assays containing RufM N242/ RufS and mutant N242L/ RufS recorded at absorbance 355 nm. Products are assigned by colour where light blue = substrate **2**, dark blue =

substrate **2** with epoxide ***epoxy*-2**, orange = alcohol product **2a**, light green = aldehyde product **2b/b*,** dark green = aldehyde product with epoxide ***epoxy*2b/b*,** light pink = dehydrated product **2d**, dark pink = dehydrated product with epoxide ***epoxy*-2d**, light purple = carboxylic acid product **2c**, dark purple = carboxylic acid product with epoxide ***epoxy*2c**.

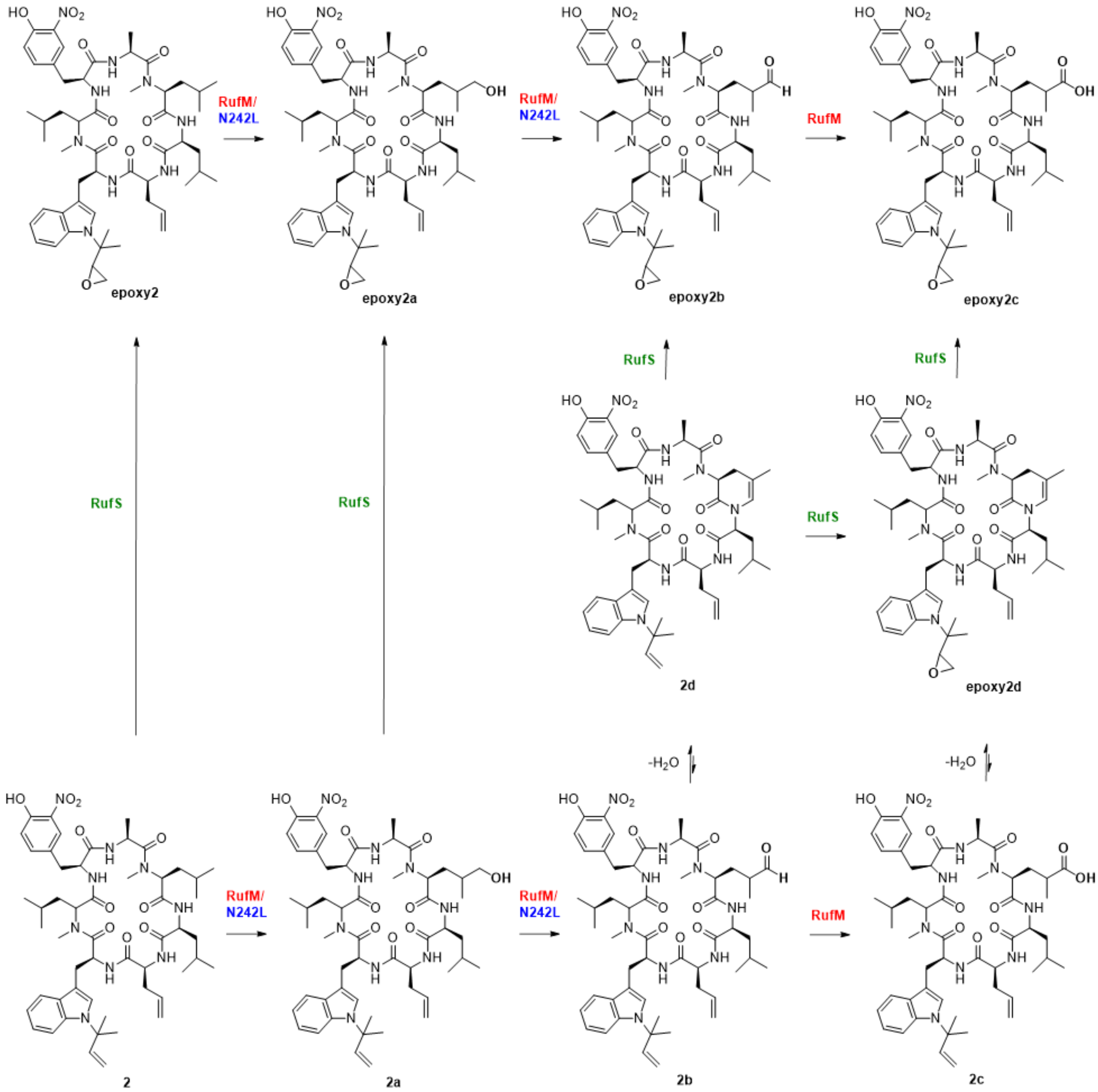

Figure S29: Transformation of 2 with RufM/ N242L and RufS.

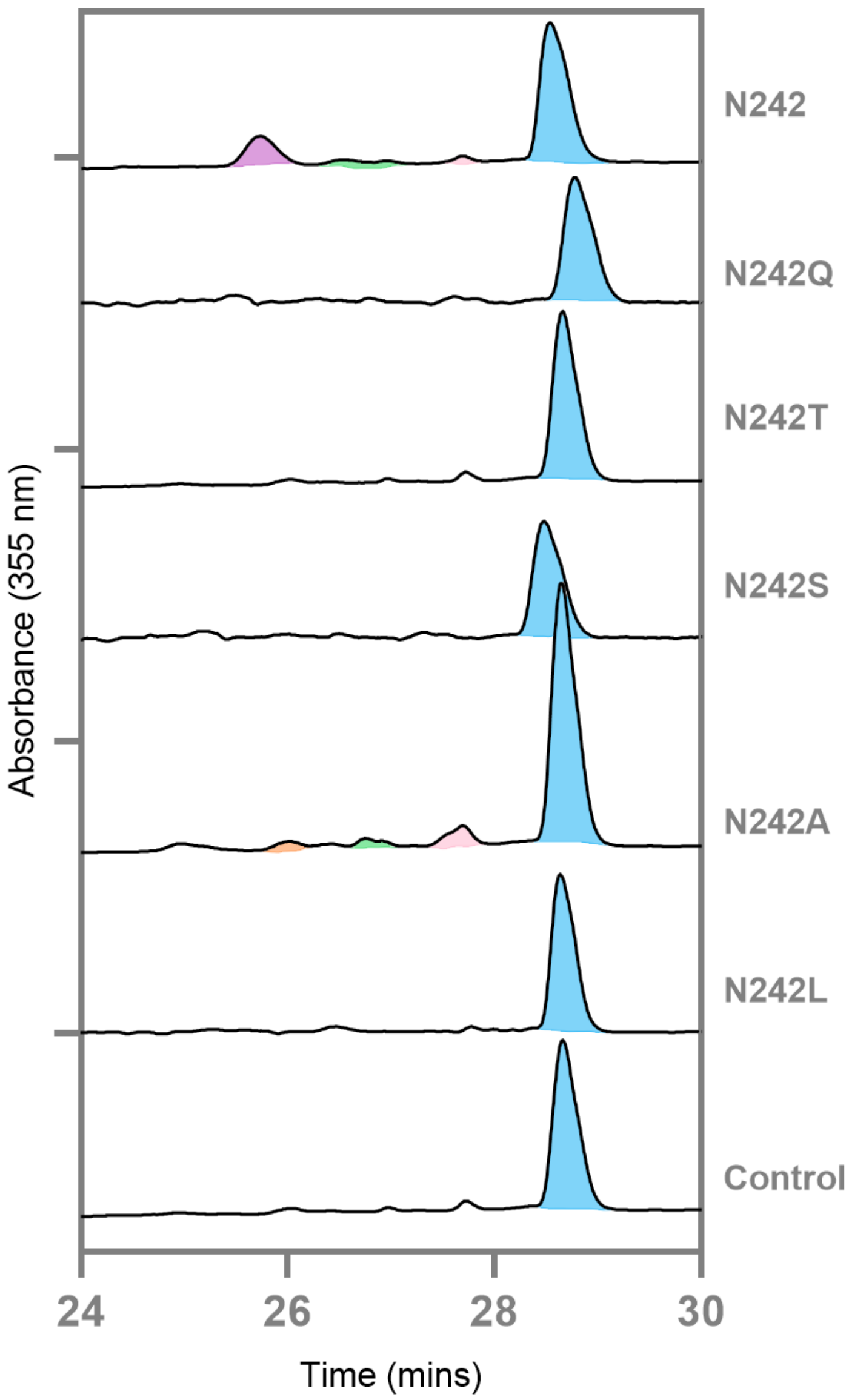

Figure S30: **LC-MS assay trace of peptide 3.** Traces show N242 and mutants N242Q, N242T, N242S, N242A and N242L recorded at absorbance 355 nm. Products are assigned by colour where blue = starting substrate **3**, orange = alcohol product **3a**, green = aldehyde product **3b**/**b***, pink = dehydrated product **3d**, purple = carboxylic acid product **3c** .

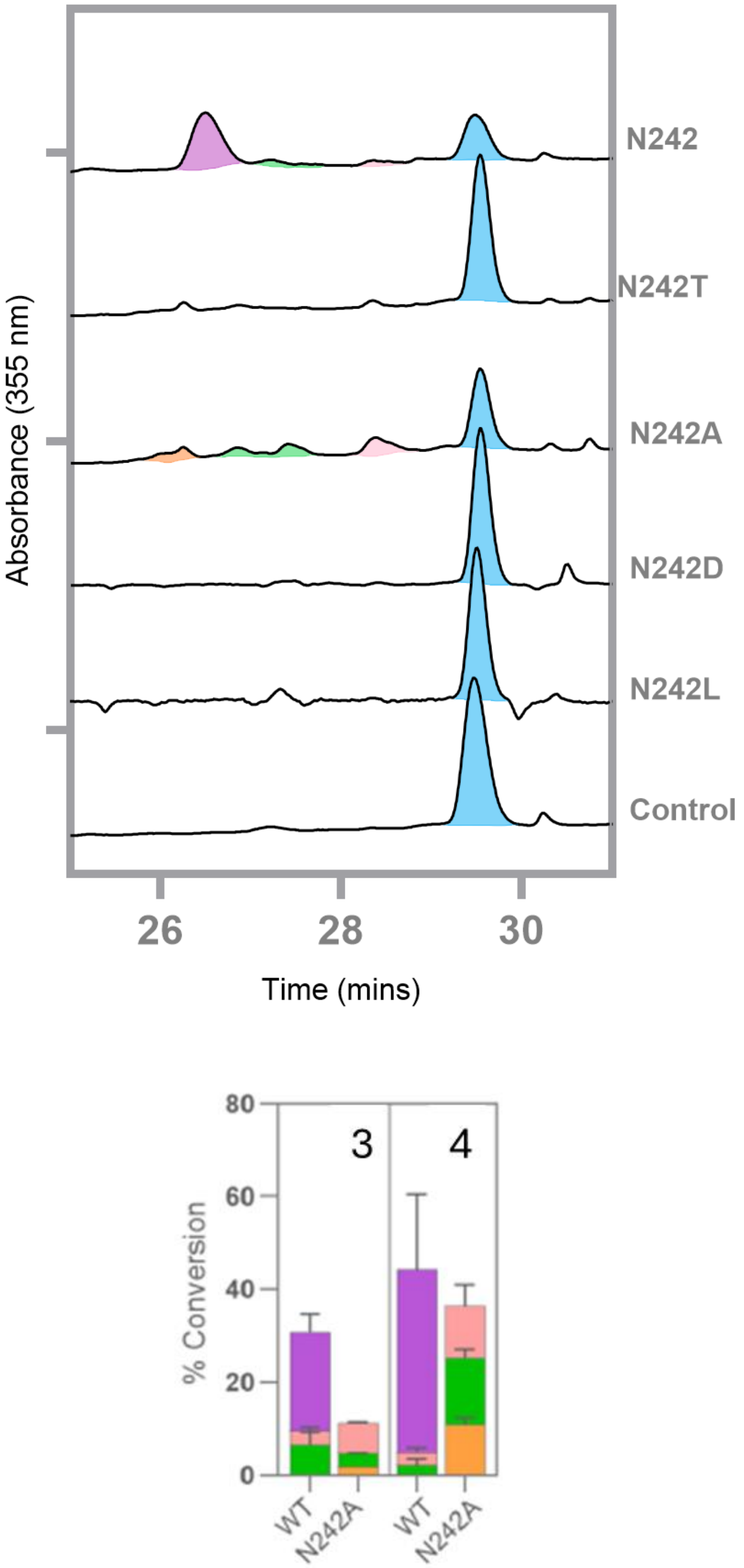

Figure S31: LC-MS assay trace of peptide 3 & 4. Top) Traces show N242 and mutants N242T, N242A, N242D and N242L recorded at absorbance 355 nm. Bottom) Chart showing proportion of products formed from reaction of RufM WT or N242A with peptides **3** & **4**. Conversions are averages of 3 biological repeats and were determined using LC-MS peak area at 355 nm. Products are assigned by colour where blue = starting substrate **2**, orange = alcohol product **4a**, green = aldehyde product **4b**/**b***, pink = dehydrated product **4d**, purple = carboxylic acid product **4c.**

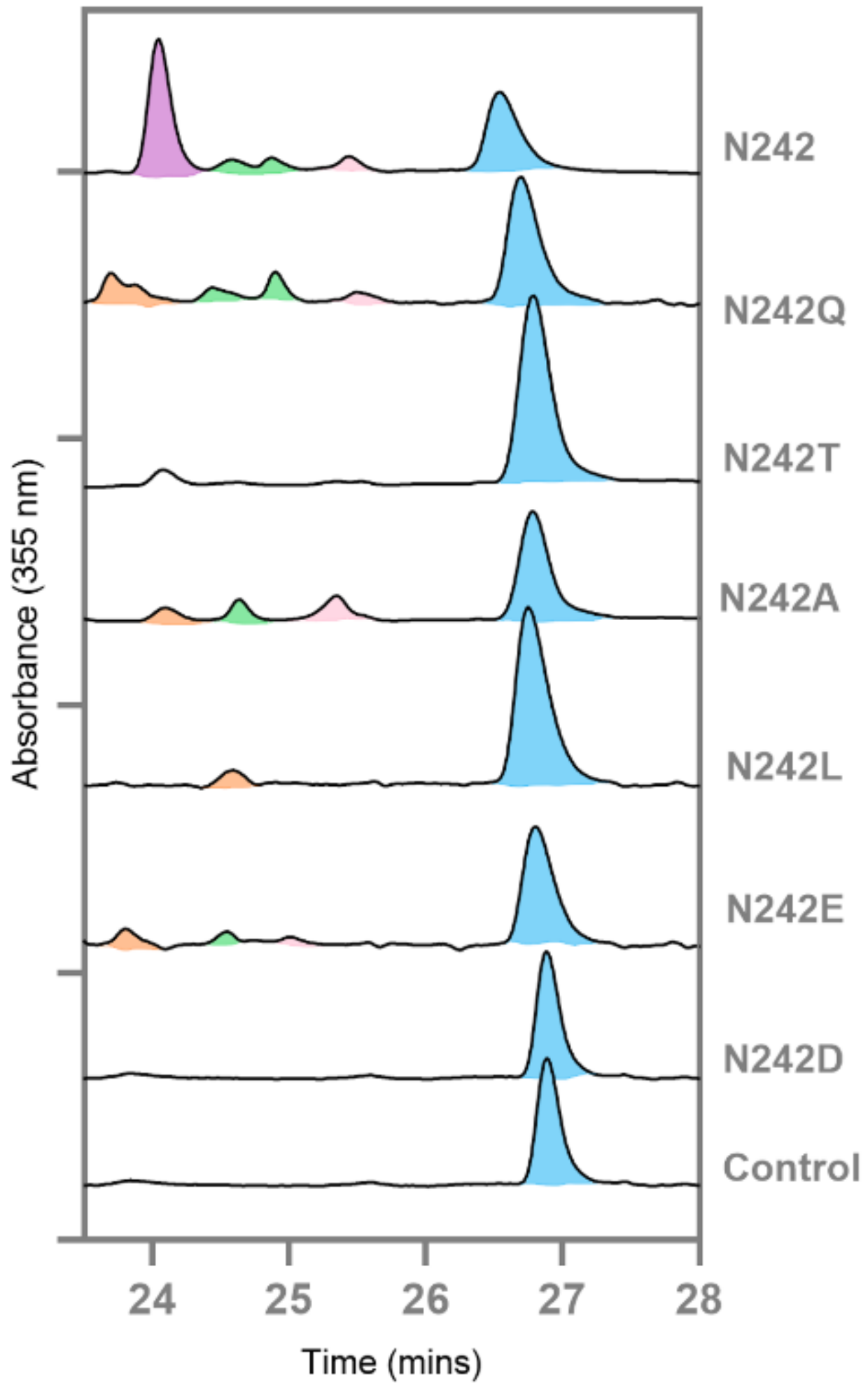

Figure S32: LC-MS assay trace of peptide 5. Traces show N242 and mutants N242Q, N242T, N242A, N242L, N242E and N242D recorded at absorbance 355 nm. Products are assigned by colour where blue = starting substrate **5**, orange = alcohol product **5a**, green = aldehyde product **5b**/**b***, pink = dehydrated product **5d**, purple = carboxylic acid product **5c**

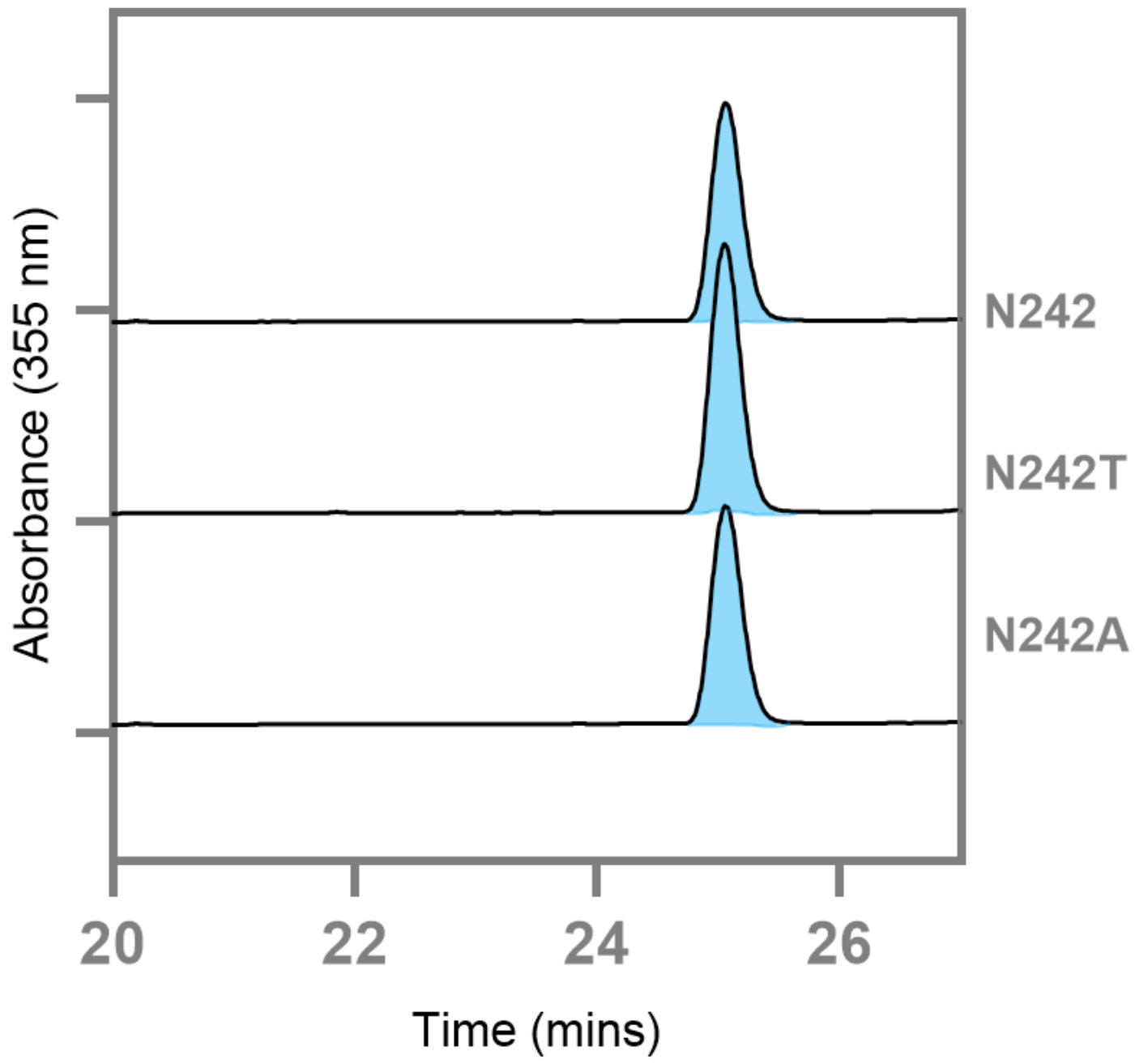

Figure S33: LC-MS assay trace of peptide 8. Traces show N242 and mutants N242T and N242A recorded at absorbance 355 nm. Products are assigned by colour where blue = starting substrate **8**.

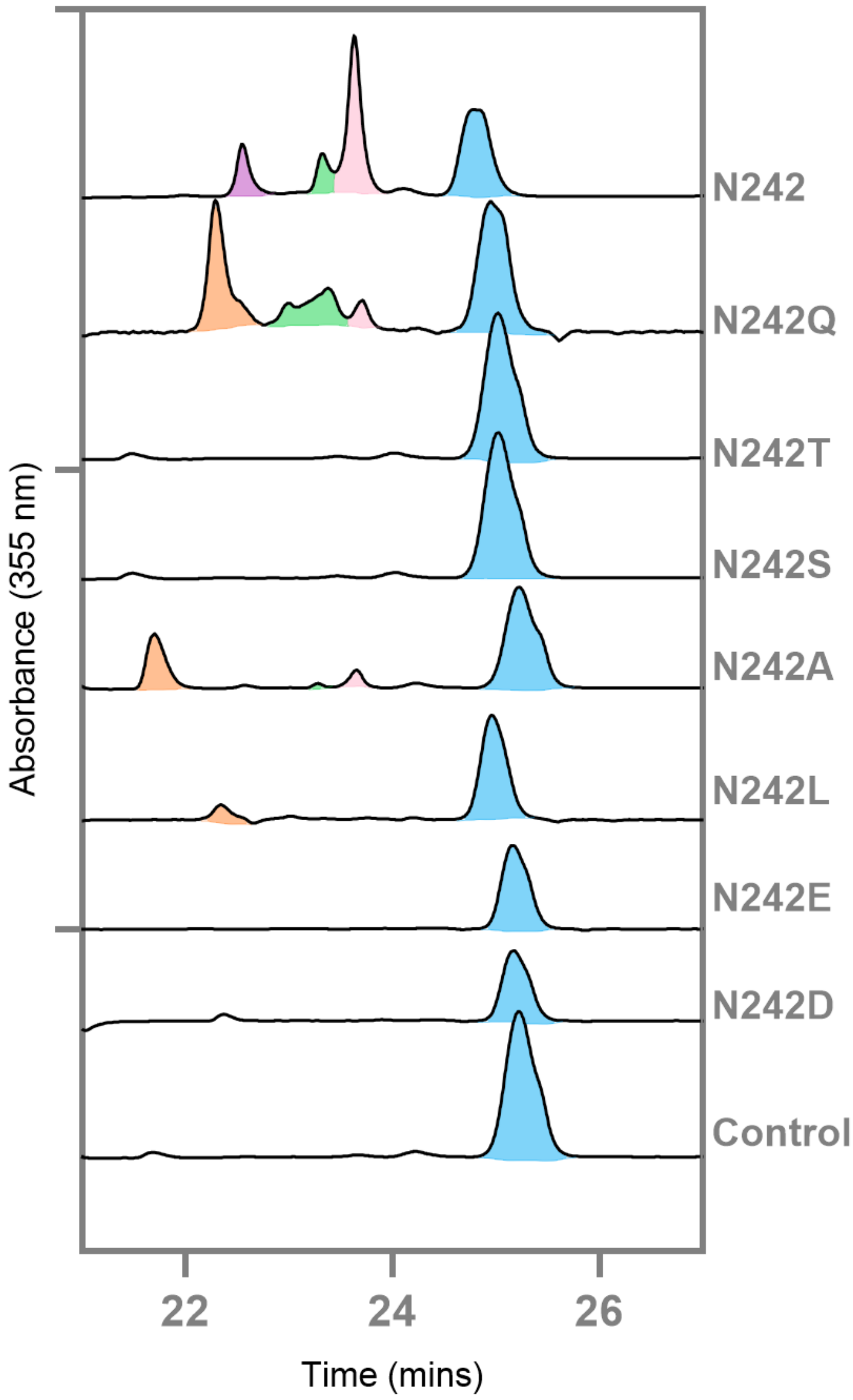

Figure S34: LC-MS assay trace of peptide 12. Traces show N242 and mutants N242Q, N242T, N242S, N242A, N242L, N242E and N242D recorded at absorbance 355 nm. Products are assigned by colour where blue = starting substrate **12**, orange = alcohol product **12a**, green = aldehyde product **12b**/**b***, pink = dehydrated product **12d**, purple = carboxylic acid product **12c**.

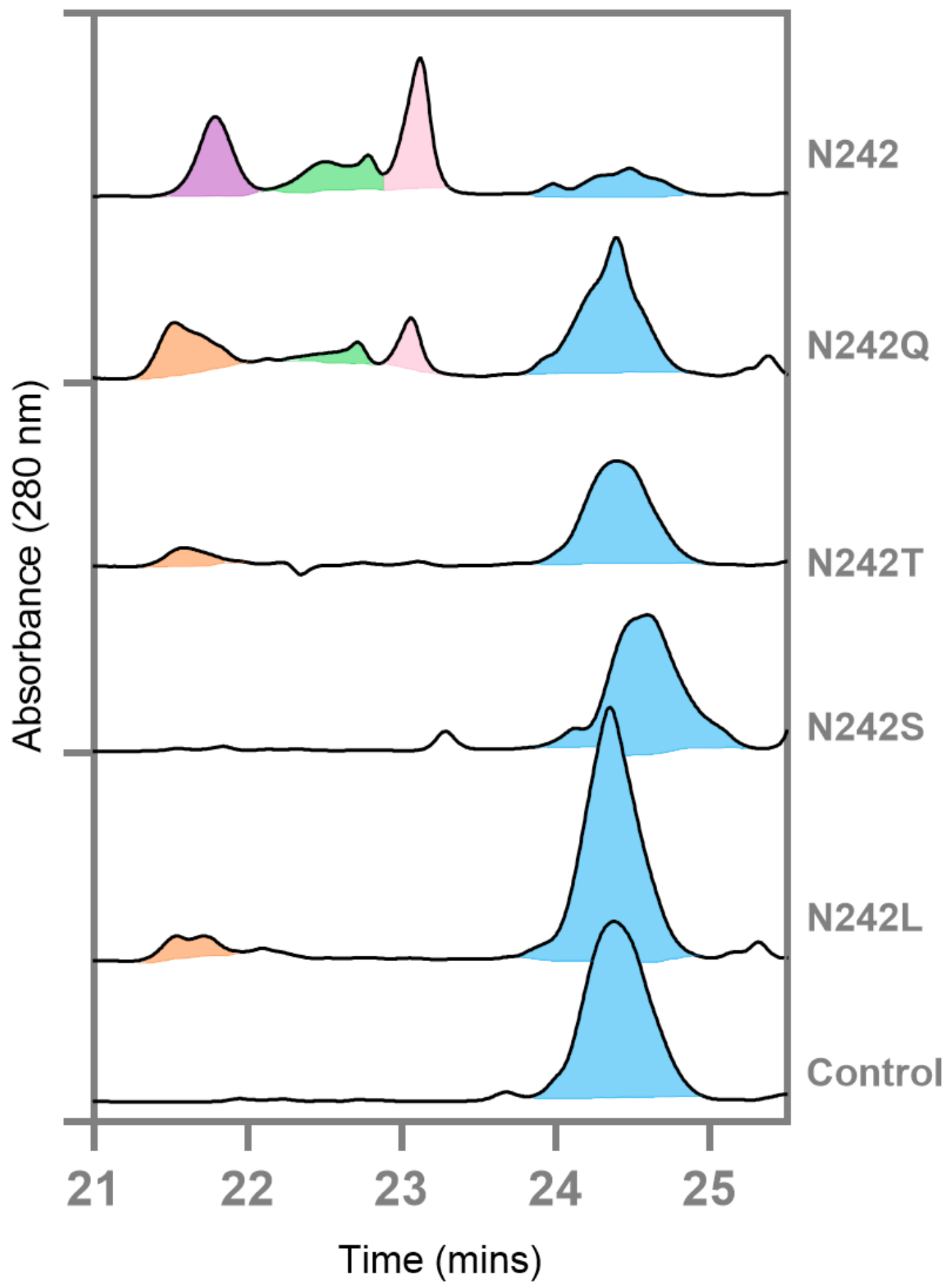

Figure S35 LC-MS assay trace of peptide 11. Traces show N242 and mutants N242Q, N242T, N242S and N242L recorded at absorbance 280 nm. Products are assigned by colour where blue = starting substrate **11**, orange = alcohol product **11a**, green = aldehyde product **11b**/**b***, pink = dehydrated product **11d**, purple = carboxylic acid product **11c**

2.8. Determining the identity of the intermediate (12d)

The assay containing peptide **12** and N242 RufM was scaled up and carried out as previously described (section 2.8). Peptide **12** in DMSO (final conc. 50 µM) was added as a substrate. 25 mM Tris buffer pH 8 was added to reach a final volume of 300 µL. Reactions were incubated for 30 minutes at 30 °C with shaking (800 rpm) in a ThermoMixer C (Eppendorf).

Material	Stock	Initial Concentration	Final Concentration	Vol (µL)
RufM	Buffer D	-	20 µM	-
Ferredoxin	0.5 mL buffer D added to vial, in 50 µL aliquots	4 g L^{-1} (45.45 µM)	0.08 g L^{-1}	6 µL

Ferredoxin Red	0.5 mL buffer D added to vial, in 50 µL aliquots	10 U in 1 mL buffer	0.08 U	6 µL
NADPH	2 mg in 50 µL Tris buffer	50 mM	1 mM	6 µL
Rufomycin	12.2 mM in DMSO	-	50 µM	-
DMSO	Used to dissolve rufomycin	100%	2%	-
Tris Buffer	25 mM pH 8	25 mM	25 mM	Up to 300 µL

Table S6: Large scale reaction mix for peptide 12 and N242 RufM.

After 30 minutes, the reaction was divided into two (A. 1/3 of total reaction mixture, B. 2/3 of total reaction mixture). Reaction A was extracted with ethyl acetate (2 × 500 µL), the solvent layer was collected and evaporated to dryness. The solid was resuspended in 50 µL 5:95 water: acetonitrile. Reaction B was extracted with ethyl acetate (2 × 1000 µL), the solvent layer collected and evaporated to dryness. It was then redissolved in degassed methanol (200 µL, 100 µM), $NaBH_4$ in degassed methanol was added to a final volume of 1 mM. The reaction was mixed under nitrogen for 30 minutes. The reaction was halved again, 100 µL was taken and extracted with 50:50 ethyl acetate: water (2 × 500 µL). The solvent layer was evaporated to dryness before being resuspended in 50 µL 5:95 water: acetonitrile (B1, Fig. S36C). The other half of the reaction (total volume 100 µL) was left for another 30 minutes stirring under nitrogen. It was then extracted with 50:50 ethyl acetate: water (2 × 500 µL) and the solvent layer was evaporated to dryness before being resuspended in 50 µL 5:95 water: acetonitrile for LCMS analysis (B2, Fig. S36C).

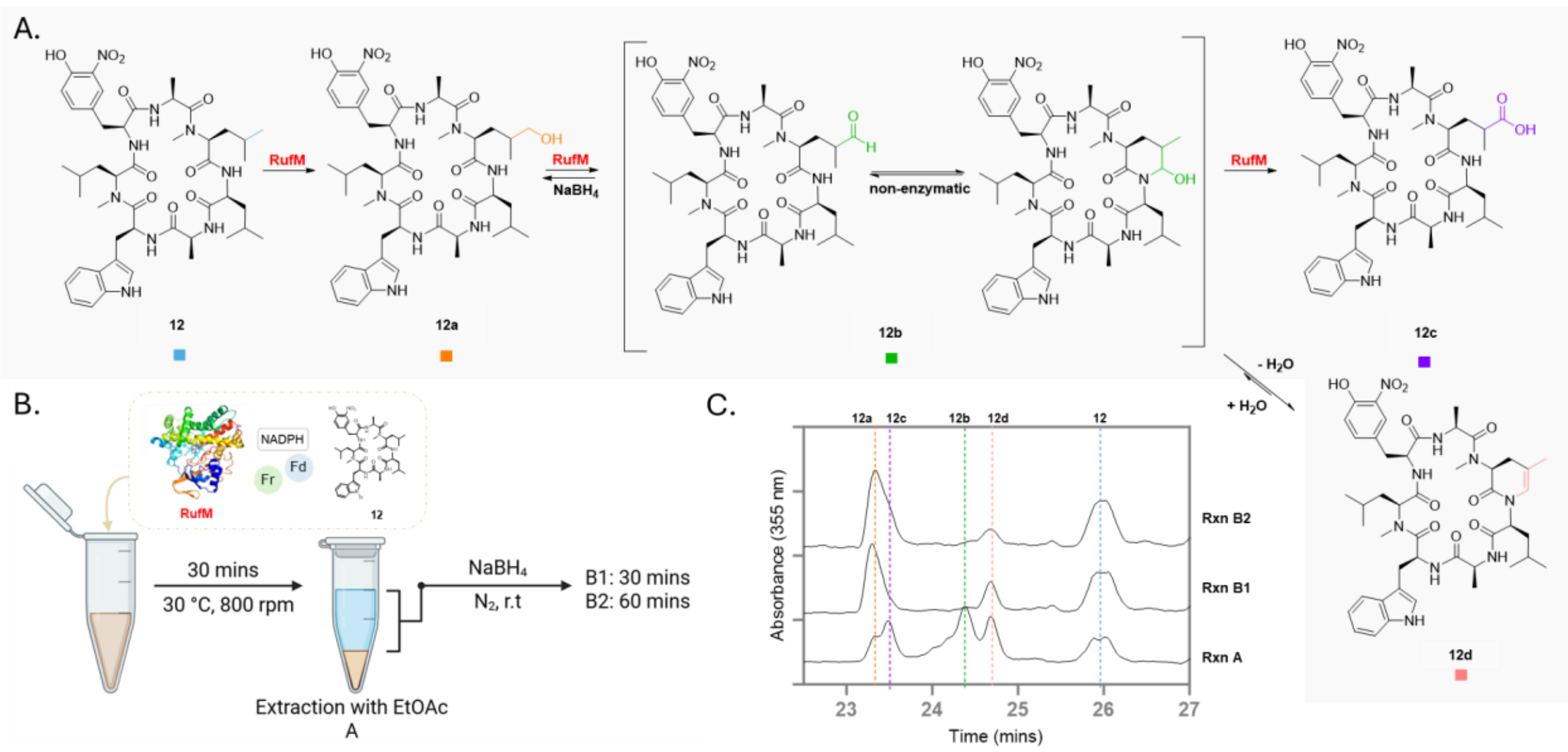

Figure S36: Determining the identity of the intermediates b and d. Peptide **12** (50 µM) assay with N242 RufM (20 µM) and the assay components (See Table S6) at 30°C for 30

minutes (A), following the reaction the peptide was reduced using sodium borohydride (1 mM) for 30 minutes (B1) and for 60 minutes (B2).

2.9. Assessing RufM product inhibition

The enzyme activity inhibition assays were carried with RufM (20 µM), according to heme incorporation, with commercial ferredoxin (final conc. 0.32 g/L) and ferredoxin reductase (final conc. 0.08 U) from *Spinacia oleracea* (Sigma). Peptide **5** in DMSO (final conc. 50 µM) were used as substrate. Tris buffer (25 mM, pH 8) was added to reach a final volume of 400 µL. NADPH (final conc. 1 mM) was added to initiate the reaction. The reactions were incubated for 3 hours at 30 °C with shaking (800 rpm) in a ThermoMixer C (Eppendorf). After 3 hours, the reactions were divided equally to two parts. Both were extracted with ethyl acetate (2 x 500 µL). The solvent layers were collected and evaporated to dryness. The solid of part 1 was resuspended in 100 µL 5:95 water: acetonitrile. The solid of part 2 was suspended in DMSO with same volume as the substrate mentioned above. The rest of reagents were added except substrate to a final volume of 200 µL. the reaction condition was same as the above active assay. After 3 hours, the products were extracted and dried with the same method as above. The solid was resuspended in 100 µL 5:95 water: acetonitrile. The samples were analysed by LC-MS using Gradient 1.

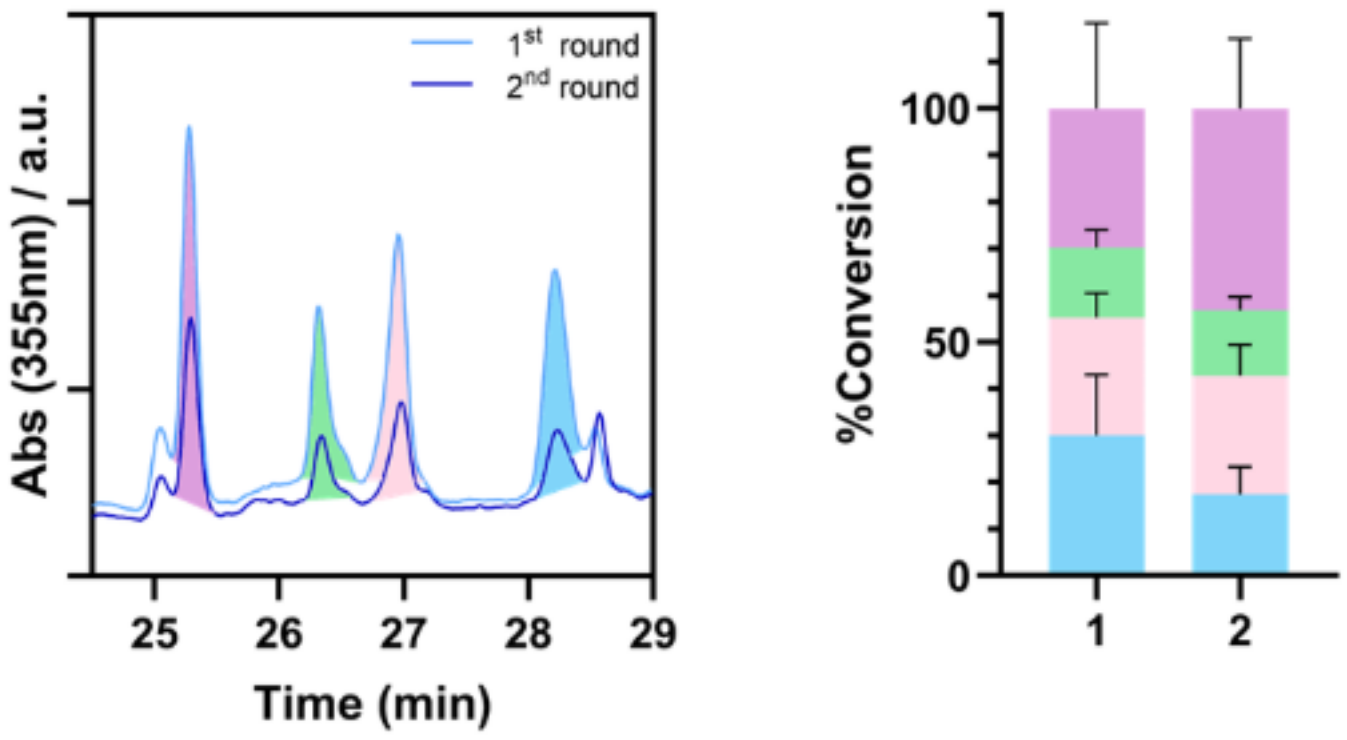

Figure S37: Assessing product inhibition of RufM. LC-MS trace at 355 nm showing products formation by RufM with peptide **5**. Light blue line is the products formation of round 1. Dark blue line is the product formation of round 2. Conversions (average of 3 repeats) were determined using LC-MS peak area at 355 nm. Products are assigned by colour where blue = substrate, green = aldehyde, pink = enamine, purple = carboxylic acid.

2.10. RufM assays: Conversion tables and HRMS of substrates and products.

Wild Type (N242) LC-MS assay conversions											
Peptide	Unmodified	SD	Alcohol	SD	diol	SD	Aldehyde/ aminal	SD	dehydrated	SD	Carboxylic acid	SD
1	53.56	12.04	0	0	0	0	2.30	1.099	3.55	1.60	40.59	9.33
2	72.58	0.48	0	0	0	0	2.60	1.06	0.64	0.06	24.18	1.49
3	69.20	9.62	0	0	0	0	5.79	1.85	2.72	1.71	22.28	6.06
4	54.69	23.46	0	0	0	0	2.41	1.19	2.59	0.87	40.37	21.396
5	40.64	3.83	0	0	0	0	5.64	0.69	6.68	0.50	47.04	2.64
6	34.81	3.28	0	0	0	0	4.14	0.056	3.07	0.49	57.98	3.71
7	86.07	1.99	7.10	0.69	0	0	2.23	0.48	4.59	0.85	0	0
8	100	0	0	0	0	0	0	0	0	0	0	0
9	39.74	2.29	0	0	0	0	11.50	0.75	14.67	0.46	34.09	3.50
10	47.06	1.40	0	0	0	0	8.26	2.96	11.07	3.74	33.61	2.18
11	33.52	15.55	0	0	0	0	11.26	2.30	30.94	4.15	24.28	9.10
12	36.16	6.37	0	0	0	0	7.80	2.12	31.48	9.69	24.56	18.17
13	17.95	0.23	8.57	1.44	11.72	1.63	25.40	2.89	17.51	0.74	18.84	1.15
14	51.00	0.88	11.95	0.31	9.89	1.54	2.77	0.23	2.57	0.49	21.84	0.37
15	66.50	2.53	18.67	1.73	0	0	8.94	0.83	0	0	5.89	0.03
16	87.45	0.73	12.55	0.73	0	0	0	0	0	0	0	0

Table S7: Wild Type (N242) LC-MS assay % conversions taken from HPLC traces at 355nm. Values are average of 3 experiments +/- standard deviation.

Mutant assays conversions with peptide 1												
Mutant	Unmodified	SD	Alcohol	SD	diol	SD	Aldehyde/ aminal	SD	dehydrated	SD	Carboxylic acid	SD
WT	53.56	12.04	0	0	0	0	2.30	1.1	3.55	1.60	40.59	9.33
N242Q	87.58	1.61	4.05	0.48	0	0	1.49	0.17	6.89	0.96	0	0
N242T	97.36	0.81	2.64	0.81	0	0	0	0	0	0	0	0
N242S	100	0	0	0	0	0	0	0	0	0	0	0
N242A	84.08	1.25	6.797	0.041	0	0	2.69	0.15	6.43	1.06	0	0
N242L	75.16	1.16	13.29	0.24	0	0	11.54	1.40	0	0	0	0
N242E	100	0	0	0	0	0	0	0	0	0	0	0
N242D	100	0	0	0	0	0	0	0	0	0	0	0

Table S8: Mutant LC-MS assay % conversions with peptide 1 from HPLC traces at 355nm. Values are average of 3 experiments +/- standard deviation.

Mutant assays conversions with peptide 5												
Mutant	Unmodified	SD	Alcohol	SD	diol	SD	Aldehyde/ aminal	SD	dehydrated	SD	Carboxylic acid	SD
WT	41.11	4.05	0	0	0	0	3.199	0.61	5.77	0.44	49.92	2.999
N242Q	63.56	0.84	27.97	0.58	0	0	6.33	0.20	2.10	0.41	0	0
N242A	70.45	0.74	9.48	1.12	0	0	6.71	1.35	13.37	0.50	0	0
N242L	94.78	0.72	5.18	0.79	0	0	0	0	0	0	0	0
N242E	84.697	3.44	10.55	2.97	0	0	2.81	0.1	2.58	0.37	0	0

Table S9: Mutant LC-MS assay % conversions with peptide 5 from HPLC traces at 355nm. Values are average of 3 experiments +/- standard deviation.

Mutant assays conversions with peptide 12												
Mutant	Unmodified	SD	Alcohol	SD	diol	SD	Aldehyde/ aminal	SD	dehydrated	SD	Carboxylic acid	SD
WT	40.78	0.15	0	0	0	0	8.73	0.804364	20.49	25.23	30.02	25.89
N242Q	63.56	0.84	27.97	0.58	0	0	6.32	0.202441	2.10	0.41	0	0
N242T	97.48	0.07	2.52	0.07	0	0	0	0	0	0	0	0
N242A	73.01	5.17	21.25	2.92	0	0	0.89	0.462296	4.85	1.78	0	0
N242L	89.63	4.33	10.37	4.33	0	0	0	0	0	0	0	0

Table S10: Mutant LC-MS assay % conversions with peptide 12 from HPLC traces at 355nm. Values are average of 3 experiments +/- standard deviation.

Peptide	Acyl hydrazide-peptide				Cyclic peptide			
	Chemical Formula	Calc. (m/z)	Found (m/z)	Error (ppm)	Chemical Formula	Calc. (m/z)	Found (m/z)	Error (ppm)
**3**	$C_{53}H_{70}N_7O_{10}^+$	1028.593	1028.5973	4.18	$C_{53}H_{74}N_9O_{10}^+$	996.5558	996.5538	-2.01
**6**	$C_{48}H_{72}N_{11}O_{10}^+$	962.5464	962.5437	-2.81	$C_{48}H_{68}N_9O_{10}^+$	930.5089	930.5095	0.64
**7**	$C_{46}H_{71}N_{10}O_{10}^+$	923.5355	923.5369	1.52	$C_{46}H_{67}N_8O_{10}^+$	891.4980	891.5040	6.73
**15**	$C_{49}H_{74}N_{11}O_{10}^+$	976.5620	976.5629	0.92	$C_{49}H_{70}N_9O_{10}^+$	944.5240	944.5269	3.07
**16**	$C_{48}H_{72}N_{11}O_{10}^+$	962.5464	962.5437	-2.81	$C_{48}H_{68}N_9O_{10}^+$	930.5189	930.5157	-3.44
**10**	$C_{48}H_{73}N_{10}O_8^+$	917.5613	917.5574	-4.25	$C_{48}H_{69}N_8O_8^+$	885.5239	885.5257	2.03
**9**	$C_{49}H_{75}N_{10}O_8^+$	931.5769	931.5773	0.43	$C_{49}H_{71}N_8O_8^+$	899.5395	899.5389	-0.67
**11**	$C_{46}H_{71}N_{10}O_8^+$	891.5457	891.5467	1.12	$C_{46}H_{67}N_8O_8^+$	859.5076	859.5083	0.81
**17**	$C_{52}H_{72}N_{11}O_{10}^+$	1010.5464	1010.5448	-1.58	$C_{52}H_{68}N_9O_{10}^+$	978.5089	978.5084	0.41
**18**	$C_{48}H_{72}N_{11}O_{10}S^+$	994.5184	994.5174	-1.00	$C_{48}H_{68}N_9O_{10}S^+$	962.4810	962.4804	0.72

Table S11: *HRMS data of synthesised hydrazide peptides and cyclic peptides. All peptide mass listed are reported as* [M+H]$^+$ *and only monoisotopic peaks are listed in the table. All peptide samples are analysed on UPLC-HRMS using Gradient 6. The synthesis of peptides* ***1,2,4,5,8,12,13****,* ***14 17 and 18*** *have been reported previously.*[1]

Peptide	Alcohol Product (a)				Aldehyde/hemiaminal Product (b/b*)			
	Chemical Formula	Calc. (m/z)	Found (m/z)	Error (ppm)	Chemical Formula	Calc. (m/z)	Found (m/z)	Error (ppm)
**1**	$C_{54}H_{78}N_9O_{11}^+$	1028.5820	1028.5829	0.87	$C_{54}H_{76}N_9O_{11}^+$	1026.5664	1026.5621	-4.19
**2**	$C_{53}H_{76}N_9O_{11}^+$	1014.5648	1014.5664	-1.57	$C_{53}H_{74}N_9O_{11}^+$	1012.5508	1012.5515	0.69
**3**	$C_{53}H_{74}N_9O_{11}^+$	1012.5502	–	–	$C_{53}H_{72}N_9O_{11}^+$	1010.5346	1010.5314	-3.76
**4**	$C_{57}H_{78}N_9O_{11}^+$	1064.5820	1064.5819	-0.09	$C_{57}H_{76}N_9O_{11}^+$	1062.5664	1062.5663	-0.094
**5**	$C_{49}H_{70}N_9O_{11}^+$	960.5195	960.5193	-0.21	$C_{49}H_{68}N_9O_{11}^+$	958.5038	958.5035	-0.31
**6**	$C_{48}H_{68}N_9O_{11}^+$	946.5033	–	–	$C_{48}H_{66}N_9O_{11}^+$	944.4882	944.4892	1.03
**7**	$C_{46}H_{67}N_8O_{11}^+$	907.4929	907.4929	0.00	$C_{46}H_{65}N_8O_{11}^+$	905.4773	905.4780	0.77
**8**	$C_{44}H_{65}N_8O_{11}^+$	881.4767	–	–	$C_{44}H_{63}N_8O_{11}^+$	879.4611	–	–
**12**	$C_{46}H_{66}N_9O_{11}^+$	920.4882	920.4857	-2.72	$C_{46}H_{64}N_9O_{11}^+$	918.4725	918.4751	2.83
**15**	$C_{49}H_{70}N_9O_{11}^+$	960.5195	960.5193	0.21	$C_{49}H_{68}N_9O_{11}^+$	958.5038	958.5035	-0.31
**16**	$C_{48}H_{68}N_9O_{11}^+$	946.5038	946.5029	-0.95	$C_{48}H_{66}N_9O_{11}^+$	957.4960	–	–

**13**	$C_{48}H_{68}N_{9}O_{12}^{+}$	962.4987	962.4993	0.62	$C_{48}H_{66}N_{9}O_{12}^{+}$	960.4831	960.4831	0.00
**14**	$C_{49}H_{70}N_{9}O_{12}^{+}$	976.5144	976.5117	-2.76	$C_{49}H_{67}N_{9}O_{12}$	974.4987	974.4983	-0.41
**10**	$C_{48}H_{69}N_{8}O_{9}^{+}$	901.5187	901.5165	-2.44	$C_{48}H_{67}N_{8}O_{9}^{+}$	899.5031	899.5021	-1.11
**9**	$C_{49}H_{71}N_{8}O_{9}^{+}$	–	–	–	$C_{49}H_{69}N_{8}O_{9}^{+}$	913.5187	913.5200	1.42
**11**	$C_{46}H_{67}N_{8}O_{9}^{+}$	875.5026	875.4996	-3.43	$C_{46}H_{65}N_{8}O_{9}^{+}$	873.4874	873.4843	-3.55
Peptide	Dehydrated product (d)				Carboxylic acid product (c)			
	Chemical Formula	Calc. (m/z)	Found (m/z)	Error (ppm)	Chemical Formula	Calc. (m/z)	Found (m/z)	Error (ppm)
**1**	$C_{54}H_{74}N_{9}O_{10}^{+}$	1008.5558	1008.5558	0.0000	$C_{54}H_{76}N_{9}O_{12}^{+}$	1042.5614	1042.5614	0.0000
**2**	$C_{53}H_{72}N_{9}O_{10}^{+}$	994.5402	994.5347	-5.53	$C_{53}H_{74}N_{9}O_{12}^{+}$	1028.5457	1028.5433	-2.33
**3**	$C_{53}H_{70}N_{9}O_{10}^{+}$	992.5245	992.5201	-4.43	$C_{53}H_{72}N_{9}O_{12}^{+}$	1026.5300	1026.5265	-3.41
**4**	$C_{57}H_{74}N_{9}O_{10}^{+}$	1044.5559	1044.5514	-4.31	$C_{57}H_{76}N_{9}O_{12}^{+}$	1078.5614	1078.5564	-4.63
**5**	$C_{49}H_{66}N_{9}O_{10}^{+}$	–	–	–	$C_{49}H_{68}N_{9}O_{12}^{+}$	974.4987	974.4983	-0.41
**6**	$C_{48}H_{64}N_{9}O_{10}^{+}$	926.4776	926.4748	-3.02	$C_{48}H_{66}N_{9}O_{12}^{+}$	960.4831	960.4813	-1.93

**7**	$C_{46}H_{63}N_8O_{10}^+$	887.4667	887.4695	3.16	$C_{46}H_{65}N_8O_{12}^+$	921.4716	–	–
**8**	$C_{44}H_{61}N_8O_{10}^+$	861.4505	–	–	$C_{44}H_{63}N_8O_{12}^+$	895.4560	–	–
**12**	$C_{46}H_{61}N_9O_{10}^+$	900.4620	900.4599	-2.33	$C_{46}H_{64}N_9O_{12}^+$	934.4675	934.4653	-2.35
**15**	–	–	–	–	$C_{49}H_{68}N_9O_{12}^+$	974.4987	974.4963	-2.46
**16**	$C_{48}H_{64}N_9O_{10}^+$	973.4909	–	–	$C_{48}H_{66}N_9O_{12}^+$	975.5066	–	–
**13**	$C_{48}H_{64}N_9O_{11}^+$	942.4725	942.4713	-1.27	$C_{48}H_{66}N_9O_{13}^+$	976.4780	976.4797	1.74
**14**	$C_{49}H_{66}N_9O_{11}^+$	956.4882	956.4897	1.56	$C_{49}H_{68}N_9O_{13}^+$	990.4937	990.4902	-3.53
**10**	$C_{48}H_{65}N_8O_8^+$	881.4926	881.4926	0.00	$C_{48}H_{67}N_8O_{10}^+$	915.4980	915.4943	-4.04
**9**	$C_{49}H_{67}N_8O_8^+$	895.5082	895.5047	-3.90	$C_{49}H_{69}N_8O_{10}^+$	929.5137	929.5109	-3.01
**11**	$C_{46}H_{63}N_8O_8$+	855.4769	855.4811	4.90	$C_{46}H_{65}N_8O_{10}^+$	889.4824	889.4825	0.11

Peptide	Bis hydroxylated product			
	Chemical Formula	Calc. (m/z)	Found (m/z)	Error (ppm)
**13e**	$C_{48}H_{68}N_9O_{13}^+$	978.4937	978.4931	1.8

**14ef**	$C_{49}H_{70}N_9O_{13}^+$	992.5093	992.5072	-2.12
Peptide	Epoxide products			
	Chemical Formula	Calc. (m/z)	Found (m/z)	Error (ppm)
***epoxy*-2**	$C_{53}H_{76}N_9O_{11}^+$	1014.5664	1014.5648	-1.58
**Epoxy-2a**	$C_{53}H_{76}N_9O_{12}^+$	1030.5614	1030.5593	-2.04
**Epoxy-2b/b***	$C_{53}H_{74}N_9O_{12}^+$	1028.5457	1028.5433	-2.33
**Epoxy-2d**	$C_{53}H_{72}N_9O_{11}^+$	1010.5352	1010.5337	-1.48
**Epoxy-2c**	$C_{53}H_{72}N_9O_{11}^+$	1044.5406	1044.5347	-5.65

Table S12 *HRMS data of products from assay with RufM. All peptide mass listed are reported as $[M+H]^+$ and only monoisotopic peaks are listed in the table. All peptide samples are analysed on UPLC-HRMS using Gradient 6. Dash indicates that species wasn't detected*

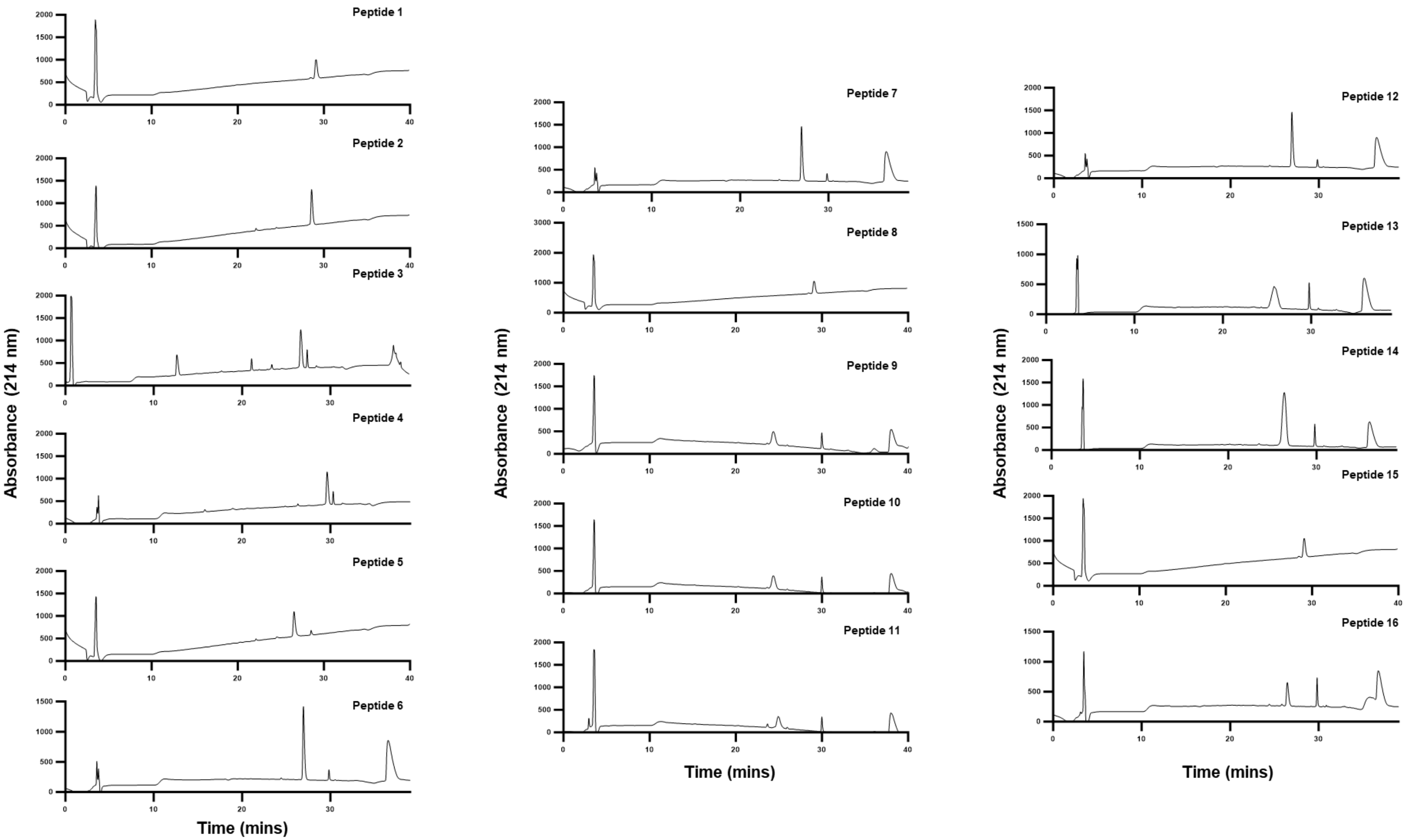
Peptide 1
Peptide 2
Peptide 3
Peptide 4
Peptide 5
Peptide 6
Peptide 7
Peptide 8
Peptide 9
Peptide 10
Peptide 11
Peptide 12
Peptide 13
Peptide 14
Peptide 15
Peptide 16
Absorbance (214 nm)
Absorbance (214 nm)
Absorbance (214 nm)
Time (mins)
Time (mins)
Time (mins)

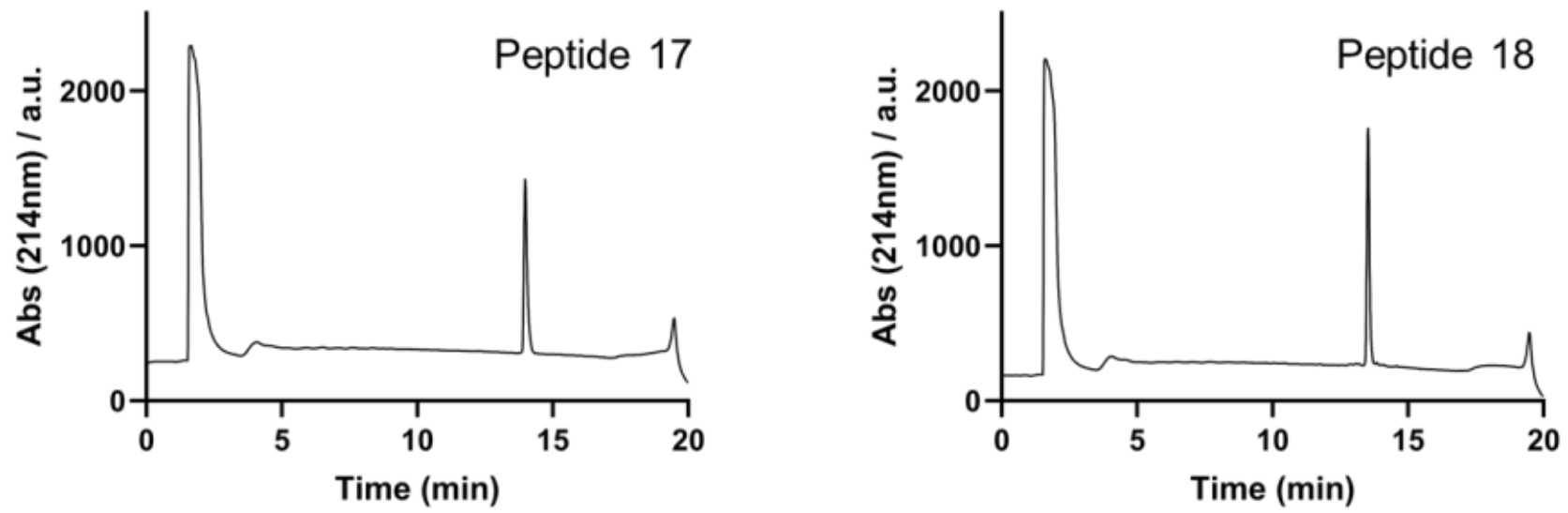

Figure S38: Purity of synthesised cyclic peptides. HPLC traces of peptides 1-18 at absorbance 214 nm using gradient 1. Peaks at 30 &~39 min are HPLC artefacts due to changes in solvent.

3. Computational Modelling

Docking of rufomycin B **1**, into a model of RufM.

3.1. Docking Heme into RufM model

A model of RufM was generated from its protein sequence using AlphaFill,[8] to counter the tendency of protein structures to collapse in the absence of bound ligands. The N-terminal 7 residues had low structural confidence and were truncated for downstream modelling. Bond lengths and angeles were idealized using the Rosetta software package.[9] The heme cofactor was placed using the initial position from AlphaFill and optimized by ligand docking in Rosetta[3] using ligand parameters generated from the AlphaFill ligand coordinates by the molfile_to_params.py script provided with the RosettaLigand software package.[10] The refined heme dock with the best interface score of 10,000 decoys was used for all subsequent docking and remodeling steps.

3.2. Docking rufomycin B, 1a, into heme bound RufM model

The RufB substrate was manually placed near the putative binding site based on homology to TamI (PDB ID: 6XA2).[11] For RufB docking, the low structural confidence loop from residues 70-79 was removed to avoid loop collapse into the substrate binding pocket and rebuilt after ligand docking to accommodate induced fit effects.

Ligand conformation parameters were generated using CREST and xtb[5].[12,13] CREST was used to generate an initial coordinate file of the 3D ligand structure.

```
$ crest RufB_3d.sdf
```

The xtb software package was used to generate an optimized 3D geometry of RufB.

```
$ xtb coord --opt
```

CREST was used to generate a broad ensemble of conformers from the xtb output coordinates.

```
$ crest xtbopt.coord —-input input/conformational_sampling_basic.toml
```

The input file specifies:

```
input='xtbopt.coord'

runtype='imtd-gc'

Threads=64
```

The output conformers were converted in OpenBabel to an sdf file format, which was converted into rosetta parameters using the molfile_to_params.py script provided with the RosettaLigand software package.[9,14]

Small-molecule ligand docking of RufB was run with default docking flags. The top 10% scoring ligand docks by interface score of 50,000 decoys were filtered for the interatomic distance between the gamma carbon of residue 5 on RufB (C15) to the heme iron atom (FE1). Of the 345 filtered pdbs, the docked RufB positions were clustered by the rotameric position of aromatic residue 1. The decoy with the best interface score was selected as the final RufB docked position.

Loop remodeling

The removed loop (residues 70-79) was remodeled using Rosetta loopmodel. 300 decoys were rebuilt around the docked RufB ligand with sidechain repacking to simulate induced fit effects of docking on the flexible loop. The final loop position was selected by loop score and RMSD back to the original AlphaFill model of RufM.

Final heme-bound RufM-RufB complexes were rescored for interface score using RosettaLigand.

Interface scores

RufB docked to RufM WT: -8.478 REU

RufB docked to RufM N242L: -5.297 REU

Images were generated using ChimeraX.[15]

Chimera Acknowledgement: Molecular graphics and analyses performed with UCSF ChimeraX, developed by the Resource for Biocomputing, Visualization, and Informatics at the University of California, San Francisco, with support from National Institutes of Health R01-GM129325 and the Office of Cyber Infrastructure and Computational Biology, National Institute of Allergy and Infectious Diseases.

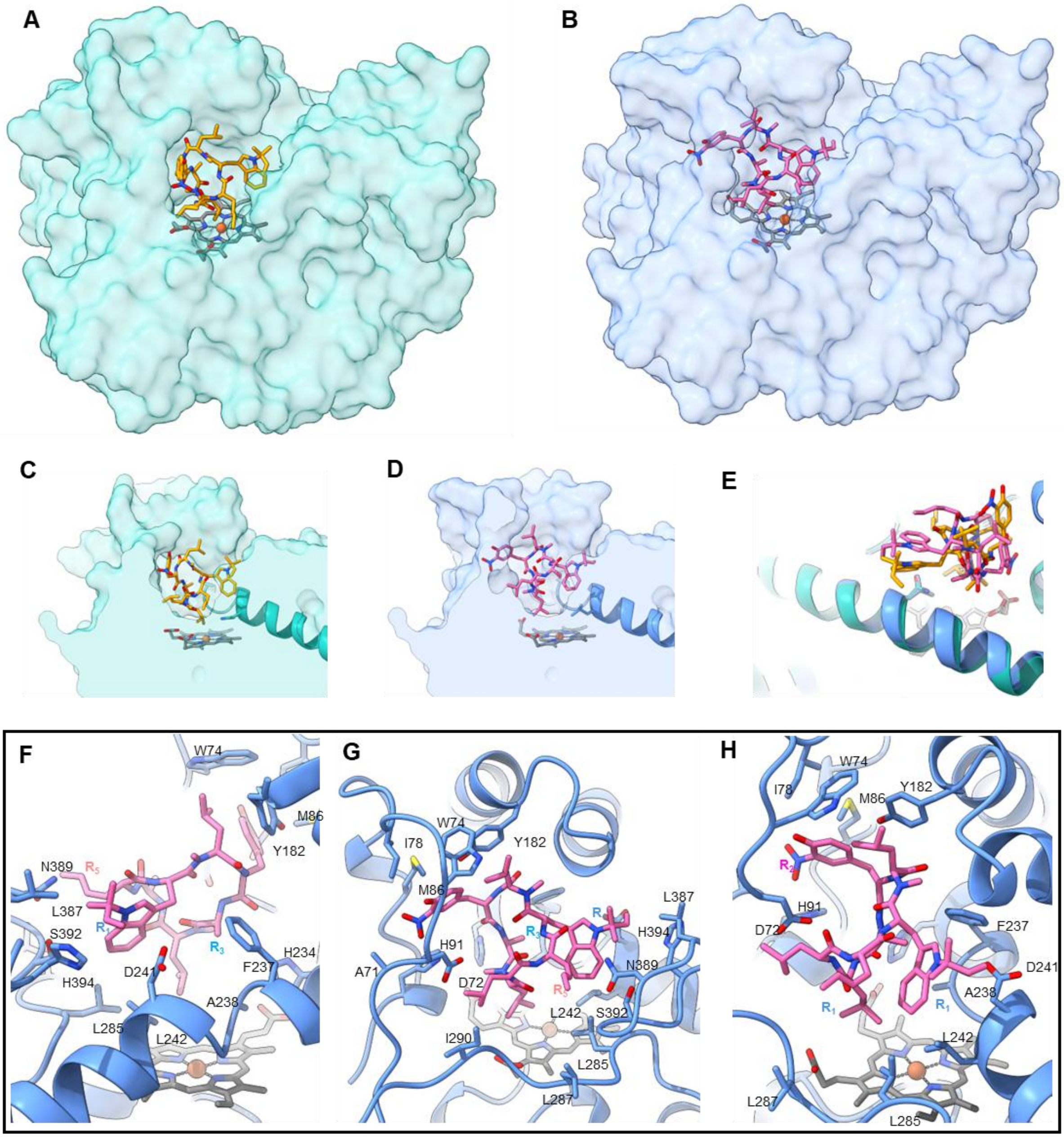

Figure S39: Modelling of RufM N242 and RufM N242L. A) Rufomycin B bound to RufM N242; B) Rufomycin B bound to RufM N242L C) Rufomycin B bound to RufM N242 highlighting heme, I helix and N242 residue; D) Rufomycin B bound to RufM N242L

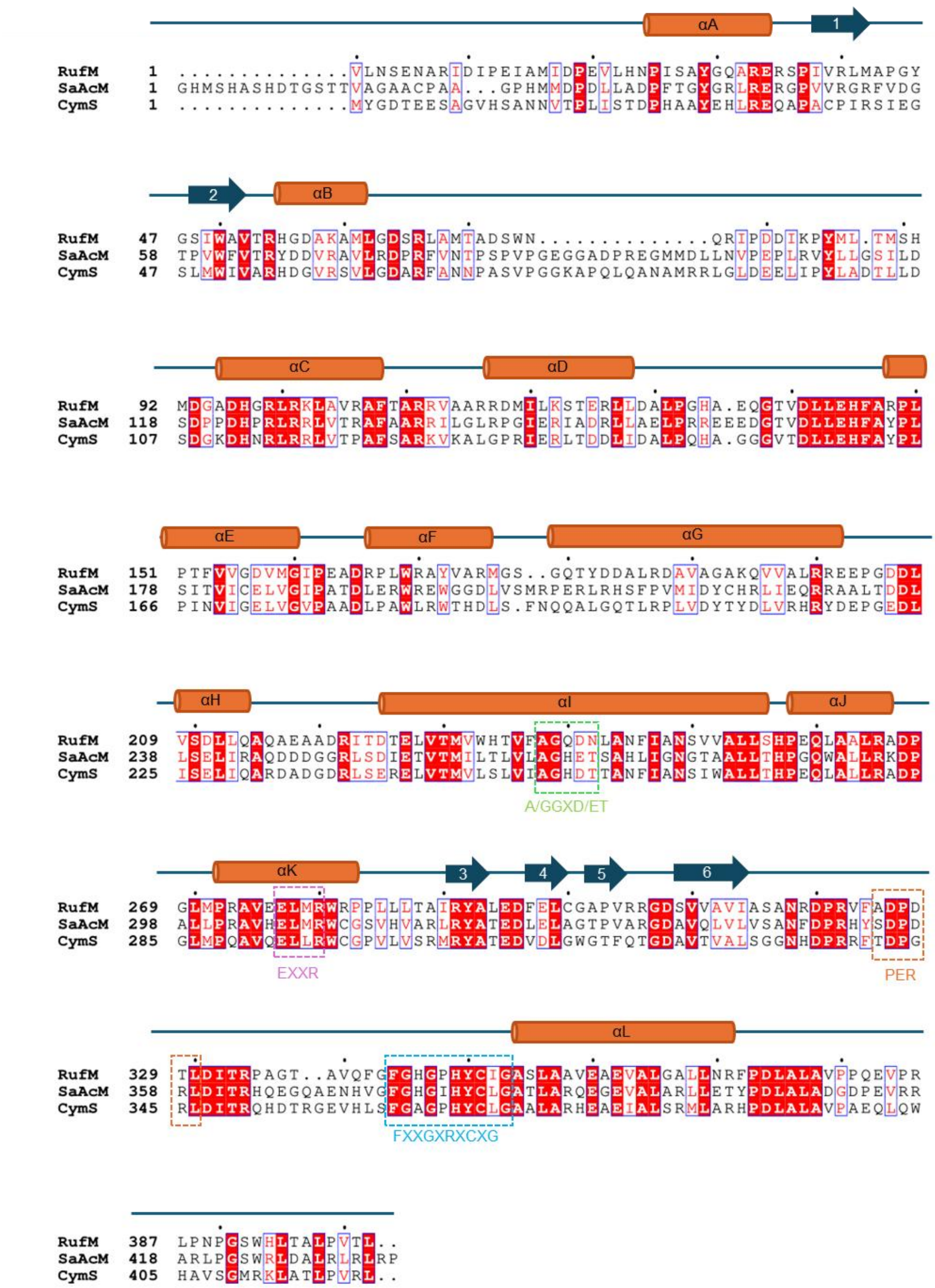

Figure S40: Clustal Omega alignment of RufM with key P450s. P450SaAcm (PDB: 5NWS), P450CymS indicating conserved structural motifs and tertiary structure. Cylinders indicate alpha helices and arrows beta sheets. Line indicates unstructured region.

Testing of Selected Rufomycin Derivatives against *Mtb*

3.3. Microbiological activity of peptides

Minimal inhibitory concentration (MIC) testing was performed using the microdilution method as previously described.[16] Briefly, all compounds were tested against *Mycobacterium tuberculosis* (*Mtb*) reference strain H37Rv (ATCC27294). The initial inoculum was subcultured on Middlebrook 7H10 agar (Becton Dickenson). Colonies were selected within 2 weeks of first observation and a inoculum of 1.5 x10^8 organisms/mL was used for the assay. Initial range finding identified the final range for serial dilutions was from 256μg/mL to 2 μg/mL for each compound and these were tested in Middlebrook 7H9 (Becton Dickenson) broth. All compounds were tested with a final DMSO concentration of 0.5%. Each peptide was tested on 2 separate occasions.

Compound	Initial ranging μg/mL	Test 1 μg/mL	Test 2 μg/mL	MIC μg/mL
5	>8	128	64	128
4	>8	128	64	128
8	>8	32	32	32

Table S13: Reults of MIC testing for peptides 4, 5 and 8

Supplementary References

1 Y. Ding, E. Lambden, J. Peate, L. J. Picken, T. W. Rees, G. Perez-Ortiz, S. A. Newgas, L. A. R. Spicer, T. Hicks, J. Hess, M. B. Ulmschneider, M. M. Müller and S. M. Barry, *J. Am. Chem. Soc.*, 2024, **146**, 16787−16801.

2 A. W. Schultz, D. C. Oh, J. R. Carney, R. T. Williamson, D. W. Udwary, P. R. Jensen, S. J. Gould, W. Fenical and B. S. Moore, *J. Am. Chem. Soc.*, 2008, **130**, 4507–4516.

3 F. W. Studier, *Protein Expr. Purif.*, 2005, **41**, 207–234.

4 B. Sielaff, J. R. Andreesen and T. Schräder, *Appl. Microbiol. Biotechnol.*, 2001, **56**, 458–464.

5 I. R. Phillips and E. A. Shephard, *CYP450 Protocols*, Humana Totowa, NJ, 2006.

6 T. Y. Hargrove, Z. Wawrzak, P. M. Fisher, S. A. Child, W. David Nes, F. Peter Guengerich, M. R. Waterman and G. I. Lepesheva, *Journal of Biological Chemistry*, 2018, **293**, 19344–19353.

7 L. C. Montemiglio, E. Gugole, I. Freda, C. Exertier, L. D'auria, C. G. Chen, A. N. Nardi, G. Cerutti, G. Parisi, M. D'abramo, C. Savino and B. Vallone, *Biomolecules*, 2022, **12**, 55.

8 M. L. Hekkelman, I. de Vries, R. P. Joosten and A. Perrakis, *Nat. Methods*, 2023, **20**, 205–213.

9 J. K. Leman, B. D. Weitzner, S. M. Lewis, J. Adolf-Bryfogle, N. Alam, R. F. Alford, M. Aprahamian, D. Baker, K. A. Barlow, P. Barth, B. Basanta, B. J. Bender, K. Blacklock, J. Bonet, S. E. Boyken, P. Bradley, C. Bystroff, P. Conway, S. Cooper, B. E. Correia, B. Coventry, R. Das, R. M. De Jong, F. DiMaio, L. Dsilva, R. Dunbrack, A. S. Ford, B. Frenz, D. Y. Fu, C. Geniesse, L. Goldschmidt, R. Gowthaman, J. J. Gray, D. Gront, S. Guffy, S. Horowitz, P. S. Huang, T. Huber, T. M. Jacobs, J. R. Jeliazkov, D. K. Johnson, K. Kappel, J. Karanicolas, H. Khakzad, K. R. Khar, S. D. Khare, F. Khatib, A. Khramushin, I. C. King, R. Kleffner, B. Koepnick, T. Kortemme, G. Kuenze, B. Kuhlman, D. Kuroda, J. W. Labonte, J. K. Lai, G. Lapidoth, A. Leaver-Fay, S. Lindert, T. Linsky, N. London, J. H. Lubin, S. Lyskov, J. Maguire, L. Malmström, E. Marcos, O. Marcu, N. A. Marze, J. Meiler, R. Moretti, V. K. Mulligan, S. Nerli, C. Norn, S. Ó'Conchúir, N. Ollikainen, S. Ovchinnikov, M. S. Pacella, X. Pan, H. Park, R. E. Pavlovicz, M. Pethe, B. G. Pierce, K. B. Pilla, B. Raveh, P. D. Renfrew, S. S. R. Burman, A. Rubenstein, M. F. Sauer, A. Scheck, W. Schief, O. Schueler-Furman, Y. Sedan, A. M. Sevy, N. G. Sgourakis, L. Shi, J. B. Siegel, D. A. Silva, S. Smith, Y. Song, A. Stein, M. Szegedy, F. D. Teets, S. B. Thyme, R. Y. R. Wang, A. Watkins, L. Zimmerman and R. Bonneau, *Nat. Methods*, 2020, **17**, 665–680.

10 G. Lemmon and J. Meiler, *Methods in Molecular Biology*, 2012, **819**, 143–155.

11 S. A. Newmister, K. R. Srivastava, R. V. Espinoza, K. Caddell Haatveit, Y. Khatri, R. M. Martini, M. Garcia-Borràs, L. M. Podust, K. N. Houk and D. H. Sherman, *ACS Catal.*, 2020, **10**, 13445–13454.

12 P. Pracht, S. Grimme, C. Bannwarth, F. Bohle, S. Ehlert, G. Feldmann, J. Gorges, M. Müller, T. Neudecker, C. Plett, S. Spicher, P. Steinbach, P. A. Wesołowski and F. Zeller, *J. Chem. Phys.*, 2024, **160**, 114110.

13 C. Bannwarth, E. Caldeweyher, S. Ehlert, A. Hansen, P. Pracht, J. Seibert, S. Spicher and S. Grimme, *Wiley Interdiscip. Rev. Comput. Mol. Sci.*, 2021, **11**, 1493.

14 N. M. O'Boyle, M. Banck, C. A. James, C. Morley, T. Vandermeersch and G. R. Hutchison, *J. Cheminform.*, 2011, **3**, 33.

15 E. C. Meng, T. D. Goddard, E. F. Pettersen, G. S. Couch, Z. J. Pearson, J. H. Morris and T. E. Ferrin, *Protein Science*, 2023, **32**, 32.

16 K. J. Seung, S. Keshavjee and M. L. Rich, *Cold Spring Harb. Perspect. Med.*, 2015, **5**, a017863.

Supplementary Characterization Data

NMR spectra were recorded on a Bruker Avance DRX 400 MHz NMR spectrometer (Ultrashield magnet) and a 700 MHz NMR spectrometer (16.4T superconducting magnet, quadruple resonance ($^{1}H/^{13}C/^{15}N$ cryoprobe with z-gradients and a Bruker Advance III console).

*^{1}H NMR spectrum of rufomycin analogue **3** in CD_3OD at 700 MHz.*

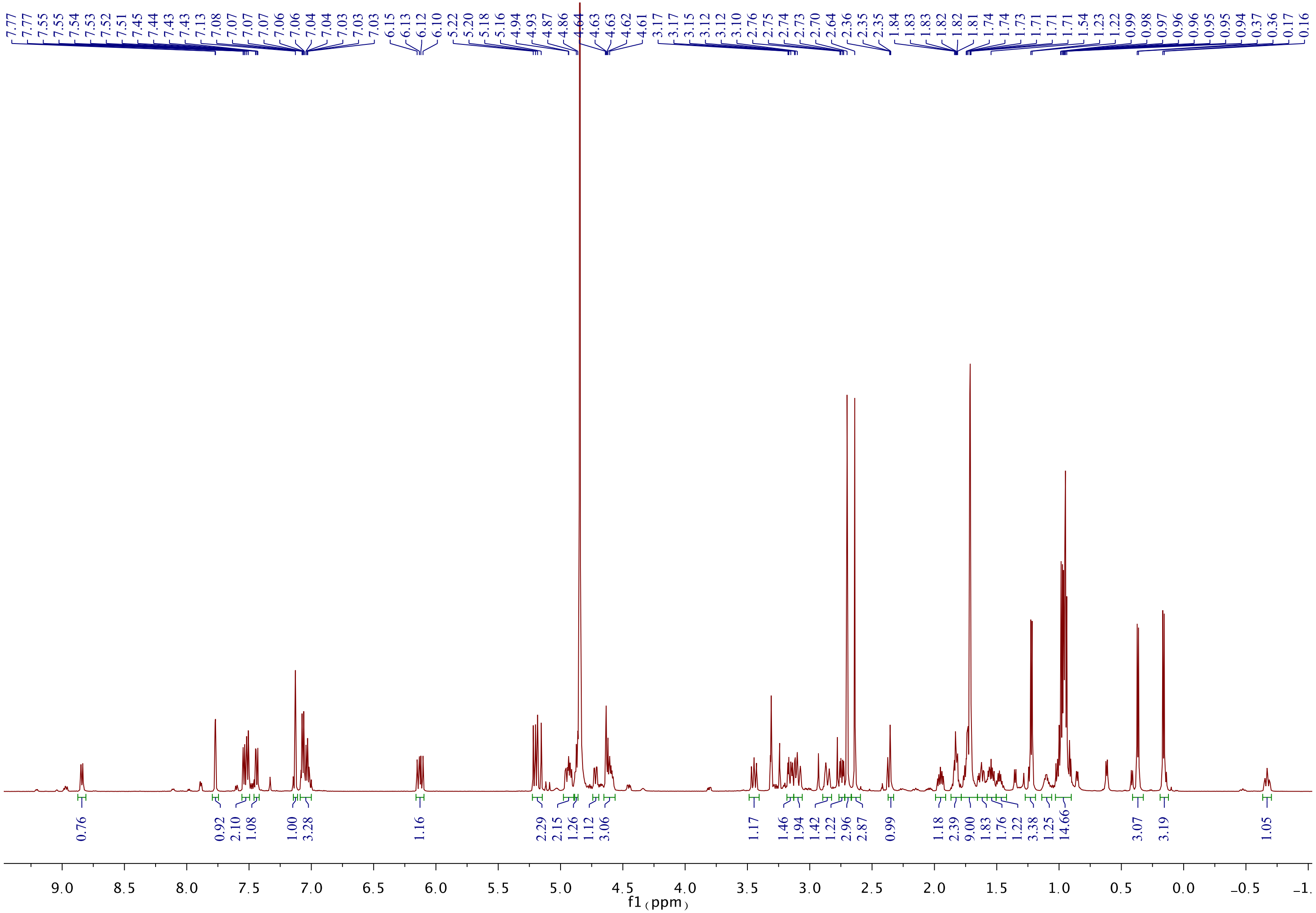

^{13}C NMR spectrum of rufomycin analogue **3** in CD_3OD at 176 MHz.

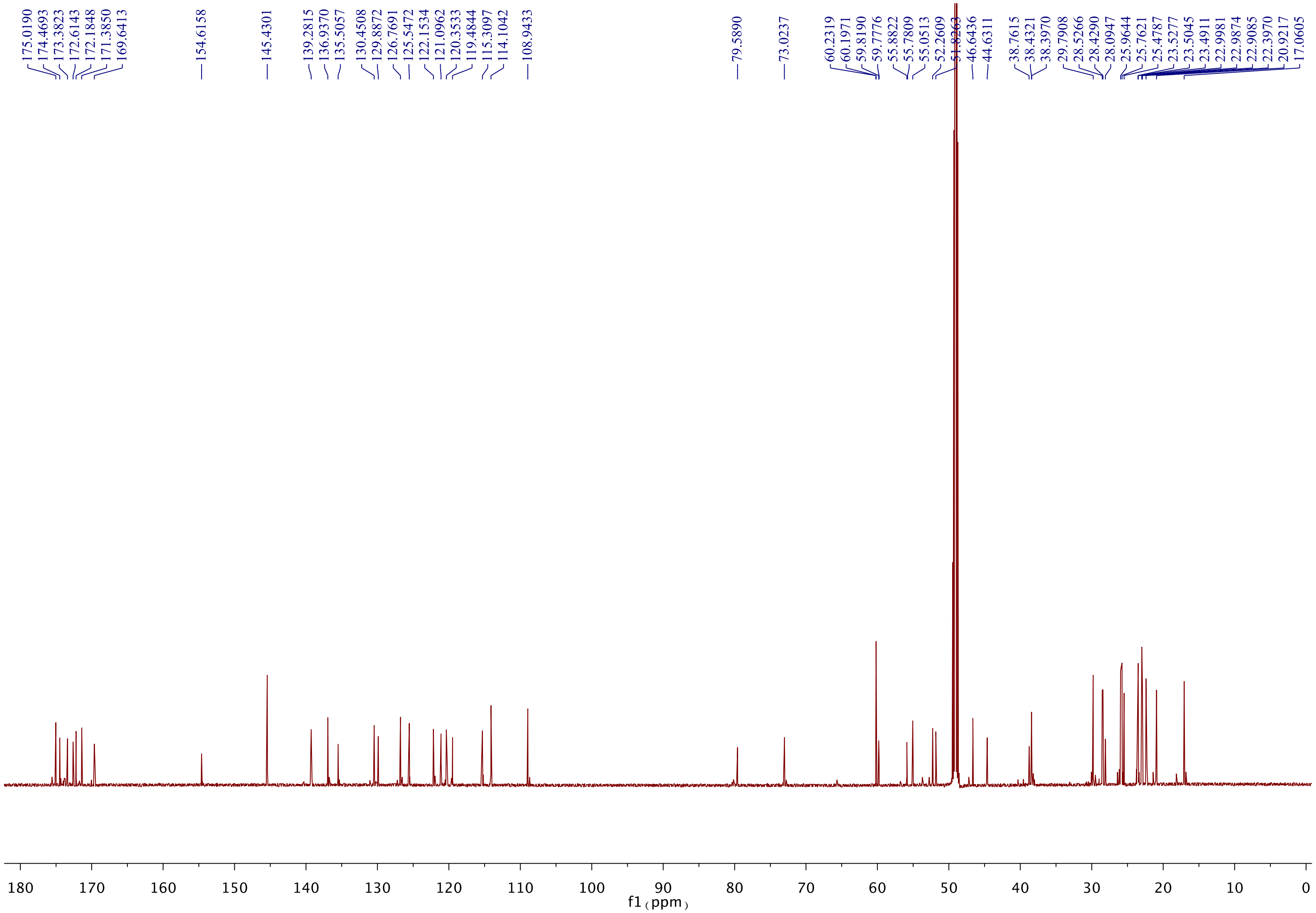

*COSY spectrum of rufomycin analogue **3** in CD_3OD.*

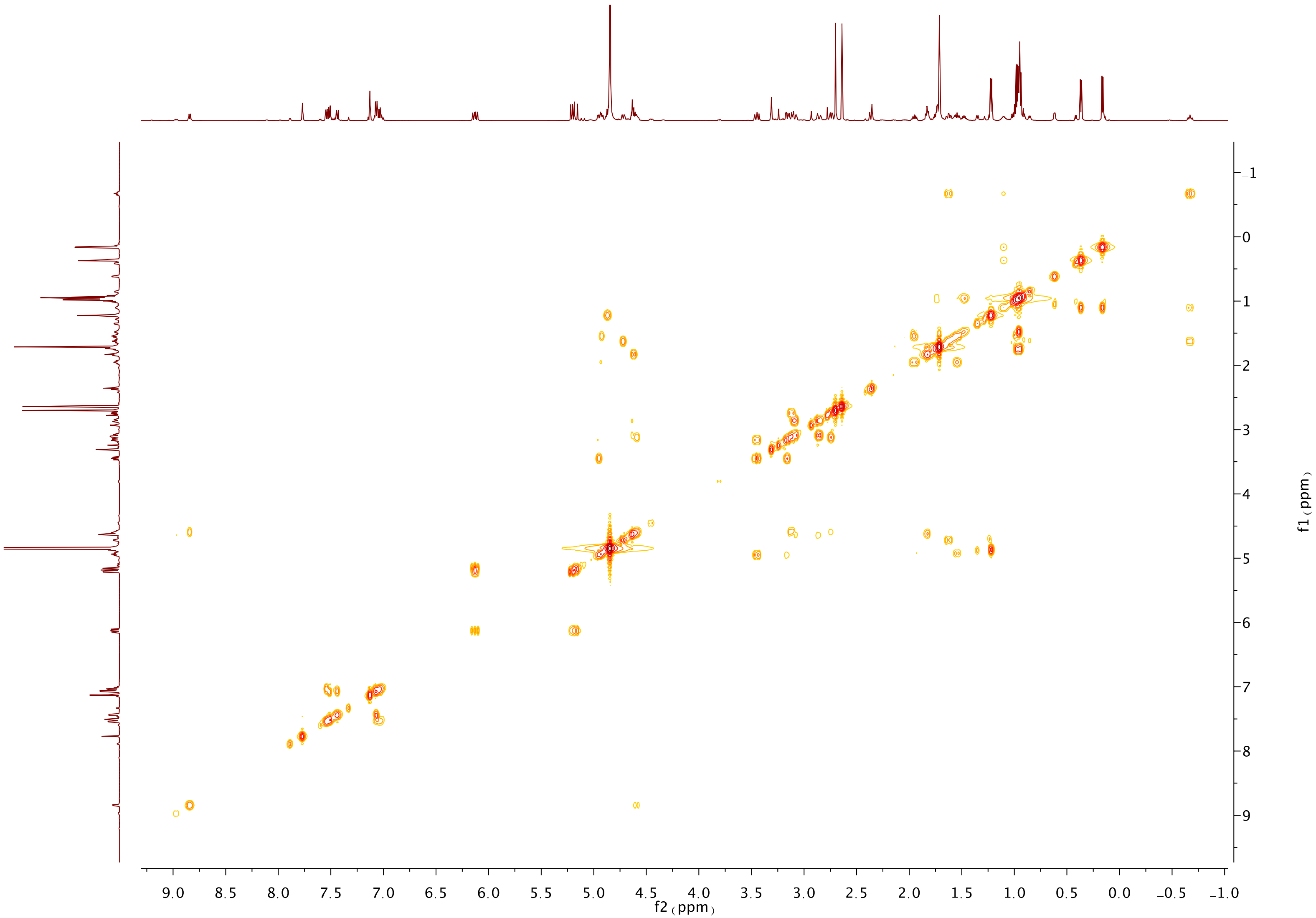

*HSQC spectrum of rufomycin analogue **3** in CD_3OD.*

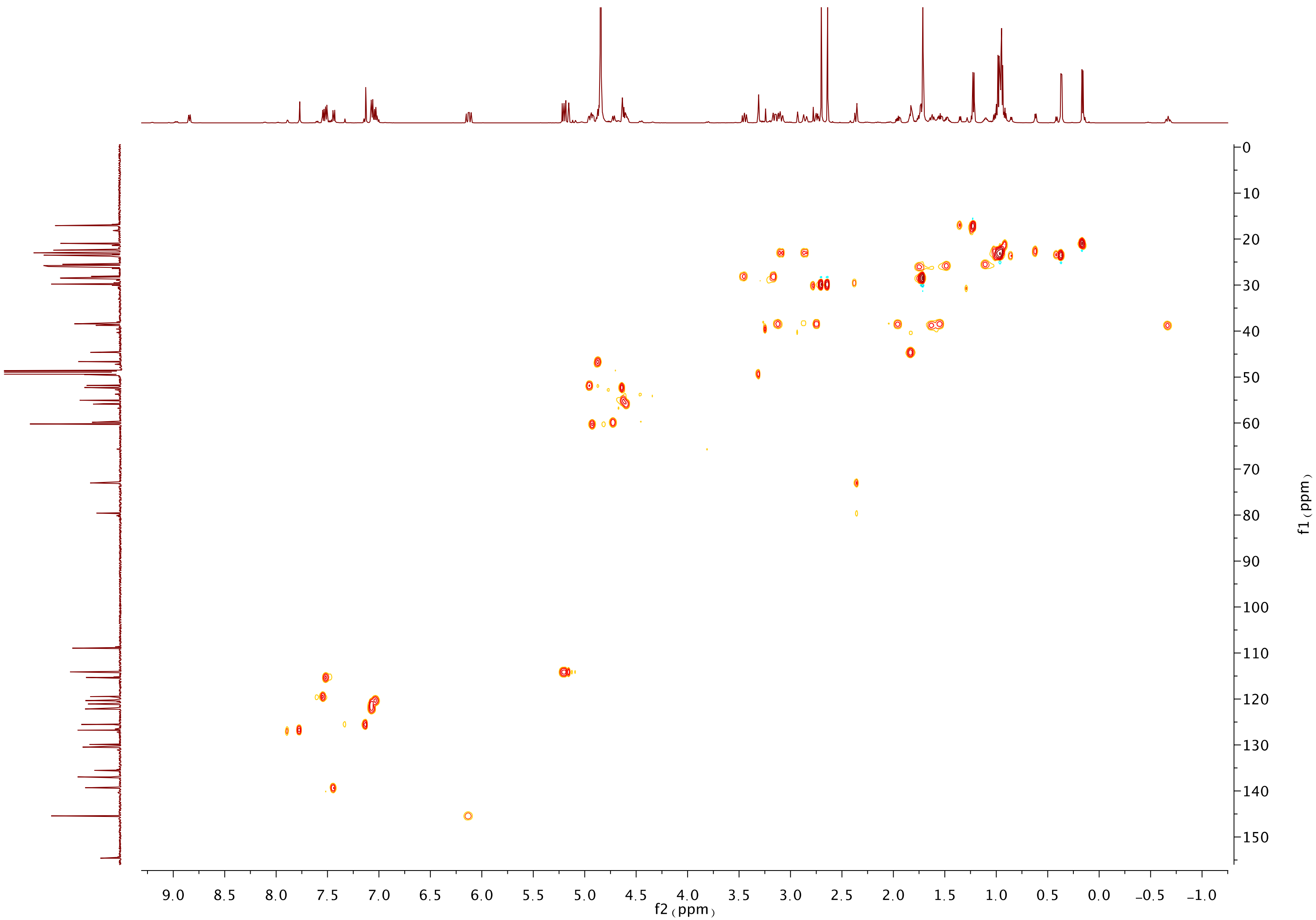

*HMBC spectrum of rufomycin analogue **3** in CD_3OD.*

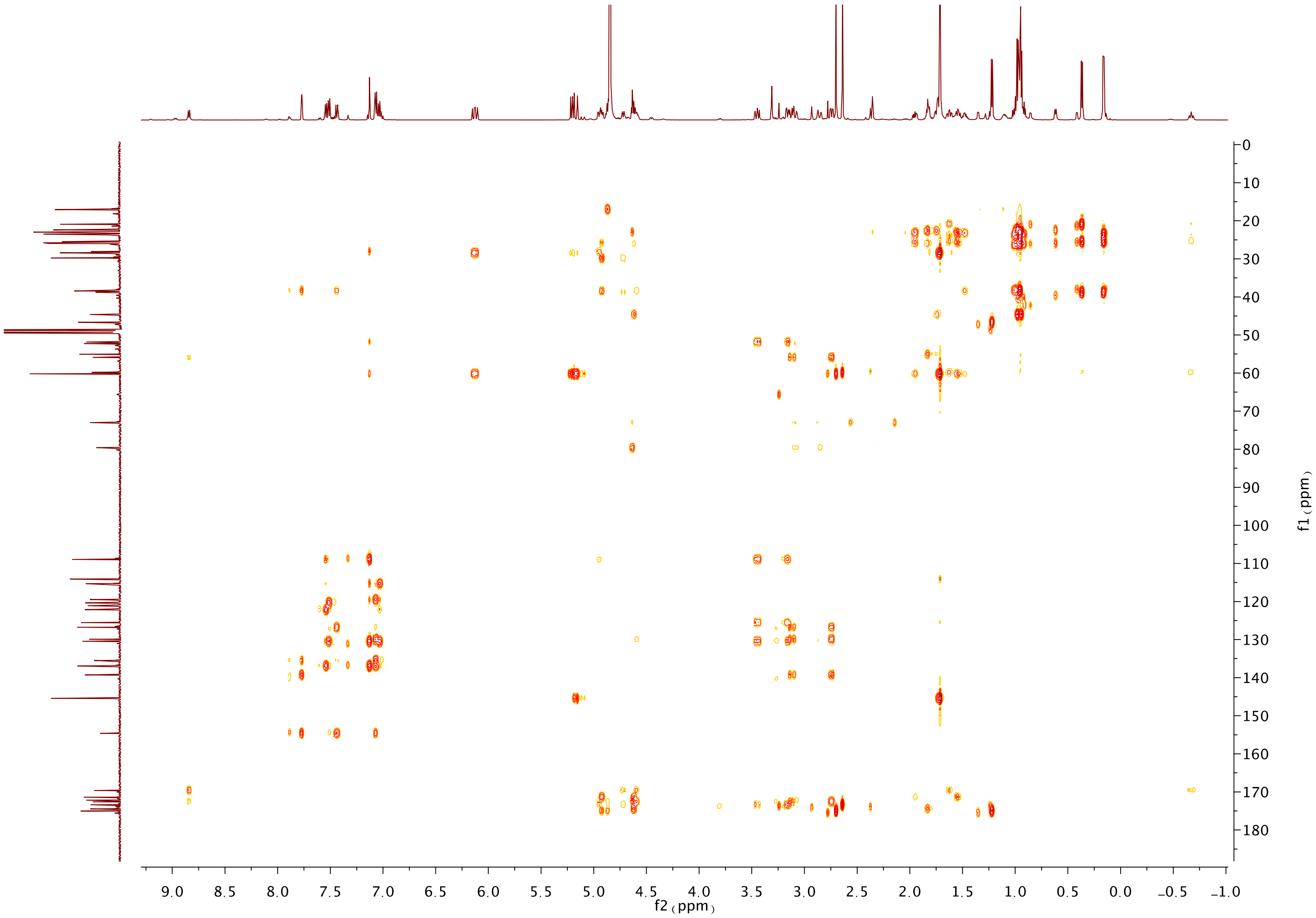

*^{1}H NMR spectrum of rufomycin analogue **6** in CD_3OD at 700 MHz.*

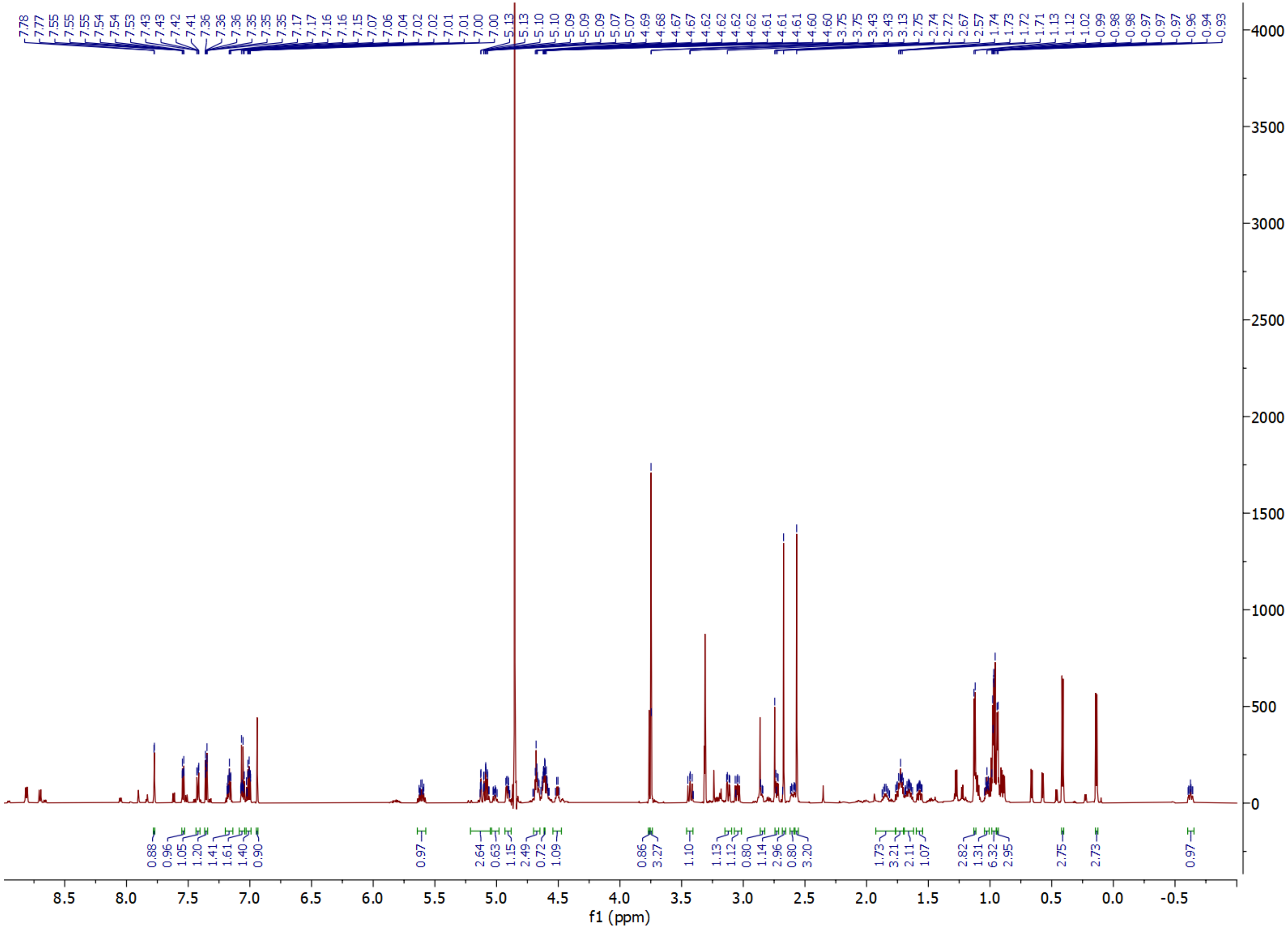

^{13}C NMR spectrum of rufomycin analogue **6** in CD_3OD at 176 MHz.

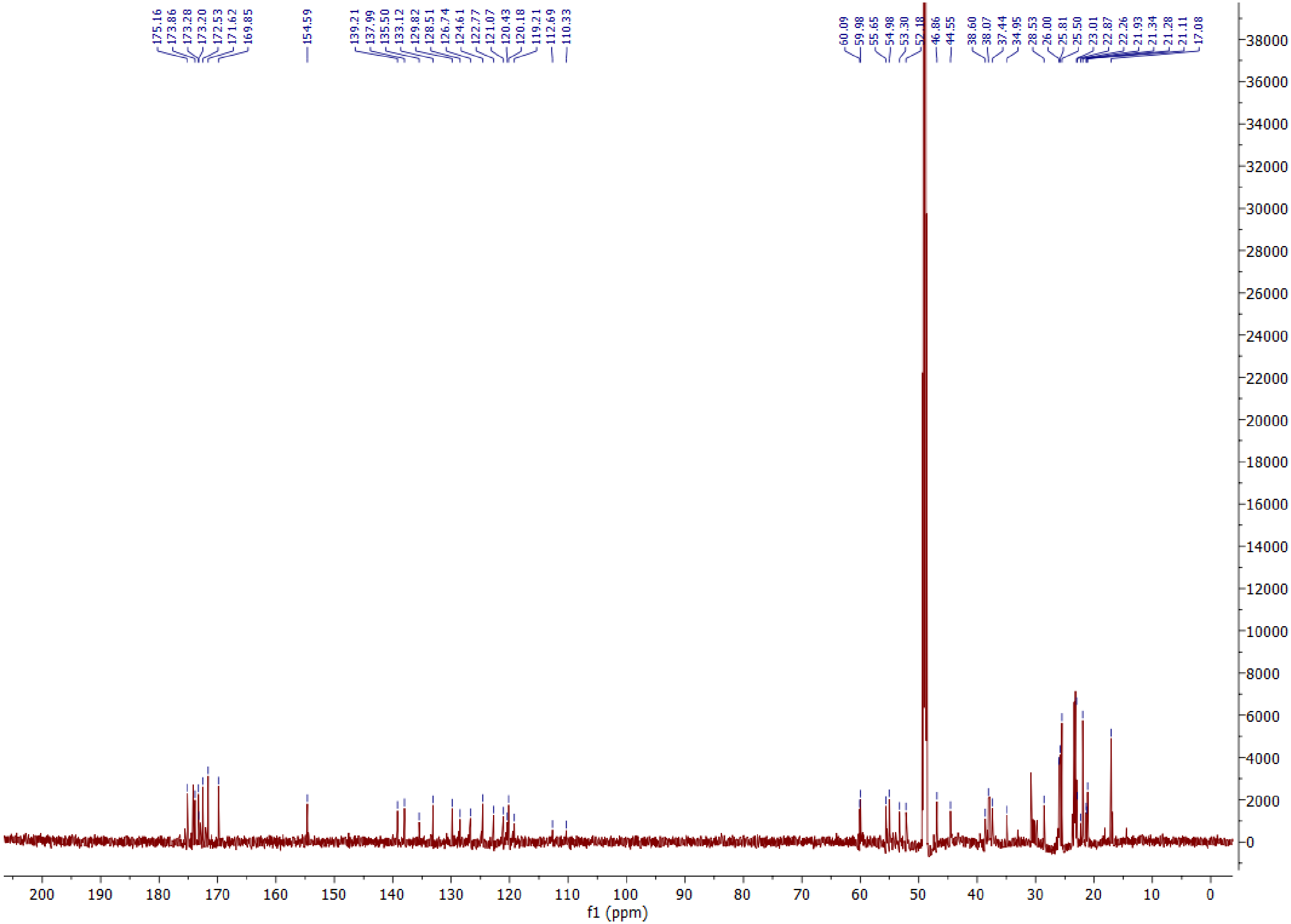

*COSY spectrum of rufomycin analogue **6** in CD_3OD.*

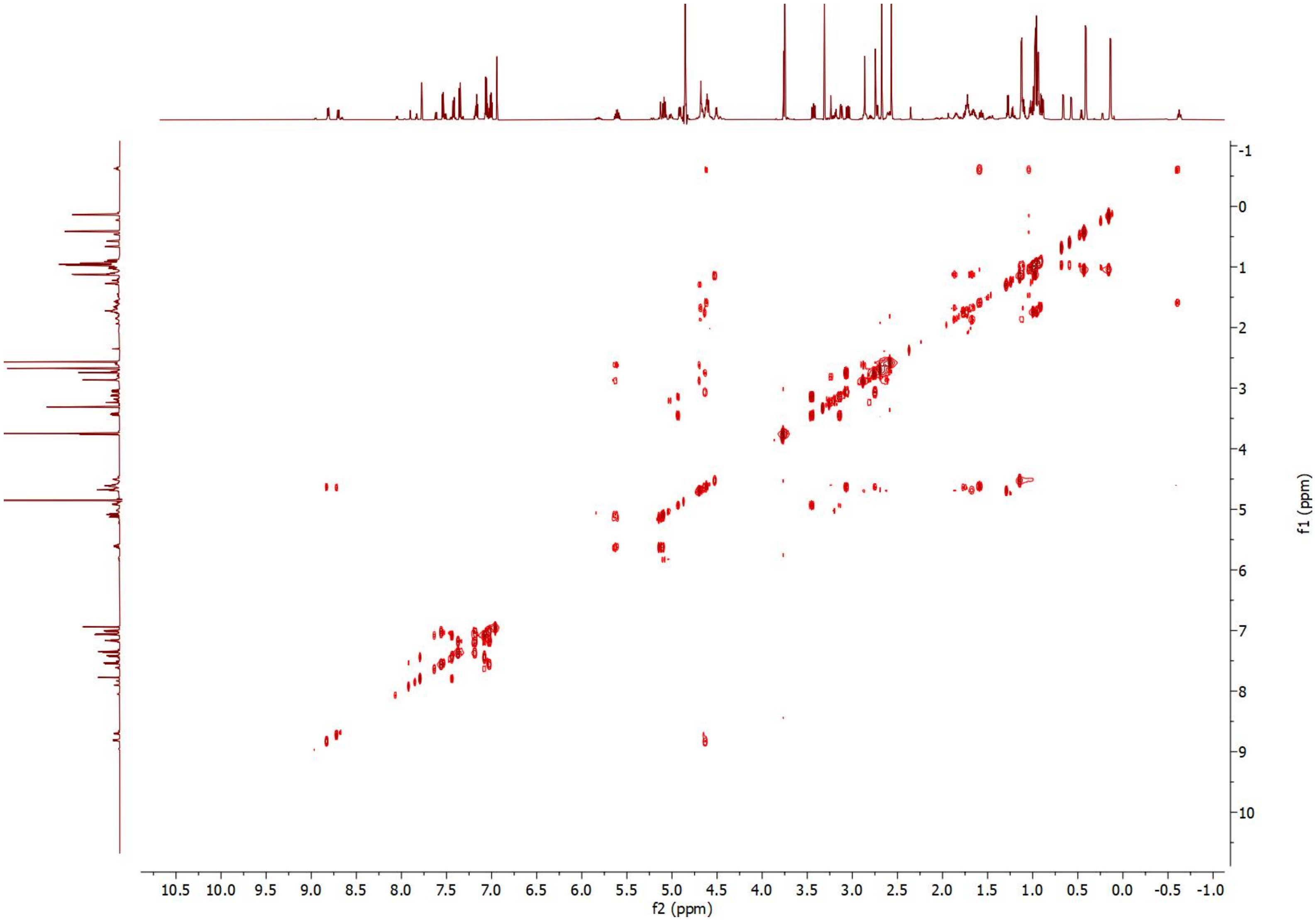

*HSQC spectrum of rufomycin analogue **6** in CD_3OD.*

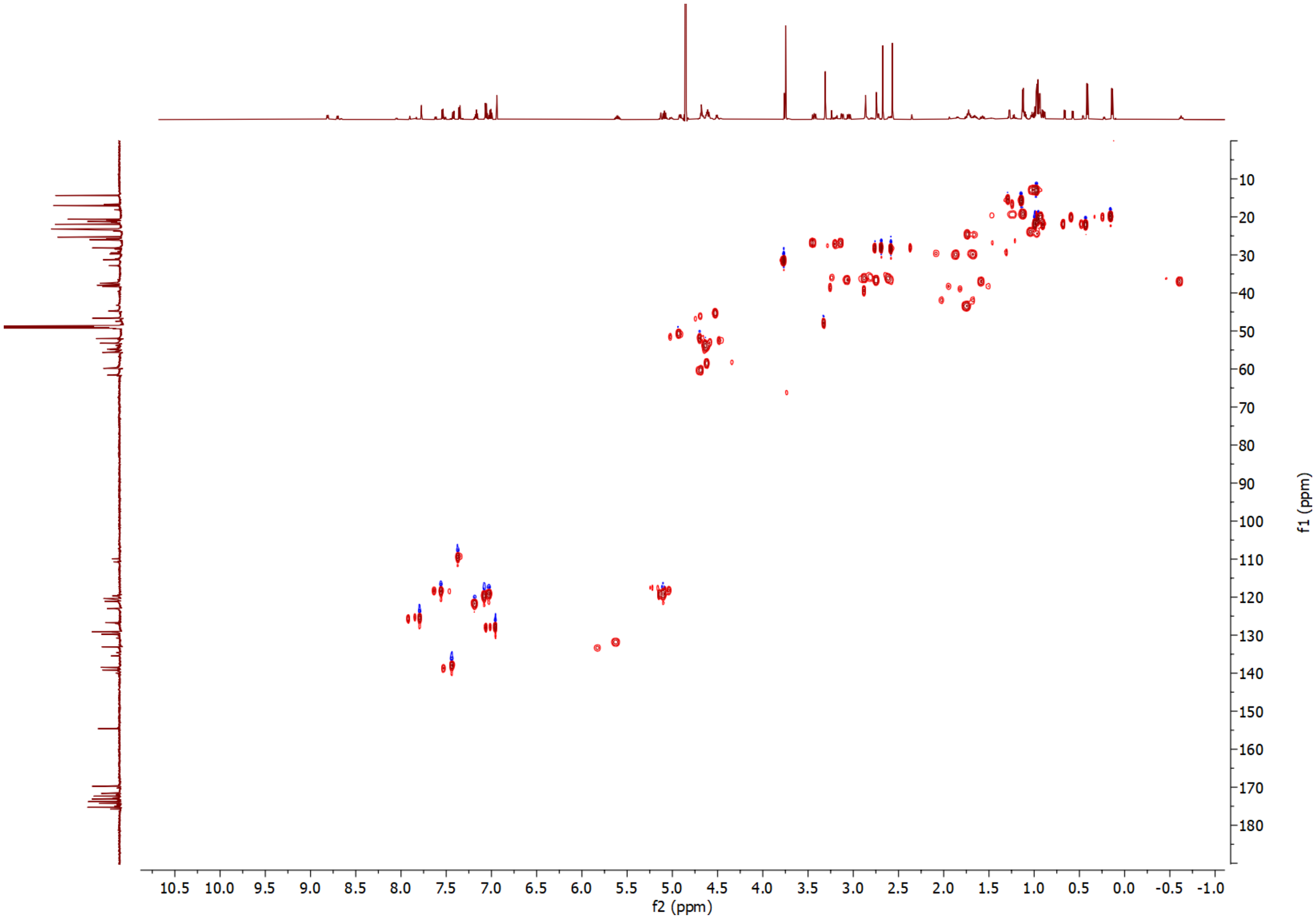

*HMBC spectrum of rufomycin analogue **6** in CD_3OD*

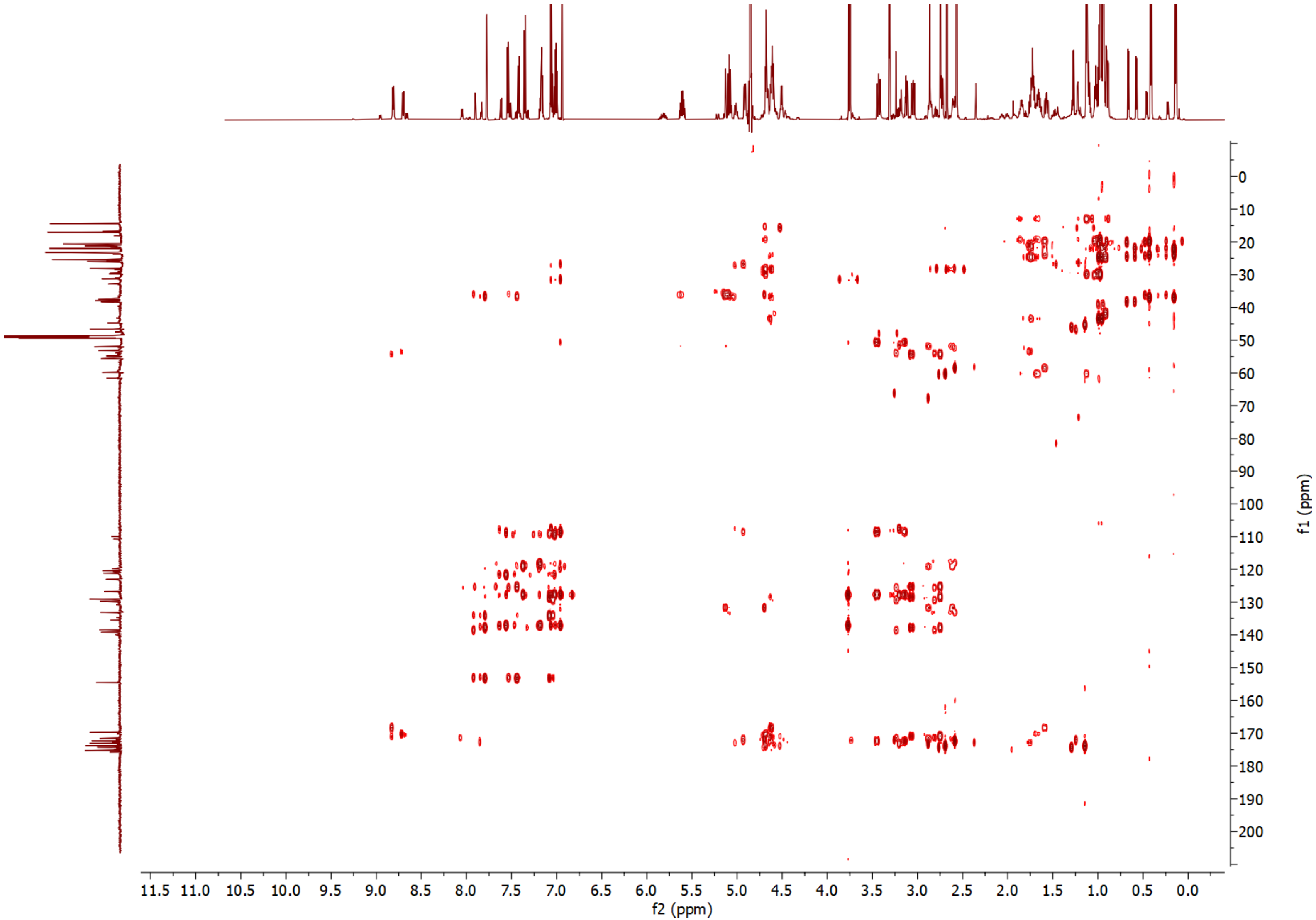

^{1}H NMR spectrum of rufomycin analogue **7** in CD_3OD at 700 MHz.

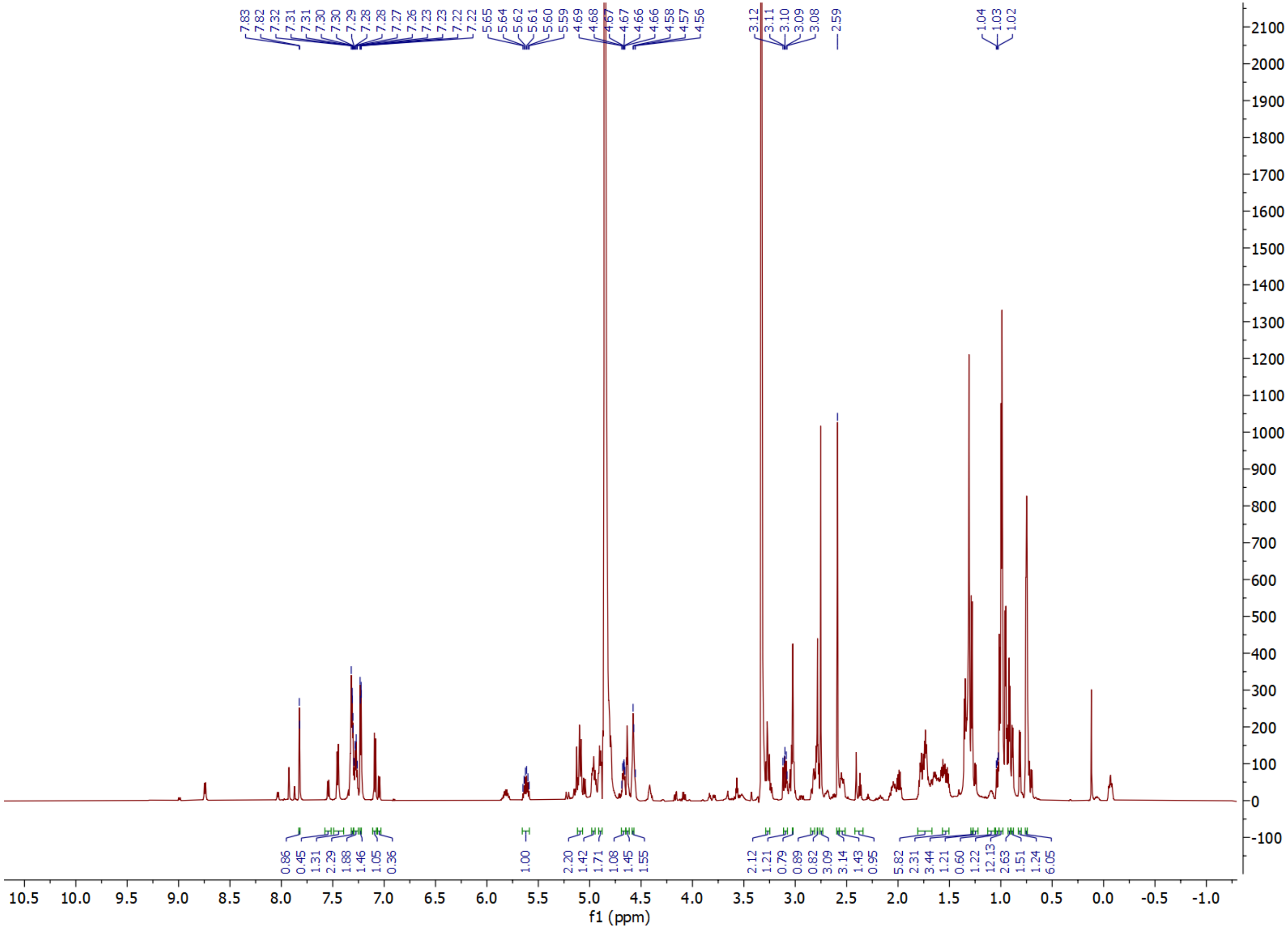

*^{13}C NMR spectrum of rufomycin analogue **7** in CD_3OD at 176 MHz.*

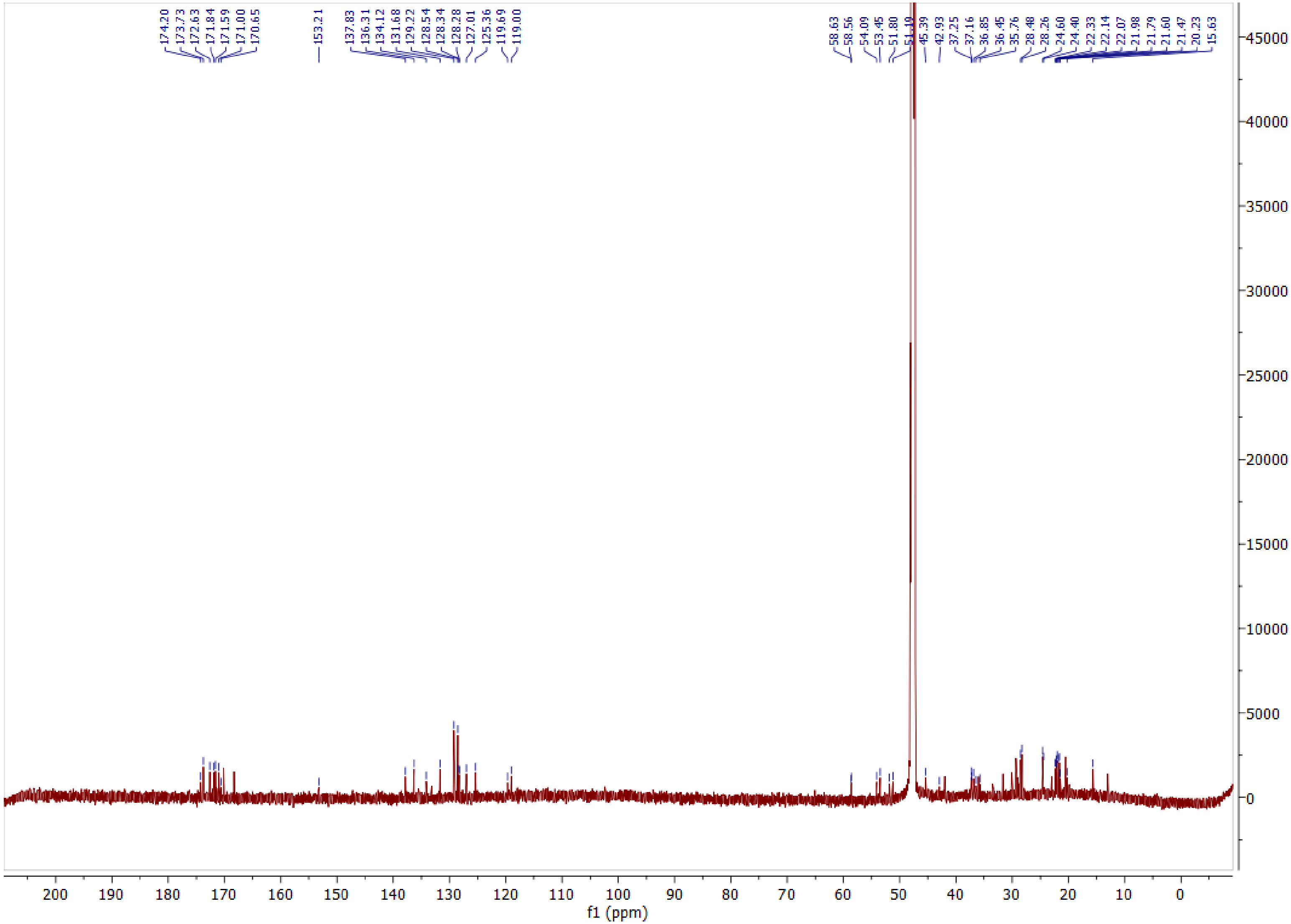

*COSY spectrum of rufomycin analogue **7** in CD_3OD.*

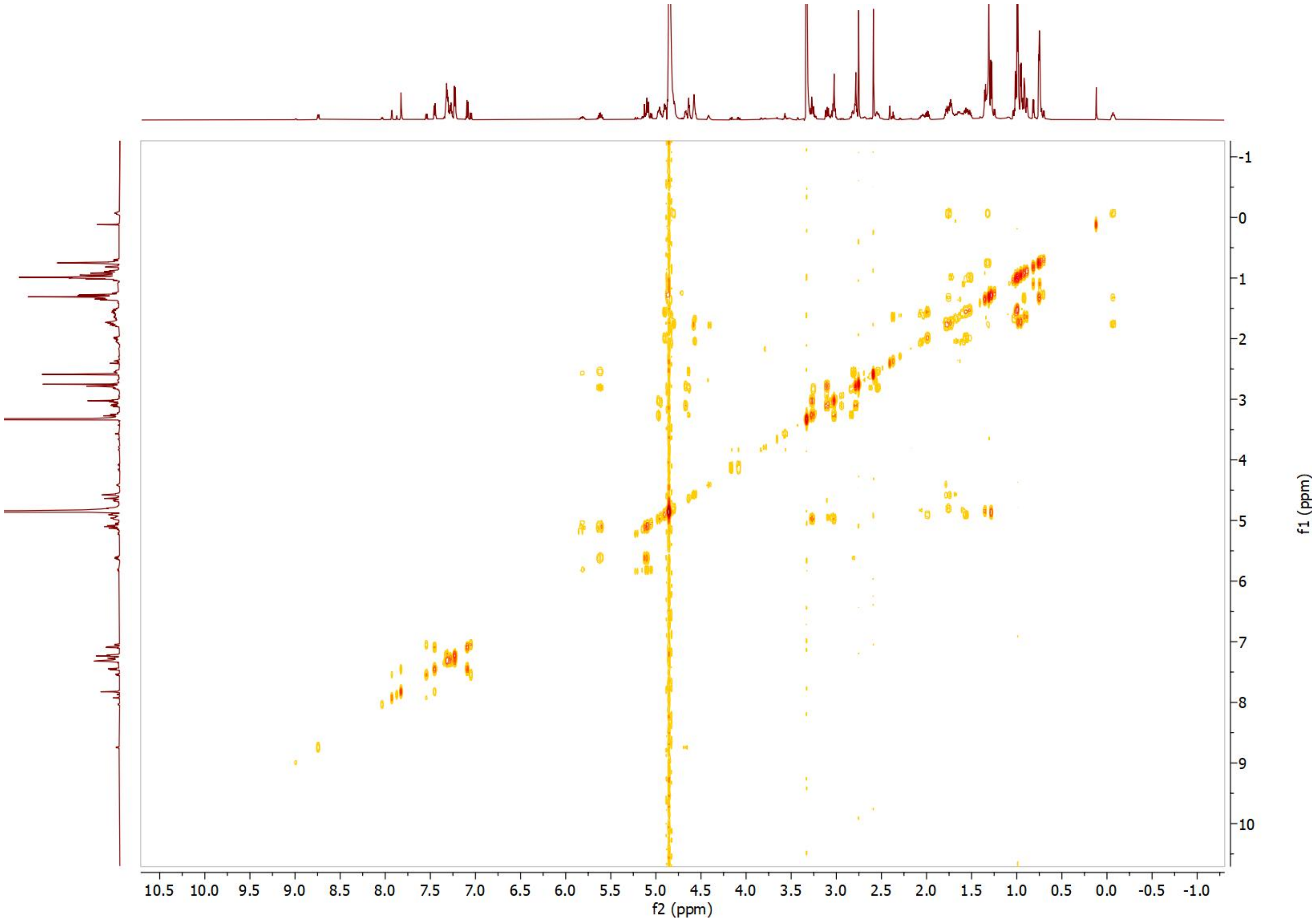

*HSQC spectrum of rufomycin analogue **7** in CD_3OD.*

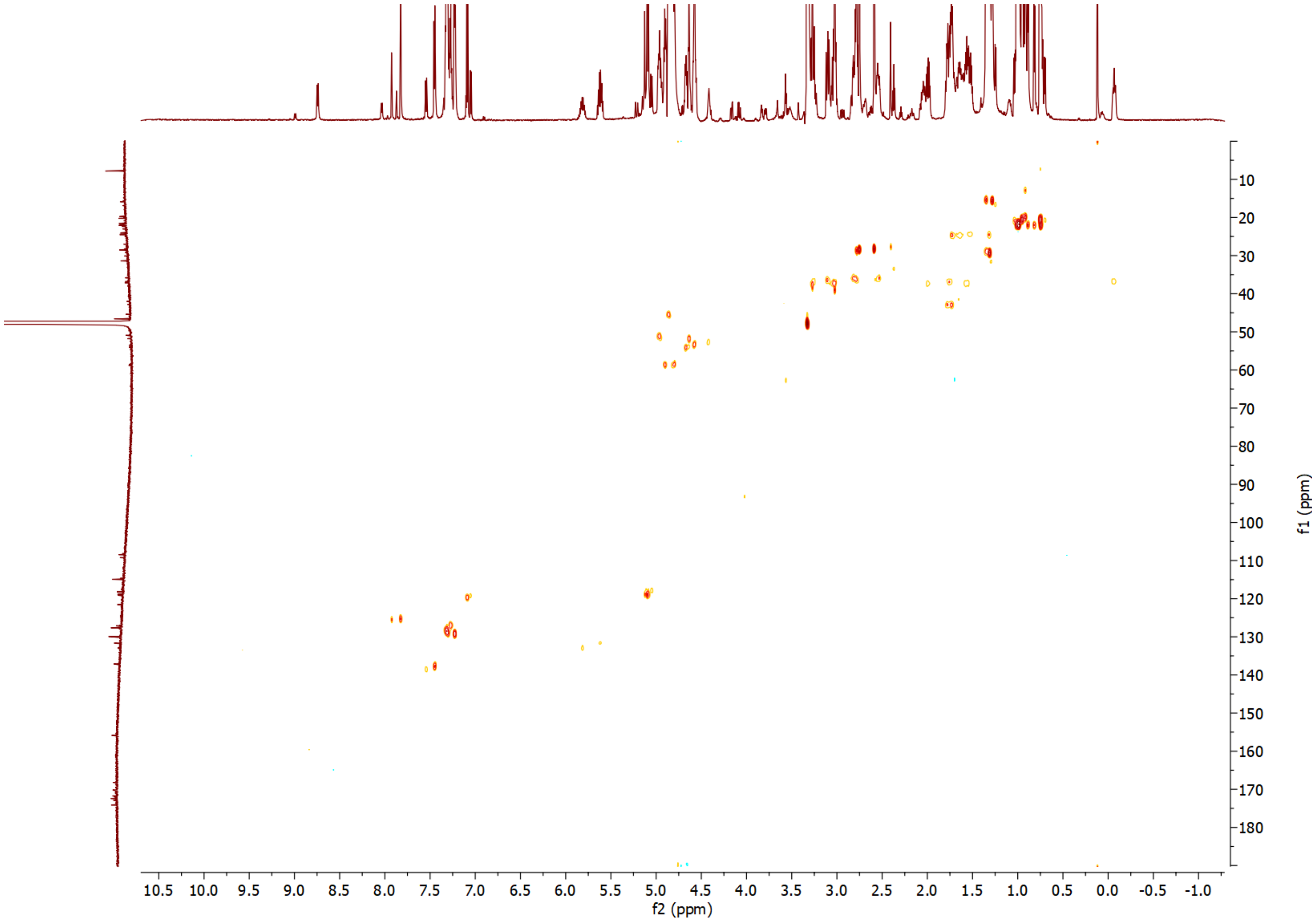

*HMBC spectrum of rufomycin analogue **7** in CD_3OD*

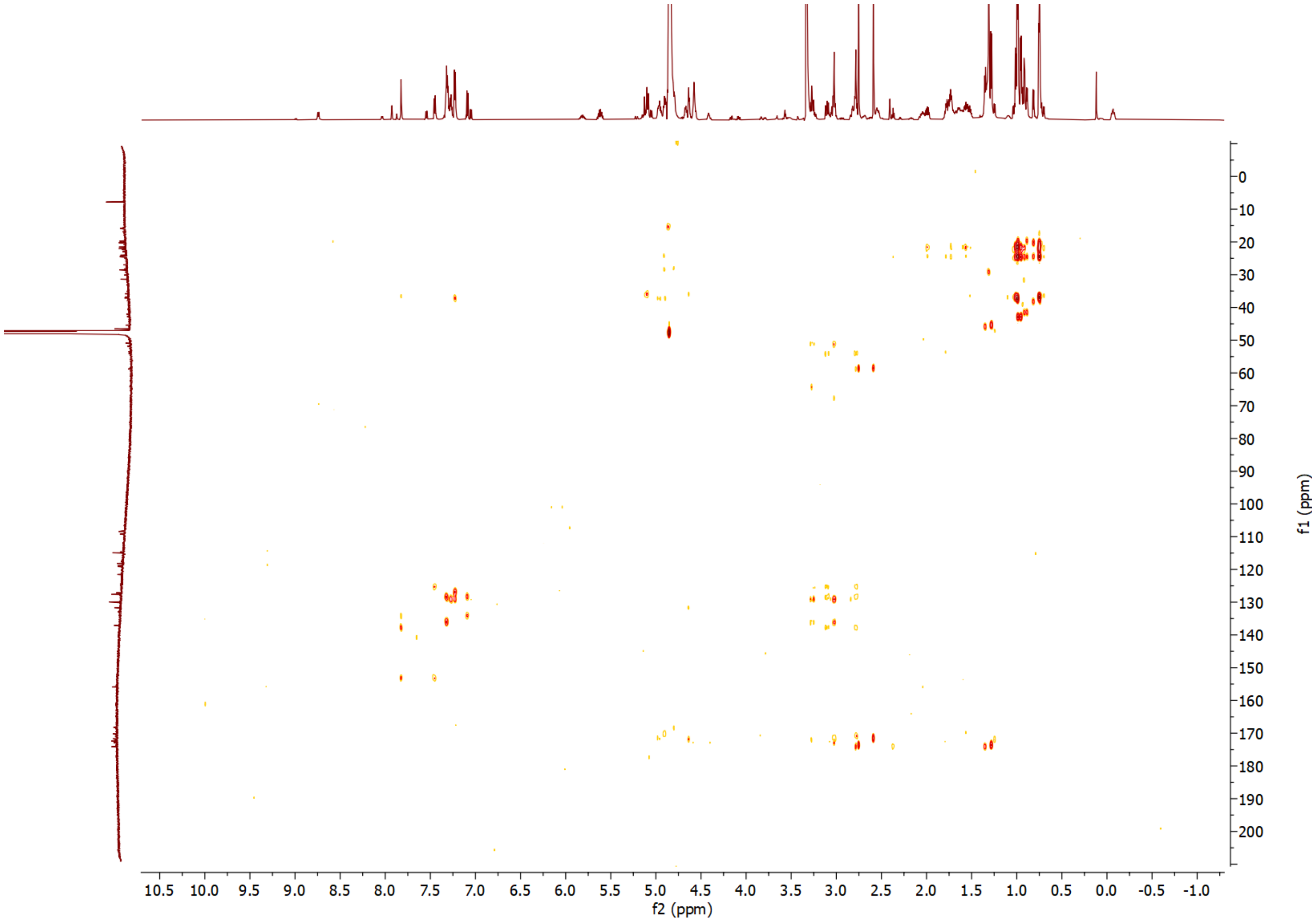

^{1}H NMR spectrum of rufomycin analogue **9** in CD_3OD at 700 MHz.

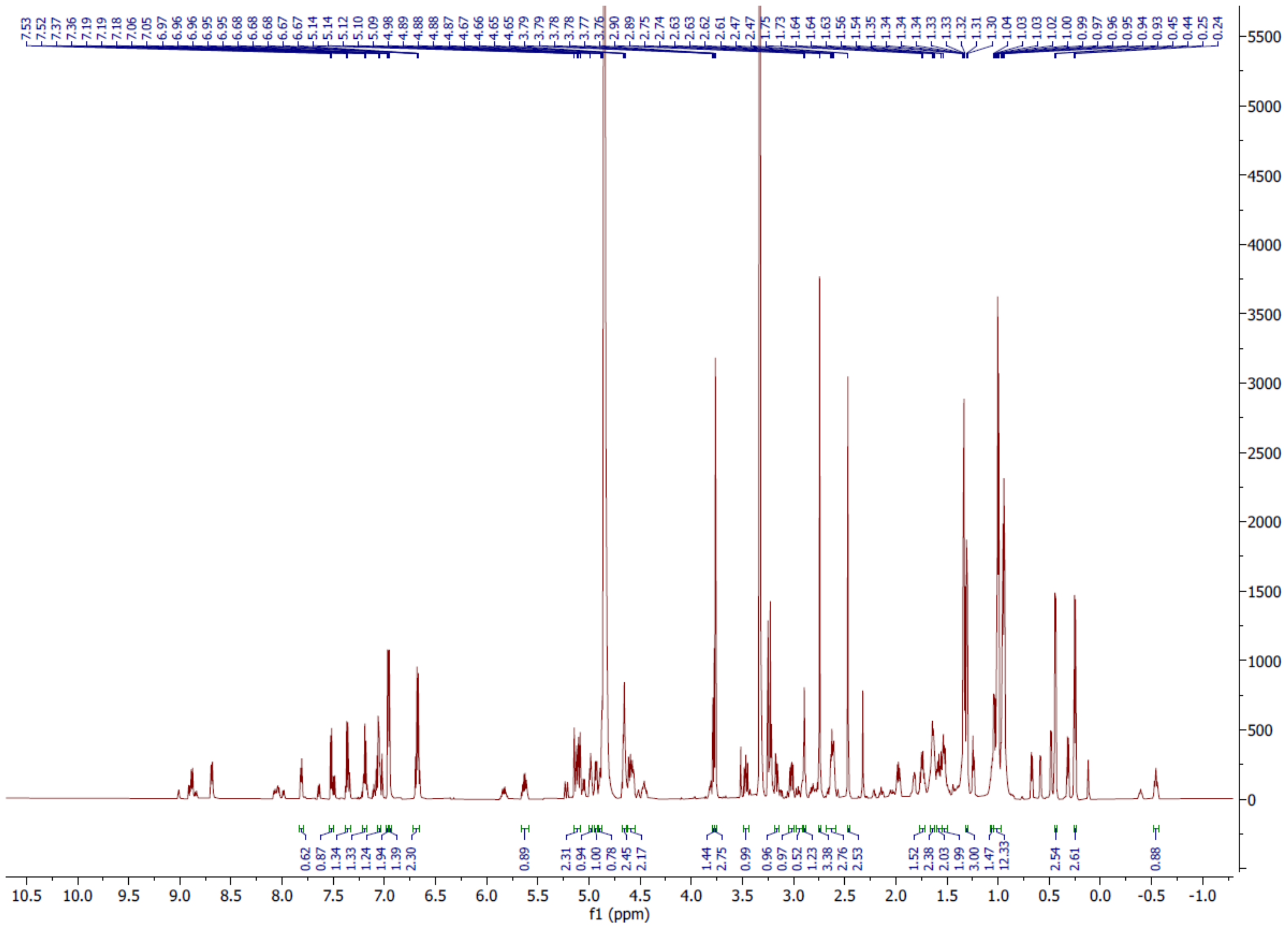

^{13}C NMR spectrum of rufomycin analogue **9** in CD_3OD at 176 MHz.

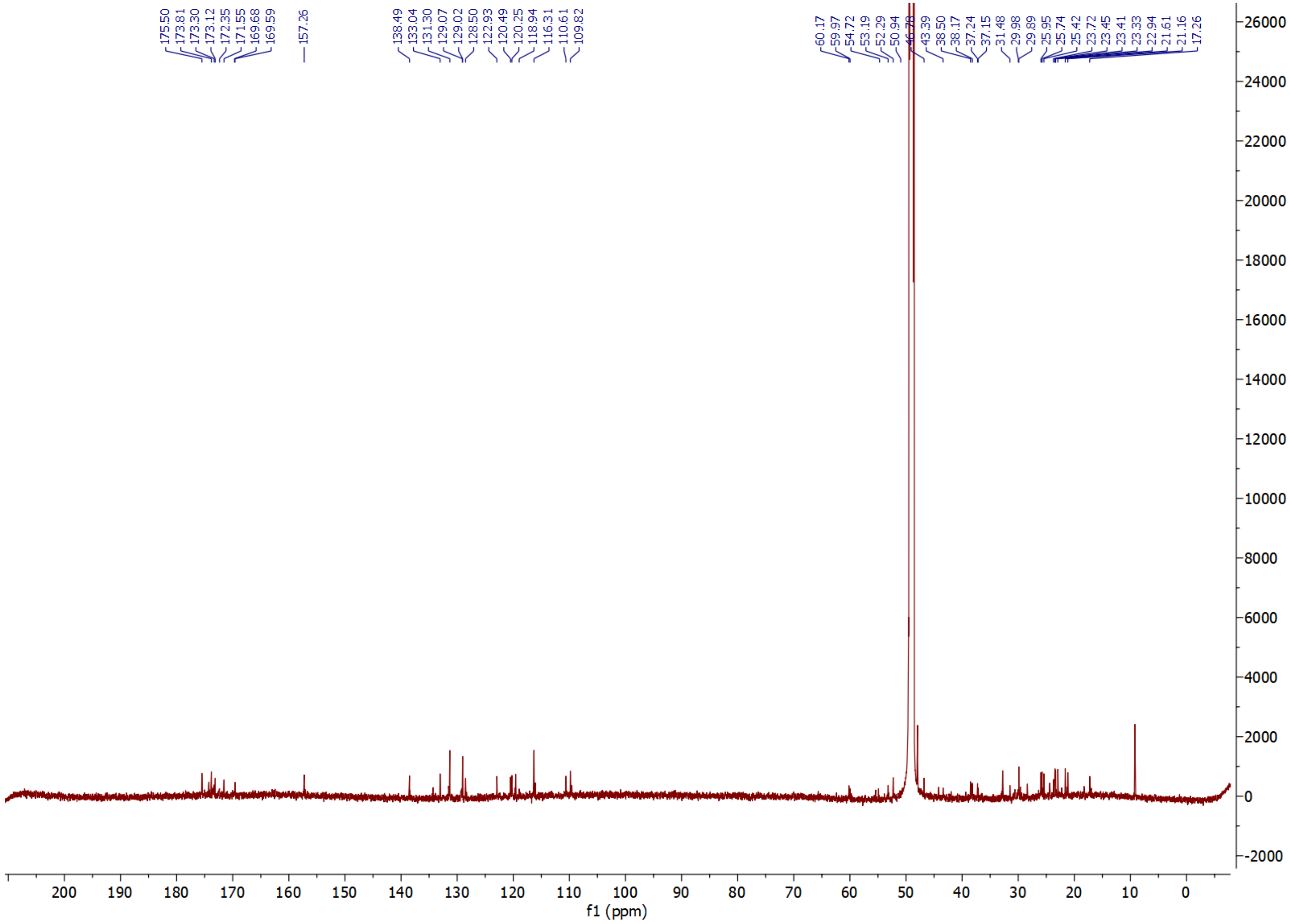

*COSY spectrum of rufomycin analogue **9** in CD_3OD.*

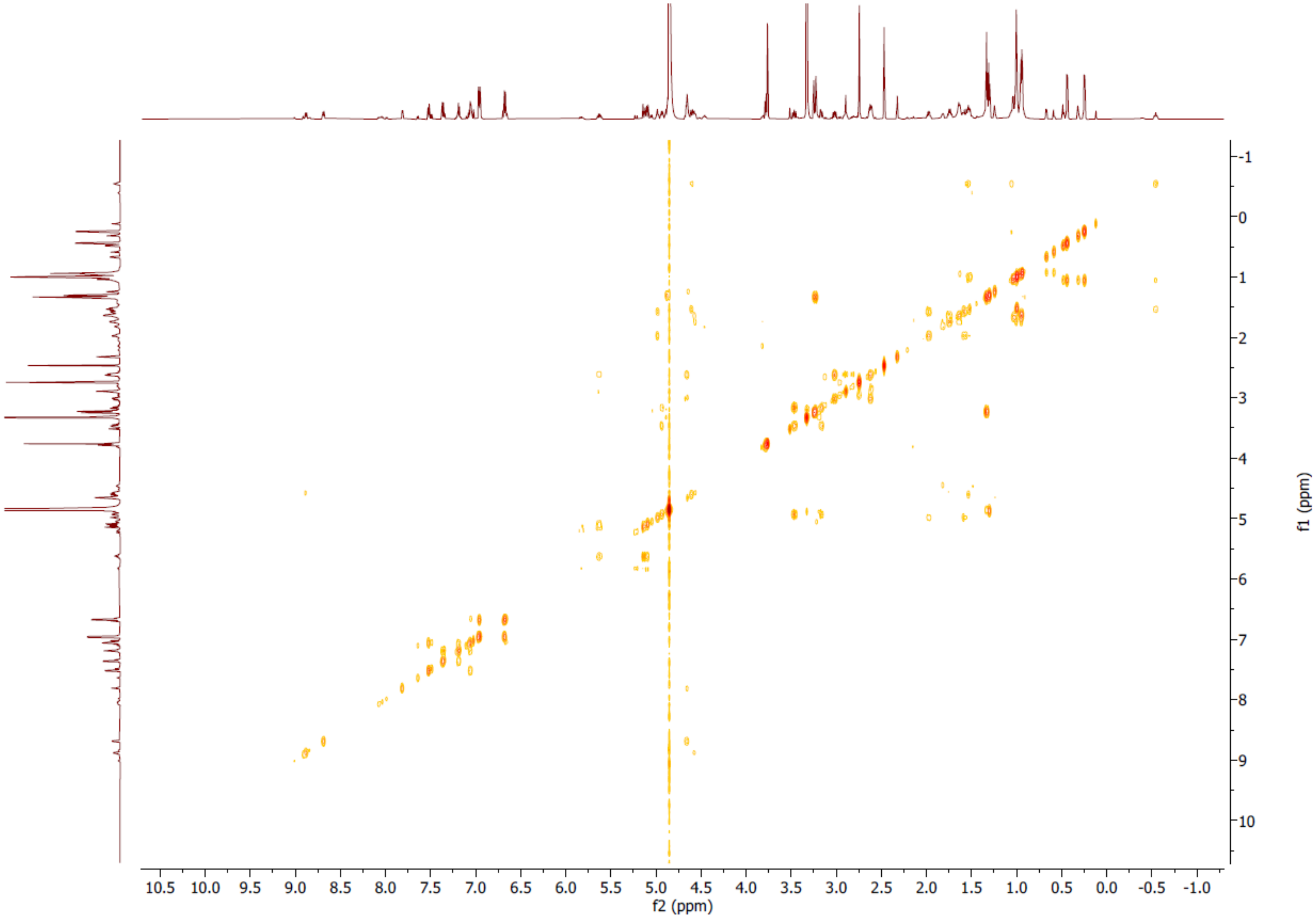

*HSQC spectrum of rufomycin analogue **9** in CD_3OD.*

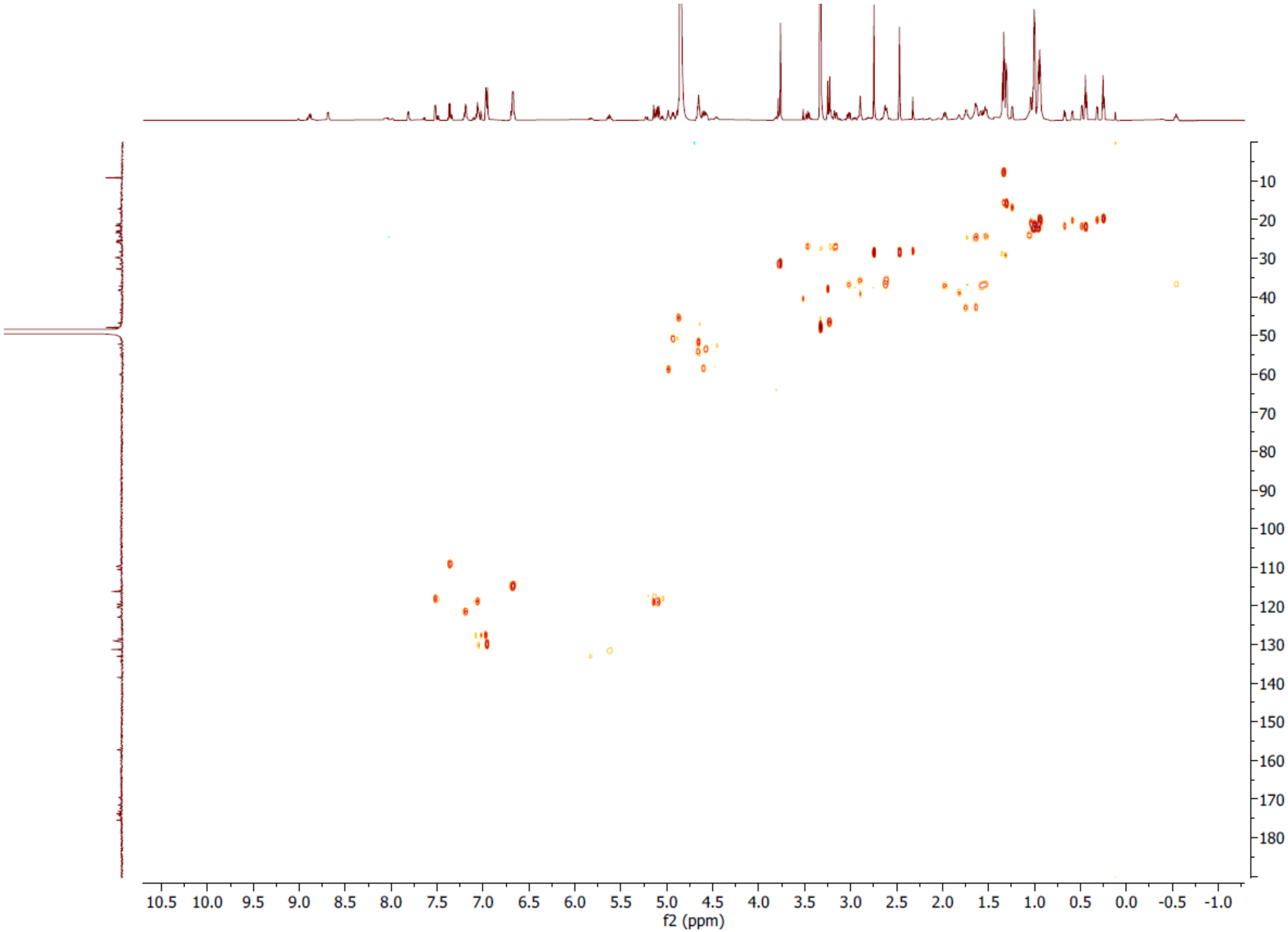

*HMBC spectrum of rufomycin analogue **9** in CD_3OD.*

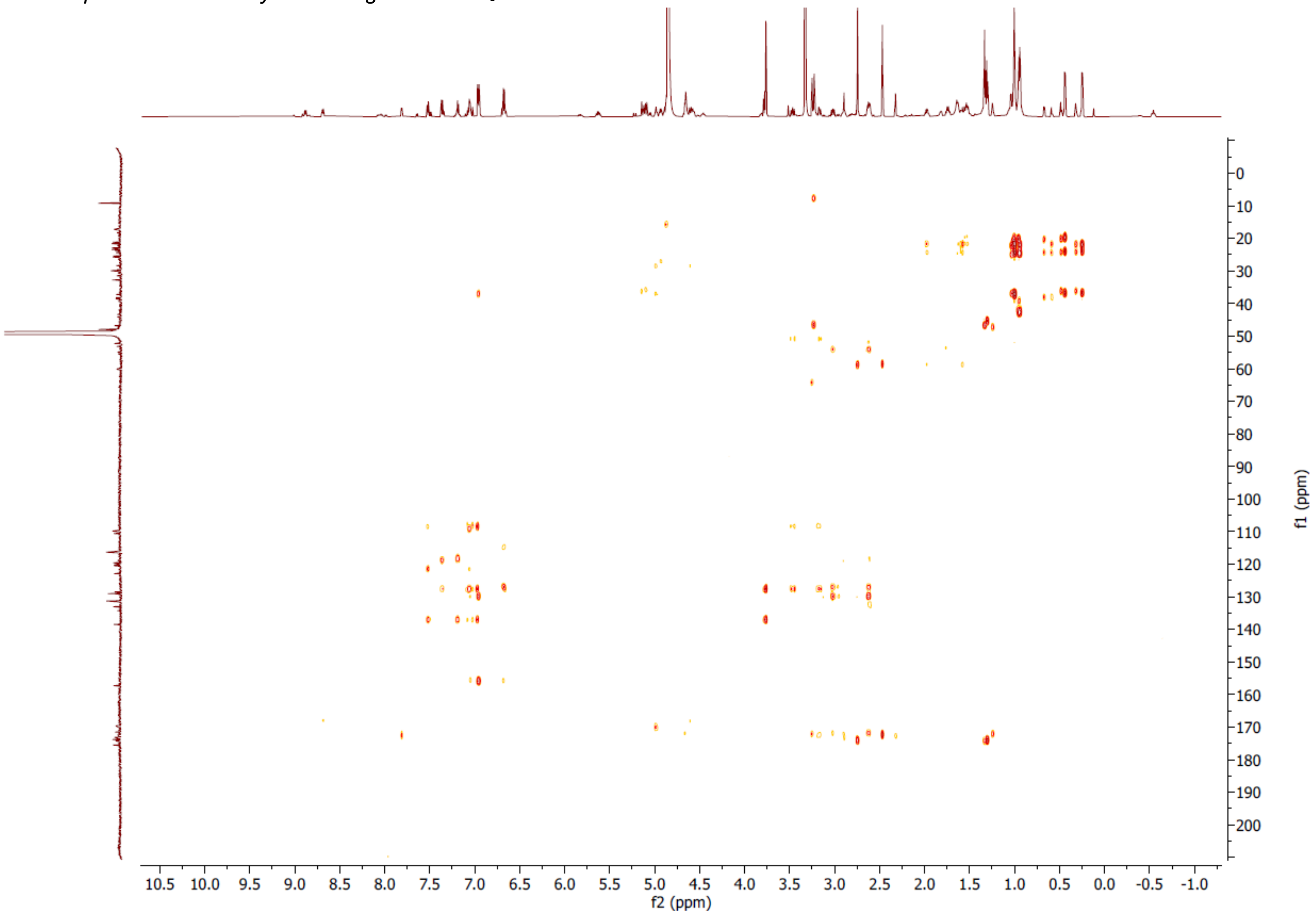

^{1}H NMR spectrum of rufomycin analogue **10** in CD_3OD at 700 MHz.

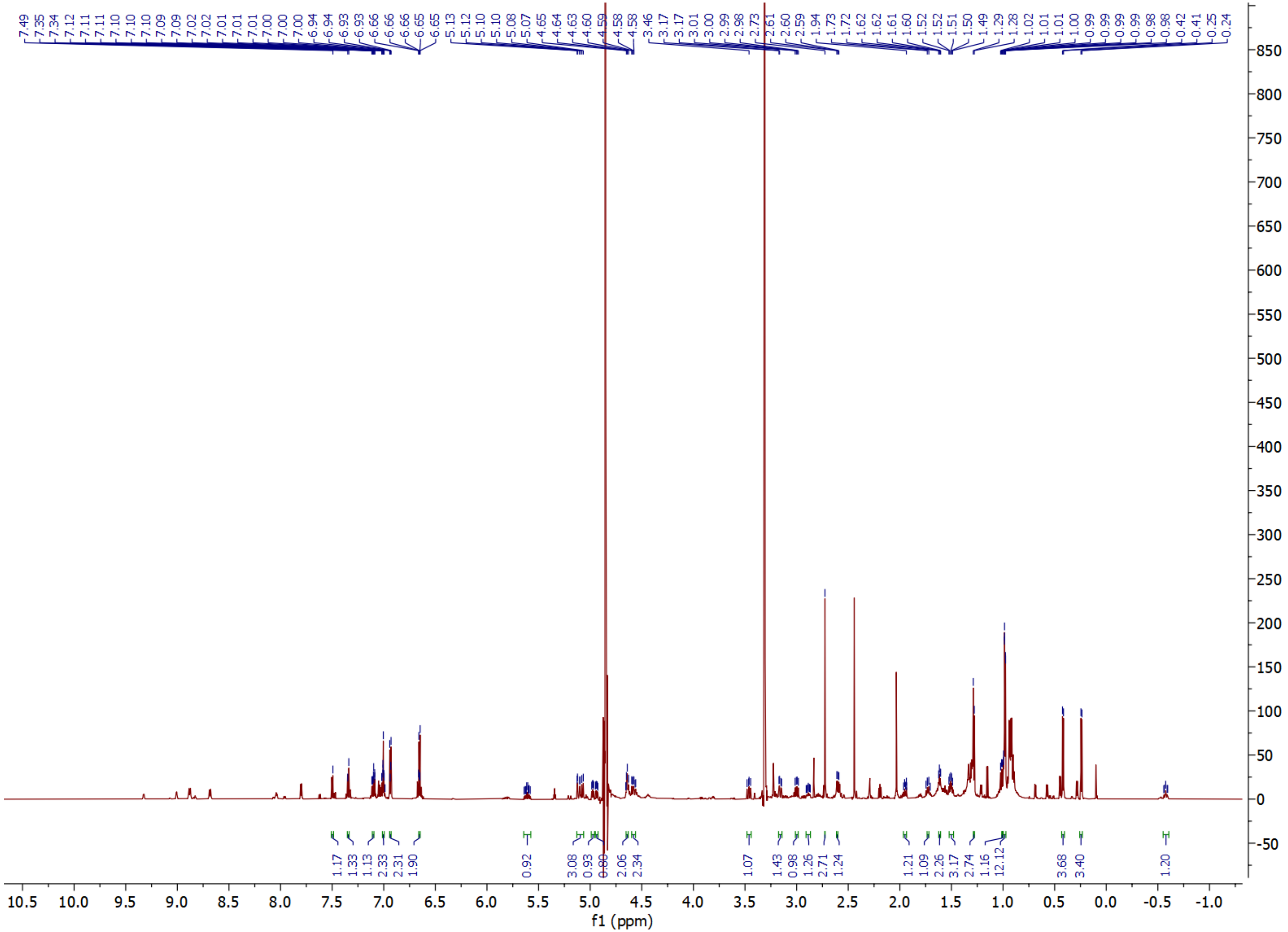

^{13}C NMR spectrum of rufomycin analogue **10** in CD_3OD at 176 MHz.

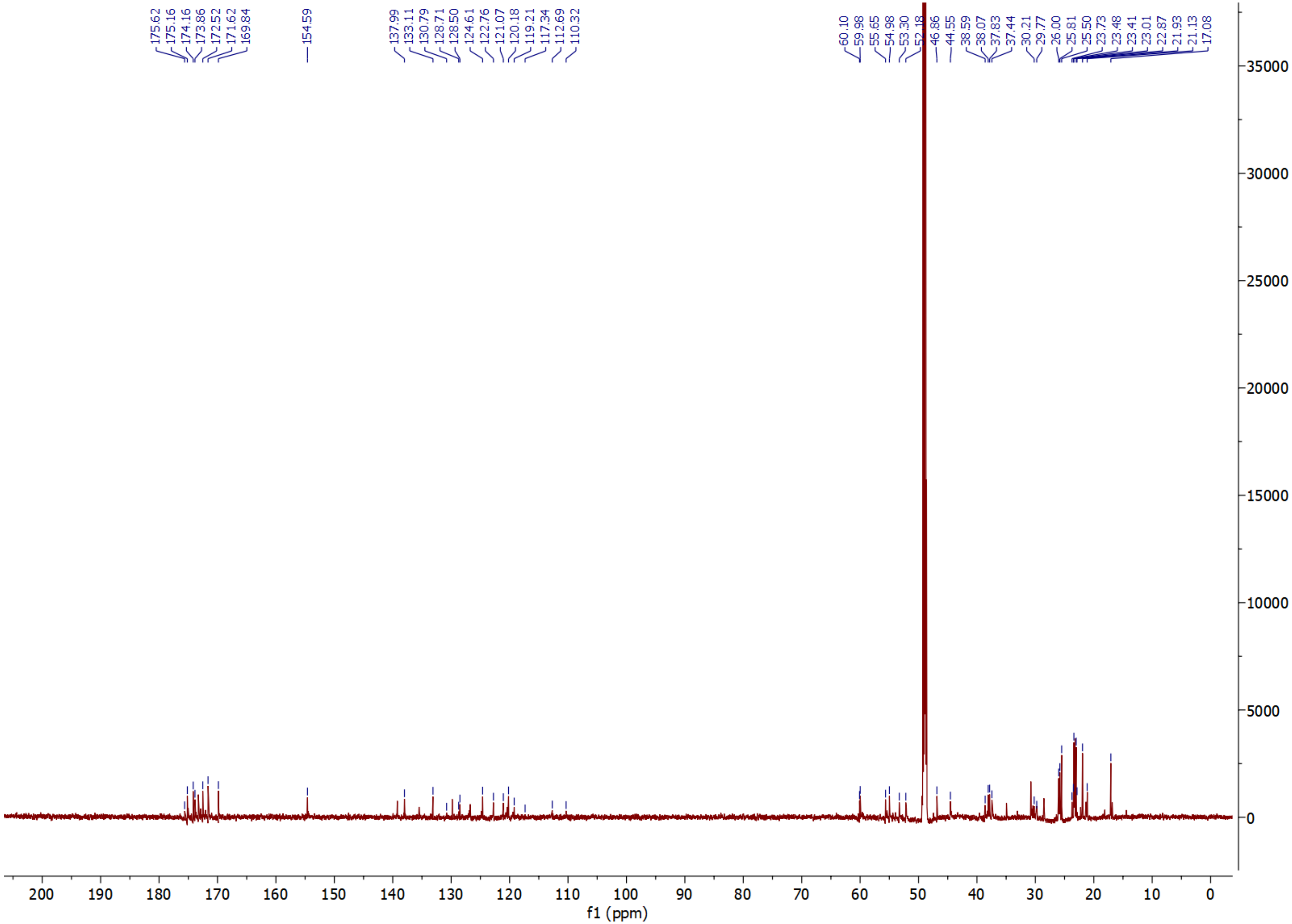

*COSY spectrum of rufomycin analogue **10**in CD_3OD.*

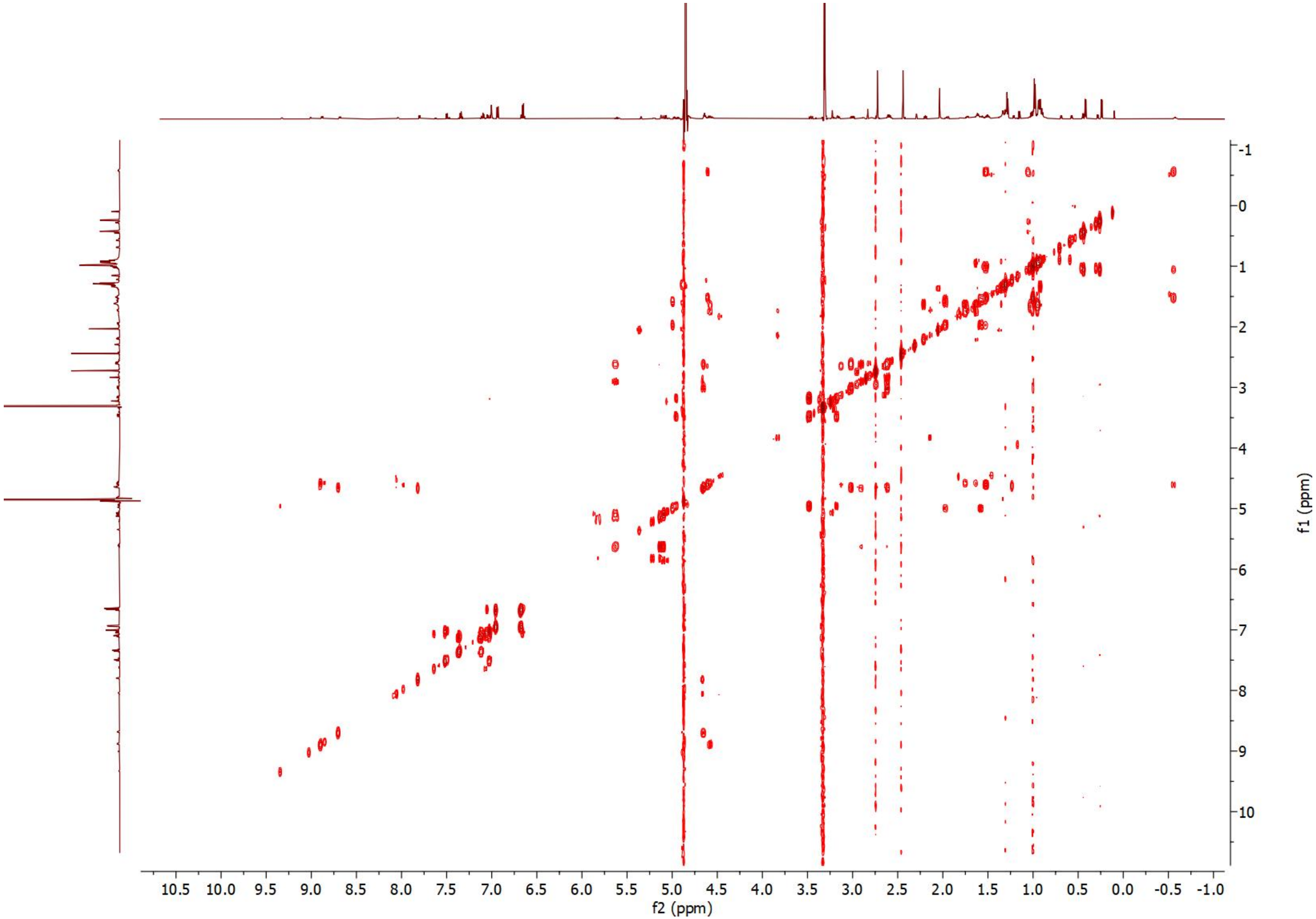

*HSQC spectrum of rufomycin analogue **10** in CD_3OD.*

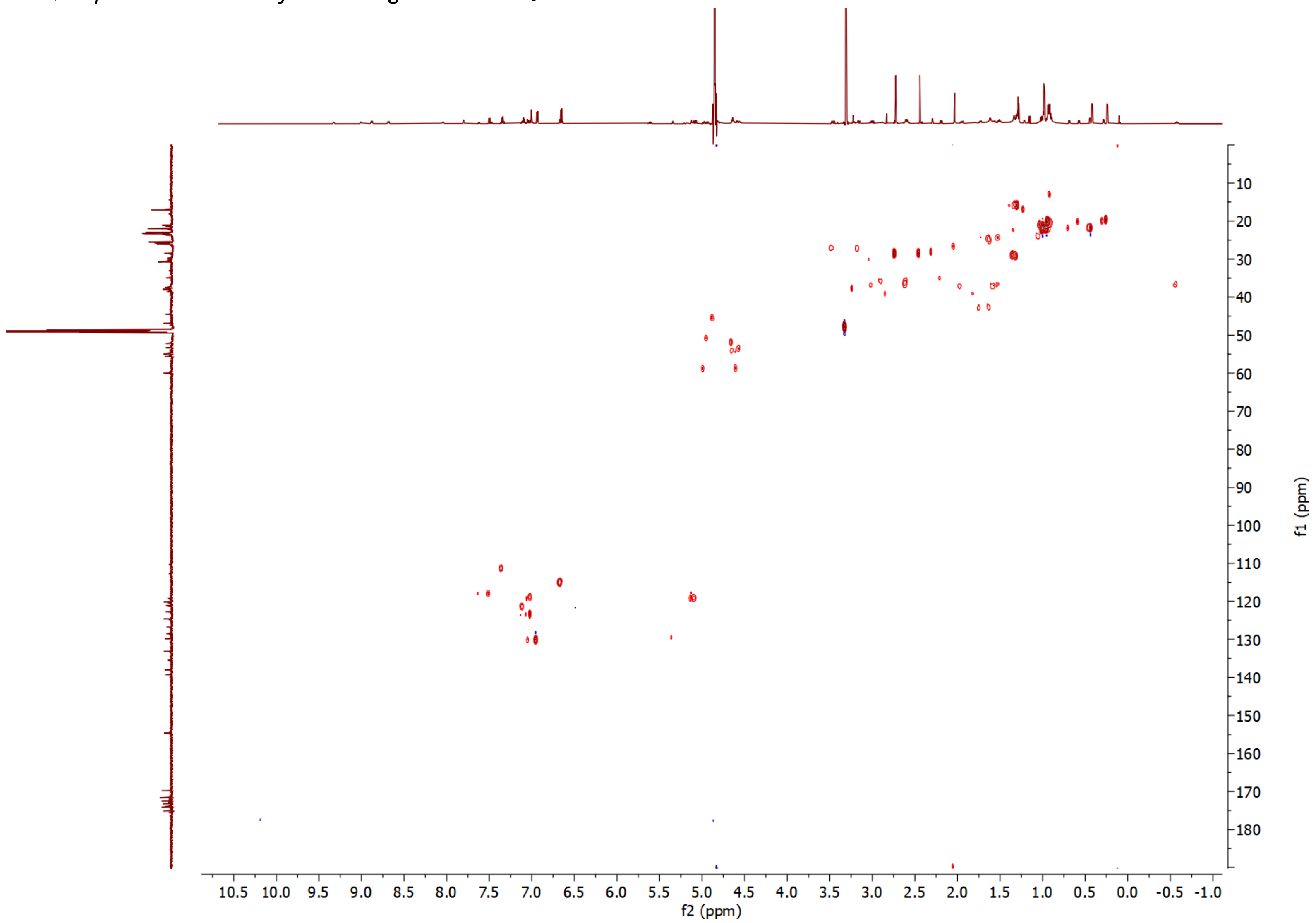

*HMBC spectrum of rufomycin analogue **10** in CD_3OD*

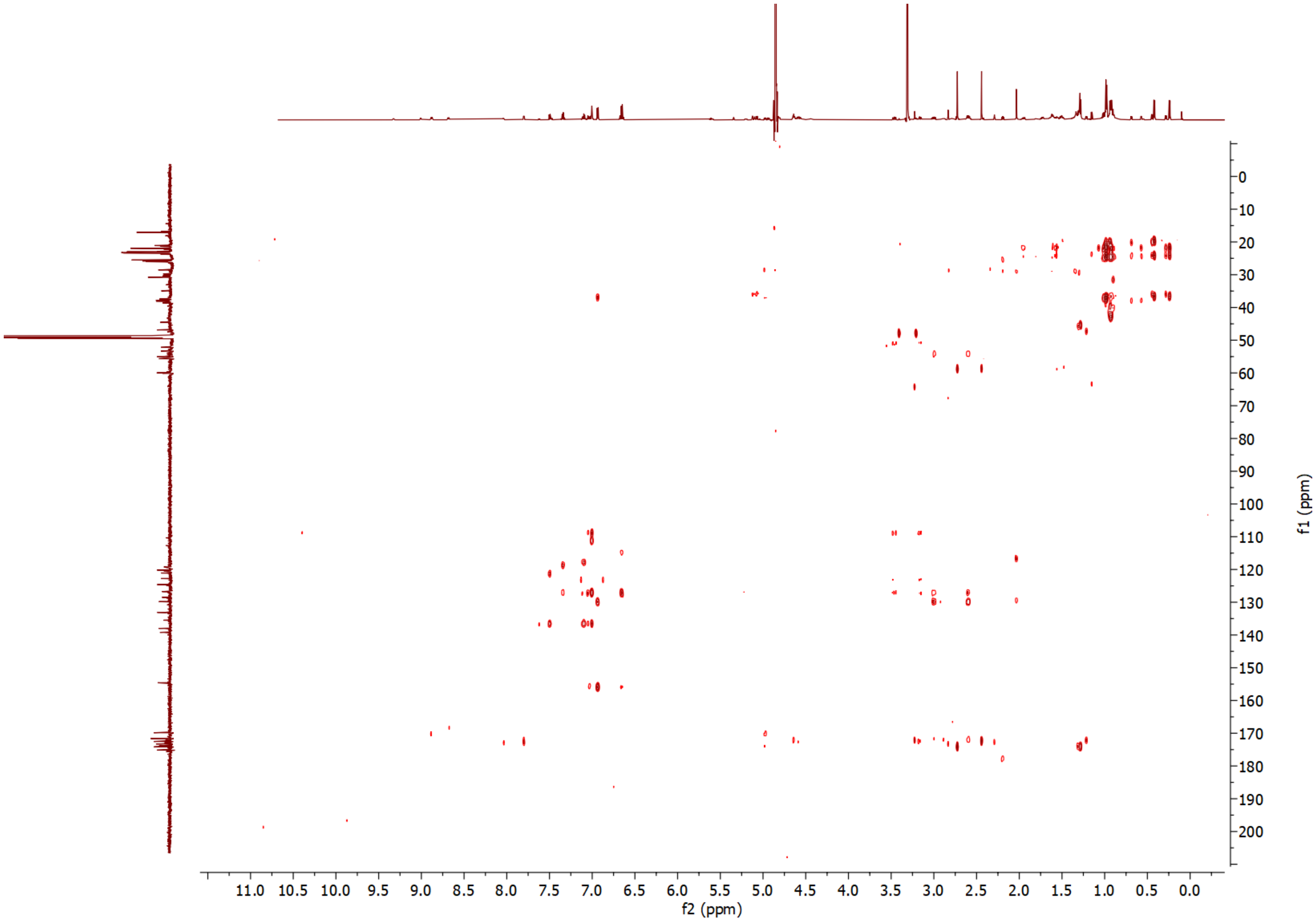

*^{1}H NMR spectrum of rufomycin analogue **15** in CD_3OD at 700 MHz.*

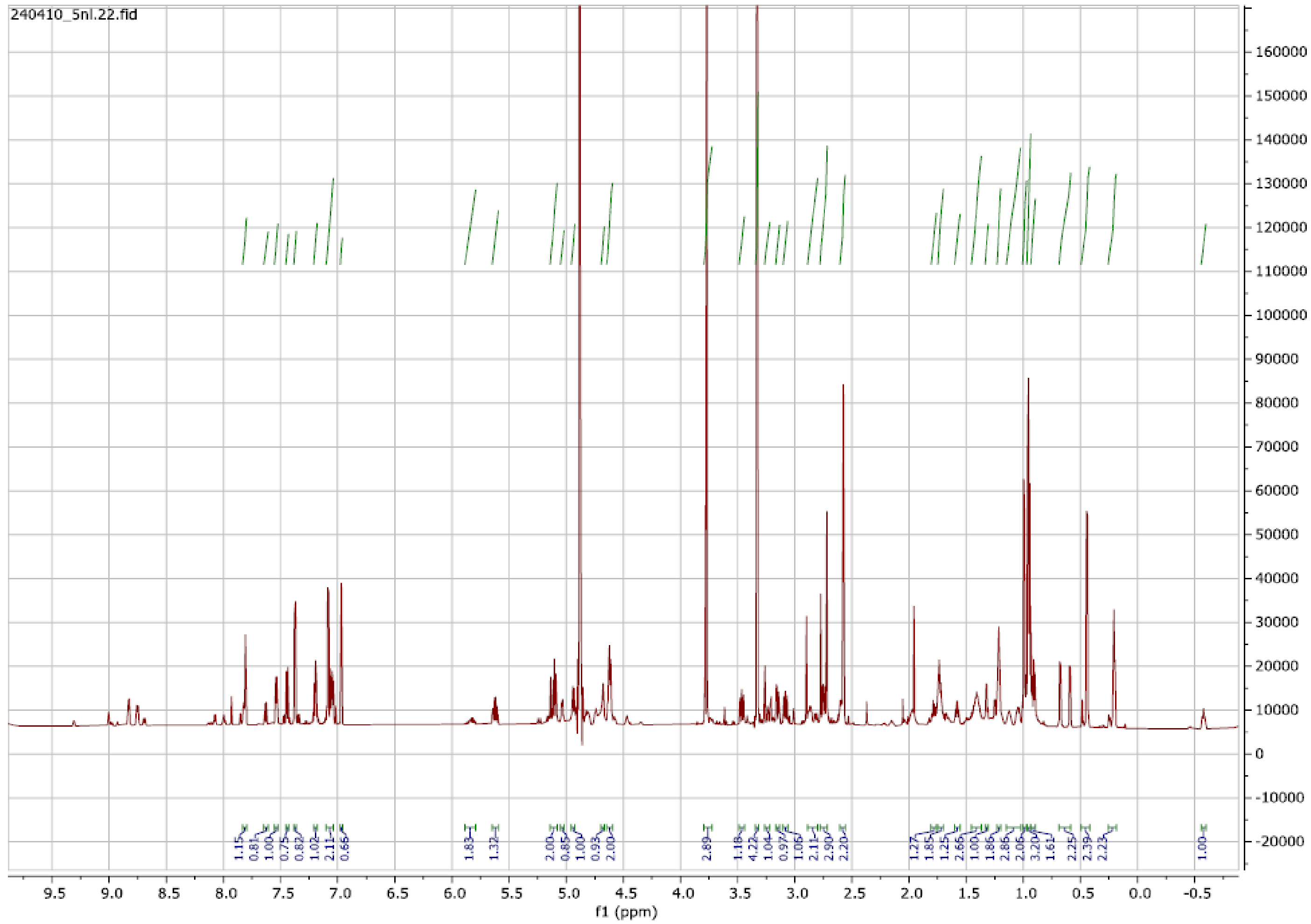

*^{13}C NMR spectrum of rufomycin analogue **15** in CD_3OD at 151 MHz.*

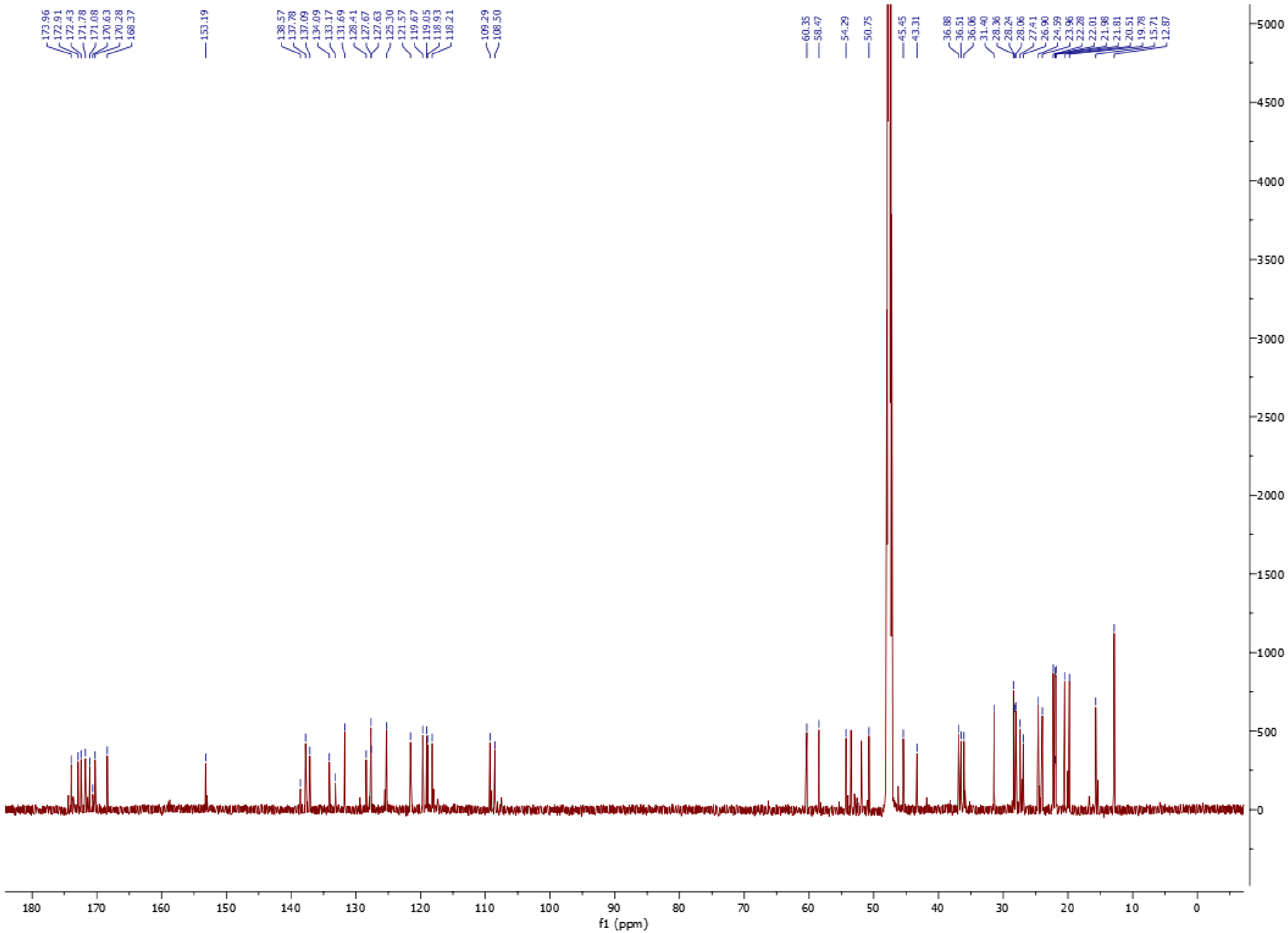

*COSY spectrum of rufomycin analogue **15** in CD_3OD.*

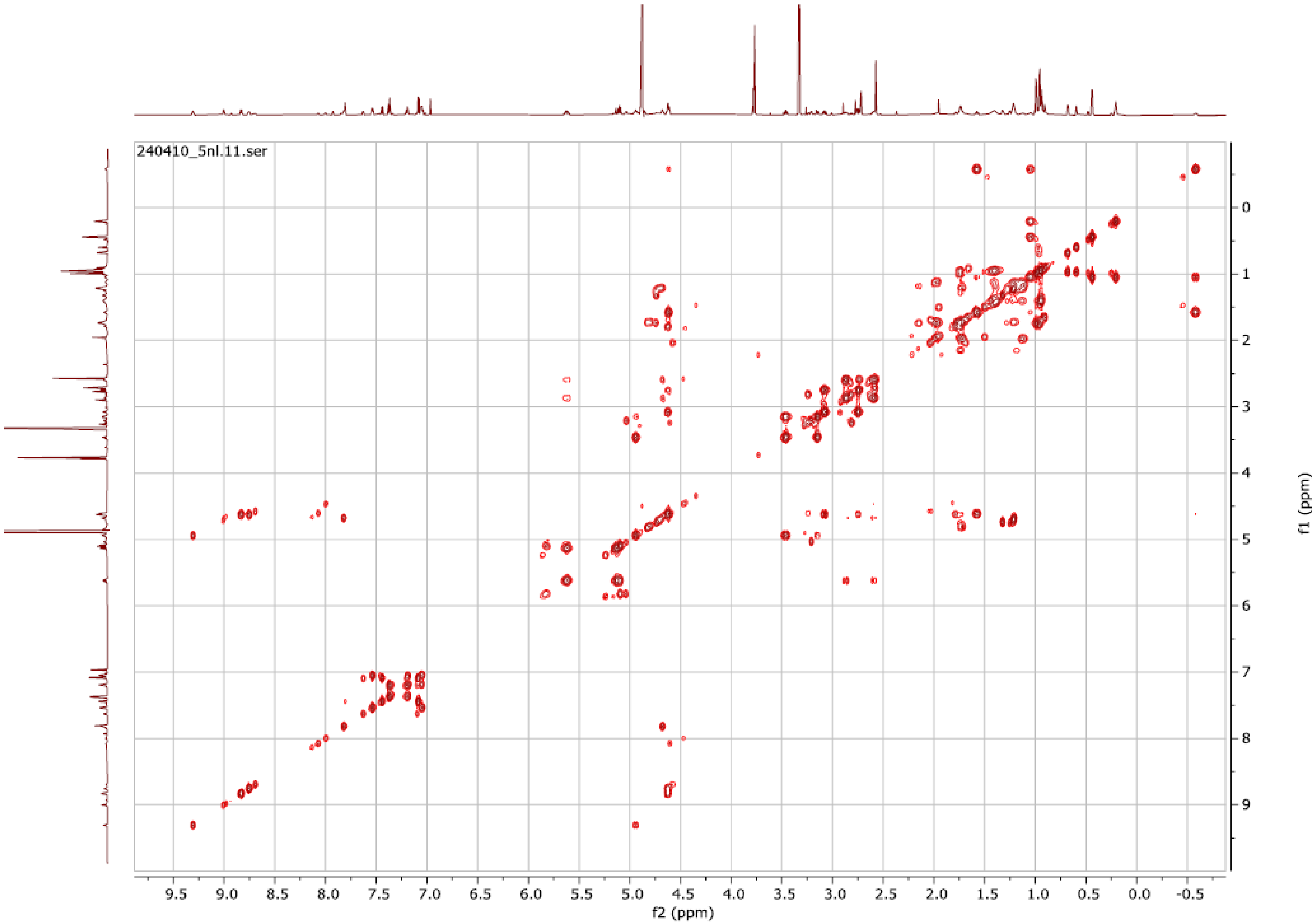

HSQC spectrum of rufomycin analogue **15** *in* CD_3OD.

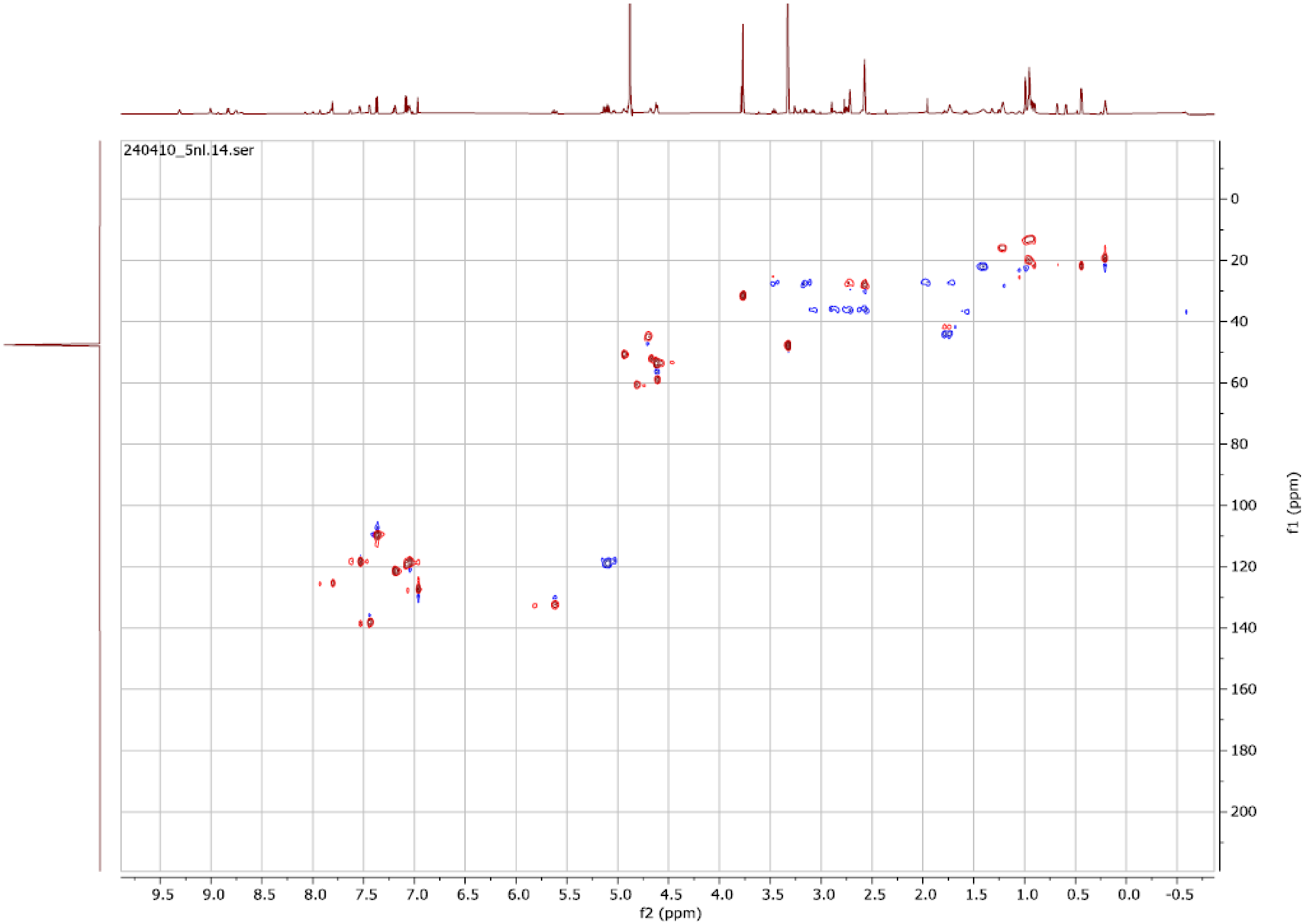

*HMBC spectrum of rufomycin analogue **15** in CD_3OD*

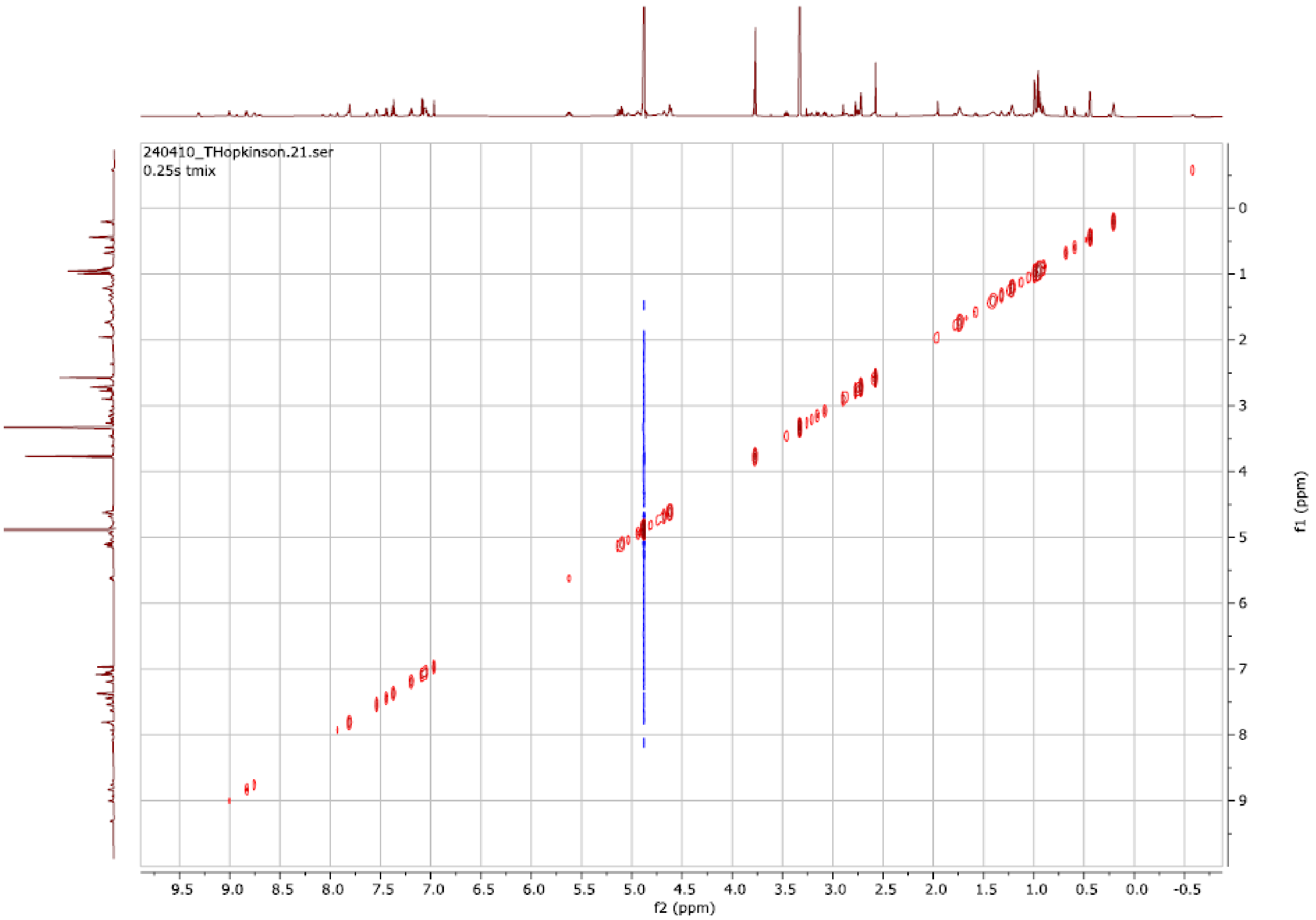

*^{1}H NMR spectrum of rufomycin analogue **16** in CD_3OD at 700 MHz.*

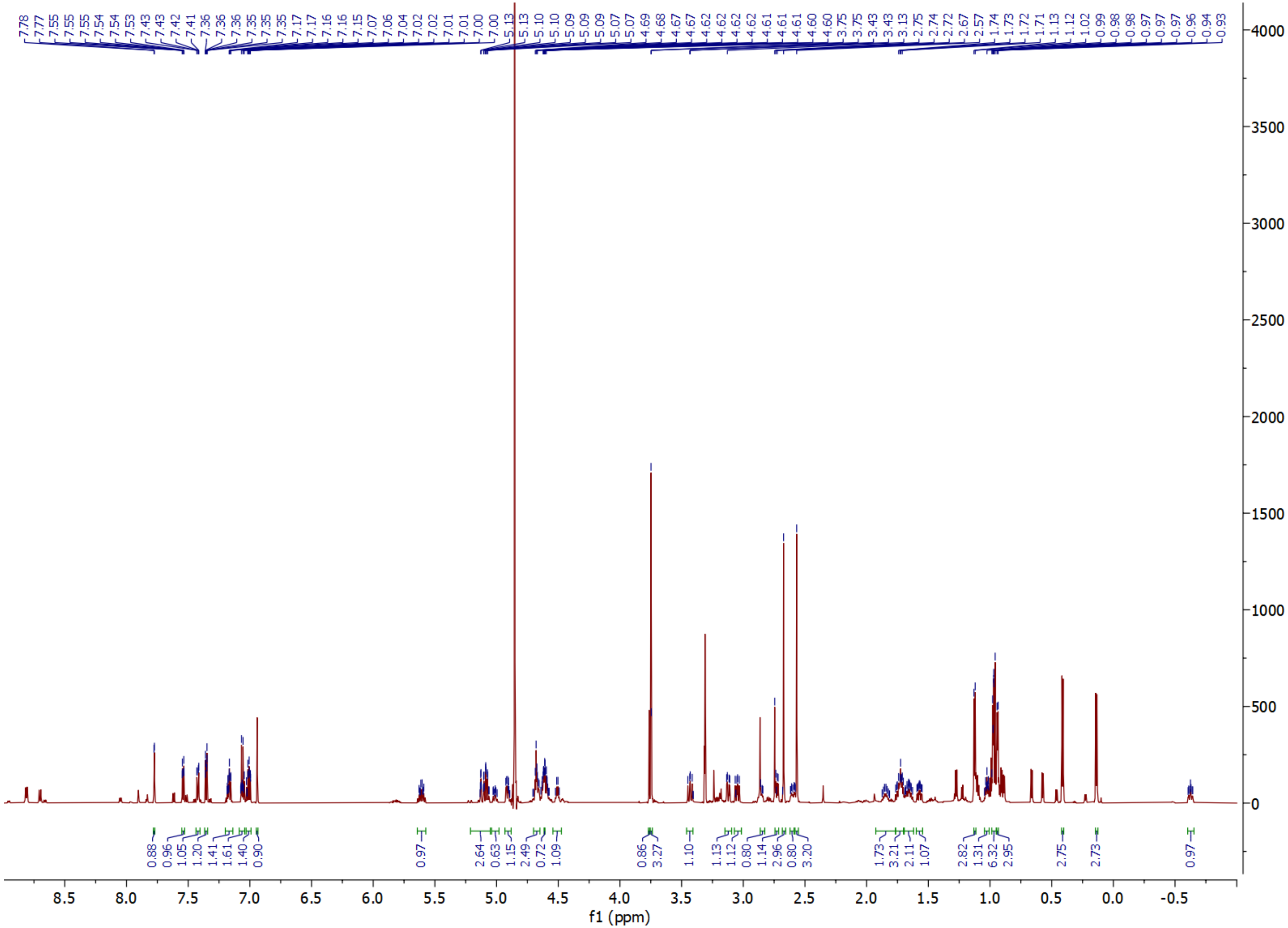

^{13}C NMR spectrum of rufomycin analogue **16** in CD_3OD at 176 MHz.

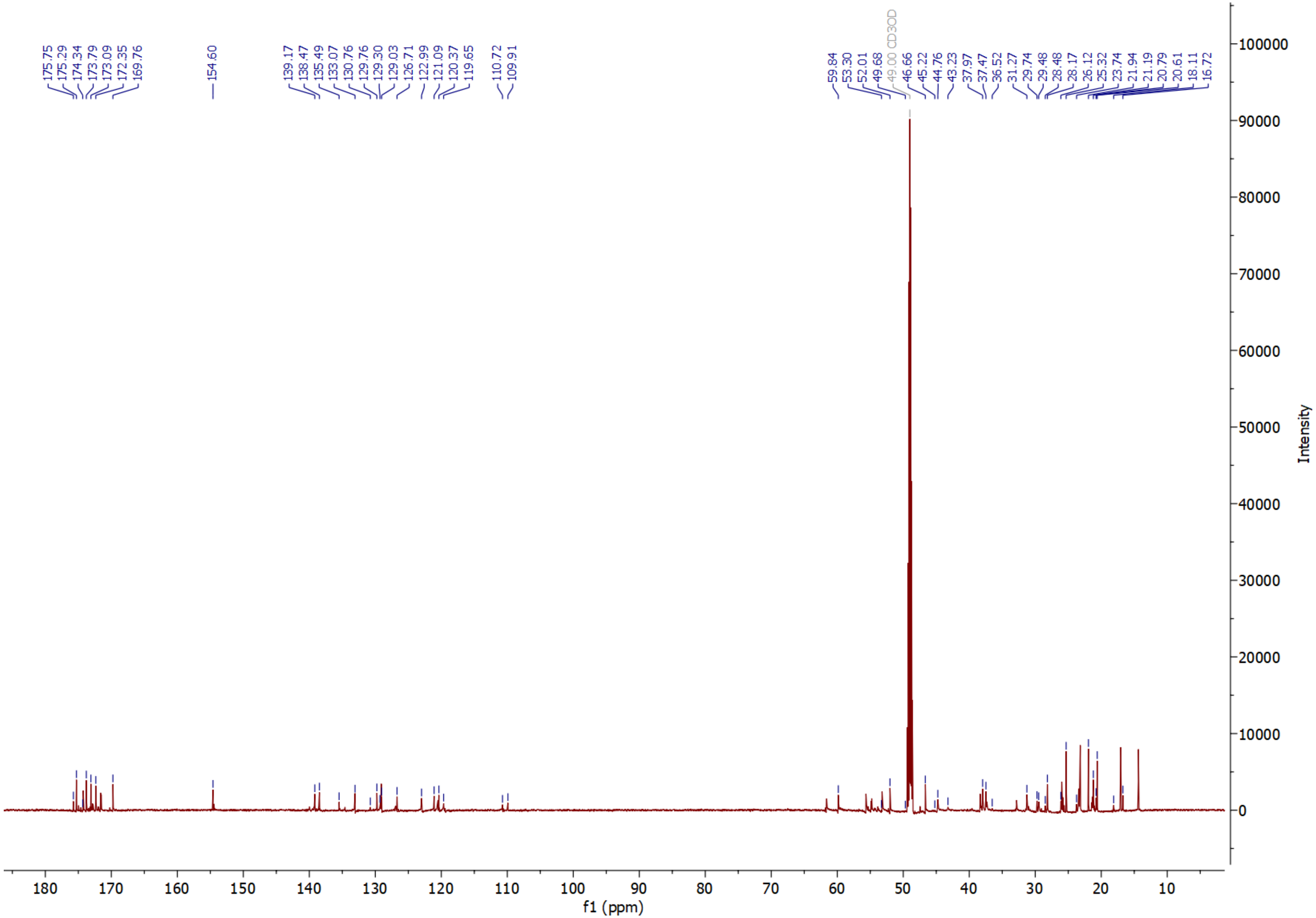

*COSY spectrum of rufomycin analogue **16** in CD_3OD.*

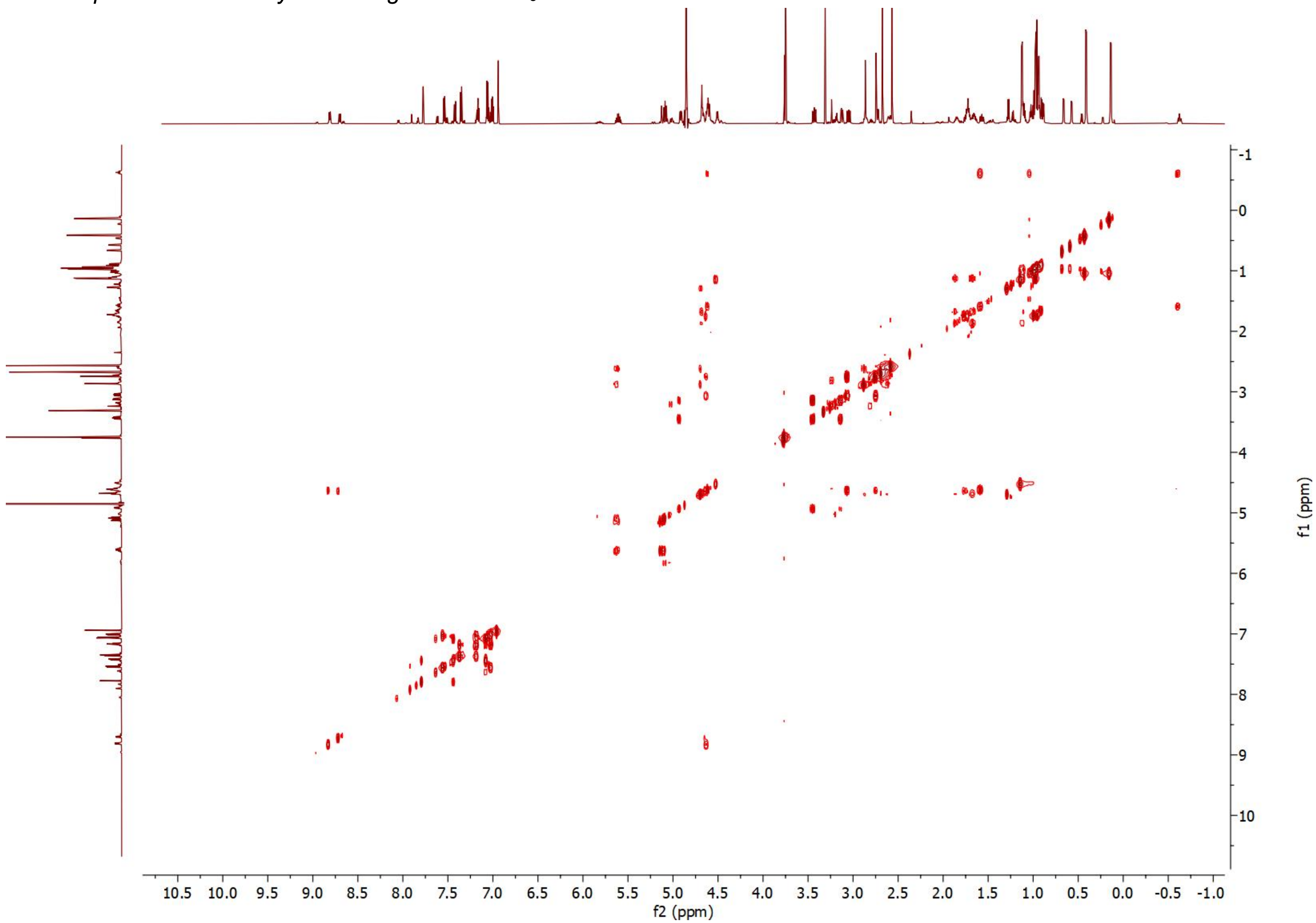

*HSQC spectrum of rufomycin analogue **16**in CD_3OD.*

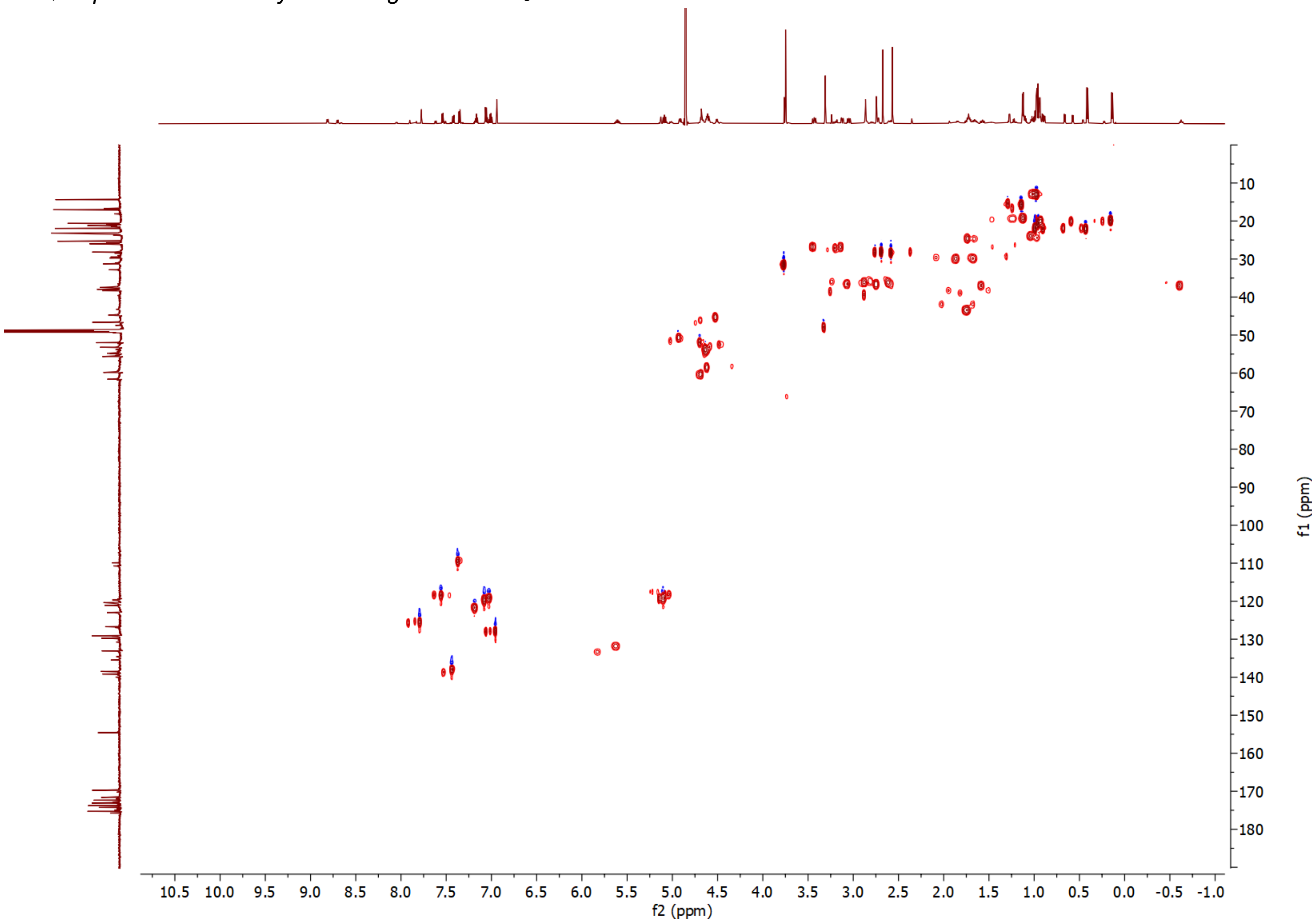

*HMBC spectrum of rufomycin analogue **16** in CD_3OD*

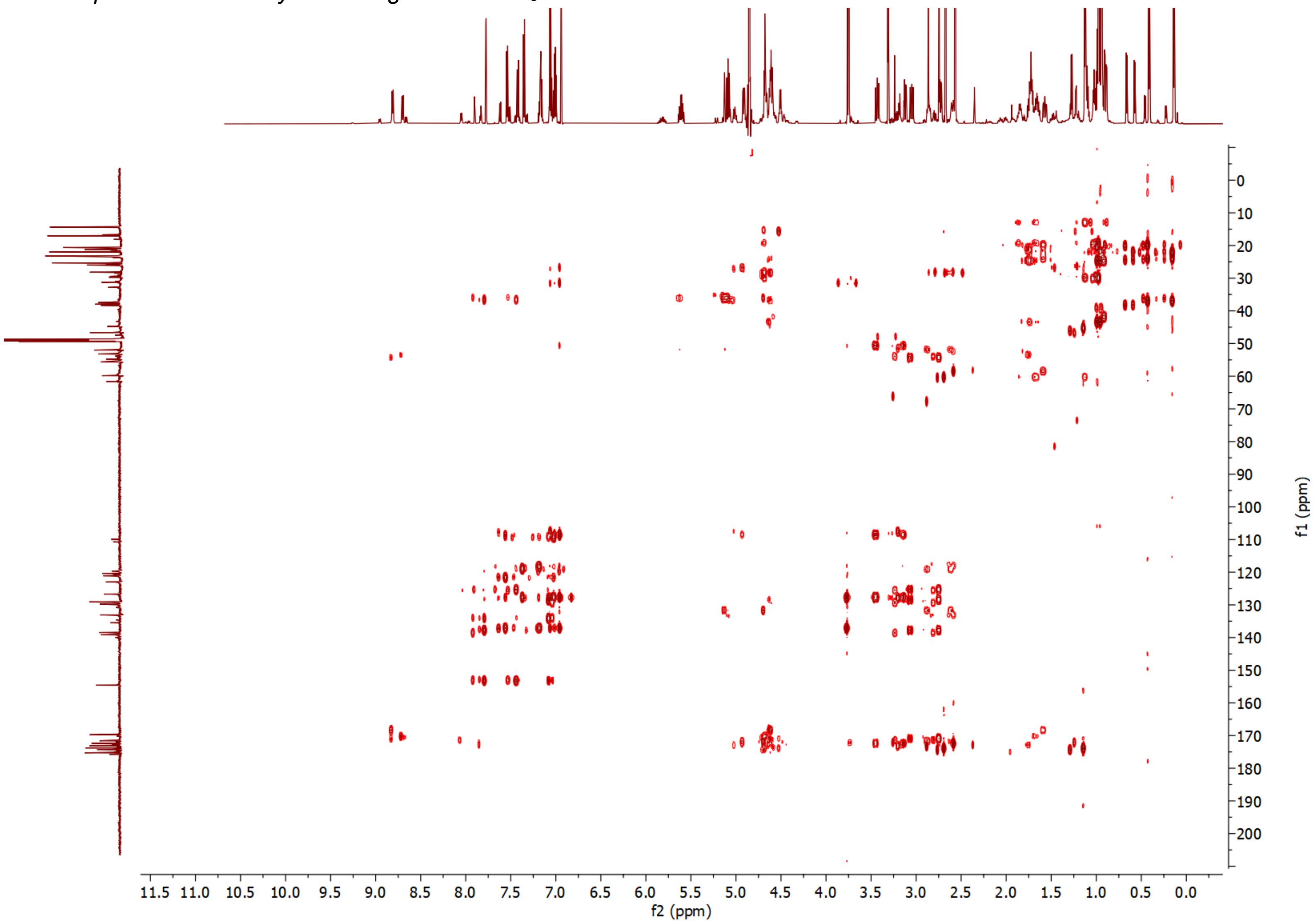

*^{1}H NMR spectrum of rufomycin analogue **17** in CD_3OD at 700 MHz.*

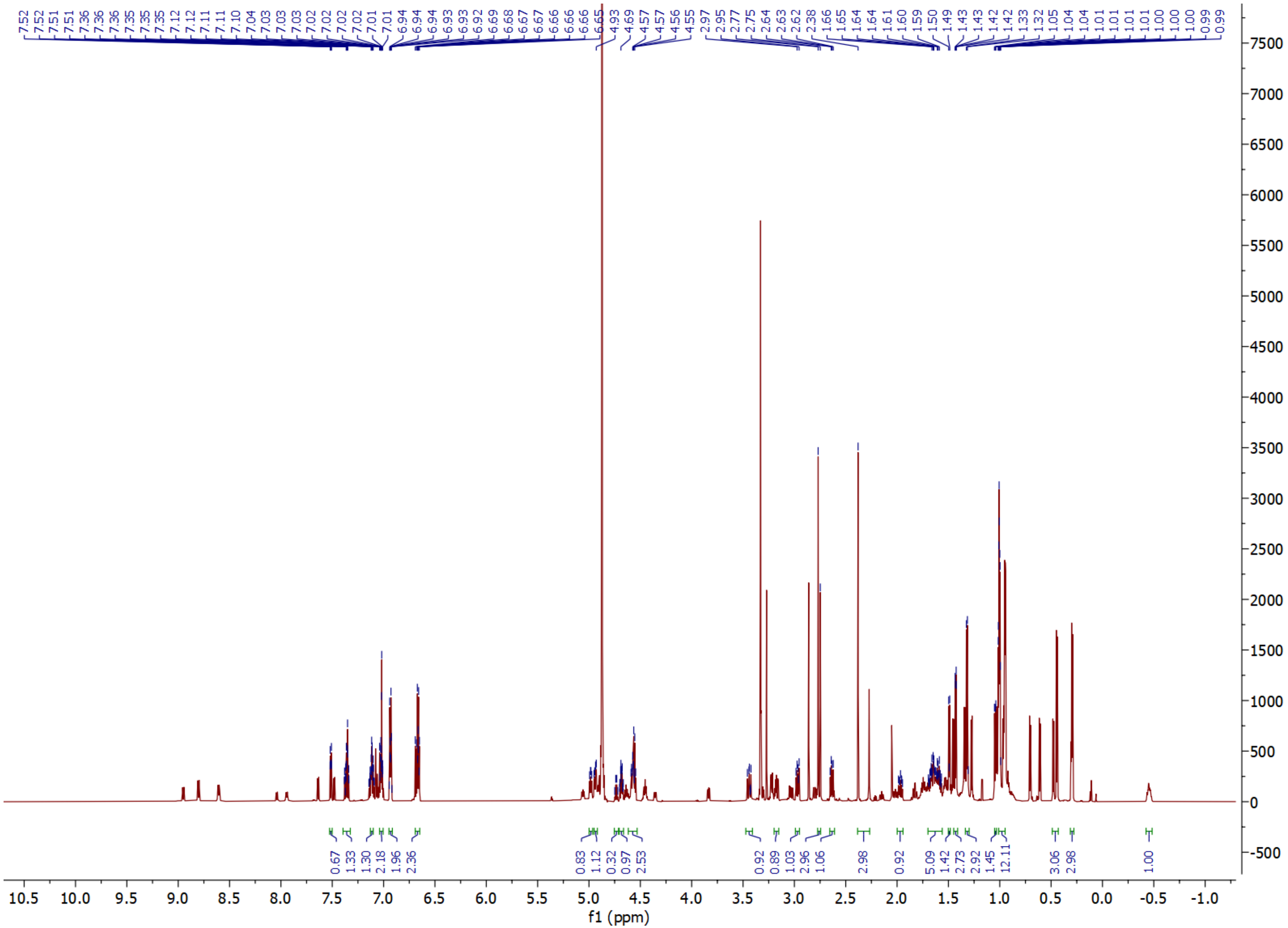

^{13}C NMR spectrum of rufomycin analogue **17** in CD_3OD at 176 MHz.

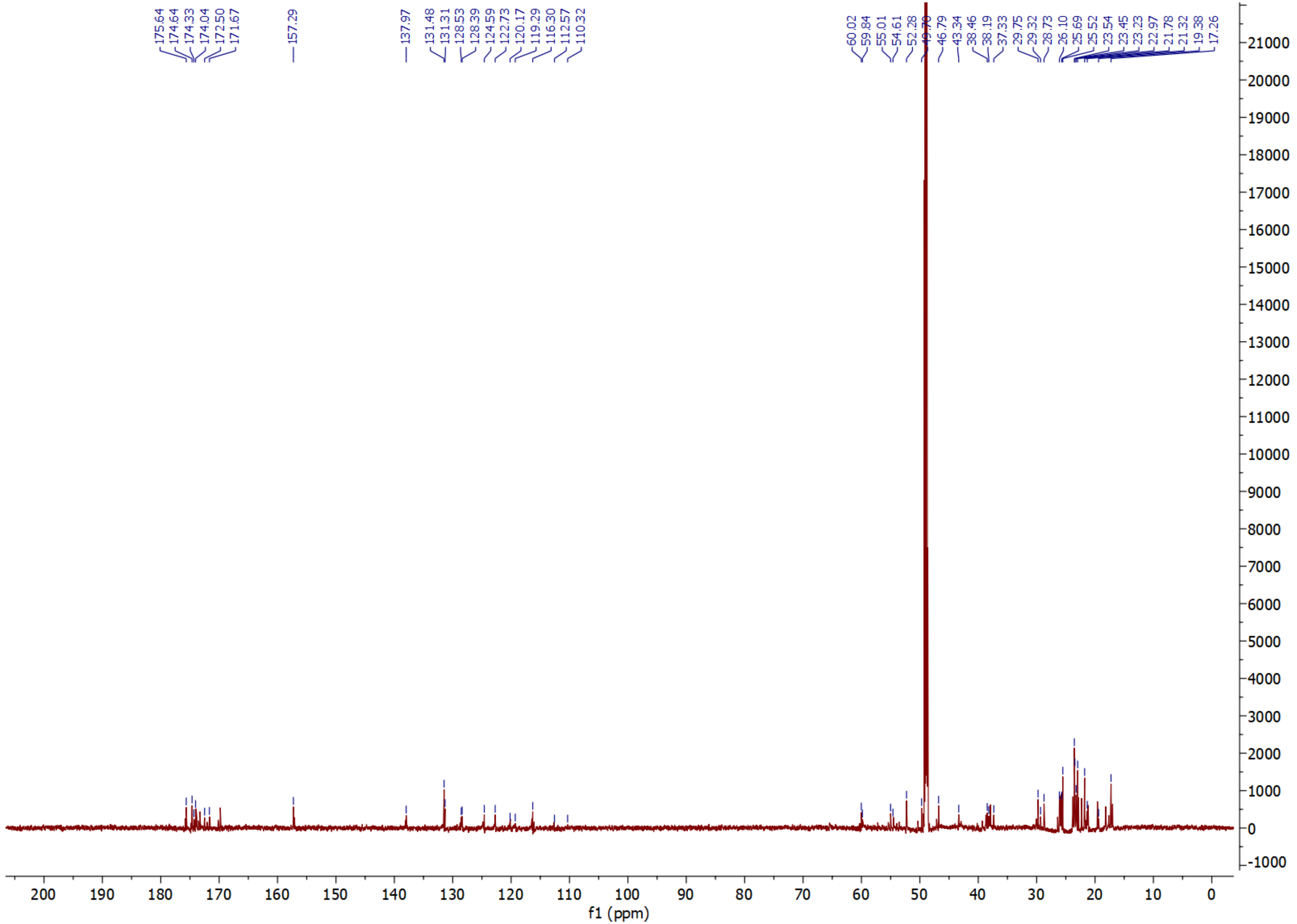

*COSY spectrum of rufomycin analogue **17** in CD_3OD.*

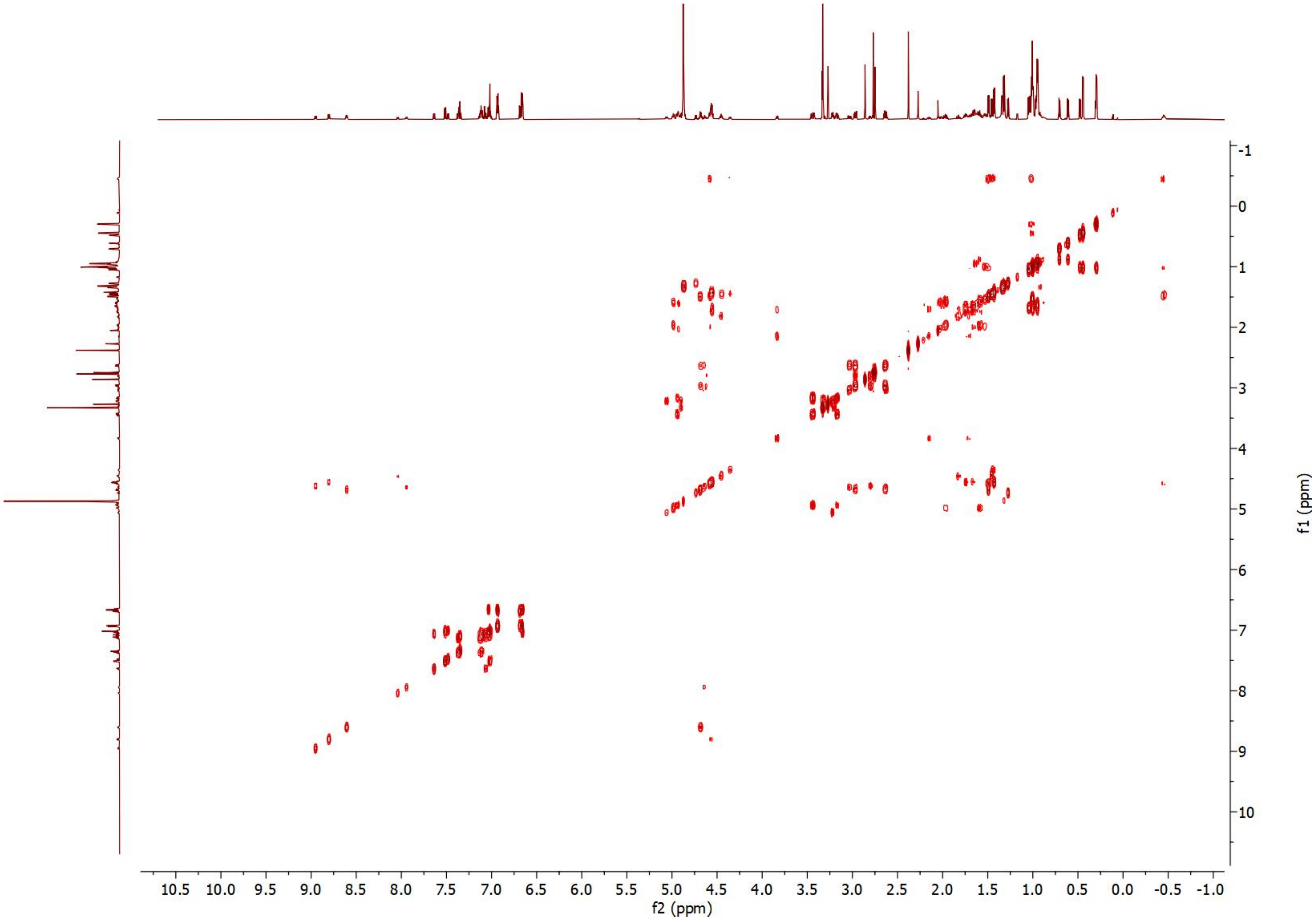

*HSQC spectrum of rufomycin analogue **17** in CD_3OD.*

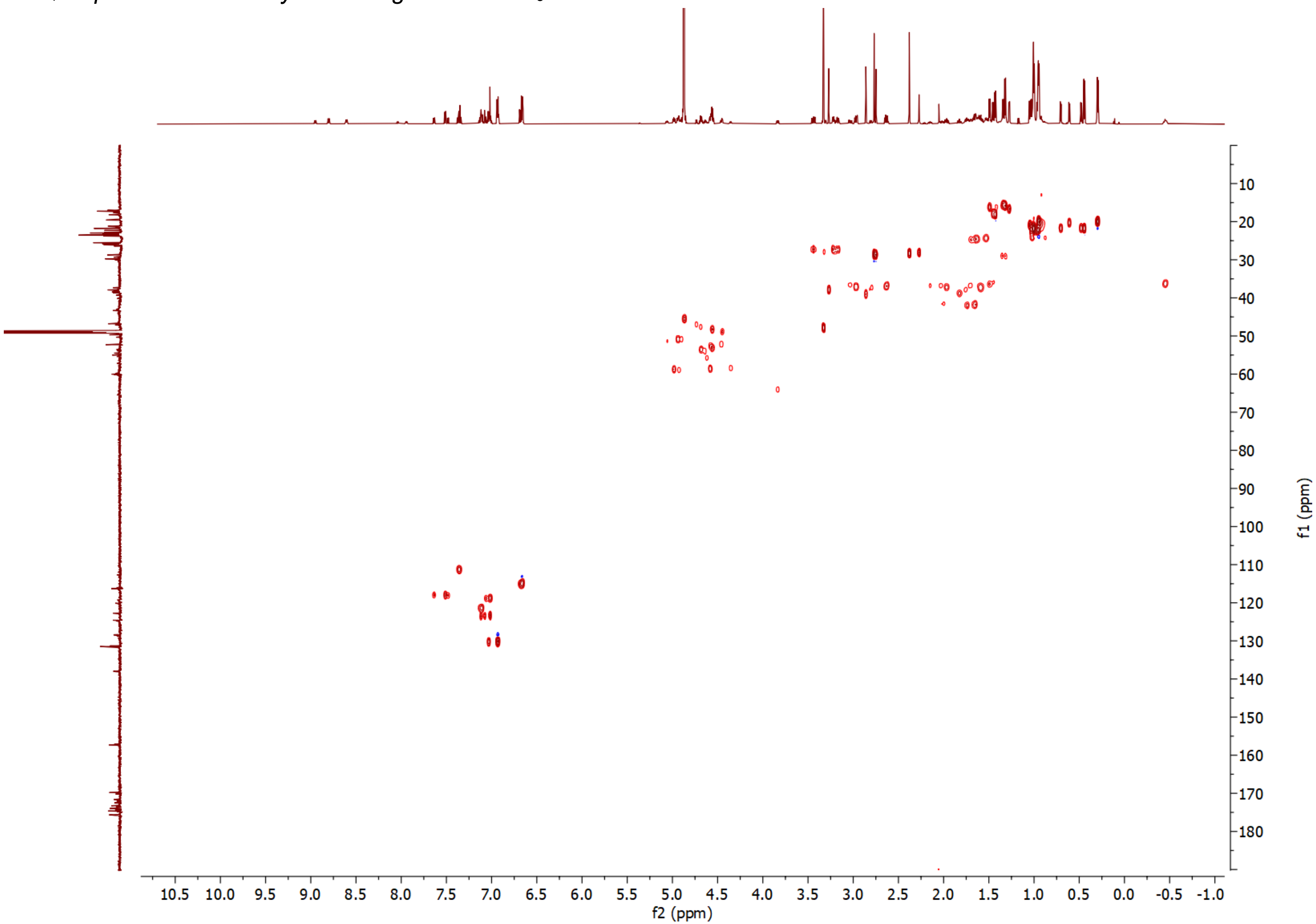

*HMBC spectrum of rufomycin analogue **17** in CD_3OD*

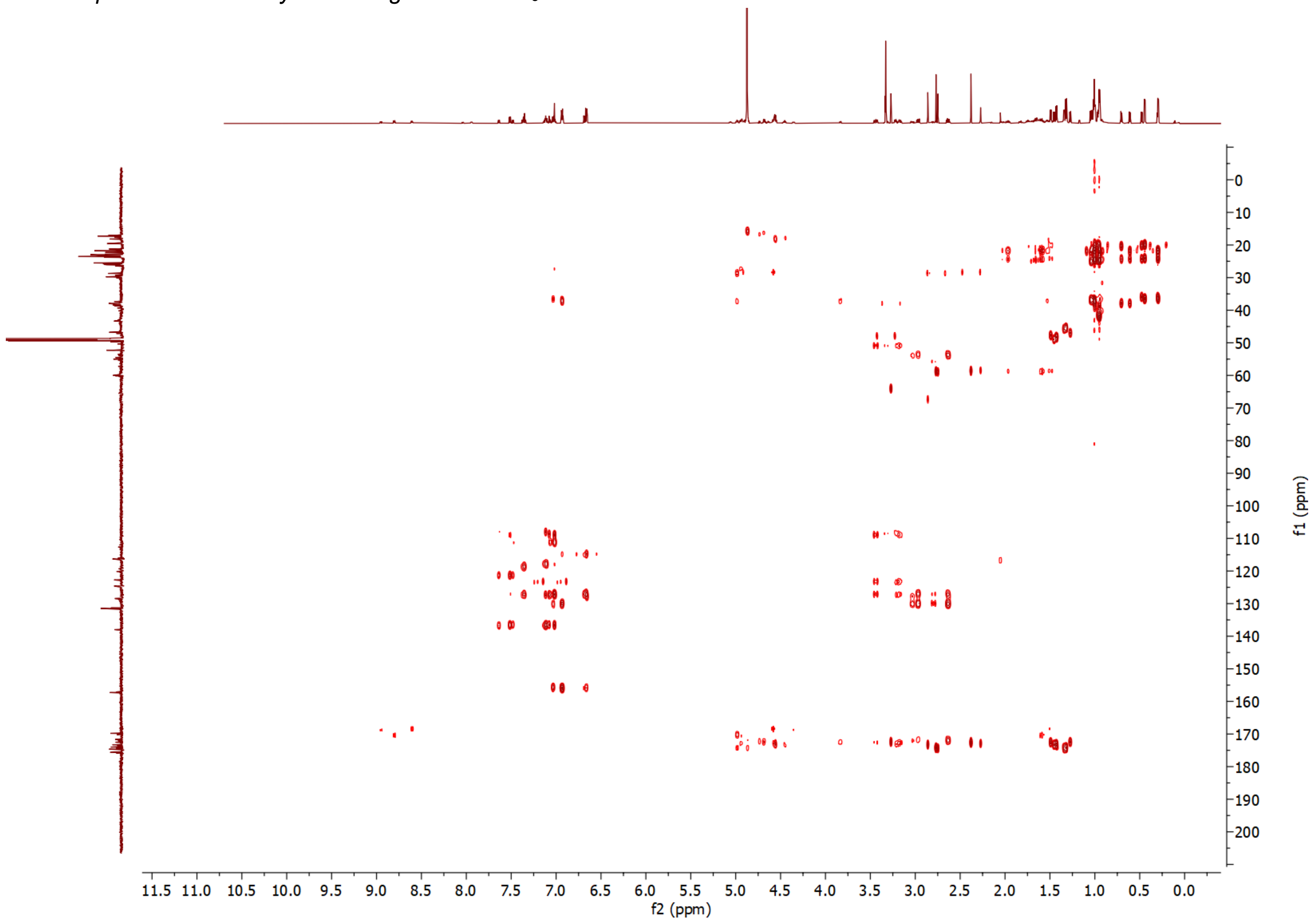

^{1}H NMR spectrum of rufomycin analogue **18** in $(CD_3)_2SO$ at 700 MHz.

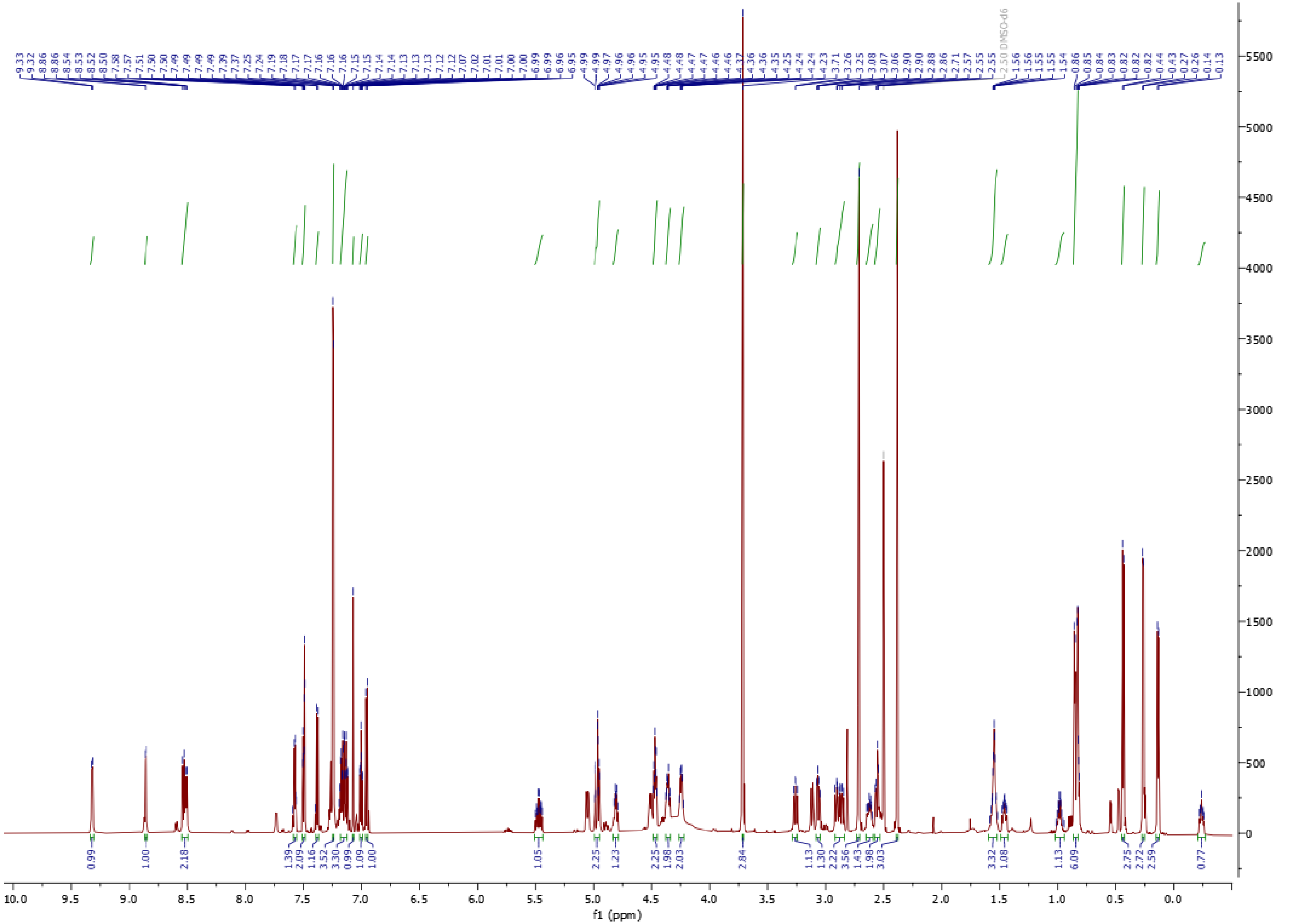

^{13}*C NMR spectrum of rufomycin analogue **18** in* $(CD_3)_2SO$ *at 151 MHz.*

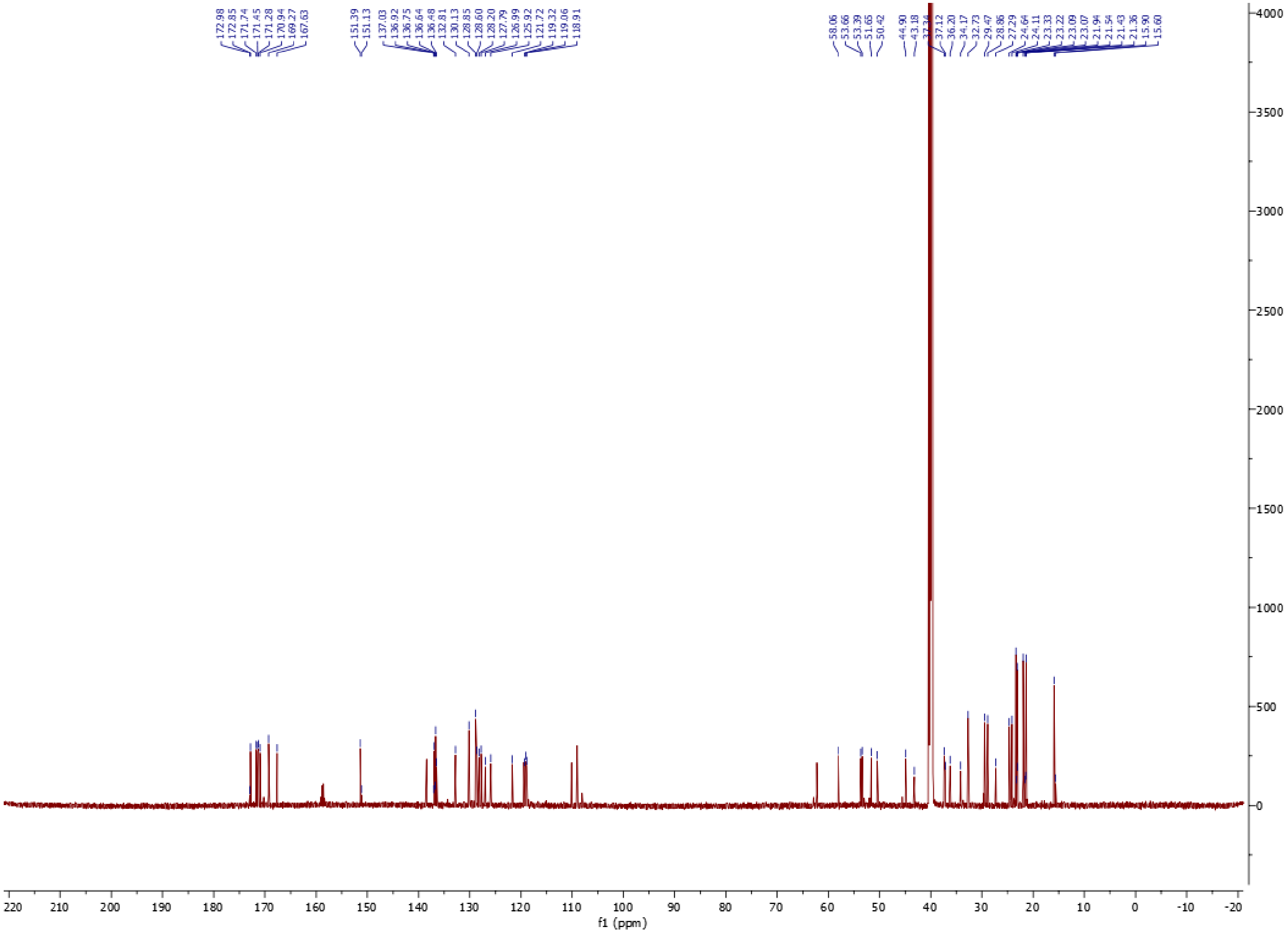

*COSY spectrum of rufomycin analogue **18** in $(CD_3)_2SO$*

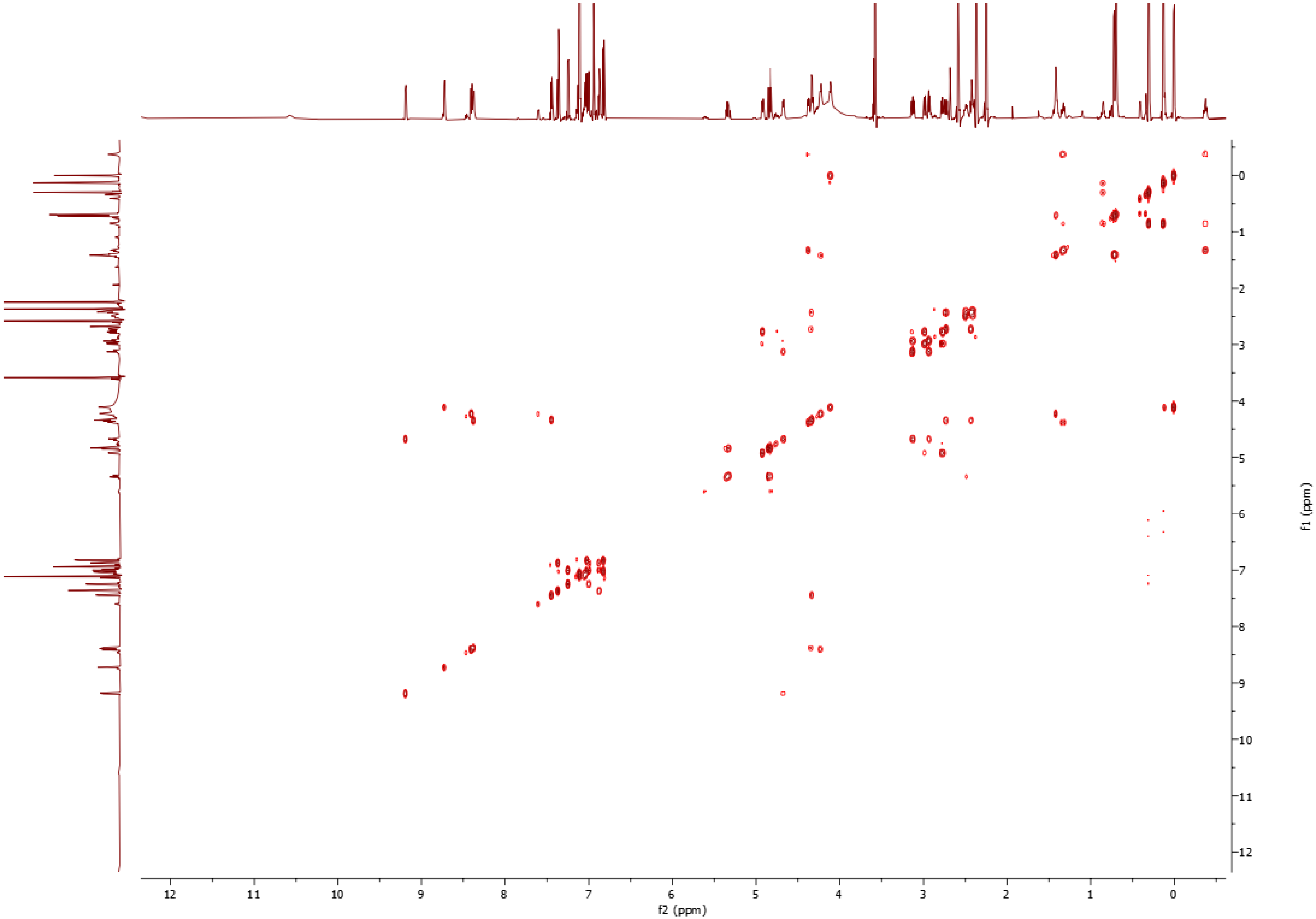

*HSQC spectrum of rufomycin analogue **18** in $(CD_3)_2SO$*

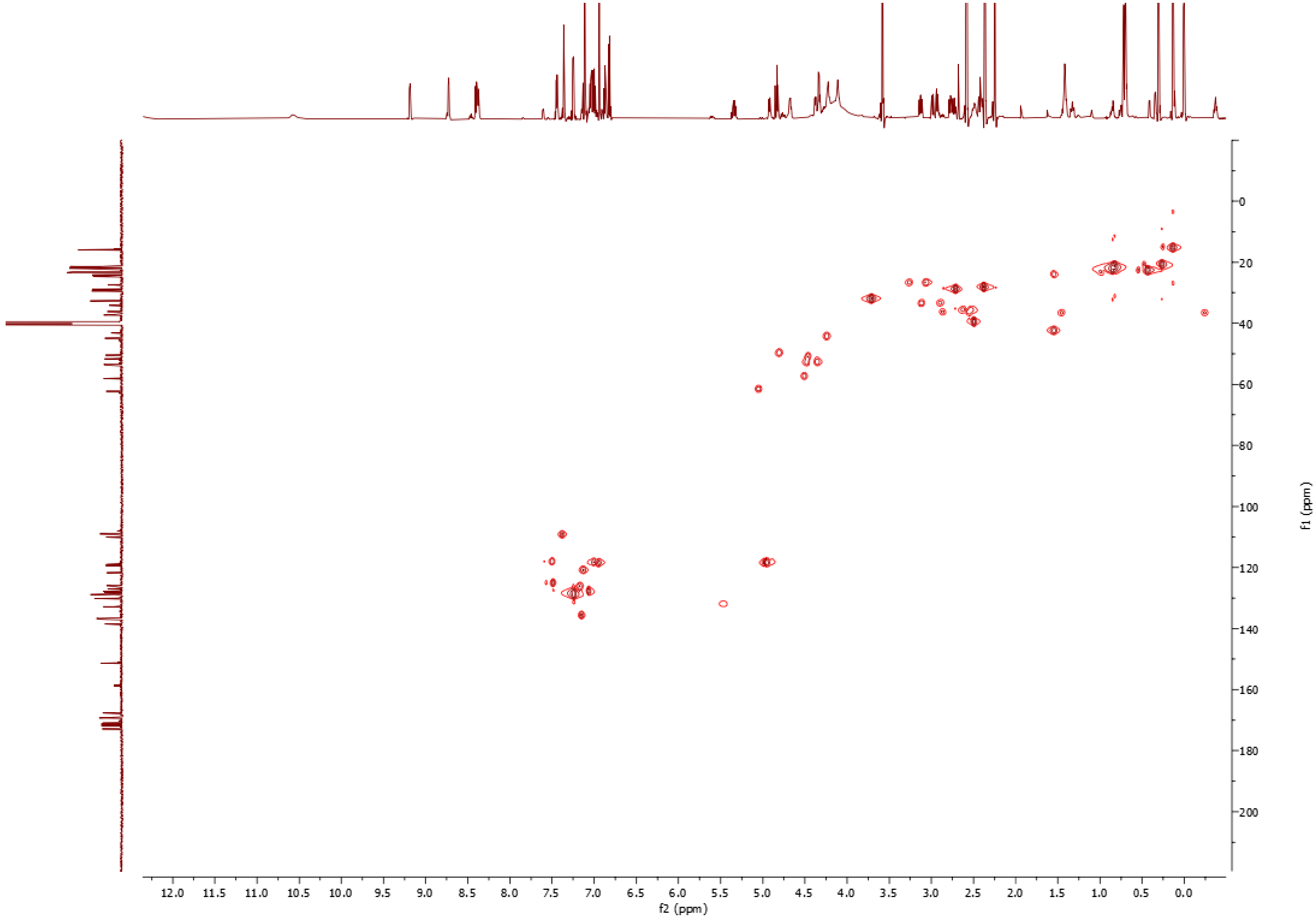

*HMBC spectrum of rufomycin analogue **18** in $(CD_3)_2SO$*

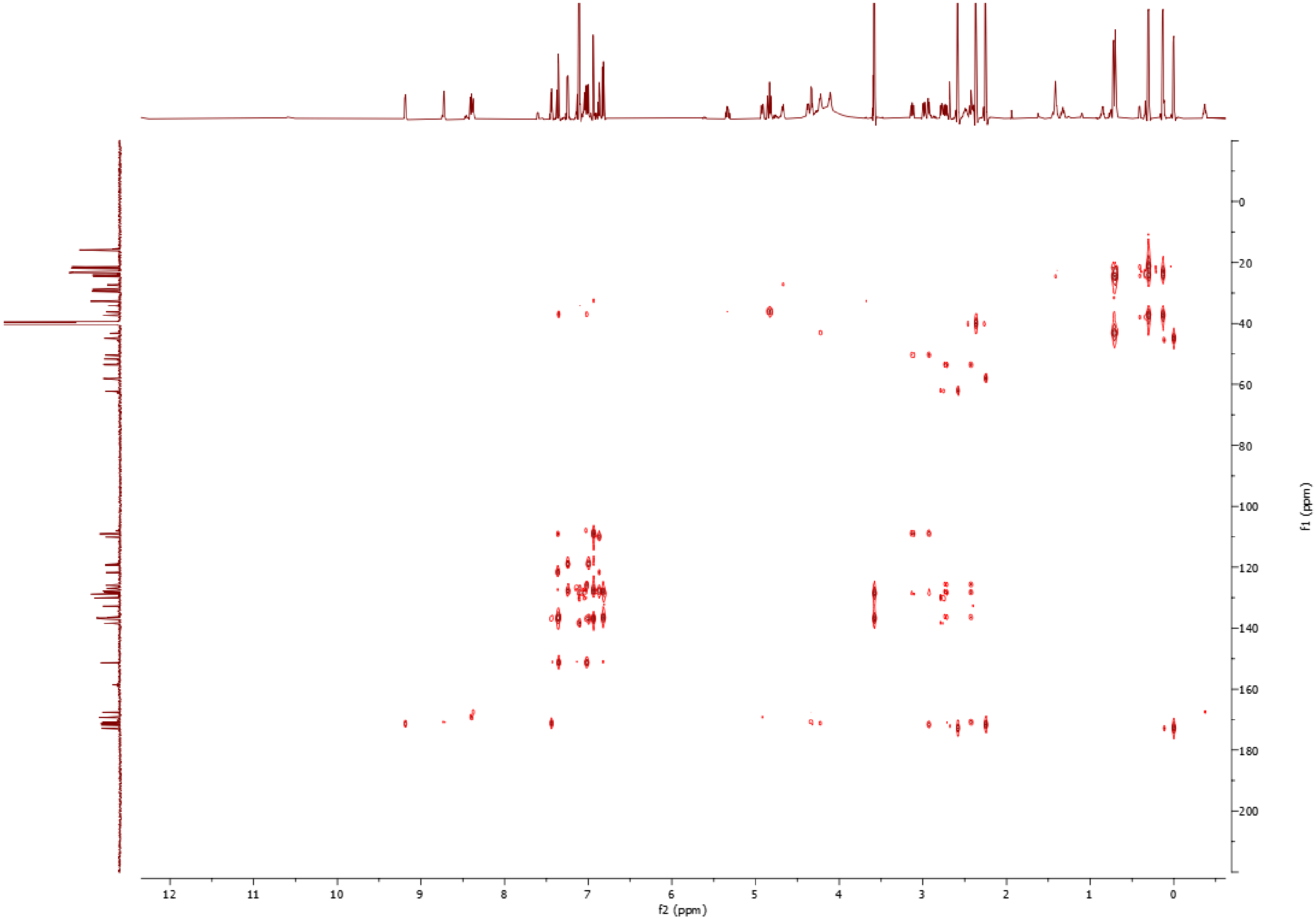

High Resolution Mass Spectra of Assay products.

HRMS extracted Ion Chromatogram of Peptide ***1*** *(top), predicted mass spectrum (middle) measured (bottom).*

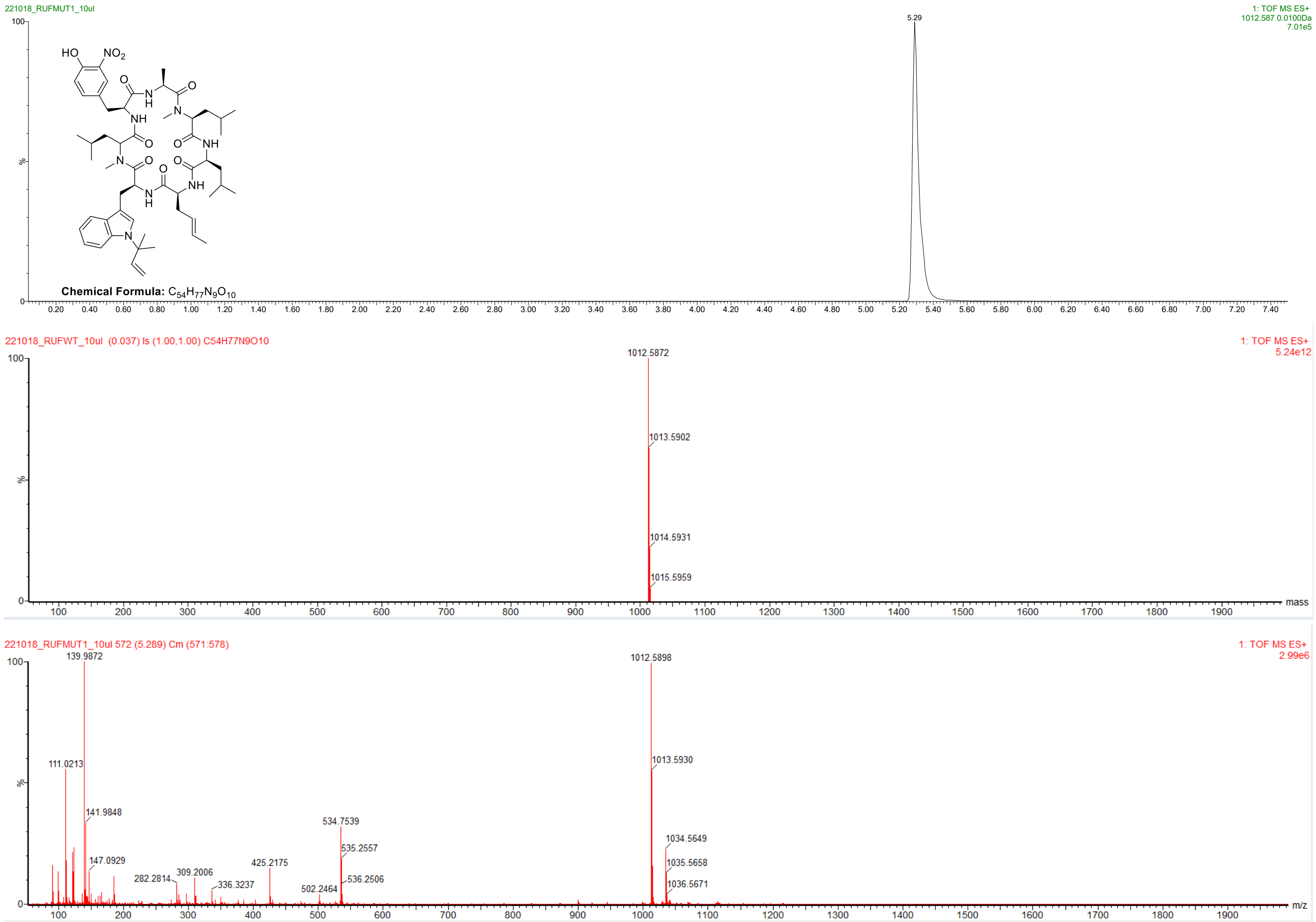

HRMS (ESI) calc. for $C_{54}H_{76}N_9O_{10}$ calc. for $[M+H]^+$: 1012.5872 m/z, found: 1012.5898 m/z.

*HRMS extracted Ion Chromatogram of Peptide **1a** (top), predicted mass spectrum (middle) measured mass spectrum (bottom).*

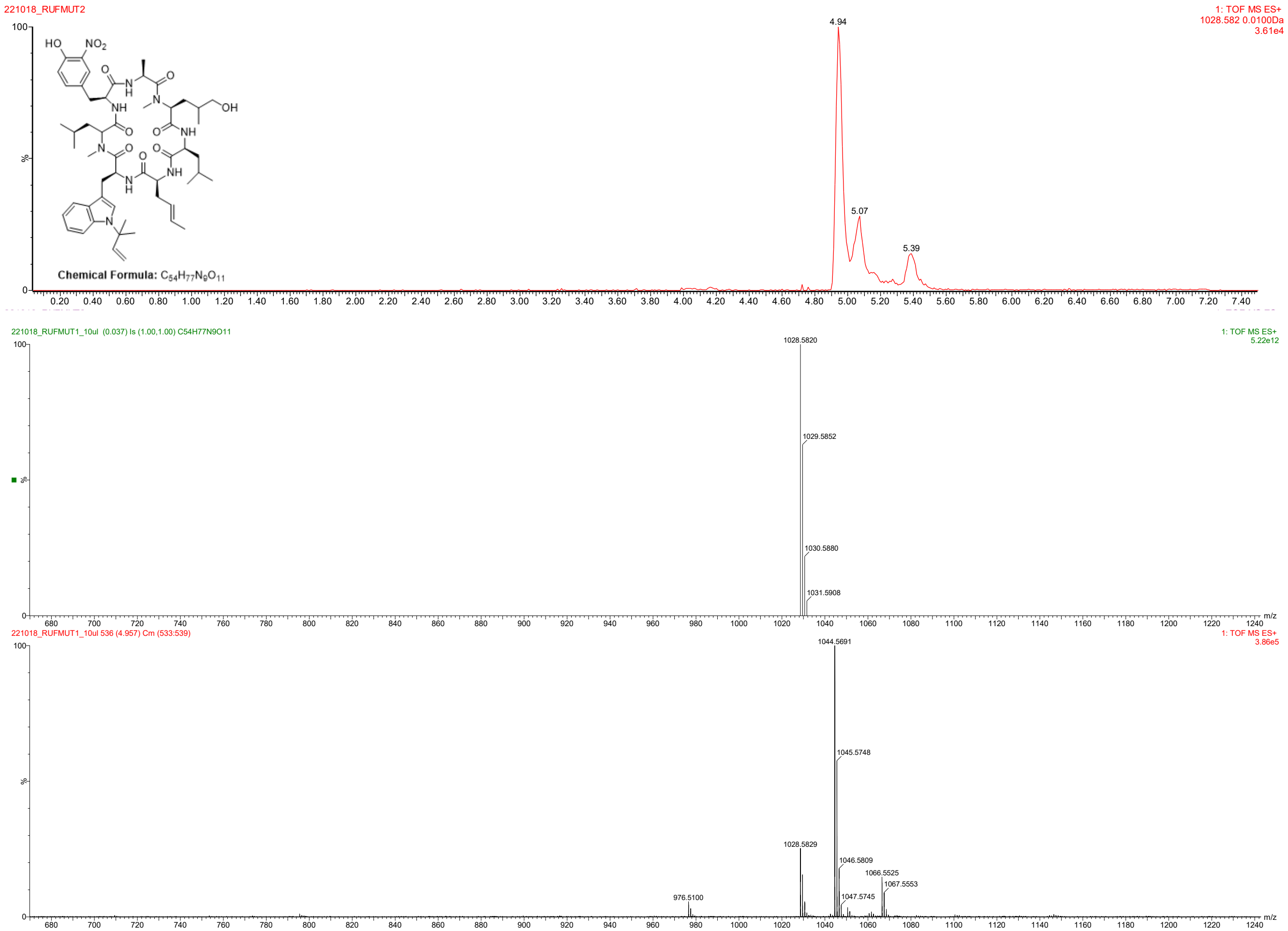

HRMS (ESI) calc. for $C_{54}H_{76}N_9O_{10}$ calc. for $[M+H]^+$: 1028.5820 m/z, found: 1028.5829 m/z.

*HRMS extracted Ion Chromatogram of Peptide **1b/b*** (top), predicted mass spectrum (middle) measured mass spectrum (bottom).*

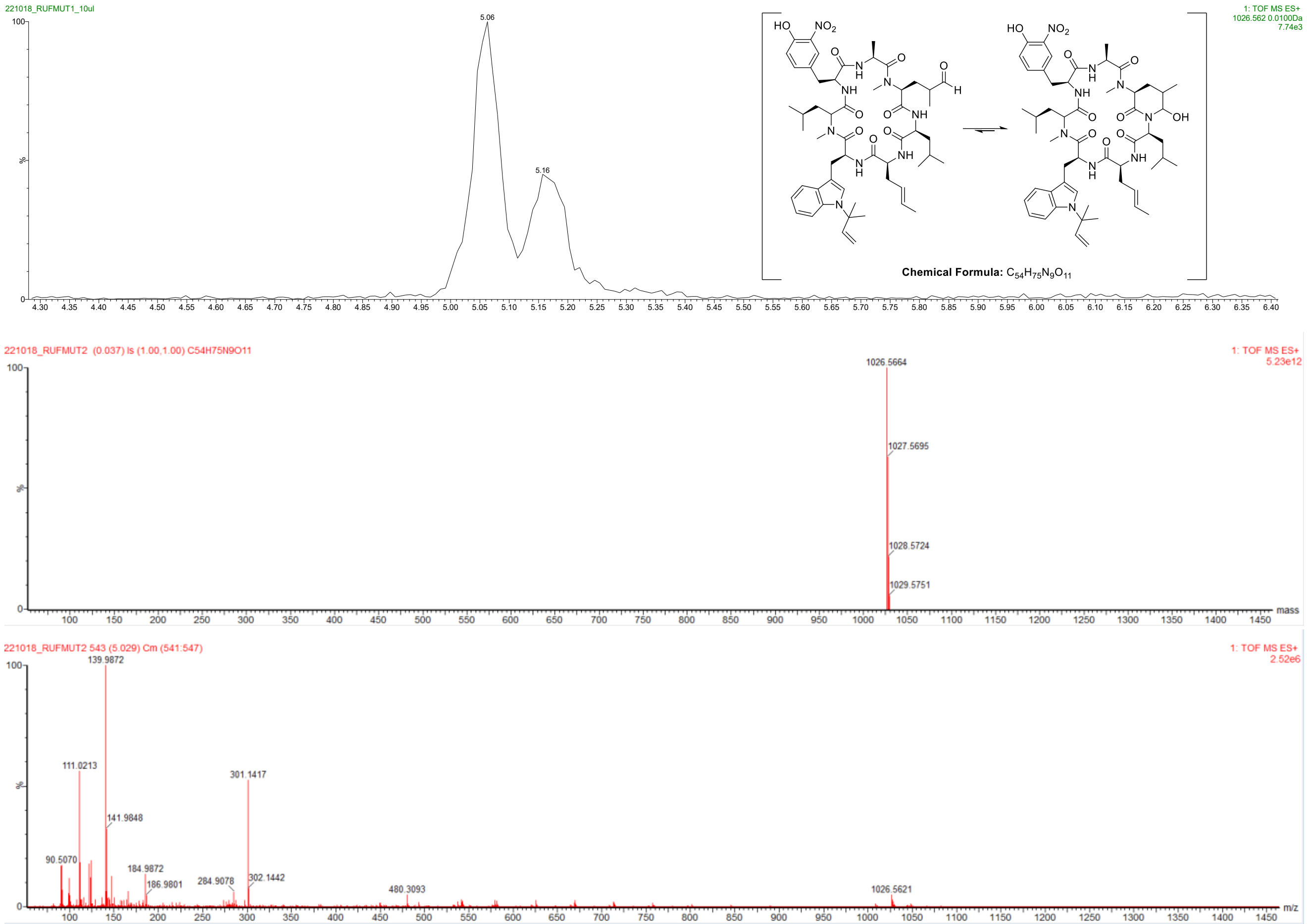

HRMS (ESI) calc. for $C_{54}H_{75}N_9O_{11}$ calc. for $[M+H]^+$: 1026.5664 m/z, found: 1026.5621 m/z.

*HRMS extracted Ion Chromatogram of Peptide **1d** (top), predicted mass spectrum (middle) measured mass spectrum (bottom).*

.

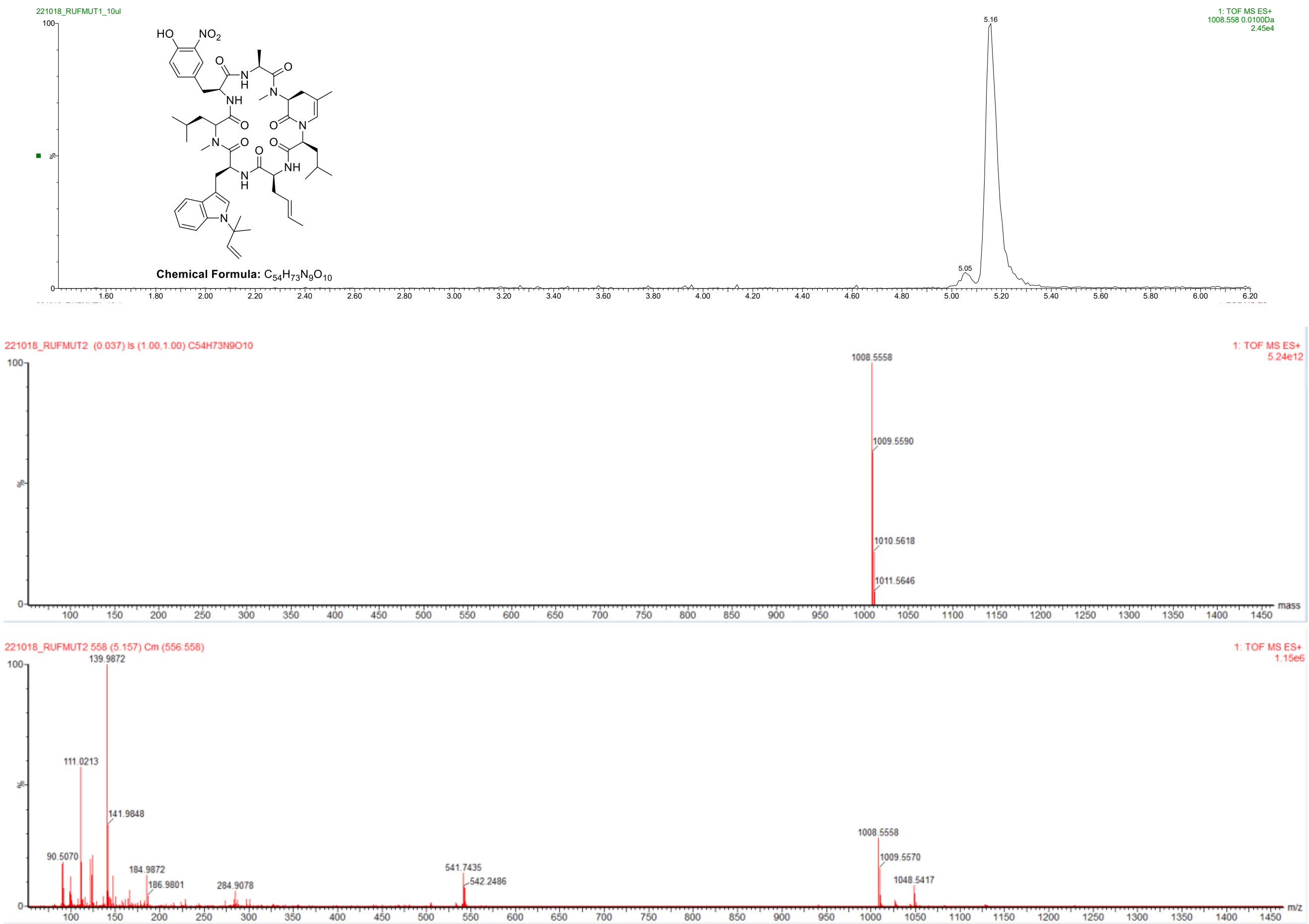

HRMS (ESI) calc. for $C_{54}H_{73}N_9O_{10}$ calc. for $[M+H]^+$: 1008.5558 m/z, found: 1008.5558 m/z (*Δ* = 0 ppm)

.

*HRMS extracted Ion Chromatogram of Peptide **1c** (top), predicted mass spectrum (middle) measured mass spectrum (bottom).*

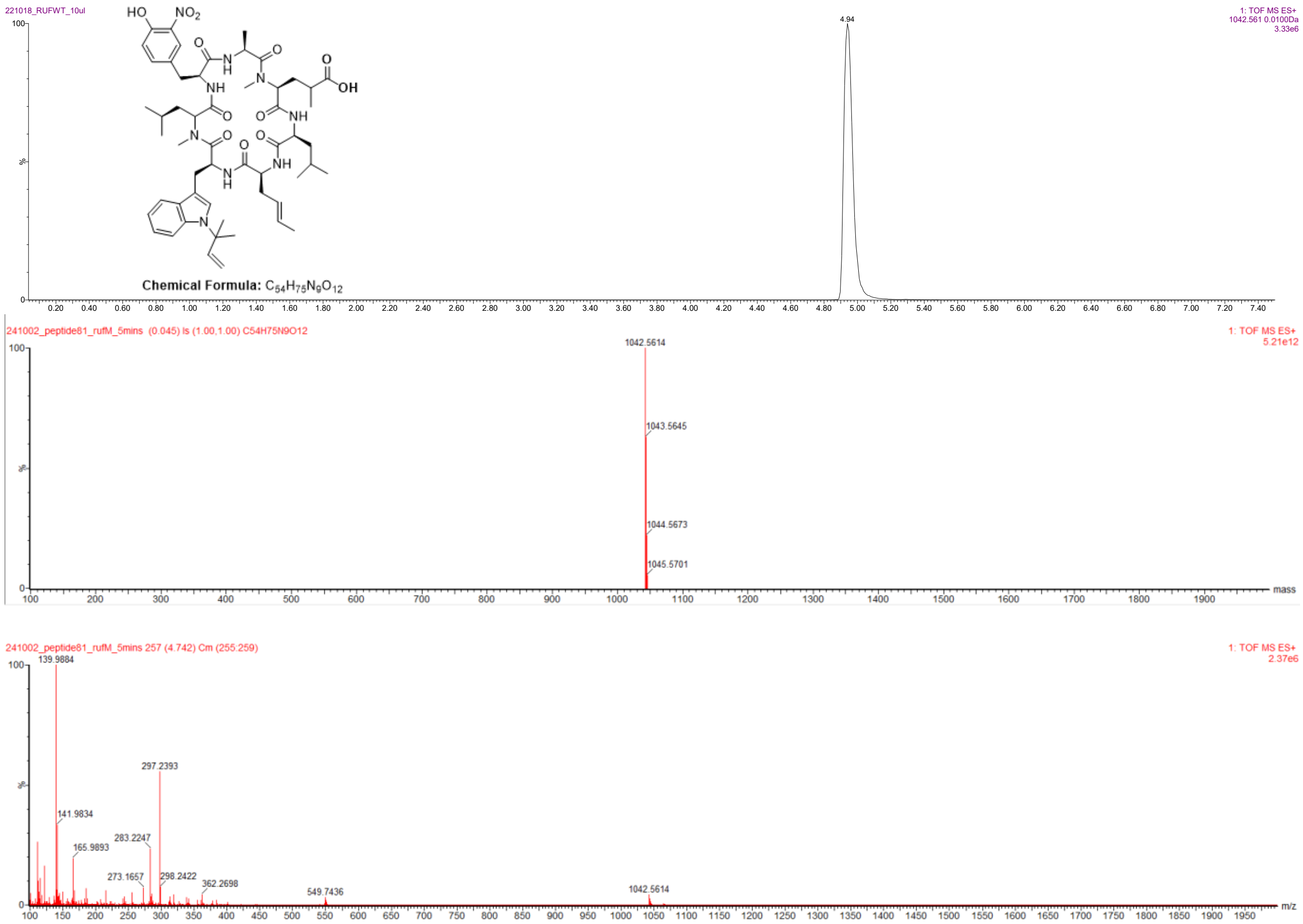

HRMS (ESI) calc. for $C_{54}H_{75}N_9O_{12}$ calc. for $[M+H]^+$: 1042.5614 m/z, found: 1042.5614 m/z. (Δ = 0 ppm)

*HRMS extracted Ion Chromatogram of Peptide **2**(top), predicted mass spectrum (middle) measured mass spectrum (bottom).*

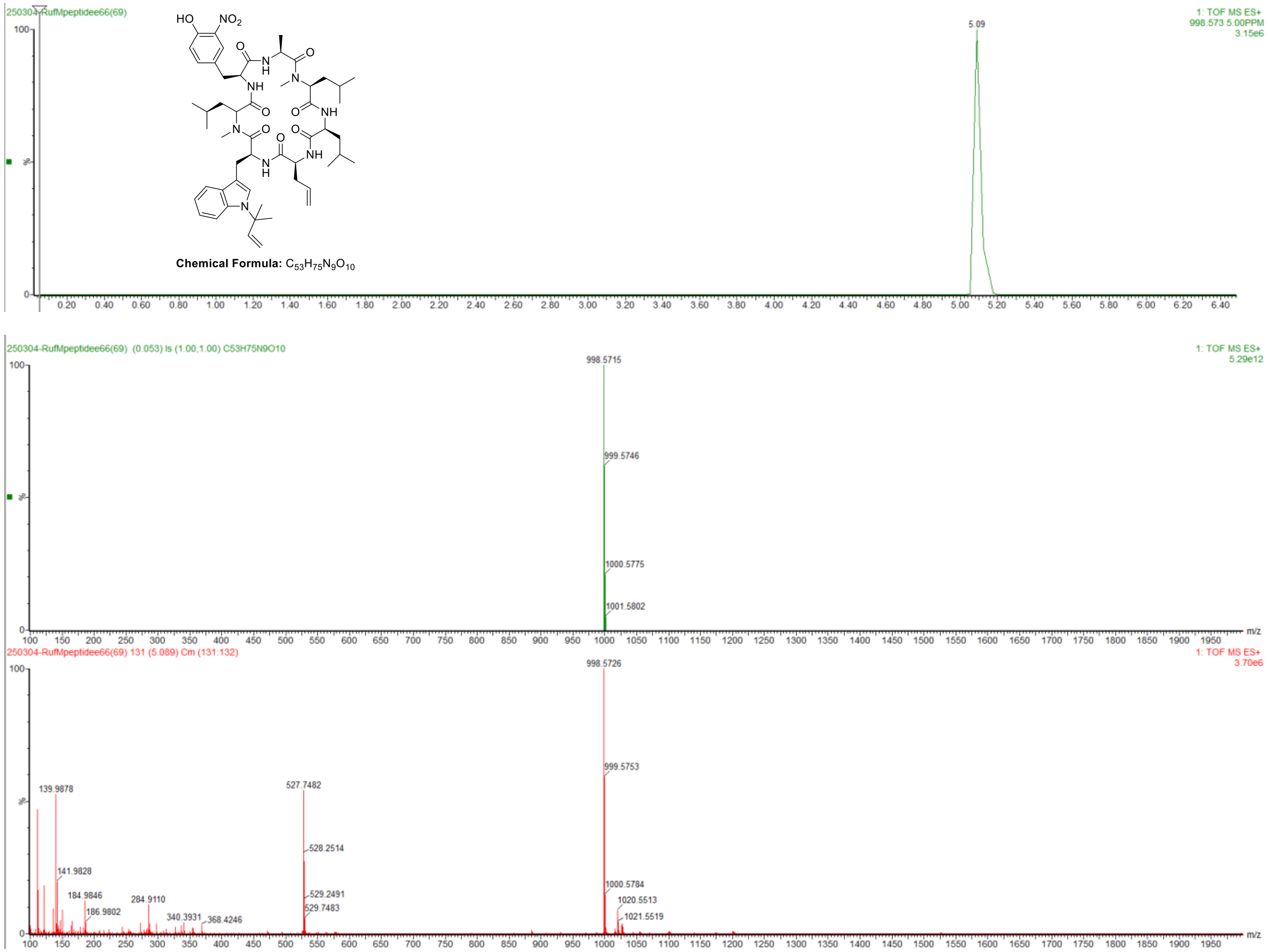

HRMS (ESI) calc. for $C_{53}H_{72}N_9O_{10}$ calc. for $[M+H]^+$: 998.5715 m/z, found: 998.5726 m/z.

*HRMS extracted Ion Chromatogram of Peptide **2a**(top), predicted mass spectrum (middle) measured mass spectrum (bottom).*

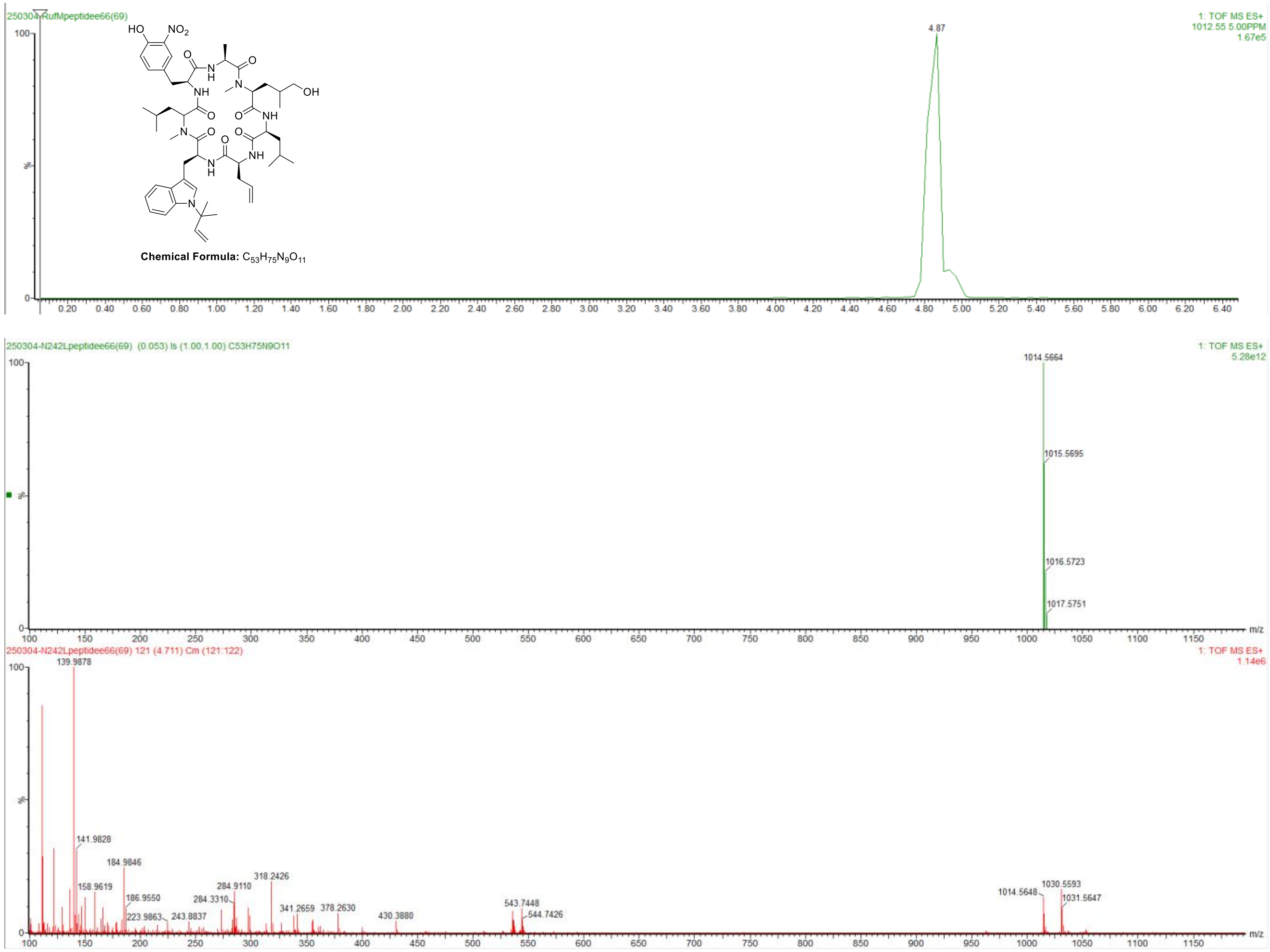

HRMS (ESI) calc. for $C_{53}H_{72}N_9O_{10}$ calc. for $[M+H]^+$: 1014.5664 m/z, found: 1014.5648 m/z.

*HRMS extracted Ion Chromatogram of Peptide **2b/b**** *(top), predicted mass spectrum (middle) measured mass spectrum (bottom).*

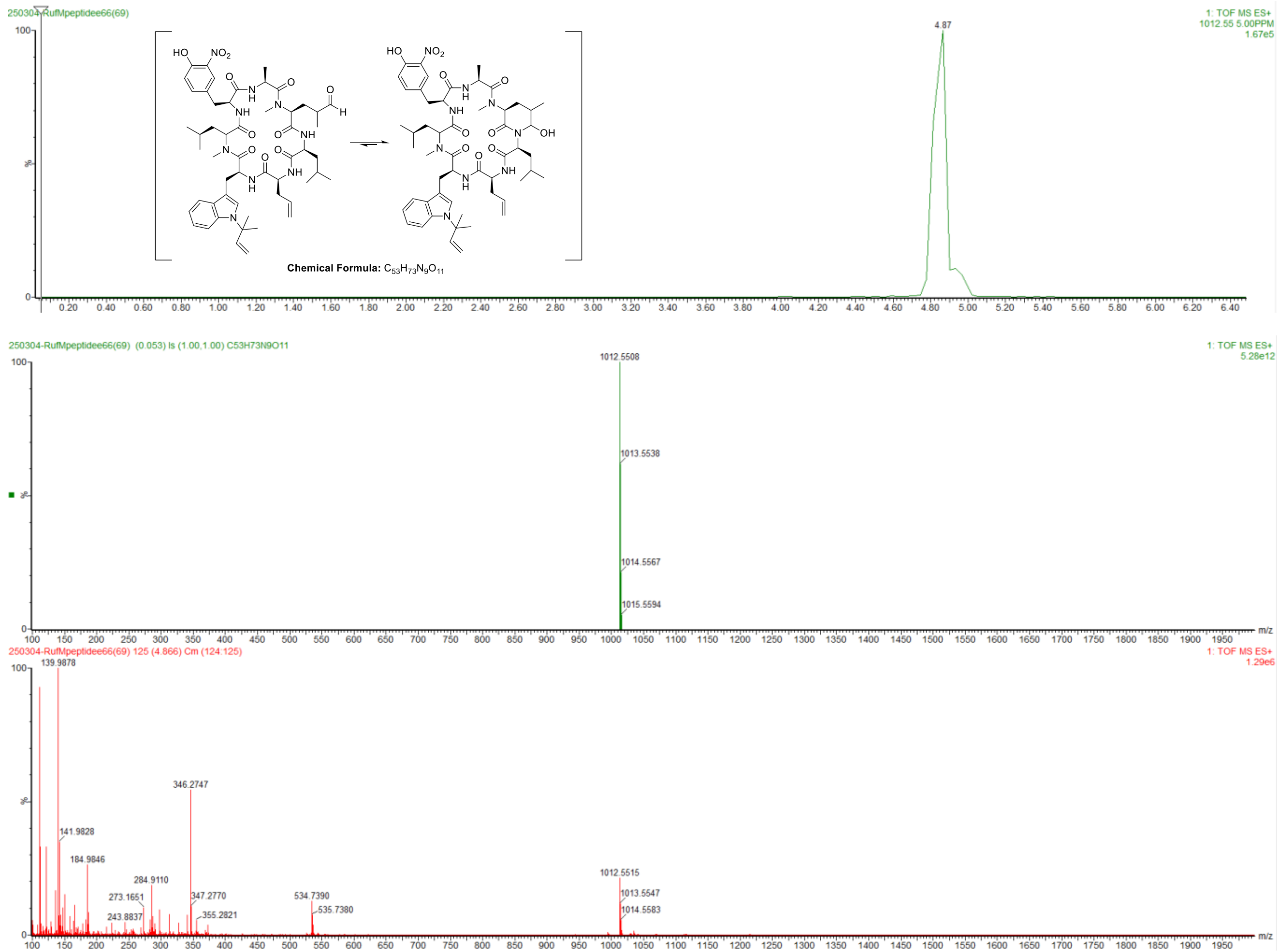

HRMS (ESI) calc. for $C_{53}H_{72}N_9O_{10}$ calc. for $[M+H]^+$: 1012.5508 m/z, found: 1012.5515 m/z.

*HRMS extracted Ion Chromatogram of Peptide **2d** (top), predicted mass spectrum (middle) measured mass spectrum (bottom).*

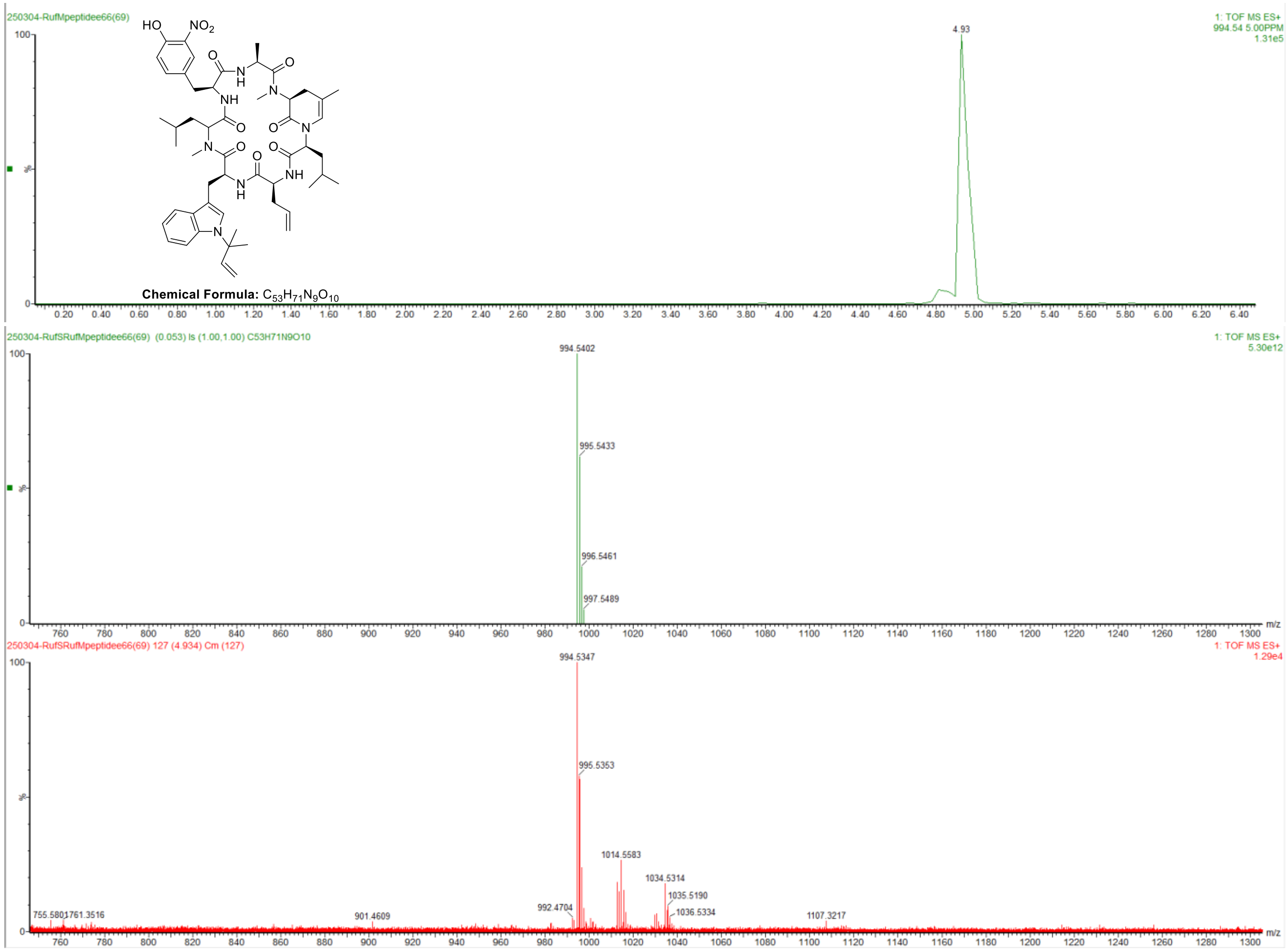

HRMS (ESI) calc. for $C_{53}H_{72}N_9O_{10}$ calc. for $[M+H]^+$: 994.5402 m/z, found: 994. 5347 m/z.

*HRMS extracted Ion Chromatogram of Peptide **2c** (top), predicted mass spectrum (middle) measured mass spectrum (bottom).*

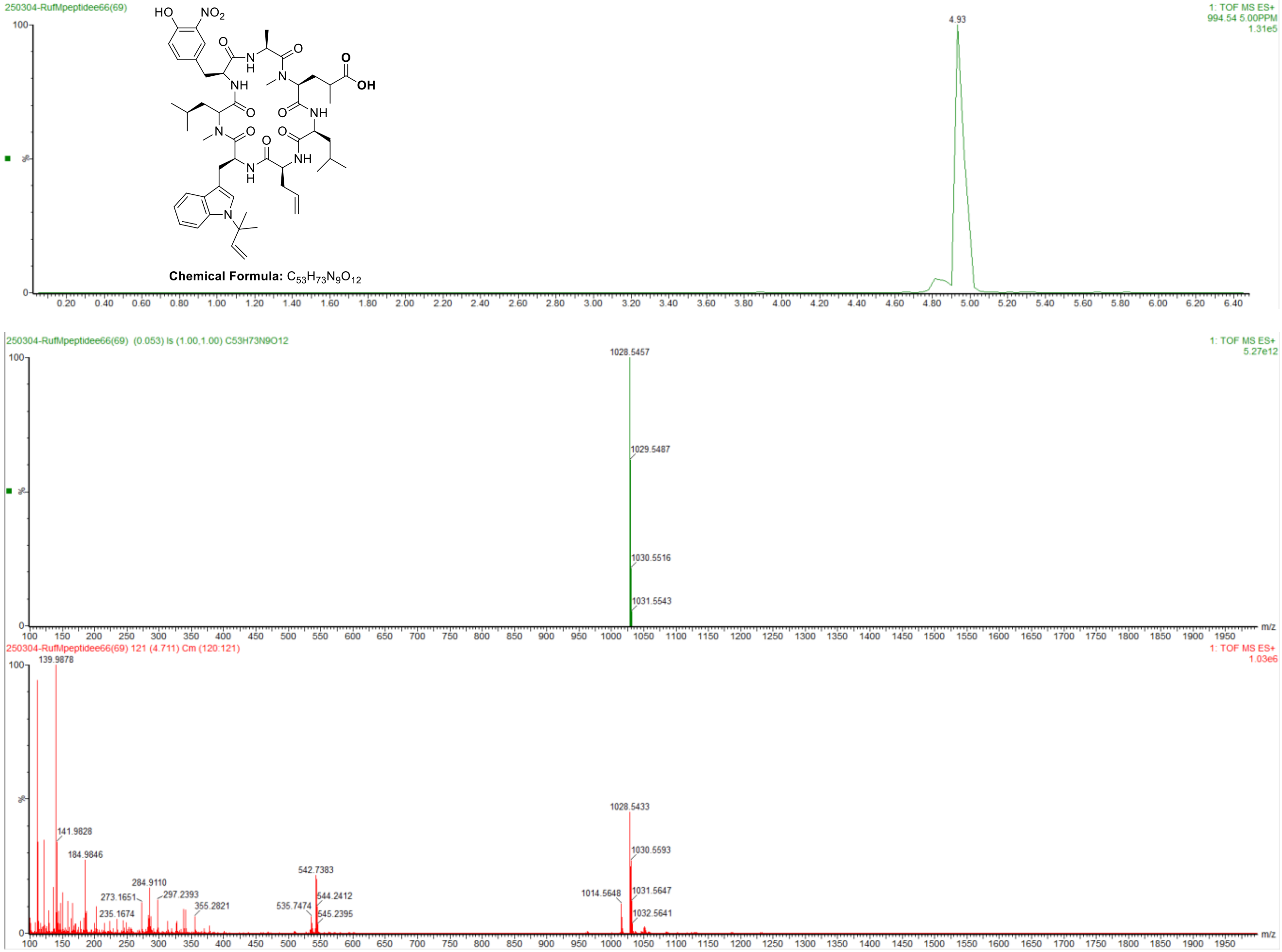

HRMS (ESI) calc. for $C_{53}H_{72}N_9O_{10}$ calc. for $[M+H]^+$: 1028.5457 m/z, found: 1028.5433 m/z.

*HRMS extracted Ion Chromatogram of Peptide **epoxy2** (top), predicted mass spectrum (middle) measured mass spectrum (bottom).*

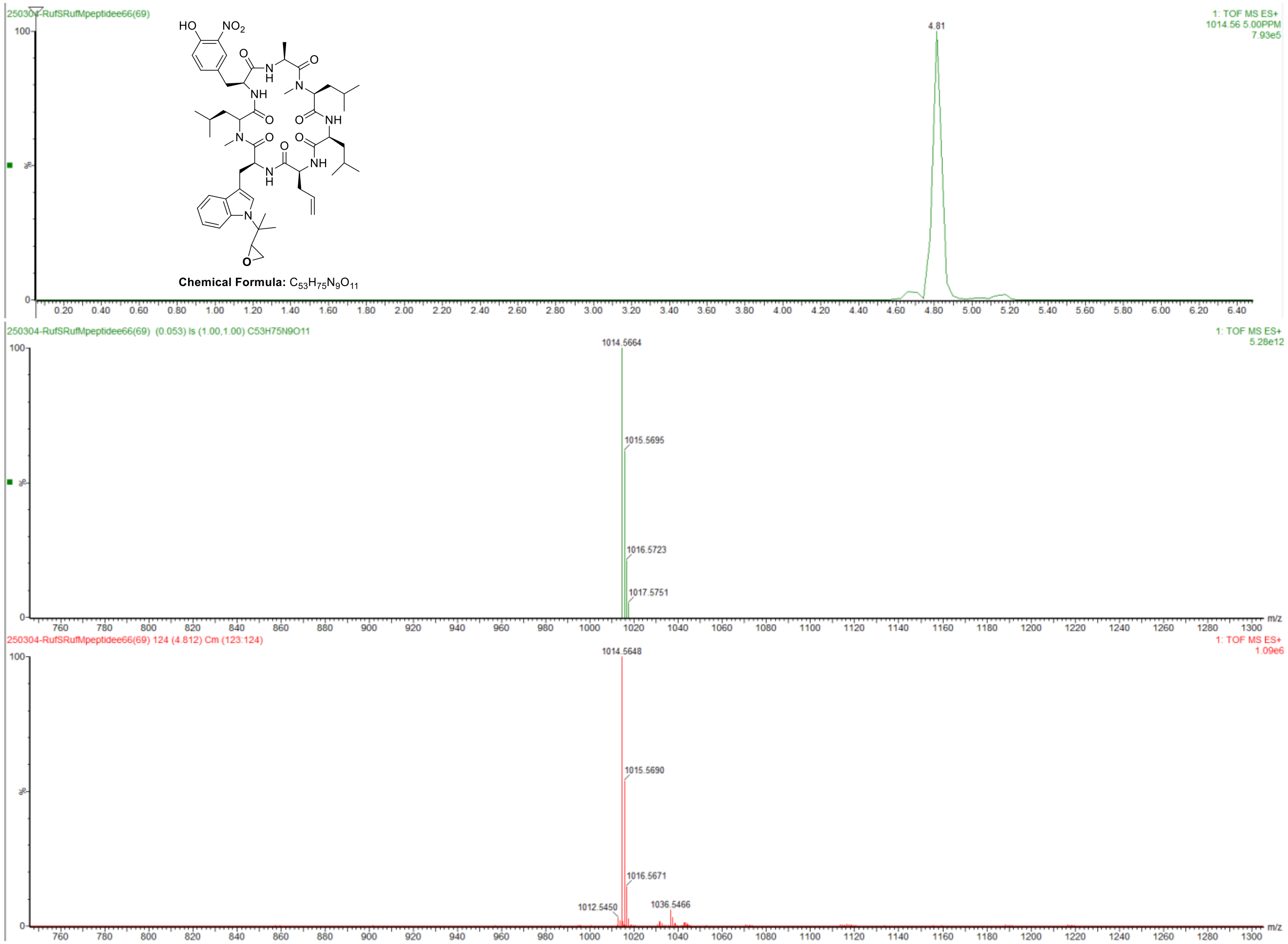

HRMS (ESI) calc. for $C_{53}H_{72}N_9O_{10}$ calc. for $[M+H]^+$: 1014.5664 m/z, found: 1014.5648 m/z.

HRMS extracted Ion Chromatogram of Peptide ***epoxy2a*** *(top), predicted mass spectrum (middle) measured mass spectrum (bottom).*

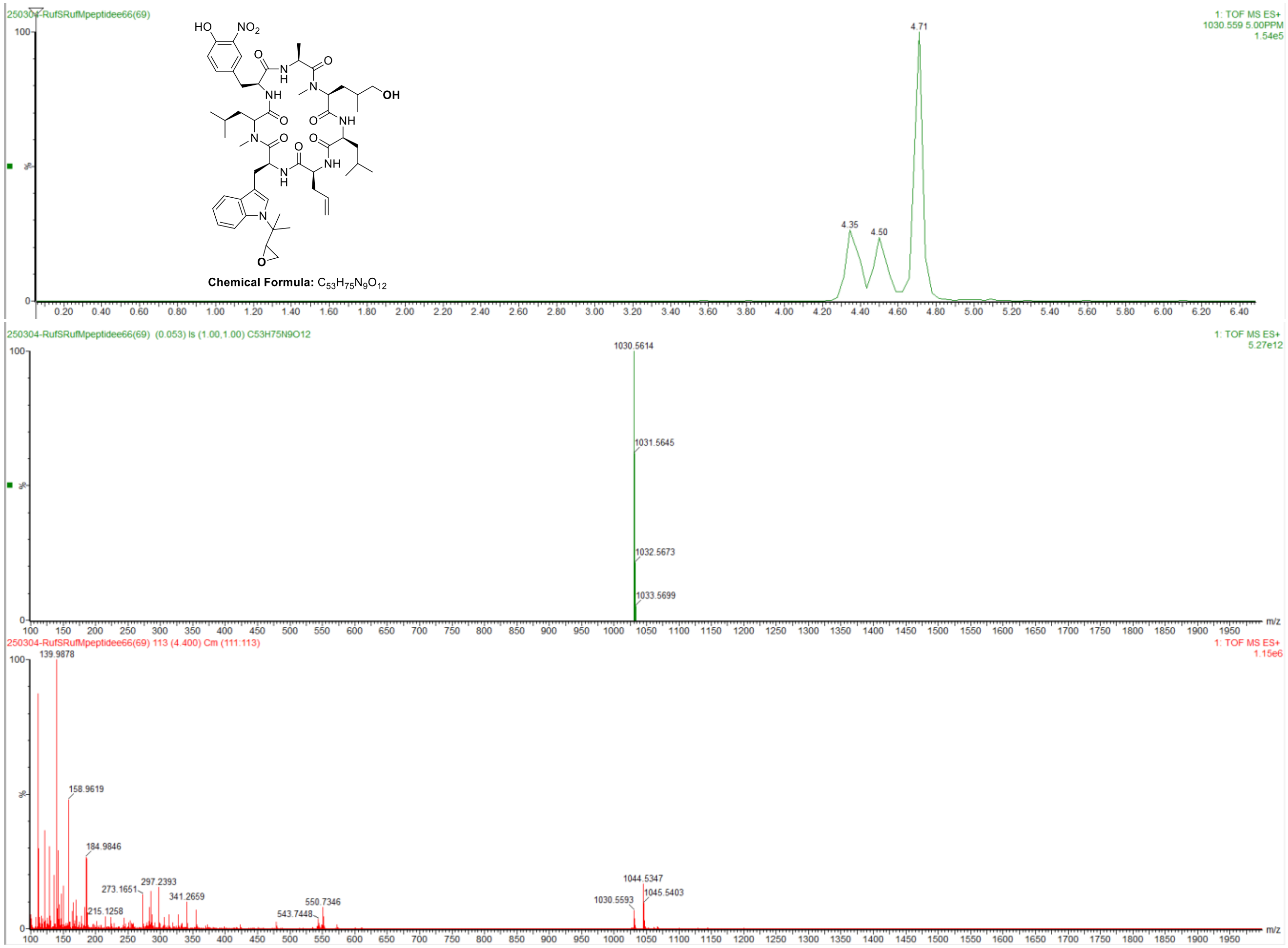

HRMS (ESI) calc. for $C_{53}H_{72}N_9O_{10}$ calc. for $[M+H]^+$: 1030.5614 m/z, found: 1030.5593 m/z.

*HRMS extracted Ion Chromatogram of Peptide **epoxy2b/b*** (top), predicted mass spectrum (middle) measured mass spectrum (bottom).*

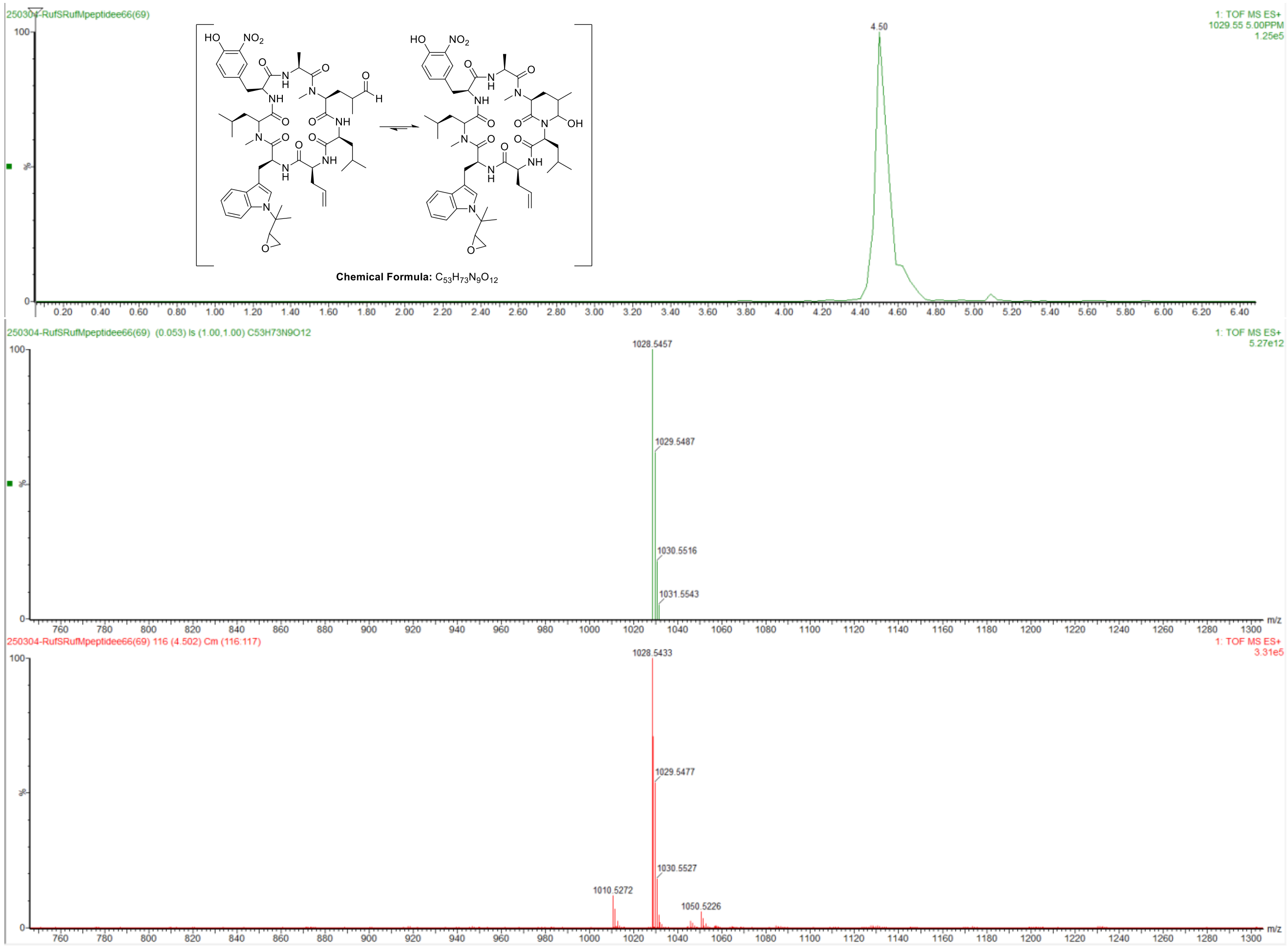

HRMS (ESI) calc. for $C_{53}H_{72}N_9O_{10}$ calc. for $[M+H]^+$: 1028.5457 m/z, found: 1028.5433 m/z.

*HRMS extracted Ion Chromatogram of Peptide **epoxy2d** (top), predicted mass spectrum (bottom) measured mass spectrum (middle).*

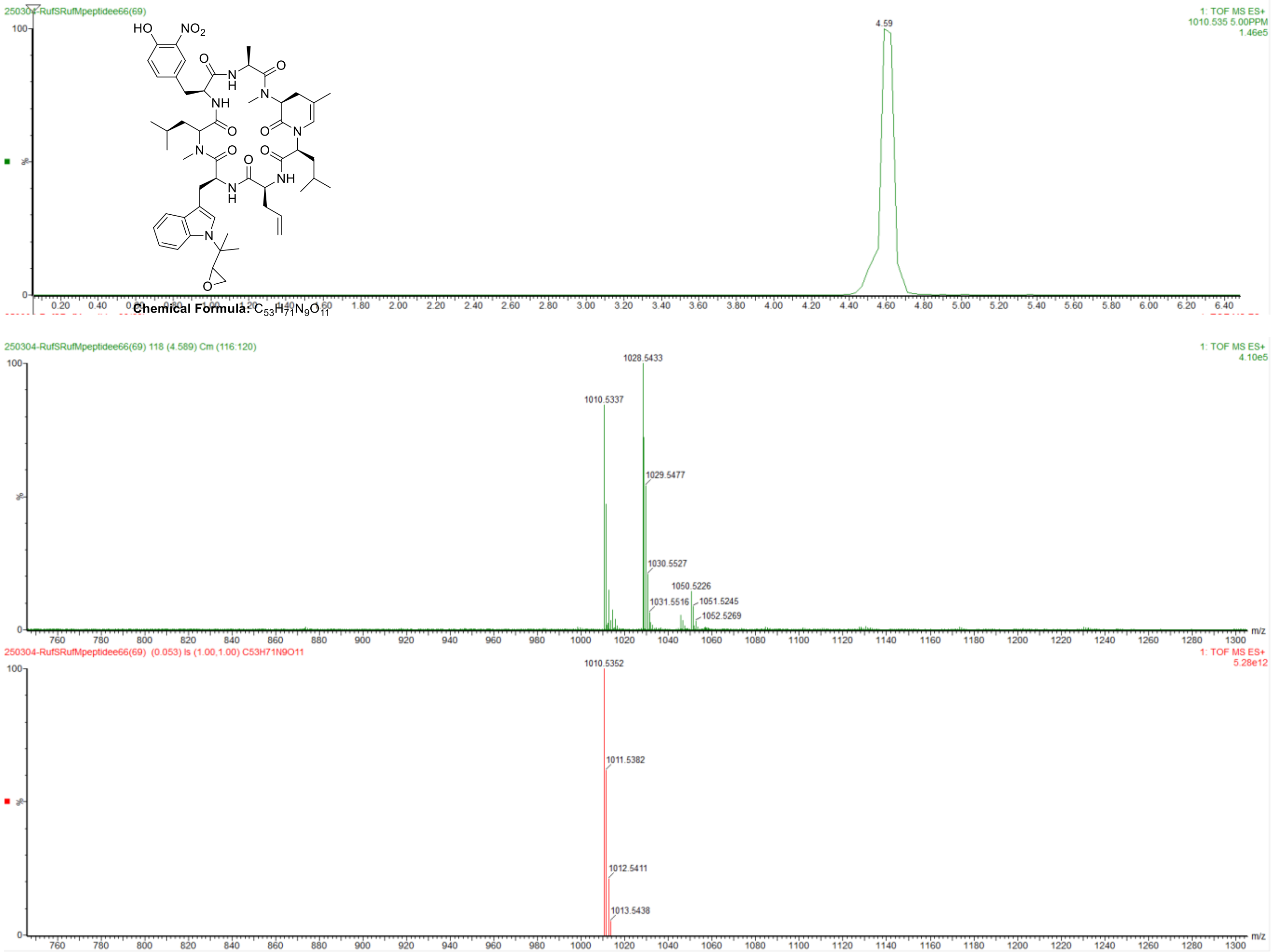

HRMS (ESI) calc. for $C_{53}H_{71}N_9O_{11}$ calc. for $[M+H]^+$: 1010.5352 m/z, found: 1010.5337 m/z.

HRMS extracted Ion Chromatogram of Peptide ***epoxy2c*** *(top), predicted mass spectrum (middle) measured mass spectrum (bottom).*

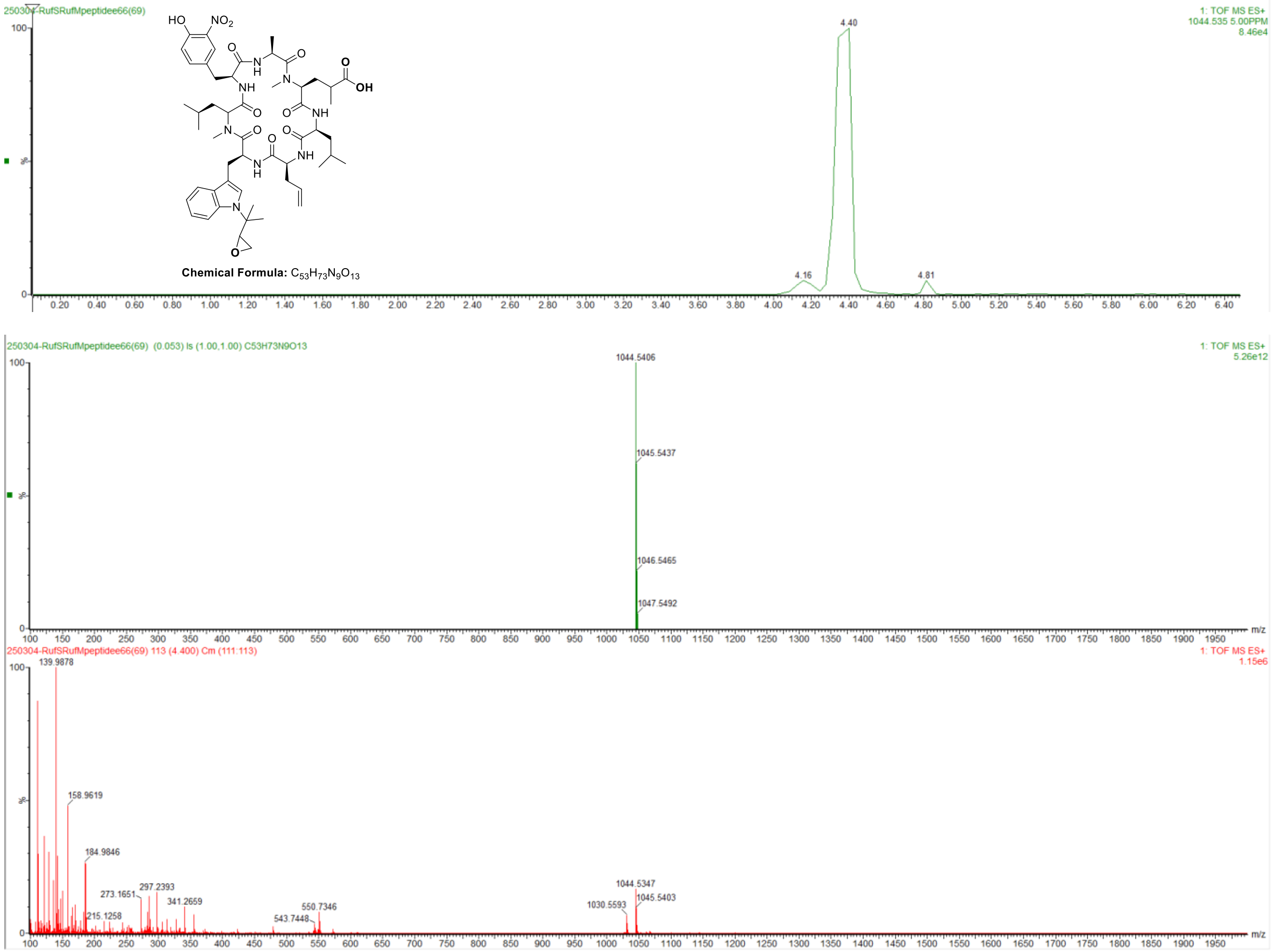

HRMS (ESI) calc. for $C_{53}H_{72}N_9O_{10}$ calc. for $[M+H]^+$: 1044.5406 m/z, found: 1044.5347 m/z.

***HRMS** of linear peptide **linear-3**. predicted mass spectrum (top) measured (bottom).*

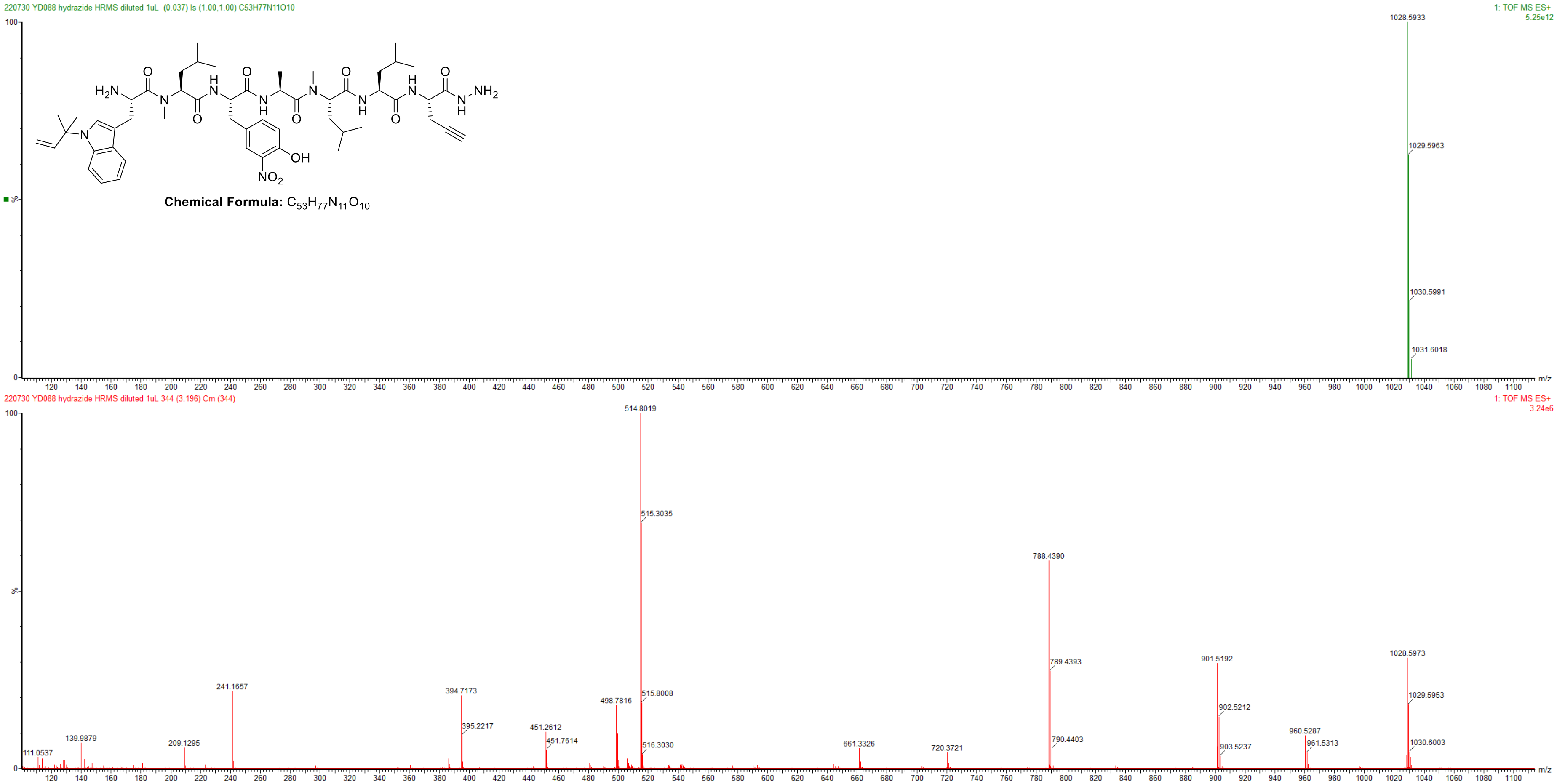

***HRMS** (ESI) calc. for $C_{53}H_{70}N_7O_{10}^+$ calc. for $[M+H]^+$: 1028.593 m/z, found: 1028.5973 m/z.*

*MS^E of Linear-**3***

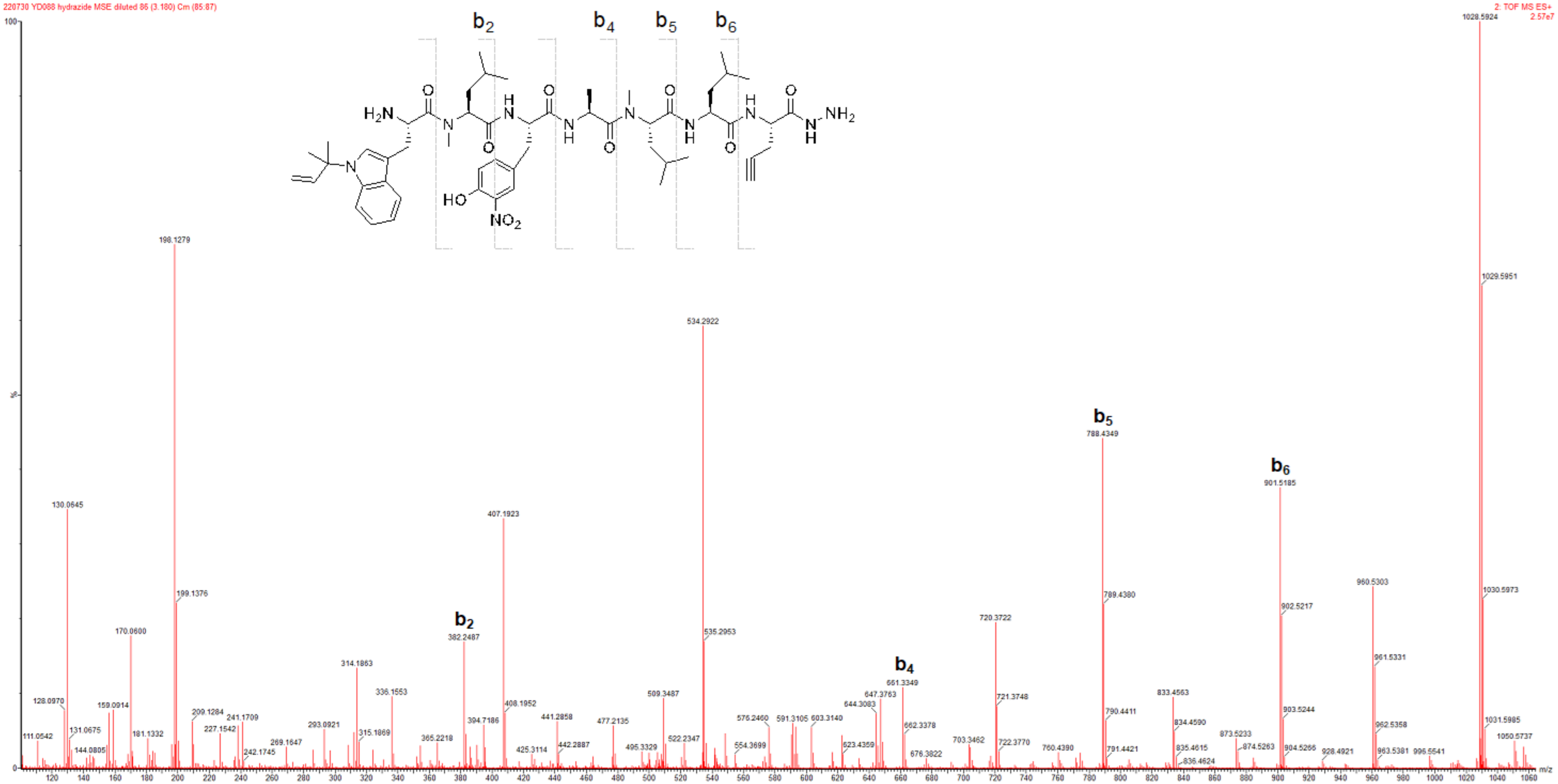

*HRMS extracted Ion Chromatogram of **3** (top), predicted mass spectrum (middle) measured mass spectrum (bottom).*

.

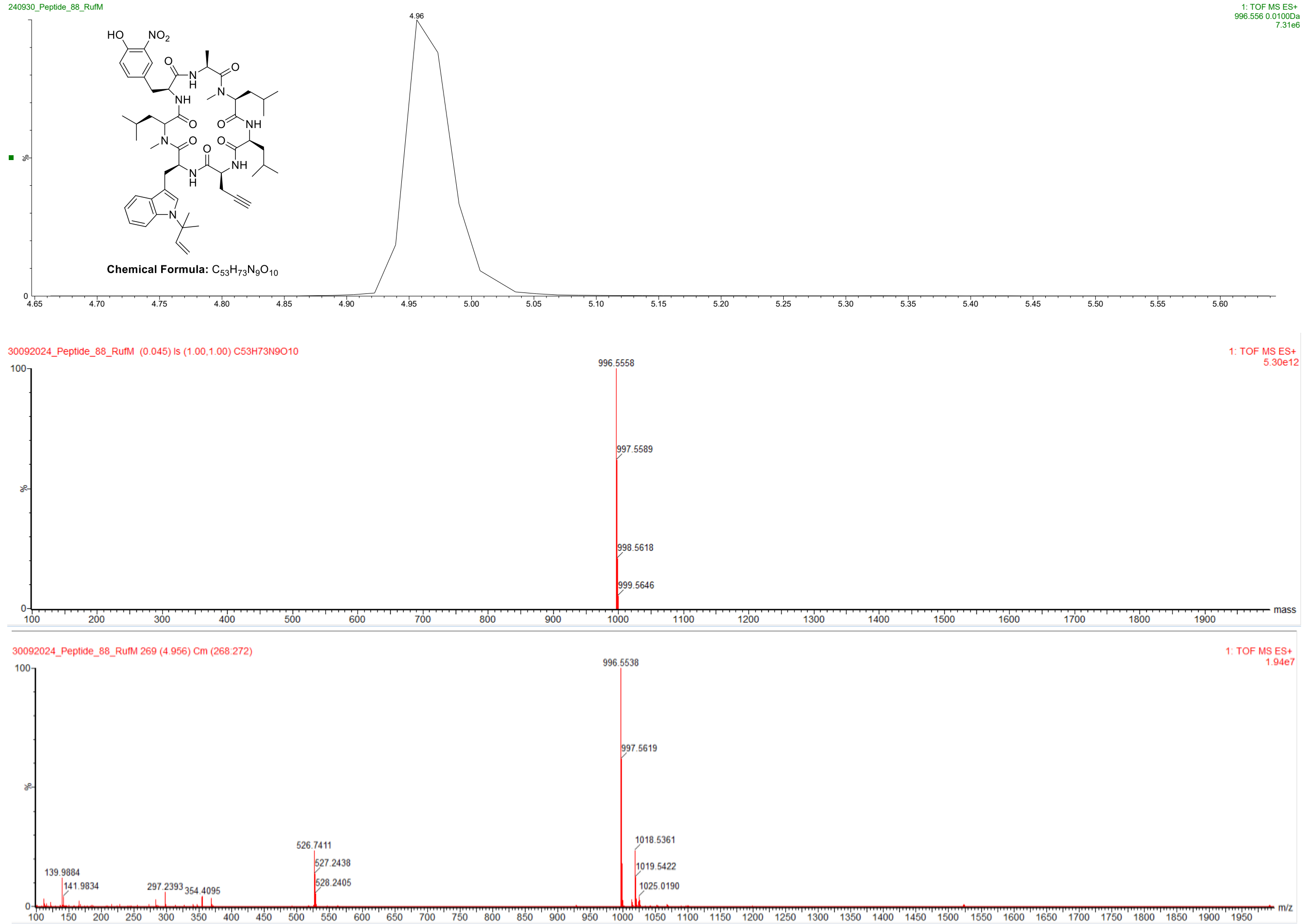

HRMS (ESI) calc. for $C_{53}H_{72}N_9O_{10}$ calc. for $[M+H]^+$: 996.5558 m/z, found: 996.5538 m/z.

*HRMS extracted Ion Chromatogram of **3b/b*** (top), predicted mass spectrum (middle) measured mass spectrum (bottom).*

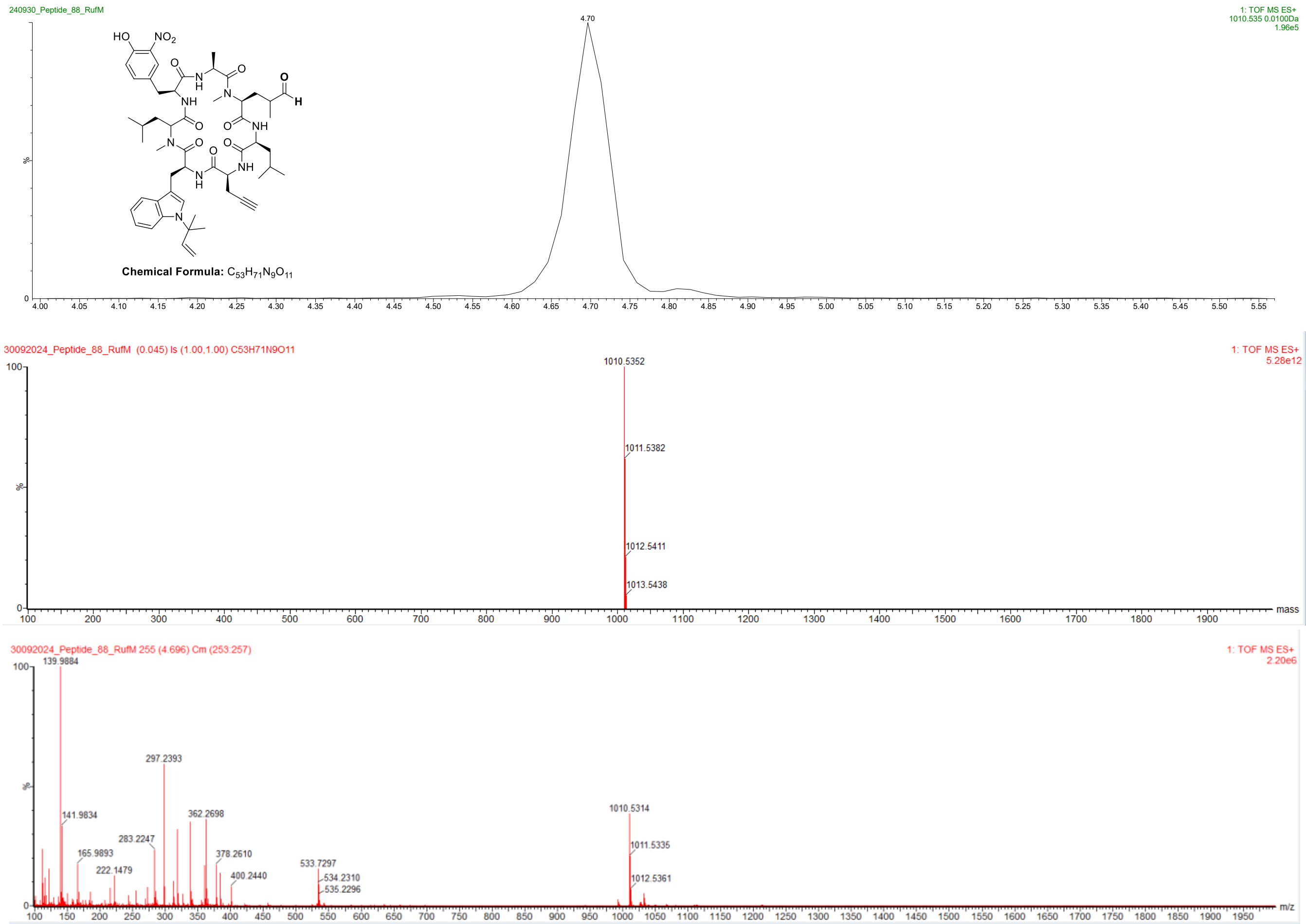

HRMS (ESI) calc. for $C_{53}H_{71}N_9O_{12}$ calc. for $[M+H]^+$: 1010.5352 m/z, found: 1010.5314 m/z.

*HRMS extracted Ion Chromatogram of Peptide **3d****(top), predicted mass spectrum (bottom) measured mass spectrum (middle).*

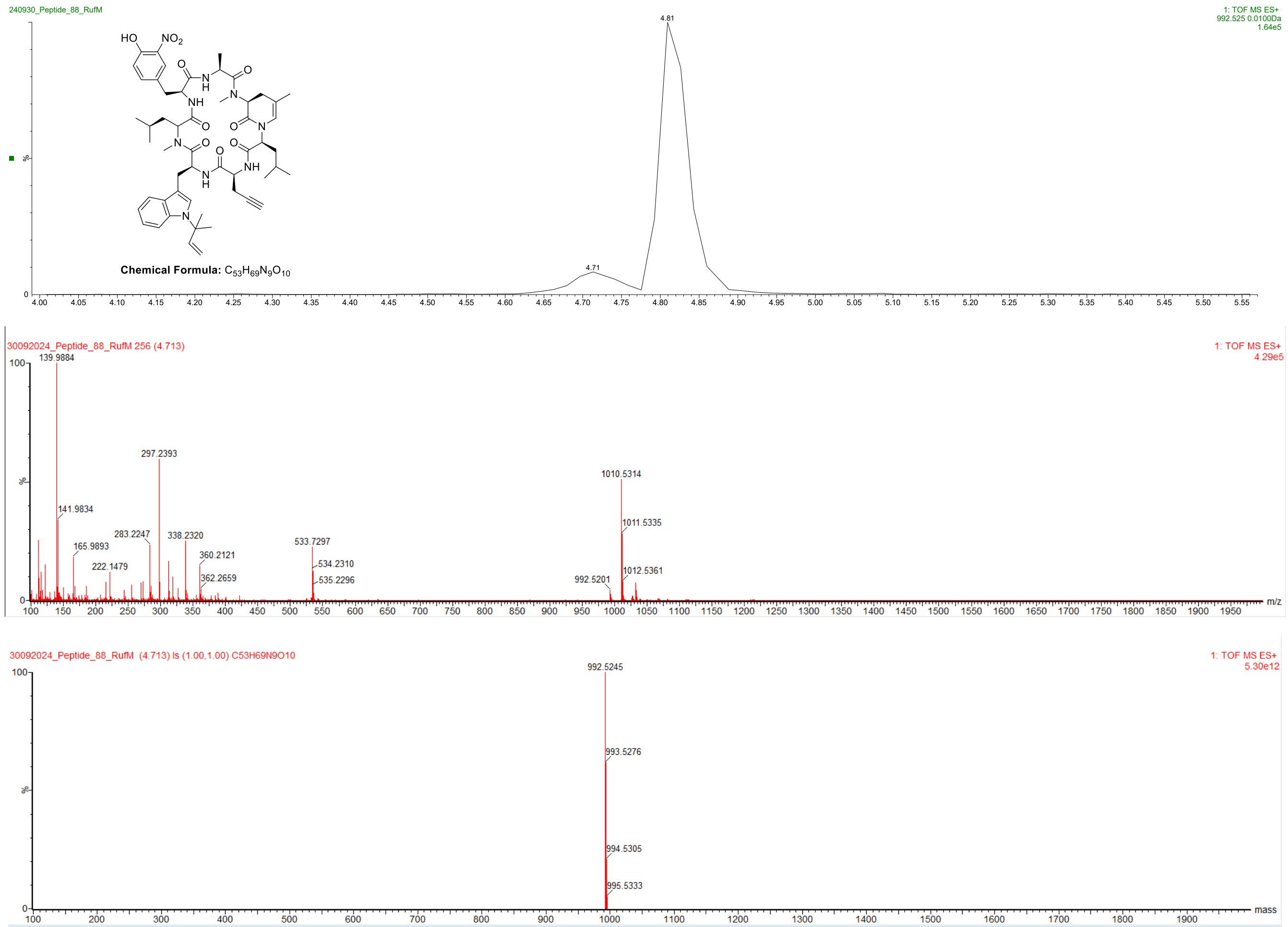

HRMS (ESI) calc. for $C_{53}H_{69}N_9O_{10}$ calc. for $[M+H]^+$: 992.5245 m/z, found: 992.5201 m/z.

*HRMS extracted Ion Chromatogram of Peptide **3c**(top), predicted mass spectrum (middle) measured mass spectrum (bottom).*

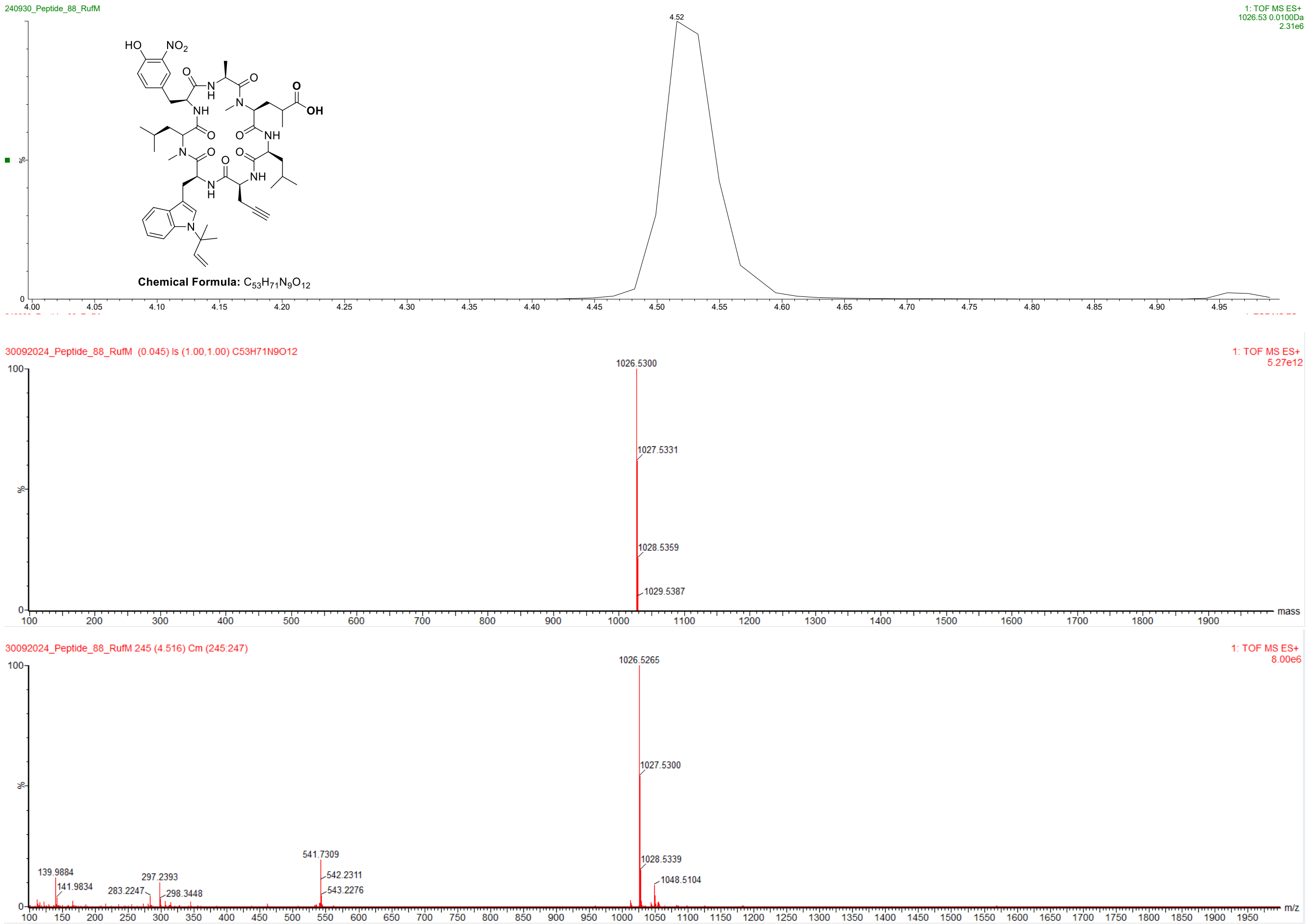

HRMS (ESI) calc. for $C_{53}H_{71}N_9O_{12}$ calc. for $[M+H]^+$: 1026.5300 m/z, found: 1026.5265 m/z.

*HRMS extracted Ion Chromatogram of Peptide **4**(top), predicted mass spectrum (middle) measured mass spectrum (bottom).*

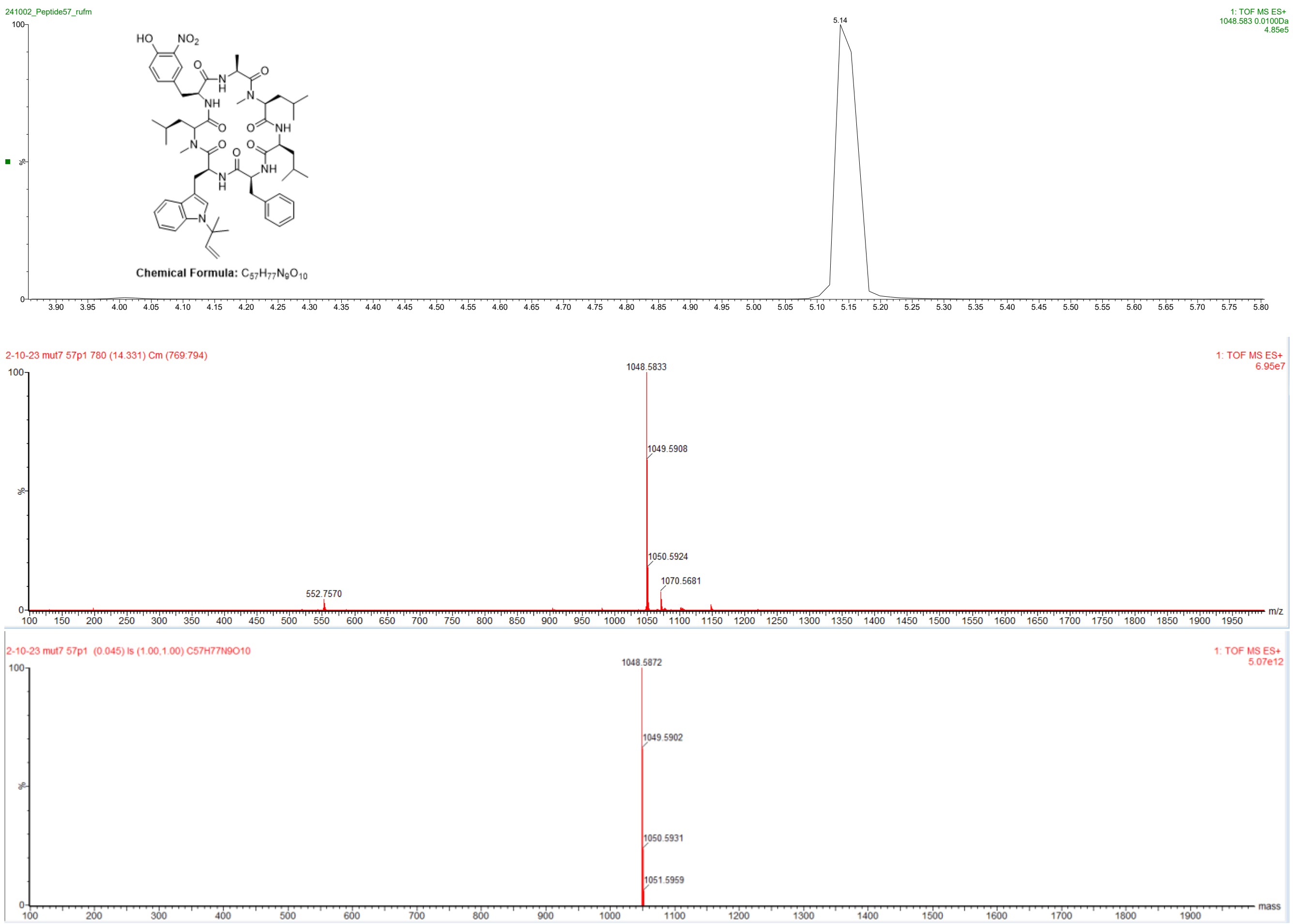

HRMS (ESI) calc. for $C_{57}H_{77}N_9O_{10}$ calc. for $[M+H]^+$: 1048.5833 m/z, found: 1048.5872 m/z

*HRMS extracted Ion Chromatogram of Peptide **4a** (top), predicted mass spectrum (middle) measured mass spectrum (bottom).*

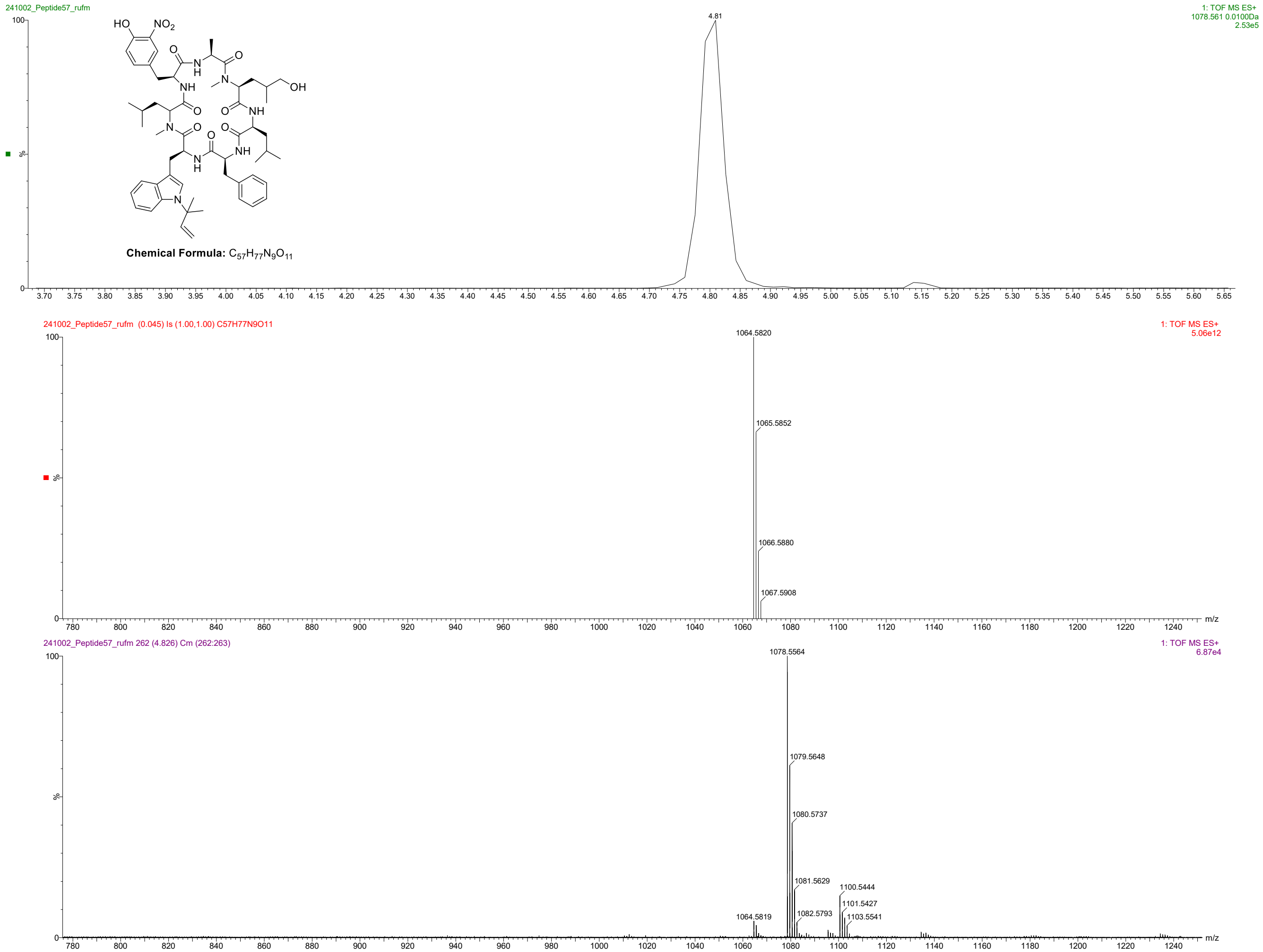

HRMS (ESI) calc. for $C_{54}H_{76}N_{9}O_{10}$ calc. for $[M+H]^{+}$: 1064.5820 m/z, found: 1064.5819 m/z.

*HRMS extracted Ion Chromatogram of Peptide **4b/b*** (top), predicted mass spectrum (middle) measured mass spectrum (bottom).*

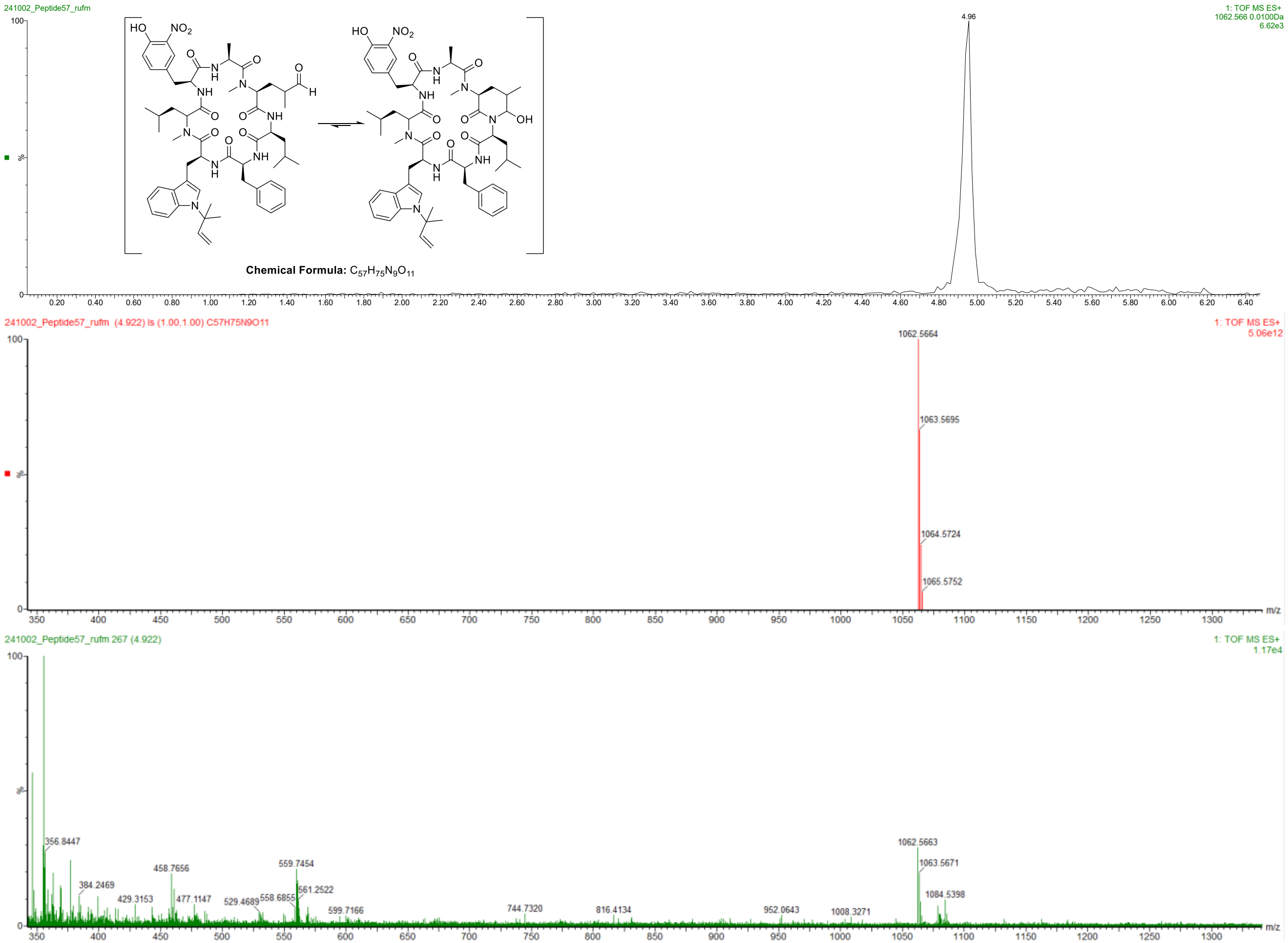

HRMS (ESI) calc. for $C_{54}H_{76}N_9O_{10}$ calc. for $[M+H]^+$: 1062.5664 m/z, found: 1062.5563 m/z.

*HRMS extracted Ion Chromatogram of Peptide **4d** (top), predicted mass spectrum (middle) measured mass spectrum (bottom).*

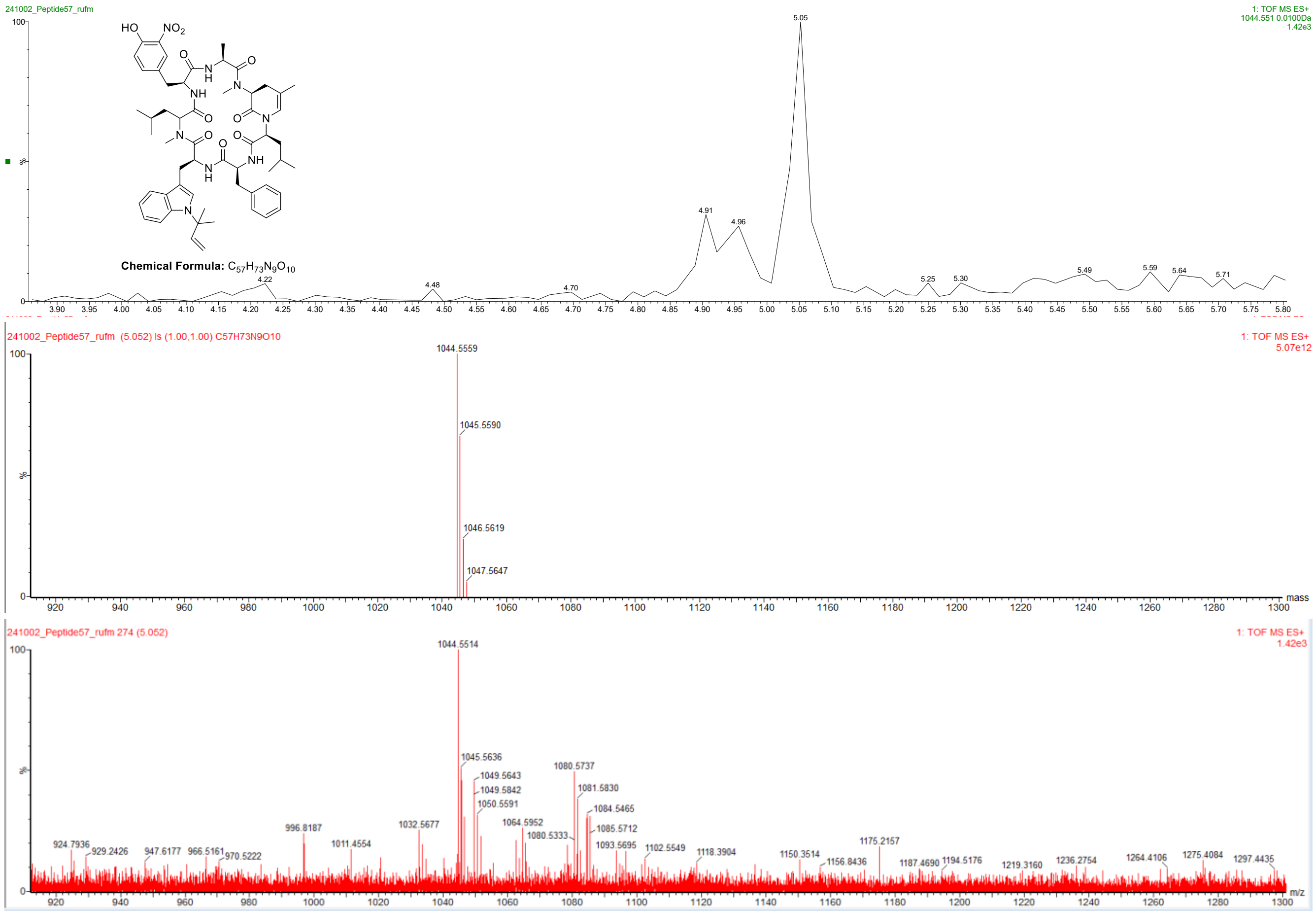

HRMS (ESI) calc. for $C_{57}H_{73}N_{9}O_{10}$ calc. for $[M+H]^{+}$: 1044.5559 m/z, found: 1044.5514 m/z.

*HRMS extracted Ion Chromatogram of Peptide **4c**(top), predicted mass spectrum (middle) measured mass spectrum (bottom).*

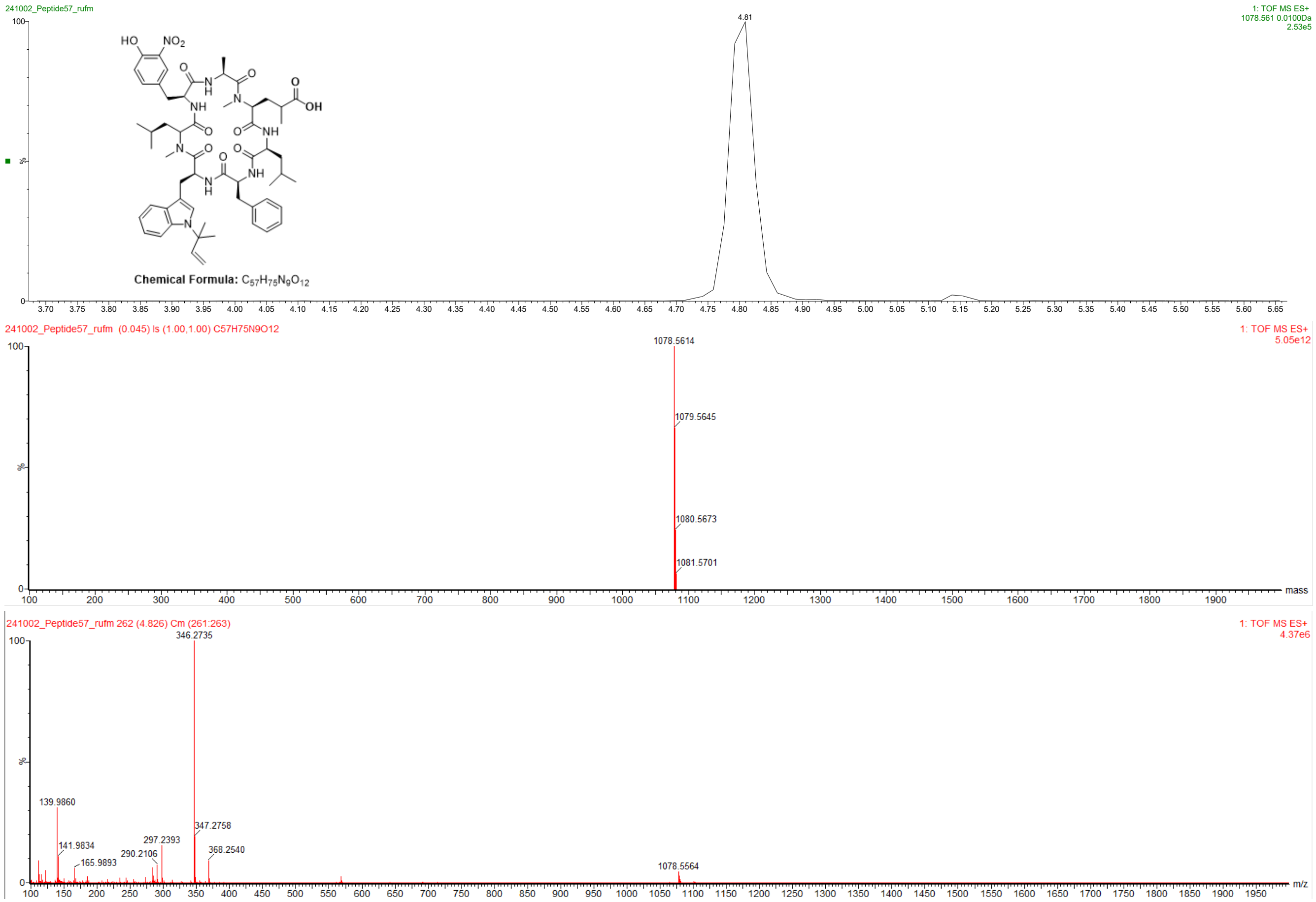

HRMS (ESI) calc. for $C_{54}H_{75}N_9O_{10}$ calc. for $[M+H]^+$: 1078.5614 m/z, found: 1078.5564 m/z.

*HRMS extracted Ion Chromatogram of Peptide **5** (predicted mass spectrum (top) measured (bottom))*

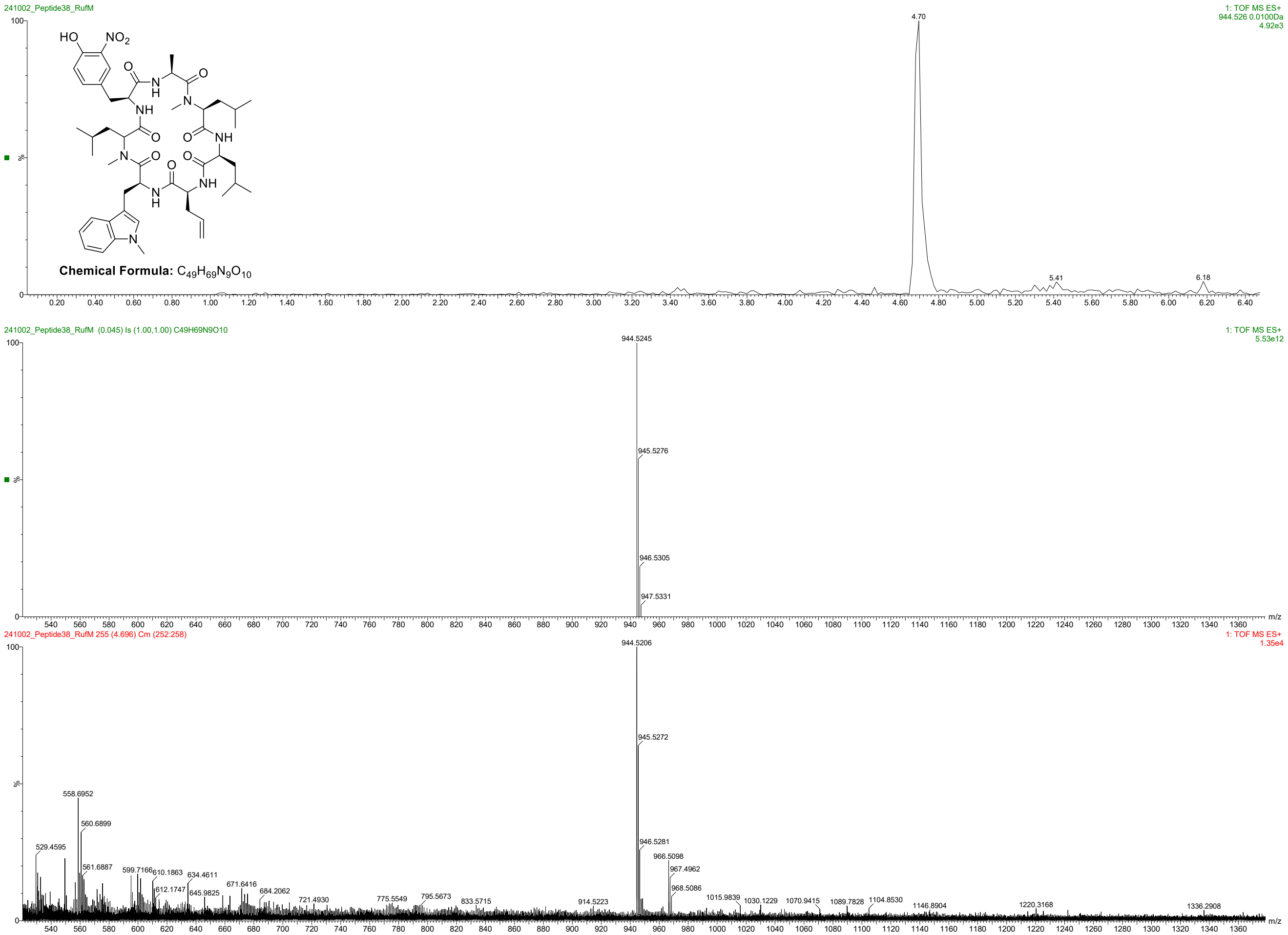

HRMS (ESI) calc. for $C_{54}H_{76}N_9O_{10}$ calc. for $[M+H]^+$: 944.5245 m/z, found: 944.5206 m/z.

*HRMS extracted Ion Chromatogram of Peptide **5a**(top), predicted mass spectrum (middle) measured mass spectrum (bottom).*

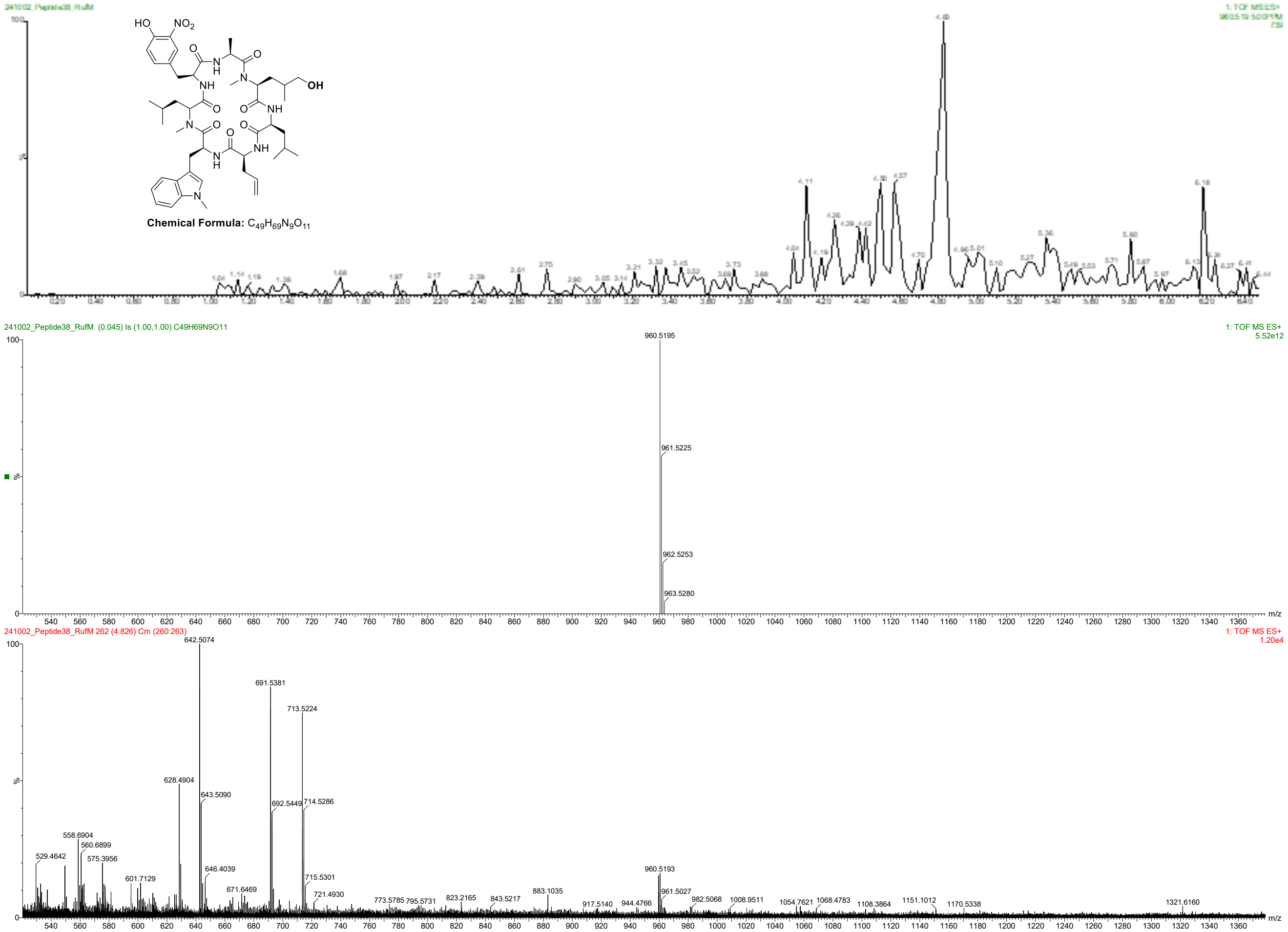

HRMS (ESI) calc. for $C_{54}H_{76}N_9O_{10}$ calc. for $[M+H]^+$: 960.5195 m/z, found: 960.5193 m/z.

HRMS Extracted Ion Chromatogram of Peptide ***5b/b**** *(top), predicted mass spectrum (middle) measured mass spectrum (bottom).*

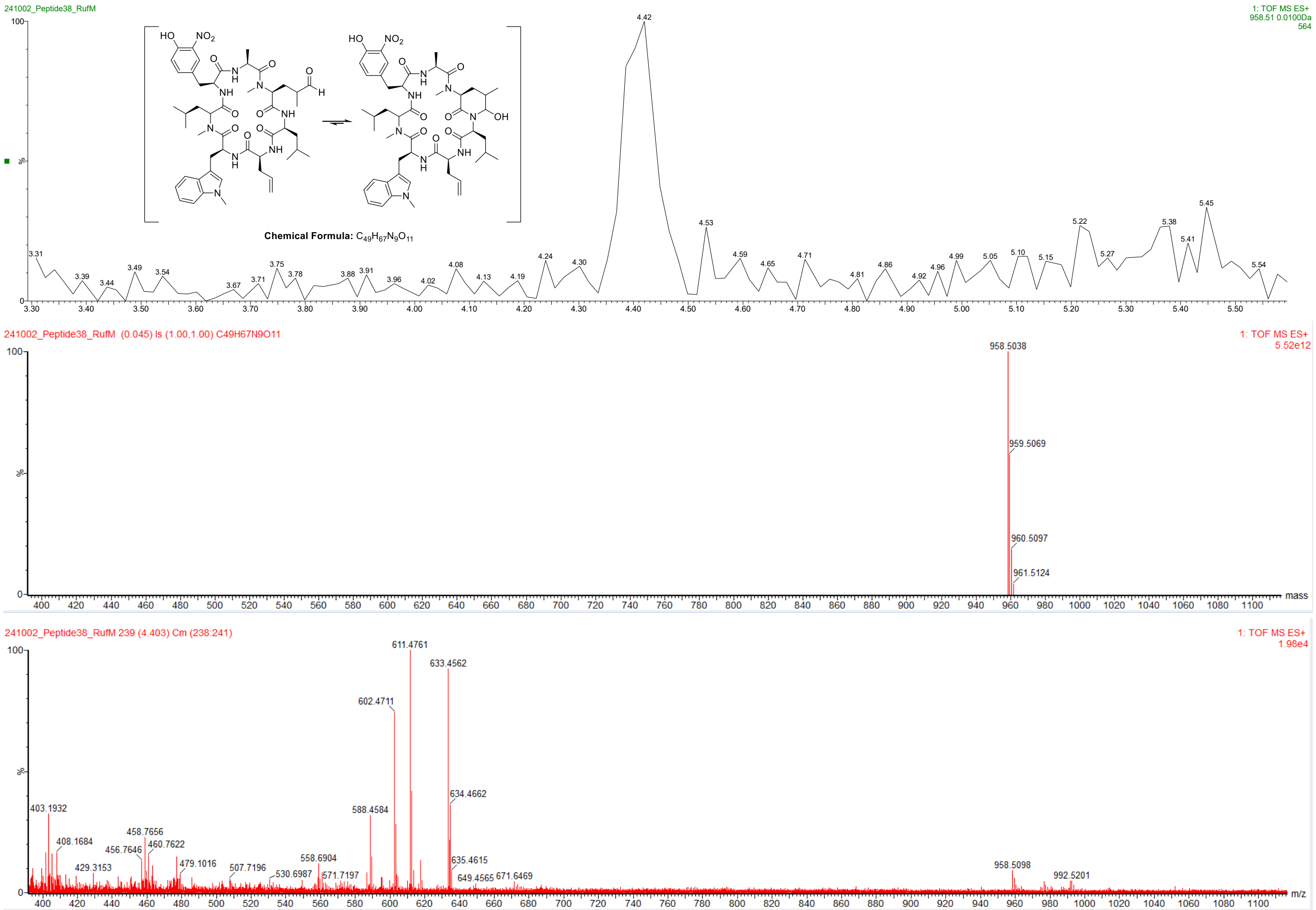

HRMS (ESI) calc. for $C_{49}H_{67}N_9O_{11}$ calc. for $[M+H]^+$: 958.5038 m/z, found: 958.5098 m/z.

*HRMS Extracted Ion Chromatogram of Peptide **5c** (top), predicted mass spectrum (middle) measured mass spectrum (bottom).*

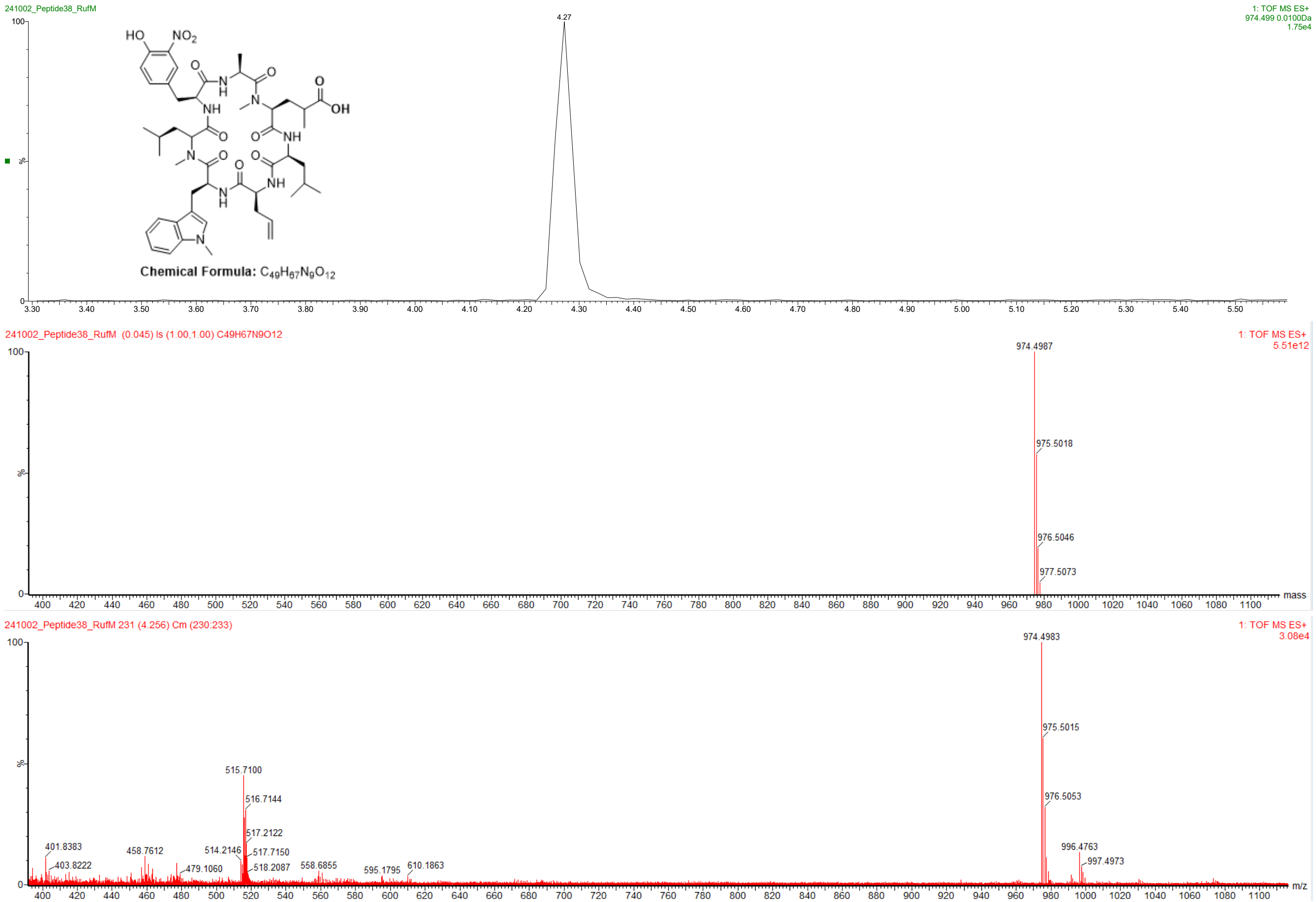

HRMS (ESI) calc. for $C_{54}H_{76}N_9O_{10}$ calc. for $[M+H]^+$: 974.4987 m/z, found: 974.4983 m/z.

*HRMS of Linear Peptide **6** (predicted mass spectrum (top) measured (bottom)).*

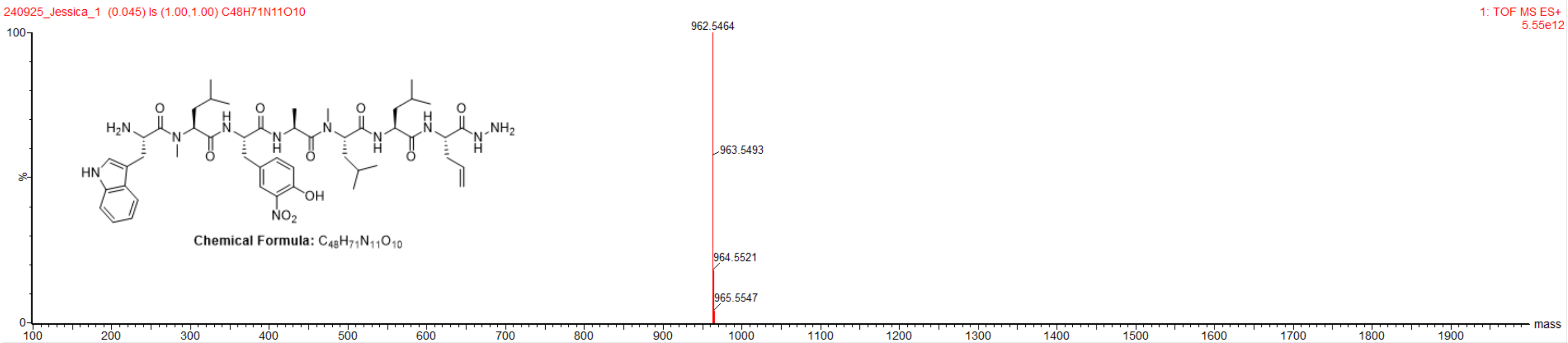

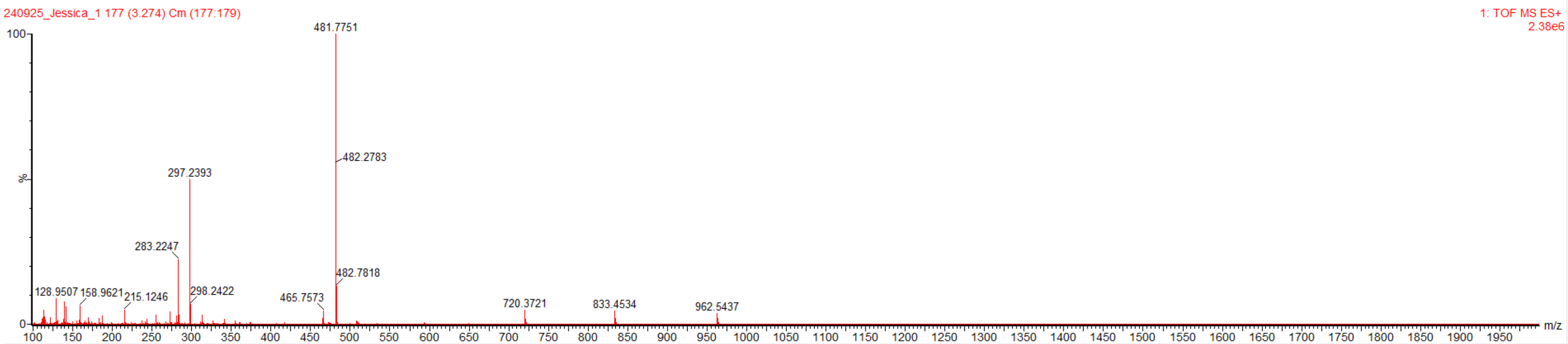

HRMS (ESI) calc. for $C_{48}H_{71}N_{11}O_{10}$ calc. for $[M+H]^+$: 962.5464 m/z, found: 962.5437 m/z.

*MS^e of Linear Peptide **6***

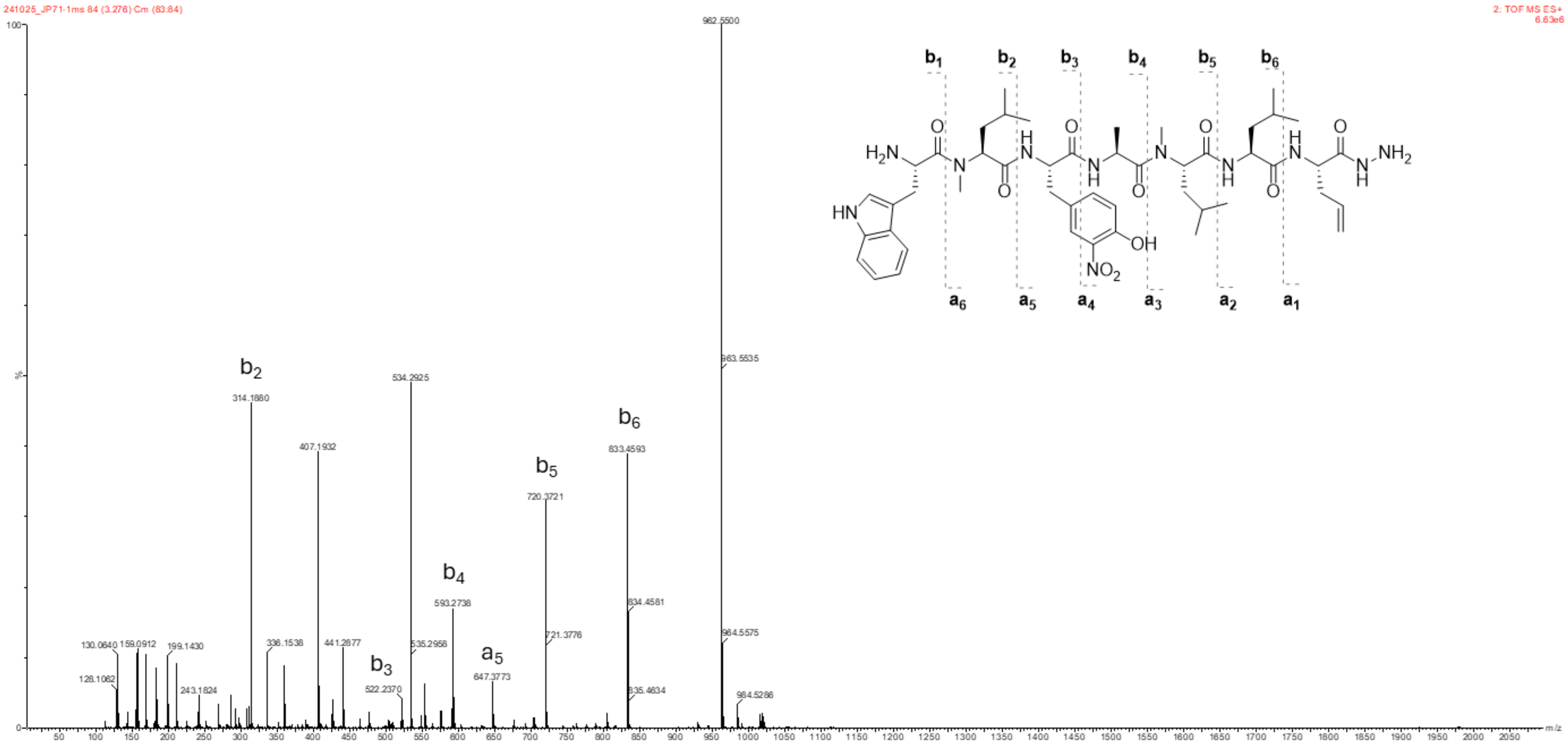

*HRMS extracted Ion Chromatogram of Peptide **6** (top), predicted mass spectrum (middle) measured mass spectrum (bottom).*

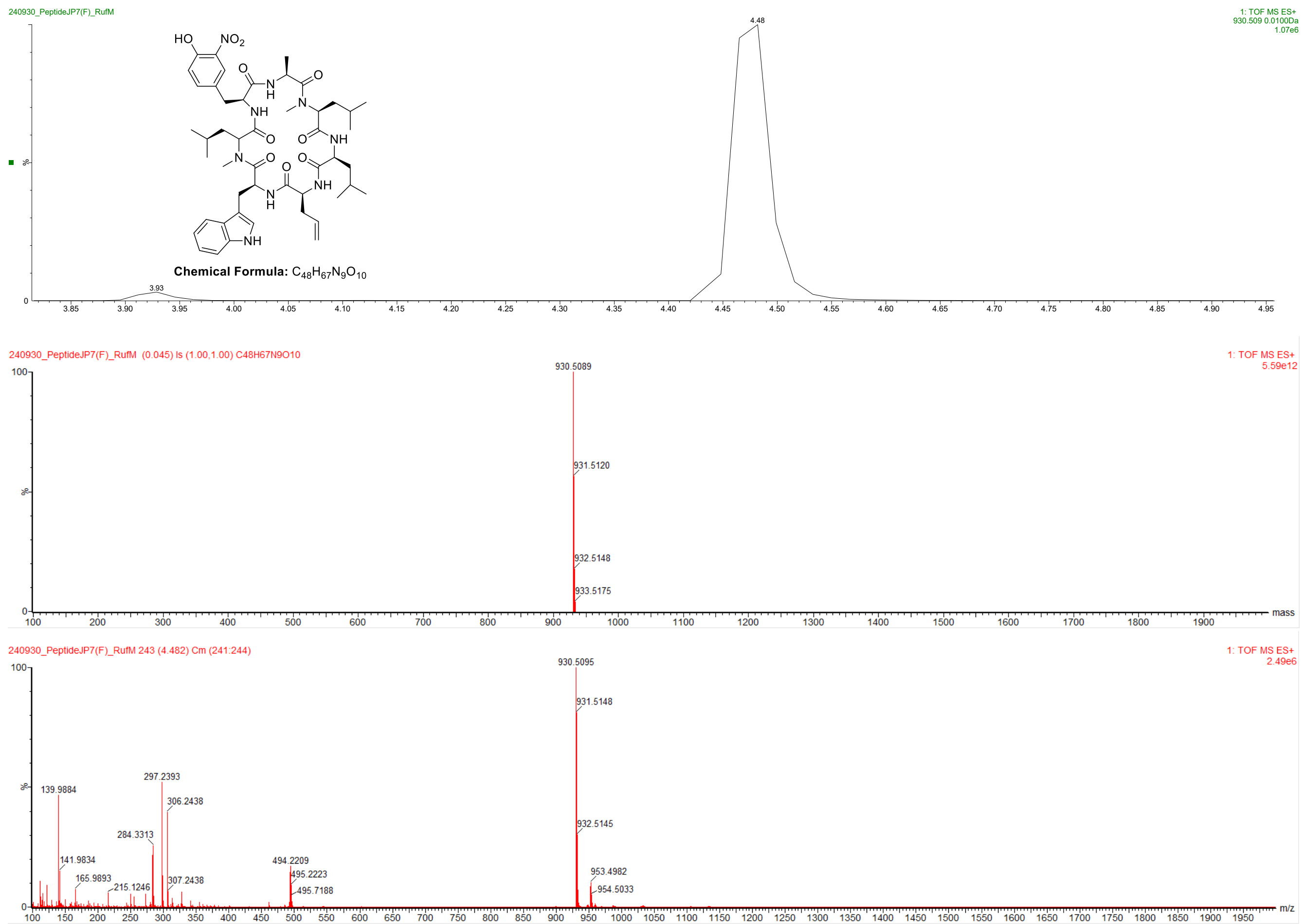

HRMS (ESI) calc. for $C_{48}H_{67}N_9O_{10}$ calc. for $[M+H]^+$: 930.5089 m/z, found: 930.5095 m/z.

HRMS Extracted Ion Chromatogram of Peptide ***6b/b**** *(top), predicted mass spectrum (middle) measured mass spectrum (bottom).*

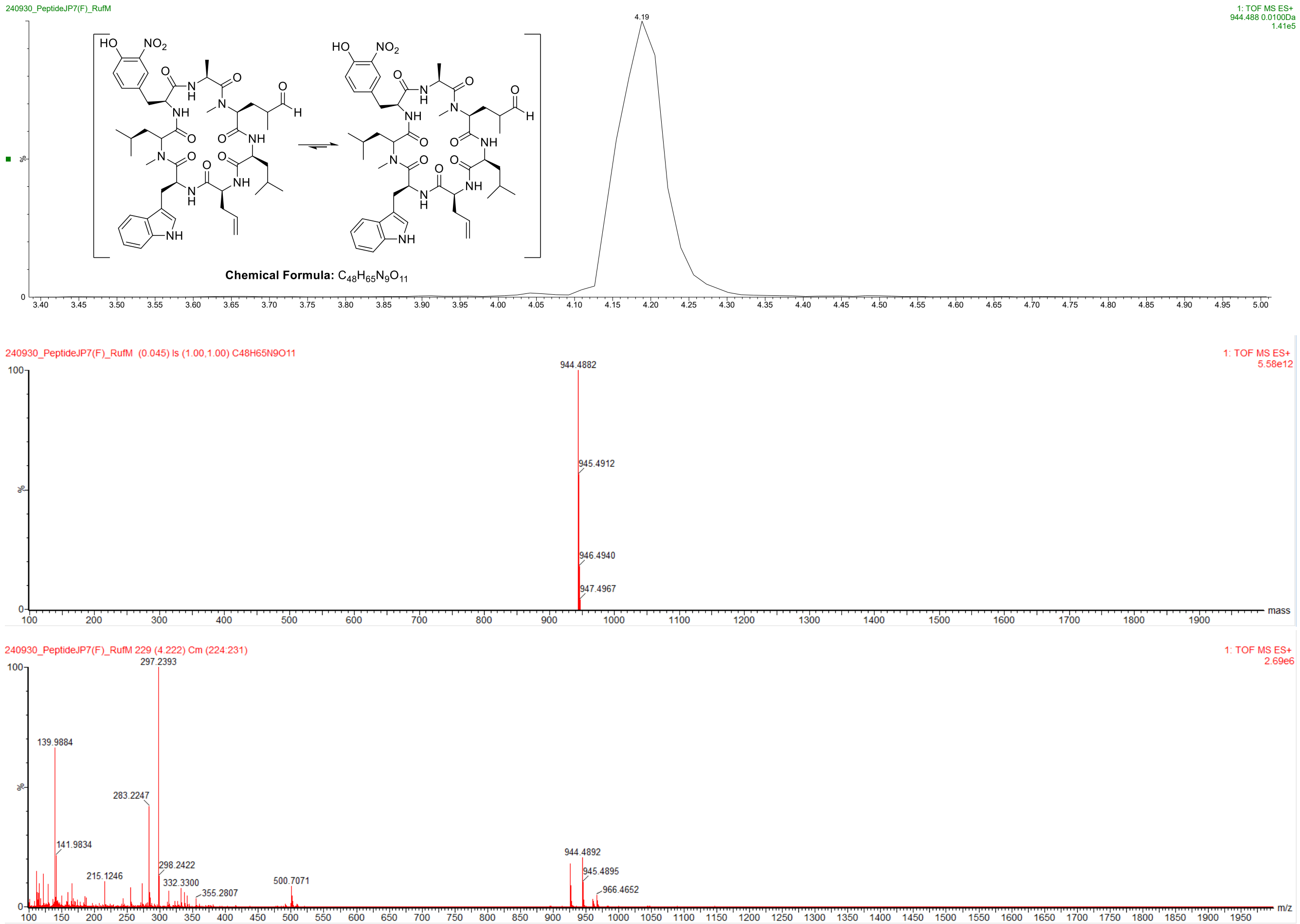

HRMS (ESI) calc. for $C_{48}H_{65}N_9O_{11}$ calc. for $[M+H]^+$: 944.4882 m/z, found: 944.4892 m/z.

HRMS Extracted Ion Chromatogram of Peptide **6d** *(top), predicted mass spectrum (middle) measured mass spectrum (bottom).*

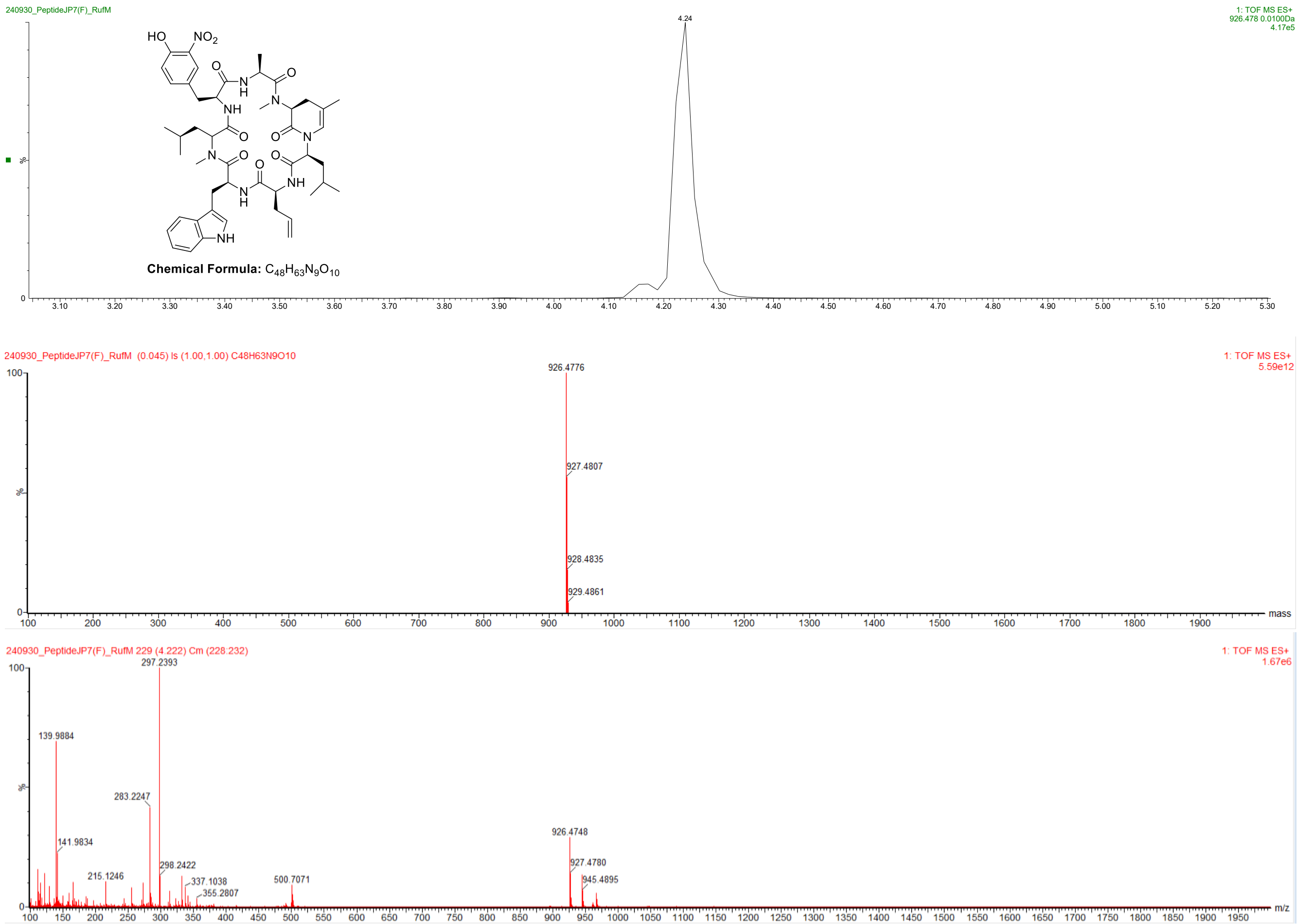

HRMS (ESI) calc. for $C_{48}H_{63}N_9O_{10}$ calc. for $[M+H]^+$: 926.4776 m/z, found: 926.4748 m/z.

*HRMS Extracted Ion Chromatogram of Peptide **6c** (top), predicted mass spectrum (middle) measured mass spectrum (bottom).*

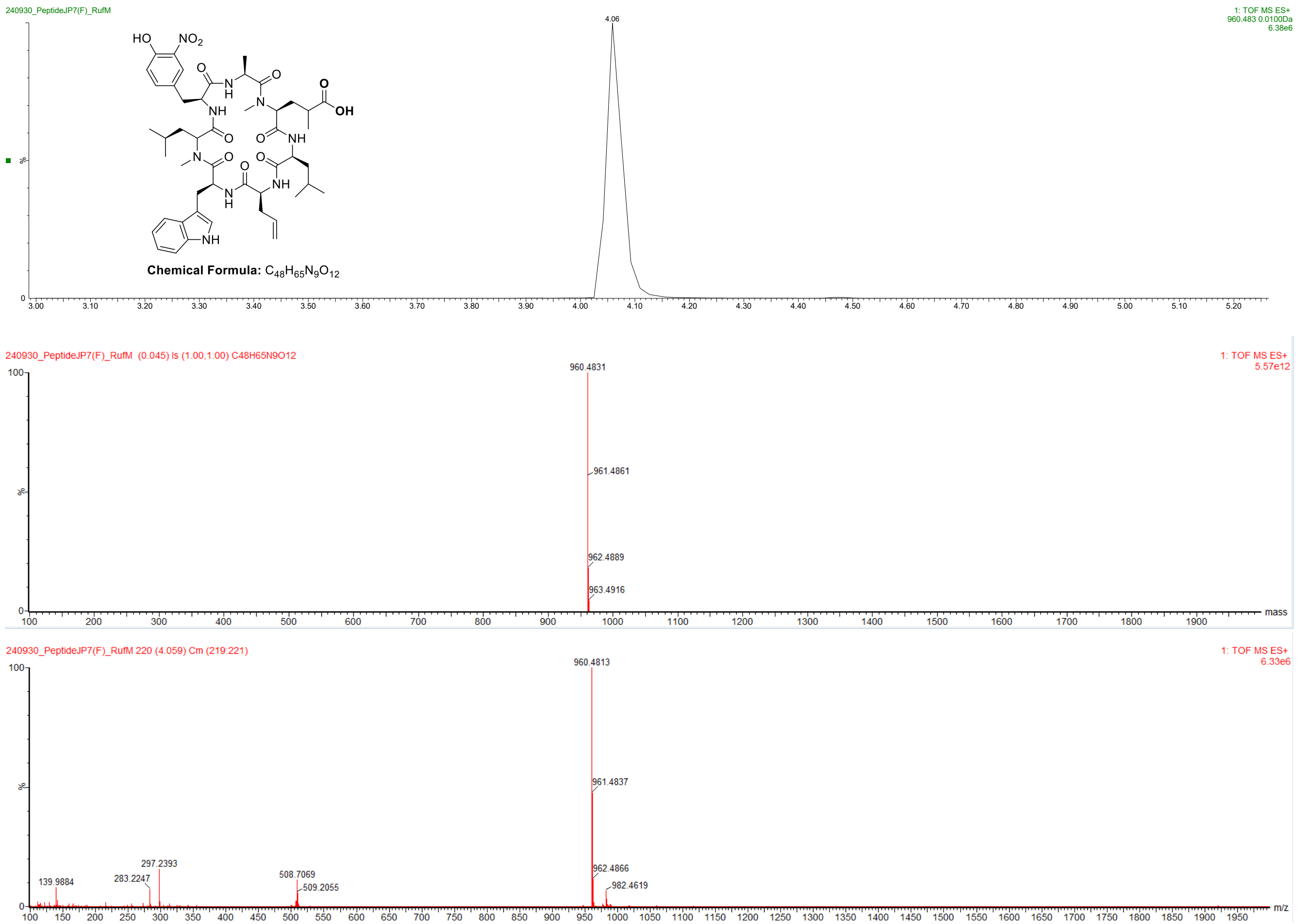

HRMS (ESI) calc. for $C_{48}H_{65}N_9O_{12}$ calc. for $[M+H]^+$: 960.4831 m/z, found: 960.4813 m/z.

*HRMS Linear Peptide **7** (top), predicted mass spectrum (top) measured mass spectrum (bottom).*

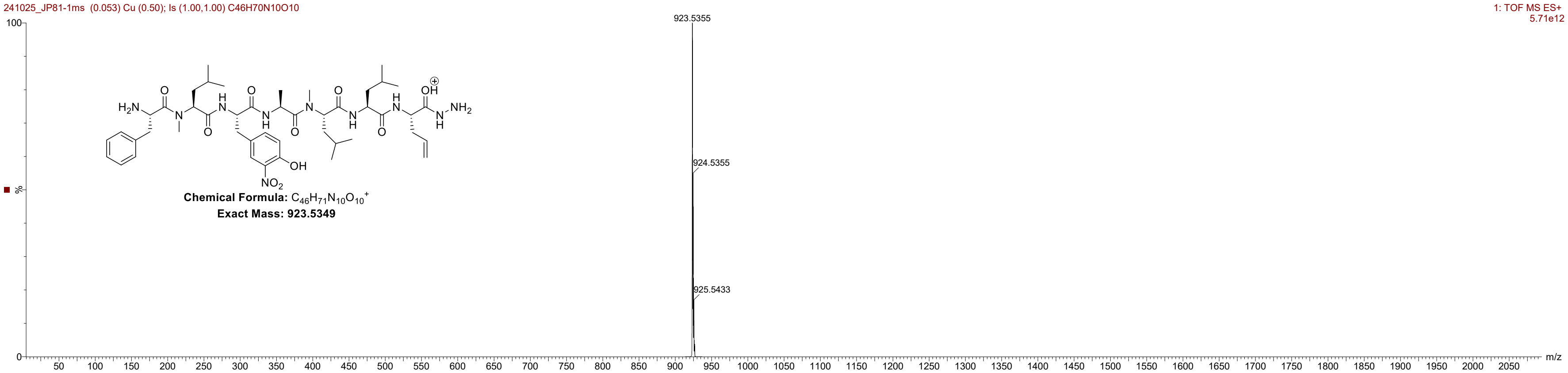

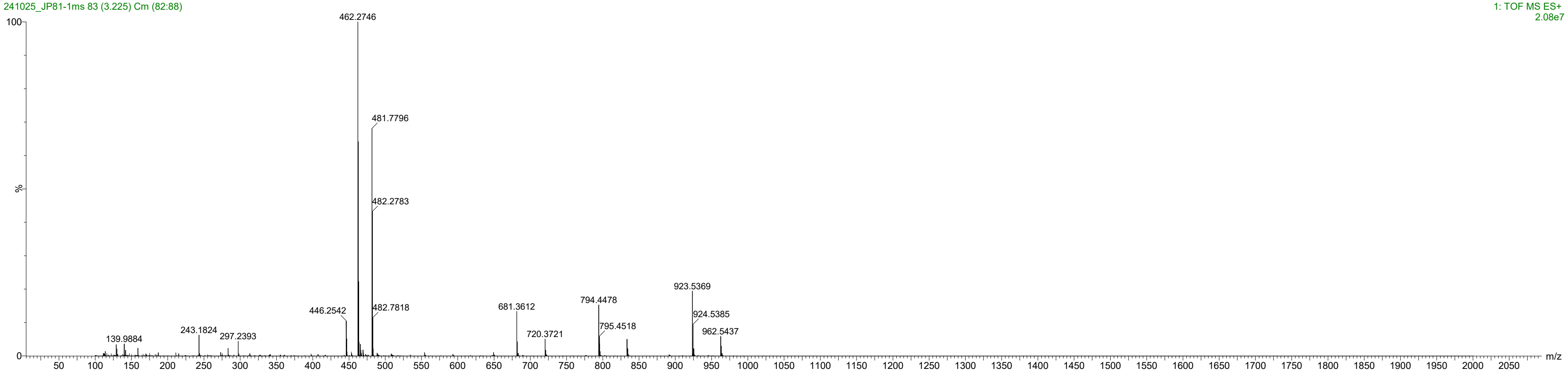

HRMS (ESI) calc. for $C_{46}H_{70}N_{10}O_{10}$ calc. for $[M+H]^+$: 923.5355 m/z, found: 923.5369 m/z.

*MS/MS of Linear Peptide **7**.*

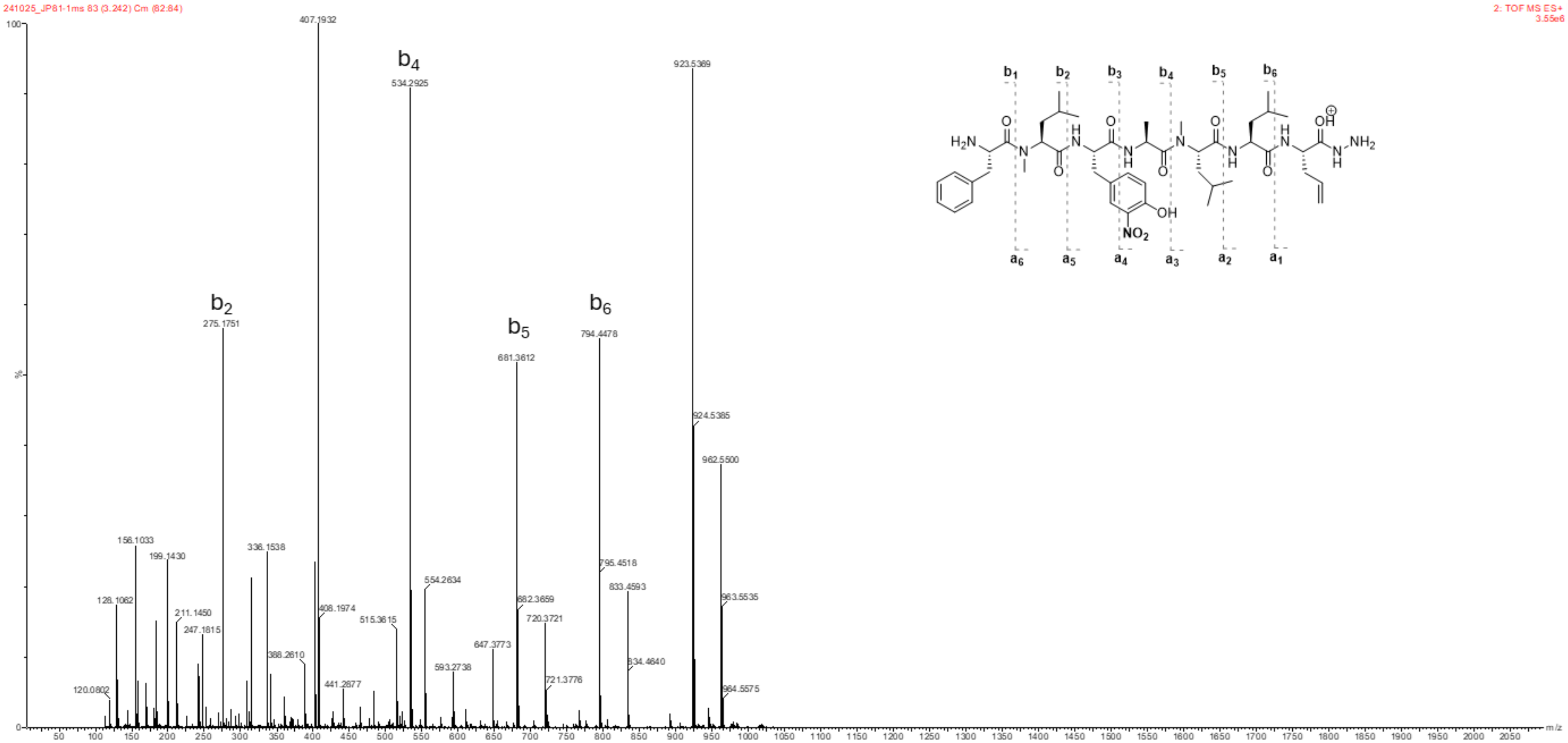

*HRMS extracted Ion Chromatogram of Peptide **7** (top), predicted mass spectrum (middle) measured mass spectrum (bottom).*

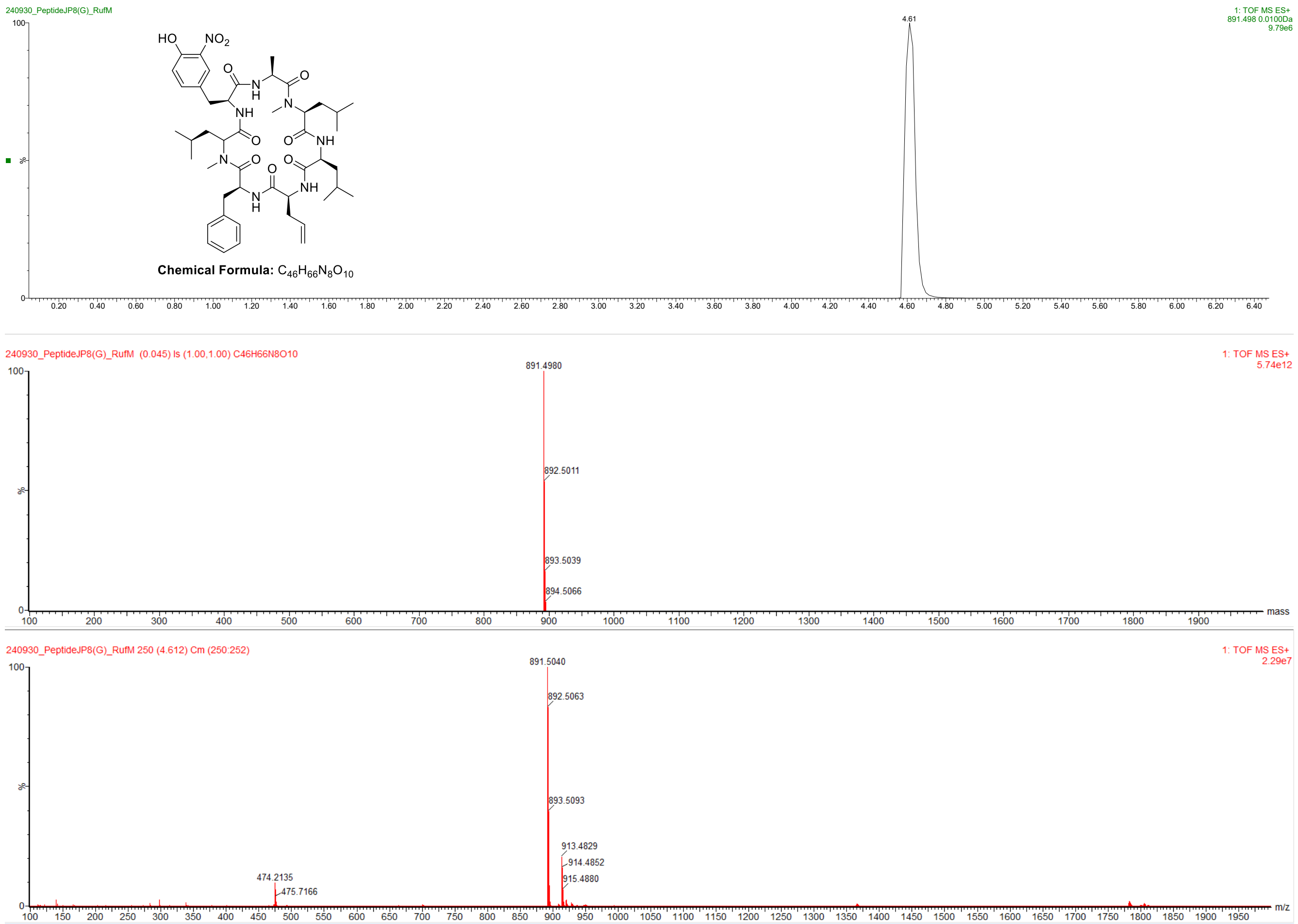

HRMS (ESI) calc. for $C_{48}H_{65}N_9O_{12}$ calc. for $[M+H]^+$: 891.4980 m/z, found: 891.5040 m/z.

*HRMS Extracted Ion Chromatogram of Peptide **7a** (top), predicted mass spectrum (middle) measured mass spectrum (bottom).*

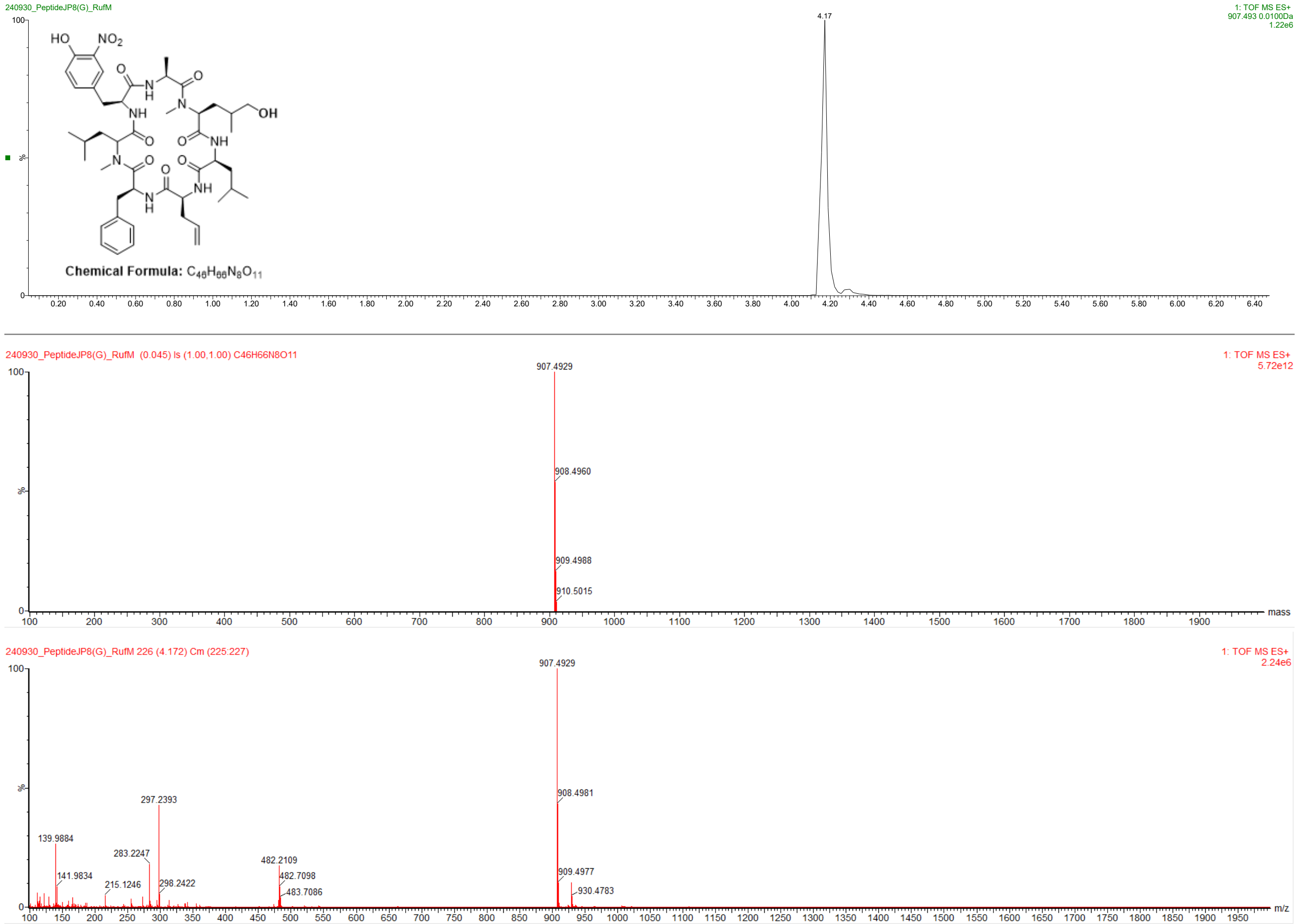

HRMS (ESI) calc. for $C_{46}H_{66}N_8O_{11}$ calc. for $[M+H]^+$: 907.4929 m/z, found: 907.4929 m/z.

*HRMS extracted Ion Chromatogram of Peptide **7b/b****(top), predicted mass spectrum (middle) measured mass spectrum (bottom).*

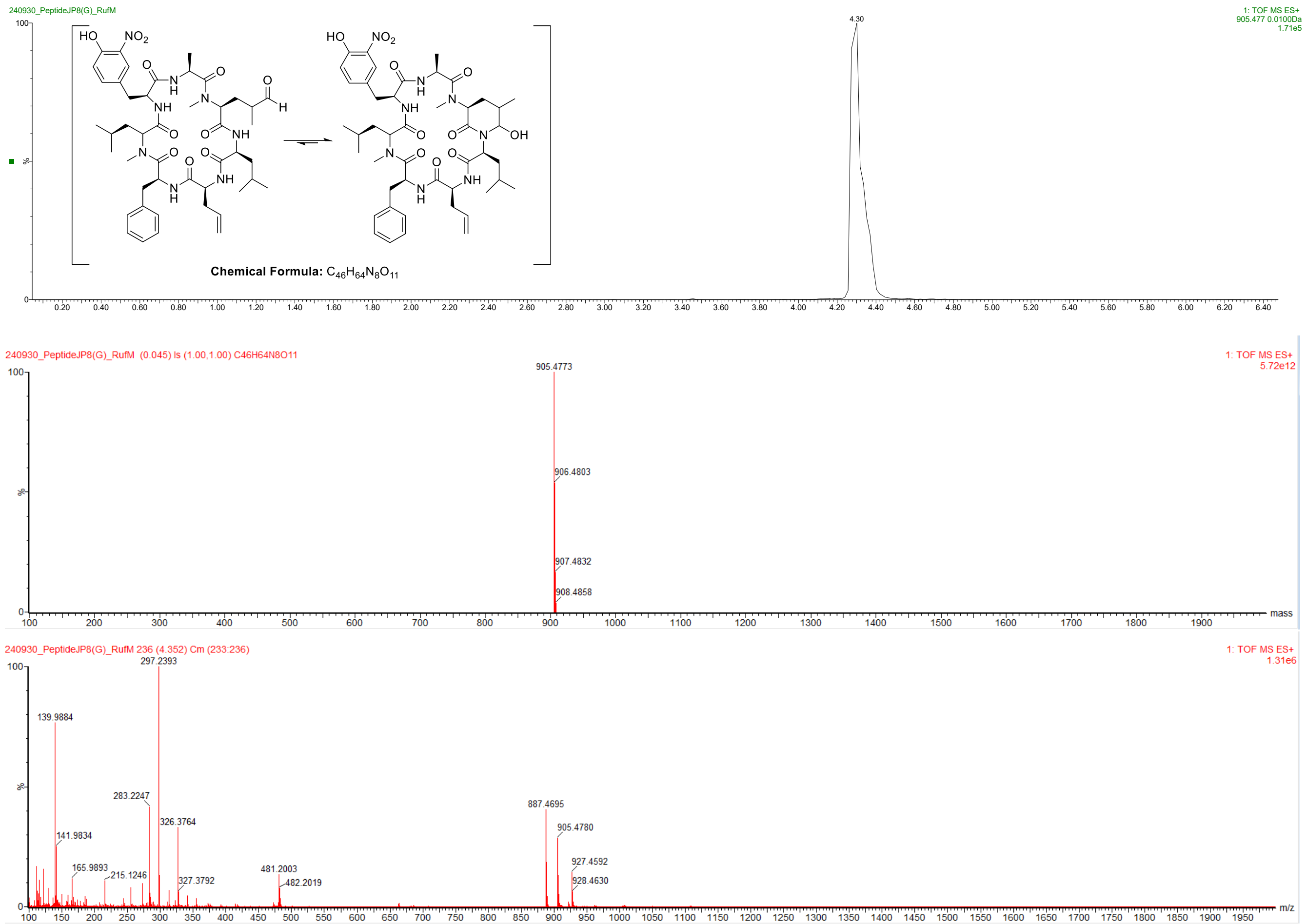

HRMS (ESI) calc. for $C_{46}H_{64}N_8O_{11}$ calc. for $[M+H]^+$: 905.4773 m/z, found: 905.4780 m/z.

*HRMS Extracted Ion Chromatogram of Peptide **7d** (top), predicted mass spectrum (middle) measured mass spectrum (bottom).*

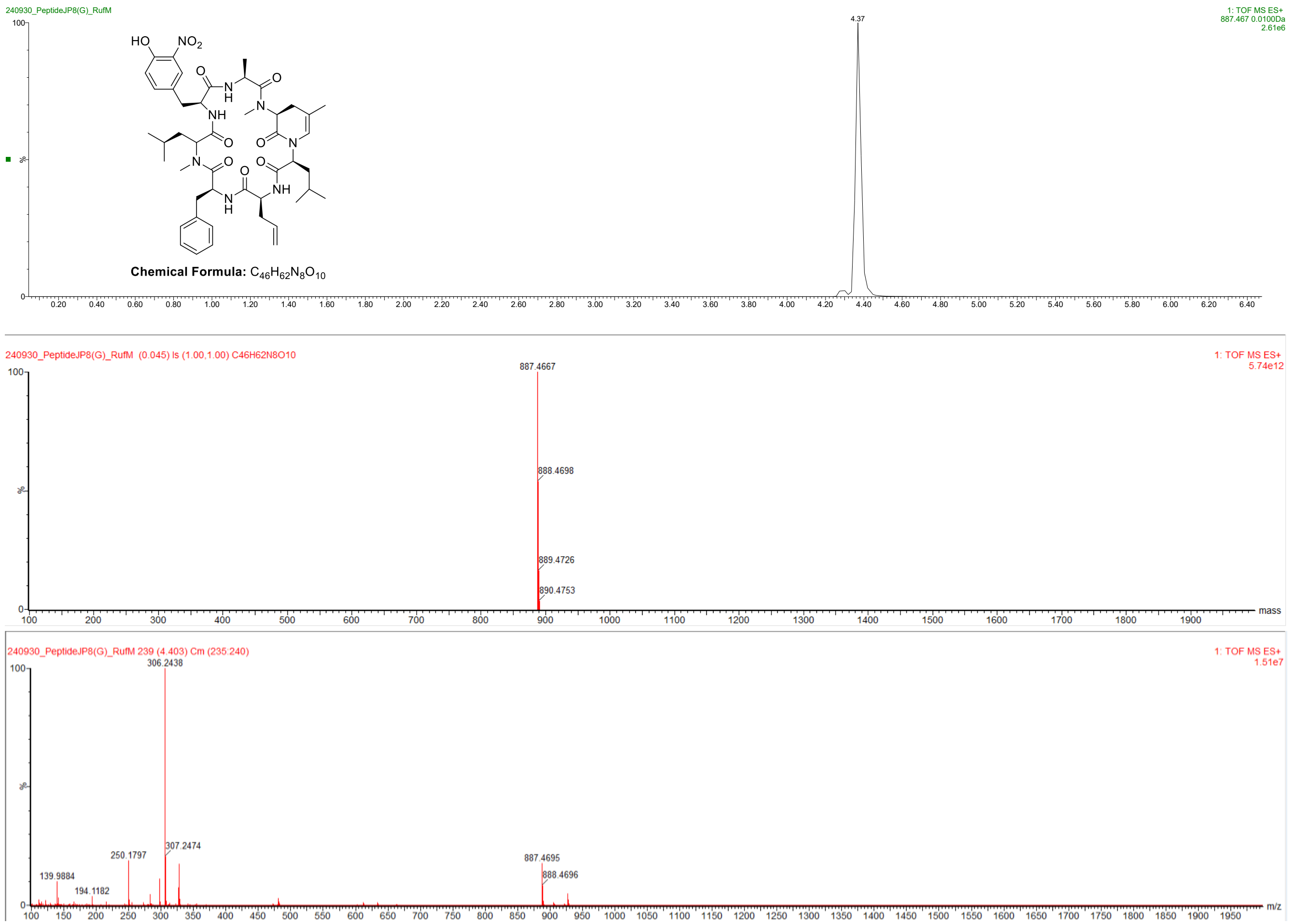

HRMS (ESI) calc. for $C_{46}H_{62}N_8O_{10}$ calc. for $[M+H]^+$: 887.4667 m/z, found: 887.4695 m/z.

*HRMS Extracted Ion Chromatogram of Peptide **8** (top), predicted mass spectrum (middle) measured mass spectrum (bottom).*

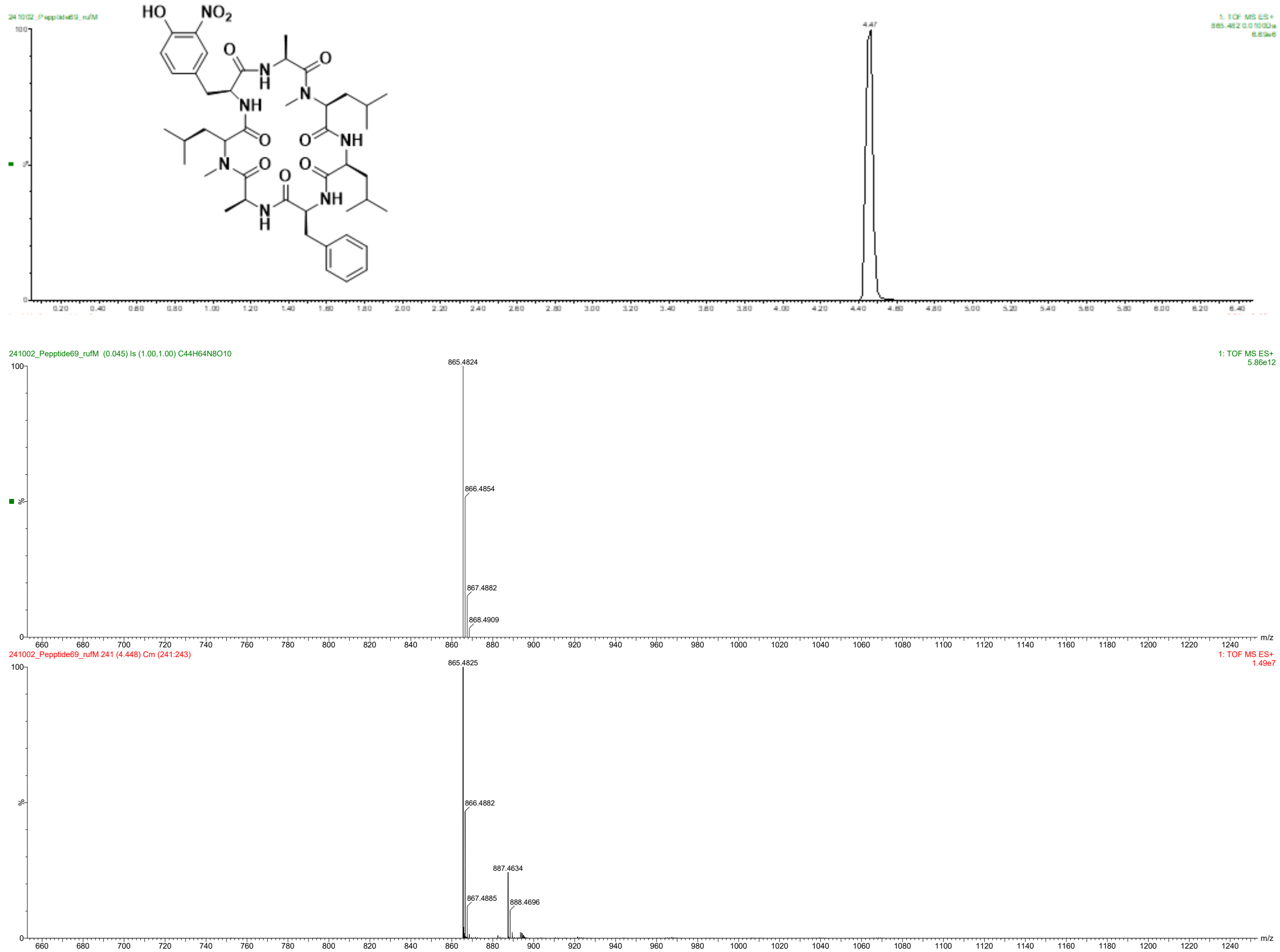

HRMS (ESI) calc. for $C_{54}H_{76}N_9O_{10}$ calc. for $[M+H]^+$: 865.4824 m/z, found: 865.4825 m/z.

*HRMS of Linear Peptide **9** (predicted mass spectrum (top) measured (bottom)).*

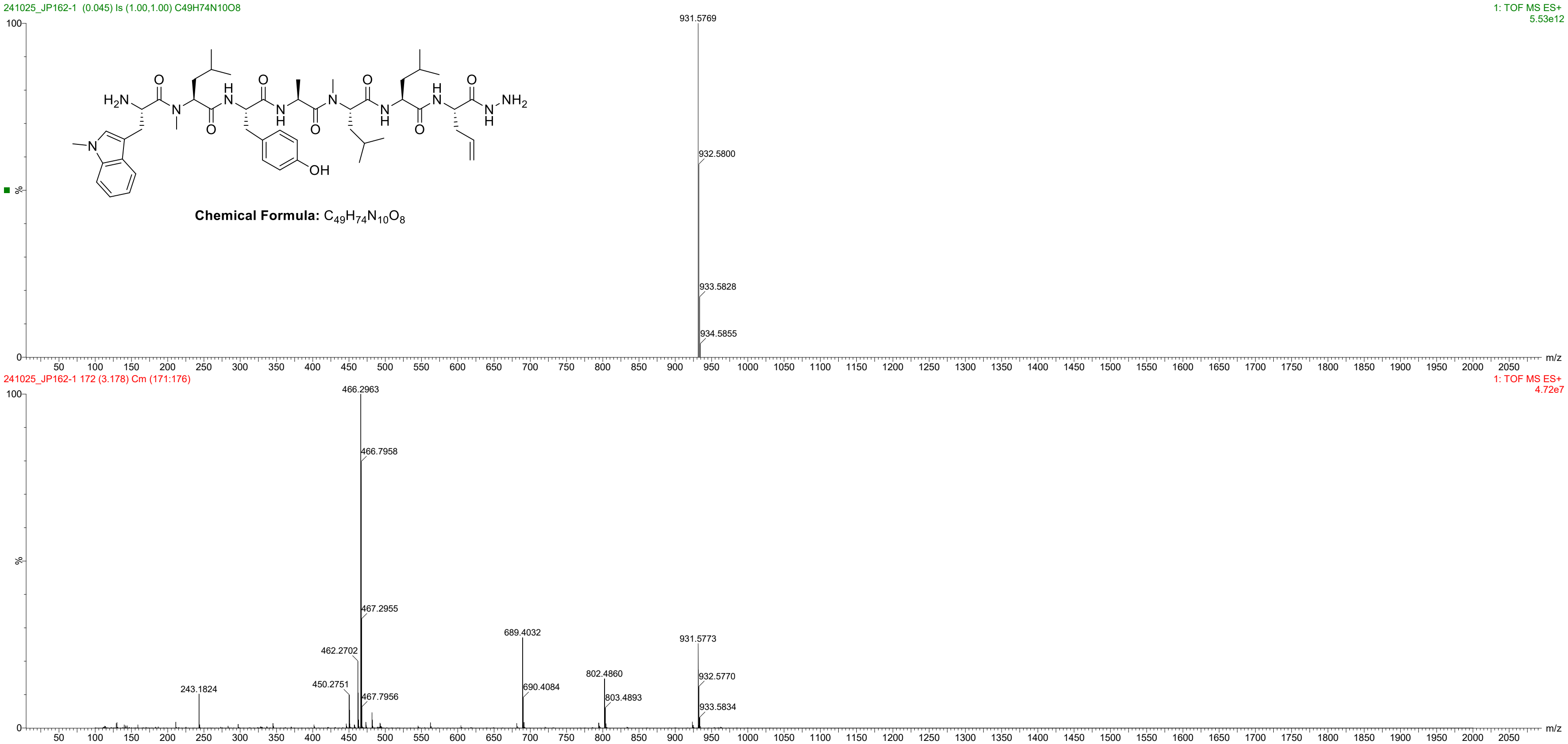

*MS^e of Linear Peptide **9***

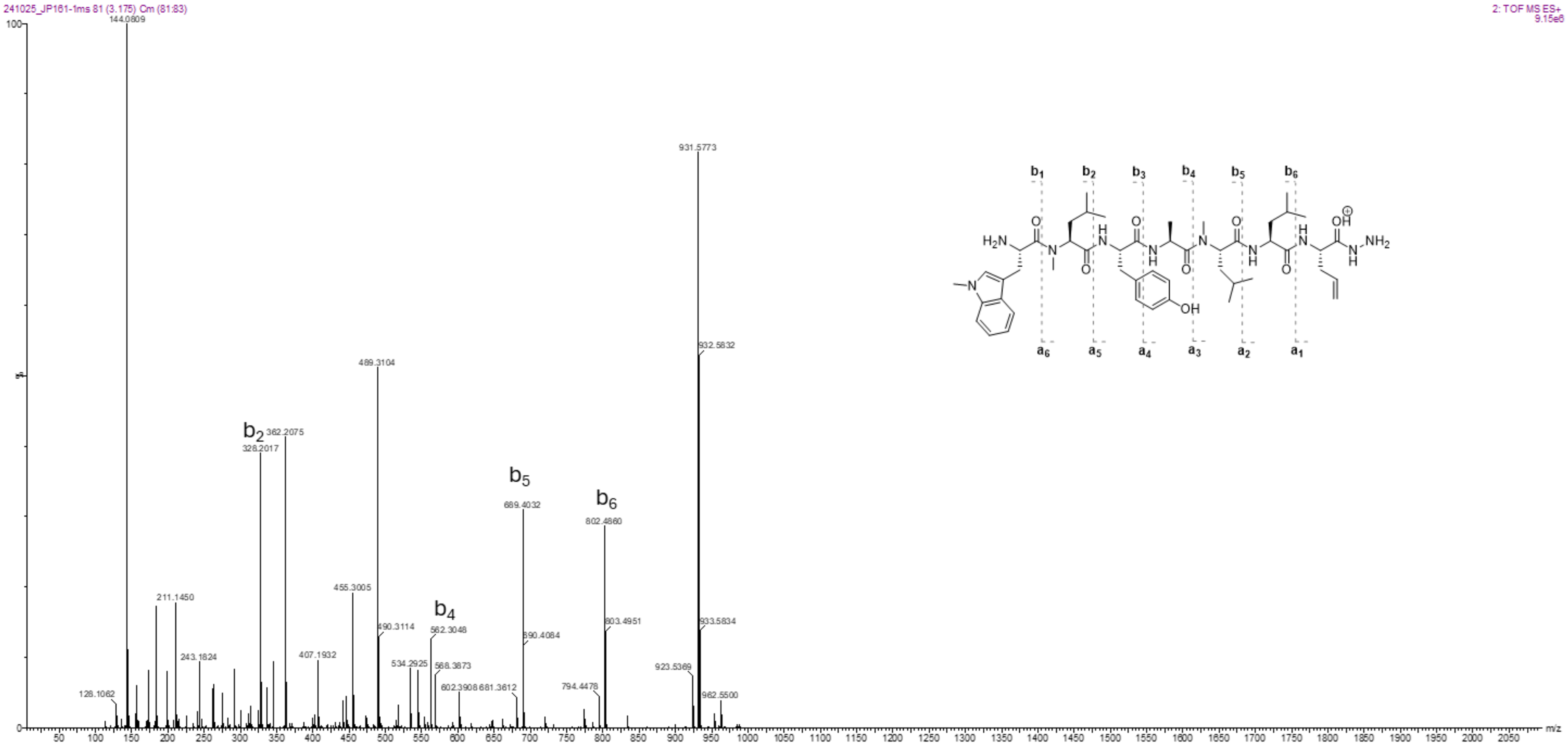

HRMS of Peptide **9** *(top), predicted mass spectrum (middle) measured mass spectrum (bottom)*.

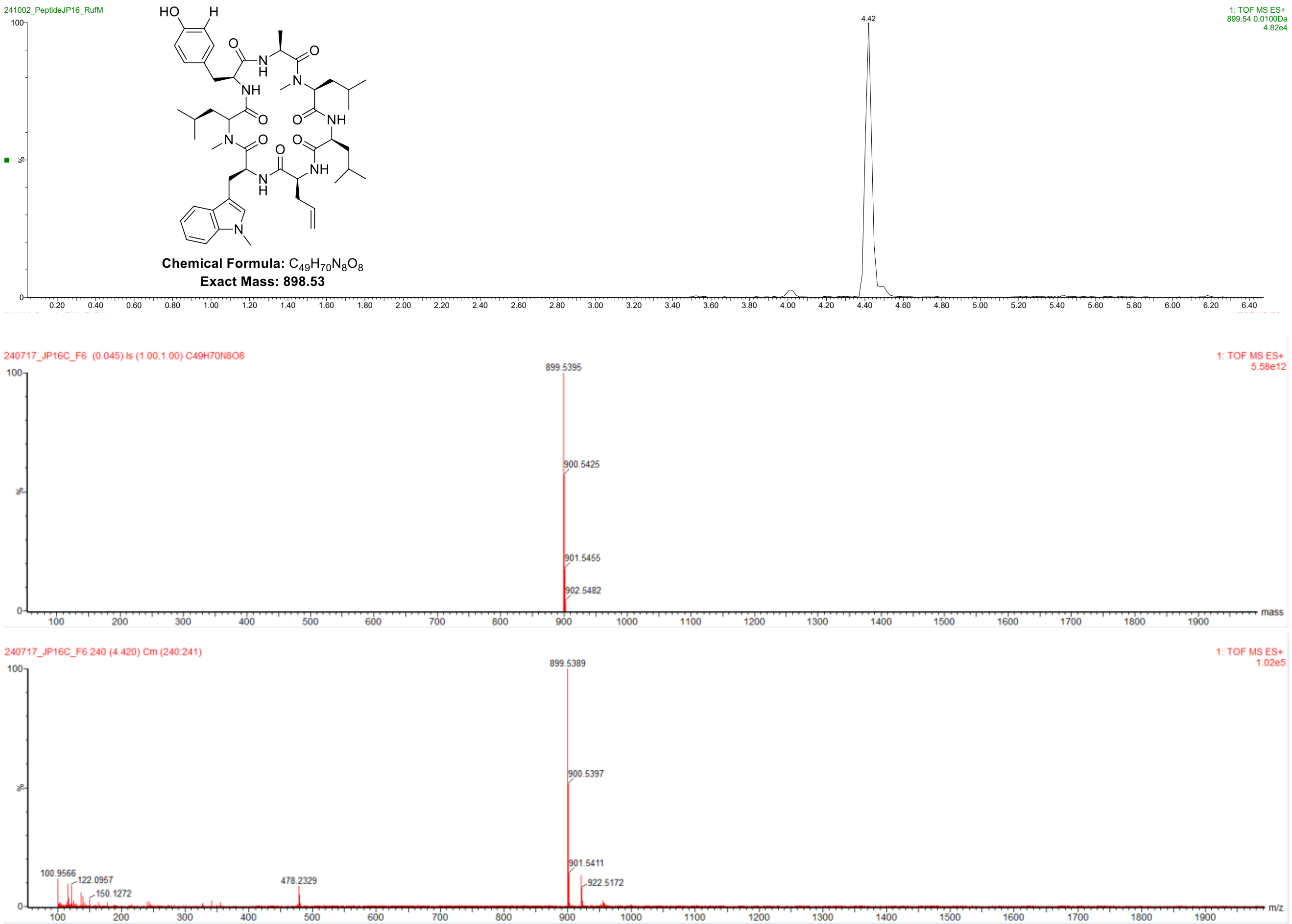

HRMS (ESI) calc. for $C_{49}H_{70}N_8O_8$ calc. for $[M+H]^+$: 899.5395 m/z, found: 899.5389 m/z.

*HRMS Extracted Ion Chromatogram of Peptide **9b/b*** (top), predicted mass spectrum (middle) measured mass spectrum (bottom)*

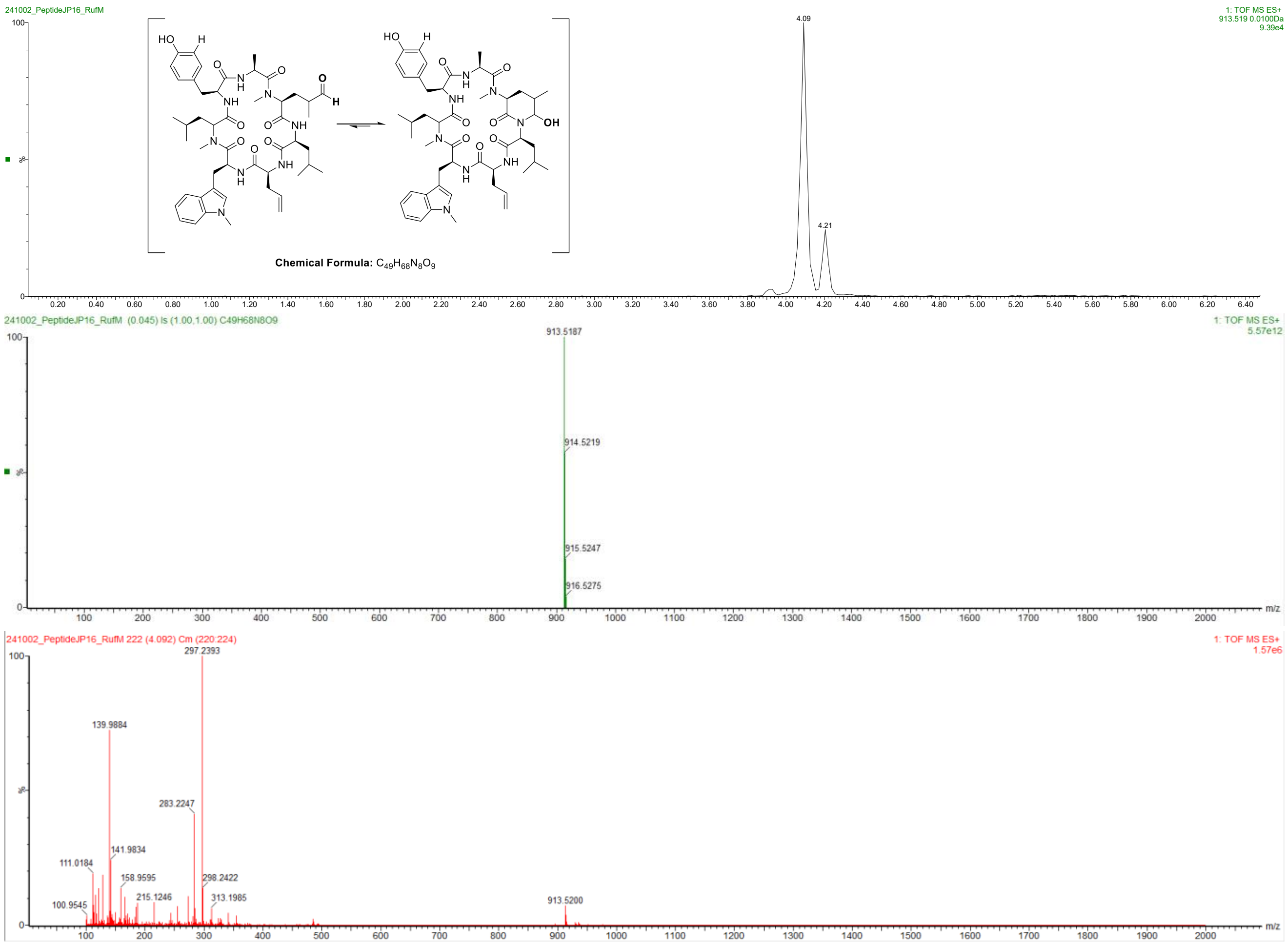

HRMS (ESI) calc. for $C_{54}H_{76}N_{9}O_{10}$ calc. for $[M+H]^{+}$: 913.5187 m/z, found: 913.5200 m/z.

*HRMS Extracted Ion Chromatogram of Peptide **9d** (top), predicted mass spectrum (middle) measured mass spectrum (bottom)*

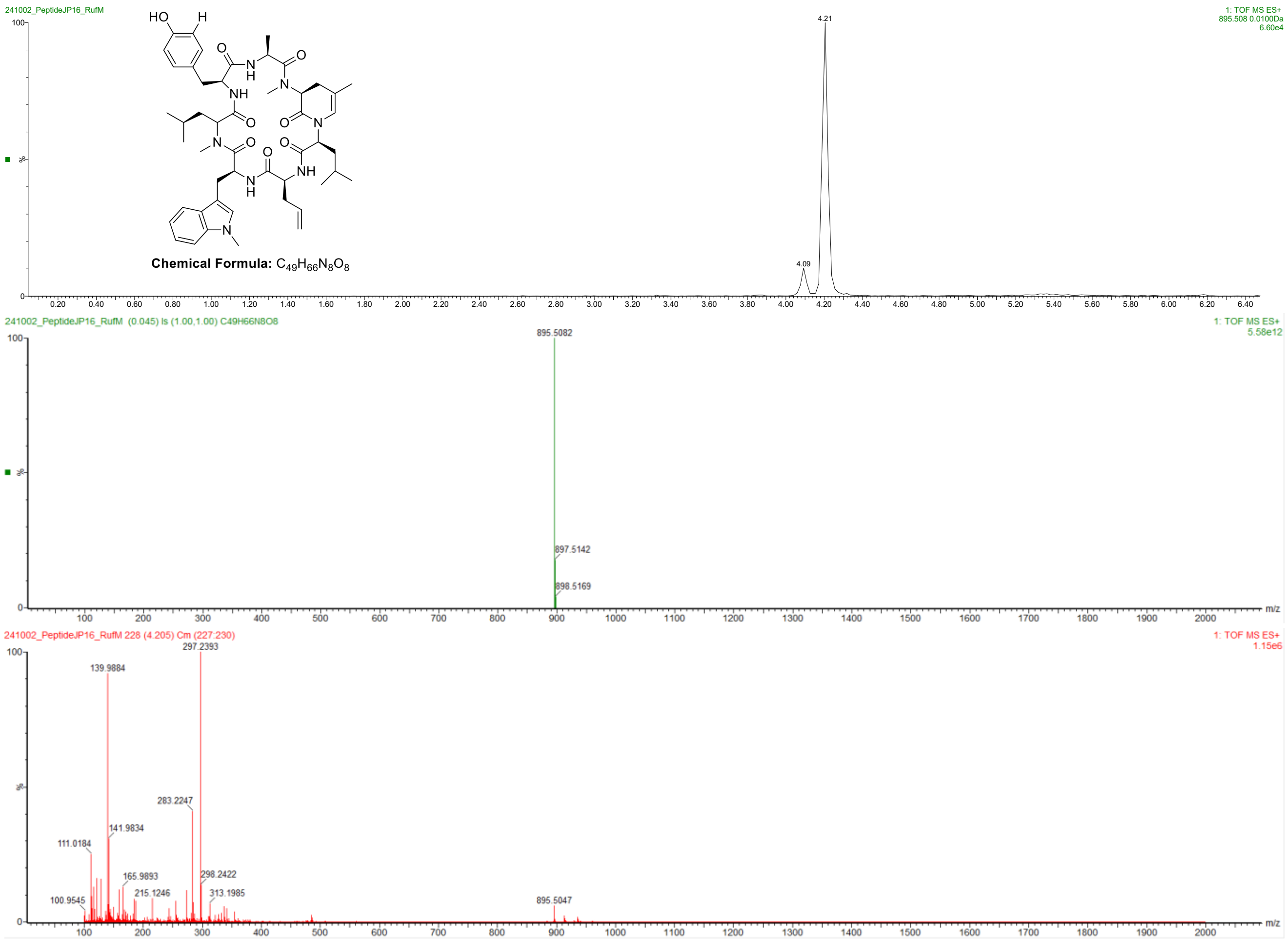

HRMS (ESI) calc. for $C_{54}H_{76}N_9O_{10}$ calc. for $[M+H]^+$: 895.5082 m/z, found: 895.5047 m/z.

*HRMS Extracted Ion Chromatogram of Peptide **9c** (top), predicted mass spectrum (middle) measured mass spectrum (bottom)*

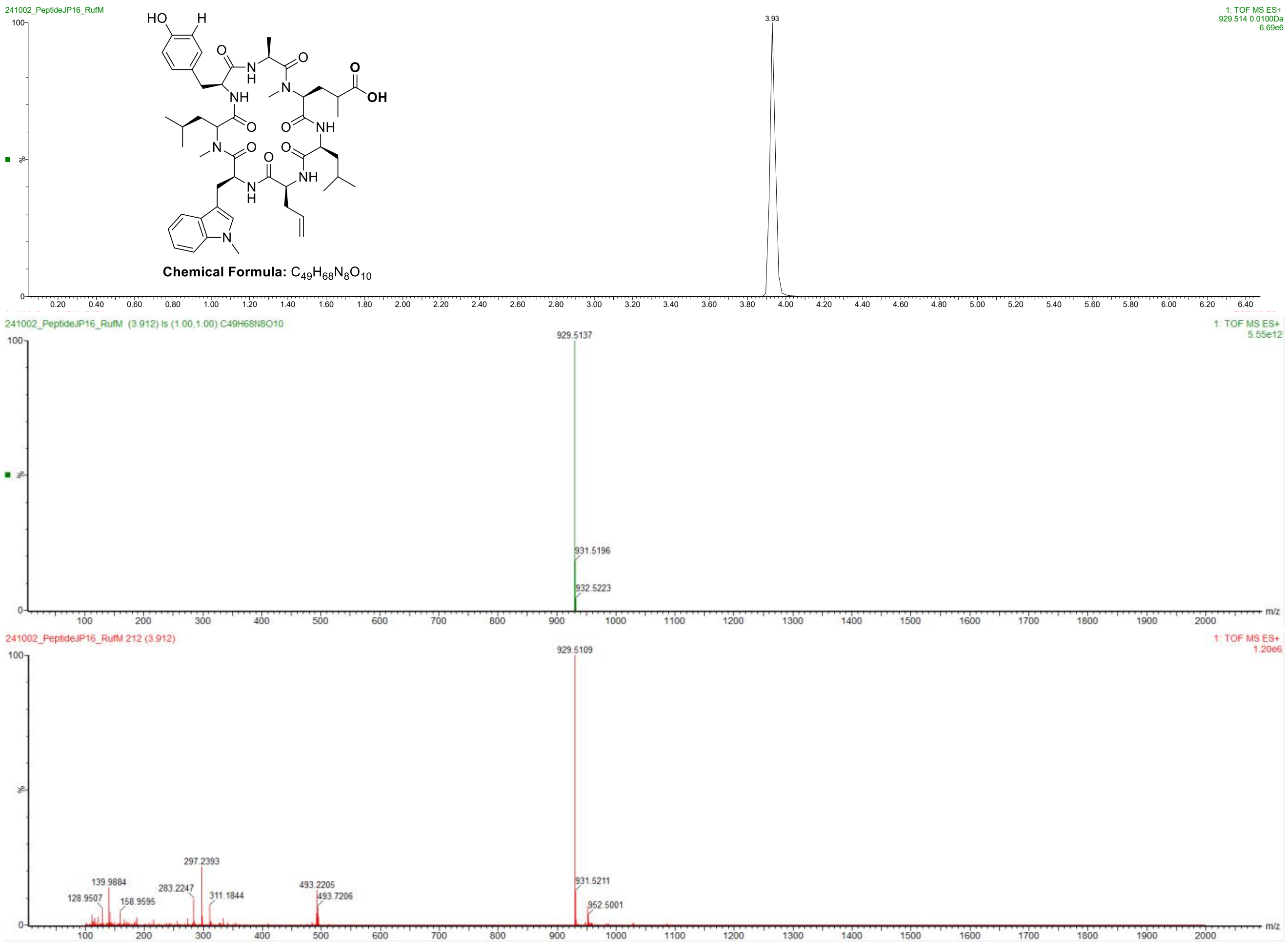

HRMS (ESI) calc. for $C_{54}H_{76}N_9O_{10}$ calc. for $[M+H]^+$: 929.5137 m/z, found: 929.5109 m/z.

*HRMS of Linear peptide **10** (predicted mass spectrum (top) measured (bottom)).*

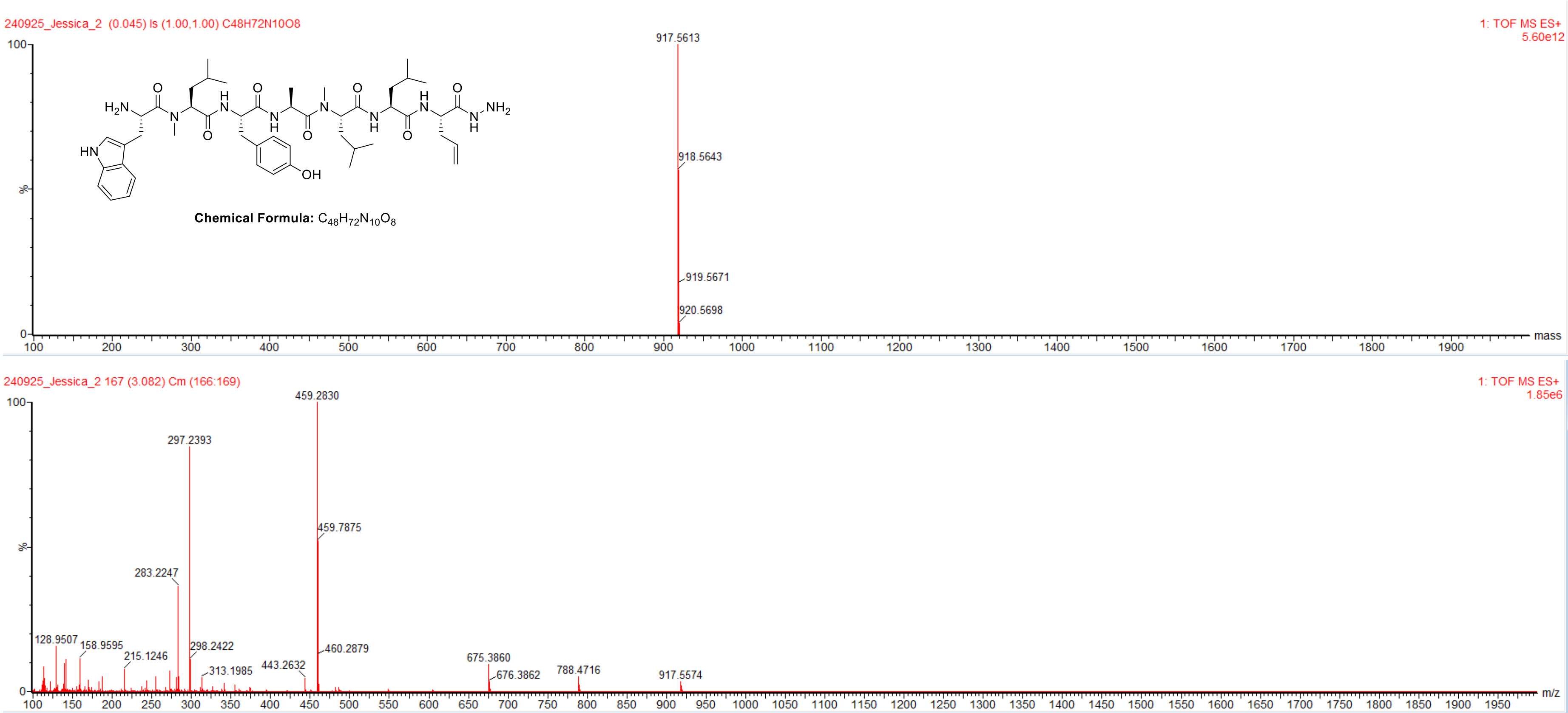

HRMS (ESI) calc. for $C_{48}H_{73}N_{10}O_8$ calc. for $[M+H]^+$: 917.5613 m/z, found: 917.5574 m/z.

*MS/MS of Linear Peptide **10***

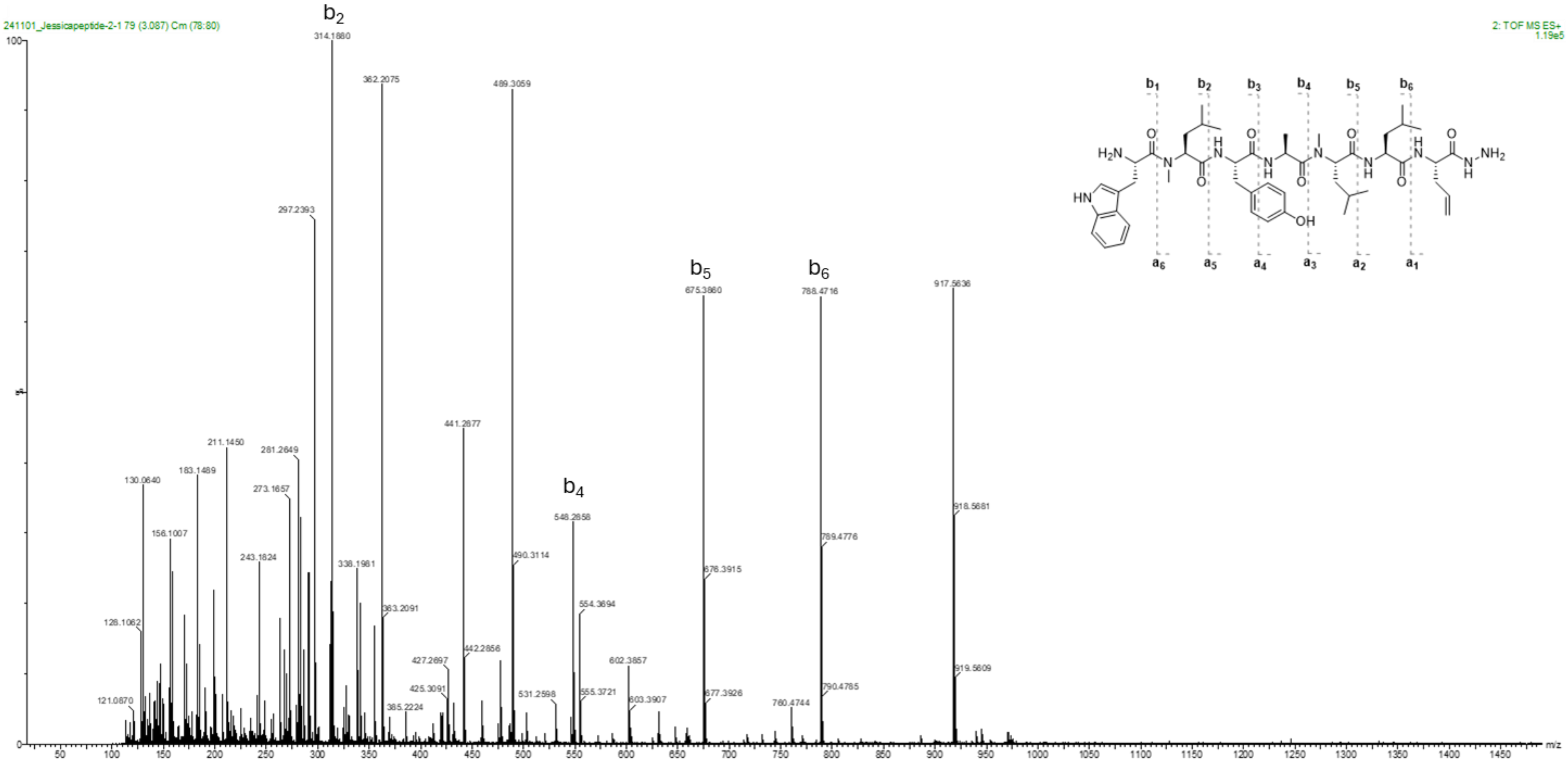

*HRMS Extracted Ion Chromatogram of Peptide **10**(top), predicted mass spectrum (middle) measured mass spectrum (bottom)*

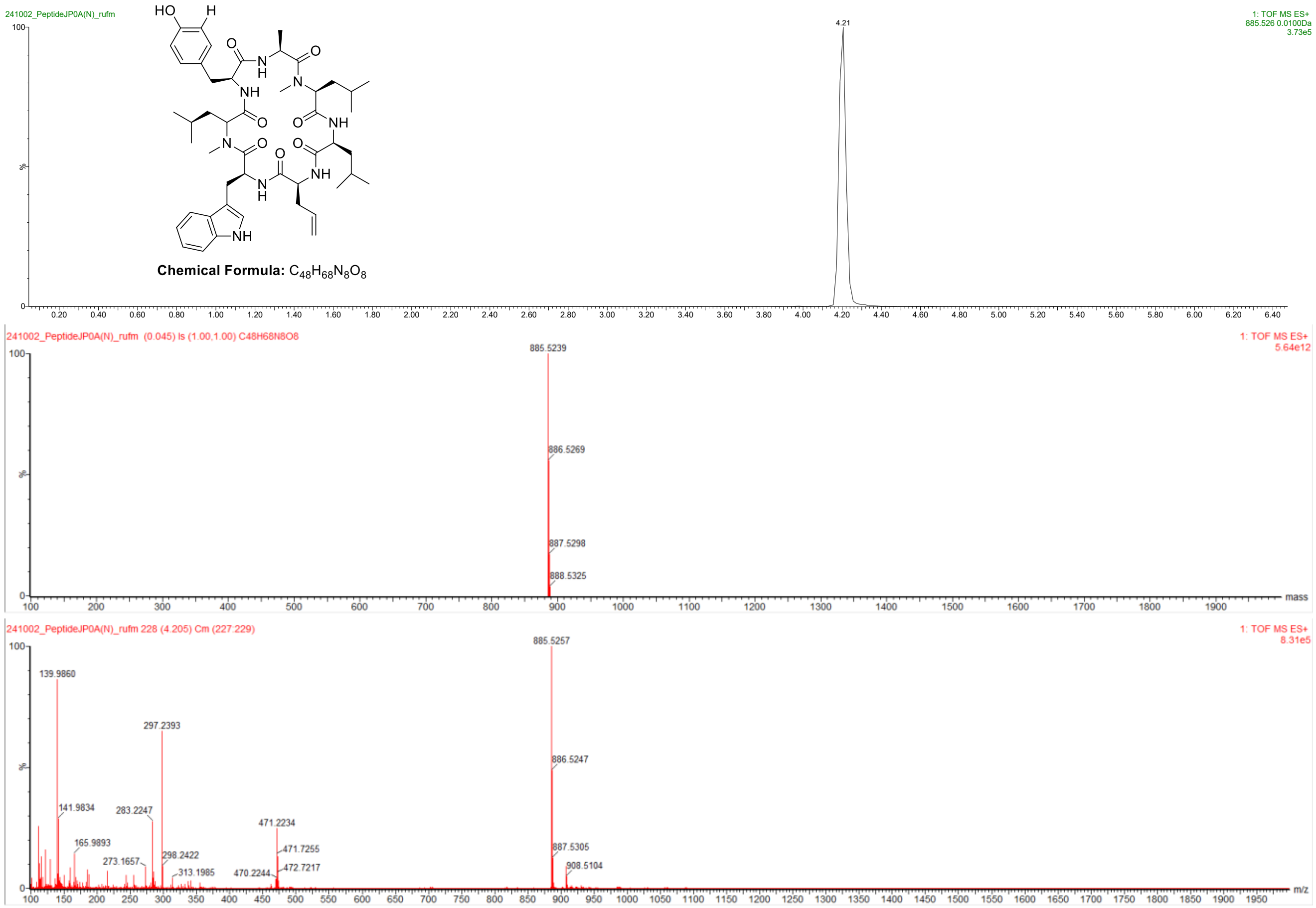

HRMS (ESI) calc. for $C_{48}H_{68}N_8O_8$ calc. for $[M+H]^+$: 885.5239 m/z, found: 885.5257 m/z.

HRMS Extracted Ion Chromatogram of Peptide ***10a*** *(top), predicted mass spectrum (middle) measured mass spectrum (bottom)*

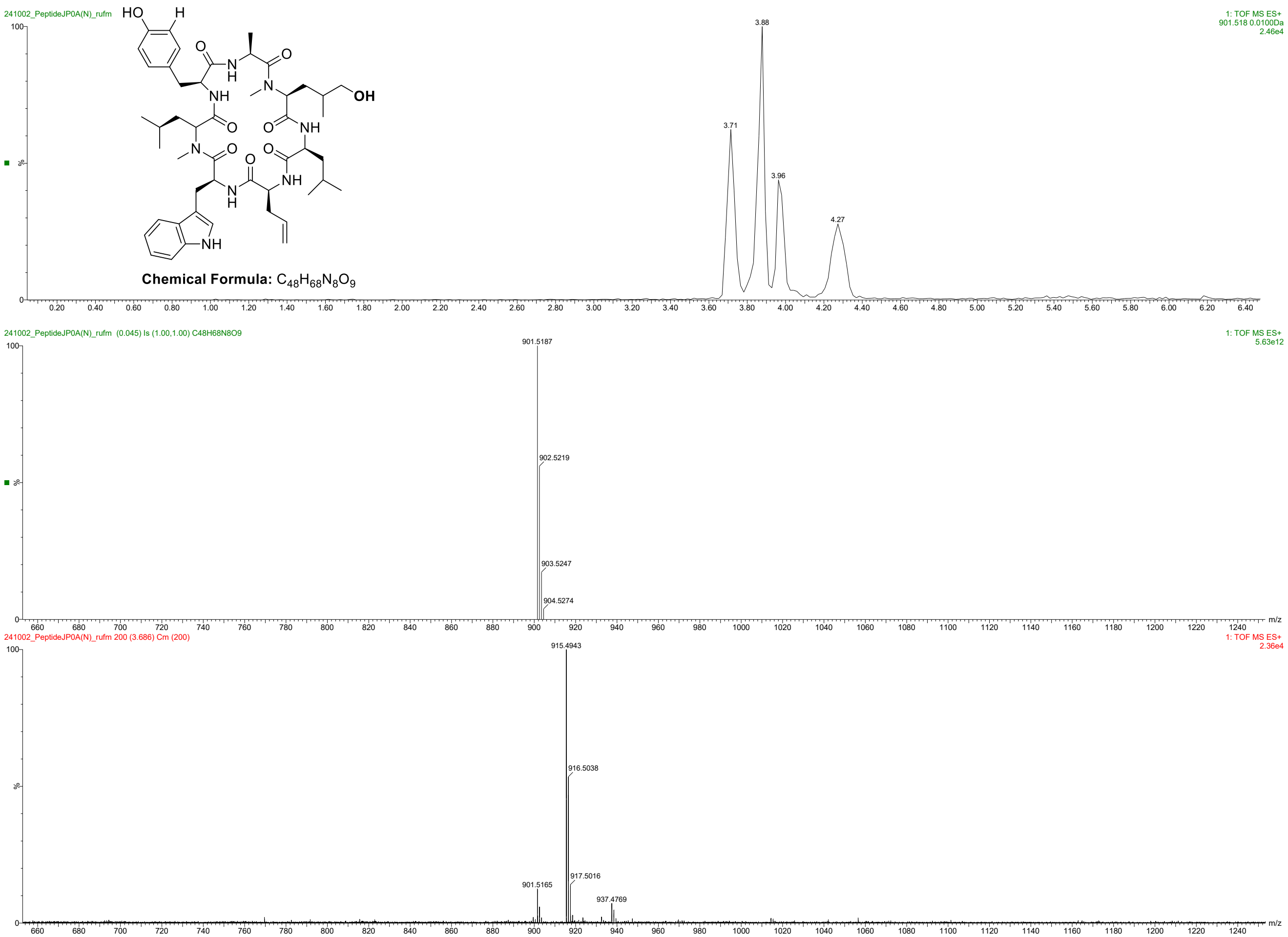

HRMS (ESI) calc. for $C_{48}H_{68}N_8O_9$ calc. for $[M+H]^+$: 901.5187 m/z, found: 901.5165 m/z.

*HRMS extracted Ion Chromatogram of Peptide **10b/b*** ((top), predicted mass spectrum (middle) measured mass spectrum (bottom)*

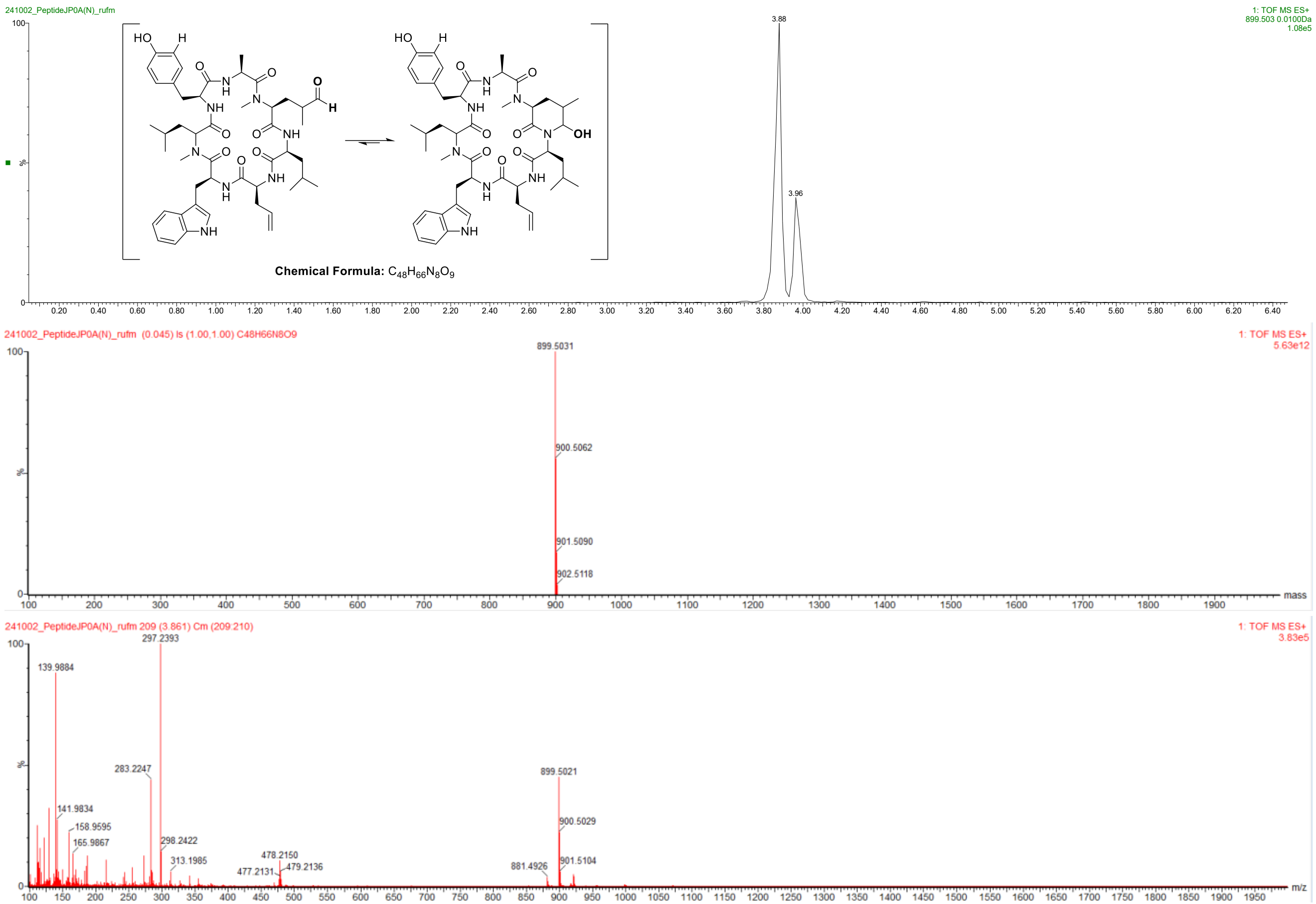

HRMS (ESI) calc. for $C_{48}H_{66}N_8O_9$ calc. for $[M+H]^+$: 899.5031 m/z, found: 899.5021 m/z.

*HRMS extracted Ion Chromatogram of Peptide **10d**(top), predicted mass spectrum (middle) measured mass spectrum (bottom)*

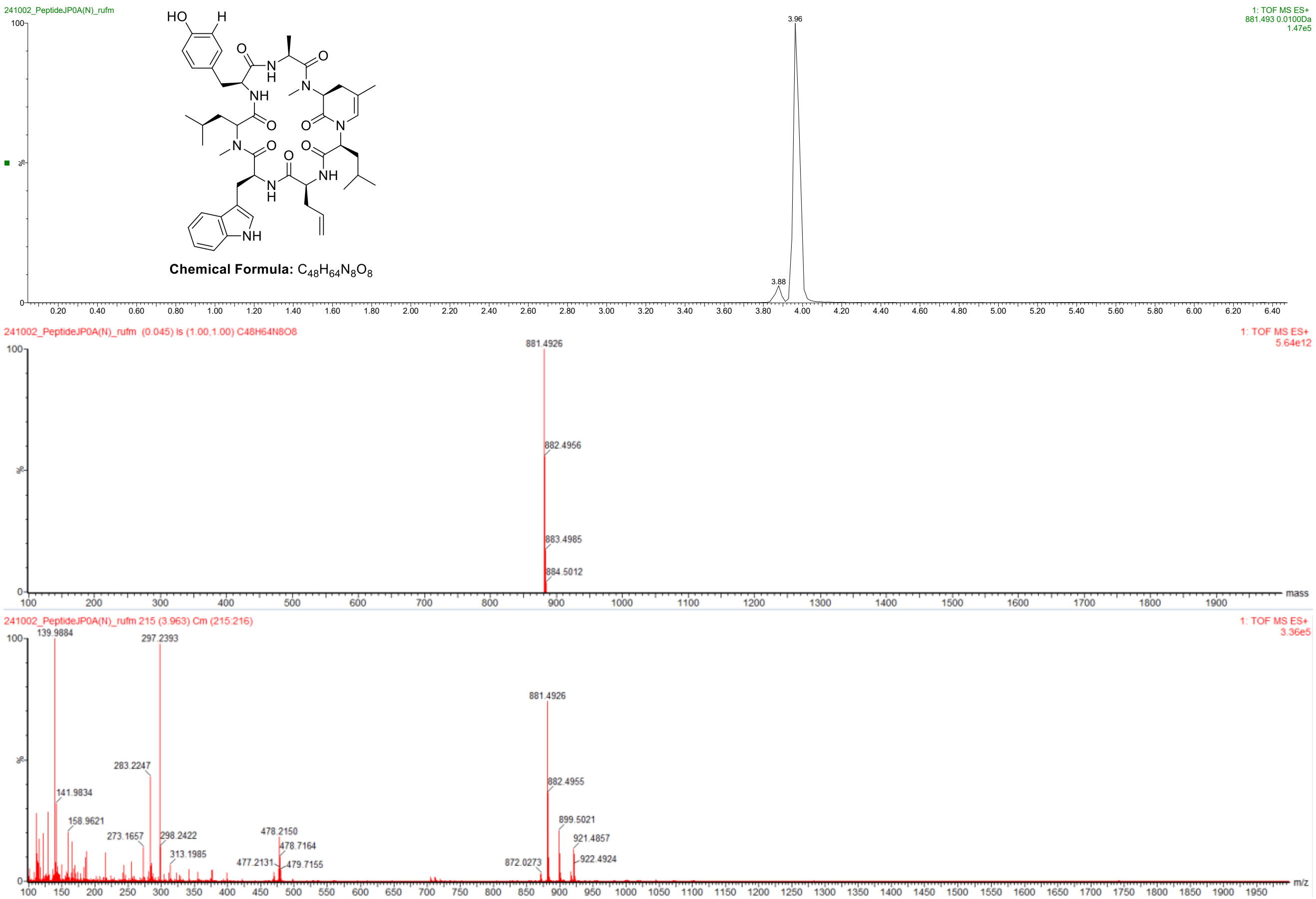

HRMS (ESI) calc. for $C_{48}H_{64}N_8O_8$ calc. for $[M+H]^+$: 881.4926 m/z, found: 881.4926 m/z.

*HRMS Extracted Ion Chromatogram of Peptide **10c** (predicted mass spectrum (top) measured (bottom))*

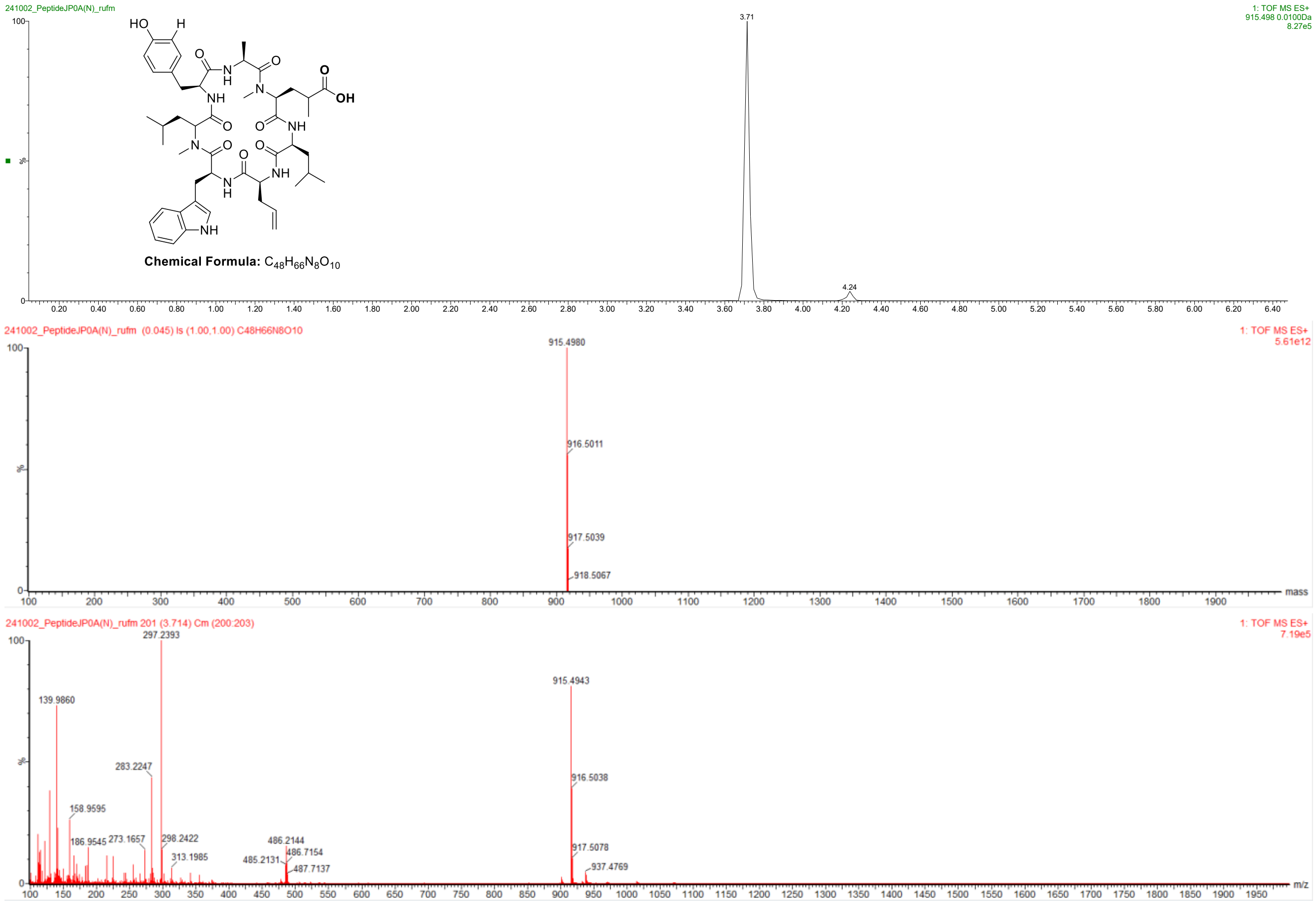

HRMS (ESI) calc. for $C_{48}H_{66}N_8O_{10}$ calc. for $[M+H]^+$: 915.4980 m/z, found: 915.4943 m/z.

*HRMS of Linear Peptide **11** predicted mass spectrum (top) measured mass spectrum (bottom)*

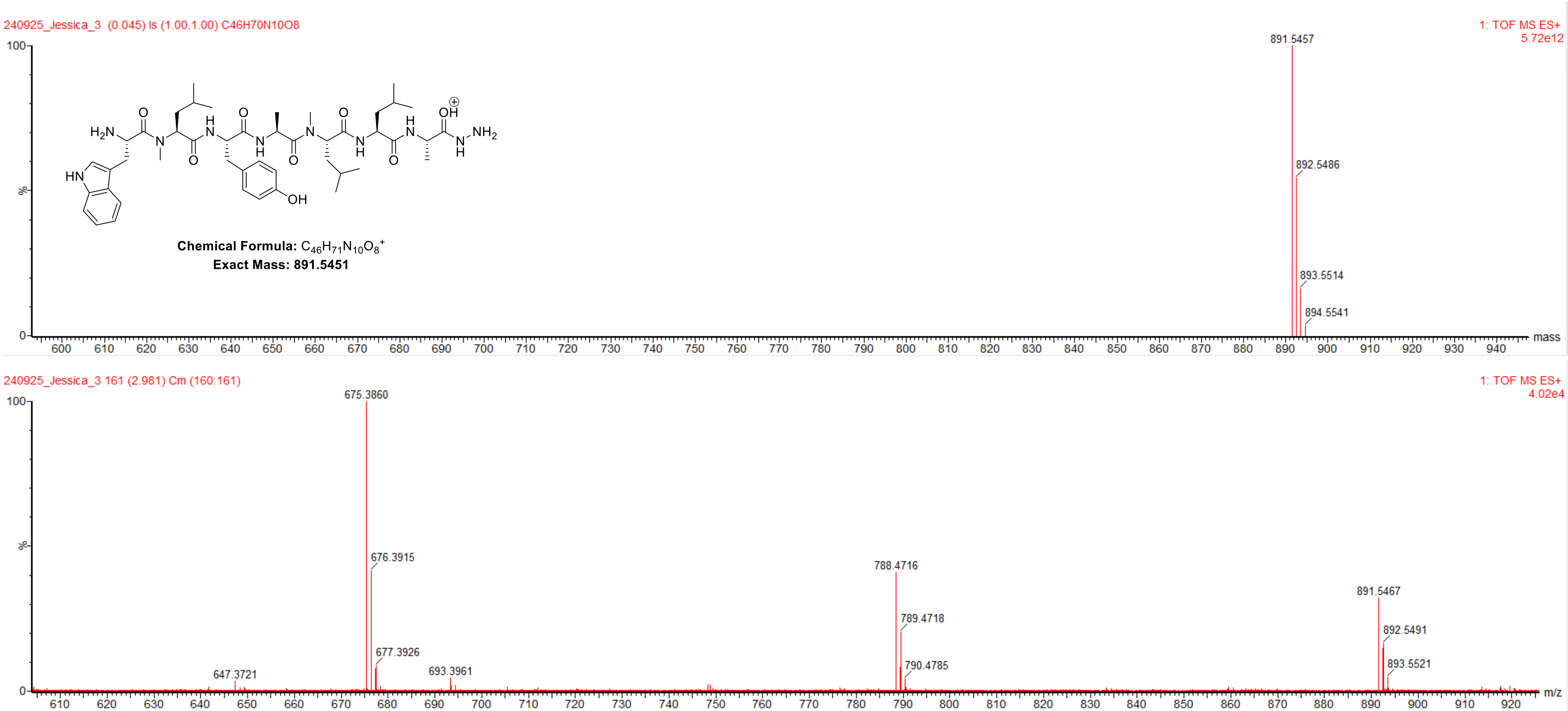

HRMS (ESI) calc. for $C_{49}H_{69}N_8O_8$ calc. for $[M+H]^+$: 891.5457 m/z, found: 891.5467 m/z.

MS^e^ of Linear Peptide 11

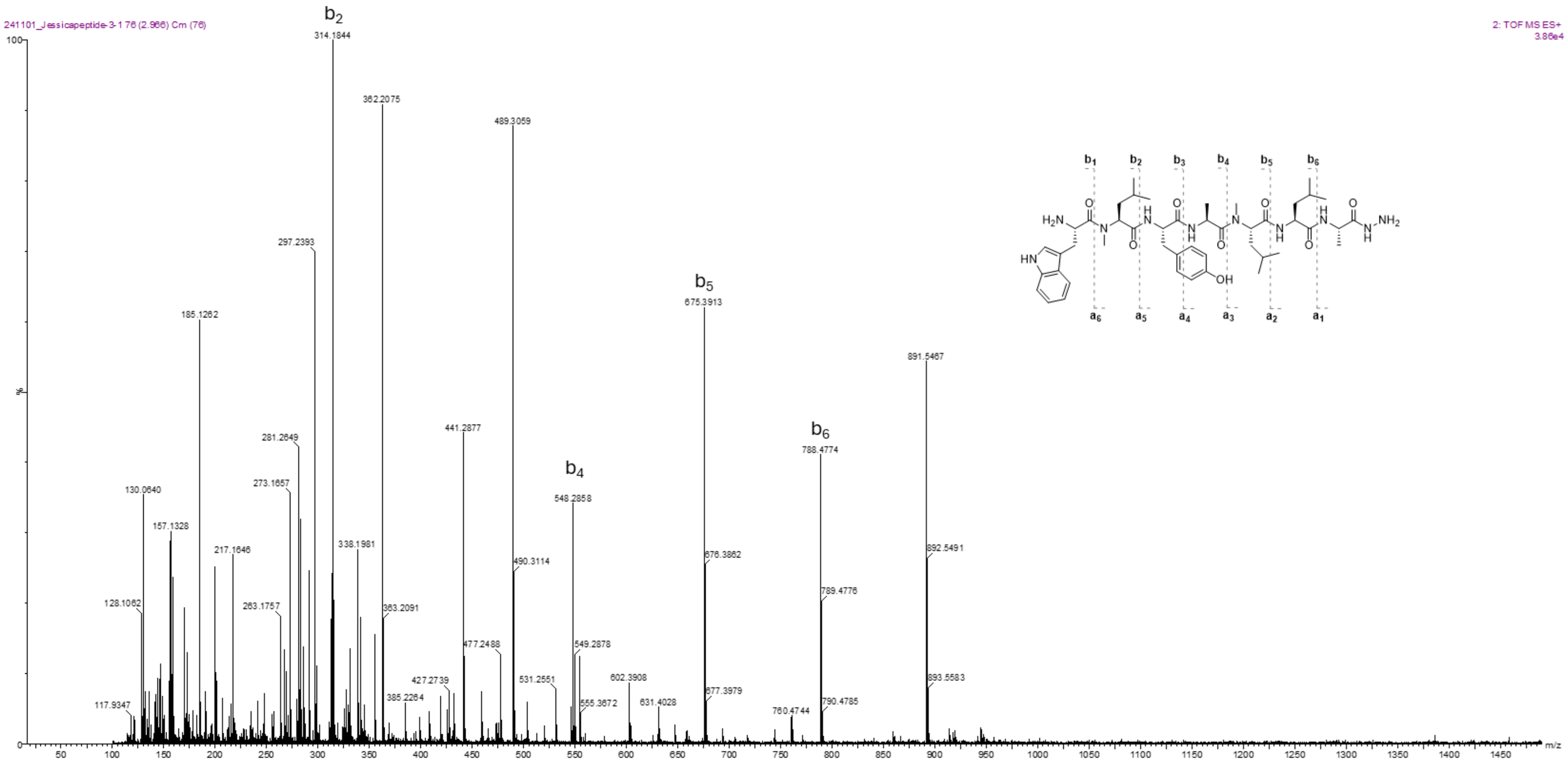

*HRMS of Peptide **11** (top), predicted mass spectrum (middle) measured mass spectrum (bottom)*

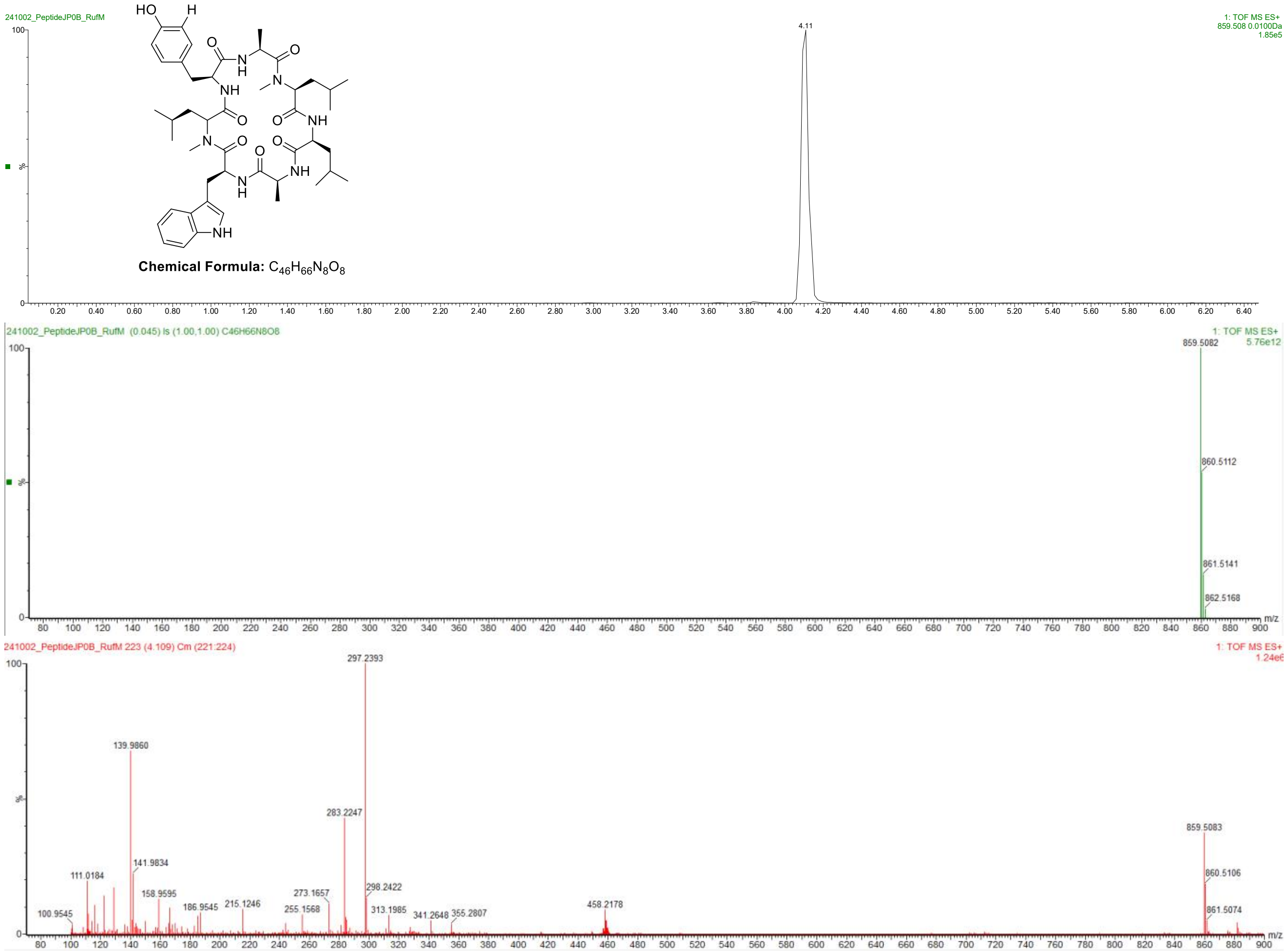

HRMS (ESI) calc. for $C_{54}H_{76}N_9O_{10}$ calc. for $[M+H]^+$: 859.5082 m/z, found: 859.5083 m/z.

*HRMS Extracted Ion Chromatogram of Peptide **11a** (top), predicted mass spectrum (middle) measured mass spectrum (bottom)*

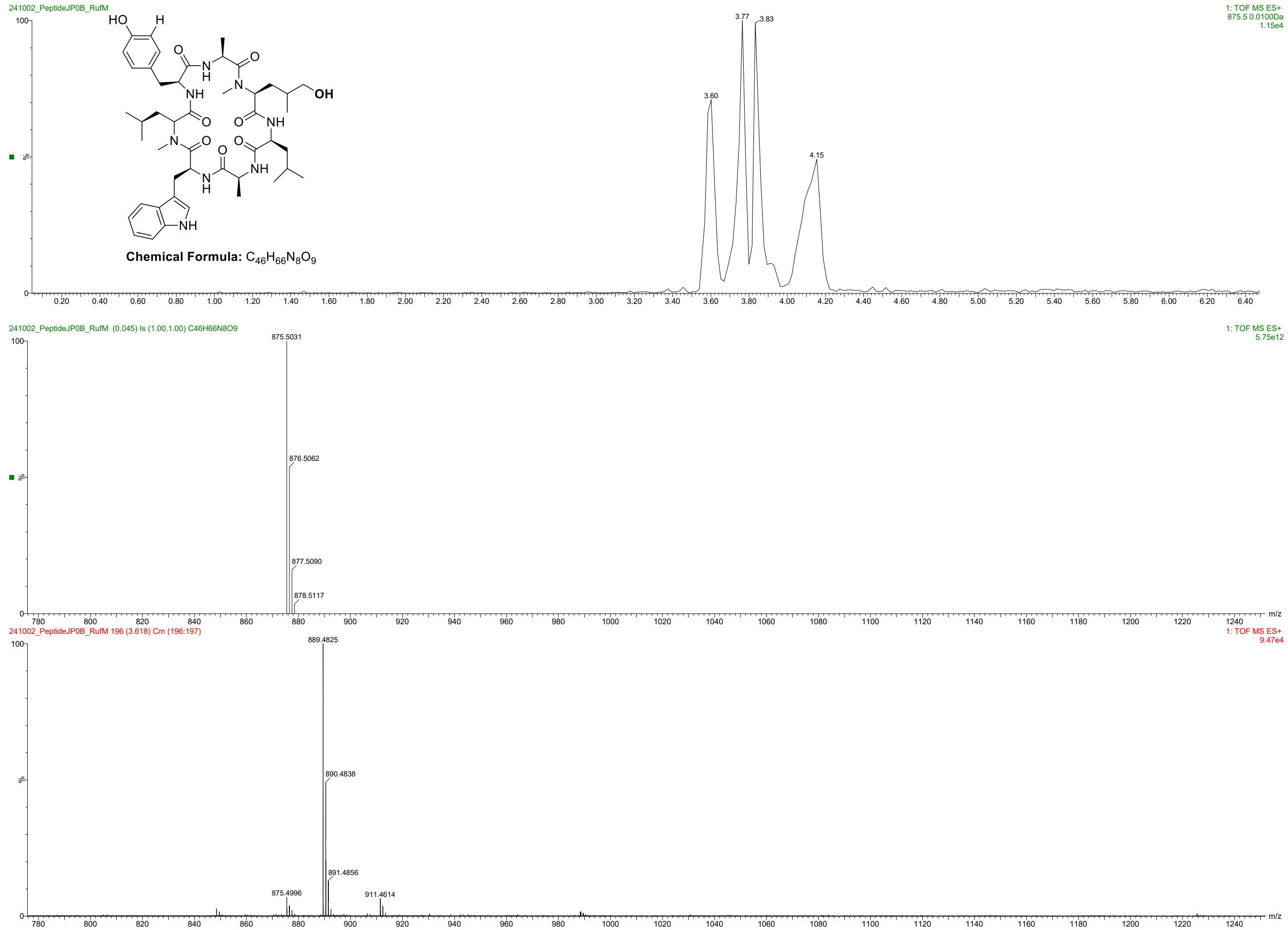

HRMS (ESI) calc. for $C_{46}H_{66}N_8O_9$ calc. for $[M+H]^+$: 875.5031 m/z, found: 875.4996 m/z.

*HRMS Extracted Ion Chromatogram of Peptide **11b/b*** (top), predicted mass spectrum (middle) measured mass spectrum (bottom)*

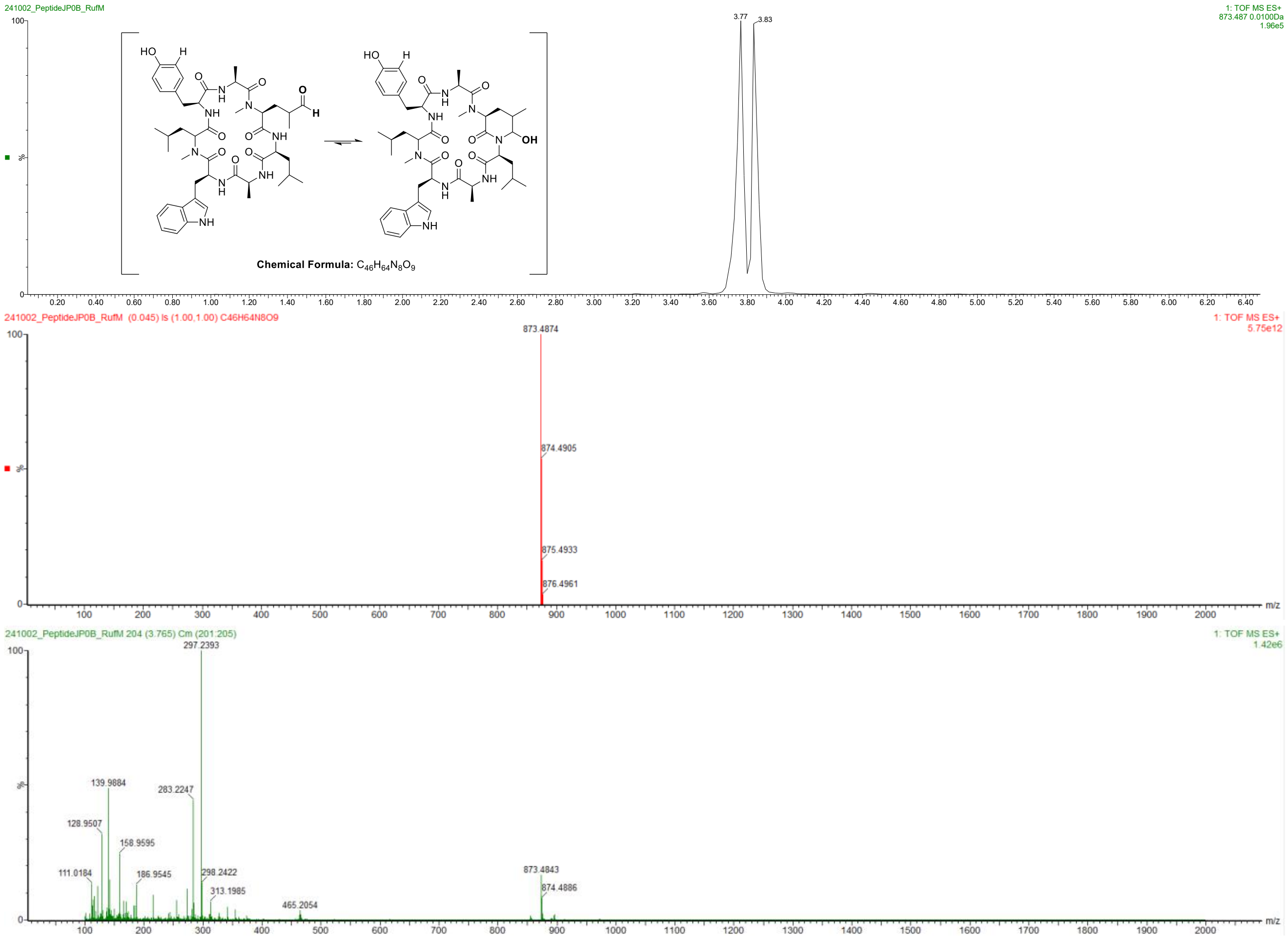

HRMS (ESI) calc. for $C_{54}H_{76}N_9O_{10}$ calc. for $[M+H]^+$: 873.4874 m/z, found: 873.4843 m/z.

*HRMS Extracted Ion Chromatogram of Peptide **11c** top), predicted mass spectrum (middle) measured mass spectrum (bottom)*

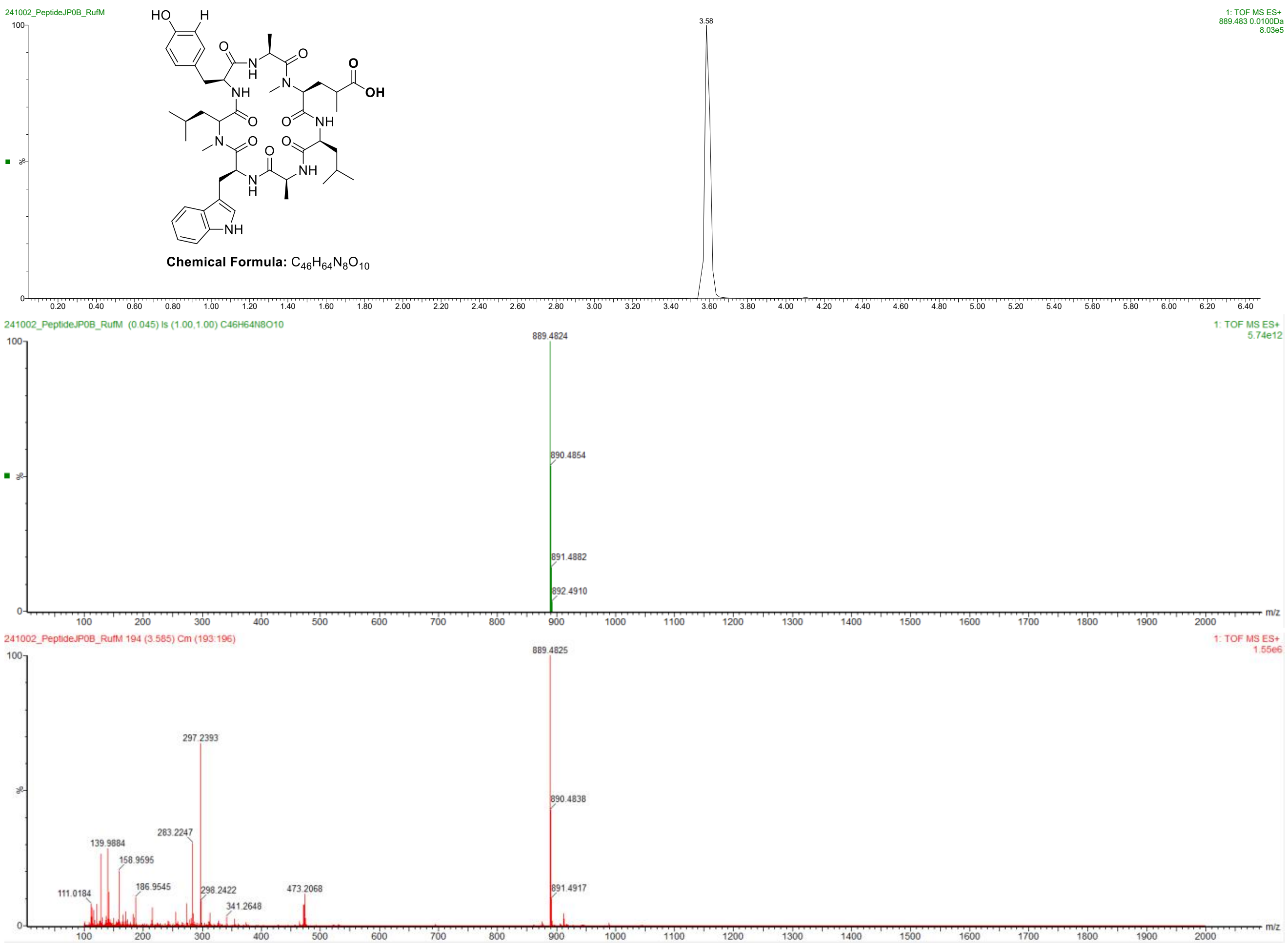

HRMS (ESI) calc. for $C_{46}H_{64}N_8O_{10}$ calc. for $[M+H]^+$: 889.4824 m/z, found: 889.4825 m/z.

*HRMS Extracted Ion Chromatogram of Peptide **12** (top), predicted mass spectrum (middle) measured mass spectrum (bottom).*

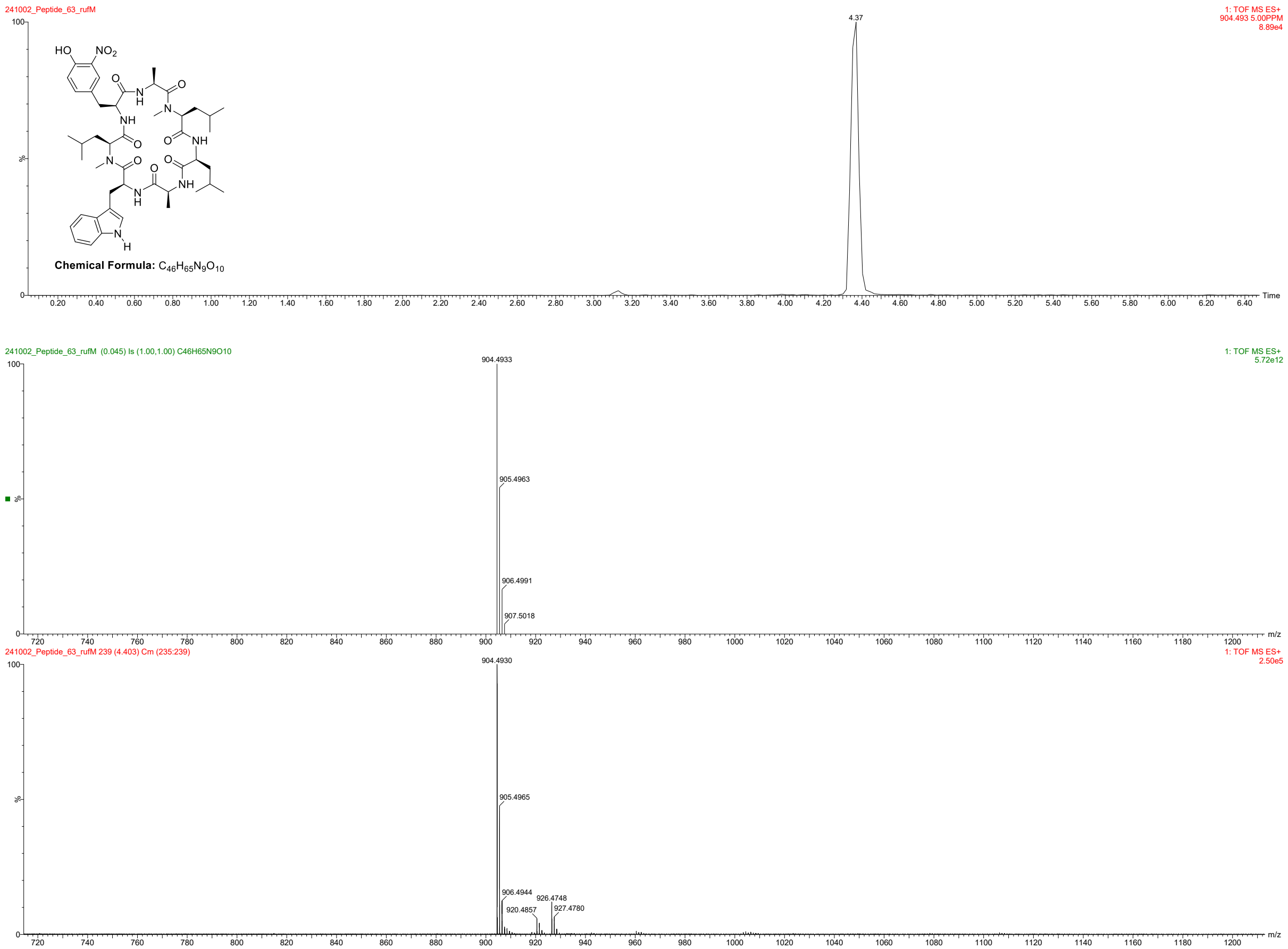

HRMS (ESI) calc. for $C_{46}H_{65}N_9O_{10}$ calc. for $[M+H]^+$: 904.4933 m/z, found: 904.4930 m/z.

*HRMS Extracted Ion Chromatogram of Peptide **12a**(top), predicted mass spectrum (middle) measured mass spectrum (bottom).*

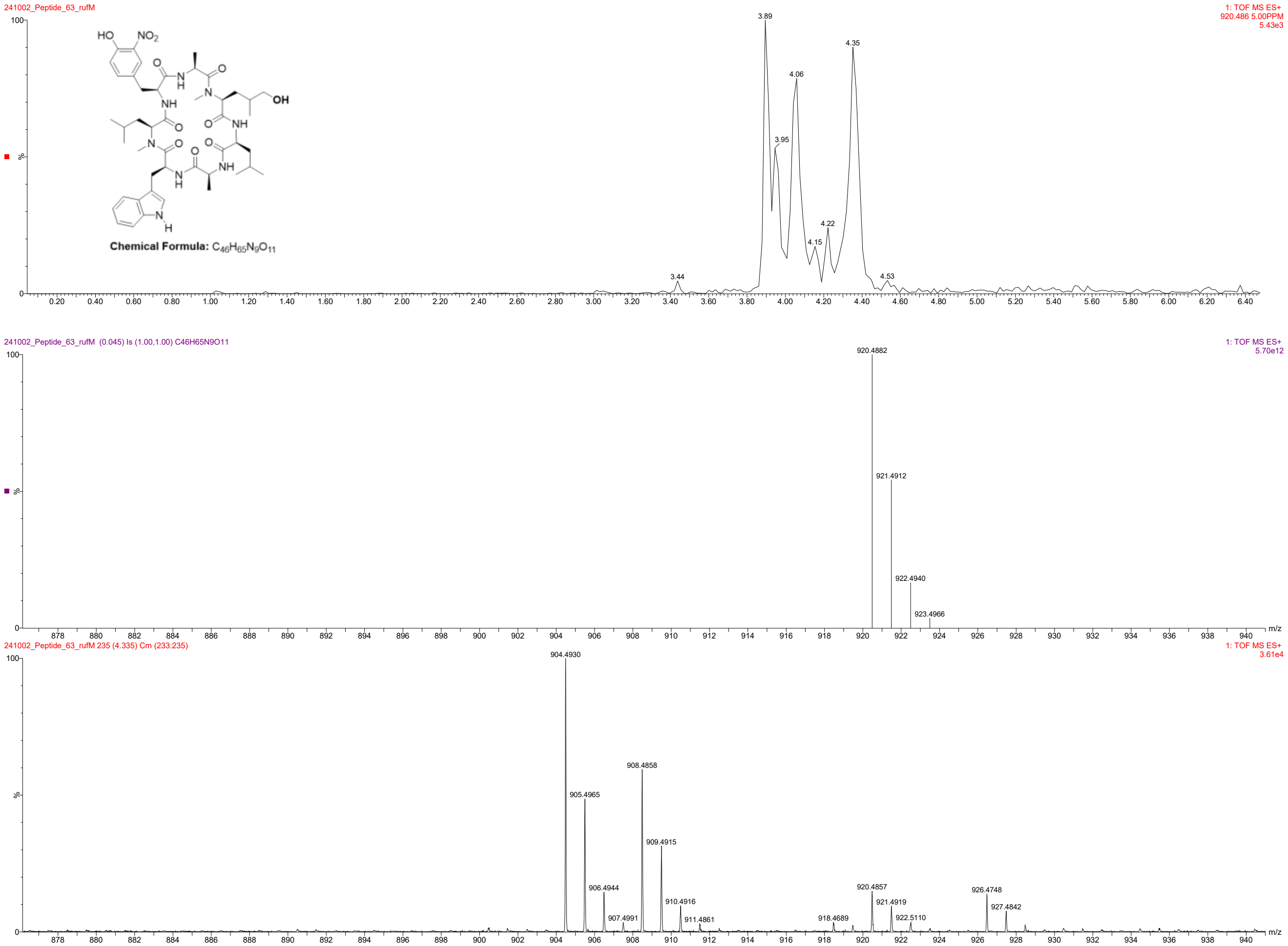

HRMS (ESI) calc. for $C_{46}H_{65}N_9O_{11}$ calc. for $[M+H]^+$: 920.4882 m/z, found: 920.4857 m/z.

*HRMS extracted Ion Chromatogram of Peptide **12b/b*** (top), predicted mass spectrum (middle) measured mass spectrum (bottom).*

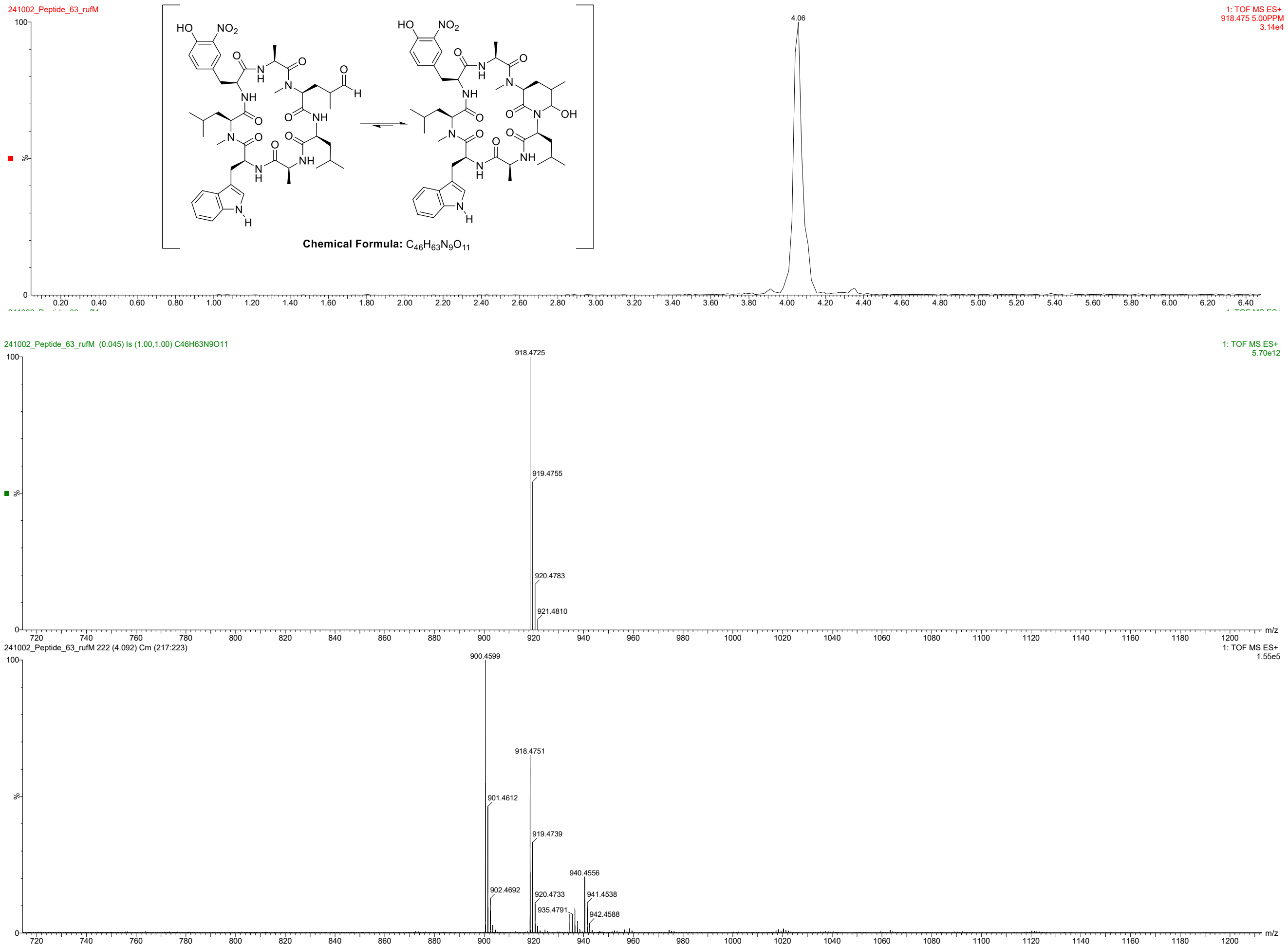

HRMS (ESI) calc. for $C_{46}H_{61}N_9O_{10}$ calc. for $[M+H]^+$: 918.4725 m/z, found: 918.4751 m/z.

*HRMS extracted Ion Chromatogram of Peptide **12d** (top), predicted mass spectrum (middle) measured mass spectrum (bottom).*

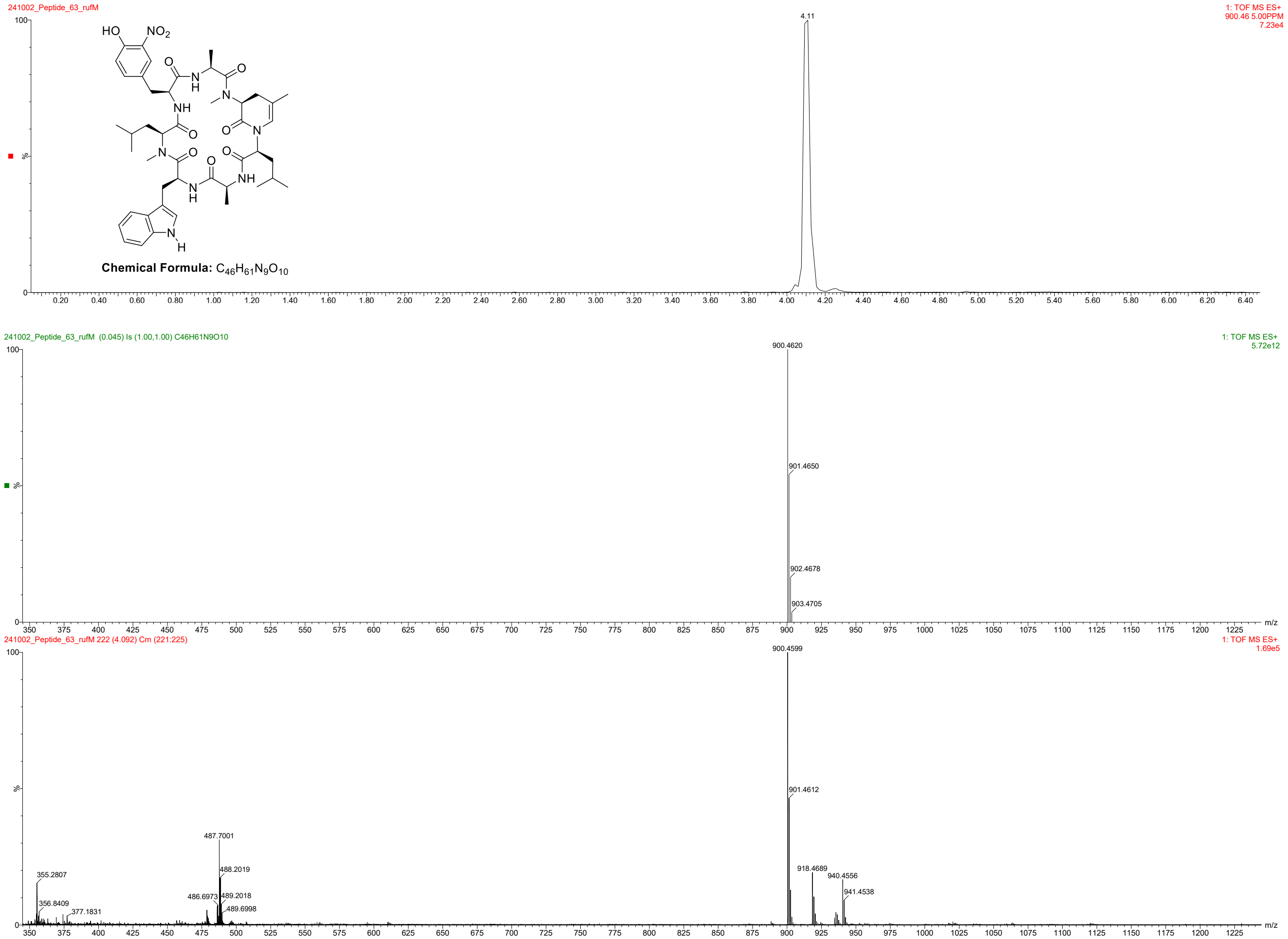

HRMS (ESI) calc. for $C_{46}H_{61}N_9O_{10}$ calc. for $[M+H]^+$: 900.4620 m/z, found: 900.4599 m/z.

HRMS extracted Ion Chromatogram of Peptide ***12c*** *(top), predicted mass spectrum (middle) measured mass spectrum (bottom)*

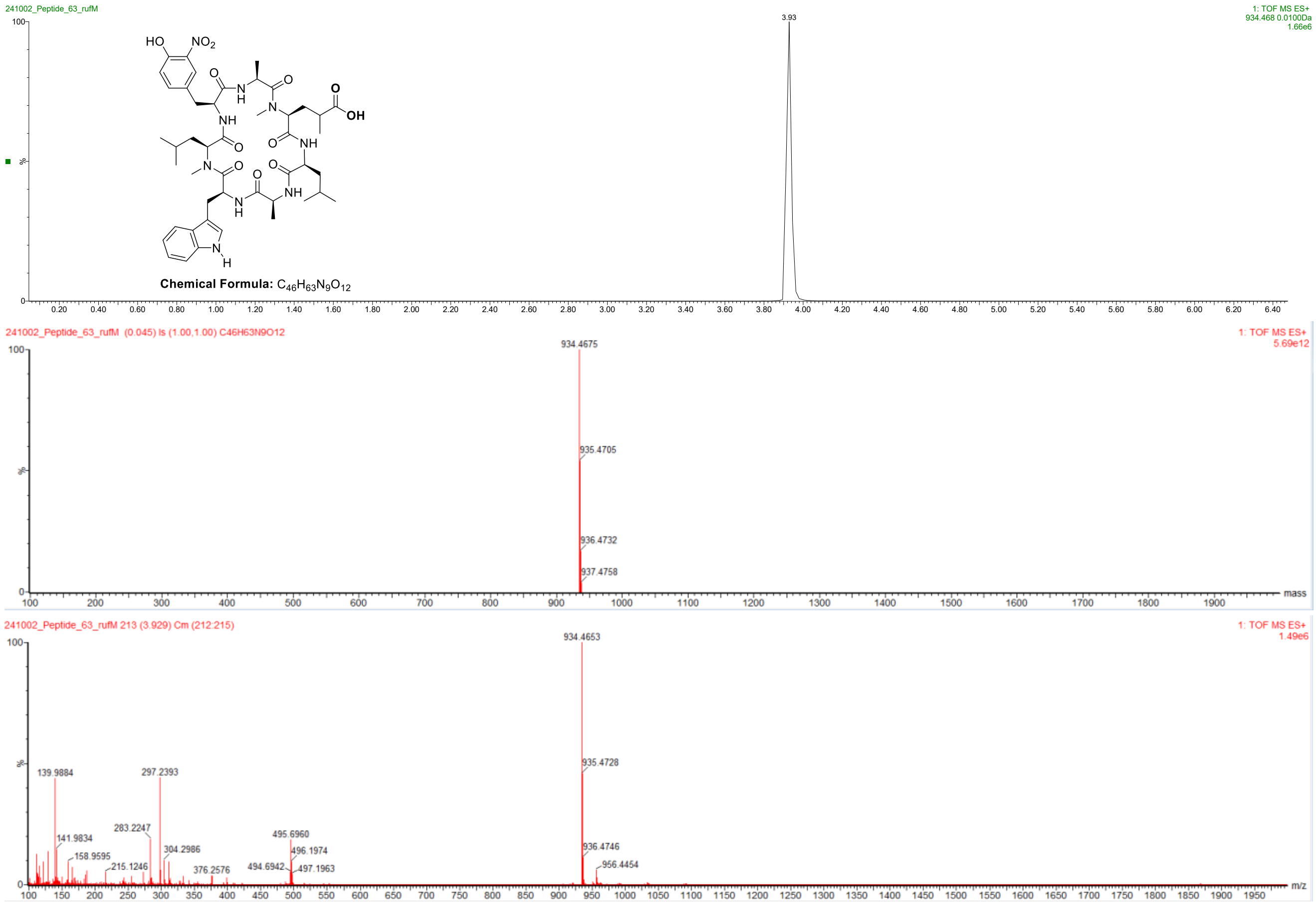

HRMS (ESI) calc. for $C_{54}H_{76}N_9O_{10}$ calc. for $[M+H]^+$: 934.4675 m/z, found: 934.4653 m/z.

*HRMS of Peptide **13** (top), predicted mass spectrum (middle) measured mass spectrum (bottom)*

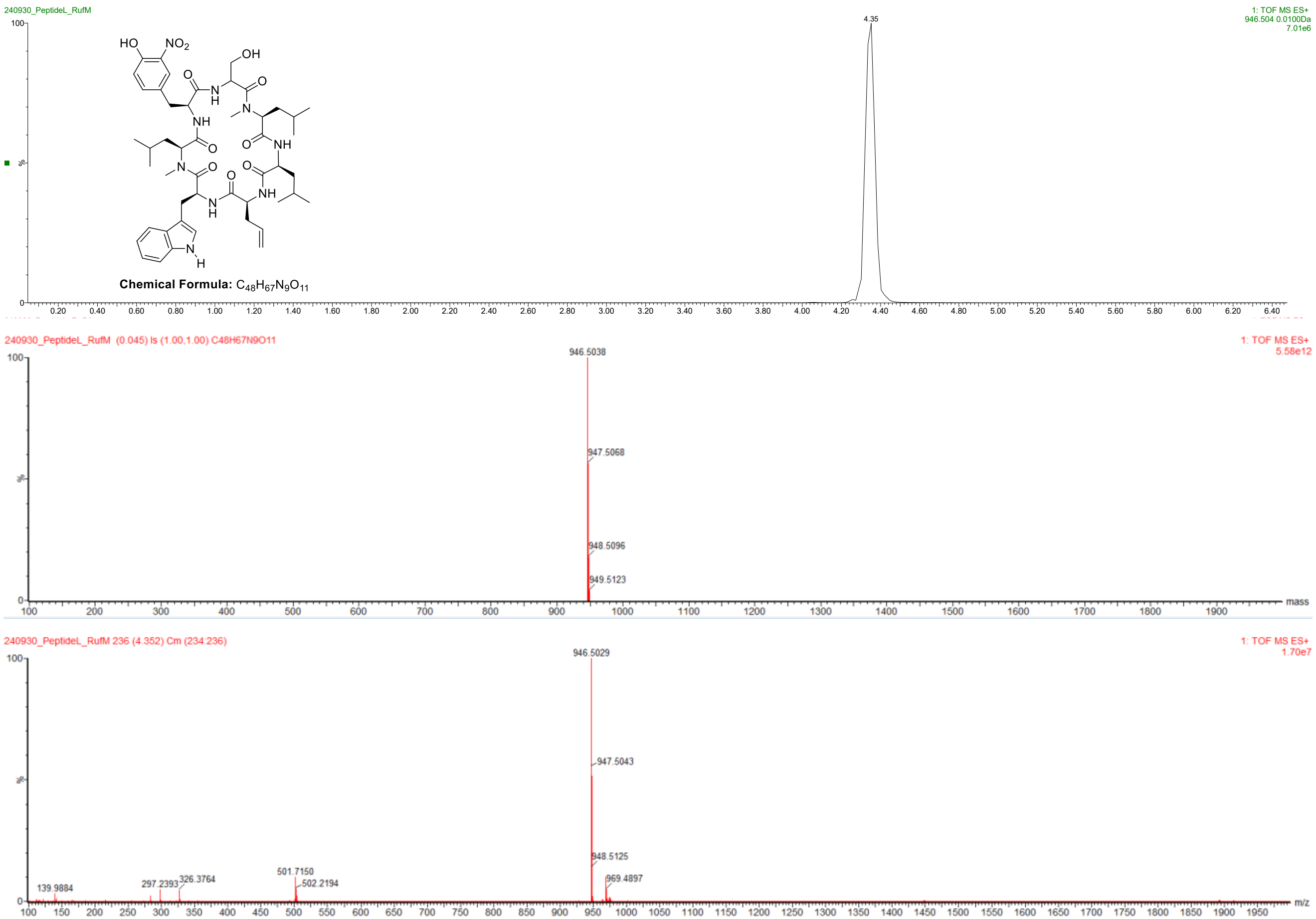

HRMS (ESI) calc. for $C_{48}H_{67}N_9O_{11}$ calc. for $[M+H]^+$: 946.5029 m/z, found: 946.5038 m/z.

HRMS Extracted Ion Chromatogram of Peptide ***13a*** *(top), predicted mass spectrum (middle) measured mass spectrum (bottom)*

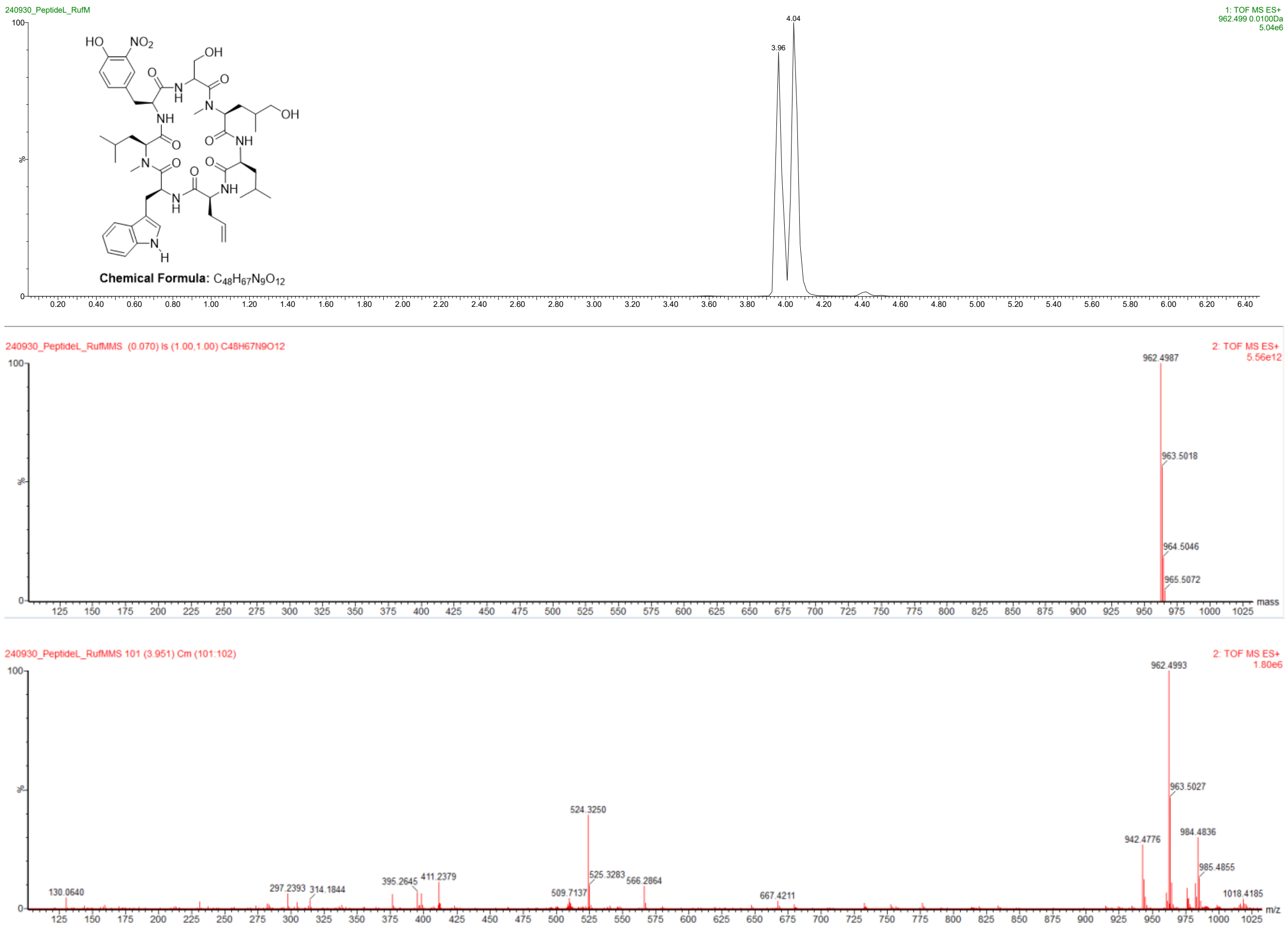

HRMS (ESI) calc. for $C_{48}H_{67}N_{9}O_{12}$ calc. for $[M+H]^{+}$: 962.4987 m/z, found: 962.4993 m/z.

*HRMS extracted Ion Chromatogram of Peptide **13e** (top), predicted mass spectrum (middle) measured mass spectrum (bottom)*

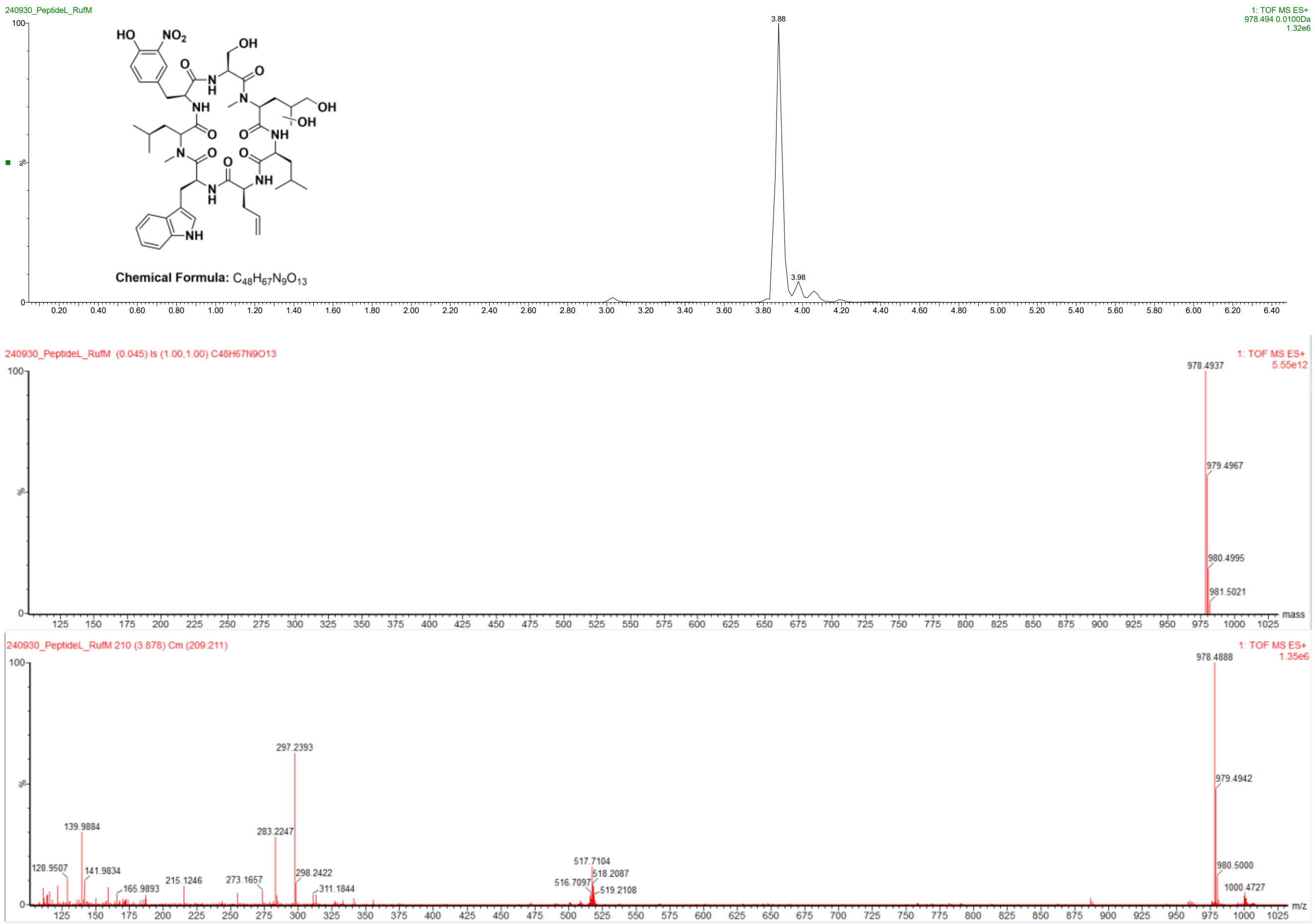

HRMS (ESI) calc. for $C_{48}H_{67}N_9O_{13}$ calc. for $[M+H]^+$: 978.4937 m/z, found: 978.4888 m/z.

*HRMS extracted Ion Chromatogram of Peptide **13b/b*** (top), predicted mass spectrum (middle) measured mass spectrum (bottom).*

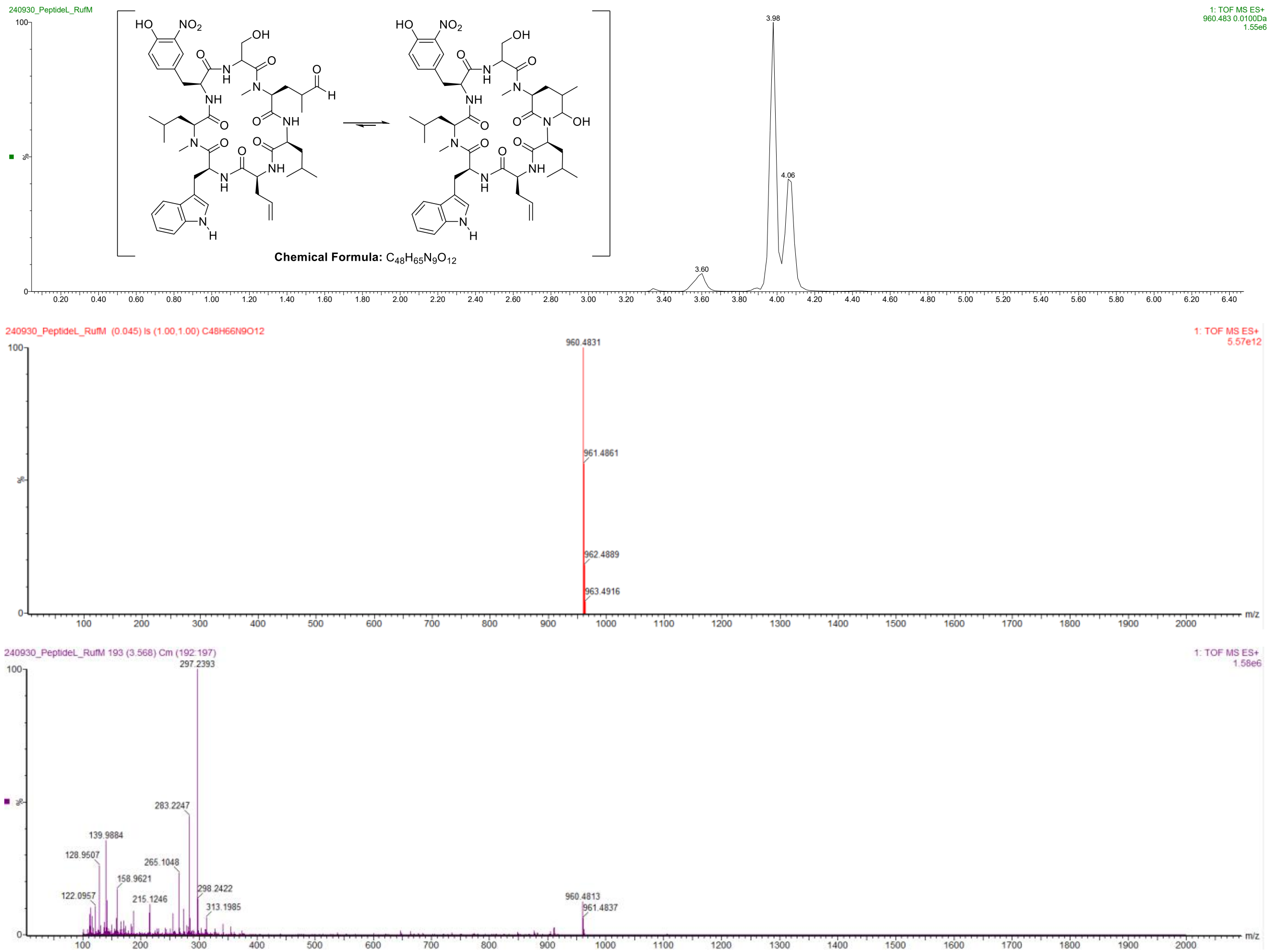

HRMS (ESI) calc. for $C_{48}H_{67}N_9O_{13}$ calc. for $[M+H]^+$: 960.4813 m/z, found: 960.4831 m/z.

*HRMS extracted Ion Chromatogram of Peptide **13d** (top), predicted mass spectrum (middle) measured mass spectrum (bottom)*

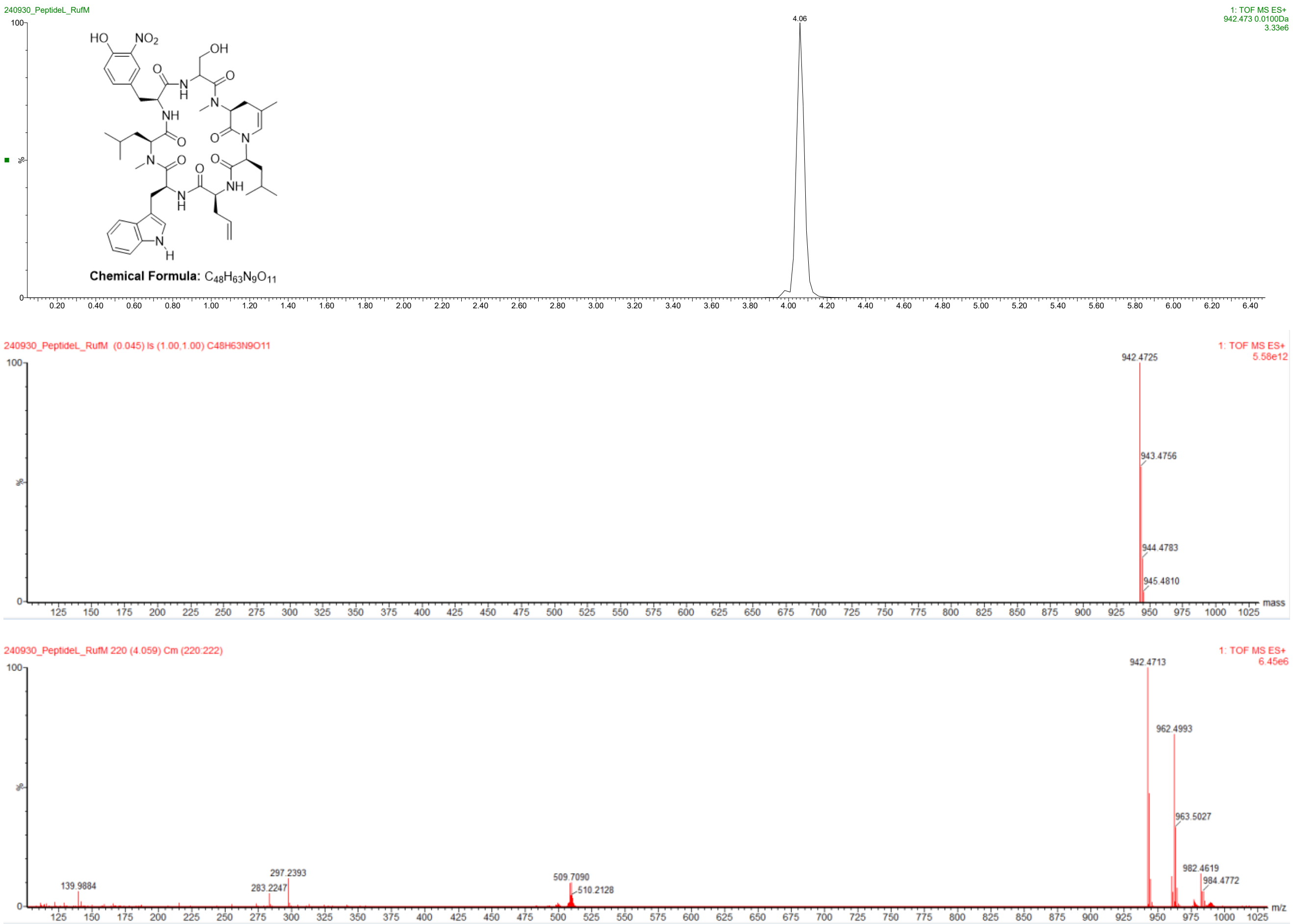

HRMS (ESI) calc. for $C_{48}H_{67}N_9O_{13}$ calc. for $[M+H]^+$: 942.4725 m/z, found: 942.4713 m/z.

*HRMS extracted Ion Chromatogram of Peptide **13c** (top), predicted mass spectrum (middle) measured mass spectrum (bottom)*

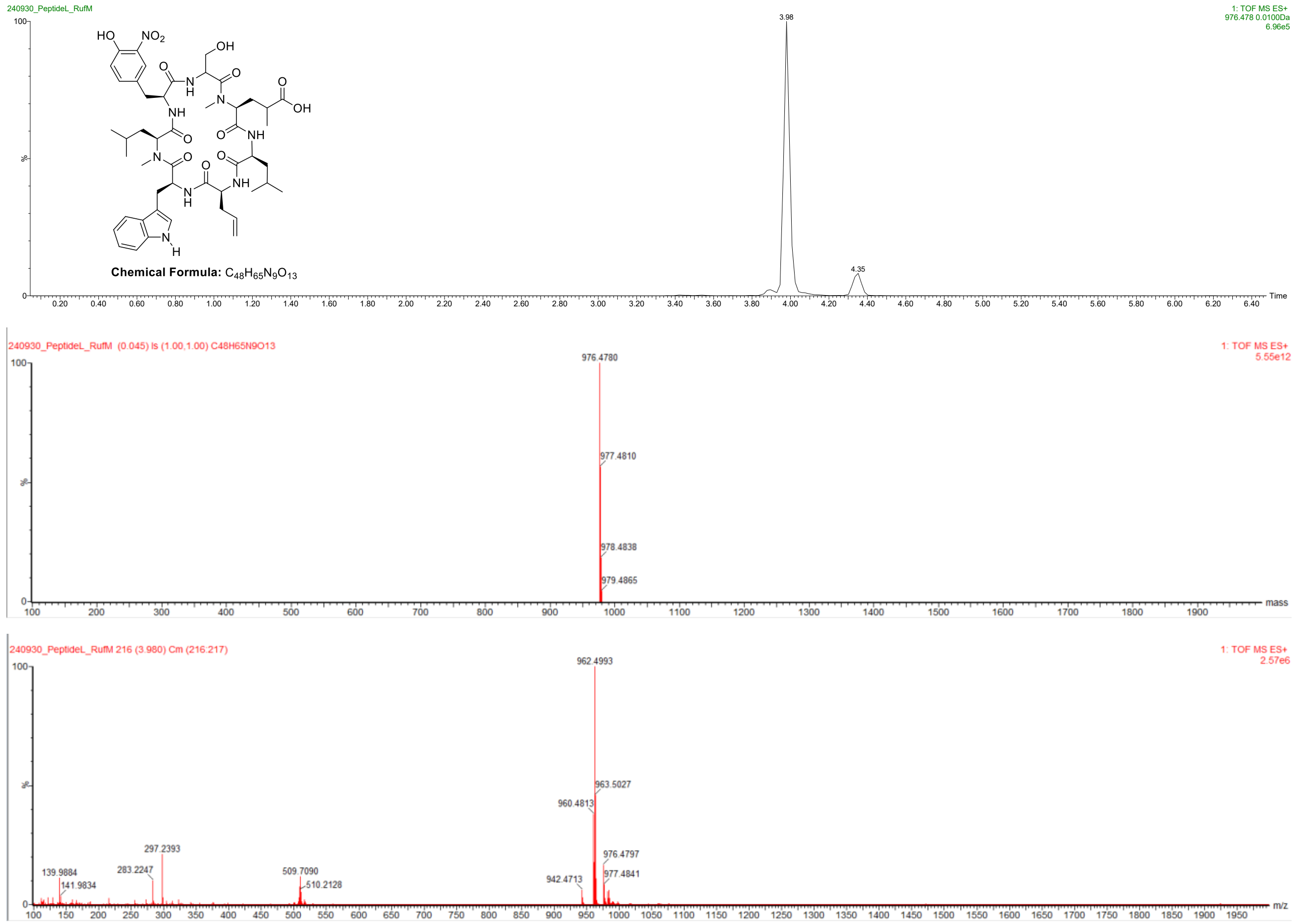

HRMS (ESI) calc. for $C_{48}H_{67}N_9O_{13}$ calc. for $[M+H]^+$: 976.4780 m/z, found: 976.4797 m/z.

*HRMS of Peptide **14** (top), predicted mass spectrum (middle) measured mass spectrum (bottom)*

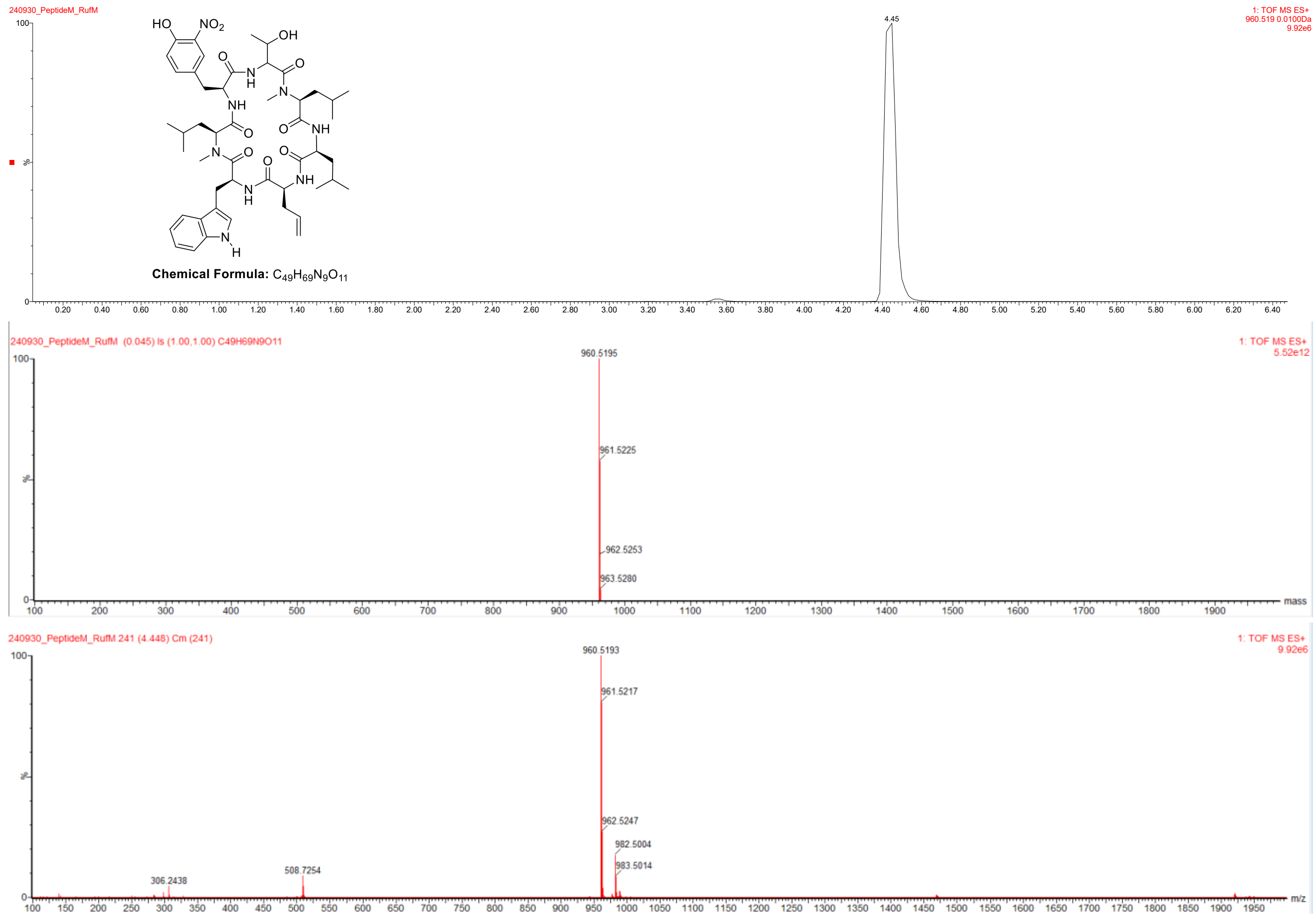

HRMS (ESI) calc. for $C_{54}H_{76}N_9O_{10}$ calc. for $[M+H]^+$: 960.5195 m/z, found: 960.5193 m/z.

*HRMS extracted Ion Chromatogram of Peptide **14a** (top), predicted mass spectrum (middle) measured mass spectrum (bottom)*

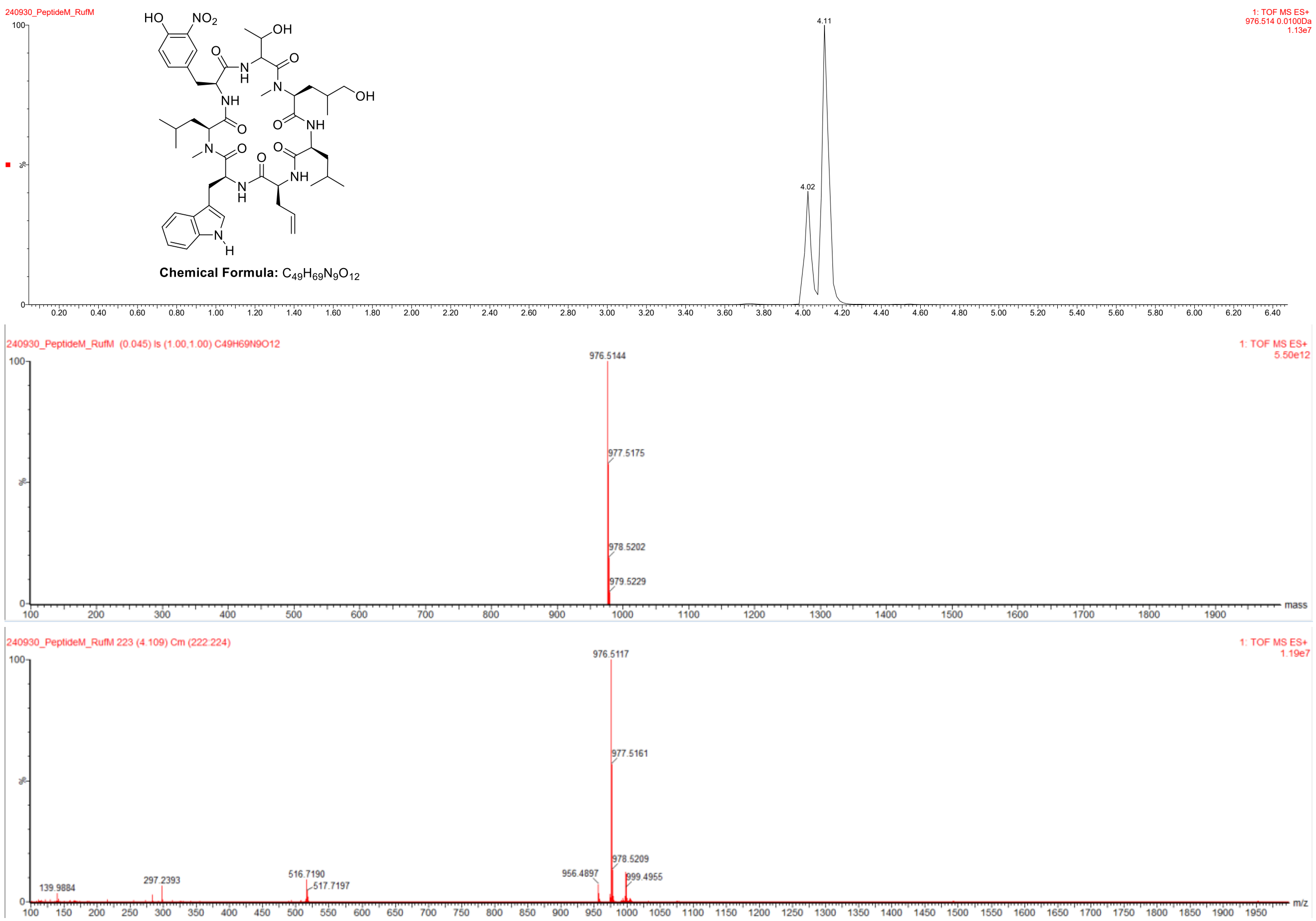

HRMS (ESI) calc. for $C_{54}H_{76}N_9O_{10}$ calc. for $[M+H]^+$: 976.5144 m/z, found: 976.5117 m/z.

*HRMS extracted Ion Chromatogram of Peptide **14e** (top), predicted mass spectrum (middle) measured mass spectrum (bottom)*

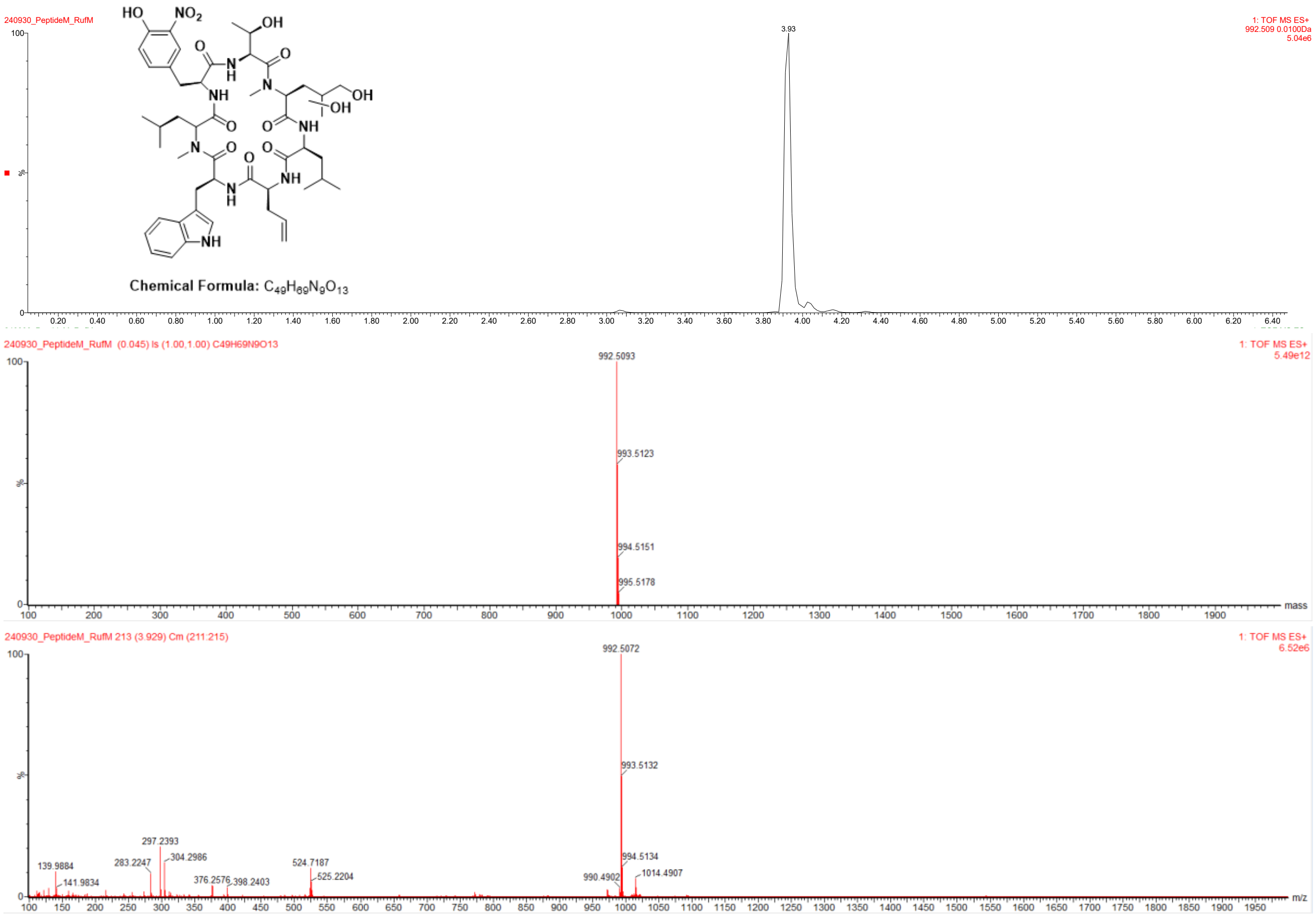

HRMS (ESI) calc. for $C_{54}H_{76}N_9O_{10}$ calc. for $[M+H]^+$: 992.5093 m/z, found: 992.5072 m/z.

HRMS extracted Ion Chromatogram of Peptide ***14b/b**** *(top), predicted mass spectrum (middle) measured mass spectrum (bottom)*

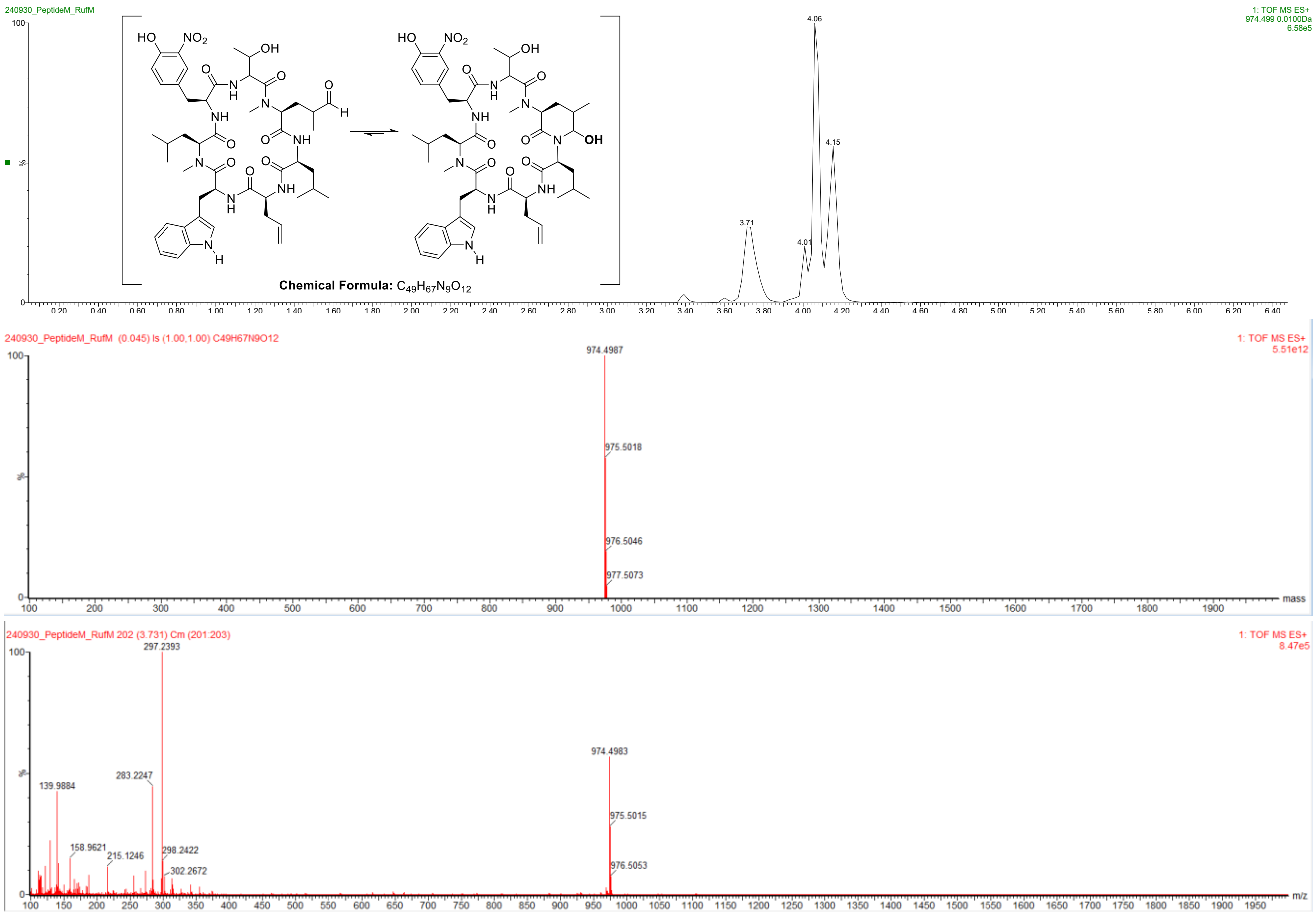

HRMS (ESI) calc. for $C_{54}H_{76}N_{9}O_{10}$ calc. for $[M+H]^{+}$: 974.4987 m/z, found: 974.4983 m/z.

*HRMS extracted Ion Chromatogram of Peptide **14d** (top), predicted mass spectrum (middle) measured mass spectrum (bottom)*

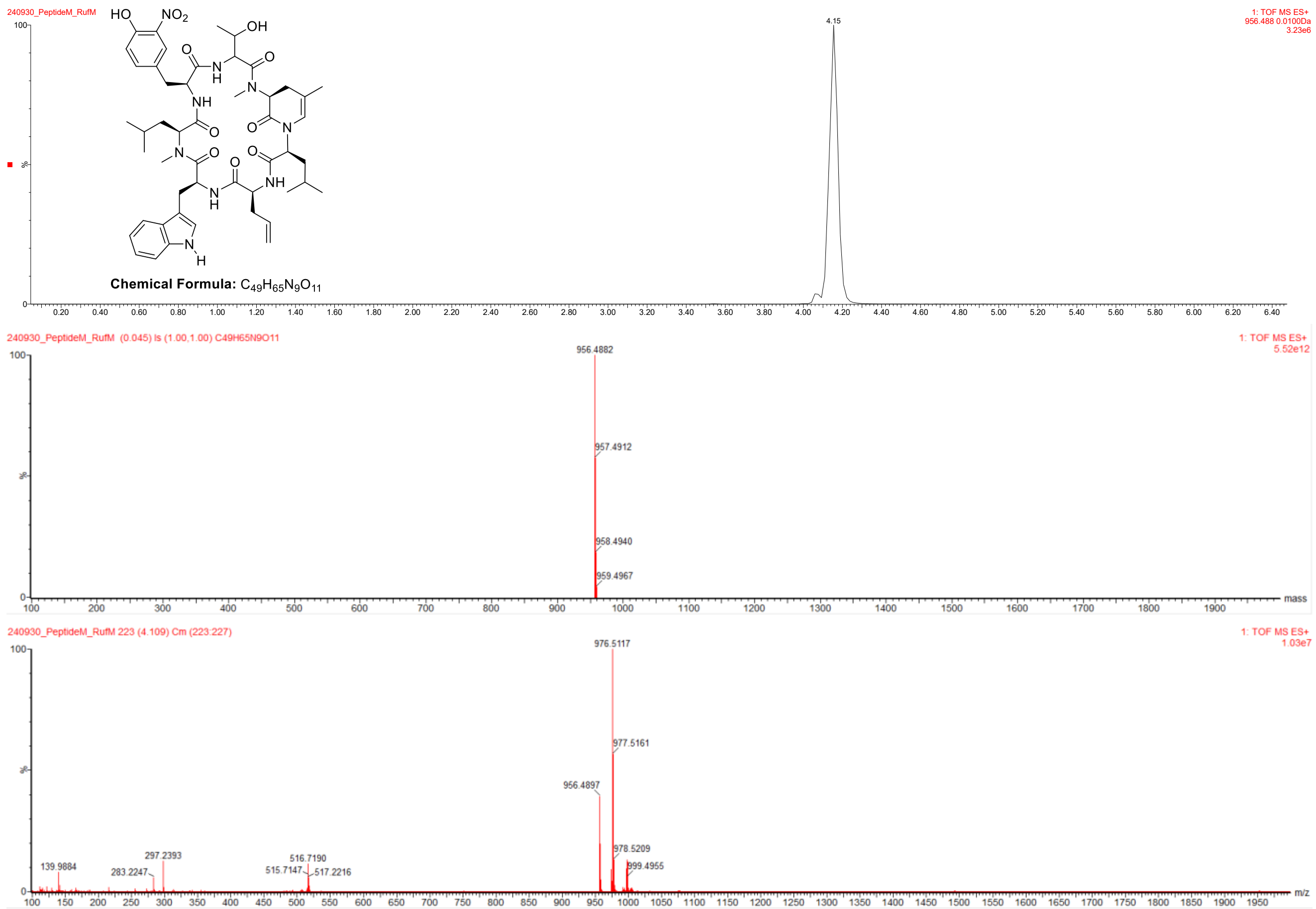

HRMS (ESI) calc. for $C_{54}H_{76}N_9O_{10}$ calc. for $[M+H]^+$: 956.4882 m/z, found: 956.4897 m/z.

*HRMS extracted Ion Chromatogram of Peptide **14c** (top), predicted mass spectrum (middle) measured mass spectrum (bottom)*

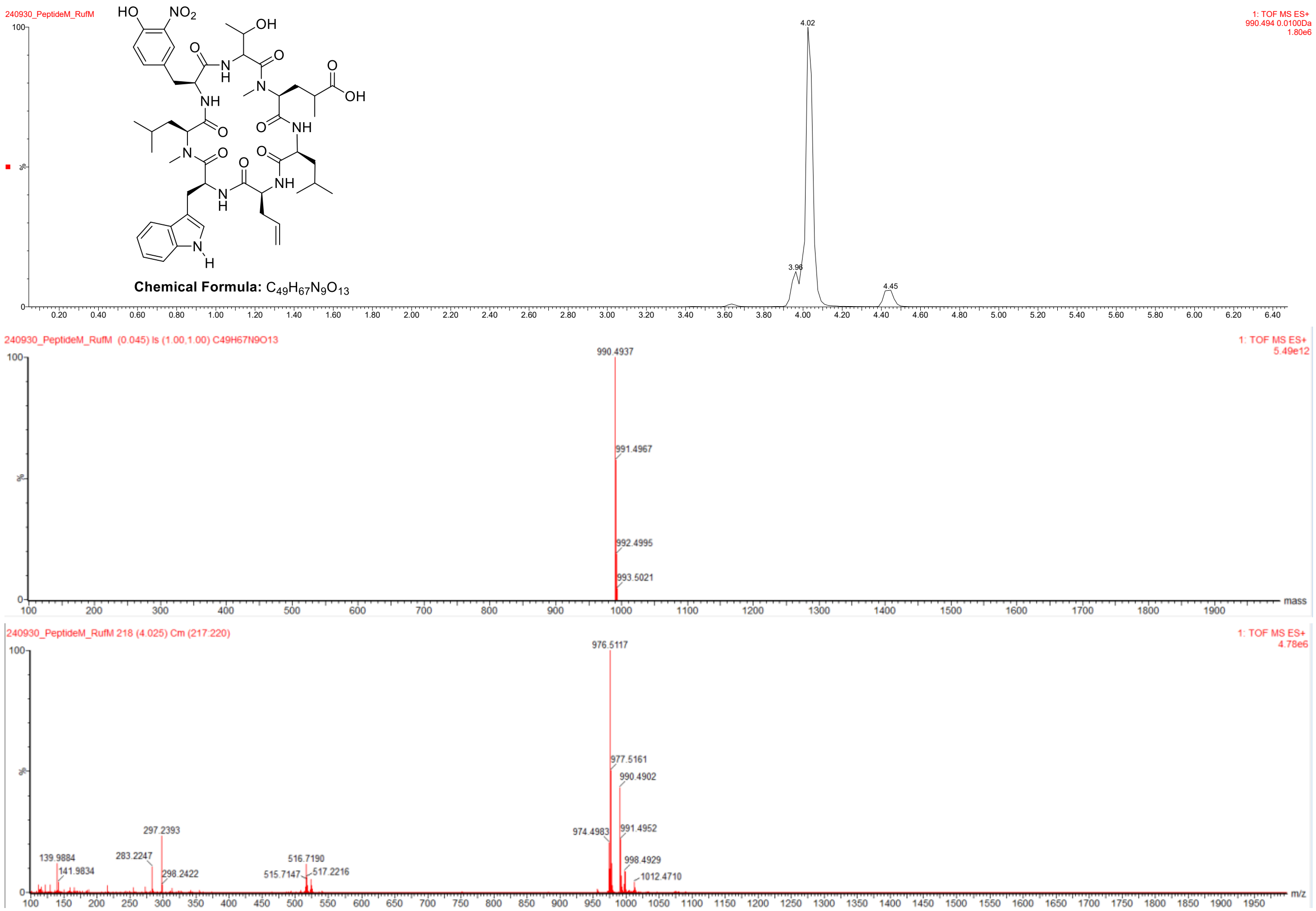

HRMS (ESI) calc. for $C_{54}H_{76}N_9O_{10}$ calc. for $[M+H]^+$: 990.4937 m/z, found: 990.4902 m/z.

*HRMS of Linear Peptide **15** predicted mass spectrum (top), measured MS^E (bottom)*

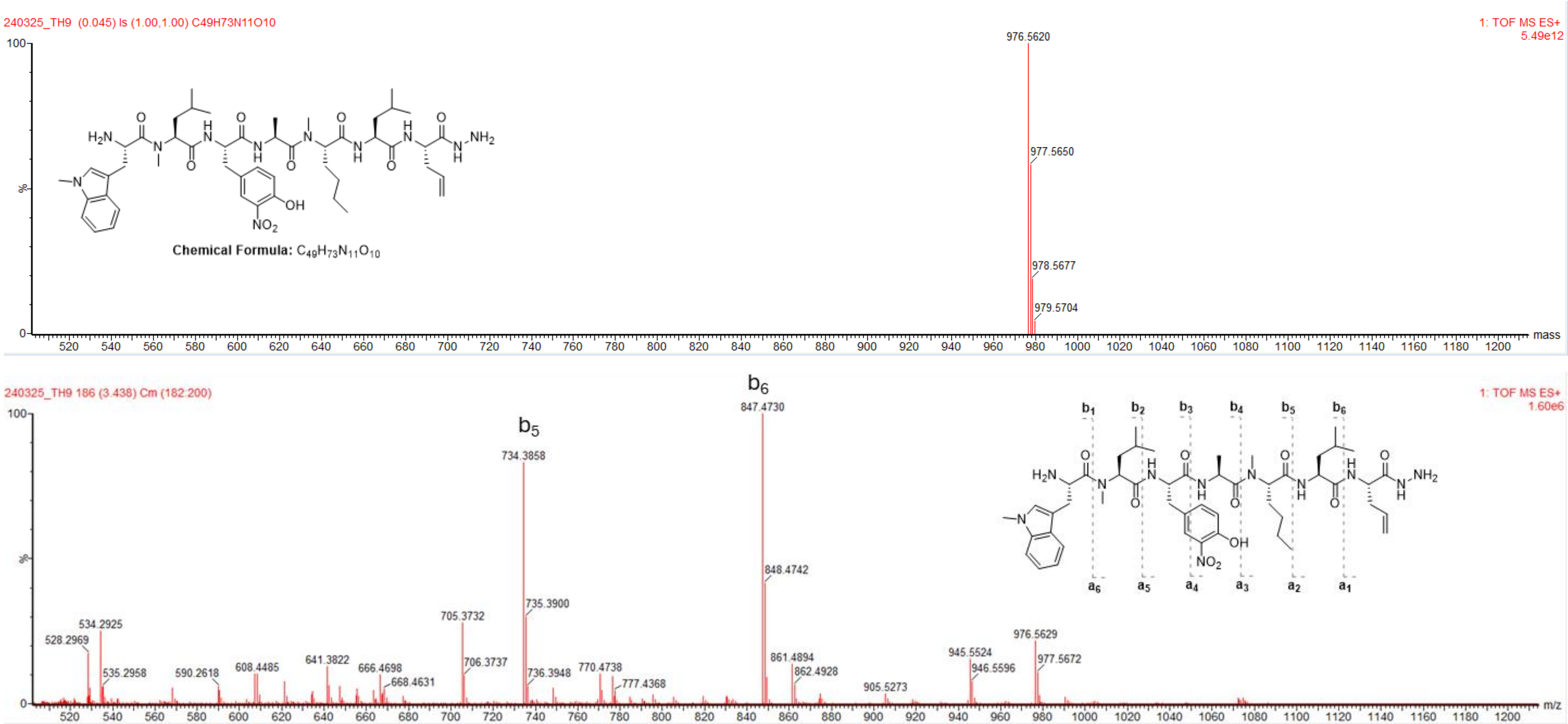

HRMS (ESI) calc. for $C_{53}H_{73}N_9O_{10}$ calc. for $[M+H]^+$: 976.5620 m/z, found: 976.5629 m/z.

*HRMS of Peptide **15** (top), predicted mass spectrum (middle) measured mass spectrum (bottom)*

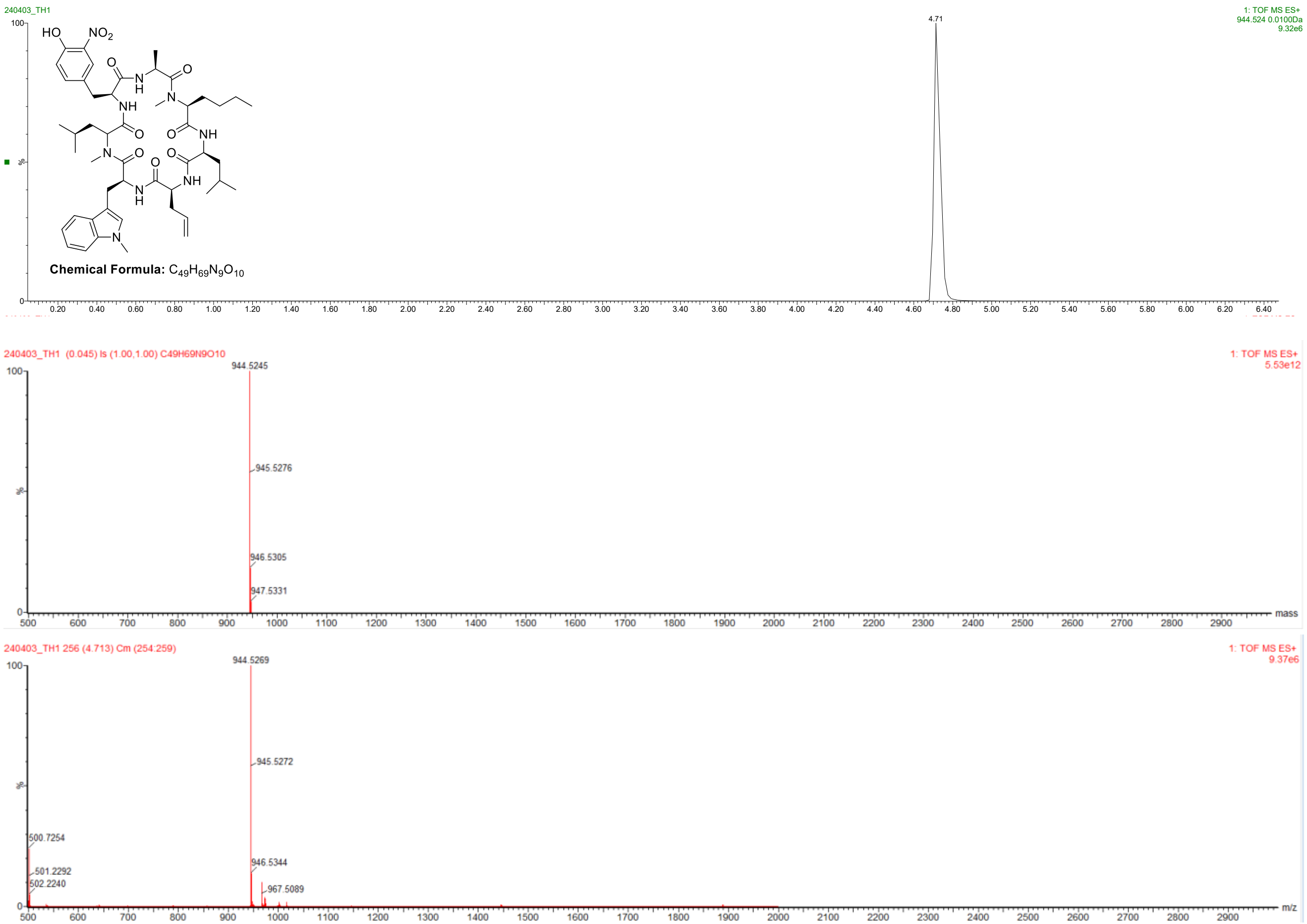

HRMS (ESI) calc. for $C_{53}H_{73}N_9O_{10}$ calc. for $[M+H]^+$: 944.5245 m/z, found: 944.5269 m/z.

*HRMS Extracted Ion Chromatogram of Peptide **15a** (top), predicted mass spectrum (middle) measured mass spectrum (bottom)*

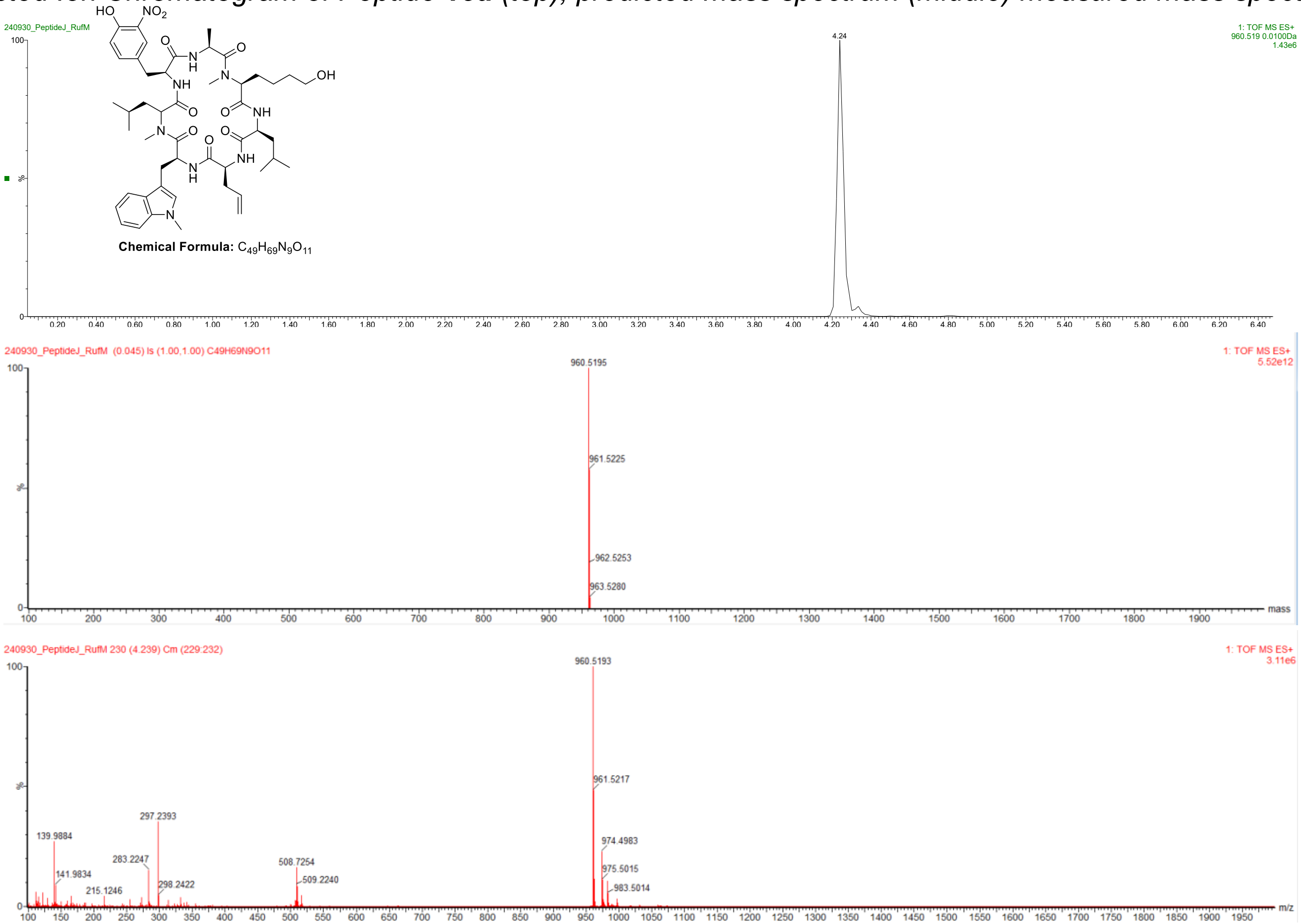

HRMS (ESI) calc. for $C_{49}H_{69}N_9O_{11}$ calc. for $[M+H]^+$: 960.5195 m/z, found: 960.5193 m/z.

HRMS Extracted Ion Chromatogram of Peptide ***15b*** *(top), predicted mass spectrum (middle) measured mass spectrum (bottom)*

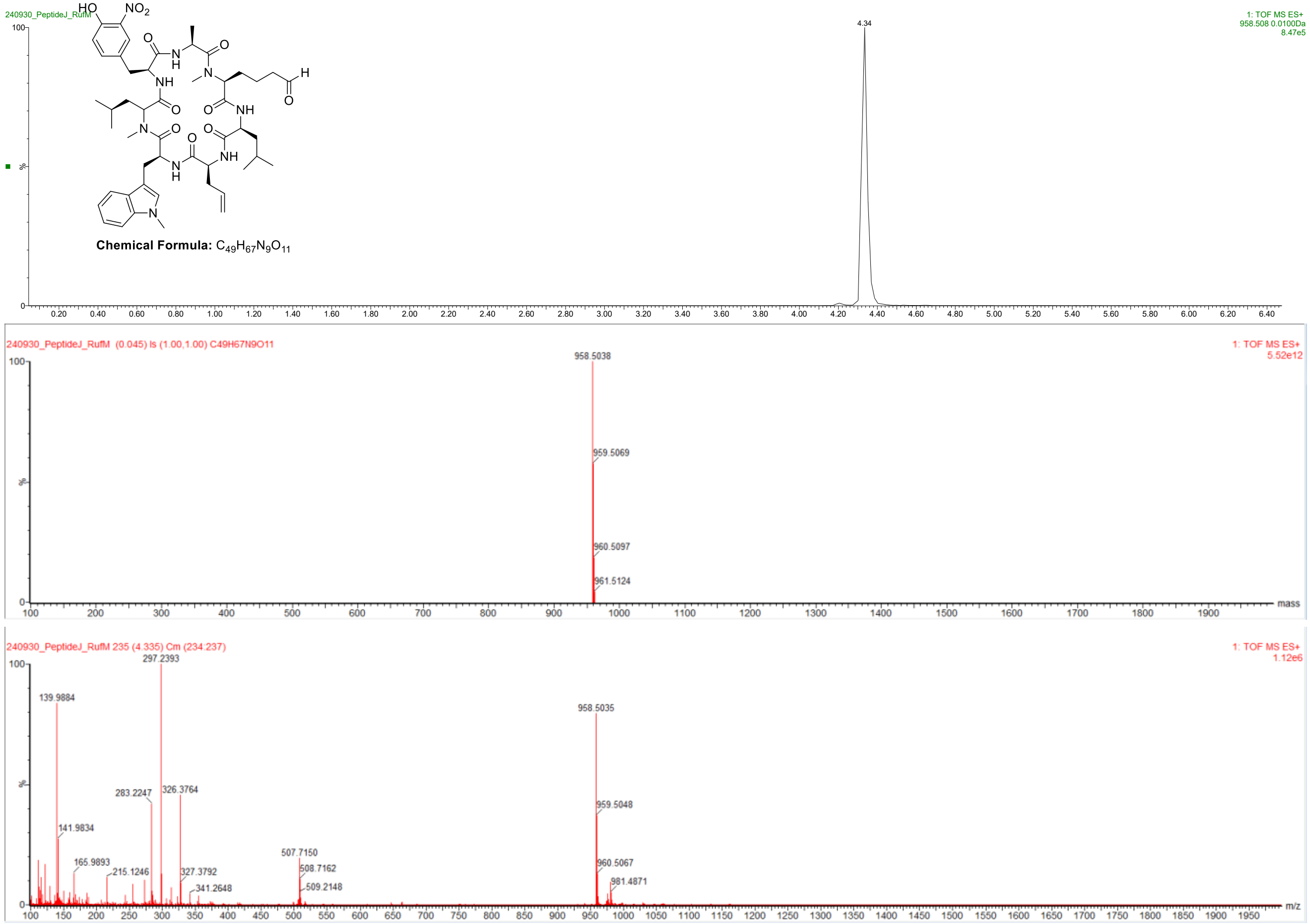

HRMS (ESI) calc. for $C_{49}H_{67}N_9O_{11}$ calc. for $[M+H]^+$: 958.5038 m/z, found: 958.5035 m/z.

*HRMS Extracted Ion Chromatogram of Peptide **15c** (top), predicted mass spectrum (middle) measured mass spectrum (bottom)*

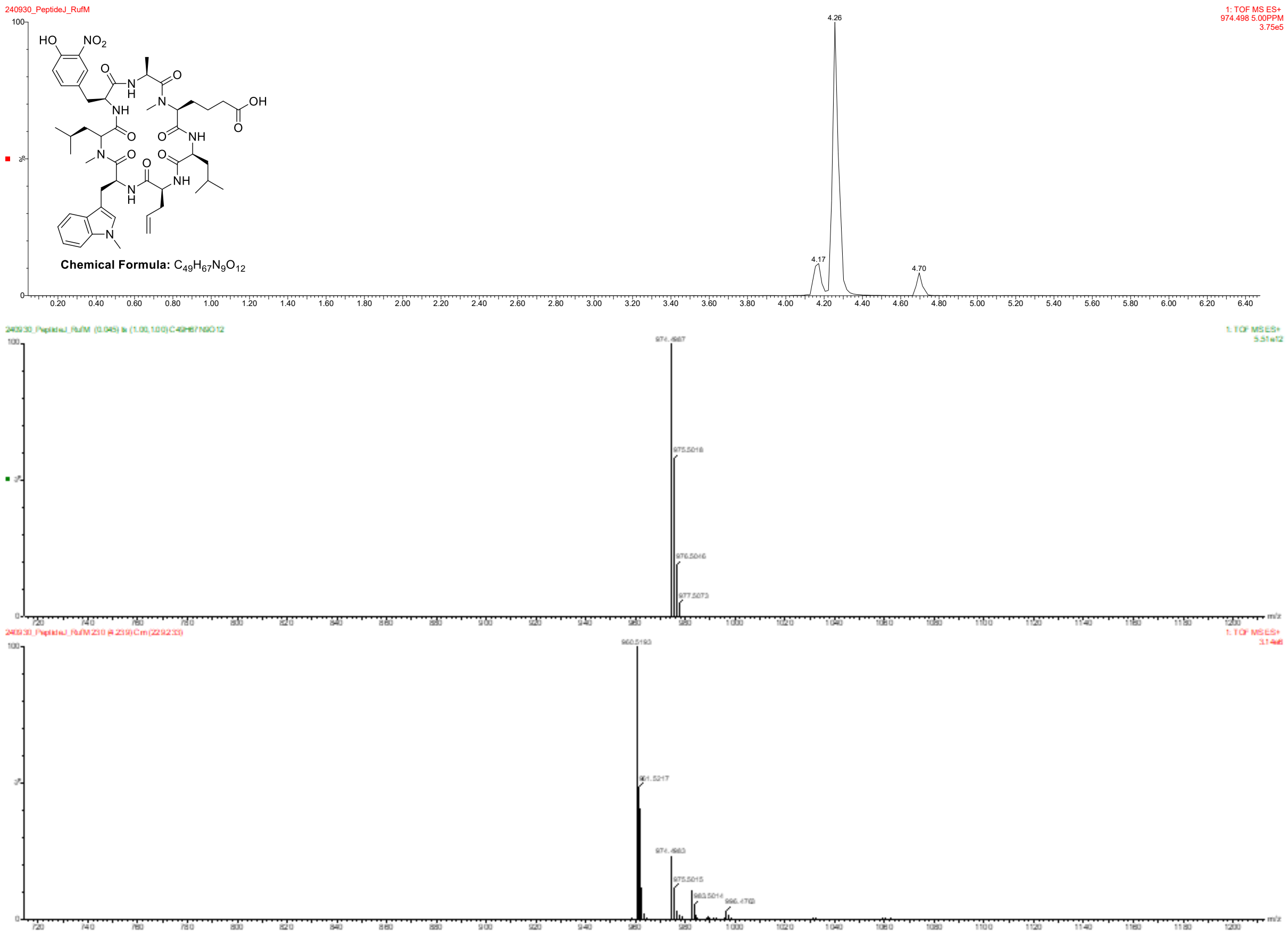

HRMS (ESI) calc. for $C_{49}H_{67}N_9O_{12}$ calc. for $[M+H]^+$: 974.4987 m/z, found: 974.4983 m/z.

*HRMS Linear Peptide **16** (predicted mass spectrum (top) measured (bottom)).*

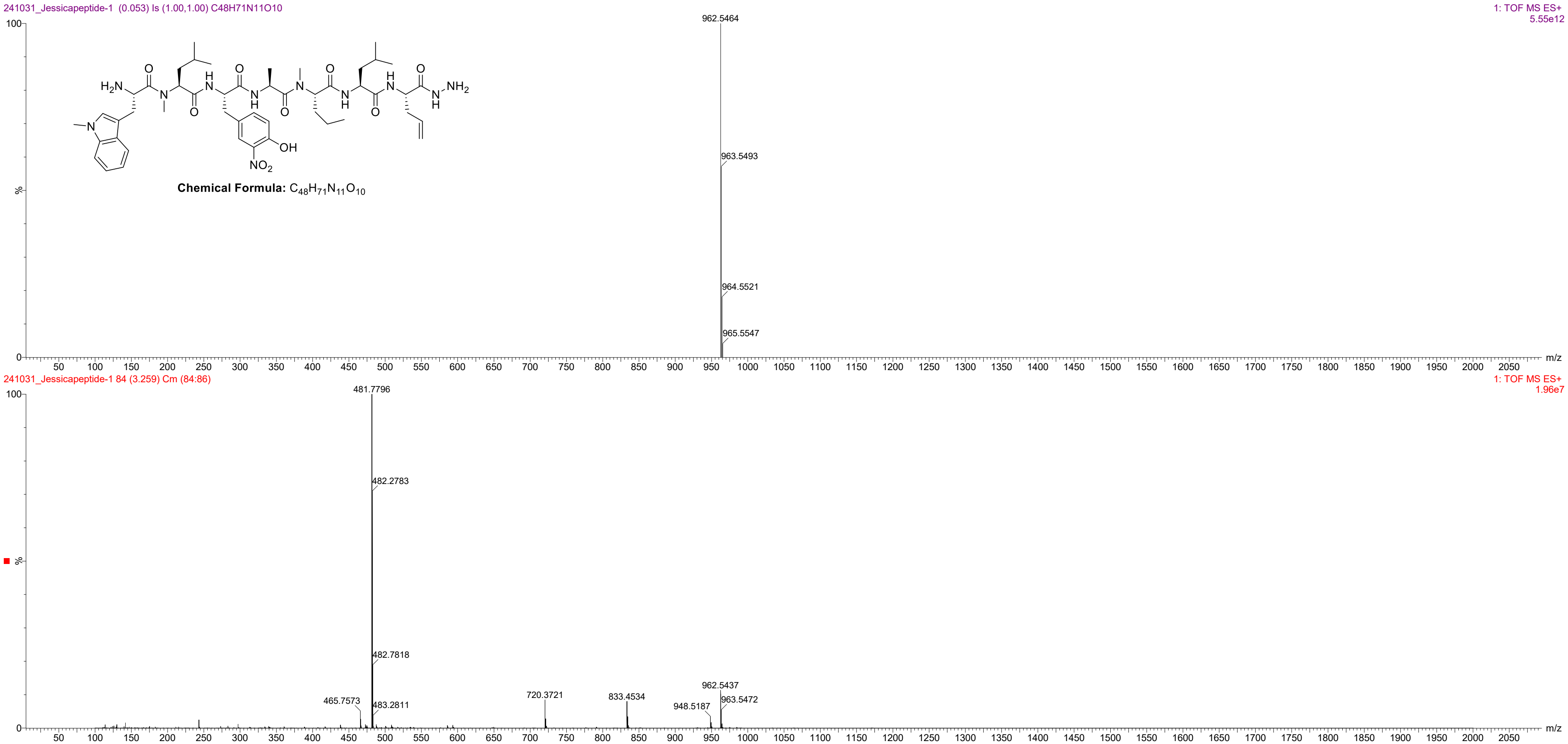

HRMS (ESI) calc. for $C_{48}H_{71}N_{11}O_{10}$ calc. for $[M+H]^+$: 962.5464 m/z, found: 962.5437 m/z.

*MS^e of Linear Peptide **16***

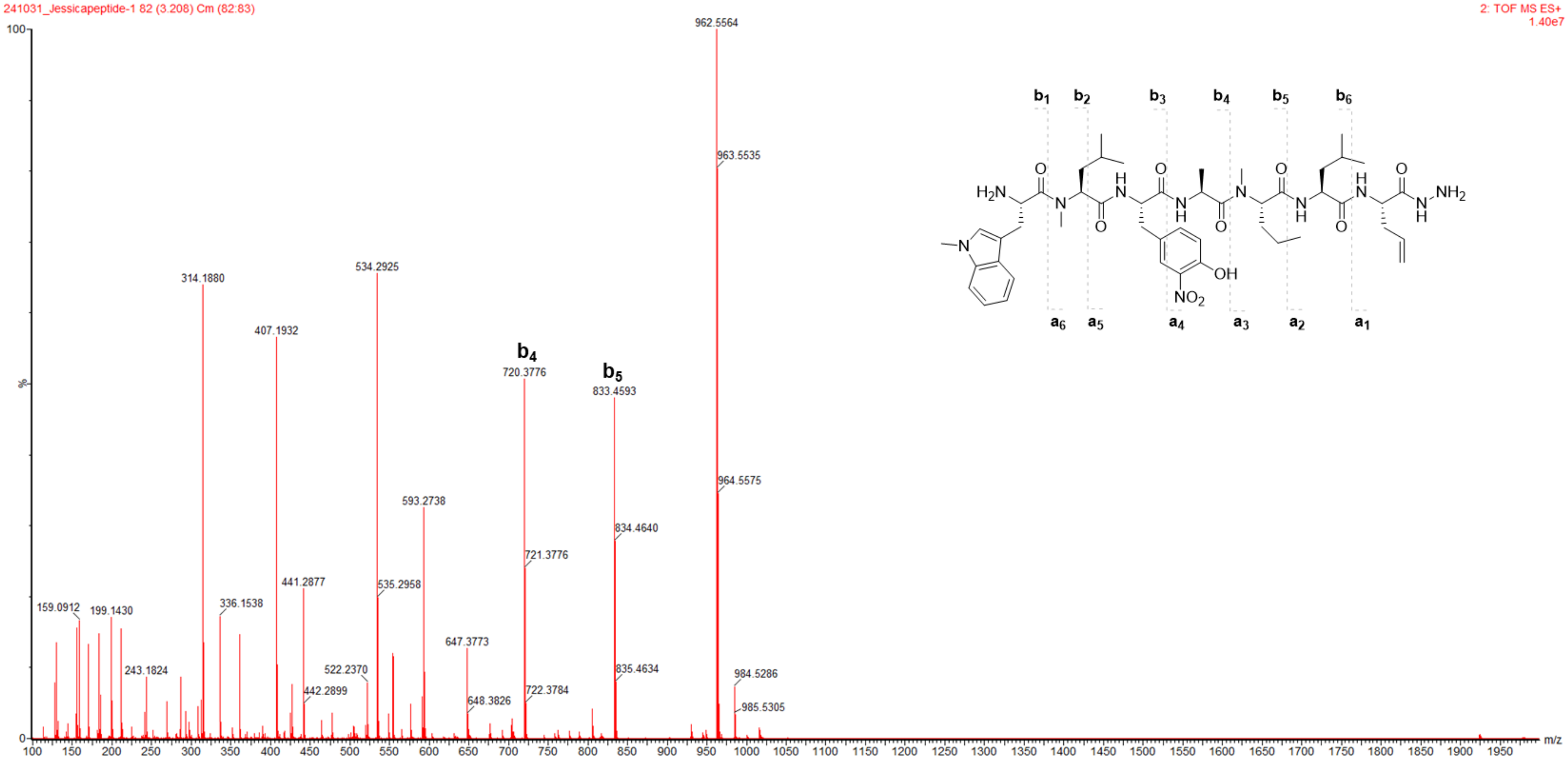

*HRMS of Peptide **16**(top), predicted mass spectrum (middle) measured mass spectrum (bottom)*

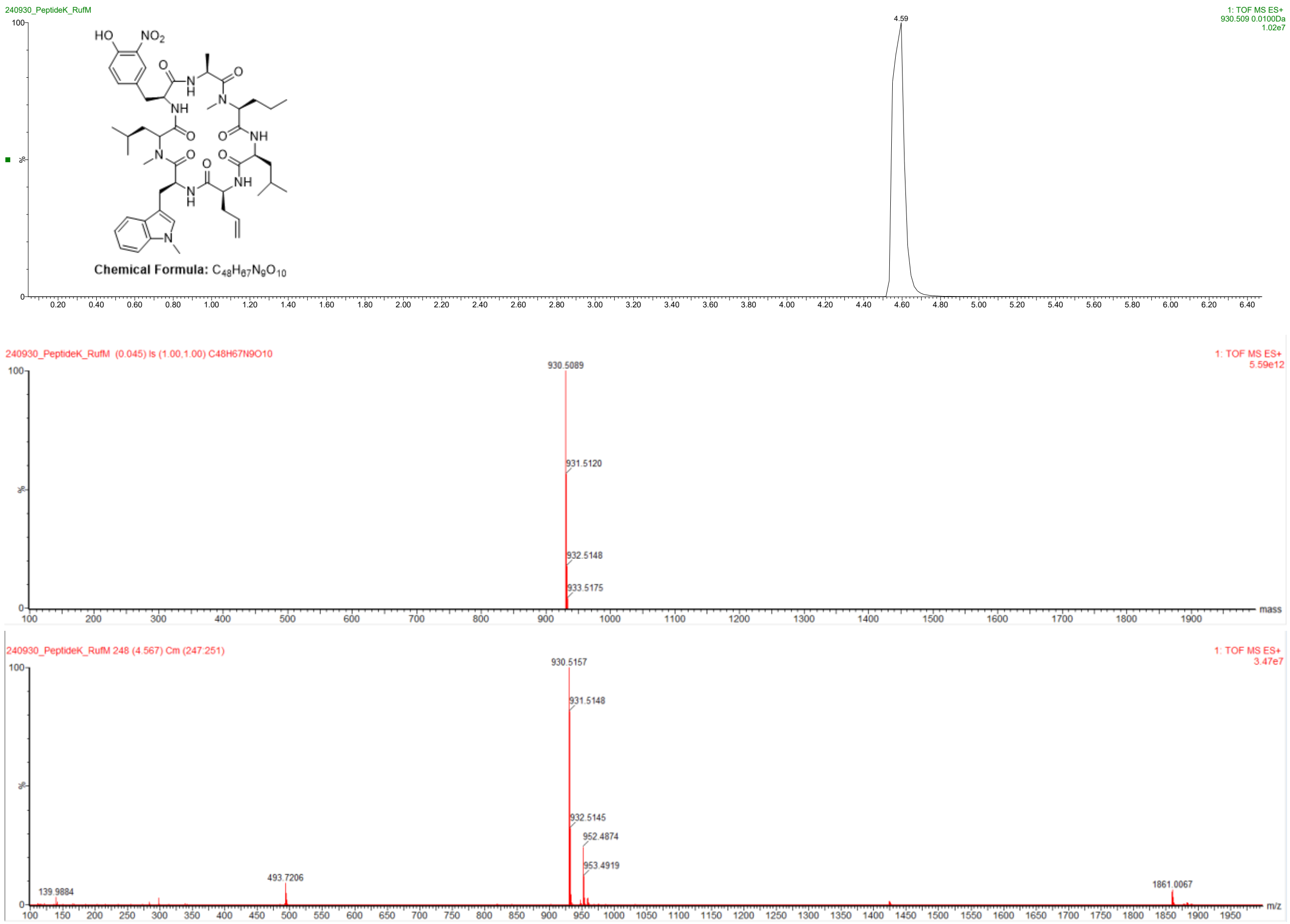

HRMS (ESI) calc. for $C_{48}H_{67}N_9O_{10}$ calc. for $[M+H]^+$: 930.5089 m/z, found: 930.5157 m/z.

*HRMS extracted Ion Chromatogram of Peptide **16a**(top), predicted mass spectrum (middle) measured mass spectrum (bottom)*

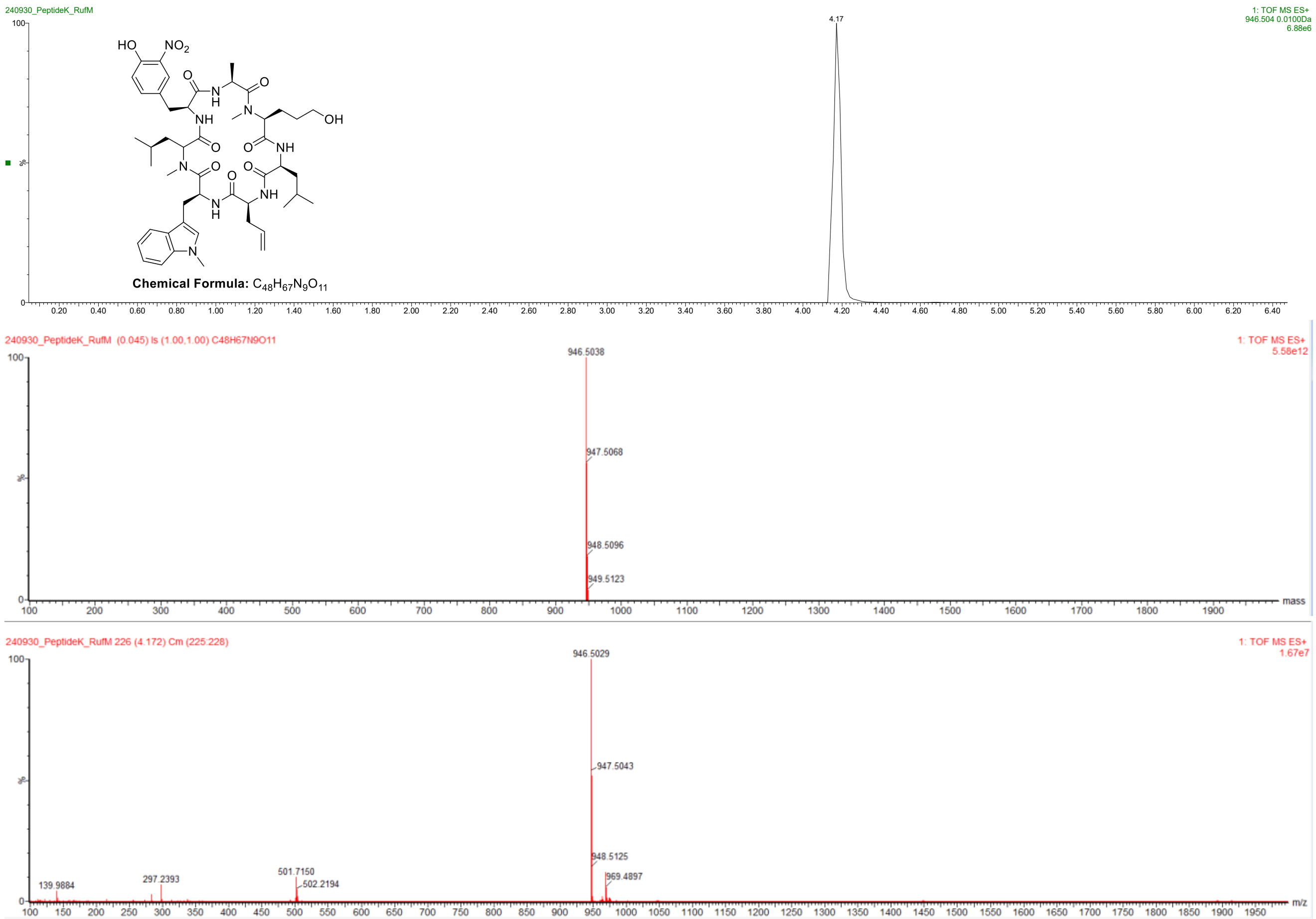

HRMS (ESI) calc. for $C_{54}H_{76}N_9O_{10}$ calc. for $[M+H]^+$: 946.5038 m/z, found: 946.5029 m/z.

MS^e of Linear Peptide 17

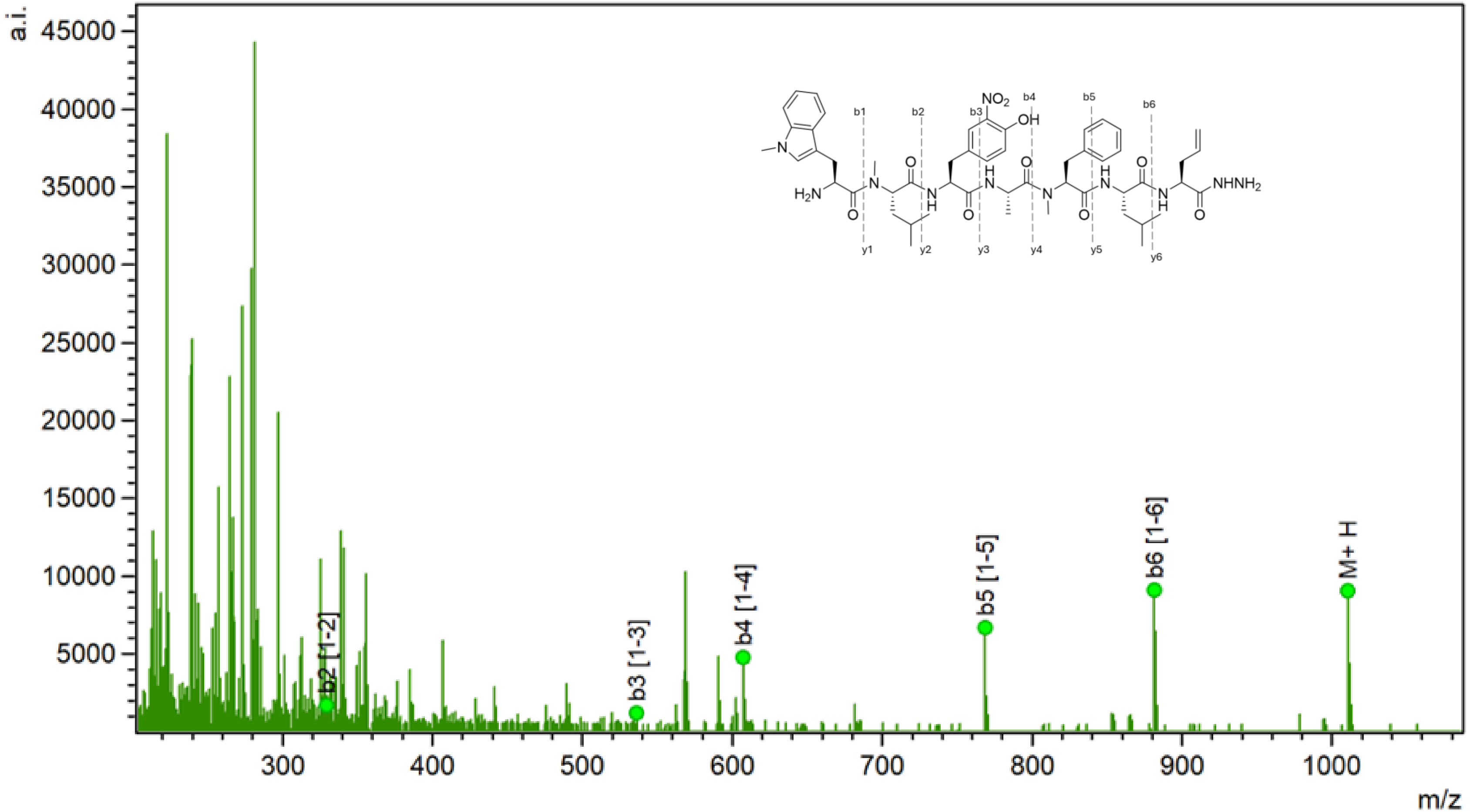

*HRMS Extracted Ion Chromatogram of Linear Peptide **17** top), predicted mass spectrum (middle) measured mass spectrum (bottom)*

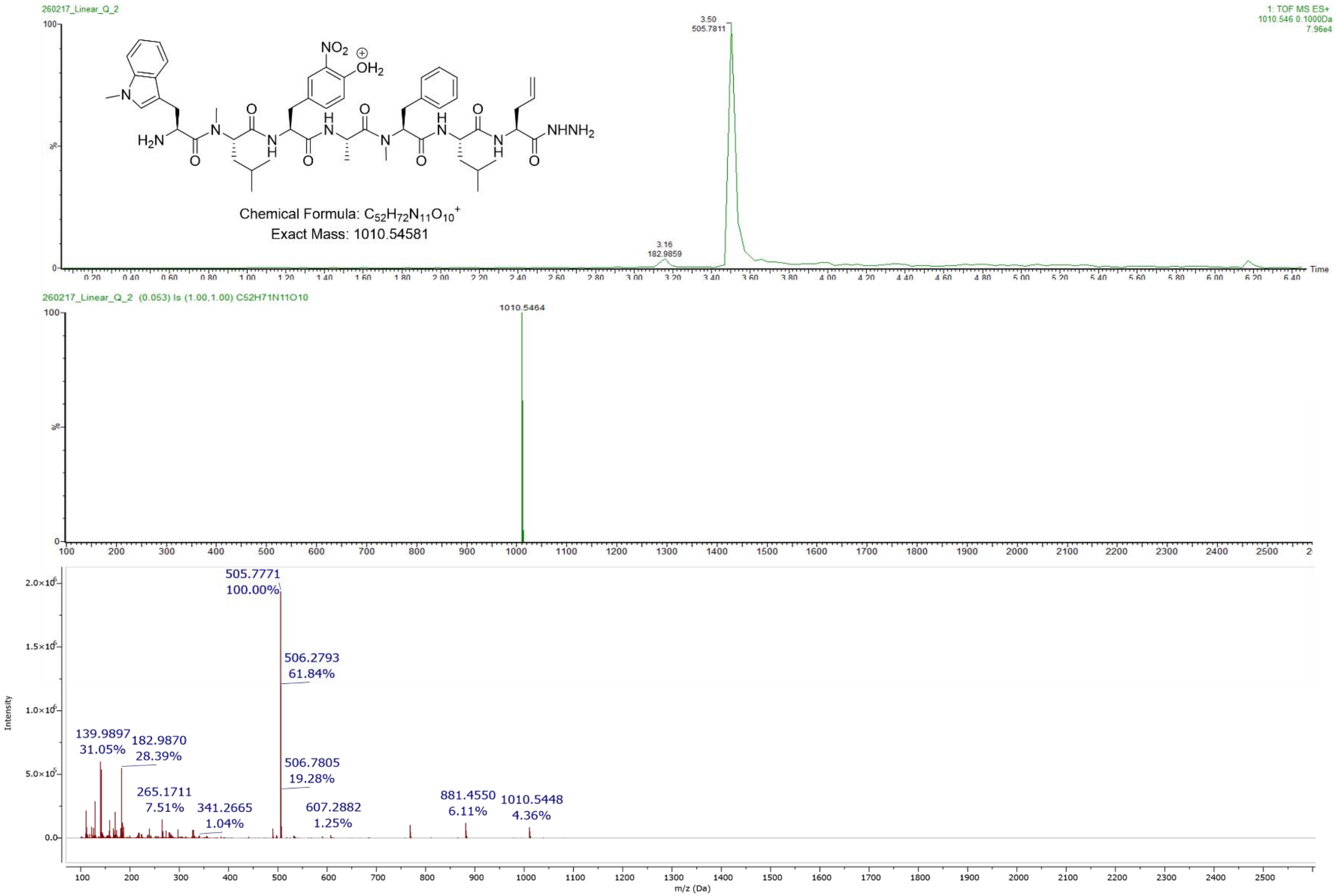

HRMS (ESI) calc. for $C_{52}H_{71}N_{11}O_{10}$ calc. for $[M+H]^{+}$: 1010.5464 m/z, found: 1010.5448 m/z.

*HRMS Extracted Ion Chromatogram of Cyclic Peptide **17** top), predicted mass spectrum (middle) measured mass spectrum (bottom)*

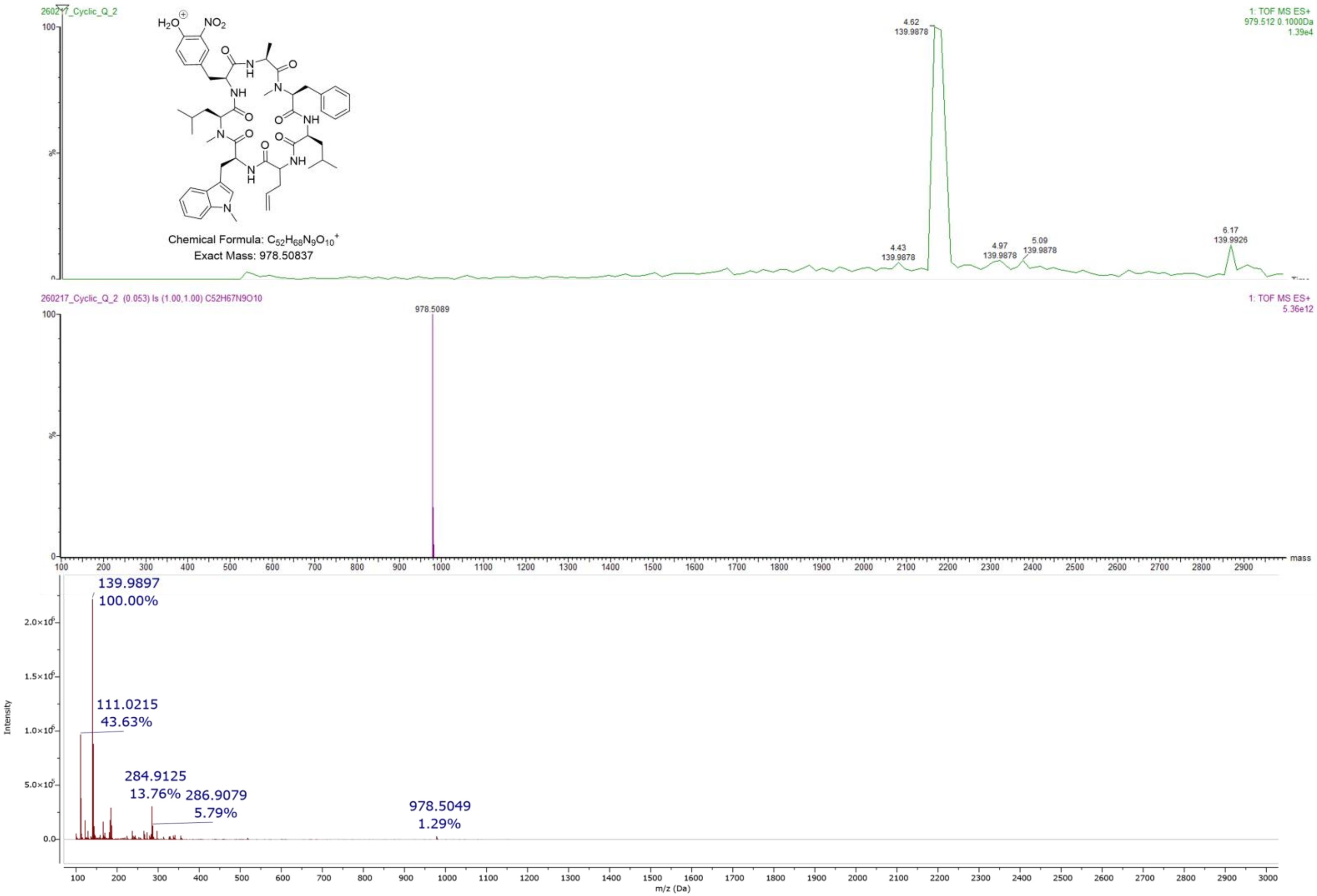

HRMS (ESI) calc. for $C_{52}H_{67}N_9O_{10}$ calc. for $[M+H]^+$: 978.5089 m/z, found: 978.5049 m/z.

HRMS Extracted Ion Chromatogram of Linear Peptide ***18*** *top), predicted mass spectrum (middle) measured mass spectrum (bottom)*

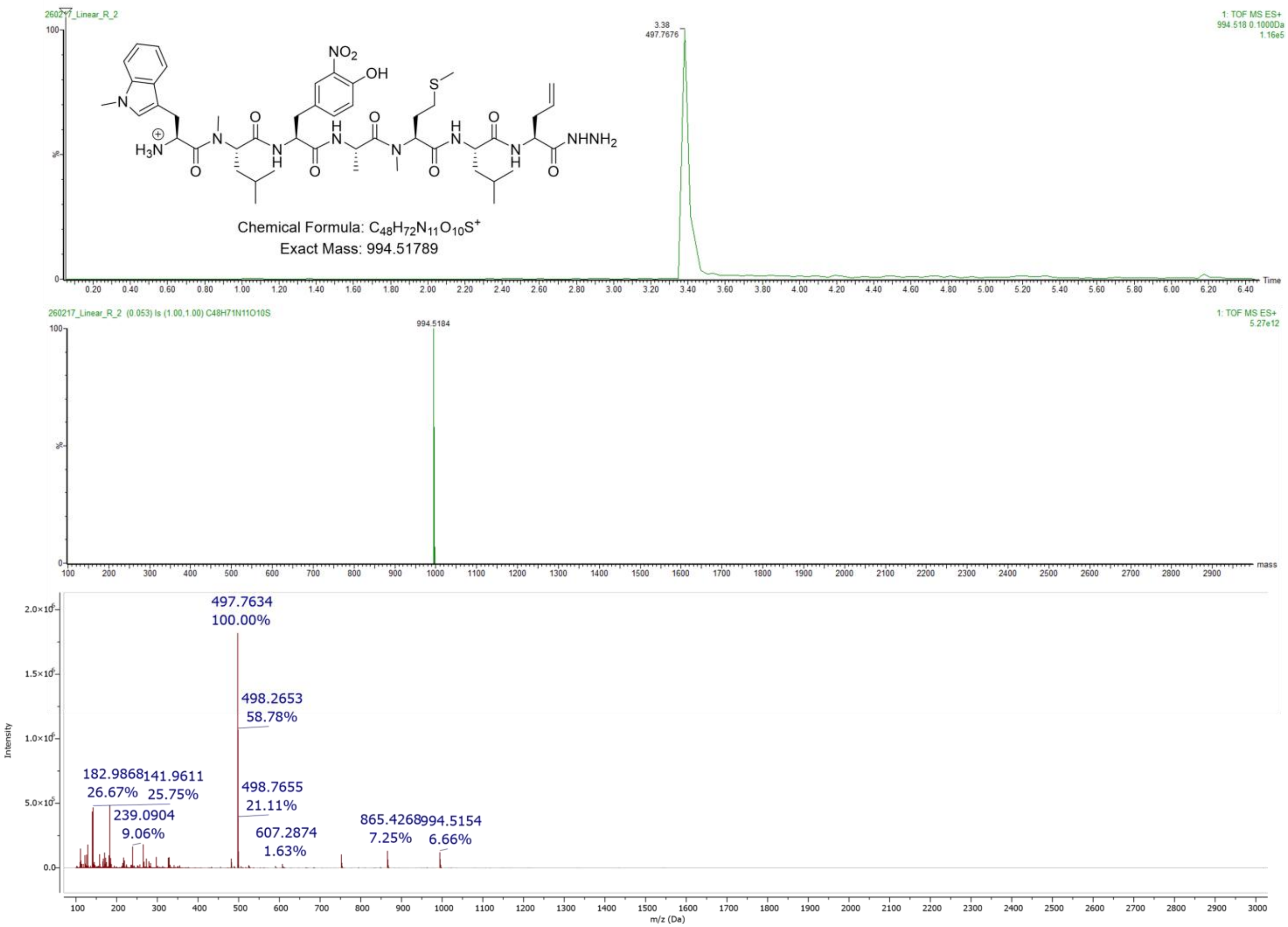

HRMS (ESI) calc. for $C_{48}H_{71}N_{11}O_{10}S$ calc. for $[M+H]^+$: 994.5184 m/z, found: 994.5154 m/z.

*MS^e of Linear Peptide **18***

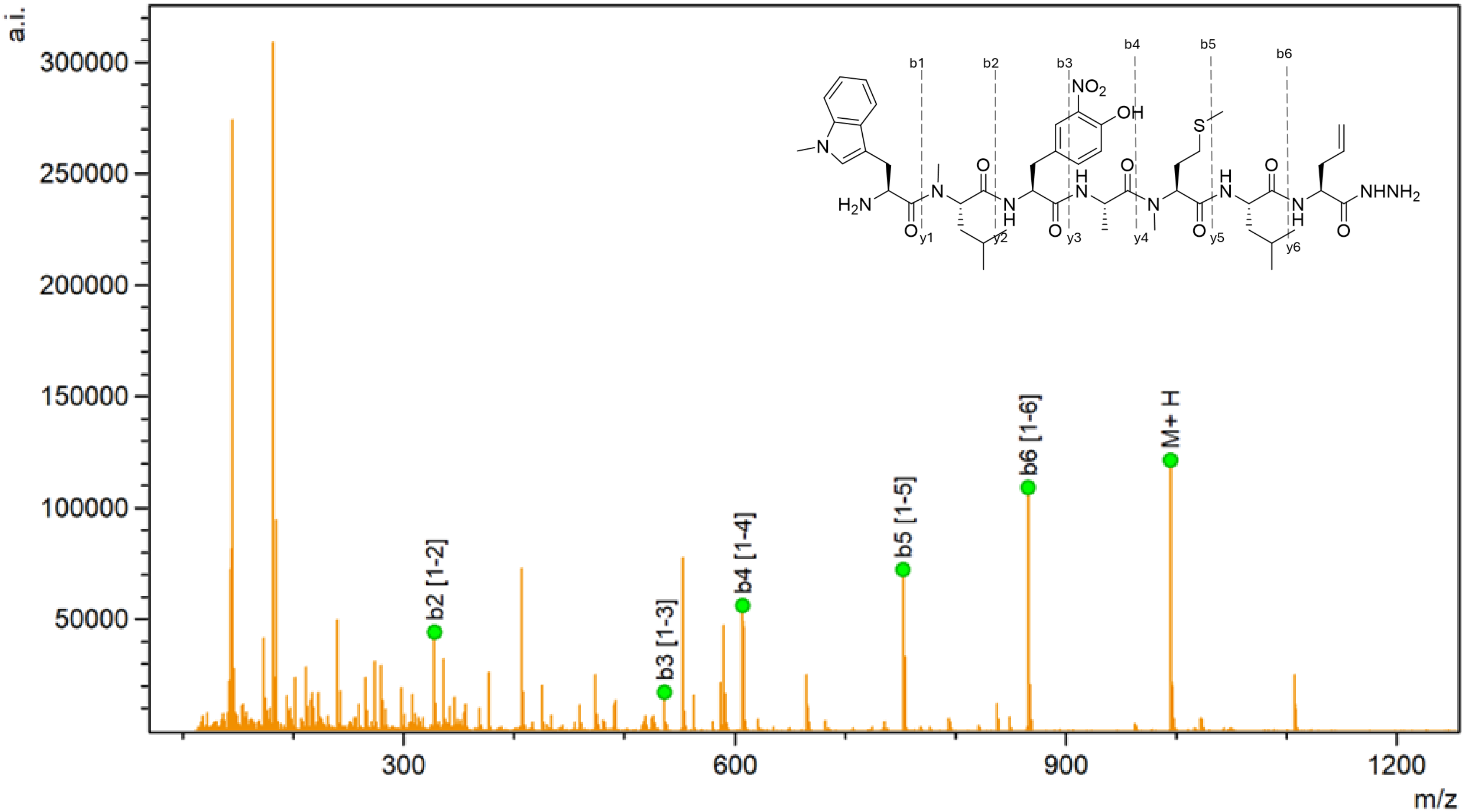

*HRMS Extracted Ion Chromatogram of Cyclic Peptide **18** top), predicted mass spectrum (middle) measured mass spectrum (bottom)*

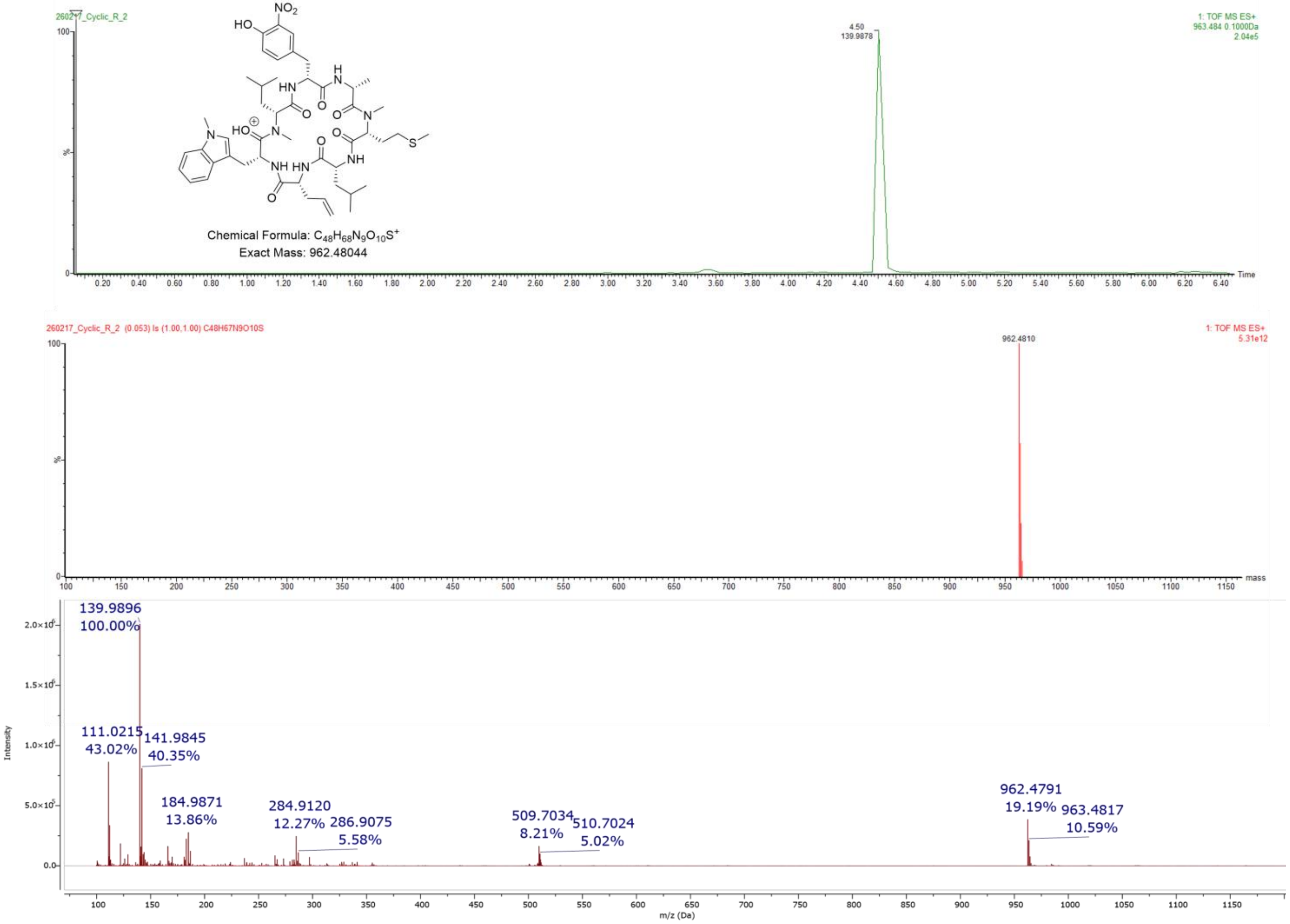

HRMS (ESI) calc. for $C_{48}H_{67}N_{9}O_{10}S$ calc. for $[M+H]^{+}$: 962.4810 m/z, found: 962.4795 m/z.
